# Supplementary material for: Multiplex Target-Redundant RT-LAMP for Robust Detection of SARS-CoV-2 Using Fluorescent Universal Displacement Probes
Source: Microbiol Spectr. 2022 Jun 16;10(4):e01583-21. doi: 10.1128/spectrum.01583-21 (PMC9430505; doi:10.1128/spectrum.01583-21)
Supplement: Supplemental file 1 — Supplemental material. Download spectrum.01583-21-s0001.pdf, PDF file, 3.0 MB [file spectrum.01583-21-s0001.pdf]

Supplementary Information

Methods:

Performance against variant sequences with known mismatch mutations – run conditions

Reactions for individual sub assays were run as described for mRT-LAMP, with all other non-essential primers and probes omitted. Due to the IAC being tied to NC1, it was omitted from reactions to avoid cross reaction and performance bias. For the complete multiplex test an IAC UDP with a Cy5 dye was substituted for the TEX615 dye due to materials availability. The reactions we analyzed as before, except the baseline for the IAC UDP with Cy5 was set to 20-50 cycles.

Results:

Supplementary Figure S1: Validation of individual targets in the multiplex assay (challenged with fragments corresponding to individual targets)

A.

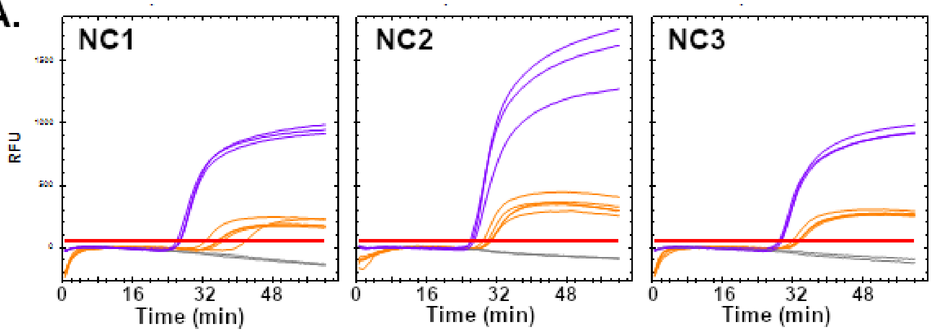

B.

LAMP Target	NC1	NC2	NC3
Target TTD (min)	26.6, 26.8,25.9	25.9, 26.3,26.7	28.8, 29.1,29.4

Supplementary Figure S1: validation of individual SARS-CoV-2 assays. A) The assay was tested with synthetic RNA fragments containing the NC1 sequence, NC2 sequence, or the NC3 sequence as separate fragments. All IAC signals (TEX615) are shown in red, SARS-CoV-2 signals (FAM) from reactions with 0 copies/NTC (n=2) are shown in gray, and SARS-CoV-2 signals (FAM) from reactions with 200 copies (n=3) are shown in blue. **B)** Time for detection of SARS-CoV-2 signals for the assay with individual target inputs from the plots in panel **A**.

Supplementary Figure S2: Effect of commonly used transport medium / contrived specimens on mRT-LAMP

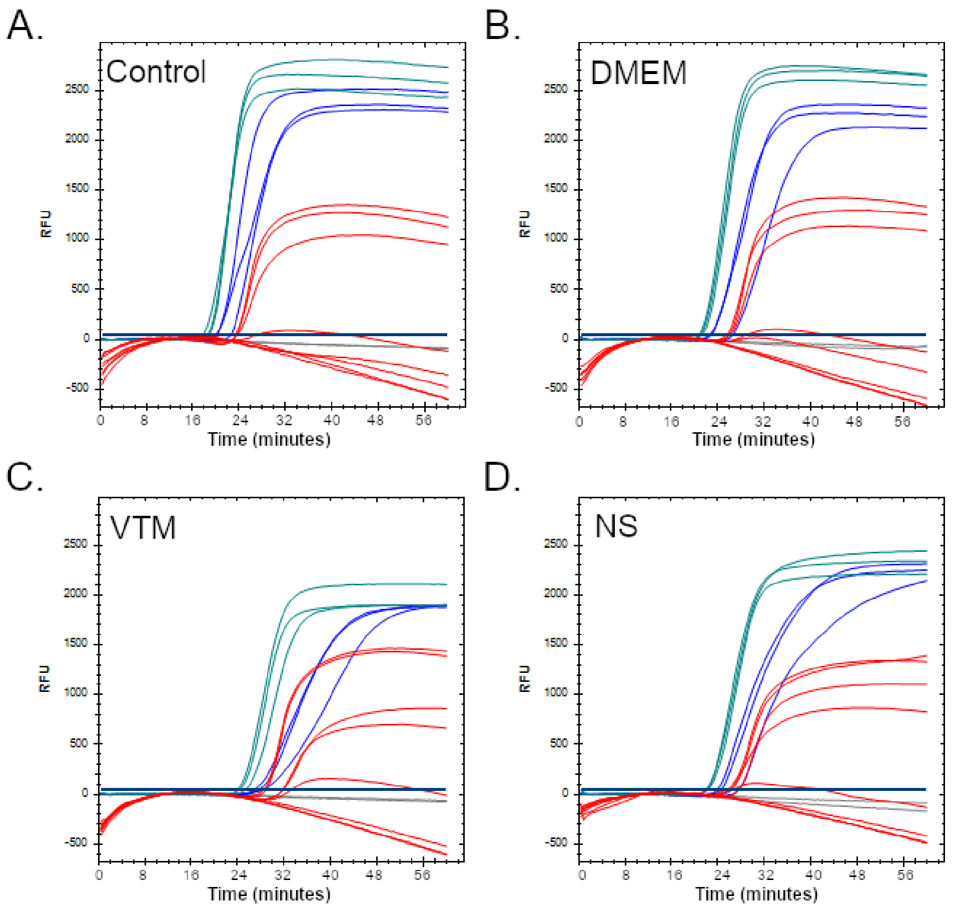

Supplementary Figure S2: Amplification of mock samples with sample transport medium. A) Water control (25% final) B) DMEM (25% final). C) VTM (25% final). D) NS (25% final). All IAC signals are shown in red (1e5 copies). SARS-CoV-2 signals for multiplexed reactions are shown as: 0 copies RNA/ NTC in gray (n=2), 20 copies RNA in blue (n=3), 200 copies RNA in teal (n=3). Thresholds are shown as blue and red lines at 50 RFU.

Supplementary Table S1: Clinical specimen panel

ID	Source	N1 Ct	N1 copies	N2 Ct	N2 copies	RP Ct	PCR result	COVID LAMP 1 (min)	IAC LAMP 1 (min)	COVID LAMP 2 (min)	IAC LAMP 2 (min)	LAMP result	Other pathogens	Original characterization
1	MTB	N/A	N/A	35.3	2.3	26.1	INC	N/A	29.2	N/A	29.0	NEG	No	POS
2	MTB	22.07	18126.0	22.3	13828.0	29.4	POS	17.8	N/A	17.7	N/A	POS	Influenza A	POS
3	MTB	27.7	455.6	26.7	731.0	32.7	POS	20.2	N/A	20.2	N/A	POS	No	POS

4	MTB	19.49	98480.0	19.2	104100.0	33.0	POS	16.1	N/A	16.2	N/A	POS	No	POS
5	MTB	26.59	939.6	26.5	831.2	31.8	POS	19.8	N/A	19.9	N/A	POS	Adenovirus	POS
6	MTB	N/A	N/A	N/A	N/A	28.9	NEG	N/A	28.2	N/A	28.0	NEG	Streptococcus pneumoniae	NEG
7	MTB	N/A	N/A	N/A	N/A	30.8	NEG	N/A	28.1	N/A	28.2	NEG	Streptococcus pneumoniae	NEG
8	MTB	N/A	N/A	N/A	N/A	28.7	NEG	N/A	28.8	N/A	28.3	NEG	Streptococcus pneumoniae	NEG
9	MTB	N/A	N/A	N/A	N/A	N/A	IND	N/A	28.5	N/A	28.0	NEG	No	NEG
10	MTB	N/A	N/A	N/A	N/A	26.9	NEG	N/A	28.2	N/A	28.4	NEG	Human parechovirus	NEG
11	MTB	N/A	N/A	N/A	N/A	N/A	IND	N/A	27.9	N/A	28.4	NEG	Rhinovirus, Influenza B, Adenovirus	NEG
12	MTB	N/A	N/A	N/A	N/A	33.7	NEG	N/A	28.4	N/A	29.4	NEG	Rhinovirus, Influenza B	NEG
13	MTB	24.9	2838.0	25.2	1972.2	24.1	POS	19.3	N/A	19.2	N/A	POS	Streptococcus pneumoniae	POS
14	MTB	N/A	N/A	N/A	N/A	30.6	NEG	N/A	28.5	N/A	28.2	NEG	No	POS
15	MTB	23.55	6874.0	24.0	4214.0	27.0	POS	18.8	N/A	18.7	N/A	POS	No	POS
17	MTB	31.13	48.0	30.5	56.7	31.1	POS	22.8	N/A	23.1	N/A	POS	No	POS
18	MTB	N/A	N/A	N/A	N/A	30.2	NEG	N/A	28.0	N/A	28.6	NEG	Rhinovirus	NEG
19	MTB	N/A	N/A	N/A	N/A	N/A	IND	N/A	28.5	N/A	28.9	NEG	Human coronavirus, Influenza A	NEG
20	MTB	N/A	N/A	N/A	N/A	25.9	NEG	N/A	28.6	N/A	28.2	NEG	Streptococcus pneumoniae, Rhinovirus	NEG
21	MTB	N/A	N/A	N/A	N/A	33.6	NEG	N/A	28.7	N/A	28.5	NEG	Streptococcus pneumoniae, Adenovirus	NEG
22	MTB	N/A	N/A	N/A	N/A	30.9	NEG	N/A	28.9	N/A	29.3	NEG	Human bocavirus	NEG
23	MTB	N/A	N/A	N/A	N/A	34.3	NEG	N/A	28.9	N/A	28.9	NEG	Rhinovirus, Influenza B	NEG
24	MTB	N/A	N/A	N/A	N/A	34.7	NEG	N/A	29.8	N/A	28.5	NEG	Rhinovirus	NEG
25	MTB	33.36	11.2	34.1	5.1	27.1	POS	28.1	30.2	27.7	30.3	POS	Adenovirus	POS
26	MTB	32.5	19.6	32.9	11.2	24.0	POS	23.6	N/A	22.8	N/A	POS	No	POS
27	MTB	40.47	0.1	37.1	0.7	26.3	POS	N/A	29.3	N/A	28.7	NEG	No	POS
29	MTB	31.54	36.8	32.1	19.6	26.6	POS	N/A	29.3	24.6	N/A	Discordant	No	POS
30	MTB	N/A	N/A	N/A	N/A	28.8	NEG	N/A	28.9	N/A	28.4	NEG	Rhinovirus, Streptococcus pneumoniae	NEG
31	MTB	N/A	N/A	N/A	N/A	27.3	NEG	N/A	28.0	N/A	28.6	NEG	Influenza A, Streptococcus pneumoniae	NEG
32	MTB	N/A	N/A	N/A	N/A	23.4	NEG	N/A	28.4	N/A	28.7	NEG	Streptococcus pneumoniae	NEG
33	MTB	N/A	N/A	N/A	N/A	37.0	NEG	N/A	29.1	N/A	28.6	NEG	Streptococcus pneumoniae	NEG
34	MTB	N/A	N/A	N/A	N/A	31.0	NEG	N/A	28.6	N/A	29.3	NEG	Human bocavirus	NEG
35	MTB	N/A	N/A	N/A	N/A	35.2	NEG	N/A	28.6	N/A	28.3	NEG	Rhinovirus, Influenza B	NEG
36	MTB	N/A	N/A	N/A	N/A	29.4	NEG	N/A	29.5	N/A	28.8	NEG	Streptococcus pneumoniae, Rhinovirus, Influenza B	NEG
37	MTB	32.7	17.2	32.9	11.1	28.1	POS	23.5	N/A	23.9	N/A	POS	Streptococcus pneumoniae	POS
38	MTB	18.27	219800.0	18.6	164700.0	28.8	POS	15.2	N/A	15.4	N/A	POS	Rhinovirus	POS
39	MTB	32.88	15.2	35.0	2.7	28.6	POS	24.5	N/A	N/A	28.9	Discordant	Rhinovirus	POS
41	MTB	19.9	75100.0	19.8	72740.0	29.8	POS	16.5	N/A	16.5	N/A	POS	Streptococcus pneumoniae	NEG

42	MTB	N/A	N/A	N/A	N/A	26.2	NEG	N/A	29.0	N/A	28.3	NEG	Influenza A, Streptococcus pneumoniae	POS
43	MTB	N/A	N/A	N/A	N/A	31.5	NEG	N/A	28.1	N/A	28.8	NEG	Rhinovirus, Streptococcus pneumoniae	NEG
44	MTB	N/A	N/A	N/A	N/A	28.8	NEG	N/A	29.0	N/A	29.0	NEG	Streptococcus pneumoniae	NEG
45	MTB	N/A	N/A	N/A	N/A	32.1	NEG	N/A	29.2	N/A	29.4	NEG	Streptococcus pneumoniae, Rhinovirus, Enterovirus D68	NEG
46	MTB	N/A	N/A	N/A	N/A	N/A	IND	N/A	28.4	N/A	28.5	NEG	Human parainfluenza	NEG
47	MTB	N/A	N/A	N/A	N/A	N/A	IND	N/A	28.8	N/A	27.8	NEG	Influenza B	NEG
48	MTB	N/A	N/A	N/A	N/A	N/A	IND	N/A	29.0	N/A	28.3	NEG	Influenza B	NEG
49	MTB	22.8	10718.0	22.5	6840.0	34.7	POS	18.7	N/A	18.4	N/A	POS	No	NEG
50	MTB	33.42	10.7	32.5	14.7	29.2	POS	23.9	N/A	26.8	28.9	POS	No	NEG
51	MTB	25.65	1742.6	25.3	1763.6	30.5	POS	19.8	N/A	19.9	N/A	POS	No	POS
53	MTB	36.88	0.2	N/A	N/A	31.4	INC	N/A	29.0	N/A	28.6	NEG	No	POS
54	MTB	N/A	N/A	N/A	N/A	33.4	NEG	N/A	28.3	N/A	28.4	NEG	Adenovirus, Human coronavirus, Streptococcus pneumoniae	NEG
55	MTB	N/A	N/A	N/A	N/A	34.0	NEG	N/A	28.7	N/A	27.9	NEG	Influenza A, Rhinovirus, Streptococcus pneumoniae	NEG
56	MTB	N/A	N/A	N/A	N/A	23.6	NEG	N/A	29.0	N/A	28.9	NEG	Streptococcus pneumoniae	NEG
57	MTB	N/A	N/A	N/A	N/A	38.8	NEG	N/A	29.8	N/A	28.9	NEG	Streptococcus pneumoniae	NEG
58	MTB	N/A	N/A	N/A	N/A	29.9	NEG	N/A	28.4	N/A	28.0	NEG	Streptococcus pneumoniae	POS
59	MTB	N/A	N/A	N/A	N/A	29.7	NEG	N/A	29.0	N/A	28.1	NEG	Rhinovirus, Influenza B	NEG
60	MTB	N/A	N/A	N/A	N/A	31.9	NEG	N/A	28.6	N/A	28.3	NEG	No	NEG
61	MTB	27.4	619.2	27.3	440.2	30.0	POS	20.1	N/A	20.4	N/A	POS	No	UNK
62	MTB	24.51	3680.0	24.3	3550.0	30.1	POS	18.6	N/A	18.5	N/A	POS	No	POS
63	NP	36.9	1.1	36.2	1.2	24.5	POS	N/A	28.5	N/A	28.3	NEG	No	POS
65	MTB	29.66	125.8	29.8	90.0	25.5	POS	20.8	N/A	21.3	N/A	POS	No	POS
67	MTB	N/A	N/A	N/A	N/A	30.7	NEG	N/A	28.0	N/A	28.0	NEG	Human metapneumov irus	NEG
68	MTB	N/A	N/A	N/A	N/A	25.3	NEG	N/A	28.2	N/A	27.6	NEG	Rhinovirus, Streptococcus pneumoniae	NEG
69	MTB	N/A	N/A	N/A	N/A	34.0	NEG	N/A	29.3	N/A	28.3	NEG	Streptococcus pneumoniae	NEG
70	MTB	N/A	N/A	N/A	N/A	28.2	NEG	N/A	28.9	N/A	27.8	NEG	Human parainfluenza	NEG
71	MTB	N/A	N/A	N/A	N/A	N/A	IND	N/A	29.4	N/A	28.4	NEG	Rhinovirus	NEG
72	MTB	N/A	N/A	N/A	N/A	31.3	NEG	N/A	29.5	N/A	29.6	NEG	No	NEG
73	MTB	25.27	2228.0	25.3	1791.0	26.9	POS	19.4	N/A	19.4	N/A	POS	No	POS
74	MTB	39.5	0.2	N/A	N/A	30.0	INC	N/A	29.6	N/A	29.8	NEG	Rhinovirus	POS
75	MTB	34.92	4.0	34.2	4.6	25.4	POS	25.7	N/A	N/A	29.1	Discorda nt	No	POS
77	MTB	20.43	53220.0	20.7	38920.0	23.3	POS	16.9	N/A	16.8	N/A	POS	No	POS
78	MTB	N/A	N/A	N/A	N/A	30.6	NEG	N/A	28.3	N/A	28.1	NEG	Human coronavius	NEG

79	MTB	N/A	N/A	N/A	N/A	28.8	NEG	N/A	28.7	N/A	28.1	NEG	Rhinovirus, Streptococcus pneumoniae	NEG
80	MTB	N/A	N/A	N/A	N/A	26.0	NEG	N/A	29.3	N/A	28.6	NEG	Streptococcus pneumoniae, Human coronavirus, Human bocavirus, Adenovirus	NEG
81	MTB	N/A	N/A	N/A	N/A	26.9	NEG	N/A	28.7	N/A	28.3	NEG	Human coronavirus	NEG
82	MTB	N/A	N/A	N/A	N/A	29.5	NEG	N/A	28.6	N/A	28.6	NEG	Streptococcus pneumoniae	NEG
83	MTB	N/A	N/A	N/A	N/A	39.1	NEG	N/A	29.5	N/A	29.3	NEG	Rhinovirus, Influenza B	NEG
84	MTB	N/A	N/A	N/A	N/A	30.1	NEG	N/A	29.0	N/A	29.4	NEG	Rhinovirus	NEG
85	MTB	26.97	733.0	27.5	407.0	27.9	POS	20.5	N/A	20.8	N/A	POS	Enterovirus, Rhinovirus	POS
86	MTB	30.78	60.3	31.1	37.6	33.7	POS	21.6	N/A	22.8	N/A	POS	No	POS
87	NP	20.19	62480.0	20.6	42300.0	28.6	POS	16.7	N/A	16.8	N/A	POS	No	POS
88	MTB	25.31	2178.0	26.0	1160.4	27.4	POS	19.9	N/A	20.0	N/A	POS	No	POS
89	MTB	28.04	364.0	28.5	220.2	27.4	POS	21.2	N/A	20.9	N/A	POS	No	POS
90	MTB	N/A	N/A	N/A	N/A	30.1	NEG	N/A	28.5	N/A	29.1	NEG	Human coronavirus, Rhinovirus	NEG
91	MTB	N/A	N/A	N/A	N/A	29.1	NEG	N/A	29.1	N/A	28.6	NEG	Streptococcu pneumoniae, Rhinovirus	NEG
92	MTB	N/A	N/A	N/A	N/A	27.2	NEG	N/A	29.4	N/A	29.7	NEG	No	NEG
93	MTB	N/A	N/A	N/A	N/A	N/A	IND	N/A	29.4	N/A	29.3	NEG	Human bocavirus	NEG
94	MTB	N/A	N/A	N/A	N/A	30.6	NEG	N/A	28.8	N/A	28.2	NEG	Rhinovirus	NEG
95	MTB	N/A	N/A	N/A	N/A	32.5	NEG	N/A	29.2	N/A	29.4	NEG	Influenza B	NEG
96	MTB	N/A	N/A	N/A	N/A	31.0	NEG	N/A	29.1	N/A	30.0	NEG	No	NEG
97	MTB	N/A	N/A	N/A	N/A	40.4	NEG	N/A	29.0	N/A	29.3	NEG	No	NEG
98	MTB	N/A	N/A	N/A	N/A	32.8	NEG	N/A	29.3	N/A	29.6	NEG	Streptococcus pneumoniae	NEG
99	MTB	N/A	N/A	N/A	N/A	N/A	IND	N/A	27.7	N/A	28.7	NEG	No	NEG
100	MTB	N/A	N/A	N/A	N/A	34.2	NEG	N/A	27.5	N/A	29.1	NEG	Rhinovirus	NEG
101	MTB	N/A	N/A	N/A	N/A	36.7	NEG	N/A	28.6	N/A	28.6	NEG	No	NEG
102	MTB	N/A	N/A	N/A	N/A	31.0	NEG	N/A	29.0	N/A	28.9	NEG	Rhinovirus, Influenza B	NEG
103	MTB	N/A	N/A	N/A	N/A	29.3	NEG	N/A	28.8	N/A	29.3	NEG	Streptococcus pneumoniae	NEG
104	MTB	N/A	N/A	N/A	N/A	36.8	NEG	N/A	29.2	N/A	29.7	NEG	Rhinovirus	NEG
105	MTB	N/A	N/A	N/A	N/A	33.4	NEG	N/A	28.3	N/A	28.6	NEG	No	NEG
106	MTB	N/A	N/A	N/A	N/A	43.3	NEG	N/A	29.0	N/A	28.7	NEG	No	NEG
107	MTB	N/A	N/A	N/A	N/A	47.1	NEG	N/A	28.8	N/A	29.1	NEG	No	NEG
108	MTB	N/A	N/A	N/A	N/A	32.1	NEG	N/A	29.1	N/A	28.8	NEG	No	NEG
109	MTB	N/A	N/A	N/A	N/A	28.4	NEG	N/A	29.0	N/A	28.8	NEG	No	NEG

Supp. Table S2

We gratefully acknowledge the following Authors from the Originating laboratories responsible for obtaining the specimens, as well as the Submitting laboratories where the genome data were generated and shared via GISAID, on which this research is based.

All Submitters of data may be contacted directly via www.gisaid.org

Authors are sorted alphabetically.

Accession ID	Originating Laboratory	Submitting Laboratory	Authors
EPI_ISL_2565995	Genome Analysis Center, Yamanashi Central Hospital	Genome Analysis Center, Yamanashi Central Hospital	Yosuke Hirotsu
EPI_ISL_2597900	WSSE w Gdasku	1. Tricity SARS-CoV-2 sequencing consortium: University of Gdansk, Medical University of Gdansk, Vaxican Ltd., Invicta Ltd. 2. National Institute of Public Health - National Institute of Hygiene, Warsaw, Poland	Maciej Kosinski, Celina Cybulska, Krystyna Bienkowska Szewczyk, Maciej Grzybek, Karolina Gackowska, Marcin Lubocki, Katarzyna Groth, Lukasz Rabalski, Katarzyna Zacharczuk, Magdalena Nowakowska, Magorzata Sadkowska-Todys, Tomasz Wokowicz
EPI_ISL_2597930	Nebraska Public Health Laboratory	NPHL COVID-19 Response Team	NPHL COVID-19 Response Team
EPI_ISL_2601940, EPI_ISL_2601941, EPI_ISL_2601942, EPI_ISL_2601945, EPI_ISL_2601947, EPI_ISL_2601948, EPI_ISL_2601949	ZOL	Jessa	Berden et al. on behalf of the Jessa_cmdLab
EPI_ISL_2604225, EPI_ISL_2604226, EPI_ISL_2612461, EPI_ISL_2612466	Montana Public Health Laboratory	Montana Public Health Laboratory	Joy Ritter, Michelle Mozer, Carrie Biskupiak, Deborah Gibson, Michael Dills
EPI_ISL_2617988, EPI_ISL_2617993, EPI_ISL_2617996, EPI_ISL_2617999, EPI_ISL_2618000, EPI_ISL_2618006, EPI_ISL_2618008, EPI_ISL_2618014, EPI_ISL_2618015	UW Virology Lab	UW Virology Lab	Pavitra Roychoudhury, Hong Xie, Lasata Shrestha, Tien V. Nguyen, Shah Mohamed Bakhash, Michelle Lin, Noah R. Baker, Ricardo Perez, Sean Ellis, Nathan Breit, Robert J. Livingston, Meei-Li Huang, Keith R Jerome, Patrick Mathias, Alexander Greninger
EPI_ISL_2627922	Kansas Health and Environmental Lab	Kansas Health and Environmental Lab	Mike Grose, Katherine Wiggins, Jonathan Barnell, Ben Olsen, and Phil Adam
EPI_ISL_2628054	LHUB-ULB	Labo Klinische Biologie, UZA	Marie Le Mercier, Jasmine Coppens, Basil Britto Xavier, Christine Lammens, Veerle Matheeussen, Herman Goossens
EPI_ISL_2628076, EPI_ISL_2628077, EPI_ISL_2628078, EPI_ISL_2628079, EPI_ISL_2628080, EPI_ISL_2628081, EPI_ISL_2628082, EPI_ISL_2628083, EPI_ISL_2628084, EPI_ISL_2628085, EPI_ISL_2628087, EPI_ISL_2628089, EPI_ISL_2628090, EPI_ISL_2628091, EPI_ISL_2628092, EPI_ISL_2628093, EPI_ISL_2628094, EPI_ISL_2628095, EPI_ISL_2628096, EPI_ISL_2628098, EPI_ISL_2628101, EPI_ISL_2628102, EPI_ISL_2628104			
see above	Platform BIS UZA/UAntwerpen	Labo Klinische Biologie, UZA	Marie Le Mercier, Jasmine Coppens, Basil Britto Xavier, Christine Lammens, Veerle Matheeussen, Herman Goossens
EPI_ISL_2635603, EPI_ISL_2635613, EPI_ISL_2635615, EPI_ISL_2635624, EPI_ISL_2635633, EPI_ISL_2635639, EPI_ISL_2635647	nordlab - Partnerschaftspraxis fur Laboratoriumsmedizin	Robert Koch Institute	unknown
EPI_ISL_2635653	amedes MVZ für Labordiagnostik Rhein-Main	Robert Koch Institute	unknown
EPI_ISL_2635663, EPI_ISL_2635672	Limbach - MVZ Humangenetik Ulm	Robert Koch Institute	unknown
EPI_ISL_2635866, EPI_ISL_2635878	Limbach - MVZ Labor Eveld & Kollegen Essen	Robert Koch Institute	unknown
EPI_ISL_2637851	GH de l'Est Francilien	Department of Virology, Henri Mondor University Hospital, Assistance Publique Hôpitaux de Paris, Université Paris-Est Créteil, INSERM U955	Christophe Rodriguez, Slim Fourati, Vanessa Demontant, Guillaume Gricourt, Melissa N'Debi, Alexandre Soulier, Elisabeth Trawinski, Jean-Michel Pawlotsky
EPI_ISL_2637903, EPI_ISL_2637951	CH.INTERCOMMUNAL DE CRETEIL	Department of Virology, Henri Mondor University Hospital, Assistance Publique Hôpitaux de Paris, Université Paris-Est Créteil, INSERM U955	Christophe Rodriguez, Slim Fourati, Vanessa Demontant, Guillaume Gricourt, Melissa N'Debi, Alexandre Soulier, Elisabeth Trawinski, Jean-Michel Pawlotsky
EPI_ISL_2637996	GH de l'Est Francilien	Department of Virology, Henri Mondor University Hospital, Assistance Publique Hôpitaux de Paris, Université Paris-Est Créteil, INSERM U955	Christophe Rodriguez, Slim Fourati, Vanessa Demontant, Guillaume Gricourt, Melissa N'Debi, Alexandre Soulier, Elisabeth Trawinski, Jean-Michel Pawlotsky
EPI_ISL_2638660	Lighthouse Lab in Milton Keynes	Wellcome Sanger Institute for the COVID-19 Genomics UK (COG-UK) Consortium	The Lighthouse Lab in Milton Keynes and Alex Alderton, Roberto Amato, Jeffrey Barrett, Sonia Goncalves, Ewan Harrison, David K. Jackson, Ian Johnston, Dominic Kwiatkowski, Cordelia Langford, John Sillitoe on behalf of the Wellcome Sanger Institute COVID-19 Surveillance Team
EPI_ISL_2638853	Lighthouse Lab in Glasgow	Wellcome Sanger Institute for the COVID-19 Genomics UK (COG-UK) Consortium	Harper VanSteenhouse, Yumi Kasai, David Gray, Carol Clugston, Anna Dominiczak and Alex Alderton, Roberto Amato, Jeffrey Barrett, Sonia Goncalves, Ewan Harrison, David K. Jackson, Ian Johnston, Dominic Kwiatkowski, Cordelia Langford, John Sillitoe on behalf of the Wellcome Sanger Institute COVID-19 Surveillance Team
EPI_ISL_2638871	Lighthouse Lab in Milton Keynes	Wellcome Sanger Institute for the COVID-19 Genomics UK (COG-UK) Consortium	The Lighthouse Lab in Milton Keynes and Alex Alderton, Roberto Amato, Jeffrey Barrett, Sonia Goncalves, Ewan Harrison, David K. Jackson, Ian Johnston, Dominic Kwiatkowski, Cordelia Langford, John Sillitoe on behalf of the Wellcome Sanger Institute COVID-19 Surveillance Team
EPI_ISL_2639029	Lighthouse Laboratory Plymouth	Wellcome Sanger Institute for the COVID-19 Genomics UK (COG-UK) Consortium	Lighthouse Laboratory Plymouth and Alex Alderton, Roberto Amato, Jeffrey Barrett, Sonia Goncalves, Ewan Harrison, David K. Jackson, Ian Johnston, Dominic Kwiatkowski, Cordelia Langford, John Sillitoe on behalf of the Wellcome Sanger Institute COVID-19 Surveillance Team
EPI_ISL_2639092	Lighthouse Lab in Milton Keynes	Wellcome Sanger Institute for the COVID-19 Genomics UK (COG-UK) Consortium	The Lighthouse Lab in Milton Keynes and Alex Alderton, Roberto Amato, Jeffrey Barrett, Sonia Goncalves, Ewan Harrison, David K. Jackson, Ian Johnston, Dominic Kwiatkowski, Cordelia Langford, John Sillitoe on behalf of the Wellcome Sanger Institute COVID-19 Surveillance Team
EPI_ISL_2639330, EPI_ISL_2639334, EPI_ISL_2639348, EPI_ISL_2639367, EPI_ISL_2639584, EPI_ISL_2639650, EPI_ISL_2639709, EPI_ISL_2639865	Lighthouse Lab in Alderley Park	Wellcome Sanger Institute for the COVID-19 Genomics UK (COG-UK) Consortium	Jacquelyn Wynn, Mairead Hyland, The Lighthouse Lab in Alderley Park and Alex Alderton, Roberto Amato, Jeffrey Barrett, Sonia Goncalves, Ewan Harrison, David K. Jackson, Ian Johnston, Dominic Kwiatkowski, Cordelia Langford, John Sillitoe on behalf of the Wellcome Sanger Institute COVID-19 Surveillance Team
EPI_ISL_2641978, EPI_ISL_2641980, EPI_ISL_2641985, EPI_ISL_2641986, EPI_ISL_2641987, EPI_ISL_2641988, EPI_ISL_2641989, EPI_ISL_2642023, EPI_ISL_2642024, EPI_ISL_2642025, EPI_ISL_2642026, EPI_ISL_2642027, EPI_ISL_2642034, EPI_ISL_2642035, EPI_ISL_2642037, EPI_ISL_2642038, EPI_ISL_2642039, EPI_ISL_2642041			
see above	Istituto Zooprofilattico Sperimentale del Mezzogiorno	Telethon Institute of Genetics and Medicine (TIGEM)	Antonio Grimaldi Patrizia Annunziata Francesco Panariello Biancamaria Pierri Claudia Tiberio Teresa Giuliano Valentina Bouche Chiara Colantuono Maria Concetta Cuomo Denise Di Concilio Lucio Di Filippo Anna Manfredi Marcello Salvi Antonio Limone Luigi Atripaldi Pellegrino Cerino Andrea Ballabio Davide Cacchiarelli
EPI_ISL_2650882, EPI_ISL_2650884, EPI_ISL_2650885, EPI_ISL_2650886	ASL TO4	Fondazione del Piemonte per l'Oncologia IRCCS	Antonino Sottile, Silvia Brossa, Paola Marino, Giorgio Giardina
EPI_ISL_2650888	ASL CN1	Fondazione del Piemonte per l'Oncologia IRCCS	Antonino Sottile, Silvia Brossa, Paola Marino, Giorgio Giardina
EPI_ISL_2650890	ASL TO4	Fondazione del Piemonte per l'Oncologia IRCCS	Antonino Sottile, Silvia Brossa, Paola Marino, Giorgio Giardina

EPI_ISL_2651107	Washington State Department of Health Public Health Laboratories	Washington State Department of Health Public Health Laboratories	Drew MacKellar, Philip Dykema, Denny Russell, Joenice Gonzalez, Hannah Gray, Geoff Melly, Vanessa De Los Santos, Darren Lucas, JohnAric Peterson, Avi Singh, Rebecca Cao
EPI_ISL_2651786, EPI_ISL_2651896	Lighthouse Lab in Glasgow	Wellcome Sanger Institute for the COVID-19 Genomics UK (COG-UK) Consortium	Harper VanSteenhouse, Yumi Kasai, David Gray, Carol Clugston, Anna Dominiczak and Alex Alderton, Roberto Amato, Jeffrey Barrett, Sonia Goncalves, Ewan Harrison, David K. Jackson, Ian Johnston, Dominic Kwiatkowski, Cordelia Langford, John Sillitoe on behalf of the Wellcome Sanger Institute COVID-19 Surveillance Team
EPI_ISL_2661534	UHTL, University Hospitals	UHTL, University Hospitals	Sadri,N., Alouani,D., Song,X.
EPI_ISL_2663213, EPI_ISL_2663217	HealthPartners Central Lab	Minnesota Department of Health, Public Health Laboratory	Alexandra Lorentz, Jacob Garfin, Matt Plumb, and Xiong Wang
EPI_ISL_2664038	Lighthouse Lab in Alderley Park	Wellcome Sanger Institute for the COVID-19 Genomics UK (COG-UK) Consortium	Jacquelyn Wynn, Mairead Hyland, The Lighthouse Lab in Alderley Park and Alex Alderton, Roberto Amato, Jeffrey Barrett, Sonia Goncalves, Ewan Harrison, David K. Jackson, Ian Johnston, Dominic Kwiatkowski, Cordelia Langford, John Sillitoe on behalf of the Wellcome Sanger Institute COVID-19 Surveillance Team
EPI_ISL_2664937, EPI_ISL_2665215	Lighthouse Lab in Milton Keynes	Wellcome Sanger Institute for the COVID-19 Genomics UK (COG-UK) Consortium	The Lighthouse Lab in Milton Keynes and Alex Alderton, Roberto Amato, Jeffrey Barrett, Sonia Goncalves, Ewan Harrison, David K. Jackson, Ian Johnston, Dominic Kwiatkowski, Cordelia Langford, John Sillitoe on behalf of the Wellcome Sanger Institute COVID-19 Surveillance Team
EPI_ISL_2665417, EPI_ISL_2665432, EPI_ISL_2665492, EPI_ISL_2665506, EPI_ISL_2665508, EPI_ISL_2665510, EPI_ISL_2665511, EPI_ISL_2665521, EPI_ISL_2665522, EPI_ISL_2665523, EPI_ISL_2665532, EPI_ISL_2665535, EPI_ISL_2665547, EPI_ISL_2665573, EPI_ISL_2665575, EPI_ISL_2665583, EPI_ISL_2665638, EPI_ISL_2665643, EPI_ISL_2665644, EPI_ISL_2665660, EPI_ISL_2665669, EPI_ISL_2665673, EPI_ISL_2665695, EPI_ISL_2665696, EPI_ISL_2665697, EPI_ISL_2665741, EPI_ISL_2665746, EPI_ISL_2665748, EPI_ISL_2665757, EPI_ISL_2665768, EPI_ISL_2665770, EPI_ISL_2665774, EPI_ISL_2665776, EPI_ISL_2665784, EPI_ISL_2665804, EPI_ISL_2665819, EPI_ISL_2665831, EPI_ISL_2665841, EPI_ISL_2665852, EPI_ISL_2665874, EPI_ISL_2665896, EPI_ISL_2665898, EPI_ISL_2665900, EPI_ISL_2665909, EPI_ISL_2665912, EPI_ISL_2665941, EPI_ISL_2665947, EPI_ISL_2665954, EPI_ISL_2665955, EPI_ISL_2665968, EPI_ISL_2665980, EPI_ISL_2665985, EPI_ISL_2666007, EPI_ISL_2666013, EPI_ISL_2666045, EPI_ISL_2666046, EPI_ISL_2666047, EPI_ISL_2666059, EPI_ISL_2666067, EPI_ISL_2666068, EPI_ISL_2666084, EPI_ISL_2666090, EPI_ISL_2666113, EPI_ISL_2666127, EPI_ISL_2666134, EPI_ISL_2666135, EPI_ISL_2666140, EPI_ISL_2666158, EPI_ISL_2666171, EPI_ISL_2666191, EPI_ISL_2666199, EPI_ISL_2666204, EPI_ISL_2666217, EPI_ISL_2666227, EPI_ISL_2666240, EPI_ISL_2666248, EPI_ISL_2666250, EPI_ISL_2666252, EPI_ISL_2666271, EPI_ISL_2666276, EPI_ISL_2666289, EPI_ISL_2666296, EPI_ISL_2666306, EPI_ISL_2666327, EPI_ISL_2666351, EPI_ISL_2666353, EPI_ISL_2666361, EPI_ISL_2666363, EPI_ISL_2666364, EPI_ISL_2666370, EPI_ISL_2666377, EPI_ISL_2666379, EPI_ISL_2666389, EPI_ISL_2666423, EPI_ISL_2666429, EPI_ISL_2666514, EPI_ISL_2666526, EPI_ISL_2666527, EPI_ISL_2666541, EPI_ISL_2666543, EPI_ISL_2666546, EPI_ISL_2666549, EPI_ISL_2666565, EPI_ISL_2666566, EPI_ISL_2666583			
see above	Department of Bacteria, Parasites and Fungi, Statens Serum Institut, Copenhagen, Denmark	Statens Serum Institut Bioinformatics and Microbial Genomics	Danish Covid-19 Genome Consortium
EPI_ISL_2666602	Molekylær Medicinsk Afdeling, Aarhus University Hospital, Aarhus, Denmark	Statens Serum Institut Bioinformatics and Microbial Genomics	Danish Covid-19 Genome Consortium
EPI_ISL_2666607, EPI_ISL_2666621, EPI_ISL_2666622, EPI_ISL_2666671, EPI_ISL_2666703, EPI_ISL_2666757, EPI_ISL_2666766, EPI_ISL_2666776, EPI_ISL_2666793, EPI_ISL_2666801, EPI_ISL_2666806, EPI_ISL_2666851, EPI_ISL_2666857, EPI_ISL_2666862, EPI_ISL_2666878, EPI_ISL_2666904, EPI_ISL_2666923, EPI_ISL_2666929, EPI_ISL_2666932, EPI_ISL_2666933			
see above	Department of Bacteria, Parasites and Fungi, Statens Serum Institut, Copenhagen, Denmark	Statens Serum Institut Bioinformatics and Microbial Genomics	Danish Covid-19 Genome Consortium
EPI_ISL_2666939	Molekylær Medicinsk Afdeling, Aarhus University Hospital, Aarhus, Denmark	Statens Serum Institut Bioinformatics and Microbial Genomics	Danish Covid-19 Genome Consortium
EPI_ISL_2666960, EPI_ISL_2666990, EPI_ISL_2666998, EPI_ISL_2666999, EPI_ISL_2667002	Department of Bacteria, Parasites and Fungi, Statens Serum Institut, Copenhagen, Denmark	Statens Serum Institut Bioinformatics and Microbial Genomics	Danish Covid-19 Genome Consortium
EPI_ISL_2667025	Molekylær Medicinsk Afdeling, Aarhus University Hospital, Aarhus, Denmark	Statens Serum Institut Bioinformatics and Microbial Genomics	Danish Covid-19 Genome Consortium
EPI_ISL_2667053, EPI_ISL_2667056, EPI_ISL_2667061, EPI_ISL_2667064, EPI_ISL_2667072, EPI_ISL_2667082, EPI_ISL_2667114, EPI_ISL_2667134, EPI_ISL_2667140, EPI_ISL_2667159, EPI_ISL_2667161, EPI_ISL_2667175, EPI_ISL_2667177, EPI_ISL_2667186, EPI_ISL_2667199, EPI_ISL_2667235, EPI_ISL_2667276, EPI_ISL_2667304, EPI_ISL_2667324, EPI_ISL_2667341, EPI_ISL_2667352, EPI_ISL_2667365, EPI_ISL_2667371, EPI_ISL_2667380, EPI_ISL_2667403, EPI_ISL_2667406, EPI_ISL_2667408, EPI_ISL_2667428, EPI_ISL_2667432, EPI_ISL_2667435, EPI_ISL_2667438, EPI_ISL_2667440, EPI_ISL_2667446, EPI_ISL_2667472, EPI_ISL_2667474, EPI_ISL_2667476, EPI_ISL_2667477, EPI_ISL_2667480, EPI_ISL_2667510, EPI_ISL_2667523, EPI_ISL_2667530, EPI_ISL_2667532, EPI_ISL_2667536, EPI_ISL_2667576, EPI_ISL_2667586, EPI_ISL_2667620, EPI_ISL_2667621, EPI_ISL_2667651, EPI_ISL_2667657, EPI_ISL_2667692, EPI_ISL_2667694, EPI_ISL_2667695, EPI_ISL_2667723, EPI_ISL_2667729, EPI_ISL_2667772, EPI_ISL_2667773, EPI_ISL_2667775, EPI_ISL_2667782, EPI_ISL_2667802, EPI_ISL_2667806, EPI_ISL_2667820, EPI_ISL_2667821, EPI_ISL_2667826, EPI_ISL_2667828, EPI_ISL_2667837, EPI_ISL_2667841, EPI_ISL_2667854, EPI_ISL_2667857, EPI_ISL_2667860, EPI_ISL_2667863, EPI_ISL_2667889, EPI_ISL_2667899, EPI_ISL_2667911, EPI_ISL_2667923, EPI_ISL_2667931, EPI_ISL_2667938, EPI_ISL_2667944, EPI_ISL_2667953, EPI_ISL_2667957, EPI_ISL_2667958, EPI_ISL_2668037, EPI_ISL_2668040, EPI_ISL_2668046, EPI_ISL_2668052, EPI_ISL_2668068, EPI_ISL_2668083, EPI_ISL_2668084, EPI_ISL_2668085, EPI_ISL_2668092, EPI_ISL_2668093, EPI_ISL_2668109, EPI_ISL_2668119, EPI_ISL_2668120, EPI_ISL_2668126, EPI_ISL_2668144, EPI_ISL_2668149, EPI_ISL_2668179, EPI_ISL_2668185, EPI_ISL_2668196, EPI_ISL_2668199, EPI_ISL_2668215, EPI_ISL_2668216, EPI_ISL_2668236, EPI_ISL_2668242, EPI_ISL_2668249, EPI_ISL_2668258, EPI_ISL_2668265, EPI_ISL_2668287, EPI_ISL_2668299, EPI_ISL_2668301, EPI_ISL_2668308, EPI_ISL_2668329, EPI_ISL_2668336, EPI_ISL_2668338, EPI_ISL_2668341, EPI_ISL_2668358, EPI_ISL_2668379, EPI_ISL_2668380, EPI_ISL_2668393, EPI_ISL_2668394, EPI_ISL_2668411, EPI_ISL_2668426, EPI_ISL_2668437, EPI_ISL_2668442, EPI_ISL_2668443			
see above	Department of Bacteria, Parasites and Fungi, Statens Serum Institut, Copenhagen, Denmark	Statens Serum Institut Bioinformatics and Microbial Genomics	Danish Covid-19 Genome Consortium
EPI_ISL_2668447	Molekylær Medicinsk Afdeling, Aarhus University Hospital, Aarhus, Denmark	Statens Serum Institut Bioinformatics and Microbial Genomics	Danish Covid-19 Genome Consortium
EPI_ISL_2668450, EPI_ISL_2668463, EPI_ISL_2668478, EPI_ISL_2668498, EPI_ISL_2668508, EPI_ISL_2668537, EPI_ISL_2668544, EPI_ISL_2668545, EPI_ISL_2668549, EPI_ISL_2668565, EPI_ISL_2668566, EPI_ISL_2668578, EPI_ISL_2668582, EPI_ISL_2668591, EPI_ISL_2668597, EPI_ISL_2668600, EPI_ISL_2668618, EPI_ISL_2668627, EPI_ISL_2668641, EPI_ISL_2668644, EPI_ISL_2668665, EPI_ISL_2668680, EPI_ISL_2668681, EPI_ISL_2668704, EPI_ISL_2668716, EPI_ISL_2668726, EPI_ISL_2668742			
see above	Department of Bacteria, Parasites and Fungi, Statens Serum Institut, Copenhagen, Denmark	Statens Serum Institut Bioinformatics and Microbial Genomics	Danish Covid-19 Genome Consortium
EPI_ISL_2668775	Molekylær Medicinsk Afdeling, Aarhus University Hospital, Aarhus, Denmark	Statens Serum Institut Bioinformatics and Microbial Genomics	Danish Covid-19 Genome Consortium
EPI_ISL_2668797, EPI_ISL_2668803, EPI_ISL_2668811, EPI_ISL_2668815, EPI_ISL_2668818, EPI_ISL_2668852, EPI_ISL_2668890, EPI_ISL_2668906, EPI_ISL_2668924, EPI_ISL_2668929, EPI_ISL_2668937, EPI_ISL_2668961, EPI_ISL_2668988, EPI_ISL_2669001, EPI_ISL_2669021, EPI_ISL_2669039, EPI_ISL_2669040, EPI_ISL_2669046, EPI_ISL_2669056, EPI_ISL_2669059, EPI_ISL_2669092, EPI_ISL_2669100, EPI_ISL_2669106, EPI_ISL_2669124, EPI_ISL_2669128, EPI_ISL_2669132, EPI_ISL_2669134, EPI_ISL_2669157, EPI_ISL_2669158, EPI_ISL_2669166, EPI_ISL_2669193, EPI_ISL_2669200, EPI_ISL_2669233, EPI_ISL_2669244, EPI_ISL_2669250, EPI_ISL_2669257, EPI_ISL_2669259, EPI_ISL_2669274, EPI_ISL_2669275, EPI_ISL_2669276, EPI_ISL_2669300, EPI_ISL_2669325, EPI_ISL_2669328, EPI_ISL_2669353, EPI_ISL_2669356, EPI_ISL_2669379, EPI_ISL_2669380, EPI_ISL_2669390, EPI_ISL_2669395, EPI_ISL_2669401, EPI_ISL_2669422, EPI_ISL_2669432, EPI_ISL_2669444, EPI_ISL_2669472, EPI_ISL_2669473, EPI_ISL_2669481, EPI_ISL_2669483, EPI_ISL_2669511, EPI_ISL_2669516, EPI_ISL_2669529, EPI_ISL_2669533, EPI_ISL_2669547, EPI_ISL_2669578, EPI_ISL_2669611, EPI_ISL_2669624, EPI_ISL_2669651, EPI_ISL_2669667, EPI_ISL_2669681, EPI_ISL_2669683, EPI_ISL_2669704, EPI_ISL_2669705, EPI_ISL_2669711, EPI_ISL_2669715, EPI_ISL_2669722, EPI_ISL_2669748			
see above	Department of Bacteria, Parasites and Fungi, Statens Serum Institut, Copenhagen, Denmark	Statens Serum Institut Bioinformatics and Microbial Genomics	Danish Covid-19 Genome Consortium
EPI_ISL_2669755	Molekylær Medicinsk Afdeling, Aarhus University Hospital, Aarhus, Denmark	Statens Serum Institut Bioinformatics and Microbial Genomics	Danish Covid-19 Genome Consortium
EPI_ISL_2669757, EPI_ISL_2669760, EPI_ISL_2669792, EPI_ISL_2669806, EPI_ISL_2669817, EPI_ISL_2669818, EPI_ISL_2669839, EPI_ISL_2669861, EPI_ISL_2669867, EPI_ISL_2669884, EPI_ISL_2669896, EPI_ISL_2669897, EPI_ISL_2669945, EPI_ISL_2669948, EPI_ISL_2669953, EPI_ISL_2669954, EPI_ISL_2669965, EPI_ISL_2669971, EPI_ISL_2669978, EPI_ISL_2669986, EPI_ISL_2670003, EPI_ISL_2670004, EPI_ISL_2670020, EPI_ISL_2670027, EPI_ISL_2670030, EPI_ISL_2670031, EPI_ISL_2670033, EPI_ISL_2670066, EPI_ISL_2670081			
see above	Department of Bacteria, Parasites and Fungi, Statens Serum Institut, Copenhagen, Denmark	Statens Serum Institut Bioinformatics and Microbial Genomics	Danish Covid-19 Genome Consortium
EPI_ISL_2670089	Molekylær Medicinsk Afdeling, Aarhus University Hospital, Aarhus, Denmark	Statens Serum Institut Bioinformatics and Microbial Genomics	Danish Covid-19 Genome Consortium
EPI_ISL_2670120, EPI_ISL_2670127, EPI_ISL_2670137, EPI_ISL_2670147, EPI_ISL_2670159, EPI_ISL_2670167, EPI_ISL_2670173, EPI_ISL_2670180, EPI_ISL_2670187, EPI_ISL_2670196, EPI_ISL_2670199, EPI_ISL_2670211, EPI_ISL_2670243, EPI_ISL_2670244, EPI_ISL_2670250, EPI_ISL_2670275, EPI_ISL_2670282, EPI_ISL_2670302, EPI_ISL_2670303, EPI_ISL_2670304, EPI_ISL_2670338, EPI_ISL_2670340, EPI_ISL_2670348, EPI_ISL_2670365, EPI_ISL_2670390, EPI_ISL_2670394, EPI_ISL_2670413, EPI_ISL_2670424, EPI_ISL_2670446, EPI_ISL_2670479, EPI_ISL_2670487, EPI_ISL_2670489, EPI_ISL_2670490, EPI_ISL_2670505, EPI_ISL_2670536, EPI_ISL_2670543, EPI_ISL_2670553, EPI_ISL_2670564, EPI_ISL_2670569, EPI_ISL_2670572, EPI_ISL_2670592, EPI_ISL_2670604, EPI_ISL_2670628, EPI_ISL_2670631, EPI_ISL_2670636, EPI_ISL_2670662, EPI_ISL_2670679, EPI_ISL_2670693, EPI_ISL_2670700, EPI_ISL_2670722, EPI_ISL_2670729, EPI_ISL_2670733, EPI_ISL_2670746, EPI_ISL_2670757, EPI_ISL_2670761, EPI_ISL_2670774, EPI_ISL_2670776, EPI_ISL_2670789, EPI_ISL_2670811, EPI_ISL_2670844, EPI_ISL_2670856, EPI_ISL_2670867, EPI_ISL_2670868, EPI_ISL_2670871, EPI_ISL_2670882, EPI_ISL_2670884, EPI_ISL_2670885, EPI_ISL_2670890, EPI_ISL_2670896, EPI_ISL_2670900, EPI_ISL_2670922, EPI_ISL_2670928, EPI_ISL_2670937, EPI_ISL_2670941, EPI_ISL_2670961, EPI_ISL_2670962, EPI_ISL_2670990, EPI_ISL_2671006, EPI_ISL_2671008, EPI_ISL_2671031, EPI_ISL_2671036, EPI_ISL_2671044, EPI_ISL_2671066, EPI_ISL_2671080, EPI_ISL_2671095, EPI_ISL_2671105, EPI_ISL_2671121, EPI_ISL_2671122, EPI_ISL_2671129, EPI_ISL_2671149, EPI_ISL_2671161, EPI_ISL_2671169, EPI_ISL_2671183, EPI_ISL_2671201, EPI_ISL_2671217, EPI_ISL_2671246, EPI_ISL_2671259, EPI_ISL_2671264, EPI_ISL_2671275, EPI_ISL_2671285, EPI_ISL_2671288, EPI_ISL_2671303, EPI_ISL_2671324, EPI_ISL_2671332, EPI_ISL_2671334, EPI_ISL_2671347			

see above	Department of Bacteria, Parasites and Fungi, Statens Serum Institut, Copenhagen, Denmark	Statens Serum Institut Bioinformatics and Microbial Genomics	Danish Covid-19 Genome Consortium
EPI_ISL_2671365	Microbiology Department, Laboratori Clínic Metropolitana Nord. Hospital Universitari Germans Trias i Pujol	Can Ruti SARS-CoV-2 Sequencing Hub (HUGTiP/IrsiCaixa/IGTP)	Marc Noguera-Julian, Pilar Armengol, Ignacio Blanco, Antoni E Bordoy, Francesc Catala-Moll, Pere-Joan Cardona, Maria Casadellà, Cristina Casañ, Gemma Clara, Bonaventura Clotet, Cristina Esteban, Montserrat Giménez, Mercedes Guerrero, Anna Not, Roger Paredes, Mariona Parera, Verónica Saludes, Alba Sánchez, and Elisa Martró on behalf of the Can Ruti SARS-CoV-2 Sequencing Hub
EPI_ISL_2671460, EPI_ISL_2671463, EPI_ISL_2671464, EPI_ISL_2671466, EPI_ISL_2671467, EPI_ISL_2671468, EPI_ISL_2671470	Center for Laboratory Medicine	Center for Laboratory Medicine	Yannick Gerth
EPI_ISL_2672103	IN State Department of Health Laboratory Services	IN State Department of Health Laboratory Services	Cassandra Campion, Jamie Yeadon, Brian Pope, Lixia Liu, Kyle Brownlee, Melissa Hindenlang, Mark Glazier
EPI_ISL_2673675	SIESP CHIETI - DRIVE IN CHIETI	Istituto Zooprofilattico Sperimentale dell'Abruzzo e Molise "G. Caporale"	Lorusso A, Marcacci M, Di Domenico M, Ancora M, Curini V, Di Lollo Valeria, Mangone I, Rinaldi A, Delli Compagni E, Scialabba S, Caporale M, Di Pasquale A, Cammà C, Puglia I, Calistri P, Savini G
EPI_ISL_2673679, EPI_ISL_2673680	PRESIDIO OSPEDALIERO TAGLIACOZZO	Istituto Zooprofilattico Sperimentale dell'Abruzzo e Molise "G. Caporale"	Lorusso A, Marcacci M, Di Domenico M, Ancora M, Curini V, Di Lollo Valeria, Mangone I, Rinaldi A, Delli Compagni E, Scialabba S, Caporale M, Di Pasquale A, Cammà C, Puglia I, Calistri P, Savini G
EPI_ISL_2673681, EPI_ISL_2673682	SIESP CHIETI - DRIVE IN LANCIANO	Istituto Zooprofilattico Sperimentale dell'Abruzzo e Molise "G. Caporale"	Lorusso A, Marcacci M, Di Domenico M, Ancora M, Curini V, Di Lollo Valeria, Mangone I, Rinaldi A, Delli Compagni E, Scialabba S, Caporale M, Di Pasquale A, Cammà C, Puglia I, Calistri P, Savini G
EPI_ISL_2673750	Akershus University Hospital, Department for Microbiology and Infectious Disease Control	Norwegian Institute of Public Health, Department of Virology	Kathrine Stene-Johansen, Kamilla Heddeland Instefjord, Hilde Elshaug, Garcia Llorente Ignacio, Jon Bråte, Engebretsen Serina Beate,Pedersen Benedikte Nevjen, Line Victoria Moen, Debech Nadia, Atiya R Ali,Marie Paulsen Madsen, Rasmus Riis Kopperud, Hilde Vollan, Karoline Bragstad, Olav Hungnes
EPI_ISL_2675085, EPI_ISL_2675086, EPI_ISL_2675087, EPI_ISL_2675088, EPI_ISL_2675089, EPI_ISL_2675090, EPI_ISL_2675091, EPI_ISL_2675092	Vestfold Hospital, Toensberg Department of Microbiology	Norwegian Institute of Public Health, Department of Virology	Kathrine Stene-Johansen, Kamilla Heddeland Instefjord, Hilde Elshaug, Garcia Llorente Ignacio, Jon Bråte, Engebretsen Serina Beate,Pedersen Benedikte Nevjen, Line Victoria Moen, Debech Nadia, Atiya R Ali,Marie Paulsen Madsen, Rasmus Riis Kopperud, Hilde Vollan, Karoline Bragstad, Olav Hungnes
EPI_ISL_2675268, EPI_ISL_2675271, EPI_ISL_2675279, EPI_ISL_2675285, EPI_ISL_2675299	Hospital General Universitario Gregorio Marañón	Hospital General Universitario Gregorio Marañón	Sergio Buenestado Serrano, Pedro Sola Campoy, Laura Pérez-Lago, Cristina Rodriguez-Grande, Marta Herranz Martin, Victor Manuel de la Cueva, Julia Suárez, Pilar Catalán, Patricia Muñoz, Darío García de Viedma
EPI_ISL_2675399, EPI_ISL_2675417, EPI_ISL_2675436, EPI_ISL_2675455, EPI_ISL_2675461, EPI_ISL_2675474, EPI_ISL_2675478, EPI_ISL_2675484, EPI_ISL_2675497, EPI_ISL_2675506, EPI_ISL_2675555, EPI_ISL_2675569, EPI_ISL_2675571, EPI_ISL_2675576, EPI_ISL_2675585, EPI_ISL_2675586, EPI_ISL_2675634, EPI_ISL_2675637, EPI_ISL_2675647, EPI_ISL_2675653, EPI_ISL_2675664, EPI_ISL_2675666, EPI_ISL_2675672, EPI_ISL_2675677, EPI_ISL_2675684, EPI_ISL_2675695, EPI_ISL_2675703, EPI_ISL_2675724, EPI_ISL_2675729, EPI_ISL_2675736, EPI_ISL_2675755, EPI_ISL_2675789, EPI_ISL_2675793, EPI_ISL_2675808, EPI_ISL_2675809, EPI_ISL_2675821, EPI_ISL_2675823, EPI_ISL_2675837, EPI_ISL_2675838, EPI_ISL_2675841, EPI_ISL_2675856, EPI_ISL_2675863, EPI_ISL_2675865			
see above	Department of Bacteria, Parasites and Fungi, Statens Serum Institut, Copenhagen, Denmark	Statens Serum Institut Bioinformatics and Microbial Genomics	Danish Covid-19 Genome Consortium
EPI_ISL_2676577, EPI_ISL_2676627	CNR Virus des Infections Respiratoires - France SUD	CNR Virus des Infections Respiratoires - France SUD	Antonin Bal, Gregory Destras, Gwendolyne Burfin, Hadrien Regue, Quentin Semanas, Martine Valette, Bruno Lina, Laurence Josset
EPI_ISL_2676702, EPI_ISL_2676704	LABORATOIRE BOUVIER	CNR Virus des Infections Respiratoires - France SUD	Antonin Bal, Gregory Destras, Gwendolyne Burfin, Hadrien Regue, Quentin Semanas, Martine Valette, Bruno Lina, Laurence Josset
EPI_ISL_2676753, EPI_ISL_2676758, EPI_ISL_2676759, EPI_ISL_2676760, EPI_ISL_2676762, EPI_ISL_2676763, EPI_ISL_2676764	MIRIALIS CLUSES BECHET	CNR Virus des Infections Respiratoires - France SUD	Antonin Bal, Gregory Destras, Gwendolyne Burfin, Hadrien Regue, Quentin Semanas, Martine Valette, Bruno Lina, Laurence Josset
EPI_ISL_2676793, EPI_ISL_2676797	CNR Virus des Infections Respiratoires - France SUD	CNR Virus des Infections Respiratoires - France SUD	Antonin Bal, Gregory Destras, Gwendolyne Burfin, Hadrien Regue, Quentin Semanas, Martine Valette, Bruno Lina, Laurence Josset
EPI_ISL_2678439, EPI_ISL_2678445	Dianovis GmbH Greiz	Robert Koch Institute	unknown
EPI_ISL_2678446	Städtisches Klinikum Dresden Institut für Klinische Chemie und Laboratoriumsmedizin	Robert Koch Institute	unknown
EPI_ISL_2678447	LABOR CHEMNITZ - Zentrum für Diagnostik am Klinikum Chemnitz	Robert Koch Institute	unknown
EPI_ISL_2678448	Landesuntersuchungsanstalt für das Gesundheits- und Veterinärwesen (LUA) Sachsen	Robert Koch Institute	unknown
EPI_ISL_2678449	LABOR CHEMNITZ - Zentrum für Diagnostik am Klinikum Chemnitz	Robert Koch Institute	unknown
EPI_ISL_2678451	Uniklinikum Carl Gustav Carus an der TU Dresden; Institut für Virologie	Robert Koch Institute	unknown
EPI_ISL_2678454	Medizinisches Labor Ostsachsen MVZ GbR Nebenbetriebsstätte Dresden	Robert Koch Institute	unknown
EPI_ISL_2678455	Städtisches Klinikum Dresden Institut für Klinische Chemie und Laboratoriumsmedizin	Robert Koch Institute	unknown
EPI_ISL_2678457	Medizinisches Labor Ostsachsen MVZ GbR Nebenbetriebsstätte Dresden	Robert Koch Institute	unknown
EPI_ISL_2678458	LABOR CHEMNITZ - Zentrum für Diagnostik am Klinikum Chemnitz	Robert Koch Institute	unknown
EPI_ISL_2678459, EPI_ISL_2678461	Uniklinikum Carl Gustav Carus an der TU Dresden; Institut für Virologie	Robert Koch Institute	unknown
EPI_ISL_2678462, EPI_ISL_2678463	Medizinisches Labor Ostsachsen MVZ GbR Nebenbetriebsstätte Dresden	Robert Koch Institute	unknown
EPI_ISL_2678467	Städtisches Klinikum Dresden Institut für Klinische Chemie und Laboratoriumsmedizin	Robert Koch Institute	unknown
EPI_ISL_2678468	Landesuntersuchungsanstalt für das Gesundheits- und Veterinärwesen (LUA) Sachsen	Robert Koch Institute	unknown
EPI_ISL_2678470	Uniklinikum Carl Gustav Carus an der TU Dresden; Institut für Virologie	Robert Koch Institute	unknown
EPI_ISL_2678471	Medizinisches Labor Ostsachsen MVZ GbR Nebenbetriebsstätte Dresden	Robert Koch Institute	unknown
EPI_ISL_2678646	MVZ Labor Dr. Quade & Kollegen GmbH	Robert Koch Institute	unknown
EPI_ISL_2678741, EPI_ISL_2678742	Klinikum Ernst von Bergmann gemeinnützige GmbH -	Robert Koch Institute	unknown

	stationärer Bereich		
EPI_ISL_2681444, EPI_ISL_2681449	Althaia. Xarxa Assistencial Universitària de Manresa	IrsiCaixa	Marc Noguera-Julian, Mariona Parera, Maria Casadellà, Pilar Armengol, Francesc Catala-Moll, Roger Paredes, Bonaventura Clotet Gloria Trujillo, Rafel Perez Vidal, Jaume Trapé, Carolina Gonzalez-Fernandez, Miquel Micó, Antonia Flor, Roger Paredes, Eulalia Grau, Bonaventura Clotet
EPI_ISL_2681468, EPI_ISL_2681469	University Hospitals of Geneva, Laboratory of Virology	HUG, Laboratory of Virology and the Health2030 Genome Center	Samuel Cordey, Ana Rita Goncalves, Laurent Kaiser, Lorenzo Cerutti, Henri Pegeot, Melyssa Elies, Deborah Penet, Keith Harshman, Ioannis Xenarios, Emmanouil Dermitzakis
EPI_ISL_2681551, EPI_ISL_2681559, EPI_ISL_2681561, EPI_ISL_2681571, EPI_ISL_2681603, EPI_ISL_2681605, EPI_ISL_2681614	Microbiology Department, Laboratori Clínic Metropolitana Nord. Hospital Universitari Germans Trias i Pujol	Can Ruti SARS-CoV-2 Sequencing Hub (HUGTiP/IrsiCaixa/IGTP)	Marc Noguera-Julian, Pilar Armengol, Ignacio Blanco, Antoni E Bordoy, Francesc Catala-Moll, Pere-Joan Cardona, Maria Casadellà, Cristina Casañ, Gemma Clara, Bonaventura Clotet, Cristina Esteban, Montserrat Giménez, Mercedes Guerrero, Anna Not, Roger Paredes, Mariona Parera, Verónica Saludes, Alba Sánchez, and Elisa Martró on behalf of the Can Ruti SARS-CoV-2 Sequencing Hub
EPI_ISL_2682680, EPI_ISL_2682739	Lighthouse Lab in Alderley Park	Wellcome Sanger Institute for the COVID-19 Genomics UK (COG-UK) Consortium	Jacquelyn Wynn, Mairead Hyland, The Lighthouse Lab in Alderley Park and Alex Alderton, Roberto Amato, Jeffrey Barrett, Sonia Goncalves, Ewan Harrison, David K. Jackson, Ian Johnston, Dominic Kwiatkowski, Cordelia Langford, John Sillitoe on behalf of the Wellcome Sanger Institute COVID-19 Surveillance Team
EPI_ISL_2684242, EPI_ISL_2684243, EPI_ISL_2684253, EPI_ISL_2684259, EPI_ISL_2684268, EPI_ISL_2684279, EPI_ISL_2684334, EPI_ISL_2684353, EPI_ISL_2684357, EPI_ISL_2684359, EPI_ISL_2684364, EPI_ISL_2684395, EPI_ISL_2684401, EPI_ISL_2684432, EPI_ISL_2684436, EPI_ISL_2684439, EPI_ISL_2684465, EPI_ISL_2684500, EPI_ISL_2684501, EPI_ISL_2684527, EPI_ISL_2684536, EPI_ISL_2684557, EPI_ISL_2684575, EPI_ISL_2684621, EPI_ISL_2684628, EPI_ISL_2684642, EPI_ISL_2684676, EPI_ISL_2684678, EPI_ISL_2684681, EPI_ISL_2684683, EPI_ISL_2684691, EPI_ISL_2684694, EPI_ISL_2684726, EPI_ISL_2684738, EPI_ISL_2684759, EPI_ISL_2684764, EPI_ISL_2684766, EPI_ISL_2684767, EPI_ISL_2684769, EPI_ISL_2684778, EPI_ISL_2684785, EPI_ISL_2684790, EPI_ISL_2684799, EPI_ISL_2684810, EPI_ISL_2684850, EPI_ISL_2684851, EPI_ISL_2684869, EPI_ISL_2684876, EPI_ISL_2684904, EPI_ISL_2684944, EPI_ISL_2685027, EPI_ISL_2685039, EPI_ISL_2685044, EPI_ISL_2685067, EPI_ISL_2685073, EPI_ISL_2685076, EPI_ISL_2685091, EPI_ISL_2685095, EPI_ISL_2685112, EPI_ISL_2685192, EPI_ISL_2685225, EPI_ISL_2685233, EPI_ISL_2685241, EPI_ISL_2685242, EPI_ISL_2685323, EPI_ISL_2685328, EPI_ISL_2685335, EPI_ISL_2685349, EPI_ISL_2685353, EPI_ISL_2685470, EPI_ISL_2685472, EPI_ISL_2685480, EPI_ISL_2685498, EPI_ISL_2685505, EPI_ISL_2685508, EPI_ISL_2685559, EPI_ISL_2685560, EPI_ISL_2685567, EPI_ISL_2685583, EPI_ISL_2685589, EPI_ISL_2685590, EPI_ISL_2685598, EPI_ISL_2685604, EPI_ISL_2685613, EPI_ISL_2685618, EPI_ISL_2685637, EPI_ISL_2685666, EPI_ISL_2685669, EPI_ISL_2685671, EPI_ISL_2685676, EPI_ISL_2685683, EPI_ISL_2685696, EPI_ISL_2685715, EPI_ISL_2685721, EPI_ISL_2685723, EPI_ISL_2685740, EPI_ISL_2685755, EPI_ISL_2685767, EPI_ISL_2685808, EPI_ISL_2685833			
see above	Statens Serum Institut Bioinformatics and Microbial Genomics	Statens Serum Institut Bioinformatics and Microbial Genomics	Danish Covid-19 Genome Consortium
EPI_ISL_2685892, EPI_ISL_2685893	Department of Clinical Microbiology	GIGA Medical Genomics	Keith Durkin, Maria Artesi, Sébastien Bontems, Raphaël Boreux, Bouchra Boujemla, Nathalie Renotte, Cécile Meex, Pierrette Melin, Marie-Pierre Hayette, Vincent Bours
EPI_ISL_2686178	Amazon Dx, AUV2 Laboratory	Amazon Dx, AUV2 Laboratory	Luke Meredith
EPI_ISL_2686618, EPI_ISL_2686619, EPI_ISL_2686656, EPI_ISL_2686657, EPI_ISL_2686694, EPI_ISL_2686739, EPI_ISL_2686740, EPI_ISL_2686751, EPI_ISL_2686786, EPI_ISL_2686789	Fulgent Genetics	Centers for Disease Control and Prevention Division of Viral Diseases, Pathogen Discovery	Dakota Howard, Dhwani Batra, Peter W. Cook, Kara Moser, Adrian Paskey, Jason Caravas, Benjamin Rambo-Martin, Shatavia Morrison, Christopher Gulvick, Scott Sammons, Yvette Unoarumhi, Darlene Wagner, Matthew Schmerer, Harry Gao, Mickey Li, John Gao, Joseph Fierro, Benafsh Sapra, Becky Tsai, Yan Meng, Doreen Ng, James Xie, Clinton R. Paden, Duncan MacCannell
EPI_ISL_2686812, EPI_ISL_2686823, EPI_ISL_2686824, EPI_ISL_2686825, EPI_ISL_2686831, EPI_ISL_2686834, EPI_ISL_2686835, EPI_ISL_2686836	EHNV	Laboratory of genomics and metagenomics	Trestan Pillonel, Damien Jacot, Sébastien Aeby, Gilbert Greub, Claire Bertelli
EPI_ISL_2687999	Department of Public Health Bucharest	National Institute of Infectious Diseases-Prof. Dr. Matei Bals Molecular Diagnostics Laboratory	Corina Casangiu, Leontina Banica, Marius Surleac, Petre Milu, Robert Hohan, Simona Paraschiv, Dan Otelea
EPI_ISL_2688239	IN State Department of Health Laboratory Services	IN State Department of Health Laboratory Services	Cassandra Campion, Jamie Yeadon, Brian Pope, Lixia Liu, Kyle Brownlee, Melissa Hindenlang, Mark Glazier
EPI_ISL_2689088	MD PHL	MD PHL	Maryland Department of Health Laboratories Administration
EPI_ISL_2690195	Institute of Virology, Biomedical Research Center of the Slovak Academy of Sciences, Bratislava	Faculty of Natural Sciences, Comenius University, Bratislava	Viktoria Cabanova, Kristina Borsova, Brona Brejova, Viktoria Hodorova, Sabina Fumacova Havlikova, Juraj Kopacek, Martina Lickova, Lubomira Lukacikova, Martina Nebohacova, Monika Slavikova, Tomas Vinar, Jozef Nosek, Boris Klempa
EPI_ISL_2690210	Institute of Virology, Biomedical Research Center of the Slovak Academy of Sciences, Bratislava	Faculty of Natural Sciences, Comenius University, Bratislava	Brona Brejova, Viktoria Cabanova, Kristina Borsova, Viktoria Hodorova, Sabina Fumacova Havlikova, Juraj Kopacek, Martina Lickova, Lubomira Lukacikova, Martina Nebohacova, Monika Slavikova, Tomas Vinar, Jozef Nosek, Boris Klempa
EPI_ISL_2690355	Arizona State University	Arizona State University	Peter T. Skidmore, LaRinda A. Holland, Matthew F. Smith, Rabia Maqsood, Nicholas J. Mellor, Joy M. Blain, Valerie Harris, Joshua LaBaer, Vel Murugan, Efrem S. Lim
EPI_ISL_2692715	Salud Digna	Instituto Nacional de Medicina Genomica	Cedro-Tanda A, Hidalgo-Miranda A, Mendoza-Vargas A, Reyes-Grajeda JP, Abraham Campos-Romero, Moreno-Camacho José Luis, Rodriguez-Gallegos Jorge, Luna-Ruiz Marco, Gonzalez-Barrera D, Rangel-DeLeon D, Munguia-Garza P, Ramirez-Vega O, Escobar-Arrazola, M, Herrera-Montalvo LA.
EPI_ISL_2693065	NHLS_VIRO	KRISP, KZN Research Innovation and Sequencing Platform	Giandhari Jennifer, Pillay Sureshnee, Yajna Ramphal, Naidoo Yeshnee, Tshabuila Derek,Tegally Houriiyah, San James, Wilkinson Eduan, de Oliveira Tulio
EPI_ISL_2693118, EPI_ISL_2693120, EPI_ISL_2693123, EPI_ISL_2693124, EPI_ISL_2693265, EPI_ISL_2693269, EPI_ISL_2693270, EPI_ISL_2693273, EPI_ISL_2693275, EPI_ISL_2693276, EPI_ISL_2693279, EPI_ISL_2693280, EPI_ISL_2693281			
see above	UW Virology Lab	UW Virology Lab	Pavitra Roychoudhury, Hong Xie, Lasata Shrestha, Tien V. Nguyen, Shah Mohamed Bakhash, Michelle Lin, Noah R. Baker, Ricardo Perez, Sean Ellis, Nathan Breit, Robert J. Livingston, Meei-Li Huang, Keith R Jerome, Patrick Mathias, Alexander Greninger
EPI_ISL_2693823	UW Virology Lab	UW Virology Lab	Pavitra Roychoudhury, Hong Xie, Lasata Shrestha, Shah Mohamed Bakhash, Tien V. Nguyen, Noah R. Baker, Sean Ellis, Meei-Li Huang, Keith R Jerome, Alexander Greninger
EPI_ISL_2693916, EPI_ISL_2693917, EPI_ISL_2693918	Virology Unit, Institut Pasteur du Cambodge	Virology Unit, Institut Pasteur du Cambodge	Cecile Troupin, Leakhena Pum, Jurre Y Siegers, Ly Sovann, Kraing Sidonn, Yi Sengdoeurn, Chin Savuth, Chau Darapheak, Veasna Duong, Erik A Karlsson
EPI_ISL_2694788, EPI_ISL_2694796	Diagnostyka. Laboratoria Medyczne.	1. ViroGenetics - BSL3 Laboratory of Virology, Maopolska Centre of Biotechnology, Jagiellonian University; 2. Diagtron Laboratoria ukasz Rbalski	Rabalski L., Gromowski,T., Rogalska-Kupiec M., Swadzba J., Wydmanski,W., Mazur-Panasiuk,N., Kowalski,M., Maciej Kosinski, Natalia Derewonko, Szulc,P., Sylwia Januszczak, Labaj,P.P., Pyrc,K.
EPI_ISL_2695060, EPI_ISL_2695061, EPI_ISL_2695062, EPI_ISL_2695063, EPI_ISL_2695064	H Divino Espirito Santo - Ponta Delgada	Instituto Nacional de Saude (INSA)	Borges et al
EPI_ISL_2695256	SESARAM	Instituto Nacional de Saude (INSA)	Borges et al
EPI_ISL_2695267	CHU Sao Joao, Porto	Instituto Nacional de Saude (INSA)	Borges et al
EPI_ISL_2695382, EPI_ISL_2695386	Labeto - CAB - Leiria	Instituto Nacional de Saude (INSA)	Borges et al
EPI_ISL_2697675	National Platform bis UMONS/Jolimont	National Platform bis UMONS/Jolimont	François Dufrasne, Guillaume Bayon-Vicente, Florian Juszczak, Gautier Detry, Ruddy Wattiez
EPI_ISL_2707632, EPI_ISL_2707634, EPI_ISL_2707691, EPI_ISL_2707701, EPI_ISL_2707702, EPI_ISL_2708085	Originating lab: Wales Specialist Virology Centre Sequencing lab: Pathogen Genomics Unit	Public Health Wales Microbiology Cardiff Wales Specialist Virology Centre	Catherine Moore, Johnathan Evans, Laura Gifford, Malorie Perry, Simon Cottrell, Angela Marchbank, Alec Birchley, Alexander Adams, Amy Gaskin, Bree Gatica-Wilcox, Jason Coombes, Joel Southgate, Lauren Gilbert, Lee Graham, Nicole Pacchiarini, Sara Kumziene-Summerhayes, Sarah Taylor, Sophie Jones, Sara Rey, Matthew Bull, Joanne Watkins, Sally Corden, Tom Connor
EPI_ISL_2709822	MD PHL	MD PHL	Maryland Department of Health Laboratories Administration
EPI_ISL_2710223, EPI_ISL_2710224	HOSPITAL UNIVERSITARIO SON ESPASES	HOSPITAL UNIVERSITARIO SON ESPASES	Servicio de Microbiología, Hospital Universitario Son Espases
EPI_ISL_2712843, EPI_ISL_2712844	Hospital Universitari Vall d'Hebron - Vall d'Hebron Institut de Recerca	Hospital Universitari Vall d'Hebron - Vall d'Hebron Institut de Recerca	Cristina Andrés, Maria Piñana, Alejandra González-Sánchez, Damir Garcia-Cehic, Ariadna Rando, Juliana Esperalba, Maria Gema Codina, Carla Castillo, Maria Carmen Martin, Tomàs Pumarola, Josep Quer, Andrés Antón
EPI_ISL_2712953, EPI_ISL_2712956	Wyoming Public Health Laboratory	Wyoming Public Health Laboratory	Jim Mildenberger, Taylor Fearing, Channing Weber, Ashley Norberg, Chayse Rowley, Marley Goetz, Brian Dominguez, Elliot Thomasson, Sam Britz, Cari Sloma, Robert Petit, and Rob Christensen

EPI_ISL_2713054	University of Wisconsin-Madison AIDS Vaccine Research Laboratories	University of Wisconsin-Madison AIDS Vaccine Research Laboratories	Gage Moreno, Katarina Braun, et al. AIDS Vaccine Research Laboratories
EPI_ISL_2713063	Oregon State Public Health Laboratory	Oregon State Public Health Laboratory	Rafia Razzaque, Eugene Yeboah, Vanda Makris, Laura Tsaknaridis, John Fontana and Shane Sevey
EPI_ISL_2716209	The Jackson Laboratory	The Jackson Laboratory	Long J, Renzette N, Adams M, Omerza G, Kelly K, Li L
EPI_ISL_2716295	Kansas Health and Environmental Lab	Kansas Health and Environmental Lab	Katherine Wiggins, Mike Grose, Jonathan Barnell, Ben Olsen, and Phil Adam
EPI_ISL_2716934, EPI_ISL_2716936	Southern Nevada Public Health Laboratory	Southern Nevada Public Health Laboratory	Michael Picker
EPI_ISL_2718506, EPI_ISL_2718525	Lighthouse Lab in Glasgow	Wellcome Sanger Institute for the COVID-19 Genomics UK (COG-UK) Consortium	Harper VanSteenhouse, Yumi Kasai, David Gray, Carol Clugston, Anna Dominiczak and Alex Alderton, Roberto Amato, Jeffrey Barrett, Sonia Goncalves, Ewan Harrison, David K. Jackson, Ian Johnston, Dominic Kwiatkowski, Cordelia Langford, John Sillitoe on behalf of the Wellcome Sanger Institute COVID-19 Surveillance Team
EPI_ISL_2724393, EPI_ISL_2724394	Viollier AG	Department of Biosystems Science and Engineering, ETH Zürich	Christian Beisel, Sarah Nadeau, Chaoran Chen, Ivan Topolsky, Kim Philipp Jablonski, Lara Fuhrmann, Rebecca Denes, Mirjam Feldkamp, Ina Nissen, Natascha Santacroce, Elodie Burcklen, Christiane Beckmann, Maurice Redondo, Olivier Kobel, Christoph Noppen, Niko Beerenwinkel, Tanja Stadler
EPI_ISL_2725105	Innlandet Hospital Trust, Division Lillehammer, Department for Medical Microbiology	Norwegian Institute of Public Health, Department of Virology	Kathrine Stene-Johansen, Kamilla Heddeland Instefjord, Hilde Elshaug, Garcia Llorente Ignacio, Jon Bråte, Engebretsen Serina Beate,Pedersen Benedikte Nevjen, Line Victoria Moen, Debech Nadia, Atiya R Ali,Marie Paulsen Madsen, Rasmus Riis Kopperud, Hilde Vollan, Karoline Bragstad, Olav Hungnes
EPI_ISL_2725185, EPI_ISL_2725187, EPI_ISL_2725188, EPI_ISL_2725189, EPI_ISL_2725202, EPI_ISL_2725203	Lab. Microbiologia e Virologia Cotugno A.O. dei Colli - Istituto Zooprofilattico Sperimentale del Mezzogiorno	Telethon Institute of Genetics and Medicine (TIGEM)	Antonio Grimaldi Patrizia Annunziata Francesco Panariello Biancamaria Pierri Claudia Tiberio Teresa Giuliano Valentina Bouche Chiara Colantuono Maria Concetta Cuomo Denise Di Concilio Lucio Di Filippo Anna Manfredi Marcello Salvi Antonio Limone Luigi Atripaldi Pellegrino Cerino Andrea Ballabio Davide Cacchiarelli
EPI_ISL_2725545, EPI_ISL_2725548	Azienda Sanitaria dell'Alto Adige - Laboratorio Aziendale di Microbiologia e Virologia	Azienda Sanitaria dell'Alto Adige	Irene Bianconi
EPI_ISL_2726701	Eurofins-NMDL	Eurofins-NMDL	Thierry Janssens, Anne Vogel, Lisa Dreesens, Roy Masius, Katja Plathan, Anco Molijn, Maurine Leversteijn-van Hall, Thomas van der Toorn, Cornel Brouwer, Diviana Lakker, Hamza Saleem, Layonel Jacobus, Rosalie Van der Stap, Marisol Williams, Chris van Koppen, Senay Ozturk, Marvin Ruiter, Mark de Jong, Alexandra Hansen, Coen de Jong, Madhavi Kharagjitsing, Ramon Koers, Rens Driel, Thomas van der Toorn, Bram Weijers, Chantal Schenker, Humaira Yousaf, Jelmer Eerland, Melek Aras, Rousjan Amir, Enes Sekerci, Goyaert Roosen, Laura Weijenberg, Mathijs Wielen, Nienke Meijer, Tobias van der Heijde, Chinmoy Saha, Glenn Bohms, Iman El-Idrioui, Jorian Flik, Lida Cen, Simon Lansu
EPI_ISL_2727017	National Institute of Public Health	National Institute of Public Health	Helena Jirincova, Jaromira Vecerova, Timotej Suri, Dusan Trnka, Alexander Nagy
EPI_ISL_2727060	MD PHL	MD PHL	Maryland Department of Health Laboratories Administration
EPI_ISL_2727580	NJDOH, Public Health and Environmental Laboratories	NJ_PHEL	Lindsey Bodnar, Shiv K. Verma, Jacquelyn Deverell, Ryan Pachucki, Dana Woell, Allison Roder, Byeong Jeong
EPI_ISL_2727815, EPI_ISL_2727819, EPI_ISL_2727821, EPI_ISL_2727822, EPI_ISL_2727829	Wisconsin State Laboratory of Hygiene Communicable Disease Division	Wisconsin State Laboratory of Hygiene Communicable Disease Division	Abigail C. Shockey, Alicia J. Mooney, Erika M. Hanson, Tonya Danz, Richard Griesser, Sara Wagner, Kelsey R. Florek
EPI_ISL_2728621, EPI_ISL_2728626	SYNLAB	GIGA Medical Genomics	Keith Durkin, Maria Artesi, Bouchra Boujemla, Nathalie Renotte, Nadine Cambisano, Sébastien Bontems, Cécile Meex, Claire Gourzonès, Olivier Ek, Laurent Gillet, Marie-Pierre Hayette, Vincent Bours
EPI_ISL_2731154, EPI_ISL_2731166, EPI_ISL_2731184, EPI_ISL_2731206, EPI_ISL_2731230, EPI_ISL_2731239, EPI_ISL_2731249, EPI_ISL_2731257, EPI_ISL_2731258, EPI_ISL_2731259, EPI_ISL_2731260			
see above	Lighthouse Lab in Glasgow	Wellcome Sanger Institute for the COVID-19 Genomics UK (COG-UK) Consortium	Harper VanSteenhouse, Yumi Kasai, David Gray, Carol Clugston, Anna Dominiczak and Alex Alderton, Roberto Amato, Jeffrey Barrett, Sonia Goncalves, Ewan Harrison, David K. Jackson, Ian Johnston, Dominic Kwiatkowski, Cordelia Langford, John Sillitoe on behalf of the Wellcome Sanger Institute COVID-19 Surveillance Team
EPI_ISL_2731353, EPI_ISL_2731360	Microvida	Microvida	Suzan D. Pas, Jaco J. Verweij, Joep J. J. M. Stohr
EPI_ISL_2754070	Wyoming Public Health Laboratory	Wyoming Public Health Laboratory	Jim Mildenberger, Taylor Fearing, Channing Weber, Ashley Norberg, Chayse Rowley, Marley Goetz, Brian Dominguez, Elliot Thomasson, Sam Britz, Cari Sloma, Robert Petit, and Rob Christensen
EPI_ISL_2754306	Arizona State Public Health Laboratory	Arizona State Public Health Laboratory	Trung Huynh, Jessica Escobar, Katherine Fullerton, Nobuko Fukushima, Matthew Contursi, Stacy White, Linda Getsinger, Victor Waddell
EPI_ISL_2757689	Presidio di Brindisi Di Summa - Perrino	Istituto Zooprofilattico Sperimentale della Puglia e della Basilicata	Parisi A., Bianco A., Capozzi L., Del Sambro L., Simone D., Difato L., Santoro A., Ridolfi D., Giannico A.
EPI_ISL_2757700	Ospedale Vito Fazzi	Istituto Zooprofilattico Sperimentale della Puglia e della Basilicata	Parisi A., Bianco A., Capozzi L., Del Sambro L., Simone D., Difato L., Lobreglio G.
EPI_ISL_2758028	Austrian Agency for Health and Food Safety (AGES)	Bergthaler laboratory, CeMM Research Center for Molecular Medicine of the Austrian Academy of Sciences	Lukas Endler, Anna Schedl, Fabian Amman, Petr Triska, Matthew Thornton, Thomas Penz, Benedikt Agerer, Maelle Le Moing, Michael Schuster, Bekir Erguner, Jan Laine, Martin Senekowitsch, Christoph Bock, Andreas Bergthaler
EPI_ISL_2758221	Laboratoire Hôpital Sainte Musse	Laboratoire Hôpital Sainte Musse	Lionel Chollet
EPI_ISL_2758371, EPI_ISL_2758376	Berkeley Medical Center	WVU and Marshall University Combined Genomics Core Facilities	James Denvir, Peter Stoilov, Peter Perrotta, Wesley Kimble, Ryan Percifield
EPI_ISL_2758400	QLabs	WVU and Marshall University Combined Genomics Core Facilities	James Denvir, Peter Stoilov, Peter Perrotta, Wesley Kimble, Ryan Percifield
EPI_ISL_2758929	The Ohio State University Applied Microbiology Services Laboratory	The Ohio State University Applied Microbiology Services Laboratory	Seth A. Faith PhD
EPI_ISL_2759726	SYNLAB MVZ Weiden	Robert Koch Institute	unknown
EPI_ISL_2759823	Uniklinikum Regensburg; Institut für klinische Mikrobiologie und Hygiene	Robert Koch Institute	unknown
EPI_ISL_2760230, EPI_ISL_2760251, EPI_ISL_2760254, EPI_ISL_2760260	Helios Universitätsklinikum Wuppertal: Universität Witten/Herdecke: Institut für Medizinische Labordiagnostik	Robert Koch Institute	unknown
EPI_ISL_2760298	MVZ Labor Krone GbR	Robert Koch Institute	unknown
EPI_ISL_2760452	MVZ Dr. Eberhard & Partner Dortmund	Robert Koch Institute	unknown
EPI_ISL_2760460, EPI_ISL_2760466	amedes MVZ DIAMEDIS Sennestadt	Robert Koch Institute	unknown
EPI_ISL_2761153, EPI_ISL_2761156, EPI_ISL_2761164, EPI_ISL_2761254, EPI_ISL_2761316, EPI_ISL_2761453, EPI_ISL_2761489, EPI_ISL_2761533, EPI_ISL_2761556	Bioscientia Labor Wermsdorf	Robert Koch Institute	unknown
EPI_ISL_2761670, EPI_ISL_2761671	MVZ Labor Dr. Fenner und Kollegen (Standort Hamburg)	Robert Koch Institute	unknown
EPI_ISL_2761752, EPI_ISL_2761754, EPI_ISL_2761761, EPI_ISL_2761773, EPI_ISL_2761817	CENTOGENE Frankfurt Laboratory: Niederlassung Industriepark Höchst	Robert Koch Institute	unknown

EPI_ISL_2761918	Medizinisch-Diagnostisches Labor Kempten allgäulab	Robert Koch Institute	unknown
EPI_ISL_2762045, EPI_ISL_2762073	Bioscientia Labor Wermsdorf	Robert Koch Institute	unknown
EPI_ISL_2762136	IMD - MVZ Labor Greifswald	Robert Koch Institute	unknown
EPI_ISL_2762137, EPI_ISL_2762140	IMD - MVZ Labor Martinsried	Robert Koch Institute	unknown
EPI_ISL_2762228, EPI_ISL_2762272	Bayerisches Landesamt für Gesundheit und Lebensmittelsicherheit (LGL)	Robert Koch Institute	unknown
EPI_ISL_2768099	Felix-Platter Spital	Clinical Bacteriology	Tim Roloff, Fanny Wegner, Helena MB Seth-Smith, Alfredo Mari, Karoline Leuzinger, Julia Bielicki, Manuel Battegay, Hans Hirsch, Adrian Egli
EPI_ISL_2769139	Spital Männedorf AG	Institute of Medical Virology	Verena Kufner, Gabriela Ziltener, Maryam Zaheri, Stefan Schmutz, Annette Audigé, Maria Grünberg, Kevin Steiner, Jon Huder, Cyril Shah, Riccarda Capaul, Guido Bloemberg, Jürg Böni, Michael Huber, Alexandra Trkola
EPI_ISL_2769143	Swiss Analysis AG	Institute of Medical Virology	Verena Kufner, Gabriela Ziltener, Maryam Zaheri, Stefan Schmutz, Annette Audigé, Maria Grünberg, Kevin Steiner, Jon Huder, Cyril Shah, Riccarda Capaul, Guido Bloemberg, Jürg Böni, Michael Huber, Alexandra Trkola
EPI_ISL_2772668	Max von Pettenkofer Institute, Virology, National Reference Center for Retroviruses, LMU Munich	Laboratory for Functional Genome Analysis; Dept. Genomics; Gene Center of the LMU Munich	Max Muenchhoff; Stefan Krebs; Alexander Graf; Oliver Keppler; Helmut Blum
EPI_ISL_2774090	Multiplex DX	Multiplex DX	Diana Drobna, Veronika Mancikova, Silvia Rybecka, Jakub Kovac, Pavol Cekan
EPI_ISL_2774218	IRCCS San Gallicano Dermatological Institute	IRCCS Regina Elena National Cancer Institute	Ludovica Ciuffreda, Sara Donzelli, Alice Massacci, Valentina Ricca, Fabrizio Ensoli, Fulvia Pimpinelli, Maurizio Fanciulli, Giovanni Blandino, Aldo Morrone, Gennaro Ciliberto
EPI_ISL_2774268, EPI_ISL_2774269, EPI_ISL_2774271, EPI_ISL_2774282	Baylor Scott & White-Temple	Baylor Scott & White-Temple	Ari Rao, Linden Morales, Kimberly Walker, Marcus Volz, Shelby Johnson
EPI_ISL_2774440, EPI_ISL_2774450, EPI_ISL_2774451	Multiplex DX	Multiplex DX	Diana Drobna, Veronika Mancikova, Silvia Rybecka, Jakub Kovac, Pavol Cekan
EPI_ISL_2812556	Communicable Disease Laboratory, Public Health Directorate	Communicable Disease Laboratory, Public Health Directorate	Alwasti,H., AlHujairi,Z., AlAbbas,Z., Marhoon,A., Almoamen,G.

Supp. Table S3

We gratefully acknowledge the following Authors from the Originating laboratories responsible for obtaining the specimens, as well as the Submitting laboratories where the genome data were generated and shared via GISAID, on which this research is based.

All Submitters of data may be contacted directly via www.gisaid.org

Authors are sorted alphabetically.

Accession ID	Originating Laboratory	Submitting Laboratory	Authors
EPI_ISL_2171924	Lighthouse Lab in Alderley Park	Wellcome Sanger Institute for the COVID-19 Genomics UK (COG-UK) Consortium	Jacquelyn Wynn, Mairead Hyland, The Lighthouse Lab in Alderley Park and Alex Alderton, Roberto Amato, Jeffrey Barrett, Sonia Goncalves, Ewan Harrison, David K. Jackson, Ian Johnston, Dominic Kwiatkowski, Cordelia Langford, John Sillitoe on behalf of the Wellcome Sanger Institute COVID-19 Surveillance Team
EPI_ISL_2179665	National Platform bis UMONS/Jolimont	National Platform bis UMONS/Jolimont	François Dufrasne, Guillaume Bayon-Vicente, Florian Juszczak, Eric Tarantino, Gautier Detry, Ruddy Wattiez
EPI_ISL_2179795	Servicio Microbiología Hospital La Paz	Servicio Microbiología Hospital La Paz	Fernando Lázaro, Rubén Cáceres, Jesús Mingorance Cruz, Elie Dahdouh
EPI_ISL_2226240	Plateforme de testing Namuroise	Plateforme de testing Namuroise	Lesly Nyinkeu Kemamen; Otto Gaetan ; Denis Olivier ; Degosserie Jonathan ; Mullier François
EPI_ISL_2228178, EPI_ISL_2228204	UMC Groningen, Clinical Virology, Department of Medical Microbiology and Infection Prevention	UMC Groningen, Clinical Virology, Department of Medical Microbiology and Infection Prevention	Hubert Niesters, Alexander Friedrich, Erley Lizarazo-Forero, Monika Fliss, Lilli Gard, Sigrid Rosema, Coretta Van Leer-Buter, Xuewei Zhou, Marjolein Knoester
EPI_ISL_2228863, EPI_ISL_2228865	CH.INTERCOMMUNAL DE CRETEIL	Department of Virology, Henri Mondor University Hospital, Assistance Publique Hôpitaux de Paris, Université Paris-Est Créteil, INSERM U955	Christophe Rodriguez, Slim Fourati, Vanessa Demontant, Guillaume Gricourt, Melissa N'Debi, Alexandre Soulier, Elisabeth Trawinski, Jean-Michel Pawlotsky
EPI_ISL_2235024, EPI_ISL_2235031	Pandemic Response Lab - NYC	Pandemic Response Lab, R&D	Henry Lee, Michael Hammerling, Melissa Hopkins, Cybill del Castillo, Shinyoung Clair Kang, William Ward, Pradeep Bugga, Sol Rey, Dylan Law, Katharine Nelson, Haiping Hao, Jon Laurent
EPI_ISL_2245449	University of Liège COVID-19 testing center	GIGA Medical Genomics	Keith Durkin, Maria Artesi, Bouchra Boujemla, Nathalie Renotte, Cécile Meex, Sébastien Bontems, Fabrice Bureau, Laurent Gillet, Wouter Coppieters, Marie-Pierre Hayette, Vincent Bours
EPI_ISL_2253116, EPI_ISL_2253117, EPI_ISL_2253124	SARS-CoV-2 testing team, National Institute of Infectious Diseases	Pathogen Genomics Center, National Institute of Infectious Diseases	Tsuyoshi Sekizuka, Kentaro Itokawa, Rina Tanaka, Masanori Hashino, Nozomu Hanaoka, Masumichi Saito, Naomi Nojiri, Hazuka Y Furihata, Sana Uchikoba, Tsuguto Fujimoto, Makoto Kuroda
EPI_ISL_2262449	MVZ Labor Dr. Limbach & Kollegen GbR	Robert Koch Institute	unknown
EPI_ISL_2263792, EPI_ISL_2263795, EPI_ISL_2263840, EPI_ISL_2263936	SYNLAB MVZ Leverkusen	Robert Koch Institute	unknown
EPI_ISL_2264542	MDI Limbach Berlin GmbH; MVZ Labor Berlin	Robert Koch Institute	unknown
EPI_ISL_2265003	Medizinisches Labor Wahl Lüdenscheid	Robert Koch Institute	unknown
EPI_ISL_2265085	SYNLAB MVZ Leinfelden-Echterdingen	Robert Koch Institute	unknown
EPI_ISL_2266111	Labor Dr. Heidrich & Kollegen MVZ GmbH Hamburg	Robert Koch Institute	unknown
EPI_ISL_2266668	SYNLAB MVZ Trier	Robert Koch Institute	unknown
EPI_ISL_2266982	SYNLAB MVZ Leverkusen	Robert Koch Institute	unknown
EPI_ISL_2267030	Synlab MVZ Augsburg	Robert Koch Institute	unknown
EPI_ISL_2267112, EPI_ISL_2267131, EPI_ISL_2267148, EPI_ISL_2267222	SYNLAB MVZ Leverkusen	Robert Koch Institute	unknown
EPI_ISL_2267408, EPI_ISL_2267536	SYNLAB MVZ Heidelberg	Robert Koch Institute	unknown
EPI_ISL_2267636, EPI_ISL_2267663, EPI_ISL_2267761	SYNLAB MVZ Leverkusen	Robert Koch Institute	unknown
EPI_ISL_2268185	CENTOGENE Frankfurt Laboratory: Niederlassung Industriepark Höchst	Robert Koch Institute	unknown
EPI_ISL_2272557	Alaska State Virology Laboratory	Alaska State Virology Laboratory	Stephanie DeRonde, Elva House, Jacob Zidek, Lisa Smith, Ph.D., Jack Chen, Ph.D.
EPI_ISL_2272765	Platform BIS UZA/UAntwerpen	Labo Klinische Biologie, UZA	Marie Le Mercier, Jasmine Coppens, Basil Britto Xavier, Christine Lammens, Veerle Matheeussen, Herman Goossens
EPI_ISL_2274245	PHV-FSS	PHV-FSS	Son Nguyen
EPI_ISL_2280872	Aegis Sciences Corporation	Centers for Disease Control and Prevention Division of Viral Diseases, Pathogen Discovery	Dakota Howard, Dhwani Batra, Peter W. Cook, Kara Moser, Adrian Paskey, Jason Caravas, Benjamin Rambo-Martin, Shatavia Morrison, Christopher Gulvick, Scott Sammons, Yvette Unoarumhi, Darlene Wagner, Matthew Schmerer, Cyndi Clark, Patrick Campbell, Rob Case, Vikramsinha Ghorpade, Holly Houdeshell, Ola Kvalvaag, Dillon Nall, Ethan Sanders, Alec Vest, Shaun Westlund, Matthew Hardison, Clinton R. Paden, Duncan MacCannell
EPI_ISL_2289468	LABORATOIRE DE BIOLOGIE MEDICALE	CNR Virus des Infections Respiratoires - France SUD	Antonin Bal, Gregory Destras, Gwendolyne Burfin, Hadrien Regue, Quentin Semanas, Martine Valette, Bruno Lina, Laurence Josset
EPI_ISL_2293466	Hospital	National Reference Center for Viruses of Respiratory Infections, Institut Pasteur, Paris	Marion Barbet, Sylvie Behillil, Méline Bizard, Angela Brisebarre, Camille Capel, Vincent Enouf, Louise Lefrançois, Frédéric Lemoine, Christophe Malabat, Corinne Maufrais, Etienne Simon-Lorière, Maud Vanpeene, Sylvie Van der Werf ,Laurence Louvet
EPI_ISL_2303970	Dutch COVID-19 response team	National Institute for Public Health and the Environment (RIVM)	Adam Meijer, Harry Vennema, Dirk Eggink, Jeroen Cremer, Sharon van den Brink, Bas van der Veer, AnneMarie van den Brandt, Lisa Wijsman, Kim Freriks, Ryanne Jaarsma, Euníce Then, Lynn Aarts, Sanne Bos, Melissa van Tuil, Linda van de Nes, Sjoerd Kuiling, James Groot, Florian Zwagemaker, Dennis Schmitz, Annelies Kroneman, Karim Hajji, Chantal Reusken, on behalf of the national COVID-19 response team
EPI_ISL_2305050, EPI_ISL_2306333, EPI_ISL_2306752, EPI_ISL_2306814, EPI_ISL_2306873	Laboratory Corporation of America	Centers for Disease Control and Prevention Division of Viral Diseases, Pathogen Discovery	Dakota Howard, Dhwani Batra, Peter W. Cook, Kara Moser, Adrian Paskey, Jason Caravas, Benjamin Rambo-Martin, Shatavia Morrison, Christopher Gulvick, Scott Sammons, Yvette Unoarumhi, Darlene Wagner, Matthew Schmerer, Minoo Agarwal, Eyad Almasri, Debbie Boles, Ayla Burns, Nuthawin Charoensri, Oren Cohen, Susan Countryman, Mary Ann Cristobal, Bobbi Croy, Suzanne Dale, Hrushikesh Deshmukh, Amanda Douglas, Vincent Drouillon, Marcia Eisenberg, Howard Engler, Rama Ghatti, Prashant Gupta, Susan Hicks, Jake Humphrey, Lax Iyer, Manoj Jain, Mohan Kolli, Brian Krueger, Tim Kuphal, Stanley Letovsky, Michael Levandoski, Craig Lukasik, Jonathan Meltzer, Brian Norvell, Mindy Nye, Scott Parker, Christos Petropoulos, John Pruitt, Steven Ragan, Scott Ryan, Mike Sapeta, Jana Schroth, Suresh Babu Selvaraju, Goran Stevovic, Amanda Suchanek, Andrea Throop, Lyndon Tilson, Thomas Urban, Joe Voshell, Kimberly Wagner, Jonathan Williams, Mary Williamson, Qian Zeng, Tricia Zwiefelhofer, Clinton R. Paden, Duncan MacCannell
EPI_ISL_2308371	Nebraska Public Health Laboratory	NPHL COVID-19 Response Team	NPHL COVID-19 Response Team
EPI_ISL_2313084	Department of Virology	Department of Virology	Massab Umair, Aamer Ikram, Muhammad Salman, Nazish Badar, Zaira Rehman, Muhammad Ammar, Adnan Haider
EPI_ISL_2313118, EPI_ISL_2313181	Limbach - MVZ Labor Eveld & Kollegen Essen	Robert Koch Institute	unknown
EPI_ISL_2313339	Robert Koch-Institut ZBS1	Robert Koch Institute	Annika Brinkmann
EPI_ISL_2313398	Bioscientia MVZ Labor Karlsruhe GmbH	Robert Koch Institute	unknown

EPI_ISL_2313849	Labor 28 MVZ GmbH	Robert Koch Institute	unknown
EPI_ISL_2313893	Laborarztpraxis Osnabrück	Robert Koch Institute	unknown
EPI_ISL_2314139	LabKom - Labor Hannover MVZ GmbH	Robert Koch Institute	unknown
EPI_ISL_2314141	Bioscientia MVZ Labor Karlsruhe GmbH	Robert Koch Institute	unknown
EPI_ISL_2314185	Department of Virology	Department of Virology	Massab Umair, Aamer Ikram, Muhammad Salman, Nazish Badar, Zaira Rehman, Muhammad Ammar, Adnan Haider
EPI_ISL_2314239, EPI_ISL_2314251	Sonic - Labor Dr. von Froreich GmbH	Robert Koch Institute	unknown
EPI_ISL_2314738	MVZ für Laboratoriumsmedizin und Mikrobiologie Koblenz-Mittelrhein (Labor Koblenz)	Robert Koch Institute	unknown
EPI_ISL_2314753	Labor Prof. Dr. G. Enders MVZ GbR	Robert Koch Institute	unknown
EPI_ISL_2314773	Limbach - MVZ Labor Ludwigsburg	Robert Koch Institute	unknown
EPI_ISL_2315020	Diagnosticum - Labor Neukirchen	Robert Koch Institute	unknown
EPI_ISL_2315141, EPI_ISL_2315167, EPI_ISL_2315218	SYNLAB MVZ Leverkusen	Robert Koch Institute	unknown
EPI_ISL_2316262	SYNLAB MVZ Leinfelden-Echterdingen	Robert Koch Institute	unknown
EPI_ISL_2316615, EPI_ISL_2316635	Klinikum Ernst von Bergmann gemeinnützige GmbH - stationärer Bereich	Robert Koch Institute	unknown
EPI_ISL_2320451	Helix/Illumina	Centers for Disease Control and Prevention Division of Viral Diseases, Pathogen Discovery	Dakota Howard, Dhwani Batra, Peter W. Cook, Kara Moser, Adrian Paskey, Jason Caravas, Benjamin Rambo-Martin, Shatavia Morrison, Christopher Gulvick, Scott Sammons, Yvette Unoarumhi, Darlene Wagner, Matthew Schmerer, Eileen de Feo, Jan Antico, Christine Tran, Matthew Tolentino, Shannon Wickline, Kim Gietzen, Brad Sickler, Jingtao Liu, Eric Allen, Phil Febbo, Nicole L. Washington, Simon White, Geraint Levan, Kelly Schiabor Barrett, Elizabeth Cirulli, Alexandre Bolze, Ary Ascencio, Charlotte Rivera-Garcia, Ryan Cho, Jason Nguyen, Sherry Wang, Jimmy Ramirez, Tyler Cassens, Efren Sandoval, Magnus Isaksson, William Lee, David Becker, Marc Laurent, James Lu, Clinton R. Paden, Duncan MacCannell
EPI_ISL_2323153	Nebraska Public Health Laboratory	NPHL COVID-19 Response Team	NPHL COVID-19 Response Team
EPI_ISL_2325091	Mayo Clinic & Mayo Clinic Laboratories	Minnesota Department of Health, Public Health Laboratory	Alexandra Lorentz, Jacob Garfin, Matt Plumb, and Xiong Wang
EPI_ISL_2332810	OLVZ Aalst	OLVZ Aalst	Anne Vankeerberghen
EPI_ISL_2332879	UW Virology Lab	UW Virology Lab	Pavitra Roychoudhury, Hong Xie, Lasata Shrestha, Tien V. Nguyen, Shah Mohamed Bakhash, Michelle Lin, Noah R. Baker, Ricardo Perez, Sean Ellis, Nathan Breit, Robert J. Livingston, Meei-Li Huang, Keith R Jerome, Patrick Mathias, Alexander Greninger
EPI_ISL_2333707	CH.INTERCOMMUNAL DE CRETEIL	Department of Virology, Henri Mondor University Hospital, Assistance Publique Hôpitaux de Paris, Université Paris-Est Créteil, INSERM U955	Christophe Rodriguez, Slim Fourati, Vanessa Demontant, Guillaume Gricourt, Melissa N'Debi, Alexandre Soulier, Elisabeth Trawinski, Jean-Michel Pawlotsky
EPI_ISL_2333760, EPI_ISL_2333761, EPI_ISL_2333762	Hôpital Avicenne	Department of Virology, Henri Mondor University Hospital, Assistance Publique Hôpitaux de Paris, Université Paris-Est Créteil, INSERM U955	Christophe Rodriguez, Slim Fourati, Vanessa Demontant, Guillaume Gricourt, Melissa N'Debi, Alexandre Soulier, Elisabeth Trawinski, Jean-Michel Pawlotsky
EPI_ISL_2333806	GH A.CHENEVIER-H.MONDOR	Department of Virology, Henri Mondor University Hospital, Assistance Publique Hôpitaux de Paris, Université Paris-Est Créteil, INSERM U955	Christophe Rodriguez, Slim Fourati, Vanessa Demontant, Guillaume Gricourt, Melissa N'Debi, Alexandre Soulier, Elisabeth Trawinski, Jean-Michel Pawlotsky
EPI_ISL_2339296	Arizona State University	Arizona State University	Peter T. Skidmore, LaRinda A. Holland, Matthew F. Smith, Rabia Maqsood, Nicholas J. Mellor, Joy M. Blain, Valerie Harris, Joshua LaBaer, Vel Murugan, Efrem S. Lim
EPI_ISL_2339612	UMC Groningen, Clinical Virology, Department of Medical Microbiology and Infection Prevention	UMC Groningen, Clinical Virology, Department of Medical Microbiology and Infection Prevention	Hubert Niesters, Alexander Friedrich, Erley Lizarazo-Forero, Monika Fliss, Lilli Gard, Sigrid Rosema, Coretta Van Leer-Buter, Xuewei Zhou, Marjolein Knoester
EPI_ISL_2340666	University of Wisconsin-Madison AIDS Vaccine Research Laboratories	University of Wisconsin-Madison AIDS Vaccine Research Laboratories	Gage Moreno, Katarina Braun, et al. AIDS Vaccine Research Laboratories
EPI_ISL_2347125, EPI_ISL_2347237	Lighthouse Lab in Milton Keynes	Wellcome Sanger Institute for the COVID-19 Genomics UK (COG-UK) Consortium	The Lighthouse Lab in Milton Keynes and Alex Alderton, Roberto Amato, Jeffrey Barrett, Sonia Goncalves, Ewan Harrison, David K. Jackson, Ian Johnston, Dominic Kwiatkowski, Cordelia Langford, John Sillitoe on behalf of the Wellcome Sanger Institute COVID-19 Surveillance Team
EPI_ISL_2348566	Department of Clinical Microbiology	GIGA Medical Genomics	Keith Durkin, Maria Artesi, Sébastien Bontems, Raphaël Boreux, Bouchra Boujemla, Nathalie Renotte, Cécile Meex, Pierrette Melin, Marie-Pierre Hayette, Vincent Bours
EPI_ISL_2348808, EPI_ISL_2348809, EPI_ISL_2348810	Faculty of Medicine, Prince of Songkla University	COVID-19 Network Investigations (CONI) Alliance	Elizabeth Batty, Wasun Chantratita, Thanat Chookajorn, Stefan Fernandez, Angkana Huang, Anthony R. Jones, Khajohn Joonlasak, Chonticha Klungtong, Theerarat Kochakarn, Namfon Kotanan, Krittikorn Kumpornsin, Duangkamon Loesbanluechai, Wudtichai Manasatienkij, Bhakbhoom Panthan, Ekawat Pasomsub, Kingkan Rakmanee, Insee Sensorn, Janjira Thaipadungpanit, Arporn Wangwiwatsin, Treewat Watthanachockchai, Smonrapat Surasombatpattana
EPI_ISL_2348820	Office of Diseases Prevention and Control Region 12 Songkhla	COVID-19 Network Investigations (CONI) Alliance	Elizabeth Batty, Wasun Chantratita, Thanat Chookajorn, Stefan Fernandez, Angkana Huang, Anthony R. Jones, Khajohn Joonlasak, Chonticha Klungtong, Theerarat Kochakarn, Namfon Kotanan, Krittikorn Kumpornsin, Duangkamon Loesbanluechai, Wudtichai Manasatienkij, Bhakbhoom Panthan, Ekawat Pasomsub, Kingkan Rakmanee, Insee Sensorn, Janjira Thaipadungpanit, Arporn Wangwiwatsin, Treewat Watthanachockchai, Sawanya Chantutanon
EPI_ISL_2348930	Oregon State Public Health Laboratory	Oregon State Public Health Laboratory	Rafia Razzaque, Eugene Yeboah, Vanda Makris, Laura Tsaknaridis, John Fontana and Shane Sevey
EPI_ISL_2349834	National Public Health Laboratory, National Centre for Infectious Diseases	National Public Health Laboratory, National Centre for Infectious Diseases	Tze Minn Mak, Zhenyang Zhou, Royce Ang, Lin Cui, Raymond Tzer Pin Lin
EPI_ISL_2350263	Clinical Microbiology, Infection Prevention and Control	Section for Molecular Diagnostics	Björn Hallström, Jonas Björkman
EPI_ISL_2352955	Lighthouse Lab in Milton Keynes	Wellcome Sanger Institute for the COVID-19 Genomics UK (COG-UK) Consortium	The Lighthouse Lab in Milton Keynes and Alex Alderton, Roberto Amato, Jeffrey Barrett, Sonia Goncalves, Ewan Harrison, David K. Jackson, Ian Johnston, Dominic Kwiatkowski, Cordelia Langford, John Sillitoe on behalf of the Wellcome Sanger Institute COVID-19 Surveillance Team
EPI_ISL_2361525	CHU LILLE	CHU Lille - Laboratoire de Virologie	AIT YAHYA Emilie, ALIDJINOU Enagnon Kazali, BOCKET Laurence, CREPIN Michel, DEMAY Christophe, ENGELMANN Ilka, GEFFROY Sandrine, GUIGON Aurélie, LAMBERT Valérie, LAZREK Mouna, NOBILLIAUX Florian, PREVOST Brigitte, THUILLIER Caroline, TINEZ Claire ,TCHANTCHOU NJOSSE YANICK
EPI_ISL_2361527	CH ROUBAIX	CHU Lille - Laboratoire de Virologie	AIT YAHYA Emilie, ALIDJINOU Enagnon Kazali, BOCKET Laurence, CREPIN Michel, DEMAY Christophe, ENGELMANN Ilka, GEFFROY Sandrine, GUIGON Aurélie, LAMBERT Valérie, LAZREK Mouna, NOBILLIAUX Florian, PREVOST Brigitte, THUILLIER Caroline, TINEZ Claire ,TCHANTCHOU NJOSSE YANICK
EPI_ISL_2361628	Viollier AG	Viollier AG	Andrea Patrizia Salzmann, Henriette Kurth, Christiane Beckmann, Maurice Redondo, Olivier Kobel, Christoph Noppen
EPI_ISL_2362786	Lab voor klinische biologie	Lab voor klinische biologie	Marija Janevska, Hannelore Hamerlinck, Bruno Verhasselt
EPI_ISL_2366297	Lighthouse Lab in Milton Keynes	Wellcome Sanger Institute for the COVID-19 Genomics UK (COG-UK) Consortium	The Lighthouse Lab in Milton Keynes and Alex Alderton, Roberto Amato, Jeffrey Barrett, Sonia Goncalves, Ewan Harrison, David K. Jackson, Ian Johnston, Dominic Kwiatkowski, Cordelia Langford, John Sillitoe on behalf of the Wellcome Sanger Institute COVID-19 Surveillance Team
EPI_ISL_2367762	CH BETHUNE	CHU Lille - Laboratoire de Virologie	AIT YAHYA Emilie, ALIDJINOU Enagnon Kazali, BOCKET Laurence, CREPIN Michel, DEMAY Christophe, ENGELMANN Ilka, GEFFROY Sandrine, GUIGON Aurélie, LAMBERT Valérie, LAZREK Mouna, NOBILLIAUX Florian, PREVOST Brigitte, THUILLIER Caroline, TINEZ Claire ,TCHANTCHOU NJOSSE YANICK
EPI_ISL_2368377	Aegis Sciences Corporation	Centers for Disease Control and Prevention Division of Viral Diseases, Pathogen Discovery	Dakota Howard, Dhwani Batra, Peter W. Cook, Kara Moser, Adrian Paskey, Jason Caravas, Benjamin Rambo-Martin, Shatavia Morrison, Christopher Gulvick, Scott Sammons, Yvette Unoarumhi, Darlene Wagner, Matthew Schmerer, Cyndi Clark, Patrick Campbell, Rob Case, Vikramsinha Ghorpade, Holly Houdeshell,

			Ola Kvalvaag, Dillon Nall, Ethan Sanders, Alec Vest, Shaun Westlund, Matthew Hardison, Clinton R. Paden, Duncan MacCannell
EPI_ISL_2372701	Microbiologia CATLAB	Can Ruti SARS-CoV-2 Sequencing Hub (HUGTiP/IrsiCaixa/IGTP)	Marc Noguera-Julian, Pilar Armengol, Ignacio Blanco, Antoni E Bordoy, Francesc Catala-Moll, Pere-Joan Cardona, Maria Casadellà, Cristina Casañ, Gemma Clara, Bonaventura Clotet, Cristina Esteban, Montserrat Giménez, Mercedes Guerrero, Anna Not, Roger Pared
EPI_ISL_2373699	CNR Virus des Infections Respiratoires - France SUD	CNR Virus des Infections Respiratoires - France SUD	Antonin Bal, Gregory Destras, Gwendolyne Burfin, Hadrien Regue, Quentin Semanas, Martine Valette, Bruno Lina, Laurence Josset
EPI_ISL_2373713	LABORATOIRE DE BIOLOGIE MEDICALE	CNR Virus des Infections Respiratoires - France SUD	Antonin Bal, Gregory Destras, Gwendolyne Burfin, Hadrien Regue, Quentin Semanas, Martine Valette, Bruno Lina, Laurence Josset
EPI_ISL_2373755, EPI_ISL_2373796	CNR Virus des Infections Respiratoires - France SUD	CNR Virus des Infections Respiratoires - France SUD	Antonin Bal, Gregory Destras, Gwendolyne Burfin, Hadrien Regue, Quentin Semanas, Martine Valette, Bruno Lina, Laurence Josset
EPI_ISL_2373822, EPI_ISL_2373823	REUNILAB	CNR Virus des Infections Respiratoires - France SUD	Antonin Bal, Gregory Destras, Gwendolyne Burfin, Hadrien Regue, Quentin Semanas, Martine Valette, Bruno Lina, Laurence Josset
EPI_ISL_2373858	LBM UNIBIO VALENTIN	CNR Virus des Infections Respiratoires - France SUD	Antonin Bal, Gregory Destras, Gwendolyne Burfin, Hadrien Regue, Quentin Semanas, Martine Valette, Bruno Lina, Laurence Josset
EPI_ISL_2373867	LBM TRONQUIERES	CNR Virus des Infections Respiratoires - France SUD	Antonin Bal, Gregory Destras, Gwendolyne Burfin, Hadrien Regue, Quentin Semanas, Martine Valette, Bruno Lina, Laurence Josset
EPI_ISL_2373877	LBM UNIBIO VALENTIN	CNR Virus des Infections Respiratoires - France SUD	Antonin Bal, Gregory Destras, Gwendolyne Burfin, Hadrien Regue, Quentin Semanas, Martine Valette, Bruno Lina, Laurence Josset
EPI_ISL_2373952	CNR Virus des Infections Respiratoires - France SUD	CNR Virus des Infections Respiratoires - France SUD	Antonin Bal, Gregory Destras, Gwendolyne Burfin, Hadrien Regue, Quentin Semanas, Martine Valette, Bruno Lina, Laurence Josset
EPI_ISL_2374957	Viollier AG	Department of Biosystems Science and Engineering, ETH Zürich	Christian Beisel, Sarah Nadeau, Chaoran Chen, Ivan Topolsky, Philipp Jablonski, Lara Fuhrmann, David Dreifuss, Katharina Jahn, Rebecca Denes, Mirjam Feldkamp, Ina Nissen, Natascha Santacroce, Elodie Burcklen, Christiane Beckmann, Maurice Redondo, Olivier Kobel, Christoph Noppen, Sophie Seidel, Noemie Santamaria de Souza, Niko Beerenwinkel, Tanja Stadler
EPI_ISL_2375390	Viollier AG	Department of Biosystems Science and Engineering, ETH Zürich	Chaoran Chen, Sarah Nadeau, Catharine Aquino, Ivan Topolsky, Philipp Jablonski, Lara Fuhrmann, David Dreifuss, Katharina Jahn, Daniel Ehrsam, Isabel Stürmer, Andreia Cabral de Gouvea, Maria Domenica Moccia, Simon Grüter, Timothy Sykes, Lennart Opitz, Griffin White, Laura Neff, Doris Popovic, Andrea Patrignani, Jay Tracy, Ralph Schlapbach, Christiane Beckmann, Maurice Redondo, Olivier Kobel, Christoph Noppen, Sophie Seidel, Noemie Santamaria de Souza, Niko Beerenwinkel, Tanja Stadler
EPI_ISL_2375591	Infinity Biologix	Centers for Disease Control and Prevention Division of Viral Diseases, Pathogen Discovery	Dakota Howard, Dhwani Batra, Peter W. Cook, Kara Moser, Adrian Paskey, Jason Caravas, Benjamin Rambo-Martin, Shatavia Morrison, Christopher Gulvick, Scott Sammons, Yvette Unoarumhi, Darlene Wagner, Matthew Schmerer, Christian Bixby, Yihe Wang, Jonathan Schultz, Chirayu Goswami, Russ Hager, Robin Grimwood, Clinton R. Paden, Duncan MacCannell
EPI_ISL_2376117	NJDOH, Public Health and Environmental Laboratories	NJ_PHEL	Lindsey Bodnar, Shiv K. Verma, Jacquelyn Deverell, Dana Woell, Allison Roder, Byeong Jeong
EPI_ISL_2376256	Gundersen Clinical Microbiology Laboratory	Kabara Cancer Research Institute	Craig S. Richmond, Paraic A. Kenny
EPI_ISL_2376587	M Health Fairview	Minnesota Department of Health, Public Health Laboratory	Alexandra Lorentz, Jacob Garfin, Matt Plumb, and Xiong Wang
EPI_ISL_2378708	Department of Health Technology and Informatics, The Hong Kong Polytechnic University	Department of Health Technology and Informatics, The Hong Kong Polytechnic University	Cheng,V.C.-C., Siu,G.K.-H., Wong,S.-C., Chen,H., Lee,L.-K., Leung,J.S.-L., Lu,K.K., Chan,C.T.-M., Lo,H.W.-H., Leung,K.S.-S., Wong,E.Y.-K., Luk,S., Ng,T.T.-L., Jim,H.-C., Lao,H.-Y., Wong,D.S.-H., Tam,K.K.-G., Mok,K.K.-S., Wong,K.N., Yeh,E.Y.-W., Lam,B.P.-H., Lam,J.Y.-W., Wu,A.K.-L., Yau,M.C.-Y., Lai,Y.W.-M., Ho,A.Y.-M., Leung,W.-S., Chan,M.-C., Lam,B.H.-S., To,W.-K., Lee,R.A., Lung,D.C., Tse,H., To,K.K.-W., Yuen,K.-Y.
EPI_ISL_2378890	Washington State Department of Health Public Health Laboratories	Washington State Department of Health Public Health Laboratories	Drew MacKellar, Philip Dykema, Denny Russell, Joenice Gonzalez, Hannah Gray, Geoff Melly, Vanessa De Los Santos, Darren Lucas, JohnAric Peterson, Avi Singh, Rebecca Cao
EPI_ISL_2379302, EPI_ISL_2379426	National Platform bis UMONS/Jolimont	National Platform bis UMONS/Jolimont	François Dufrasne, Guillaume Bayon-Vicente, Florian Juszczak, Gautier Detry, Ruddy Wattiez
EPI_ISL_2382840, EPI_ISL_2382855	University of Liège COVID-19 testing center	GIGA Medical Genomics	Keith Durkin, Maria Artesi, Bouchra Boujemla, Nathalie Renotte, Cécile Meex, Sébastien Bontems, Fabrice Bureau, Laurent Gillet, Wouter Coppieters, Marie-Pierre Hayette, Vincent Bours
EPI_ISL_2382899	Jessa	Jessa	Raymaekers et al. on behalf of Jessa_cmdLab
EPI_ISL_2383950	SC Dept of Health and Env. Control-Bureau of Laboratories	Centers for Disease Control and Prevention Division of Viral Diseases, Pathogen Discovery	Mili Sheth, Sarah Nobles, Jasmine Padilla, Mark Burroughs, Shoshona Le, Katie Dillon, Peter Cook, Clinton R. Paden, Dhwani Batra, Krista Queen, Kristen Knipe, Dakota Howard, Yvette Unoarumhi, Darlene Wagner, Matthew Schmerer, Ben L. Rambo-Martin, Kristine Lacek, Sam Shepard, Alison Laufer Halpin, Dave Wentworth, Vivien Dugan, Suxiang Tong, Justin Lee
EPI_ISL_2386188	MVZ für Laboratoriumsmedizin und Mikrobiologie Koblenz-Mittelrhein (Labor Koblenz)	Robert Koch Institute	unknown
EPI_ISL_2386311	Synlab MVZ Augsburg	Robert Koch Institute	unknown
EPI_ISL_2386546	Labor Dr. Wisplinghoff - Köln	Robert Koch Institute	unknown
EPI_ISL_2386796	Labor ZOTZ|KLIMAS; MVZ Düsseldorf-Centrum	Robert Koch Institute	unknown
EPI_ISL_2388210	Laborarztpraxis Osnabrück	Robert Koch Institute	unknown
EPI_ISL_2388317	LabKom - Labor Hannover MVZ GmbH	Robert Koch Institute	unknown
EPI_ISL_2388328	LabKom - Labor Augsburg MVZ GmbH	Robert Koch Institute	unknown
EPI_ISL_2388569	Sonic - MVZ Medizinisches Labor Bremen GmbH	Robert Koch Institute	unknown
EPI_ISL_2388597, EPI_ISL_2388615	LabKom - Labor Augsburg MVZ GmbH	Robert Koch Institute	unknown
EPI_ISL_2388918	Bioscientia Labor Wermsdorf	Robert Koch Institute	unknown
EPI_ISL_2388957	Sonic - Bioscientia - MVZ Labor Saar GmbH	Robert Koch Institute	unknown
EPI_ISL_2389057	Bioscientia Labor Wermsdorf	Robert Koch Institute	unknown
EPI_ISL_2389351	Labor Dr. Heidrich & Kollegen MVZ GmbH Hamburg	Robert Koch Institute	unknown
EPI_ISL_2389582	SYNLAB MVZ Berlin	Robert Koch Institute	unknown
EPI_ISL_2389619	SYNLAB MVZ Leverkusen	Robert Koch Institute	unknown
EPI_ISL_2389744	SYNLAB MVZ Trier	Robert Koch Institute	unknown
EPI_ISL_2389848	SYNLAB MVZ Heidelberg	Robert Koch Institute	unknown
EPI_ISL_2390144	MVZ Labor Dr. Fenner und Kollegen (Standort Hamburg)	Robert Koch Institute	unknown
EPI_ISL_2392826, EPI_ISL_2395820	Lighthouse Lab in Milton Keynes	Wellcome Sanger Institute for the COVID-19 Genomics UK (COG-UK) Consortium	The Lighthouse Lab in Milton Keynes and Alex Alderton, Roberto Amato, Jeffrey Barrett, Sonia Goncalves, Ewan Harrison, David K. Jackson, Ian Johnston, Dominic Kwiatkowski, Cordelia Langford, John Sillitoe on behalf of the Wellcome Sanger Institute COVID-19 Surveillance Team
EPI_ISL_2398631, EPI_ISL_2398802, EPI_ISL_2398951, EPI_ISL_2398994, EPI_ISL_2399066, EPI_ISL_2399251, EPI_ISL_2399285	Laboratory Corporation of America	Centers for Disease Control and Prevention Division of Viral Diseases, Pathogen Discovery	Dakota Howard, Dhwani Batra, Peter W. Cook, Kara Moser, Adrian Paskey, Jason Caravas, Benjamin Rambo-Martin, Shatavia Morrison, Christopher Gulvick, Scott Sammons, Yvette Unoarumhi, Darlene Wagner, Matthew Schmerer, Minoo Agarwal, Eyad Almasri, Debbie Boles, Ayla Burns, Nuthawin Charoensri, Oren Cohen, Susan Countryman, Mary Ann Cristobal, Bobbi Croy, Suzanne Dale, Hrushikesh Deshmukh, Amanda Douglas, Vincent Drouillon, Marcia Eisenberg, Howard Engler, Rama Ghatti, Prashant Gupta, Susan Hicks, Jake Humphrey, Lax Iyer, Lisa Pfefferle, Manoj Jain, Matthew Robinson, Mohan Kolli, Brian Krueger, Tim Kuphal, Stanley Letovsky, Michael Levandoski, Craig Lukasik, Jonathan Meltzer, Brian Norvell, Mindy Nye, Scott Parker, Christos Petropoulos, John Pruitt, Steven Ragan, Scott Ryan, Mike Sapeta, Jana Schroth, Suresh Babu Selvaraju, Goran Stevovic, Amanda Suchanek, Andrea Throop, Lyndon Tilson, Thomas Urban, Joe Voshell, Kimberly Wagner, Jonathan Williams, Mary Williamson, Qian Zeng, Tricia Zwiefelhofer, Clinton R. Paden, Duncan MacCannell
EPI_ISL_2400368, EPI_ISL_2400369	University of Michigan Clinical Microbiology Laboratory	Lauring Lab, University of Michigan, Department of Microbiology and Immunology	Gilbert

EPI_ISL_2400808	Laboratoires d'analyses medicales - Ketterthill	Laboratoire national de sante, Microbiology, Microbial Genomics Platform	Anke Wienecke-Baldacchino, Catherine Ragimbeau,Jessica Tapp, Fatu Djabi, Lise Pignon, Raoul Salmon, Serge Vedy, Caroline Scheiber, Tamir Abdelrahman
EPI_ISL_2401594, EPI_ISL_2401595, EPI_ISL_2401596, EPI_ISL_2401670, EPI_ISL_2401706, EPI_ISL_2401733, EPI_ISL_2401740	BioneXt Lab	Laboratoire national de sante, Microbiology, Microbial Genomics Platform	Anke Wienecke-Baldacchino, Catherine Ragimbeau,Jessica Tapp, Fatu Djabi, Lise Pignon, Raoul Salmon, Thibault Ferrandon, Tamir Abdelrahman
EPI_ISL_2401864	Hospital Center Emile Mayrisch	Laboratoire national de sante, Microbiology, Microbial Genomics Platform	Anke Wienecke-Baldacchino, Catherine Ragimbeau,Jessica Tapp, Fatu Djabi, Lise Pignon, Raoul Salmon, Cynthia Oxacelay, Tamir Abdelrahman
EPI_ISL_2402615	Centracare Laboratory Services	Minnesota Department of Health, Public Health Laboratory	Alexandra Lorentz, Jacob Garfin, Matt Plumb, and Xiong Wang
EPI_ISL_2403347, EPI_ISL_2403353, EPI_ISL_2403354	Ministry of Health Turkey	Ministry of Health Turkey	Fatma Bayrakdar, Yasemin Cosgun, Suleyman Yalcin, Gulay Korukluoglu
EPI_ISL_2406058, EPI_ISL_2406090	Dutch COVID-19 response team	National Institute for Public Health and the Environment (RIVM)	Adam Meijer, Harry Vennema, Dirk Eggink, Jeroen Cremer, Sharon van den Brink, Bas van der Veer, AnneMarie van den Brandt, Lisa Wijsman, Kim Freriks, Ryanne Jaarsma, Euníce Then, Lynn Aarts, Sanne Bos, Melissa van Tuil, Linda van de Nes, Sjoerd Kuiling, James Groot, Florian Zwagemaker, Dennis Schmitz, Annelies Kroneman, Karim Hajji, Chantal Reusken, on behalf of the national COVID-19 response team
EPI_ISL_2406492	Middlemore Hospital	Institute of Environmental Science and Research (ESR)	Rachel Boyle, SallyAnn Harbison, Olivia Stroeven, Xiaoyun Ren, Matt Storey, Nikki Freed, Muhammad Faisal, Jing Wang, Hermes Perez, Anja Werno, Antje van der Linden, Arlo Upton, Chris Mansell, David Hammer, Dragana Drinkovic, Gary McAuliffe, Hana Sofia Andersson, James Ussher, Jill Sherwood, Josh Freeman, Julia Howard, Juliet Elvy, Mary DeAlmeida, Matt Blakiston, Matthew Rogers, Max Bloomfield, Michael Addidle, Michelle Balm, Sally Roberts, Sarah Jefferies, Sharmini Muttaiyah, Susan Morpeth, Susan Taylor, Timothy Blackmore, Vani Sathyendran, Veronica Playle, Virginia Hope, Erasmus Smit, Lauren Jelly, Olin Silander, Joep de Ligt
EPI_ISL_2406499, EPI_ISL_2406502	Laboratoire Virologie Saint Louis APHP	Laboratoire Virologie Saint Louis APHP	Maud Salmona, Marie Laure Chaix, Severine Mercier Delarue, Marie Laure Néré, Linda Feghoul, Jérome Le Goff, Constance Delauguerre, Sophia Achaibou
EPI_ISL_2408141, EPI_ISL_2408194	Swedish national genomic surveillance program of SARS-CoV-2	The Public Health Agency of Sweden	Maximilian Riess, Maria Lind Karlberg, Alma Brolund, Swedish national genomic surveillance program of SARS-CoV-2
EPI_ISL_2408469	Labor Team W Ag	Clinical Bacteriology	Tim Roloff, Fanny Wegner, Helena MB Seth-Smith, Alfredo Mari, Karoline Leuzinger, Julia Bielicki, Manuel Battegay, Hans Hirsch, Adrian Egli
EPI_ISL_2414909, EPI_ISL_2415495, EPI_ISL_2415508, EPI_ISL_2415699	Swedish national genomic surveillance program of SARS-CoV-2	The Public Health Agency of Sweden	Maximilian Riess, Maria Lind Karlberg, Alma Brolund, Swedish national genomic surveillance program of SARS-CoV-2
EPI_ISL_2417501	Department of Bacteria, Parasites and Fungi, Statens Serum Institut, Copenhagen, Denmark	Statens Serum Institut Bioinformatics and Microbial Genomics	Danish Covid-19 Genome Consortium
EPI_ISL_2418035, EPI_ISL_2418047, EPI_ISL_2418218, EPI_ISL_2418225, EPI_ISL_2418291, EPI_ISL_2418292, EPI_ISL_2418301, EPI_ISL_2418309, EPI_ISL_2418324, EPI_ISL_2418576, EPI_ISL_2418792, EPI_ISL_2418816, EPI_ISL_2418824, EPI_ISL_2418865, EPI_ISL_2418875, EPI_ISL_2418881, EPI_ISL_2418887, EPI_ISL_2418888, EPI_ISL_2418897, EPI_ISL_2418903, EPI_ISL_2418905, EPI_ISL_2418916, EPI_ISL_2418935, EPI_ISL_2418936, EPI_ISL_2418968, EPI_ISL_2419549			
see above	Swedish national genomic surveillance program of SARS-CoV-2	The Public Health Agency of Sweden	Maximilian Riess, Maria Lind Karlberg, Alma Brolund, Swedish national genomic surveillance program of SARS-CoV-2
EPI_ISL_2420697, EPI_ISL_2420700, EPI_ISL_2420701	Viollier AG	Viollier AG	Andrea Patrizia Salzmann, Henriette Kurth, Christiane Beckmann, Maurice Redondo, Olivier Kobel, Christoph Noppen
EPI_ISL_2422284, EPI_ISL_2422287, EPI_ISL_2422291, EPI_ISL_2422310	SUNY UPSTATE MEDICAL UNIVERSITY	Wadsworth Center, New York State Department of Health	Kirsten St. George, Daryl M. Lamson, Alexis Russell, Matthew Shudt, Melissa A Leisner, Jonathan Plitnick, Catharine Prussing, Navjot Singh, John Kelly, Erasmus Schneider, Erica Lasek-Nesselquist
EPI_ISL_2422357	URMC LABS	Wadsworth Center, New York State Department of Health	Kirsten St. George, Daryl M. Lamson, Alexis Russell, Matthew Shudt, Melissa A Leisner, Jonathan Plitnick, Catharine Prussing, Navjot Singh, John Kelly, Erasmus Schneider, Erica Lasek-Nesselquist
EPI_ISL_2424246	Oregon State Public Health Laboratory	Oregon State Public Health Laboratory	Rafia Razzaque, Eugene Yeboah, Vanda Makris, Laura Tsaknaridis, John Fontana and Shane Sevey
EPI_ISL_2425456, EPI_ISL_2425469	Virginia Division of Consolidated Laboratory Services	Virginia Division of Consolidated Laboratory Services	Virginia DCLS
EPI_ISL_2426477, EPI_ISL_2426495, EPI_ISL_2426505, EPI_ISL_2426555	UW Virology Lab	UW Virology Lab	Pavitra Roychoudhury, Hong Xie, Lasata Shrestha, Tien V. Nguyen, Shah Mohamed Bakhash, Michelle Lin, Noah R. Baker, Sean Ellis, Meei-Li Huang, Keith R Jerome, Alexander Greninger
EPI_ISL_2426579, EPI_ISL_2426610	SYNLAB	GIGA Medical Genomics	Keith Durkin, Maria Artesi, Sébastien Bontems, Raphaël Boreux, Bouchra Boujemla, Nathalie Renotte, Cécile Meex, Pierrette Melin, Marie-Pierre Hayette, Vincent Bours
EPI_ISL_2426657, EPI_ISL_2426658	Servicio de Microbiologia Hospital Ramon y Cajal	Servicio de Microbiologia Hospital Ramon y Cajal	Ponce M, Galan JC, Martinez L. Abreu M, y Gonzalez-Alba JM
EPI_ISL_2429049	Clinical Microbiology, Infection Prevention and Control	Section for Molecular Diagnostics	Björn Hallström, Jonas Björkman
EPI_ISL_2431800	Department of Bacteria, Parasites and Fungi, Statens Serum Institut, Copenhagen, Denmark	Statens Serum Institut Bioinformatics and Microbial Genomics	Danish Covid-19 Genome Consortium
EPI_ISL_2432662	UW Virology Lab	UW Virology Lab	Pavitra Roychoudhury, Hong Xie, Lasata Shrestha, Tien V. Nguyen, Shah Mohamed Bakhash, Michelle Lin, Noah R. Baker, Ricardo Perez, Sean Ellis, Nathan Breit, Robert J. Livingston, Meei-Li Huang, Keith R Jerome, Patrick Mathias, Alexander Greninger
EPI_ISL_2433565, EPI_ISL_2433566, EPI_ISL_2433567, EPI_ISL_2433568, EPI_ISL_2433569, EPI_ISL_2433570, EPI_ISL_2433571	Naradhiwas Rajanagarindra Hospital	Division of Genomic Medicine and Innovation support,Department of Medical Sciences, Ministry of Public Health, Thailand	Surakameth Mahasirimongkol,Nuanjun Wichukchinda,Archawin Rojanawiwat,Pilailuk Akkapaiboon Okada ,Waritta Sawaengdee,Penpitcha Thawong,Pundharika Piboonsiri,Jirapha Pakdee,Natthakul Bunneang
EPI_ISL_2434247	Department of Virology	Department of Virology	Massab Umair, Aamer Ikram, Muhammad Salman, Nazish Badar, Zaira Rehman, Muhammad Ammar, Syed Adnan Haider
EPI_ISL_2438544	Lighthouse Lab in Milton Keynes	Wellcome Sanger Institute for the COVID-19 Genomics UK (COG-UK) Consortium	The Lighthouse Lab in Milton Keynes and Alex Alderton, Roberto Amato, Jeffrey Barrett, Sonia Goncalves, Ewan Harrison, David K. Jackson, Ian Johnston, Dominic Kwiatkowski, Cordelia Langford, John Sillitoe on behalf of the Wellcome Sanger Institute COVID-19 Surveillance Team
EPI_ISL_2438793	Dept. of Microbiology and Infection Control, Akershus University Hospital HF	Dept. of Microbiology and Infection Control, Akershus University Hospital HF	Hege Vangstein Aamot, Alexander Hesselberg Løvestad
EPI_ISL_2440109	Helix/Illumina	Centers for Disease Control and Prevention Division of Viral Diseases, Pathogen Discovery	Dakota Howard, Dhwani Batra, Peter W. Cook, Kara Moser, Adrian Paskey, Jason Caravas, Benjamin Rambo-Martin, Shatavia Morrison, Christopher Gulvick, Scott Sammons, Yvette Unoarumhi, Darlene Wagner, Matthew Schmerer, Eileen de Feo, Jan Antico, Christine Tran, Matthew Tolentino, Shannon Wickline, Kim Gietzen, Brad Sickler, Jingtao Liu, Eric Allen, Phil Febbo, Nicole L. Washington, Simon White, Geraint Levan, Kelly Schiabor Barrett, Elizabeth Cirulli, Alexandre Bolze, Ary Ascencio, Charlotte Rivera-Garcia, Ryan Cho, Jason Nguyen, Sherry Wang, Jimmy Ramirez, Tyler Cassens, Efren Sandoval, Magnus Isaksson, William Lee, David Becker, Marc Laurent, James Lu, Clinton R. Paden, Duncan MacCannell
EPI_ISL_2442052	AZDelta	AZ Delta Medical Laboratories in Roeselare, Belgium	Geert Martens, Dieter De Smet, Merijn Vanhee, on behalf of AZ Delta COVID-19 Genomics core (member of Genomic surveillance of SARS-CoV-2 in Belgium network)
EPI_ISL_2443143, EPI_ISL_2443194	Kansas Health and Environmental Lab	Kansas Health and Environmental Lab	Katherine Wiggins, Mike Grose, Jonathan Barnell, Ben Olsen, and Phil Adam
EPI_ISL_2444388	CH TOURCOING	CHU Lille - Laboratoire de Virologie	AIT YAHYA Emilie, ALIDJINOU Enagnon Kazali, BOCKET Laurence, CREPIN Michel, DEMAY Christophe, ENGELMANN Ilka, GEFFROY Sandrine, GUIGON Aurélie, LAMBERT Valérie, LAZREK Mouna, NOBILLIAUX Florian, PREVOST Brigitte, THUILLIER Caroline, TINEZ Claire
EPI_ISL_2447604, EPI_ISL_2447608, EPI_ISL_2447610, EPI_ISL_2447639, EPI_ISL_2447662	SARS-CoV-2 testing team, National Institute of Infectious Diseases	Pathogen Genomics Center, National Institute of Infectious Diseases	Tsuyoshi Sekizuka, Kentaro Itokawa, Rina Tanaka, Masanori Hashino, Nozomu Hanaoka, Masumichi Saito, Naomi Nojiri, Hazuka Y Furihata, Sana Uchikoba, Tsuguto Fujimoto, Makoto Kuroda

EPI_ISL_2448024	Microbiology Department, Laboratori Clínic Metropolitana Nord. Hospital Universitari Germans Trias i Pujol	Can Ruti SARS-CoV-2 Sequencing Hub (HUGTiP/IrsiCaixa/IGTP)	Marc Noguera-Julian, Pilar Armengol, Ignacio Blanco, Antoni E Bordoy, Francesc Catala-Moll, Pere-Joan Cardona, Maria Casadell, Cristina Casa, Gemma Clara, Bonaventura Clotet, Cristina Esteban, Montserrat Gimnez, Mercedes Guerrero, Anna Not, Roger Paredes, Mariona Parera, Vernica Saludes, Alba S nchez, and Elisa Martron behalf of the Can Ruti SARS-CoV-2 Sequencing Hub.
EPI_ISL_2448643	Medilab	CHU Poitiers	Caroline MICHAUD, Maxime PICHON, Manon PRAT, Valentin BON-BARET, Birama N'DIAYE, Magali GARCIA, Agnes BEBY-DEFAUX, Nicolas LEVEQUE
EPI_ISL_2451794, EPI_ISL_2451799	IL Dept. of Public Health Springfield Laboratory	Centers for Disease Control and Prevention Division of Viral Diseases, Pathogen Discovery	Mili Sheth, Sarah Nobles, Jasmine Padilla, Mark Burroughs, Shoshona Le, Katie Dillon, Peter Cook, Clinton R. Paden, Dhwani Batra, Krista Queen, Kristen Knipe, Dakota Howard, Yvette Unoarumhi, Darlene Wagner, Matthew Schmerer, Ben L. Rambo-Martin, Kristine Lacek, Sam Shepard, Alison Laufer Halpin, Dave Wentworth, Vivien Dugan, Suxiang Tong, Justin Lee
EPI_ISL_2455229	New South Wales Health Pathology Royal Prince Alfred Hospital	Microbiology RPAH	Foster, C.; Au, J.; Ruiz Silva, M.; Deveson, I.; Bull, R.; Van Hal, S.; Rawlinson, W.
EPI_ISL_2455266, EPI_ISL_2455275	Johns Hopkins Hospital Department of Pathology	Johns Hopkins Hospital Department of Pathology	C. Paul Morris, Chun Huai Luo, Adannaya Amadi, Matthew Schwartz, Nicholas Gallagher, Heba H. Mostafa
EPI_ISL_2456639	Lighthouse Lab in Milton Keynes	Wellcome Sanger Institute for the COVID-19 Genomics UK (COG-UK) Consortium	The Lighthouse Lab in Milton Keynes and Alex Alderton, Roberto Amato, Jeffrey Barrett, Sonia Goncalves, Ewan Harrison, David K. Jackson, Ian Johnston, Dominic Kwiatkowski, Cordelia Langford, John Sillitoe on behalf of the Wellcome Sanger Institute COVID-19 Surveillance Team
EPI_ISL_2458463	Utah Public Health Laboratory	Utah Public Health Laboratory	Erin L. Young, Kelly F. Oakeson, Tara Gallagher
EPI_ISL_2462870	AZ Sint-Lucas Gent	AZ Sint-Lucas Gent	Jos Van Acker, Charlotte Verfaillie, Elke Vanlaere, Klara De Rauw
EPI_ISL_2462952	Labo Analyses Med	National Reference Center for Viruses of Respiratory Infections, Institut Pasteur, Paris	Marion Barbet, Sylvie Behillil, Méline Bizard, Angela Brisebarre, Camille Capel, Vincent Enouf, Louise Lefrançois, Frédéric Lemoine, Christophe Malabat, Corinne Maufrais, Etienne Simon-Lorière, Maud Vanpeene, Sylvie Van der Werf ,Patricia Stoessel-Thouvenin
EPI_ISL_2462953, EPI_ISL_2462954	Hospital	National Reference Center for Viruses of Respiratory Infections, Institut Pasteur, Paris	Marion Barbet, Sylvie Behillil, Méline Bizard, Angela Brisebarre, Camille Capel, Vincent Enouf, Louise Lefrançois, Frédéric Lemoine, Christophe Malabat, Corinne Maufrais, Etienne Simon-Lorière, Maud Vanpeene, Sylvie Van der Werf ,Pascale Martres
EPI_ISL_2462955	Labo Analyses Med	National Reference Center for Viruses of Respiratory Infections, Institut Pasteur, Paris	Marion Barbet, Sylvie Behillil, Méline Bizard, Angela Brisebarre, Camille Capel, Vincent Enouf, Louise Lefrançois, Frédéric Lemoine, Christophe Malabat, Corinne Maufrais, Etienne Simon-Lorière, Maud Vanpeene, Sylvie Van der Werf ,Vincent Vieillefond
EPI_ISL_2462958	Hospital	National Reference Center for Viruses of Respiratory Infections, Institut Pasteur, Paris	Marion Barbet, Sylvie Behillil, Méline Bizard, Angela Brisebarre, Camille Capel, Vincent Enouf, Louise Lefrançois, Frédéric Lemoine, Christophe Malabat, Corinne Maufrais, Etienne Simon-Lorière, Maud Vanpeene, Sylvie Van der Werf ,AgnèS Scanvic
EPI_ISL_2462960, EPI_ISL_2462962	Hospital	National Reference Center for Viruses of Respiratory Infections, Institut Pasteur, Paris	Marion Barbet, Sylvie Behillil, Méline Bizard, Angela Brisebarre, Camille Capel, Vincent Enouf, Louise Lefrançois, Frédéric Lemoine, Christophe Malabat, Corinne Maufrais, Etienne Simon-Lorière, Maud Vanpeene, Sylvie Van der Werf ,Axelle Paquin
EPI_ISL_2462963	Hospital	National Reference Center for Viruses of Respiratory Infections, Institut Pasteur, Paris	Marion Barbet, Sylvie Behillil, Méline Bizard, Angela Brisebarre, Camille Capel, Vincent Enouf, Louise Lefrançois, Frédéric Lemoine, Christophe Malabat, Corinne Maufrais, Etienne Simon-Lorière, Maud Vanpeene, Sylvie Van der Werf ,CéLine Ramanantsoa
EPI_ISL_2462988	ZOL	Jessa	Berden et al. on behalf of the Jessa_cmdLab
EPI_ISL_2463242	Quest Diagnostics Incorporated	Centers for Disease Control and Prevention Division of Viral Diseases, Pathogen Discovery	Dakota Howard, Dhwani Batra, Peter W. Cook, Kara Moser, Adrian Paskey, Jason Caravas, Benjamin Rambo-Martin, Shatavia Morrison, Christopher Gulvick, Scott Sammons, Yvette Unoarumhi, Darlene Wagner, Matthew Schmerer, S. H. Rosenthal, A. Gerasimova, R. M. Kagan, B. Anderson, M. Hua, Y. Liu, L.E. Bernstein, K.E. Livingston, A. Perez, I. A. Shlyakhter, R. V. Rolando, R. Owen, P. Tanpaiboon, F. Lacbawan, Clinton R. Paden, Duncan MacCannell
EPI_ISL_2463305, EPI_ISL_2463306, EPI_ISL_2463307, EPI_ISL_2463310	LANCET LABORATORY	National Institute for Communicable Diseases of the National Health Laboratory Service	Sisonke Team, Amoako DG, Everatt J, Viana R, Glass A, Bhiman JN
EPI_ISL_2464082	Hospital	National Reference Center for Viruses of Respiratory Infections, Institut Pasteur, Paris	Marion Barbet, Sylvie Behillil, Méline Bizard, Angela Brisebarre, Camille Capel, Vincent Enouf, Louise Lefrançois, Frédéric Lemoine, Christophe Malabat, Corinne Maufrais, Etienne Simon-Lorière, Maud Vanpeene, Sylvie Van der Werf ,Marianne Burgard
EPI_ISL_2464088, EPI_ISL_2464089	Hospital	National Reference Center for Viruses of Respiratory Infections, Institut Pasteur, Paris	Marion Barbet, Sylvie Behillil, Méline Bizard, Angela Brisebarre, Camille Capel, Vincent Enouf, Louise Lefrançois, Frédéric Lemoine, Christophe Malabat, Corinne Maufrais, Etienne Simon-Lorière, Maud Vanpeene, Sylvie Van der Werf ,AgnèS Scanvic
EPI_ISL_2464098	Armies	National Reference Center for Viruses of Respiratory Infections, Institut Pasteur, Paris	Marion Barbet, Sylvie Behillil, Méline Bizard, Angela Brisebarre, Camille Capel, Vincent Enouf, Louise Lefrançois, Frédéric Lemoine, Christophe Malabat, Corinne Maufrais, Etienne Simon-Lorière, Maud Vanpeene, Sylvie Van der Werf ,Marine Desroches
EPI_ISL_2464156	Labo Analyses Med	National Reference Center for Viruses of Respiratory Infections, Institut Pasteur, Paris	Marion Barbet, Sylvie Behillil, Méline Bizard, Angela Brisebarre, Camille Capel, Vincent Enouf, Louise Lefrançois, Frédéric Lemoine, Christophe Malabat, Corinne Maufrais, Emmanuelle Permal, Etienne Simon-Lorière, Maud Vanpeene, Sylvie Van der Werf ,Arditi Cocco
EPI_ISL_2464168, EPI_ISL_2464177, EPI_ISL_2464183	Labo Analyses Med	National Reference Center for Viruses of Respiratory Infections, Institut Pasteur, Paris	Marion Barbet, Sylvie Behillil, Méline Bizard, Angela Brisebarre, Camille Capel, Vincent Enouf, Louise Lefrançois, Frédéric Lemoine, Christophe Malabat, Corinne Maufrais, Etienne Simon-Lorière, Maud Vanpeene, Sylvie Van der Werf ,Arditi Cocco
EPI_ISL_2464242	Hospital	National Reference Center for Viruses of Respiratory Infections, Institut Pasteur, Paris	Marion Barbet, Sylvie Behillil, Méline Bizard, Angela Brisebarre, Camille Capel, Vincent Enouf, Louise Lefrançois, Frédéric Lemoine, Christophe Malabat, Corinne Maufrais, Etienne Simon-Lorière, Maud Vanpeene, Sylvie Van der Werf ,Marianne Burgard

EPI_ISL_2464248	Hospital	National Reference Center for Viruses of Respiratory Infections, Institut Pasteur, Paris	Marion Barbet, Sylvie Behillil, Méline Bizard, Angela Brisebarre, Camille Capel, Vincent Enouf, Louise Lefrançois, Frédéric Lemoine, Christophe Malabat, Corinne Maufrais, Etienne Simon-Lorière, Maud Vanpeene, Sylvie Van der Werf ,AgnèS Scanvic
EPI_ISL_2464422, EPI_ISL_2464423, EPI_ISL_2464424	GH de l'Est Francilien	Department of Virology, Henri Mondor University Hospital, Assistance Publique Hôpitaux de Paris, Université Paris-Est Créteil, INSERM U955	Christophe Rodriguez, Slim Fourati, Vanessa Demontant, Guillaume Gricourt, Melissa N'Debi, Alexandre Soulier, Elisabeth Trawinski, Jean-Michel Pawlotsky
EPI_ISL_2464442	Hôpital Bicêtre	Department of Virology, Henri Mondor University Hospital, Assistance Publique Hôpitaux de Paris, Université Paris-Est Créteil, INSERM U955	Christophe Rodriguez, Slim Fourati, Vanessa Demontant, Guillaume Gricourt, Melissa N'Debi, Alexandre Soulier, Elisabeth Trawinski, Jean-Michel Pawlotsky
EPI_ISL_2464473, EPI_ISL_2464479	Biogroup Bio Lam-LCD Saint-Denis	Department of Virology, Henri Mondor University Hospital, Assistance Publique Hôpitaux de Paris, Université Paris-Est Créteil, INSERM U955	Christophe Rodriguez, Slim Fourati, Vanessa Demontant, Guillaume Gricourt, Melissa N'Debi, Alexandre Soulier, Elisabeth Trawinski, Jean-Michel Pawlotsky
EPI_ISL_2464484, EPI_ISL_2464493, EPI_ISL_2464495	Hôpital Avicenne	Department of Virology, Henri Mondor University Hospital, Assistance Publique Hôpitaux de Paris, Université Paris-Est Créteil, INSERM U955	Christophe Rodriguez, Slim Fourati, Vanessa Demontant, Guillaume Gricourt, Melissa N'Debi, Alexandre Soulier, Elisabeth Trawinski, Jean-Michel Pawlotsky
EPI_ISL_2464502, EPI_ISL_2464512, EPI_ISL_2464513	GH A.CHENEVIER-H.MONDOR	Department of Virology, Henri Mondor University Hospital, Assistance Publique Hôpitaux de Paris, Université Paris-Est Créteil, INSERM U955	Christophe Rodriguez, Slim Fourati, Vanessa Demontant, Guillaume Gricourt, Melissa N'Debi, Alexandre Soulier, Elisabeth Trawinski, Jean-Michel Pawlotsky
EPI_ISL_2464521	HOPITAL EMILE ROUX	Department of Virology, Henri Mondor University Hospital, Assistance Publique Hôpitaux de Paris, Université Paris-Est Créteil, INSERM U955	Christophe Rodriguez, Slim Fourati, Vanessa Demontant, Guillaume Gricourt, Melissa N'Debi, Alexandre Soulier, Elisabeth Trawinski, Jean-Michel Pawlotsky
EPI_ISL_2464548, EPI_ISL_2464549	Hôpital Cochin	Department of Virology, Henri Mondor University Hospital, Assistance Publique Hôpitaux de Paris, Université Paris-Est Créteil, INSERM U955	Christophe Rodriguez, Slim Fourati, Vanessa Demontant, Guillaume Gricourt, Melissa N'Debi, Alexandre Soulier, Elisabeth Trawinski, Jean-Michel Pawlotsky
EPI_ISL_2464780	Laboratoire CBM 25 TERRE ROUGE	Department of Virology, Henri Mondor University Hospital, Assistance Publique Hôpitaux de Paris, Université Paris-Est Créteil, INSERM U955	Christophe Rodriguez, Slim Fourati, Vanessa Demontant, Guillaume Gricourt, Melissa N'Debi, Alexandre Soulier, Elisabeth Trawinski, Jean-Michel Pawlotsky
EPI_ISL_2464788	Besançon Laboratoire CBM25 Terre Rouge	Department of Virology, Henri Mondor University Hospital, Assistance Publique Hôpitaux de Paris, Université Paris-Est Créteil, INSERM U955	Christophe Rodriguez, Slim Fourati, Vanessa Demontant, Guillaume Gricourt, Melissa N'Debi, Alexandre Soulier, Elisabeth Trawinski, Jean-Michel Pawlotsky
EPI_ISL_2464887	CH.INTERCOMMUNAL DE CRETEIL	Department of Virology, Henri Mondor University Hospital, Assistance Publique Hôpitaux de Paris, Université Paris-Est Créteil, INSERM U955	Christophe Rodriguez, Slim Fourati, Vanessa Demontant, Guillaume Gricourt, Melissa N'Debi, Alexandre Soulier, Elisabeth Trawinski, Jean-Michel Pawlotsky
EPI_ISL_2464936	CH. ROBERT BALLANGER	Department of Virology, Henri Mondor University Hospital, Assistance Publique Hôpitaux de Paris, Université Paris-Est Créteil, INSERM U955	Christophe Rodriguez, Slim Fourati, Vanessa Demontant, Guillaume Gricourt, Melissa N'Debi, Alexandre Soulier, Elisabeth Trawinski, Jean-Michel Pawlotsky
EPI_ISL_2464980	Biogroup Bio Lam-LCD Saint-Denis	Department of Virology, Henri Mondor University Hospital, Assistance	Christophe Rodriguez, Slim Fourati, Vanessa Demontant, Guillaume Gricourt, Melissa N'Debi, Alexandre Soulier, Elisabeth Trawinski, Jean-Michel Pawlotsky

		Publique Hôpitaux de Paris, Université Paris-Est Créteil, INSERM U955	
EPI_ISL_2465017	SELAS CERBALLIANCE Normandie ouest	Department of Virology, Henri Mondor University Hospital, Assistance Publique Hôpitaux de Paris, Université Paris-Est Créteil, INSERM U955	Christophe Rodriguez, Slim Fourati, Vanessa Demontant, Guillaume Gricourt, Melissa N'Debi, Alexandre Soulier, Elisabeth Trawinski, Jean-Michel Pawlotsky
EPI_ISL_2466325	National Platform bis UMONS/Jolimont	National Platform bis UMONS/Jolimont	François Dufrasne, Guillaume Bayon-Vicente, Florian Juszczak, Gautier Detry, Ruddy Wattiez
EPI_ISL_2466510	National Institute of Public Health	State Veterinary Institute Prague	Nagy,A;Jirincova,H;Suri,T;Trnka,D;Vecerova,J
EPI_ISL_2466736, EPI_ISL_2466737, EPI_ISL_2466738	Nebraska Public Health Laboratory	NPHL COVID-19 Response Team	NPHL COVID-19 Response Team
EPI_ISL_2467794	University of Wisconsin-Madison AIDS Vaccine Research Laboratories	University of Wisconsin-Madison AIDS Vaccine Research Laboratories	Gage Moreno, Katarina Braun, et al. AIDS Vaccine Research Laboratories
EPI_ISL_2467803	Alaska State Virology Laboratory	Alaska State Virology Laboratory	Stephanie DeRonde, Elva House, Jacob Zidek, Lisa Smith, Ph.D., Jack Chen, Ph.D.
EPI_ISL_2467881	NC State Laboratory of Public Health	Genomics and Discovery, Respiratory Viruses Branch, Division of Viral Diseases, Centers for Disease Control and Prevention	Ying Tao, Yan Li, Jing Zhang, Anna Kelleher, Brian Lynch, Krista Queen, Anna Uehara, Peter Cook, Han Jia Justin Ng, Rachel Marine, Clinton R. Paden, Dhwani Batra, Haibin Wang, Tara Coalter, Jasmine Padilla, Morgan Davis, Mili Sheth, Sarah Nobles, Mark Burroughs, Justin Lee, Adam Retchless, Suxiang Tong
EPI_ISL_2469571	SYNLAB MVZ Leinfelden-Echterdingen	Robert Koch Institute	unknown
EPI_ISL_2470239	LabKom - Labor Hannover MVZ GmbH	Robert Koch Institute	unknown
EPI_ISL_2470249, EPI_ISL_2470309	Sonic - Bioscientia - MVZ Labor Saar GmbH	Robert Koch Institute	unknown
EPI_ISL_2471027	Labormedizin Darmstadt	Robert Koch Institute	unknown
EPI_ISL_2471047, EPI_ISL_2471057	Labor Dr. Heidrich & Kollegen MVZ GmbH Hamburg	Robert Koch Institute	unknown
EPI_ISL_2471241	CENTOGENE Frankfurt Laboratory: Niederlassung Industriepark Höchst	Robert Koch Institute	unknown
EPI_ISL_2471529	MVZ Medizinisches Labor Hannover GmbH	Robert Koch Institute	unknown
EPI_ISL_2471644, EPI_ISL_2471651	Eurofins LifeCodexx GmbH	Robert Koch Institute	unknown
EPI_ISL_2471910	MVZ Labor Dr. Limbach & Kollegen GbR	Robert Koch Institute	unknown
EPI_ISL_2472394	MDI Limbach Berlin GmbH; MVZ Labor Berlin	Robert Koch Institute	unknown
EPI_ISL_2472470, EPI_ISL_2472488, EPI_ISL_2472491, EPI_ISL_2472494, EPI_ISL_2472499	MVZ Labor Dr. Limbach & Kollegen GbR	Robert Koch Institute	unknown
EPI_ISL_2472547	Labor Dr. Spranger	Robert Koch Institute	unknown
EPI_ISL_2472859	Labor ZOTZ|KLIMAS; MVZ Düsseldorf-Centrum	Robert Koch Institute	unknown
EPI_ISL_2473327, EPI_ISL_2473341, EPI_ISL_2473362, EPI_ISL_2473477, EPI_ISL_2473531	State Testing Facility	Altius Institute for Biomedical Research	Daniel Bates, Rebecca Bruders, Michael Buckley, Mark Frerker, Amanda Gale, Clem Green, Muhammad Halimun, Kneshay Harper, Matt Hartman, Alex Isner, Audra Johnson, Jessica Kunder, Lauren Mitchell, Jemma Nelson, Alex Nguyen, Sofia Olsson, Sadie Patraw, Tobias Ragoczy, Joshua Richards, Jean Robinston, Jacob Rodriguez, John Stamatoyannopoulos, Eric Thorland, Julia Wald
EPI_ISL_2476301, EPI_ISL_2476305, EPI_ISL_2476306	Dutch COVID-19 response team	National Institute for Public Health and the Environment (RIVM)	Adam Meijer, Harry Vennema, Dirk Eggink, Jeroen Cremer, Sharon van den Brink, Bas van der Veer, AnneMarie van den Brandt, Lisa Wijsman, Kim Freriks, Ryanne Jaarsma, Euníce Then, Lynn Aarts, Sanne Bos, Melissa van Tuil, Linda van de Nes, Florian Zwagemaker, Dennis Schmitz, Annelies Kroneman, Karim Hajji, Chantal Reusken, on behalf of the national COVID-19 response team
EPI_ISL_2480245, EPI_ISL_2480912, EPI_ISL_2481076, EPI_ISL_2481340, EPI_ISL_2481776, EPI_ISL_2481896, EPI_ISL_2482231	Laboratory Corporation of America	Centers for Disease Control and Prevention Division of Viral Diseases, Pathogen Discovery	Dakota Howard, Dhwani Batra, Peter W. Cook, Kara Moser, Adrian Paskey, Jason Caravas, Benjamin Rambo-Martin, Shatavia Morrison, Christopher Gulvick, Scott Sammons, Yvette Unoarumhi, Darlene Wagner, Matthew Schmerer, Minoo Agarwal, Eyad Almasri, Debbie Boles, Ayla Burns, Nuthawin Charoensri, Oren Cohen, Susan Countryman, Mary Ann Cristobal, Bobbi Croy, Suzanne Dale, Hrushikesh Deshmukh, Amanda Douglas, Vincent Drouillon, Marcia Eisenberg, Howard Engler, Rama Ghatti, Prashant Gupta, Susan Hicks, Jake Humphrey, Lax Iyer, Lisa Pfefferle, Manoj Jain, Matthew Robinson, Mohan Kolli, Brian Krueger, Tim Kuphal, Stanley Letovsky, Michael Levandoski, Craig Lukasik, Jonathan Meltzer, Brian Norvell, Mindy Nye, Scott Parker, Christos Petropoulos, John Pruitt, Steven Ragan, Scott Ryan, Mike Sapeta, Jana Schroth, Suresh Babu Selvaraju,

			Goran Stevovic, Amanda Suchanek, Andrea Throop, Lyndon Tilson, Thomas Urban, Joe Voshell, Kimberly Wagner, Jonathan Williams, Mary Williamson, Qian Zeng, Tricia Zwiefelhofer, Clinton R. Paden, Duncan MacCannell
EPI_ISL_2482521	LABORATOIRE DE BIOLOGIE MEDICALE	CNR Virus des Infections Respiratoires - France SUD	Antonin Bal, Gregory Destras, Gwendolyne Burfin, Hadrien Regue, Quentin Semanas, Martine Valette, Bruno Lina, Laurence Josset
EPI_ISL_2482590	LABORATOIRE UNILIANS DECINES	CNR Virus des Infections Respiratoires - France SUD	Antonin Bal, Gregory Destras, Gwendolyne Burfin, Hadrien Regue, Quentin Semanas, Martine Valette, Bruno Lina, Laurence Josset
EPI_ISL_2482608	MIRIALIS CLUSES BECHET	CNR Virus des Infections Respiratoires - France SUD	Antonin Bal, Gregory Destras, Gwendolyne Burfin, Hadrien Regue, Quentin Semanas, Martine Valette, Bruno Lina, Laurence Josset
EPI_ISL_2482633	LABORATOIRE UNILIANS DECINES	CNR Virus des Infections Respiratoires - France SUD	Antonin Bal, Gregory Destras, Gwendolyne Burfin, Hadrien Regue, Quentin Semanas, Martine Valette, Bruno Lina, Laurence Josset
EPI_ISL_2482669	LABORATOIRE CREAVALLEE	CNR Virus des Infections Respiratoires - France SUD	Antonin Bal, Gregory Destras, Gwendolyne Burfin, Hadrien Regue, Quentin Semanas, Martine Valette, Bruno Lina, Laurence Josset
EPI_ISL_2482787	LABORATOIRE CERBALLIANCE PLT VILLON	CNR Virus des Infections Respiratoires - France SUD	Antonin Bal, Gregory Destras, Gwendolyne Burfin, Hadrien Regue, Quentin Semanas, Martine Valette, Bruno Lina, Laurence Josset
EPI_ISL_2482799	HOPITAL SAINT ANDRE	CNR Virus des Infections Respiratoires - France SUD	Antonin Bal, Gregory Destras, Gwendolyne Burfin, Hadrien Regue, Quentin Semanas, Martine Valette, Bruno Lina, Laurence Josset
EPI_ISL_2482828	C.H.R.U. MONTPIED	CNR Virus des Infections Respiratoires - France SUD	Antonin Bal, Gregory Destras, Gwendolyne Burfin, Hadrien Regue, Quentin Semanas, Martine Valette, Bruno Lina, Laurence Josset
EPI_ISL_2482829	HOPITAL ALBI	CNR Virus des Infections Respiratoires - France SUD	Antonin Bal, Gregory Destras, Gwendolyne Burfin, Hadrien Regue, Quentin Semanas, Martine Valette, Bruno Lina, Laurence Josset
EPI_ISL_2482837, EPI_ISL_2482841, EPI_ISL_2482845	LBM UNIBIO VALENTIN	CNR Virus des Infections Respiratoires - France SUD	Antonin Bal, Gregory Destras, Gwendolyne Burfin, Hadrien Regue, Quentin Semanas, Martine Valette, Bruno Lina, Laurence Josset
EPI_ISL_2482918, EPI_ISL_2482920, EPI_ISL_2482921	CERBAILLIANCE LA REUNION	CNR Virus des Infections Respiratoires - France SUD	Antonin Bal, Gregory Destras, Gwendolyne Burfin, Hadrien Regue, Quentin Semanas, Martine Valette, Bruno Lina, Laurence Josset
EPI_ISL_2482922	CERBALLIANCE REUNION	CNR Virus des Infections Respiratoires - France SUD	Antonin Bal, Gregory Destras, Gwendolyne Burfin, Hadrien Regue, Quentin Semanas, Martine Valette, Bruno Lina, Laurence Josset
EPI_ISL_2482927, EPI_ISL_2482930	CERBAILLIANCE LA REUNION	CNR Virus des Infections Respiratoires - France SUD	Antonin Bal, Gregory Destras, Gwendolyne Burfin, Hadrien Regue, Quentin Semanas, Martine Valette, Bruno Lina, Laurence Josset
EPI_ISL_2482941	ORIAPOLE - BACTERIOLOGIE	CNR Virus des Infections Respiratoires - France SUD	Antonin Bal, Gregory Destras, Gwendolyne Burfin, Hadrien Regue, Quentin Semanas, Martine Valette, Bruno Lina, Laurence Josset
EPI_ISL_2483090	Furst Medical Laboratory	Norwegian Institute of Public Health, Department of Virology	Kathrine Stene-Johansen, Kamilla Heddeland Instefjord, Hilde Elshaug, Garcia Llorente Ignacio, Jon Bråte, Engebretsen Serina Beate,Pedersen Benedikte Nevjen, Line Victoria Moen, Debech Nadia, Atiya R Ali,Marie Paulsen Madsen, Rasmus Riis Kopperud, Hilde Vollan, Karoline Bragstad, Olav Hungnes
EPI_ISL_2483103	Ostfold Hospital Trust - Kalnes, Centre for Laboratory Medicine, Section for gene technology and infection serology	Norwegian Institute of Public Health, Department of Virology	Kathrine Stene-Johansen, Kamilla Heddeland Instefjord, Hilde Elshaug, Garcia Llorente Ignacio, Jon Bråte, Engebretsen Serina Beate,Pedersen Benedikte Nevjen, Line Victoria Moen, Debech Nadia, Atiya R Ali,Marie Paulsen Madsen, Rasmus Riis Kopperud, Hilde Vollan, Karoline Bragstad, Olav Hungnes
EPI_ISL_2483247	Platform BIS UZA/UAntwerpen	Labo Klinische Biologie, UZA	Jasmine Coppens, Marie Le Mercier, Basil Britto Xavier, Christine Lammens, Veerle Matheeussen, Herman Goossens
EPI_ISL_2483708	National Platform bis UMONS/Jolimont	National Platform bis UMONS/Jolimont	François Dufrasne, Guillaume Bayon-Vicente, Florian Juszczak, Gautier Detry, Ruddy Wattiez
EPI_ISL_2483754	Ospedale Sandro Pertini ASL Roma 2	INMI Lazzaro Spallanzani IRCCS	G Bonfiglio, O Butera, F Messina, CEM Gruber, G Orlandi, E Sperandio, MC Cava, R Longo, B Bartolini
EPI_ISL_2484019	Hospital General Universitario Gregorio Marañón	Hospital General Universitario Gregorio Marañón	Sergio Buenestado Serrano, Pedro Sola Campoy, Laura Pérez-Lago, Cristina Rodriguez-Grande, Marta Herranz Martin, Victor Manuel de la Cueva, Julia Suárez, Pilar Catalán, Patricia Muñoz, Darío García de Viedma
EPI_ISL_2484046	Arizona State University	Arizona State University	Peter T. Skidmore, LaRinda A. Holland, Matthew F. Smith, Rabia Maqsood, Nicholas J. Mellor, Joy M. Blain, Valerie Harris, Joshua LaBaer, Vel Murugan, Efrem S. Lim
EPI_ISL_2484882	Southern Nevada Public Health Laboratory	Southern Nevada Public Health Laboratory	Michael Picker
EPI_ISL_2487022	Lighthouse Lab in Milton Keynes	Wellcome Sanger Institute for the COVID-19 Genomics UK (COG-UK) Consortium	The Lighthouse Lab in Milton Keynes and Alex Alderton, Roberto Amato, Jeffrey Barrett, Sonia Goncalves, Ewan Harrison, David K. Jackson, Ian Johnston, Dominic Kwiatkowski, Cordelia Langford, John Sillitoe on behalf of the Wellcome Sanger Institute COVID-19 Surveillance Team
EPI_ISL_2491660	Centre Hospitalier Universitaire Clermont-Ferrand	CHU Clermont-Ferrand, service de virologie	Bisseux Maxime, Mirand Audrey, Combes Patricia, Henquell Cécile
EPI_ISL_2491664	Centre hospitalier D'AURILLAC	CHU Clermont-Ferrand, service de virologie	Bisseux Maxime, Mirand Audrey, Combes Patricia, Henquell Cécile
EPI_ISL_2493041	AMPATH	CERI, Centre for Epidemic Response and Innvoation, Stellenbosch University and KRISP,	Giandhari J, Pillay S, Naidoo Y, Ramphal U, Lessells R, Mdlalose K, York D, Khan S, Emmanuel SJ, Tegally H, Wilkinson E, de Oliveira T

		KZN Research Innovation and Sequencing Platform, UKZN.	
EPI_ISL_2493147	Hospital Universitari Arnau de Vilanova	Hospital Universitari Vall d'Hebron - Vall d'Hebron Institut de Recerca	Cristina Andrés, Maria Piñana, Alejandra González-Sánchez, Damir Garcia-Cehic, Ariadna Rando, Juliana Esperalba, Maria Gema Codina, Carla Castillo, Maria Carmen Martin, Tomàs Pumarola, Josep Quer, Andrés Antón
EPI_ISL_2495603	MB-Cadham Provincial laboratory	National Microbiology Laboratory (NML)	Anna Majer, Shari Tyson, Grace Seo, Philip Mabon, Elsie Grudeski, Rhiannon Huzarewich, Russell Mandes, Anneliese Landgraff, Jennifer Tanner, Natalie Knox, Morag Graham, Gary Van Domselaar, Paul Van Caeseele, Jared Bullard, David Alexander, Kerry Dust, Nathalie Bastien, Yan Li, Timothy Booth, Darian Hole, Madison Chapel, Kirsten Biggar, CanCOGeN's metadata curation team, Public Health Agency of Canada CanCOGeN team
EPI_ISL_2497353, EPI_ISL_2497382, EPI_ISL_2497422	UW Virology Lab	UW Virology Lab	Pavitra Roychoudhury, Hong Xie, Lasata Shrestha, Tien V. Nguyen, Shah Mohamed Bakhash, Michelle Lin, Noah R. Baker, Ricardo Perez, Sean Ellis, Nathan Breit, Robert J. Livingston, Meei-Li Huang, Keith R Jerome, Patrick Mathias, Alexander Greninger
EPI_ISL_2499991	University of Liège COVID-19 testing center	GIGA Medical Genomics	Keith Durkin, Maria Artesi, Bouchra Boujemla, Nathalie Renotte, Cécile Meex, Sébastien Bontems, Fabrice Bureau, Laurent Gillet, Wouter Coppieters, Marie-Pierre Hayette, Vincent Bours
EPI_ISL_2500498	CHUV	Laboratory of genomics and metagenomics	Trestan Pillonel, Damien Jacot, Sébastien Aeby, Gilbert Greub, Claire Bertelli
EPI_ISL_2501036	Virology Unit, Institut Pasteur du Cambodge	Virology Unit, Institut Pasteur du Cambodge	Cecile Troupin, Leakhena Pum, Jurre Y Siegers, Ly Sovann, Kraing Sidonn, Yi Sengdoeurn, Chin Savuth, Chau Darapheak, Veasna Duong, Erik A Karlsson
EPI_ISL_2501090	AMPATH	KRISP, KZN Research Innovation and Sequencing Platform	Sisonke Team, Giandhari J, Pillay S, Naidoo Y, Ramphal U, Lessells R, Mdlalose K, York D, Khan S, Emmanuel SJ, Tegally H, Wilkinson E, de Oliveira T
EPI_ISL_2501092, EPI_ISL_2501094, EPI_ISL_2501095	Netcare St Augustines Hospital	KRISP, KZN Research Innovation and Sequencing Platform	Sisonke Team, Giandhari J, Pillay S, Naidoo Y, Ramphal U, Lessells R, Mdlalose K, York D, Khan S, Emmanuel SJ, Tegally H, Wilkinson E, de Oliveira T
EPI_ISL_2501301	CH LENS	CHU Lille - Laboratoire de Virologie	AIT YAHYA Emilie, ALIDJINOU Enagnon Kazali, BOCKET Laurence, CREPIN Michel, DEMAY Christophe, ENGELMANN Ilka, GEFFROY Sandrine, GUIGON Aurélie, LAMBERT Valérie, LAZREK Mouna, NOBILLIAUX Florian, PREVOST Brigitte, THUILLIER Caroline, TINEZ Claire ,TCHANTCHOU NJOSSE YANICK
EPI_ISL_2501719	Yale Clinical Virology Lab	Grubaugh Lab - Yale School of Public Health	Joseph Fauver, Mallery Breban, Isabel Ott, Tara Alpert, Mary Petrone, Anderson Brito, Chantal Vogels, Annie Watkins, Chaney Kalinich, Jessica Rothman, Marie L. Landry, Nathan Grubaugh
EPI_ISL_2501929	Missouri State Public Health Laboratory	Missouri State Public Health Laboratory	Matthew Sinn, Joshua Barry, Ashley New
EPI_ISL_2504068, EPI_ISL_2504071, EPI_ISL_2504072, EPI_ISL_2504073, EPI_ISL_2504088	Botswana Harvard HIV Reference Laboratory	Botswana Harvard HIV Reference Laboratory	Sikhulile Moyo, Dorcas Maruapula, Wonderful T. Choga, Thongbotho Mphoyakgosi, Boitumelo Zuze, Botshelo Radibe, Legodile Kooepile, Ontlametse T. Bareng, Keoratile Ntshambiwa, Modisa Motswaledi, Madisa Mine, Joseph Makhema, Roger Shapiro, Shahin Lockman, Mosepele Mosepele, Simani Gaseitsiwe
EPI_ISL_2504114	Palapye Primary Hospital Laboratory	Botswana Harvard HIV Reference Laboratory	Sikhulile Moyo, Dorcas Maruapula, Wonderful T. Choga, Thongbotho Mphoyakgosi, Boitumelo Zuze, Botshelo Radibe, Legodile Kooepile, Ontlametse T. Bareng, Letsibogo Gaoraelwe, Thela Tefelo, Keoratile Ntshambiwa, Modisa Motswaledi, Madisa Mine, Joseph Makhema, Roger Shapiro, Shahin Lockman, Mosepele Mosepele, Simani Gaseitsiwe
EPI_ISL_2508402, EPI_ISL_2508446, EPI_ISL_2508463	Microbiology Division, SC DHEC	Microbiology Division, SC DHEC	Freeman,J., Flores,H.
EPI_ISL_2508715, EPI_ISL_2508716, EPI_ISL_2508723, EPI_ISL_2508724, EPI_ISL_2508725, EPI_ISL_2508801, EPI_ISL_2508802, EPI_ISL_2508803, EPI_ISL_2508804, EPI_ISL_2508888, EPI_ISL_2509024, EPI_ISL_2509025, EPI_ISL_2509026, EPI_ISL_2509027, EPI_ISL_2509028			
see above	National Public Health Laboratory, National Centre for Infectious Diseases	National Public Health Laboratory, National Centre for Infectious Diseases	Tze Minn Mak, Zhenyang Zhou, Royce Ang, Lin Cui, Raymond Tzer Pin Lin
EPI_ISL_2509334	Palapye Primary Hospital Laboratory	Botswana Harvard HIV Reference Laboratory	Sikhulile Moyo, Dorcas Maruapula, Wonderful T. Choga, Thongbotho Mphoyakgosi, Boitumelo Zuze, Botshelo Radibe, Legodile Kooepile, Ontlametse T. Bareng, Letsibogo Gaoraelwe, Thela Tefelo, Keoratile Ntshambiwa, Modisa Motswaledi, Madisa Mine, Joseph Makhema, Roger Shapiro, Shahin Lockman, Mosepele Mosepele, Simani Gaseitsiwe
EPI_ISL_2510596	Servicio de Microbiología Clínica (Complejo Hospitalario de Navarra, Pamplona)	Centro de Secuenciación NASERTIC	Carmen Ezpeleta Baquedano, Ana Navascués, Ana Miqueleiz
EPI_ISL_2516556	Hospital Universitari Bellvitge	Microbiology Department	Sara Marti, Aida Gonzalez-Diaz, Laura Calatayud, Jordi Niubó, Miguel Fernandez-Huerta, Carmen Ardanuy, Jordi Camara, M Angeles Domínguez
EPI_ISL_2516672, EPI_ISL_2516682, EPI_ISL_2516690	Servicio de Microbiología Clínica (Complejo Hospitalario de Navarra, Pamplona)	Centro de Secuenciación NASERTIC	Carmen Ezpeleta Baquedano, Ana Navascués, Ana Miqueleiz
EPI_ISL_2521974	Plateforme de testing Namuroise	Plateforme de testing Namuroise	Nicolas Gilliard; Demars Aurore ; Maschietto Céline ; Nobis Chloé ; Otto Gaetan ; Denis Olivier ; Degosserie Jonathan ; Mullier François
EPI_ISL_2521995, EPI_ISL_2521996	Nucleic Acid Testing, National Reference Laboratory	GIGA Medical Genomics	Yvan Butera, Keith Durkin, Maria Artesi, Bouchra Boujemla, Robert Rutayisire, Patrick Tuyisenge, Esperence Umumararungu, Sébastien Bontems, Marie-Pierre Hayette, Nathalie Renotte, Swaibu Gatare, Jacob Souopgui, Sabin Nsanzimana, Vincent Bours, Léon Mutesa
EPI_ISL_2523862	HELIX LLC	WHO National Influenza Centre Russian Federation	Andrey Komissarov, Artem Fadeev, Kseniya Komissarova, Oula Mansour, Kirill Varchenko, Mikhail Bakaev, Tamila Musaeva, Maria Timofeeva, Veronika Eder, Maria Pisareva, Nikita Yolshin, Daria Danilenko, Ksenia Safina, Elena Nabieva,

			Georgii Bazykin, Dmitry Lioznov
EPI_ISL_2524774	Hospital Universitari Arnau de Vilanova	Hospital Universitari Vall d'Hebron - Vall d'Hebron Institut de Recerca	Cristina Andrés, Maria Piñana, Alejandra González-Sánchez, Damir Garcia-Cehic, Ariadna Rando, Juliana Esperalba, Maria Gema Codina, Carla Castillo, Maria Carmen Martin, Tomàs Pumarola, Josep Quer, Andrés Antón
EPI_ISL_2531827	Hospital	National Reference Center for Viruses of Respiratory Infections, Institut Pasteur, Paris	Marion Barbet, Sylvie Behillil, Méline Bizard, Angela Brisebarre, Camille Capel, Vincent Enouf, Louise Lefrançois, Frédéric Lemoine, Christophe Malabat, Corinne Maufrais, Etienne Simon-Lorière, Maud Vanpeene, Sylvie Van der Werf ,Pascale Martres
EPI_ISL_2531877	Hospital	National Reference Center for Viruses of Respiratory Infections, Institut Pasteur, Paris	Marion Barbet, Sylvie Behillil, Méline Bizard, Angela Brisebarre, Camille Capel, Vincent Enouf, Louise Lefrançois, Frédéric Lemoine, Christophe Malabat, Corinne Maufrais, Etienne Simon-Lorière, Maud Vanpeene, Sylvie Van der Werf ,Marianne Burgard
EPI_ISL_2531934	RIIP	National Reference Center for Viruses of Respiratory Infections, Institut Pasteur, Paris	Marion Barbet, Sylvie Behillil, Méline Bizard, Angela Brisebarre, Camille Capel, Vincent Enouf, Louise Lefrançois, Frédéric Lemoine, Christophe Malabat, Corinne Maufrais, Etienne Simon-Lorière, Maud Vanpeene, Sylvie Van der Werf ,StéPhanie Guyomard-Rabenirina
EPI_ISL_2531962	Labo Analyses Med	National Reference Center for Viruses of Respiratory Infections, Institut Pasteur, Paris	Marion Barbet, Sylvie Behillil, Méline Bizard, Angela Brisebarre, Camille Capel, Vincent Enouf, Louise Lefrançois, Frédéric Lemoine, Christophe Malabat, Corinne Maufrais, Etienne Simon-Lorière, Maud Vanpeene, Sylvie Van der Werf ,Vicky Le
EPI_ISL_2532279, EPI_ISL_2532280, EPI_ISL_2532281	Labo Analyses Med	National Reference Center for Viruses of Respiratory Infections, Institut Pasteur, Paris	Marion Barbet, Sylvie Behillil, Méline Bizard, Angela Brisebarre, Camille Capel, Vincent Enouf, Louise Lefrançois, Frédéric Lemoine, Christophe Malabat, Corinne Maufrais, Etienne Simon-Lorière, Maud Vanpeene, Sylvie Van der Werf ,OphéLie Said-Delattre
EPI_ISL_2532284, EPI_ISL_2532285, EPI_ISL_2532286	Labo Analyses Med	National Reference Center for Viruses of Respiratory Infections, Institut Pasteur, Paris	Marion Barbet, Sylvie Behillil, Méline Bizard, Angela Brisebarre, Camille Capel, Vincent Enouf, Louise Lefrançois, Frédéric Lemoine, Christophe Malabat, Corinne Maufrais, Etienne Simon-Lorière, Maud Vanpeene, Sylvie Van der Werf ,Eve Haguenoer
EPI_ISL_2532287	Labo Analyses Med	National Reference Center for Viruses of Respiratory Infections, Institut Pasteur, Paris	Marion Barbet, Sylvie Behillil, Méline Bizard, Angela Brisebarre, Camille Capel, Vincent Enouf, Louise Lefrançois, Frédéric Lemoine, Christophe Malabat, Corinne Maufrais, Etienne Simon-Lorière, Maud Vanpeene, Sylvie Van der Werf ,GréGoire Potiron
EPI_ISL_2532311, EPI_ISL_2532312, EPI_ISL_2532313	Hospital	National Reference Center for Viruses of Respiratory Infections, Institut Pasteur, Paris	Marion Barbet, Sylvie Behillil, Méline Bizard, Angela Brisebarre, Camille Capel, Vincent Enouf, Louise Lefrançois, Frédéric Lemoine, Christophe Malabat, Corinne Maufrais, Etienne Simon-Lorière, Maud Vanpeene, Sylvie Van der Werf ,Pascale Martres
EPI_ISL_2532315	Hospital	National Reference Center for Viruses of Respiratory Infections, Institut Pasteur, Paris	Marion Barbet, Sylvie Behillil, Méline Bizard, Angela Brisebarre, Camille Capel, Vincent Enouf, Louise Lefrançois, Frédéric Lemoine, Christophe Malabat, Corinne Maufrais, Amaury Vaysse, Etienne Simon-Lorière, Maud Vanpeene, Sylvie Van der Werf ,Pascale Martres
EPI_ISL_2532388	Hospital	National Reference Center for Viruses of Respiratory Infections, Institut Pasteur, Paris	Marion Barbet, Sylvie Behillil, Méline Bizard, Angela Brisebarre, Camille Capel, Vincent Enouf, Louise Lefrançois, Frédéric Lemoine, Christophe Malabat, Corinne Maufrais, Etienne Simon-Lorière, Maud Vanpeene, Sylvie Van der Werf ,AgnèS Scanvic
EPI_ISL_2532412	Labo Analyses Med	National Reference Center for Viruses of Respiratory Infections, Institut Pasteur, Paris	Marion Barbet, Sylvie Behillil, Méline Bizard, Angela Brisebarre, Camille Capel, Vincent Enouf, Louise Lefrançois, Frédéric Lemoine, Christophe Malabat, Corinne Maufrais, Etienne Simon-Lorière, Maud Vanpeene, Sylvie Van der Werf ,Pierre-Yves Leonard
EPI_ISL_2532465	Labo Analyses Med	National Reference Center for Viruses of Respiratory Infections, Institut Pasteur, Paris	Marion Barbet, Sylvie Behillil, Méline Bizard, Angela Brisebarre, Camille Capel, Vincent Enouf, Louise Lefrançois, Frédéric Lemoine, Christophe Malabat, Corinne Maufrais, Etienne Simon-Lorière, Maud Vanpeene, Sylvie Van der Werf
EPI_ISL_2533826	Laboratoire Virologie Saint Louis APHP	Laboratoire Virologie Saint Louis APHP	Maud Salmona, Marie Laure Chaix, Severine Mercier Delarue, Marie Laure Néré, Linda Feghoul, Jérome Le Goff, Constance Delauguerre, Sophia Achaibou
EPI_ISL_2535237	Labo Analyses Med	National Reference Center for Viruses of Respiratory Infections, Institut Pasteur, Paris	Marion Barbet, Sylvie Behillil, Méline Bizard, Angela Brisebarre, Camille Capel, Vincent Enouf, Louise Lefrançois, Frédéric Lemoine, Christophe Malabat, Corinne Maufrais, Etienne Simon-Lorière, Maud Vanpeene, Sylvie Van der Werf
EPI_ISL_2535271	Hospital	National Reference Center for Viruses of Respiratory Infections, Institut Pasteur, Paris	Marion Barbet, Sylvie Behillil, Méline Bizard, Angela Brisebarre, Camille Capel, Vincent Enouf, Louise Lefrançois, Frédéric Lemoine, Christophe Malabat, Corinne Maufrais, Damien Mornico, Etienne Simon-Lorière, Maud Vanpeene, Sylvie Van der Werf ,Pascale Martres
EPI_ISL_2535274	Hospital	National Reference Center for Viruses of Respiratory Infections, Institut Pasteur, Paris	Marion Barbet, Sylvie Behillil, Méline Bizard, Angela Brisebarre, Camille Capel, Vincent Enouf, Louise Lefrançois, Frédéric Lemoine, Christophe Malabat, Corinne Maufrais, Etienne Simon-Lorière, Maud Vanpeene, Sylvie Van der Werf ,Pascale Martres
EPI_ISL_2535276	Labo Analyses Med	National Reference Center for Viruses of Respiratory Infections, Institut Pasteur, Paris	Marion Barbet, Sylvie Behillil, Méline Bizard, Angela Brisebarre, Camille Capel, Vincent Enouf, Louise Lefrançois, Frédéric Lemoine, Christophe Malabat, Corinne Maufrais, Victoire Baillet, Etienne Simon-Lorière, Maud Vanpeene, Sylvie Van der Werf ,OphéLie Said-Delattre
EPI_ISL_2535281	Labo Analyses Med	National Reference Center for Viruses of Respiratory Infections, Institut Pasteur, Paris	Marion Barbet, Sylvie Behillil, Méline Bizard, Angela Brisebarre, Camille Capel, Vincent Enouf, Louise Lefrançois, Frédéric Lemoine, Christophe Malabat, Corinne Maufrais, Etienne Simon-Lorière, Maud Vanpeene, Sylvie Van der Werf ,OphéLie Said-Delattre
EPI_ISL_2535524	Labo Analyses Med	National Reference Center for Viruses of Respiratory Infections, Institut Pasteur, Paris	Marion Barbet, Sylvie Behillil, Méline Bizard, Angela Brisebarre, Camille Capel, Vincent Enouf, Louise Lefrançois, Frédéric Lemoine, Christophe Malabat, Corinne Maufrais, Etienne Simon-Lorière, Maud Vanpeene, Sylvie Van der Werf , Hugues Leroy
EPI_ISL_2535555	Hospital	National Reference Center for Viruses of Respiratory Infections, Institut Pasteur, Paris	Marion Barbet, Sylvie Behillil, Méline Bizard, Angela Brisebarre, Camille Capel, Vincent Enouf, Louise Lefrançois, Frédéric Lemoine, Christophe Malabat, Corinne Maufrais, Etienne Simon-Lorière, Maud Vanpeene, Sylvie Van der Werf ,Isabelle Woercel

EPI_ISL_2535559	Hospital	National Reference Center for Viruses of Respiratory Infections, Institut Pasteur, Paris	Marion Barbet, Sylvie Behillil, Méline Bizard, Angela Brisebarre, Camille Capel, Vincent Enouf, Louise Lefrançois, Frédéric Lemoine, Christophe Malabat, Corinne Maufrais, Etienne Simon-Lorière, Maud Vanpeene, Sylvie Van der Werf ,CéCile Farrugia
EPI_ISL_2535560	Labo Analyses Med	National Reference Center for Viruses of Respiratory Infections, Institut Pasteur, Paris	Marion Barbet, Sylvie Behillil, Méline Bizard, Angela Brisebarre, Camille Capel, Vincent Enouf, Louise Lefrançois, Frédéric Lemoine, Christophe Malabat, Corinne Maufrais, Etienne Simon-Lorière, Maud Vanpeene, Sylvie Van der Werf ,Benedicte Baccouch
EPI_ISL_2536001	Gravity Diagnostics, LLC	Gravity Diagnostics, LLC	Gravity Diagnostics
EPI_ISL_2538830	Lighthouse Lab in Milton Keynes	Wellcome Sanger Institute for the COVID-19 Genomics UK (COG-UK) Consortium	The Lighthouse Lab in Milton Keynes and Alex Alderton, Roberto Amato, Jeffrey Barrett, Sonia Goncalves, Ewan Harrison, David K. Jackson, Ian Johnston, Dominic Kwiatkowski, Cordelia Langford, John Sillitoe on behalf of the Wellcome Sanger Institute COVID-19 Surveillance Team
EPI_ISL_2543479	Genetica Molecular and Subdepartamento de Virologia ISP Chile	Instituto de Salud Publica de Chile	Karen Orostica, Constanza Campano, Barbara Parra, Loredana Arata, Gisselle Barra, Patricia Bustos, Rodrigo Fasce, Javier Tognarelli, Andres Castillo, Soledad Ulloa, Jorge Fernandez
EPI_ISL_2544259	Invenimus AG	Institute of Medical Virology	Verena Kufner, Gabriela Ziltener, Maryam Zaheri, Stefan Schmutz, Annette Audigé, Maria Grünberg, Kevin Steiner, Jon Huder, Cyril Shah, Riccarda Capaul, Guido Bloemberg, Jürg Böni, Michael Huber, Alexandra Trkola
EPI_ISL_2544783	MD PHL	MD PHL	Maryland Department of Health Laboratories Administration
EPI_ISL_2544916, EPI_ISL_2544922, EPI_ISL_2544951, EPI_ISL_2544960, EPI_ISL_2544970	UW Virology Lab	UW Virology Lab	Pavitra Roychoudhury, Hong Xie, Lasata Shrestha, Shah Mohamed Bakhash, Tien V. Nguyen, Noah R. Baker, Sean Ellis, Meei-Li Huang, Keith R Jerome, Alexander Greninger
EPI_ISL_2545648	LHUB-ULB	Labo Klinische Biologie, UZA	Jasmine Coppens, Marie Le Mercier, Basil Britto Xavier, Christine Lammens, Veerle Matheeussen, Herman Goossens
EPI_ISL_2547365	Calvinia Hospital	National Health Laboratory Service/University of Cape Town (NHLS/UCT)	Arash Iranzadeh, Deelan Doolabh, Lynn Tyers, Bruna Galvao, Innocent Mudau, Marvin Hsiao, Gert Marais, Diana Hardie, Stephen Korsman, Carolyn Williamson
EPI_ISL_2547367	Vredendal North Clinic wc VDN	National Health Laboratory Service/University of Cape Town (NHLS/UCT)	Arash Iranzadeh, Deelan Doolabh, Lynn Tyers, Bruna Galvao, Innocent Mudau, Marvin Hsiao, Gert Marais, Diana Hardie, Stephen Korsman, Carolyn Williamson
EPI_ISL_2547370	Kuruman Hospital	National Health Laboratory Service/University of Cape Town (NHLS/UCT)	Arash Iranzadeh, Deelan Doolabh, Lynn Tyers, Bruna Galvao, Innocent Mudau, Marvin Hsiao, Gert Marais, Diana Hardie, Stephen Korsman, Carolyn Williamson
EPI_ISL_2547371	Postdene Clinic	National Health Laboratory Service/University of Cape Town (NHLS/UCT)	Arash Iranzadeh, Deelan Doolabh, Lynn Tyers, Bruna Galvao, Innocent Mudau, Marvin Hsiao, Gert Marais, Diana Hardie, Stephen Korsman, Carolyn Williamson
EPI_ISL_2547411, EPI_ISL_2547413	2 Military Hospital wc MAA	National Health Laboratory Service/University of Cape Town (NHLS/UCT)	Arash Iranzadeh, Deelan Doolabh, Lynn Tyers, Bruna Galvao, Innocent Mudau, Marvin Hsiao, Gert Marais, Diana Hardie, Stephen Korsman, Carolyn Williamson
EPI_ISL_2547415	Knysna Hospital wc KNY	National Health Laboratory Service/University of Cape Town (NHLS/UCT)	Arash Iranzadeh, Deelan Doolabh, Lynn Tyers, Bruna Galvao, Innocent Mudau, Marvin Hsiao, Gert Marais, Diana Hardie, Stephen Korsman, Carolyn Williamson
EPI_ISL_2547418, EPI_ISL_2547419	Groote Schuur Hospital wc GSH	National Health Laboratory Service/University of Cape Town (NHLS/UCT)	Arash Iranzadeh, Deelan Doolabh, Lynn Tyers, Bruna Galvao, Innocent Mudau, Marvin Hsiao, Gert Marais, Diana Hardie, Stephen Korsman, Carolyn Williamson
EPI_ISL_2547420	Nelspoort Clinic wc NPP	National Health Laboratory Service/University of Cape Town (NHLS/UCT)	Arash Iranzadeh, Deelan Doolabh, Lynn Tyers, Bruna Galvao, Innocent Mudau, Marvin Hsiao, Gert Marais, Diana Hardie, Stephen Korsman, Carolyn Williamson
EPI_ISL_2547421, EPI_ISL_2547422	2 Military Hospital wc MAA	National Health Laboratory Service/University of Cape Town (NHLS/UCT)	Arash Iranzadeh, Deelan Doolabh, Lynn Tyers, Bruna Galvao, Innocent Mudau, Marvin Hsiao, Gert Marais, Diana Hardie, Stephen Korsman, Carolyn Williamson
EPI_ISL_2547424, EPI_ISL_2547425	Groote Schuur Hospital wc GSH	National Health Laboratory Service/University of Cape Town (NHLS/UCT)	Arash Iranzadeh, Deelan Doolabh, Lynn Tyers, Bruna Galvao, Innocent Mudau, Marvin Hsiao, Gert Marais, Diana Hardie, Stephen Korsman, Carolyn Williamson
EPI_ISL_2547426	Great Brak River Clinic wc GBC	National Health Laboratory Service/University of Cape Town (NHLS/UCT)	Arash Iranzadeh, Deelan Doolabh, Lynn Tyers, Bruna Galvao, Innocent Mudau, Marvin Hsiao, Gert Marais, Diana Hardie, Stephen Korsman, Carolyn Williamson
EPI_ISL_2547427	Hornlee Clinic wc HLC	National Health Laboratory Service/University of Cape Town (NHLS/UCT)	Arash Iranzadeh, Deelan Doolabh, Lynn Tyers, Bruna Galvao, Innocent Mudau, Marvin Hsiao, Gert Marais, Diana Hardie, Stephen Korsman, Carolyn Williamson
EPI_ISL_2549077	Furst Medical Laboratory	Norwegian Institute of Public Health, Department of Virology	Kathrine Stene-Johansen, Kamilla Heddeland Instefjord, Hilde Elshaug, Garcia Llorente Ignacio, Jon Bråte, Engebretsen Serina Beate,Pedersen Benedikte Nevjen, Line Victoria Moen, Debech Nadia, Atiya R Ali,Marie Paulsen Madsen, Rasmus Riis Kopperud, Hilde Vollan, Karoline Bragstad, Olav Hungnes
EPI_ISL_2550817	Emory Molecular Diagnostics Laboratory, Emory Healthcare	Piantadosi Lab, Emory Department of Pathology	Ahmed Babiker, Dara Khosravi, Anne Piantadosi

EPI_ISL_2554885	Lighthouse Lab in Alderley Park	Wellcome Sanger Institute for the COVID-19 Genomics UK (COG-UK) Consortium	Jacquelyn Wynn, Mairead Hyland, The Lighthouse Lab in Alderley Park and Alex Alderton, Roberto Amato, Jeffrey Barrett, Sonia Goncalves, Ewan Harrison, David K. Jackson, Ian Johnston, Dominic Kwiatkowski, Cordelia Langford, John Sillitoe on behalf of the Wellcome Sanger Institute COVID-19 Surveillance Team
EPI_ISL_2557932	Oregon State Public Health Laboratory	Oregon State Public Health Laboratory	Rafia Razzaque, Eugene Yeboah, Vanda Makris, Laura Tsaknaridis, John Fontana and Shane Sevey
EPI_ISL_2558623	Wisconsin State Laboratory of Hygiene Communicable Disease Division	Wisconsin State Laboratory of Hygiene Communicable Disease Division	Abigail C. Shockey, Alicia J. Mooney, Erika M. Hanson, Tonya Danz, Richard Griesser, Sara Wagner, Kelsey R. Florek
EPI_ISL_2562728	NC State Laboratory of Public Health	Genomics and Discovery, Respiratory Viruses Branch, Division of Viral Diseases, Centers for Disease Control and Prevention	Yan Li, Jing Zhang, Anna Kelleher, Ying Tao, Brian Lynch, Krista Queen, Anna Uehara, Peter Cook, Han Jia Justin Ng, Rachel Marine, Clinton R. Paden, Dhwani Batra, Haibin Wang, Tara Coalter, Jasmine Padilla, Morgan Davis, Mili Sheth, Sarah Nobles, Mark Burroughs, Justin Lee, Adam Retchless, Suxiang Tong
EPI_ISL_2566449	Department of Public Health Bucharest	National Institute of Infectious Diseases-Prof. Dr. Matei Bals Molecular Diagnostics Laboratory	Corina Casangiu, Leontina Banica, Marius Surleac, Petre Milu, Robert Hohan, Simona Paraschiv, Dan Otelea
EPI_ISL_2566591	Hospital Universitari Joan XXIII	Hospital Universitari Joan XXIII	Gemma Recio, Cristina Gutiérrez, Clara Benavent, Carla Martín, Margarida Terrón, Ester Picó, Natalia Bastón
EPI_ISL_2567051, EPI_ISL_2567063	Shamir Medical Center (Asaf Harofe)	Shamir Medical Center (Asaf Harofe)	Nir Rainy,Chen Weiner,Reut Sorek Abramovich,Yevgeni Yegorov,Anna Vishnevsky,Patricia Benveniste-Lekovitz,Abu Hamad Ramzia,Adina Bar Chaim
EPI_ISL_2567368	CENTRE HOSPITALIER SUD GIRONDE	CNR Virus des Infections Respiratoires - France SUD	Antonin Bal, Gregory Destras, Gwendolyne Burfin, Hadrien Regue, Quentin Semanas, Martine Valette, Bruno Lina, Laurence Josset
EPI_ISL_2567373	CENTRE HOSPITALIER POITIERS	CNR Virus des Infections Respiratoires - France SUD	Antonin Bal, Gregory Destras, Gwendolyne Burfin, Hadrien Regue, Quentin Semanas, Martine Valette, Bruno Lina, Laurence Josset
EPI_ISL_2567384, EPI_ISL_2567391	LBM UNIBIO VALENTIN	CNR Virus des Infections Respiratoires - France SUD	Antonin Bal, Gregory Destras, Gwendolyne Burfin, Hadrien Regue, Quentin Semanas, Martine Valette, Bruno Lina, Laurence Josset
EPI_ISL_2567437, EPI_ISL_2567460	MIRIALIS CLUSES BECHET	CNR Virus des Infections Respiratoires - France SUD	Antonin Bal, Gregory Destras, Gwendolyne Burfin, Hadrien Regue, Quentin Semanas, Martine Valette, Bruno Lina, Laurence Josset
EPI_ISL_2567463, EPI_ISL_2567464, EPI_ISL_2567465	GROUPE LCD - UNIBIO LA PLAINE	CNR Virus des Infections Respiratoires - France SUD	Antonin Bal, Gregory Destras, Gwendolyne Burfin, Hadrien Regue, Quentin Semanas, Martine Valette, Bruno Lina, Laurence Josset
EPI_ISL_2567469	BIOGROUP LABORATOIRE ST DIE	CNR Virus des Infections Respiratoires - France SUD	Antonin Bal, Gregory Destras, Gwendolyne Burfin, Hadrien Regue, Quentin Semanas, Martine Valette, Bruno Lina, Laurence Josset
EPI_ISL_2567508	LBM UNIBIO VALENTIN	CNR Virus des Infections Respiratoires - France SUD	Antonin Bal, Gregory Destras, Gwendolyne Burfin, Hadrien Regue, Quentin Semanas, Martine Valette, Bruno Lina, Laurence Josset
EPI_ISL_2568858, EPI_ISL_2570464	Lighthouse Lab in Milton Keynes	Wellcome Sanger Institute for the COVID-19 Genomics UK (COG-UK) Consortium	The Lighthouse Lab in Milton Keynes and Alex Alderton, Roberto Amato, Jeffrey Barrett, Sonia Goncalves, Ewan Harrison, David K. Jackson, Ian Johnston, Dominic Kwiatkowski, Cordelia Langford, John Sillitoe on behalf of the Wellcome Sanger Institute COVID-19 Surveillance Team
EPI_ISL_2574071	Center for Laboratory Medicine	Center for Laboratory Medicine	Yannick Gerth
EPI_ISL_2598363	Hospital General Universitario de Alicante - Instituto de Investigación Sanitaria y Biomédica de Alicante	SeqCOVID-SPAIN consortium/IBV(CSIC)	Maripaz Ventero Martín, Carmen Molina Pardines and SeqCOVID-SPAIN consortium
EPI_ISL_2599733	Quest Diagnostics Incorporated	Centers for Disease Control and Prevention Division of Viral Diseases, Pathogen Discovery	Dakota Howard, Dhwani Batra, Peter W. Cook, Kara Moser, Adrian Paskey, Jason Caravas, Benjamin Rambo-Martin, Shatavia Morrison, Christopher Gulvick, Scott Sammons, Yvette Unoarumhi, Darlene Wagner, Matthew Schmerer, S. H. Rosenthal, A. Gerasimova, R. M. Kagan, B. Anderson, M. Hua, Y. Liu, L.E. Bernstein, K.E. Livingston, A. Perez, I. A. Shlyakhter, R. V. Rolando, R. Owen, P. Tanpaiboon, F. Lacbawan, Clinton R. Paden, Duncan MacCannell
EPI_ISL_2599792	Dianalabs SA	Genesupport	Tanguy ARAUD, Geraldine Jost, Katia Jaton, Nadia Liassine
EPI_ISL_2599879	Quest Diagnostics Incorporated	Centers for Disease Control and Prevention Division of Viral Diseases, Pathogen Discovery	Dakota Howard, Dhwani Batra, Peter W. Cook, Kara Moser, Adrian Paskey, Jason Caravas, Benjamin Rambo-Martin, Shatavia Morrison, Christopher Gulvick, Scott Sammons, Yvette Unoarumhi, Darlene Wagner, Matthew Schmerer, S. H. Rosenthal, A. Gerasimova, R. M. Kagan, B. Anderson, M. Hua, Y. Liu, L.E. Bernstein, K.E. Livingston, A. Perez, I. A. Shlyakhter, R. V. Rolando, R. Owen, P. Tanpaiboon, F. Lacbawan, Clinton R. Paden, Duncan MacCannell
EPI_ISL_2600368	Institute of Epidemiology, Disease Control and Research (IEDCR)	IEDCR-ideSHi-icddr,b	Hassan Afrad, Sadia Rahman, Manjur Hossain Khan, Firdausi Qadri, Tahmina Shirin
EPI_ISL_2602434	Laboratorio di Genetica Medica Ospedale Belcolle	INMI Lazzaro Spallanzani IRCCS	F Santini, F Messina, O Butera, M Rueca, S Donzelli, M Pallocca, F Natoni, G Pessina, E Giombini
EPI_ISL_2602697, EPI_ISL_2602707	KEMRI-Wellcome Trust Research Programme,Kilifi	KEMRI-Wellcome Trust Research Programme,Kilifi	Githinji G.,Mohamed K.S.,de Laurent Z.,Mburu M.W.,Matoke D.,Onyango C.,
EPI_ISL_2606787	Lighthouse Lab in Milton Keynes	Wellcome Sanger Institute for the COVID-19 Genomics UK (COG-UK) Consortium	The Lighthouse Lab in Milton Keynes and Alex Alderton, Roberto Amato, Jeffrey Barrett, Sonia Goncalves, Ewan Harrison, David K. Jackson, Ian Johnston, Dominic Kwiatkowski, Cordelia Langford, John Sillitoe on behalf of the Wellcome Sanger Institute COVID-19 Surveillance Team

EPI_ISL_2612528	Centracare Laboratory Services	Minnesota Department of Health, Public Health Laboratory	Alexandra Lorentz, Jacob Garfin, Matt Plumb, and Xiong Wang
EPI_ISL_2612834, EPI_ISL_2612835	SYNLAB	GIGA Medical Genomics	Keith Durkin, Maria Artesi, Sébastien Bontems, Raphaël Boreux, Bouchra Boujemla, Nathalie Renotte, Cécile Meex, Pierrette Melin, Marie-Pierre Hayette, Vincent Bours
EPI_ISL_2615283, EPI_ISL_2615285, EPI_ISL_2615335, EPI_ISL_2615385	Swedish national genomic surveillance program of SARS-CoV-2	The Public Health Agency of Sweden	Maximilian Riess, Maria Lind Karlberg, Alma Brolund, Swedish national genomic surveillance program of SARS-CoV-2
EPI_ISL_2615889	Berkeley Medical Center	WVU and Marshall University Combined Genomics Core Facilities	James Denvir, Peter Stoilov, Peter Perrotta, Wesley Kimble, Ryan Percifield
EPI_ISL_2616364	Austrian Agency for Health and Food Safety (AGES)	Bergthaler laboratory, CeMM Research Center for Molecular Medicine of the Austrian Academy of Sciences	Lukas Endler, Anna Schedl, Fabian Amman, Petr Triska, Matthew Thornton, Thomas Penz, Benedikt Agerer, Maelle Le Moing, Michael Schuster, Bekir Erguner, Jan Laine, Martin Senekowitsch, Christoph Bock, Andreas Bergthaler
EPI_ISL_2618719	UW Virology Lab	UW Virology Lab	Pavitra Roychoudhury, Hong Xie, Lasata Shrestha, Shah Mohamed Bakhash, Tien V. Nguyen, Noah R. Baker, Sean Ellis, Meei-Li Huang, Keith R Jerome, Alexander Greninger
EPI_ISL_2618857, EPI_ISL_2618993, EPI_ISL_2619469, EPI_ISL_2619470, EPI_ISL_2619551, EPI_ISL_2619560, EPI_ISL_2619730, EPI_ISL_2619773, EPI_ISL_2619778, EPI_ISL_2619968, EPI_ISL_2619981, EPI_ISL_2620413			
see above	Swedish national genomic surveillance program of SARS-CoV-2	The Public Health Agency of Sweden	Maximilian Riess, Maria Lind Karlberg, Alma Brolund, Swedish national genomic surveillance program of SARS-CoV-2
EPI_ISL_2621132, EPI_ISL_2621133	Groote Schuur Hospital wc GSH	NHLS/UCT	Arash Iranzadeh, Deelan Doolabh, Lynn Tyers, Bruna Galvao, Innocent Mudau, Marvin Hsiao, Gert Marais, Diana Hardie, Stephen Korsman, Carolyn Williamson
EPI_ISL_2621136	2 Military Hospital wc MAA	NHLS/UCT	Arash Iranzadeh, Deelan Doolabh, Lynn Tyers, Bruna Galvao, Innocent Mudau, Marvin Hsiao, Gert Marais, Diana Hardie, Stephen Korsman, Carolyn Williamson
EPI_ISL_2621137	George Hospital wc GRH	NHLS/UCT	Arash Iranzadeh, Deelan Doolabh, Lynn Tyers, Bruna Galvao, Innocent Mudau, Marvin Hsiao, Gert Marais, Diana Hardie, Stephen Korsman, Carolyn Williamson
EPI_ISL_2621143	D'Almeida Clinic wc DAL	NHLS/UCT	Arash Iranzadeh, Deelan Doolabh, Lynn Tyers, Bruna Galvao, Innocent Mudau, Marvin Hsiao, Gert Marais, Diana Hardie, Stephen Korsman, Carolyn Williamson
EPI_ISL_2621144	2 Military Hospital wc MAA	NHLS/UCT	Arash Iranzadeh, Deelan Doolabh, Lynn Tyers, Bruna Galvao, Innocent Mudau, Marvin Hsiao, Gert Marais, Diana Hardie, Stephen Korsman, Carolyn Williamson
EPI_ISL_2621145	Knysna CDC wc WLC	NHLS/UCT	Arash Iranzadeh, Deelan Doolabh, Lynn Tyers, Bruna Galvao, Innocent Mudau, Marvin Hsiao, Gert Marais, Diana Hardie, Stephen Korsman, Carolyn Williamson
EPI_ISL_2621146	Dysselsdorp Clinic wc DDC	NHLS/UCT	Arash Iranzadeh, Deelan Doolabh, Lynn Tyers, Bruna Galvao, Innocent Mudau, Marvin Hsiao, Gert Marais, Diana Hardie, Stephen Korsman, Carolyn Williamson
EPI_ISL_2621738, EPI_ISL_2621752, EPI_ISL_2621763, EPI_ISL_2621765, EPI_ISL_2621769, EPI_ISL_2621773	SARS-CoV-2 testing team, National Institute of Infectious Diseases	Pathogen Genomics Center, National Institute of Infectious Diseases	Tsuyoshi Sekizuka, Kentaro Itokawa, Rina Tanaka, Masanori Hashino, Nozomu Hanaoka, Masumichi Saito, Naomi Nojiri, Hazuka Y Furihata, Sana Uchikoba, Hiromizu Takahashi, Tsuguto Fujimoto, Makoto Kuroda
EPI_ISL_2621921	National Public Health Laboratory, National Centre for Infectious Diseases	National Public Health Laboratory, National Centre for Infectious Diseases	Tze Minn Mak, Zhenyang Zhou, Royce Ang, Lin Cui, Raymond Tzer Pin Lin
EPI_ISL_2622100	CHUV	Laboratory of genomics and metagenomics	Trestan Pillonel, Damien Jacot, Sébastien Aeby, Gilbert Greub, Claire Bertelli
EPI_ISL_2623677	Quadram Institute Bioscience	COVID-19 Genomics UK (COG-UK) Consortium	Dave J. Baker, Gemma L. Kay, Alp Aydin, Thanh Le-Viet, Steven Rudder, Ana P. Tedim, Anastasia Kolyva, Maria Diaz, Leonardo de Oliveira Martins, Nabil-Fareed Alikhan, Lizzie Meadows, Rachael Stanley, Ngozi Elumogo, Muhammed Yasir, Nicholas M. Thomson, Alexander J Trotter, Rachel Gilroy, Samuel Bloomfield, Claire Stuart, Andrew Bell, Reenesh Prakash, Samir Dervisevic, Alison E. Mather, John Wain, Mark Webber, Andrew J. Page, Justin O'Grady
EPI_ISL_2624573	Oxford Viromics, NDM, University of Oxford; Oxford University Hospitals; Basingstoke and North Hampshire Hospital	COVID-19 Genomics UK (COG-UK) Consortium	Tanya Golubchik, David Bonsall, George Macintyre, Amy Trebes, Mariateresa de Cesare, Catrin Moore, Alex Mobbs, Anita Justice, Robert Shaw, Monique Andersson, Timothy Peto, Emma Wise, Nathan Moore, Jessica Lynch, Nick Cortes, Matilde Mori, Stephen Kidd, David Buck, John Todd, Christophe Fraser
EPI_ISL_2626081	Clinique Saint-Pierre Ottignies	UCLouvain/IREC/MBLG	Jean Ruelle, Bertrand Bearzatto, Benoit Kabamba Mukadi
EPI_ISL_2626175	HOSPITAL UNIVERSITARIO SON ESPASES	HOSPITAL UNIVERSITARIO SON ESPASES	Servicio de Microbiología, Hospital Universitario Son Espases
EPI_ISL_2626181	Usansolo-Galdakao University Hospital	Cruces University Hospital	Ana Gual-de-Torrella, Izaskun Alejo-Cancho, Mikel Gallego, Ana Belén de la Hoz
EPI_ISL_2626872	CENTRUM VOOR MEDISCHE ANALYSE	Labo Klinische Biologie, UZA	Marie Le Mercier, Jasmine Coppens, Basil Britto Xavier, Christine Lammens, Veerle Matheeussen, Herman Goossens
EPI_ISL_2626938	TEMPUS LABS INC	Wadsworth Center, New York State Department of Health	Kirsten St. George, Daryl M. Lamson, Alexis Russell, Matthew Shudt, Melissa A Leisner, Jonathan Plitnick, Catharine Prussing, Navjot Singh, John Kelly, Erasmus Schneider, Erica Lasek-Nesselquist
EPI_ISL_2627001	ALBANY MEDICAL CENTER	Wadsworth Center, New York State Department of Health	Kirsten St. George, Daryl M. Lamson, Alexis Russell, Matthew Shudt, Melissa A Leisner, Jonathan Plitnick, Catharine Prussing, Navjot Singh, John Kelly, Erasmus Schneider, Erica Lasek-Nesselquist
EPI_ISL_2627062	URMC LABS	Wadsworth Center, New York State Department of Health	Kirsten St. George, Daryl M. Lamson, Alexis Russell, Matthew Shudt, Melissa A Leisner, Jonathan Plitnick, Catharine Prussing, Navjot Singh, John Kelly, Erasmus Schneider, Erica Lasek-Nesselquist

EPI_ISL_2627948	Hospital	National Reference Center for Viruses of Respiratory Infections, Institut Pasteur, Paris	Marion Barbet, Sylvie Behillil, Méline Bizard, Angela Brisebarre, Camille Capel, Vincent Enouf, Louise Lefrançois, Frédéric Lemoine, Christophe Malabat, Corinne Maufrais, Etienne Simon-Lorière, Maud Vanpeene, Sylvie Van der Werf ,ValéRie Serazin
EPI_ISL_2627953, EPI_ISL_2627954, EPI_ISL_2627955	Hospital	National Reference Center for Viruses of Respiratory Infections, Institut Pasteur, Paris	Marion Barbet, Sylvie Behillil, Méline Bizard, Angela Brisebarre, Camille Capel, Vincent Enouf, Louise Lefrançois, Frédéric Lemoine, Christophe Malabat, Corinne Maufrais, Etienne Simon-Lorière, Maud Vanpeene, Sylvie Van der Werf ,Pascale Martres
EPI_ISL_2627961, EPI_ISL_2627963	Hospital	National Reference Center for Viruses of Respiratory Infections, Institut Pasteur, Paris	Marion Barbet, Sylvie Behillil, Méline Bizard, Angela Brisebarre, Camille Capel, Vincent Enouf, Louise Lefrançois, Frédéric Lemoine, Christophe Malabat, Corinne Maufrais, Etienne Simon-Lorière, Maud Vanpeene, Sylvie Van der Werf ,JéRome Guinard
EPI_ISL_2627975, EPI_ISL_2627976, EPI_ISL_2627978	Hospital	National Reference Center for Viruses of Respiratory Infections, Institut Pasteur, Paris	Marion Barbet, Sylvie Behillil, Méline Bizard, Angela Brisebarre, Camille Capel, Vincent Enouf, Louise Lefrançois, Frédéric Lemoine, Christophe Malabat, Corinne Maufrais, Etienne Simon-Lorière, Maud Vanpeene, Sylvie Van der Werf ,Pascale Martres
EPI_ISL_2627993, EPI_ISL_2627994	Hospital	National Reference Center for Viruses of Respiratory Infections, Institut Pasteur, Paris	Marion Barbet, Sylvie Behillil, Méline Bizard, Angela Brisebarre, Camille Capel, Vincent Enouf, Louise Lefrançois, Frédéric Lemoine, Christophe Malabat, Corinne Maufrais, Etienne Simon-Lorière, Maud Vanpeene, Sylvie Van der Werf ,Nabil Gastli
EPI_ISL_2628151	Hospital Universitari Vall d'Hebron - Vall d'Hebron Institut de Recerca	Hospital Universitari Vall d'Hebron - Vall d'Hebron Institut de Recerca	Cristina Andrés, Maria Piñana, Alejandra González-Sánchez, Damir Garcia-Cehic, Ariadna Rando, Juliana Esperalba, Maria Gema Codina, Carla Castillo, Maria Carmen Martin, Tomàs Pumarola, Josep Quer, Andrés Antón
EPI_ISL_2629257, EPI_ISL_2629313	LAM GEN-BIO GRAVANCHES	CNR Virus des Infections Respiratoires - France SUD	Antonin Bal, Gregory Destras, Gwendolyne Burfin, Hadrien Regue, Quentin Semanas, Martine Valette, Bruno Lina, Laurence Josset
EPI_ISL_2629520	PLATEAU TECHNIQUE L'ABO+	CNR Virus des Infections Respiratoires - France SUD	Antonin Bal, Gregory Destras, Gwendolyne Burfin, Hadrien Regue, Quentin Semanas, Martine Valette, Bruno Lina, Laurence Josset
EPI_ISL_2629523, EPI_ISL_2629525, EPI_ISL_2629528, EPI_ISL_2629529, EPI_ISL_2629532, EPI_ISL_2629533	CERBAILLIANCE LA REUNION	CNR Virus des Infections Respiratoires - France SUD	Antonin Bal, Gregory Destras, Gwendolyne Burfin, Hadrien Regue, Quentin Semanas, Martine Valette, Bruno Lina, Laurence Josset
EPI_ISL_2629564	LABM DU PRE - LABOMAINE	CNR Virus des Infections Respiratoires - France SUD	Antonin Bal, Gregory Destras, Gwendolyne Burfin, Hadrien Regue, Quentin Semanas, Martine Valette, Bruno Lina, Laurence Josset
EPI_ISL_2631153	Oregon State Public Health Laboratory	Oregon State Public Health Laboratory	Rafia Razzaque, Eugene Yeboah, Vanda Makris, Laura Tsaknaridis, John Fontana and Shane Sevey
EPI_ISL_2631566, EPI_ISL_2631582	SYNLAB MVZ Leinfelden-Echterdingen	Robert Koch Institute	unknown
EPI_ISL_2631601	SYNLAB MVZ Leverkusen	Robert Koch Institute	unknown
EPI_ISL_2631616, EPI_ISL_2631694	SYNLAB MVZ Leinfelden-Echterdingen	Robert Koch Institute	unknown
EPI_ISL_2631828	Labor Dr. Wisplinghoff - Köln	Robert Koch Institute	unknown
EPI_ISL_2631923, EPI_ISL_2631924, EPI_ISL_2631930, EPI_ISL_2631931	Eurofins LifeCodexx GmbH	Robert Koch Institute	unknown
EPI_ISL_2632328	Niedersächsisches Landesgesundheitsamt (NLGA)	Robert Koch Institute	unknown
EPI_ISL_2632382	Sonic - Bioscientia - MVZ Labor Saar GmbH	Robert Koch Institute	unknown
EPI_ISL_2632814, EPI_ISL_2632815, EPI_ISL_2632822	Niedersächsisches Landesgesundheitsamt (NLGA)	Robert Koch Institute	unknown
EPI_ISL_2633408, EPI_ISL_2633420, EPI_ISL_2633452, EPI_ISL_2633457	Laborarztpraxis Osnabrück	Robert Koch Institute	unknown
EPI_ISL_2634518, EPI_ISL_2634543	Eurofins LifeCodexx GmbH	Robert Koch Institute	unknown
EPI_ISL_2635142	Missouri State Public Health Laboratory	Missouri State Public Health Laboratory	Matthew Sinn, Joshua Barry, Ashley New
EPI_ISL_2635450	SYNLAB MVZ Weiden	Robert Koch Institute	unknown
EPI_ISL_2635538	Labor Becker & Kollegen (Standort München)	Robert Koch Institute	unknown
EPI_ISL_2635612	amedes MVZ für Labordiagnostik Rhein-Main	Robert Koch Institute	unknown
EPI_ISL_2635908, EPI_ISL_2635920	Labor Becker & Kollegen (Standort München)	Robert Koch Institute	unknown
EPI_ISL_2636152	Israel Central Virology laboratory	Israel National Consortium for SARS-CoV-2 sequencing	Neta Zuckerman, Efrat Dahan Bucris, Michal Mandelboim, Dana Bar-Ilan, Miranda Geva, Oran Erster, Efrat Glick-Saar, Omri Nayshool, Gideon Rechavi, Ella Mendelson, Orna Mor
EPI_ISL_2636232, EPI_ISL_2636245, EPI_ISL_2636303	Diagnosticum - Labor Neukirchen	Robert Koch Institute	unknown

EPI_ISL_2636305	Israel Central Virology laboratory	Israel National Consortium for SARS-CoV-2 sequencing	Neta Zuckerman, Efrat Dahan Bucris, Michal Mandelboim, Dana Bar-Ilan, Miranda Geva, Oran Erster, Efrat Glick-Saar, Omri Nayshool, Gideon Rechavi, Ella Mendelson, Orna Mor
EPI_ISL_2636320, EPI_ISL_2636327	Diagnosticum - Labor Neukirchen	Robert Koch Institute	unknown
EPI_ISL_2636778	MVZ Labor Krone GbR	Robert Koch Institute	unknown
EPI_ISL_2636891	Laborarztpraxis Osnabrück	Robert Koch Institute	unknown
EPI_ISL_2637364	CLILAB	Microbiology Department	Sara Marti, Aida Gonzalez-Diaz, Laura Calatayud, Jordi Niubó, Miguel Fernandez-Huerta, Carmen Ardanuy, Jordi Camara, M Angeles Domínguez
EPI_ISL_2637398	Hôpital Bichat-Claude Bernard	Department of Virology, Henri Mondor University Hospital, Assistance Publique Hôpitaux de Paris, Université Paris-Est Créteil, INSERM U955	Christophe Rodriguez, Slim Fourati, Vanessa Demontant, Guillaume Gricourt, Melissa N'Debi, Alexandre Soulier, Elisabeth Trawinski, Jean-Michel Pawlotsky
EPI_ISL_2637399	Hôpital Avicenne	Department of Virology, Henri Mondor University Hospital, Assistance Publique Hôpitaux de Paris, Université Paris-Est Créteil, INSERM U955	Christophe Rodriguez, Slim Fourati, Vanessa Demontant, Guillaume Gricourt, Melissa N'Debi, Alexandre Soulier, Elisabeth Trawinski, Jean-Michel Pawlotsky
EPI_ISL_2637437	laboratoire Belle Epine	Department of Virology, Henri Mondor University Hospital, Assistance Publique Hôpitaux de Paris, Université Paris-Est Créteil, INSERM U955	Christophe Rodriguez, Slim Fourati, Vanessa Demontant, Guillaume Gricourt, Melissa N'Debi, Alexandre Soulier, Elisabeth Trawinski, Jean-Michel Pawlotsky
EPI_ISL_2637449	Hôpital Ambroise-Paré	Department of Virology, Henri Mondor University Hospital, Assistance Publique Hôpitaux de Paris, Université Paris-Est Créteil, INSERM U955	Christophe Rodriguez, Slim Fourati, Vanessa Demontant, Guillaume Gricourt, Melissa N'Debi, Alexandre Soulier, Elisabeth Trawinski, Jean-Michel Pawlotsky
EPI_ISL_2637466	laboratoire Belle Epine	Department of Virology, Henri Mondor University Hospital, Assistance Publique Hôpitaux de Paris, Université Paris-Est Créteil, INSERM U955	Christophe Rodriguez, Slim Fourati, Vanessa Demontant, Guillaume Gricourt, Melissa N'Debi, Alexandre Soulier, Elisabeth Trawinski, Jean-Michel Pawlotsky
EPI_ISL_2637467, EPI_ISL_2637468	GROUPE HOSPITALIER SUD ILE DE FRANCE	Department of Virology, Henri Mondor University Hospital, Assistance Publique Hôpitaux de Paris, Université Paris-Est Créteil, INSERM U955	Christophe Rodriguez, Slim Fourati, Vanessa Demontant, Guillaume Gricourt, Melissa N'Debi, Alexandre Soulier, Elisabeth Trawinski, Jean-Michel Pawlotsky
EPI_ISL_2637473, EPI_ISL_2637476, EPI_ISL_2637481	laboratoire Belle Epine	Department of Virology, Henri Mondor University Hospital, Assistance Publique Hôpitaux de Paris, Université Paris-Est Créteil, INSERM U955	Christophe Rodriguez, Slim Fourati, Vanessa Demontant, Guillaume Gricourt, Melissa N'Debi, Alexandre Soulier, Elisabeth Trawinski, Jean-Michel Pawlotsky
EPI_ISL_2637494	Hôpital Cochin	Department of Virology, Henri Mondor University Hospital, Assistance Publique Hôpitaux de Paris, Université Paris-Est Créteil, INSERM U955	Christophe Rodriguez, Slim Fourati, Vanessa Demontant, Guillaume Gricourt, Melissa N'Debi, Alexandre Soulier, Elisabeth Trawinski, Jean-Michel Pawlotsky
EPI_ISL_2637524	laboratoire Belle Epine	Department of Virology, Henri Mondor University Hospital, Assistance Publique Hôpitaux de Paris, Université Paris-Est Créteil, INSERM U955	Christophe Rodriguez, Slim Fourati, Vanessa Demontant, Guillaume Gricourt, Melissa N'Debi, Alexandre Soulier, Elisabeth Trawinski, Jean-Michel Pawlotsky
EPI_ISL_2637532, EPI_ISL_2637567	BIO ARD'AISNE	Department of Virology, Henri Mondor University Hospital, Assistance Publique Hôpitaux de Paris, Université Paris-Est Créteil, INSERM U955	Christophe Rodriguez, Slim Fourati, Vanessa Demontant, Guillaume Gricourt, Melissa N'Debi, Alexandre Soulier, Elisabeth Trawinski, Jean-Michel Pawlotsky
EPI_ISL_2637615	Hôpital Cochin	Department of Virology, Henri Mondor University Hospital, Assistance Publique Hôpitaux de	Christophe Rodriguez, Slim Fourati, Vanessa Demontant, Guillaume Gricourt, Melissa N'Debi, Alexandre Soulier, Elisabeth Trawinski, Jean-Michel Pawlotsky

		Paris, Université Paris-Est Créteil, INSERM U955	
EPI_ISL_2637638	laboratoire Belle Epine	Department of Virology, Henri Mondor University Hospital, Assistance Publique Hôpitaux de Paris, Université Paris-Est Créteil, INSERM U955	Christophe Rodriguez, Slim Fourati, Vanessa Demontant, Guillaume Gricourt, Melissa N'Debi, Alexandre Soulier, Elisabeth Trawinski, Jean-Michel Pawlotsky
EPI_ISL_2637653	BROUSSAIS	Department of Virology, Henri Mondor University Hospital, Assistance Publique Hôpitaux de Paris, Université Paris-Est Créteil, INSERM U955	Christophe Rodriguez, Slim Fourati, Vanessa Demontant, Guillaume Gricourt, Melissa N'Debi, Alexandre Soulier, Elisabeth Trawinski, Jean-Michel Pawlotsky
EPI_ISL_2637668, EPI_ISL_2637683	laboratoire Belle Epine	Department of Virology, Henri Mondor University Hospital, Assistance Publique Hôpitaux de Paris, Université Paris-Est Créteil, INSERM U955	Christophe Rodriguez, Slim Fourati, Vanessa Demontant, Guillaume Gricourt, Melissa N'Debi, Alexandre Soulier, Elisabeth Trawinski, Jean-Michel Pawlotsky
EPI_ISL_2637816	BIOMNIS EUROFINS IVRY	Department of Virology, Henri Mondor University Hospital, Assistance Publique Hôpitaux de Paris, Université Paris-Est Créteil, INSERM U955	Christophe Rodriguez, Slim Fourati, Vanessa Demontant, Guillaume Gricourt, Melissa N'Debi, Alexandre Soulier, Elisabeth Trawinski, Jean-Michel Pawlotsky
EPI_ISL_2637828	CHU Rouen Normandie	Department of Virology, Henri Mondor University Hospital, Assistance Publique Hôpitaux de Paris, Université Paris-Est Créteil, INSERM U955	Christophe Rodriguez, Slim Fourati, Vanessa Demontant, Guillaume Gricourt, Melissa N'Debi, Alexandre Soulier, Elisabeth Trawinski, Jean-Michel Pawlotsky
EPI_ISL_2637858	BIO ARD'AISNE	Department of Virology, Henri Mondor University Hospital, Assistance Publique Hôpitaux de Paris, Université Paris-Est Créteil, INSERM U955	Christophe Rodriguez, Slim Fourati, Vanessa Demontant, Guillaume Gricourt, Melissa N'Debi, Alexandre Soulier, Elisabeth Trawinski, Jean-Michel Pawlotsky
EPI_ISL_2637880	GROUPE HOSPITALIER SUD ILE DE FRANCE	Department of Virology, Henri Mondor University Hospital, Assistance Publique Hôpitaux de Paris, Université Paris-Est Créteil, INSERM U955	Christophe Rodriguez, Slim Fourati, Vanessa Demontant, Guillaume Gricourt, Melissa N'Debi, Alexandre Soulier, Elisabeth Trawinski, Jean-Michel Pawlotsky
EPI_ISL_2637890	BIOMNIS EUROFINS IVRY	Department of Virology, Henri Mondor University Hospital, Assistance Publique Hôpitaux de Paris, Université Paris-Est Créteil, INSERM U955	Christophe Rodriguez, Slim Fourati, Vanessa Demontant, Guillaume Gricourt, Melissa N'Debi, Alexandre Soulier, Elisabeth Trawinski, Jean-Michel Pawlotsky
EPI_ISL_2637926	laboratoire Belle Epine	Department of Virology, Henri Mondor University Hospital, Assistance Publique Hôpitaux de Paris, Université Paris-Est Créteil, INSERM U955	Christophe Rodriguez, Slim Fourati, Vanessa Demontant, Guillaume Gricourt, Melissa N'Debi, Alexandre Soulier, Elisabeth Trawinski, Jean-Michel Pawlotsky
EPI_ISL_2637932	BIO ARD'AISNE	Department of Virology, Henri Mondor University Hospital, Assistance Publique Hôpitaux de Paris, Université Paris-Est Créteil, INSERM U955	Christophe Rodriguez, Slim Fourati, Vanessa Demontant, Guillaume Gricourt, Melissa N'Debi, Alexandre Soulier, Elisabeth Trawinski, Jean-Michel Pawlotsky
EPI_ISL_2637944	BIOMNIS EUROFINS IVRY	Department of Virology, Henri Mondor University Hospital, Assistance Publique Hôpitaux de Paris, Université Paris-Est Créteil, INSERM U955	Christophe Rodriguez, Slim Fourati, Vanessa Demontant, Guillaume Gricourt, Melissa N'Debi, Alexandre Soulier, Elisabeth Trawinski, Jean-Michel Pawlotsky
EPI_ISL_2638017	GROUPE HOSPITALIER SUD ILE DE FRANCE	Department of Virology, Henri Mondor University Hospital, Assistance Publique Hôpitaux de	Christophe Rodriguez, Slim Fourati, Vanessa Demontant, Guillaume Gricourt, Melissa N'Debi, Alexandre Soulier, Elisabeth Trawinski, Jean-Michel Pawlotsky

		Paris, Université Paris-Est Créteil, INSERM U955	
EPI_ISL_2638109	AULSS 2 Marca Trevigiana	Istituto Zooprofilattico Sperimentale delle Venezie	Adelaide Milani, Alessia Schivo, Annalisa Salviato, Elisa Palumbo, Erika Giorgia Quaranta, Luca Tassoni, Ambra Pastori, Edoardo Giussani, Alice Fusaro, Isabella Monne, Calogero Terregino, Antonia Ricci
EPI_ISL_2645092	Fimlab Laboratoriot Oy Tampere	Expert Microbiology, National Institute for Health and Welfare	Soile Blomqvist, Jani Halkilahti, Kirsi Liitsola, Haider al-Hello, Päivi Laurila, Erika Lindh, Teemu Smura, Ravi Kant, Phuoc Truong, Olli Vapalahti, Sari Hannula, Pekka Ellonen, Niina Ikonen, Carita Savolainen-Kopra
EPI_ISL_2645124	Islab, Pohjois-Savon aluelaboratorio	Expert Microbiology, National Institute for Health and Welfare	Soile Blomqvist, Jani Halkilahti, Kirsi Liitsola, Haider al-Hello, Päivi Laurila, Erika Lindh, Teemu Smura, Ravi Kant, Phuoc Truong, Olli Vapalahti, Sari Hannula, Pekka Ellonen, Niina Ikonen, Carita Savolainen-Kopra
EPI_ISL_2645201	Fimlab Laboratoriot Oy Tampere	Expert Microbiology, National Institute for Health and Welfare	Soile Blomqvist, Jani Halkilahti, Kirsi Liitsola, Haider al-Hello, Päivi Laurila, Erika Lindh, Teemu Smura, Ravi Kant, Phuoc Truong, Olli Vapalahti, Sari Hannula, Pekka Ellonen, Niina Ikonen, Carita Savolainen-Kopra
EPI_ISL_2646145	CH CALAIS	CHU Lille - Laboratoire de Virologie	AIT YAHYA Emilie, ALIDJINOU Enagnon Kazali, BOCKET Laurence, CREPIN Michel, DEMAY Christophe, ENGELMANN Ilka, GEFFROY Sandrine, GUIGON Aurélie, LAMBERT Valérie, LAZREK Mouna, NOBILLIAUX Florian, PREVOST Brigitte, THUILLIER Caroline, TINEZ Claire ,TCHANTCHOU NJOSSE YANICK
EPI_ISL_2646175	CH BETHUNE	CHU Lille - Laboratoire de Virologie	AIT YAHYA Emilie, ALIDJINOU Enagnon Kazali, BOCKET Laurence, CREPIN Michel, DEMAY Christophe, ENGELMANN Ilka, GEFFROY Sandrine, GUIGON Aurélie, LAMBERT Valérie, LAZREK Mouna, NOBILLIAUX Florian, PREVOST Brigitte, THUILLIER Caroline, TINEZ Claire ,TCHANTCHOU NJOSSE YANICK
EPI_ISL_2646195	CH ROUBAIX	CHU Lille - Laboratoire de Virologie	AIT YAHYA Emilie, ALIDJINOU Enagnon Kazali, BOCKET Laurence, CREPIN Michel, DEMAY Christophe, ENGELMANN Ilka, GEFFROY Sandrine, GUIGON Aurélie, LAMBERT Valérie, LAZREK Mouna, NOBILLIAUX Florian, PREVOST Brigitte, THUILLIER Caroline, TINEZ Claire ,TCHANTCHOU NJOSSE YANICK
EPI_ISL_2646285	CHU de la Réunion - Sud	Laboratoire de virologie, CNR arbovirus Associé, Chu de la Réunion	Etienne Frumence, Marie-Christine Jaffar Bandjee, Nicolas Traversier, Sabrina Petit Genet
EPI_ISL_2646288	CHU de la Réunion - Nord	Laboratoire de virologie, CNR arbovirus Associé, Chu de la Réunion	Etienne Frumence, Marie-Christine Jaffar Bandjee, Nicolas Traversier, Sabrina Petit Genet
EPI_ISL_2646289, EPI_ISL_2646292, EPI_ISL_2646293, EPI_ISL_2646297	CHU de la Réunion - Sud	Laboratoire de virologie, CNR arbovirus Associé, Chu de la Réunion	Etienne Frumence, Marie-Christine Jaffar Bandjee, Nicolas Traversier, Sabrina Petit Genet
EPI_ISL_2646298	CHU de la Réunion - Nord	Laboratoire de virologie, CNR arbovirus Associé, Chu de la Réunion	Etienne Frumence, Marie-Christine Jaffar Bandjee, Nicolas Traversier, Sabrina Petit Genet
EPI_ISL_2647311	Mako Medical	Centers for Disease Control and Prevention Division of Viral Diseases, Pathogen Discovery	Dakota Howard, Dhwani Batra, Peter W. Cook, Kara Moser, Adrian Paskey, Jason Caravas, Benjamin Rambo-Martin, Shatavia Morrison, Christopher Gulvick, Scott Sammons, Yvette Unoarumhi, Darlene Wagner, Matthew Schmerer,Matthew Tugwell, Lauren Moon, Clinton R. Paden, Duncan MacCannell
EPI_ISL_2648206	Debswana Orapa Mine Hospital Laboratory	Botswana Harvard HIV Reference Laboratory	Sikhulile Moyo, Wonderful T. Choga, Dorcas Maruapula, Keoratile Ntshambiwa, Lesedi Magama, Mpo Molapisi, Koketso Maotwe, Thongbotho Mphoyakgosi, Boitumelo Zuze, Botshelo Radibe, Legodile Kooepile, Ontlametse T. Bareng, Roger Shapiro, Shahin Lockman, Joseph Makhema, Madisa Mine, Mosepele Mosepele, Simani Gaseitsiwe
EPI_ISL_2648239	Kasane Primary Hospital Laboratory	Botswana Harvard HIV Reference Laboratory	Sikhulile Moyo, Wonderful T. Choga, Dorcas Maruapula, Agnes Karutwaeng, Rose Munyere, Keoratile Ntshambiwa, Thongbotho Mphoyakgosi, Boitumelo Zuze, Botshelo Radibe, Legodile Kooepile, Ontlametse T. Bareng, Roger Shapiro, Shahin Lockman, Joseph Makhema, Madisa Mine, Mosepele Mosepele, Simani Gaseitsiwe
EPI_ISL_2649991	Kudat Hospital	Institute for Medical Research, Infectious Disease Research Centre, National Institutes of Health, Ministry of Health Malaysia	Suppiah J, Kamel K, Mohd Zawawi Z, Azizan MA, Ramly N, Robert F, Thayan R
EPI_ISL_2649994	Tuanku Fauziah Hopsital	Institute for Medical Research, Infectious Disease Research Centre, National Institutes of Health, Ministry of Health Malaysia	Suppiah J, Kamel K, Mohd Zawawi Z, Azizan MA, Ramly N, Robert F, Thayan R
EPI_ISL_2649998, EPI_ISL_2649999	Penang General Hospital	Institute for Medical Research, Infectious Disease Research Centre, National Institutes of Health, Ministry of Health Malaysia	Suppiah J, Kamel K, Mohd Zawawi Z, Azizan MA, Ramly N, Robert F, Thayan R
EPI_ISL_2650000	Sultanah Nur Zahirah Hospital	Institute for Medical Research, Infectious Disease Research Centre, National Institutes of Health, Ministry of Health Malaysia	Suppiah J, Kamel K, Mohd Zawawi Z, Azizan MA, Ramly N, Robert F, Thayan R

EPI_ISL_2650001	Hospital Sultanah Nur Zahirah Kuala Terengganu	Institute for Medical Research, Infectious Disease Research Centre, National Institutes of Health, Ministry of Health Malaysia	Suppiah J, Kamel K, Mohd Zawawi Z, Azizan MA, Ramly N, Robert F, Thayan R
EPI_ISL_2650004, EPI_ISL_2650005	Sungai Buloh Hospital	Institute for Medical Research, Infectious Disease Research Centre, National Institutes of Health, Ministry of Health Malaysia	Suppiah J, Kamel K, Mohd Zawawi Z, Azizan MA, Ramly N, Robert F, Thayan R
EPI_ISL_2650006	Tumpat Hospital	Institute for Medical Research, Infectious Disease Research Centre, National Institutes of Health, Ministry of Health Malaysia	Suppiah J, Kamel K, Mohd Zawawi Z, Azizan MA, Ramly N, Robert F, Thayan R
EPI_ISL_2650007	Ipoh Public Health Laboratory (MKAI), Ministry of Health Malaysia	Institute for Medical Research, Infectious Disease Research Centre, National Institutes of Health, Ministry of Health Malaysia	Suppiah J, Kamel K, Mohd Zawawi Z, Azizan MA, Ramly N, Robert F, Thayan R
EPI_ISL_2650010	Sungai Buloh Hospital	Institute for Medical Research, Infectious Disease Research Centre, National Institutes of Health, Ministry of Health Malaysia	Suppiah J, Kamel K, Mohd Zawawi Z, Azizan MA, Ramly N, Robert F, Thayan R
EPI_ISL_2650028	Sultanah Aminah Hospital	Institute for Medical Research, Infectious Disease Research Centre, National Institutes of Health, Ministry of Health Malaysia	Suppiah J, Kamel K, Mohd Zawawi Z, Azizan MA, Ramly N, Robert F, Thayan R
EPI_ISL_2650250	Microbiology Department, Laboratori Clínic Metropolitana Nord. Hospital Universitari Germans Trias i Pujol	Can Ruti SARS-CoV-2 Sequencing Hub (HUGTiP/IrsiCaixa/IGTP)	Marc Noguera-Julian, Pilar Armengol, Ignacio Blanco, Antoni E Bordoy, Francesc Catala-Moll, Pere-Joan Cardona, Maria Casadellà, Cristina Casañ, Gemma Clara, Bonaventura Clotet, Cristina Esteban, Montserrat Giménez, Mercedes Guerrero, Anna Not, Roger Paredes, Mariona Parera, Verónica Saludes, Alba Sánchez, and Elisa Martró on behalf of the Can Ruti SARS-CoV-2 Sequencing Hub
EPI_ISL_2650259	Microbiologia CATLAB	Can Ruti SARS-CoV-2 Sequencing Hub (HUGTiP/IrsiCaixa/IGTP)	Marc Noguera-Julian, Pilar Armengol, Ignacio Blanco, Antoni E Bordoy, Francesc Catala-Moll, Pere-Joan Cardona, Maria Casadellà, Cristina Casañ, Gemma Clara, Bonaventura Clotet, Cristina Esteban, Montserrat Giménez, Mercedes Guerrero, Anna Not, Roger Paredes, Mariona Parera, Verónica Saludes, Alba Sánchez, and Elisa Martró on behalf of the Can Ruti SARS-CoV-2 Sequencing Hub
EPI_ISL_2650430	Queen Elizabeth Hospital	Institute for Medical Research, Infectious Disease Research Centre, National Institutes of Health, Ministry of Health Malaysia	Suppiah J, Kamel K, Mohd Zawawi Z, Azizan MA, Ramly N, Robert F, Thayan R
EPI_ISL_2650468	Sydney South West Pathology Service (SSWPS) - Royal Prince Alfred Hospital - NSW Health Pathology	NSW Health Pathology - Institute of Clinical Pathology and Medical Research; Westmead Hospital; University of Sydney	CIDM-PH et al.
EPI_ISL_2650499, EPI_ISL_2650505	Queen Elizabeth Hospital	Institute for Medical Research, Infectious Disease Research Centre, National Institutes of Health, Ministry of Health Malaysia	Suppiah J, Kamel K, Mohd Zawawi Z, Azizan MA, Ramly N, Robert F, Thayan R
EPI_ISL_2651123	Temporary Specimen Collection Centre at the AsiaWorld-Expo	Hong Kong Department of Health	Alan K.L. Tsang, Peter C.W. Yip, Ken H.L. Ng, Edman T.K. Lam, Rickjason C.W. Chan, Dominic N.C. Tsang
EPI_ISL_2651266, EPI_ISL_2651267	CHU de la Réunion - Nord	Laboratoire de virologie, CNR arbovirus Associé, Chu de la Réunion	Etienne Frumence, Marie-Christine Jaffar Bandjee, Nicolas Traversier, Sabrina Petit Genet
EPI_ISL_2651270, EPI_ISL_2651274, EPI_ISL_2651275, EPI_ISL_2651276	CHU de la Réunion - Sud	Laboratoire de virologie, CNR arbovirus Associé, Chu de la Réunion	Etienne Frumence, Marie-Christine Jaffar Bandjee, Nicolas Traversier, Sabrina Petit Genet
EPI_ISL_2651279	CHU de la Réunion - Nord	Laboratoire de virologie, CNR arbovirus Associé,	Etienne Frumence, Marie-Christine Jaffar Bandjee, Nicolas Traversier, Sabrina Petit Genet

		Chu de la Réunion	
EPI_ISL_2651365	CHU de la Réunion - Sud	Laboratoire de virologie, CNR arbovirus Associé, Chu de la Réunion	Etienne Frumence, Marie-Christine Jaffar Bandjee, Nicolas Traversier, Sabrina Petit Genet
EPI_ISL_2651367, EPI_ISL_2651368	CHU de la Réunion - Nord	Laboratoire de virologie, CNR arbovirus Associé, Chu de la Réunion	Etienne Frumence, Marie-Christine Jaffar Bandjee, Nicolas Traversier, Sabrina Petit Genet
EPI_ISL_2651370, EPI_ISL_2651372, EPI_ISL_2651373, EPI_ISL_2651374, EPI_ISL_2651378, EPI_ISL_2651380, EPI_ISL_2651381, EPI_ISL_2651382	CHU de la Réunion - Sud	Laboratoire de virologie, CNR arbovirus Associé, Chu de la Réunion	Etienne Frumence, Marie-Christine Jaffar Bandjee, Nicolas Traversier, Sabrina Petit Genet
EPI_ISL_2651386, EPI_ISL_2651388	CHU de la Réunion - Nord	Laboratoire de virologie, CNR arbovirus Associé, Chu de la Réunion	Etienne Frumence, Marie-Christine Jaffar Bandjee, Nicolas Traversier, Sabrina Petit Genet
EPI_ISL_2651391	CHU de la Réunion - Sud	Laboratoire de virologie, CNR arbovirus Associé, Chu de la Réunion	Etienne Frumence, Marie-Christine Jaffar Bandjee, Nicolas Traversier, Sabrina Petit Genet
EPI_ISL_2651395, EPI_ISL_2651396	CHU de la Réunion - Nord	Laboratoire de virologie, CNR arbovirus Associé, Chu de la Réunion	Etienne Frumence, Marie-Christine Jaffar Bandjee, Nicolas Traversier, Sabrina Petit Genet
EPI_ISL_2651404, EPI_ISL_2651405	CHU de la Réunion - Sud	Laboratoire de virologie, CNR arbovirus Associé, Chu de la Réunion	Etienne Frumence, Marie-Christine Jaffar Bandjee, Nicolas Traversier, Sabrina Petit Genet
EPI_ISL_2652536, EPI_ISL_2652577, EPI_ISL_2652723, EPI_ISL_2652753, EPI_ISL_2652755, EPI_ISL_2652798, EPI_ISL_2652920	Infinity Biologix	Centers for Disease Control and Prevention Division of Viral Diseases, Pathogen Discovery	Dakota Howard, Dhwani Batra, Peter W. Cook, Kara Moser, Adrian Paskey, Jason Caravas, Benjamin Rambo-Martin, Shatavia Morrison, Christopher Gulvick, Scott Sammons, Yvette Unoarumhi, Darlene Wagner, Matthew Schmerer, Christian Bixby, Yihe Wang, Jonathan Schultz, Chirayu Goswami, Russ Hager, Robin Grimwood, Clinton R. Paden, Duncan MacCannell
EPI_ISL_2657919	Public Health Authority of the Slovak Republic	Laboratory of Genomics and Bioinformatics, Comenius University Science Park	Tatiana Sedláková, Diana Rusáková, Miroslav Böhmer, Anna Giová, Jaroslav Budiš, Tomáš Szemes, Jakub Styk
EPI_ISL_2658517	Minnesota Department of Health, Public Health Laboratory	Minnesota Department of Health, Public Health Laboratory	Alexandra Lorentz, Jacob Garfin, Matt Plumb, and Xiong Wang
EPI_ISL_2662306, EPI_ISL_2662429, EPI_ISL_2662437	Viollier AG	Department of Biosystems Science and Engineering, ETH Zürich	Andrea Patrizia Salzmann, Henriette Kurth, Christiane Beckmann, Ivan Topolsky, Chaoran Chen, Sarah Nadeau, Kim Philipp Jablonski, Lara Fuhrmann, Niko Beerenwinkel, Christoph Noppen, Olivier Kobel, Maurice Redondo, Tanja Stadler
EPI_ISL_2662570, EPI_ISL_2662572, EPI_ISL_2662575, EPI_ISL_2662577, EPI_ISL_2662579, EPI_ISL_2662580, EPI_ISL_2662584, EPI_ISL_2662587, EPI_ISL_2662601, EPI_ISL_2662602, EPI_ISL_2662612			
see above	TAMBO MEMORIAL LABORATORY	National Institute for Communicable Diseases of the National Health Laboratory Service	Amoako DG, Everatt J, Scheepers C, Mohale T, Ntuli N, Mahlangu B, Mnguni A, Ismail A, Bhiman JN
EPI_ISL_2662620, EPI_ISL_2662638, EPI_ISL_2662644, EPI_ISL_2662645, EPI_ISL_2662656	CHRIS HANI BARAGWANATH LABORATORY	National Institute for Communicable Diseases of the National Health Laboratory Service	Amoako DG, Everatt J, Scheepers C, Mohale T, Ntuli N, Mahlangu B, Mnguni A, Ismail A, Bhiman JN
EPI_ISL_2662664	KOPANONG LABORATORY	National Institute for Communicable Diseases of the National Health Laboratory Service	Amoako DG, Everatt J, Scheepers C, Mohale T, Ntuli N, Mahlangu B, Mnguni A, Ismail A, Bhiman JN
EPI_ISL_2662710, EPI_ISL_2662712, EPI_ISL_2662714, EPI_ISL_2662716, EPI_ISL_2662723, EPI_ISL_2662725	National Institute for Communicable Diseases of the National Health Laboratory Service	National Institute for Communicable Diseases of the National Health Laboratory Service	Amoako DG, Everatt J, Scheepers C, Mohale T, Ntuli N, Mahlangu B, Mnguni A, Ismail A, Bhiman JN
EPI_ISL_2665942, EPI_ISL_2670086	Department of Bacteria, Parasites and Fungi, Statens Serum Institut, Copenhagen, Denmark	Statens Serum Institut Bioinformatics and Microbial Genomics	Danish Covid-19 Genome Consortium
EPI_ISL_2674251	National Institute of Public Health	State Veterinary Institute Prague	Nagy,A;Cernikov,L;Stara,M;Suri,T;Vecerova,J;Jirincova,H
EPI_ISL_2674806	SARS-CoV-2 testing team, National Institute of Infectious Diseases	Pathogen Genomics Center, National Institute of Infectious Diseases	Tsuyoshi Sekizuka, Kentaro Itokawa, Rina Tanaka, Masanori Hashino, Nozomu Hanaoka, Masumichi Saito, Naomi Nojiri, Hazuka Y Furihata, Hiromizu Takahashi, Tsuguto Fujimoto, Makoto Kuroda
EPI_ISL_2676579	LABORATOIRE DE BIOLOGIE MEDICALE	CNR Virus des Infections Respiratoires - France SUD	Antonin Bal, Gregory Destras, Gwendolyne Burfin, Hadrien Regue, Quentin Semanas, Martine Valette, Bruno Lina, Laurence Josset
EPI_ISL_2676654, EPI_ISL_2676657, EPI_ISL_2676667, EPI_ISL_2676668, EPI_ISL_2676671	REUNILAB	CNR Virus des Infections Respiratoires - France SUD	Antonin Bal, Gregory Destras, Gwendolyne Burfin, Hadrien Regue, Quentin Semanas, Martine Valette, Bruno Lina, Laurence Josset

EPI_ISL_2676706, EPI_ISL_2676708	ASTRALAB TASSIGNY	CNR Virus des Infections Respiratoires - France SUD	Antonin Bal, Gregory Destras, Gwendolyne Burfin, Hadrien Regue, Quentin Semanas, Martine Valette, Bruno Lina, Laurence Josset
EPI_ISL_2676713	CHR LA REUNION FELIX GUYON	CNR Virus des Infections Respiratoires - France SUD	Antonin Bal, Gregory Destras, Gwendolyne Burfin, Hadrien Regue, Quentin Semanas, Martine Valette, Bruno Lina, Laurence Josset
EPI_ISL_2676721, EPI_ISL_2676725	LAM DE SAINT BENOIT	CNR Virus des Infections Respiratoires - France SUD	Antonin Bal, Gregory Destras, Gwendolyne Burfin, Hadrien Regue, Quentin Semanas, Martine Valette, Bruno Lina, Laurence Josset
EPI_ISL_2676741	LBM UNIBIO VALENTIN	CNR Virus des Infections Respiratoires - France SUD	Antonin Bal, Gregory Destras, Gwendolyne Burfin, Hadrien Regue, Quentin Semanas, Martine Valette, Bruno Lina, Laurence Josset
EPI_ISL_2678111, EPI_ISL_2678112	Office of Diseases Prevention and Control Region 12 Songkhla	COVID-19 Network Investigations (CONI) Alliance	Elizabeth Batty, Wasun Chantratita, Thanat Chookajorn, Stefan Fernandez, Angkana Huang, Anthony R. Jones, Khajohn Joonlasak, Chonticha Klungtong, Theerarat Kochakarn, Namfon Kotanan, Krittikorn Kumpornsin, Duangkamon Loesbanluechai, Wudtichai Manasatienkij, Bhakbhoom Panthan, Ekawat Pasomsub, Kingkan Rakmanee, Insee Sensorn, Janjira Thaipadungpanit, Arporn Wangwiwatsin, Treewat Watthanachockchai, Wiphaporn Thabthimthong, Sawanya Chantutanon
EPI_ISL_2678988, EPI_ISL_2679374, EPI_ISL_2679376, EPI_ISL_2679441, EPI_ISL_2679473, EPI_ISL_2679475	Utah Public Health Laboratory	Utah Public Health Laboratory	Erin L. Young, Kelly F. Oakeson, Tara Gallagher
EPI_ISL_2684231, EPI_ISL_2684232, EPI_ISL_2684233	Institute for Medical Research, Infectious Disease Research Centre, National Institutes of Health, Ministry of Health Malaysia	Institute for Medical Research, Infectious Disease Research Centre, National Institutes of Health, Ministry of Health Malaysia	Suppiah J, Kamel K, Mohd Zawawi Z, Azizan MA, Ramly N, Robert F, Thayan R
EPI_ISL_2684234, EPI_ISL_2684235	Melaka Hospital	Institute for Medical Research, Infectious Disease Research Centre, National Institutes of Health, Ministry of Health Malaysia	Suppiah J, Kamel K, Mohd Zawawi Z, Azizan MA, Ramly N, Robert F, Thayan R
EPI_ISL_2684236	Sultanah Bahiyah Hospital, Alor Setar	Institute for Medical Research, Infectious Disease Research Centre, National Institutes of Health, Ministry of Health Malaysia	Suppiah J, Kamel K, Mohd Zawawi Z, Azizan MA, Ramly N, Robert F, Thayan R
EPI_ISL_2684545	Institute for Medical Research, Infectious Disease Research Centre, National Institutes of Health, Ministry of Health Malaysia	Institute for Medical Research, Infectious Disease Research Centre, National Institutes of Health, Ministry of Health Malaysia	Suppiah J, Kamel K, Mohd Zawawi Z, Azizan MA, Ramly N, Robert F, Thayan R
EPI_ISL_2684686	Statens Serum Institut Bioinformatics and Microbial Genomics	Statens Serum Institut Bioinformatics and Microbial Genomics	Danish Covid-19 Genome Consortium
EPI_ISL_2685359, EPI_ISL_2685766, EPI_ISL_2685835, EPI_ISL_2685836, EPI_ISL_2685841, EPI_ISL_2685843, EPI_ISL_2685844, EPI_ISL_2685845	Institute for Medical Research, Infectious Disease Research Centre, National Institutes of Health, Ministry of Health Malaysia	Institute for Medical Research, Infectious Disease Research Centre, National Institutes of Health, Ministry of Health Malaysia	Suppiah J, Kamel K, Mohd Zawawi Z, Azizan MA, Ramly N, Robert F, Thayan R
EPI_ISL_2686278, EPI_ISL_2686279	National Institute of Public Health	National Institute of Public Health	Helena Jirincova, Jaromira Vecerova, Timotej Suri, Dusan Trnka, Alexander Nagy
EPI_ISL_2687328	Aegis Sciences Corporation	Centers for Disease Control and Prevention Division of Viral Diseases, Pathogen Discovery	Dakota Howard, Dhwani Batra, Peter W. Cook, Kara Moser, Adrian Paskey, Jason Caravas, Benjamin Rambo-Martin, Shatavia Morrison, Christopher Gulvick, Scott Sammons, Yvette Unoarumhi, Darlene Wagner, Matthew Schmerer, Cyndi Clark, Patrick Campbell, Rob Case, Vikramsinha Ghorpade, Holly Houdeshell, Ola Kvalvaag, Dillon Nall, Ethan Sanders, Alec Vest, Shaun Westlund, Matthew Hardison, Clinton R. Paden, Duncan MacCannell
EPI_ISL_2687946	National Platform bis UMONS/Jolimont	National Platform bis UMONS/Jolimont	François Dufrasne, Guillaume Bayon-Vicente, Florian Juszczak, Gautier Detry, Ruddy Wattiez
EPI_ISL_2688532, EPI_ISL_2688534, EPI_ISL_2688537	National Health Laboratory Service, South Africa	KRISP, KZn Research Innovation and Sequencing Platform	Giandhari Jennifer, Pillay Sureshnee, Yajna Ramphal, Naidoo Yeshnee, Tshabuila Derek,Tegally Houriiyah, San James, Wilkinson Eduan, de Oliveira Tulio
EPI_ISL_2688547, EPI_ISL_2688548	KRISP, KZn Research Innovation and Sequencing Platform	KRISP, KZn Research Innovation and Sequencing Platform	Giandhari Jennifer, Pillay Sureshnee, Yajna Ramphal, Naidoo Yeshnee, Tshabuila Derek,Tegally Houriiyah, San James, Wilkinson Eduan, de Oliveira Tulio
EPI_ISL_2688549, EPI_ISL_2688557, EPI_ISL_2688560,	National Health Laboratory Service, South Africa	KRISP, KZn Research Innovation and Sequencing Platform	Giandhari Jennifer, Pillay Sureshnee, Yajna Ramphal, Naidoo Yeshnee, Tshabuila Derek,Tegally Houriiyah, San James, Wilkinson Eduan, de Oliveira Tulio

EPI_ISL_2688561, EPI_ISL_2688562, EPI_ISL_2688568, EPI_ISL_2688570, EPI_ISL_2688571, EPI_ISL_2688572, EPI_ISL_2688574			
EPI_ISL_2689595, EPI_ISL_2689597	GA Department of Public Health Laboratory	Genomics and Discovery, Respiratory Viruses Branch, Division of Viral Diseases, Centers for Disease Control and Prevention	Jing Zhang, Anna Kelleher, Ying Tao, Yan Li, Brian Lynch, Krista Queen, Anna Uehara, Peter Cook, Han Jia Justin Ng, Rachel Marine, Clinton R. Paden, Dhwani Batra, Haibin Wang, Tara Coalter, Jasmine Padilla, Morgan Davis, Mili Sheth, Sarah Nobles, Mark Burroughs, Justin Lee, Adam Retchless, Suxiang Tong
EPI_ISL_2689731	Servicio de Microbiologia Hospital Ramon y Cajal	Servicio de Microbiologia Hospital Ramon y Cajal	Ponce M, Galan JC, Martinez L. Abreu M, y Gonzalez-Alba JM
EPI_ISL_2690056	KS Health and Environmental Laboratories	Centers for Disease Control and Prevention Division of Viral Diseases, Pathogen Discovery	Mili Sheth, Sarah Nobles, Jasmine Padilla, Mark Burroughs, Shoshona Le, Katie Dillon, Peter Cook, Clinton R. Paden, Dhwani Batra, Krista Queen, Kristen Knipe, Dakota Howard, Yvette Unoarumhi, Darlene Wagner, Matthew Schmerer, Ben L. Rambo-Martin, Kristine Lacek, Sam Shepard, Alison Laufer Halpin, Dave Wentworth, Vivien Dugan, Suxiang Tong, Justin Lee
EPI_ISL_2691928	Rush University Medical Center	RIPHL at Rush University Medical Center	Stefan Green, Kevin Kunstman, Max Kolton, Marieta Hyde, Laura Furtado, Felix Araujo Perez, Mary Hayden, Joyce Houlihan, Diane Springer
EPI_ISL_2693028	Oregon State Public Health Laboratory	Oregon State Public Health Laboratory	Rafia Razzaque, Eugene Yeboah, Vanda Makris, Laura Tsaknaridis, John Fontana and Shane Sevey
EPI_ISL_2693075	NHLS_VIRO	KRISP, KZN Research Innovation and Sequencing Platform	Giandhari Jennifer, Pillay Sureshnee, Yajna Ramphal, Naidoo Yeshnee, Tshabuila Derek,Tegally Houriiyah, San James, Wilkinson Eduan, de Oliveira Tulio
EPI_ISL_2693321	Swellendam PHC Clinic wc SHC	NHLS/UCT	Arash Iranzadeh, Deelan Doolabh, Lynn Tyers, Bruna Galvao, Innocent Mudau, Marvin Hsiao, Gert Marais, Diana Hardie, Stephen Korsman, Carolyn Williamson
EPI_ISL_2693324, EPI_ISL_2693325, EPI_ISL_2693327	Groote Schuur Hospital wc GSH	NHLS/UCT	Arash Iranzadeh, Deelan Doolabh, Lynn Tyers, Bruna Galvao, Innocent Mudau, Marvin Hsiao, Gert Marais, Diana Hardie, Stephen Korsman, Carolyn Williamson
EPI_ISL_2693330, EPI_ISL_2693332	NHLS/UCT	NHLS/UCT	Arash Iranzadeh, Deelan Doolabh, Lynn Tyers, Bruna Galvao, Innocent Mudau, Marvin Hsiao, Gert Marais, Diana Hardie, Stephen Korsman, Carolyn Williamson
EPI_ISL_2693736	UW Virology Lab	UW Virology Lab	Pavitra Roychoudhury, Hong Xie, Lasata Shrestha, Shah Mohamed Bakhash, Tien V. Nguyen, Noah R. Baker, Sean Ellis, Meei-Li Huang, Keith R Jerome, Alexander Greninger
EPI_ISL_2693857	NHLS_VIRO Eastern Cape	KRISP, KZN Research Innovation and Sequencing Platform	Giandhari Jennifer, Pillay Sureshnee, Yajna Ramphal, Naidoo Yeshnee, Tshabuila Derek,Tegally Houriiyah, San James, Wilkinson Eduan, de Oliveira Tulio
EPI_ISL_2694612	TAMBO MEMORIAL	National Institute for Communicable Diseases of the National Health Laboratory Service	Amoako DG, Everatt J, Scheepers C, Mohale T, Ntuli N, Mahlangu B, Mnguni A, Ismail A, Bhiman JN
EPI_ISL_2694614, EPI_ISL_2694615	KOPANONG	National Institute for Communicable Diseases of the National Health Laboratory Service	Amoako DG, Everatt J, Scheepers C, Mohale T, Ntuli N, Mahlangu B, Mnguni A, Ismail A, Bhiman JN
EPI_ISL_2694618, EPI_ISL_2694620, EPI_ISL_2694625	TAMBO MEMORIAL	National Institute for Communicable Diseases of the National Health Laboratory Service	Amoako DG, Everatt J, Scheepers C, Mohale T, Ntuli N, Mahlangu B, Mnguni A, Ismail A, Bhiman JN
EPI_ISL_2694629, EPI_ISL_2694630, EPI_ISL_2694631, EPI_ISL_2694633, EPI_ISL_2694635, EPI_ISL_2694647, EPI_ISL_2694656, EPI_ISL_2694660, EPI_ISL_2694673, EPI_ISL_2694674, EPI_ISL_2694675, EPI_ISL_2694676			
see above	CHRIS HANI BARAGWANATH	National Institute for Communicable Diseases of the National Health Laboratory Service	Amoako DG, Everatt J, Scheepers C, Mohale T, Ntuli N, Mahlangu B, Mnguni A, Ismail A, Bhiman JN
EPI_ISL_2694682, EPI_ISL_2694695, EPI_ISL_2694703, EPI_ISL_2694707, EPI_ISL_2694712, EPI_ISL_2694717, EPI_ISL_2694720, EPI_ISL_2694721, EPI_ISL_2694722, EPI_ISL_2695504	TAMBO MEMORIAL	National Institute for Communicable Diseases of the National Health Laboratory Service	Amoako DG, Everatt J, Scheepers C, Mohale T, Ntuli N, Mahlangu B, Mnguni A, Ismail A, Bhiman JN
EPI_ISL_2695506	Bertha gxowa hospital	National Institute for Communicable Diseases of the National Health Laboratory Service	Amoako DG, Everatt J, Scheepers C, Mohale T, Ntuli N, Mahlangu B, Mnguni A, Ismail A, Bhiman JN
EPI_ISL_2695509, EPI_ISL_2695511, EPI_ISL_2695528	TAMBO MEMORIAL	National Institute for Communicable Diseases of the National Health Laboratory Service	Amoako DG, Everatt J, Scheepers C, Mohale T, Ntuli N, Mahlangu B, Mnguni A, Ismail A, Bhiman JN
EPI_ISL_2695541, EPI_ISL_2695542, EPI_ISL_2695544, EPI_ISL_2695545, EPI_ISL_2695546, EPI_ISL_2695547, EPI_ISL_2695552, EPI_ISL_2695553, EPI_ISL_2695554, EPI_ISL_2695558, EPI_ISL_2695559, EPI_ISL_2695560, EPI_ISL_2695563, EPI_ISL_2695567, EPI_ISL_2695568, EPI_ISL_2695571, EPI_ISL_2695575, EPI_ISL_2695577, EPI_ISL_2695582			
see above	POLOKWANE MANKWENG HOSPITAL	National Institute for Communicable Diseases	Amoako DG, Everatt J, Scheepers C, Mohale T, Ntuli N, Mahlangu B, Mnguni A, Ismail A, Bhiman JN

		of the National Health Laboratory Service	
EPI_ISL_2695584, EPI_ISL_2695585	PATHCARE LABORATORY	National Institute for Communicable Diseases of the National Health Laboratory Service	Amoako DG, Everatt J, Scheepers C, Mohale T, Ntuli N, Mahlangu B, Mnguni A, Ismail A, Bhiman JN
EPI_ISL_2695590, EPI_ISL_2695606	HELEN JOSEPH	National Institute for Communicable Diseases of the National Health Laboratory Service	Amoako DG, Everatt J, Scheepers C, Mohale T, Ntuli N, Mahlangu B, Mnguni A, Ismail A, Bhiman JN
EPI_ISL_2695608, EPI_ISL_2695609	ROB FERREIRA LABORATORY	National Institute for Communicable Diseases of the National Health Laboratory Service	Amoako DG, Everatt J, Scheepers C, Mohale T, Ntuli N, Mahlangu B, Mnguni A, Ismail A, Bhiman JN
EPI_ISL_2695732	HELEN JOSEPH	National Institute for Communicable Diseases of the National Health Laboratory Service	Amoako DG, Everatt J, Scheepers C, Mohale T, Ntuli N, Mahlangu B, Mnguni A, Ismail A, Bhiman JN
EPI_ISL_2695760, EPI_ISL_2695761	KOPANONG LABORATORY	National Institute for Communicable Diseases of the National Health Laboratory Service	Amoako DG, Everatt J, Scheepers C, Mohale T, Ntuli N, Mahlangu B, Mnguni A, Ismail A, Bhiman JN
EPI_ISL_2695811, EPI_ISL_2695812, EPI_ISL_2695813, EPI_ISL_2695814, EPI_ISL_2695816, EPI_ISL_2695824, EPI_ISL_2695826, EPI_ISL_2695827, EPI_ISL_2695830, EPI_ISL_2695831, EPI_ISL_2695832, EPI_ISL_2695833, EPI_ISL_2695838, EPI_ISL_2695839, EPI_ISL_2695840, EPI_ISL_2695848			
see above	PathCare, Cape Town	Division of Medical Virology, National Health Laboratory Service (NHLS), Tygerberg Hospital / Stellenbosch University	Susan Engelbrecht, Tongai Maponga, Bronwyn Kleinhans, Tania Stander, Jean Maritz, Petra Raimond, San Emmanual James, Tulio de Oliveira, Gert van Zyl, Wolfgang Preiser
EPI_ISL_2695850, EPI_ISL_2695858, EPI_ISL_2695863, EPI_ISL_2695867	Division of Medical Virology, National Health Laboratory Service (NHLS), Tygerberg Hospital / Stellenbosch University	Division of Medical Virology, National Health Laboratory Service (NHLS), Tygerberg Hospital / Stellenbosch University	Susan Engelbrecht, Tongai Maponga, Bronwyn Kleinhans, Tania Stander, Gert van Zyl, San Emmanual James, Tulio de Oliveira, Wolfgang Preiser
EPI_ISL_2709261	Labo Analyses Med	National Reference Center for Viruses of Respiratory Infections, Institut Pasteur, Paris	Marion Barbet, Sylvie Behillil, Méline Bizard, Angela Brisebarre, Camille Capel, Vincent Enouf, Louise Lefrançois, Frédéric Lemoine, Christophe Malabat, Corinne Maufrais, Etienne Simon-Lorière, Maud Vanpeene, Sylvie Van der Werf ,Patricia Tamby
EPI_ISL_2709270	Labo Analyses Med	National Reference Center for Viruses of Respiratory Infections, Institut Pasteur, Paris	Marion Barbet, Sylvie Behillil, Méline Bizard, Angela Brisebarre, Camille Capel, Vincent Enouf, Louise Lefrançois, Frédéric Lemoine, Christophe Malabat, Corinne Maufrais, Etienne Simon-Lorière, Maud Vanpeene, Sylvie Van der Werf ,Anne-Laure Garand
EPI_ISL_2709294	Labo Analyses med	National Reference Center for Viruses of Respiratory Infections, Institut Pasteur, Paris	Marion Barbet, Sylvie Behillil, Méline Bizard, Angela Brisebarre, Camille Capel, Vincent Enouf, Louise Lefrançois, Frédéric Lemoine, Christophe Malabat, Corinne Maufrais, Damien Mornico, Etienne Simon-Lorière, Maud Vanpeene, Sylvie Van der Werf ,OphéLie Said-Delattre
EPI_ISL_2709304	Labo Analyses med	National Reference Center for Viruses of Respiratory Infections, Institut Pasteur, Paris	Marion Barbet, Sylvie Behillil, Méline Bizard, Angela Brisebarre, Camille Capel, Vincent Enouf, Louise Lefrançois, Frédéric Lemoine, Christophe Malabat, Corinne Maufrais, Etienne Simon-Lorière, Maud Vanpeene, Sylvie Van der Werf ,OphéLie Said-Delattre
EPI_ISL_2709851, EPI_ISL_2709862	POLOKWANE MANKWENG HOSPITAL	National Institute for Communicable Diseases of the National Health Laboratory Service	Amoako DG, Everatt J, Scheepers C, Mohale T, Ntuli N, Mahlangu B, Mnguni A, Ismail A, Bhiman JN
EPI_ISL_2709863, EPI_ISL_2709872, EPI_ISL_2709875, EPI_ISL_2709878	HELEN JOSEPH	National Institute for Communicable Diseases of the National Health Laboratory Service	Amoako DG, Everatt J, Scheepers C, Mohale T, Ntuli N, Mahlangu B, Mnguni A, Ismail A, Bhiman JN
EPI_ISL_2709880, EPI_ISL_2709883, EPI_ISL_2709884	CHRIS HANI BARAGWANATH	National Institute for Communicable Diseases of the National Health Laboratory Service	Amoako DG, Everatt J, Scheepers C, Mohale T, Ntuli N, Mahlangu B, Mnguni A, Ismail A, Bhiman JN
EPI_ISL_2709885	HELEN JOSEPH	National Institute for Communicable Diseases of the National Health Laboratory Service	Amoako DG, Everatt J, Scheepers C, Mohale T, Ntuli N, Mahlangu B, Mnguni A, Ismail A, Bhiman JN
EPI_ISL_2709897, EPI_ISL_2709900, EPI_ISL_2709901, EPI_ISL_2709906, EPI_ISL_2709908, EPI_ISL_2709909, EPI_ISL_2709911, EPI_ISL_2709917, EPI_ISL_2709919, EPI_ISL_2709924, EPI_ISL_2709926, EPI_ISL_2709930, EPI_ISL_2709931, EPI_ISL_2709933, EPI_ISL_2709940			
see above	CHRIS HANI BARAGWANATH	National Institute for Communicable Diseases of the National Health Laboratory Service	Amoako DG, Everatt J, Scheepers C, Mohale T, Ntuli N, Mahlangu B, Mnguni A, Ismail A, Bhiman JN
EPI_ISL_2709953, EPI_ISL_2709957, EPI_ISL_2709960, EPI_ISL_2709979, EPI_ISL_2709982, EPI_ISL_2709987, EPI_ISL_2709988	TAMBO MEMORIAL LABORATORY	National Institute for Communicable Diseases of the National Health Laboratory Service	Amoako DG, Everatt J, Scheepers C, Mohale T, Ntuli N, Mahlangu B, Mnguni A, Ismail A, Bhiman JN
EPI_ISL_2710263,	NHLS Charlotte Maxeke	KRISP, KZN Research	Florette Treurnicht, Bulelani Manene, Kathleen Subramoney, Giandhari Jennifer,

EPI_ISL_2710289, EPI_ISL_2710291	Johannesburg Academic Hospital and the University of the Witwatersrand	Innovation and Sequencing Platform	Pillay Sureshnee, Yajna Ramphal, Naidoo Yeshnee, Tshabuila Derek,Tegally Houriiyah, San James, Wilkinson Eduan, de Oliveira Tulio
EPI_ISL_2710309, EPI_ISL_2710310, EPI_ISL_2710311, EPI_ISL_2710317, EPI_ISL_2710321	BARC / Lancet	KRISP, KZN Research Innovation and Sequencing Platform	Sisonke, Giandhari Jennifer, Pillay Sureshnee, Yajna Ramphal, Naidoo Yeshnee, Tshabuila Derek,Tegally Houriiyah, San James, Wilkinson Eduan, de Oliveira Tulio
EPI_ISL_2710799	Orebro University Hospital, Dept Laboratory Medicine, Clinical Microbiology	Orebro University Hospital	Sundqvist M et al
EPI_ISL_2713000	TXDSHS	TXDSHS	Rashmi Tuladhar, Bonnie Oh, Jenny Zhang, Maliha Rahman, Mayela Pedrueza, Anita Pokharel, Karen Bobier, Lorraine Rodriguez, Myong Koag, Chun Wang, Rachel Lee, Grace Kubin
EPI_ISL_2715018	Atlas Genomics	Seattle Flu Study	Deborah A. Nickerson, Chris D. Frazar, Jover Lee, Benjamin Pelle, Erica Ryke, Matthew Richardson, Amanda Adler, Elisabeth Brandstetter, Peter D. Han, Kairsten Fay, Misja Ilcisin, Kirsten Lacombe, Thomas R. Sibley, Melissa Truong, Caitlin R. Wolf, Romesh Gautom, Geoff Melly, Brian Hiatt, Philip Dykema, Scott Lindquist, Michael Boeckh, Janet A. Englund, Michael Famulare, Barry R. Lutz, Mark J. Rieder, Lea M. Starita, Matthew Thompson, Helen Y. Chu, Jay Shendure, Trevor Bedford
EPI_ISL_2715174	Northwest Laboratory	Seattle Flu Study	Deborah A. Nickerson, Chris D. Frazar, Jover Lee, Benjamin Pelle, Erica Ryke, Matthew Richardson, Amanda Adler, Elisabeth Brandstetter, Peter D. Han, Kairsten Fay, Misja Ilcisin, Kirsten Lacombe, Thomas R. Sibley, Melissa Truong, Caitlin R. Wolf, Romesh Gautom, Geoff Melly, Brian Hiatt, Philip Dykema, Scott Lindquist, Michael Boeckh, Janet A. Englund, Michael Famulare, Barry R. Lutz, Mark J. Rieder, Lea M. Starita, Matthew Thompson, Helen Y. Chu, Jay Shendure, Trevor Bedford
EPI_ISL_2726322, EPI_ISL_2726331, EPI_ISL_2726335, EPI_ISL_2726336, EPI_ISL_2726337, EPI_ISL_2726338, EPI_ISL_2726339, EPI_ISL_2726340, EPI_ISL_2726341, EPI_ISL_2726342, EPI_ISL_2726512, EPI_ISL_2726514			
see above	KU Leuven, Rega Institute, Clinical and Epidemiological Virology	KU Leuven, Rega Institute, Clinical and Epidemiological Virology	Tony Wawina-Bokalanga, Bert Vanmechelen, Joan Marti-Carerras, Piet Maes
EPI_ISL_2727331, EPI_ISL_2727333, EPI_ISL_2727336, EPI_ISL_2727337, EPI_ISL_2727339, EPI_ISL_2727340, EPI_ISL_2727341, EPI_ISL_2727342, EPI_ISL_2727344, EPI_ISL_2727346, EPI_ISL_2727348			
see above	ZARV/NHLS, Department Medical Virology, University of Pretoria	KRISP, KZn Research Innovation and Sequencing Platform	Amy Strydom, Adriano Mendes, Micheala Davids, Sim Mayaphi and Marietjie Venter, Giandhari J, Pillay S, Naidoo Y, Ramphal U, Lessells R, Emmanuel SJ, Tegally H, Wilkinson E, de Oliveira T
EPI_ISL_2736453	Seattle Flu Study	Seattle Flu Study	Deborah A. Nickerson, Chris D. Frazar, Jover Lee, Benjamin Pelle, Erica Ryke, Matthew Richardson, Amanda Adler, Elisabeth Brandstetter, Peter D. Han, Kairsten Fay, Misja Ilcisin, Kirsten Lacombe, Thomas R. Sibley, Melissa Truong, Caitlin R. Wolf, Michael Boeckh, Janet A. Englund, Michael Famulare, Barry R. Lutz, Mark J. Rieder, Lea M. Starita, Matthew Thompson, Jay Shendure, Trevor Bedford, Helen Y. Chu
EPI_ISL_2757735, EPI_ISL_2757740, EPI_ISL_2757745, EPI_ISL_2757746, EPI_ISL_2757747, EPI_ISL_2757748, EPI_ISL_2757750, EPI_ISL_2757751, EPI_ISL_2757753, EPI_ISL_2757754	Department of Virology	Department of Virology	Massab Umair, Aamer Ikram, Muhammad Salman, Nazish Badar, Zaira Rehman, Muhammad Ammar, Syed Adnan Haider
EPI_ISL_2758864, EPI_ISL_2758869	The Ohio State University Applied Microbiology Services Laboratory	The Ohio State University Applied Microbiology Services Laboratory	Seth A. Faith PhD
EPI_ISL_2760111	Bayerisches Landesamt für Gesundheit und Lebensmittelsicherheit (LGL)	Robert Koch Institute	unknown
EPI_ISL_2761339	Sonic - Bioscientia - MVZ Labor Saar GmbH	Robert Koch Institute	unknown
EPI_ISL_2761420	LabKom - MVZ Labor Bochum MLB GmbH	Robert Koch Institute	unknown
EPI_ISL_2761798	CENTOGENE Frankfurt Laboratory: Niederlassung Industriepark Höchst	Robert Koch Institute	unknown
EPI_ISL_2762020, EPI_ISL_2762074	Sonic - Bioscientia - MVZ Labor Saar GmbH	Robert Koch Institute	unknown
EPI_ISL_2762313	Diagnosticum - Labor Neukirchen	Robert Koch Institute	unknown
EPI_ISL_2768014	National Institute of Laboratory Medicine and Referral Center	Genomic Research Lab, BCSIR	Md. Murshed Hasan Sarkar, Mohammad Samir Uzzaman, Eshrar Osman, Md. Ahasan Habib, Shahina Akter, Tanjina Akhter Banu, Barna Goswami, Iffat Jahan, Mohammad Mohi Uddin, Md. Kamrul Islam, Tasnim Nafisa, Md. Maruf Ahmed Molla, Mahmuda Yeasmin, Asish Kumar Ghosh, Arifa Akram, Md. Salim Khan
EPI_ISL_2769141, EPI_ISL_2769142	UniversitätsSpital Zürich	Institute of Medical Virology	Verena Kufner, Gabriela Ziltener, Maryam Zaheri, Stefan Schmutz, Annette Audigé, Maria Grünberg, Kevin Steiner, Jon Huder, Cyril Shah, Riccarda Capaul, Guido Bloemberg, Jürg Böni, Michael Huber, Alexandra Trkola
EPI_ISL_2770441, EPI_ISL_2770481	NHLS Charlotte Maxeke Johannesburg Academic	KRISP, KZN Research Innovation and	Florette Treurnicht, Bulelani Manene, Kathleen Subramoney, Giandhari Jennifer, Pillay Sureshnee, Yajna Ramphal, Naidoo Yeshnee, Tshabuila Derek,Tegally

	Hospital and the University of the Witwatersrand	Sequencing Platform	Houriiyah, San James, Wilkinson Eduan, de Oliveira Tulio
EPI_ISL_2772528	National Center of Infectious and Parasitic Diseases	National Center of Infectious and Parasitic Diseases	Alexiev et al

Supp. Table S4

We gratefully acknowledge the following Authors from the Originating laboratories responsible for obtaining the specimens, as well as the Submitting laboratories where the genome data were generated and shared via GISAID, on which this research is based.

All Submitters of data may be contacted directly via www.gisaid.org

Authors are sorted alphabetically.

Accession ID	Originating Laboratory	Submitting Laboratory	Authors
EPI_ISL_2597929, EPI_ISL_2597932	Nebraska Public Health Laboratory	NPHL COVID-19 Response Team	NPHL COVID-19 Response Team
EPI_ISL_2617990, EPI_ISL_2617991, EPI_ISL_2618009, EPI_ISL_2618010, EPI_ISL_2618013, EPI_ISL_2618016, EPI_ISL_2618018, EPI_ISL_2618019	UW Virology Lab	UW Virology Lab	Pavitra Roychoudhury, Hong Xie, Lasata Shrestha, Tien V. Nguyen, Shah Mohamed Bakhash, Michelle Lin, Noah R. Baker, Ricardo Perez, Sean Ellis, Nathan Breit, Robert J. Livingston, Meei-Li Huang, Keith R Jerome, Patrick Mathias, Alexander Greninger
EPI_ISL_2620723	Genomik Solidaritas Indonesia Laboratorium	Genomik Solidaritas Indonesia Laboratorium	Dhahlia Agustina Cahyono, Alfin Mohammad Abdillah, Normastuti Adhini Tantyo, Carissa Sintca Wijaya, Vania Gavrila Wikasa, Ahmad Zoebad Foeady, Annisa Muthiah Sukirman, Gracia Felias Enos Korompis, Anuraj Shankar, Meutia Ayuputeri Kumaheri, Ariel Pradipta
EPI_ISL_2623094	University College London, Great Ormond Street Hospital for Children NHS Foundation Trust, Imperial College Healthcare NHS Trust	COVID-19 Genomics UK (COG-UK) Consortium	Sergi Castellano, Rachel Williams, Mark Kristiansen, Paola Resende Silva, Sunando Roy, Tony Brooks, Helena Tutill, Paola Niola, Patricia Dyal, Charlotte Williams, Leysa Forrest, Yasmin Panchbhaya, Jacqueline Findlay, Samuel Weeks, Julianne Brown, Kathryn Harris, Paul Randell, James Price, Alison Holmes, Judith Breuer
EPI_ISL_2625108, EPI_ISL_2625119, EPI_ISL_2625142, EPI_ISL_2625143, EPI_ISL_2625144, EPI_ISL_2625145, EPI_ISL_2625146, EPI_ISL_2625149, EPI_ISL_2625151, EPI_ISL_2625153, EPI_ISL_2625157, EPI_ISL_2625159, EPI_ISL_2625160, EPI_ISL_2625165, EPI_ISL_2625166, EPI_ISL_2625168, EPI_ISL_2625169, EPI_ISL_2625171, EPI_ISL_2625172, EPI_ISL_2625173, EPI_ISL_2625177, EPI_ISL_2625178, EPI_ISL_2625180, EPI_ISL_2625181, EPI_ISL_2625182, EPI_ISL_2625184, EPI_ISL_2625185, EPI_ISL_2625186, EPI_ISL_2625187, EPI_ISL_2625188, EPI_ISL_2625189, EPI_ISL_2625190, EPI_ISL_2625191, EPI_ISL_2625192, EPI_ISL_2625195, EPI_ISL_2625196, EPI_ISL_2625197, EPI_ISL_2625199			
see above	Centre for Enzyme Innovation, University of Portsmouth / Translational Research Laboratory, Portsmouth Hospitals NHS Trust / Hampshire Hospitals NHS Foundation Trust / Bournemouth University / University Hospitals Dorset NHS Foundation Trust / Brighton and Sussex University Hospitals NHS Trust / Dartford and Gravesham NHS Trust / Isle of Wight NHS Trust / Maidstone and Tunbridge Wells NHS Trust / University Hospital Southampton NHS Foundation Trust	COVID-19 Genomics UK (COG-UK) Consortium	Samuel Robson, Angela Beckett, Salman Goudarzi, Christopher Fearn, Kate Cook, Katie Loveson, Sharon Glaysher, Scott Elliott, Kelly Bicknell, Sarah Wyllie, Allyson Lloyd, Robert Impey, Anoop Chauhan, Stephen Kidd, Nathan Moore, Nick Cortes, Claire Thomas, Anna Mantzaouratou, Sarah Buchan, Magdalena Barrow, Andrew Butt, Liz Sheridan, Jonnie Seymour, Dorian Crudgington, Ben Macklin, Mohammed Hassan-Ibrahim, Cassandra Malone, Benjamin Cogger, Kevin Tucker, Samirakhon Raupova, Rachael Jeremiah, Anibolina Castigador, Emily Macnaughton, Karen Withell, Kordo Saeed, Jacqui Prieto, Adhyana Mahanama, Buddhini Samaraweera, Siona Silvieri, Emanuela Pelosi, Eleri Wilson-Davies, Sarah Jeremiah, Helen Wheeler, Matthew Harvey, Thea Sass, Helen Umpleby, Stephen Aplin
EPI_ISL_2627547, EPI_ISL_2627555, EPI_ISL_2627562, EPI_ISL_2627566, EPI_ISL_2627567, EPI_ISL_2627571, EPI_ISL_2627573, EPI_ISL_2627575, EPI_ISL_2627578	National Public Health Laboratory, National Centre for Infectious Diseases	National Public Health Laboratory, National Centre for Infectious Diseases	Tze Minn Mak, Katherine Ching, Zhenyang Zhou, Royce Ang, Lin Cui, Raymond Tzer Pin Lin
EPI_ISL_2628025, EPI_ISL_2628026	Montana Public Health Laboratory	Montana Public Health Laboratory	Joy Ritter, Michelle Mozer, Carrie Biskupiak, Deborah Gibson, Michael Dills
EPI_ISL_2628086, EPI_ISL_2628100, EPI_ISL_2628103	Platform BIS UZA/UAntwerpen	Labo Klinische Biologie, UZA	Marie Le Mercier, Jasmine Coppens, Basil Britto Xavier, Christine Lammens, Veerle Matheeussen, Herman Goossens
EPI_ISL_2635462	Synlab MVZ Augsburg	Robert Koch Institute	unknown
EPI_ISL_2635591	Medizinische Laboratorien Düsseldorf	Robert Koch Institute	unknown
EPI_ISL_2635602, EPI_ISL_2635611, EPI_ISL_2635629, EPI_ISL_2635648, EPI_ISL_2635657, EPI_ISL_2635660	nordlab - Partnerschaftspraxis fur Laboratoriumsmedizin	Robert Koch Institute	unknown
EPI_ISL_2635903, EPI_ISL_2635905	Limbach - MVZ Labor Ludwigsburg	Robert Koch Institute	unknown
EPI_ISL_2638165	Lighthouse Lab in Glasgow	Wellcome Sanger Institute for the COVID-19 Genomics UK (COG-UK) Consortium	Harper VanSteenhouse, Yumi Kasai, David Gray, Carol Clugston, Anna Dominiczak and Alex Alderton, Roberto Amato, Jeffrey Barrett, Sonia Goncalves, Ewan Harrison, David K. Jackson, Ian Johnston, Dominic Kwiatkowski, Cordelia Langford, John Sillitoe on behalf of the Wellcome Sanger Institute COVID-19 Surveillance Team
EPI_ISL_2638169, EPI_ISL_2638171, EPI_ISL_2638203, EPI_ISL_2638205	Lighthouse Lab in Alderley Park	Wellcome Sanger Institute for the COVID-19 Genomics UK (COG-UK) Consortium	Jacquelyn Wynn, Mairead Hyland, The Lighthouse Lab in Alderley Park and Alex Alderton, Roberto Amato, Jeffrey Barrett, Sonia Goncalves, Ewan Harrison, David K. Jackson, Ian Johnston, Dominic Kwiatkowski, Cordelia Langford, John Sillitoe on behalf of the Wellcome Sanger Institute COVID-19 Surveillance Team
EPI_ISL_2638223	Lighthouse Lab in Glasgow	Wellcome Sanger Institute for the COVID-19 Genomics UK (COG-UK) Consortium	Harper VanSteenhouse, Yumi Kasai, David Gray, Carol Clugston, Anna Dominiczak and Alex Alderton, Roberto Amato, Jeffrey Barrett, Sonia Goncalves, Ewan Harrison, David K. Jackson, Ian Johnston, Dominic Kwiatkowski, Cordelia Langford, John Sillitoe on behalf of the Wellcome Sanger Institute COVID-19 Surveillance Team
EPI_ISL_2638224, EPI_ISL_2638228, EPI_ISL_2638231, EPI_ISL_2638248	Lighthouse Lab in Alderley Park	Wellcome Sanger Institute for the COVID-19 Genomics UK (COG-UK) Consortium	Jacquelyn Wynn, Mairead Hyland, The Lighthouse Lab in Alderley Park and Alex Alderton, Roberto Amato, Jeffrey Barrett, Sonia Goncalves, Ewan Harrison, David K. Jackson, Ian Johnston, Dominic Kwiatkowski, Cordelia Langford, John Sillitoe on behalf of the Wellcome Sanger Institute COVID-19 Surveillance Team
EPI_ISL_2638265	Lighthouse Lab in Milton Keynes	Wellcome Sanger Institute for the COVID-19 Genomics UK (COG-UK) Consortium	The Lighthouse Lab in Milton Keynes and Alex Alderton, Roberto Amato, Jeffrey Barrett, Sonia Goncalves, Ewan Harrison, David K. Jackson, Ian Johnston, Dominic Kwiatkowski, Cordelia Langford, John Sillitoe on behalf of the Wellcome Sanger Institute COVID-19 Surveillance Team
EPI_ISL_2638269, EPI_ISL_2638275, EPI_ISL_2638278, EPI_ISL_2638283, EPI_ISL_2638290	Lighthouse Lab in Alderley Park	Wellcome Sanger Institute for the COVID-19 Genomics UK (COG-UK) Consortium	Jacquelyn Wynn, Mairead Hyland, The Lighthouse Lab in Alderley Park and Alex Alderton, Roberto Amato, Jeffrey Barrett, Sonia Goncalves, Ewan Harrison, David K. Jackson, Ian Johnston, Dominic Kwiatkowski, Cordelia Langford, John Sillitoe on behalf of the Wellcome Sanger Institute COVID-19 Surveillance Team
EPI_ISL_2638377	Lighthouse Lab in Glasgow	Wellcome Sanger Institute for the COVID-19 Genomics UK (COG-UK) Consortium	Harper VanSteenhouse, Yumi Kasai, David Gray, Carol Clugston, Anna Dominiczak and Alex Alderton, Roberto Amato, Jeffrey Barrett, Sonia Goncalves, Ewan Harrison, David K. Jackson, Ian Johnston, Dominic Kwiatkowski, Cordelia Langford, John Sillitoe on behalf of the Wellcome Sanger Institute COVID-19 Surveillance Team
EPI_ISL_2638384, EPI_ISL_2638388	Lighthouse Lab in Alderley Park	Wellcome Sanger Institute for the COVID-19 Genomics UK (COG-UK) Consortium	Jacquelyn Wynn, Mairead Hyland, The Lighthouse Lab in Alderley Park and Alex Alderton, Roberto Amato, Jeffrey Barrett, Sonia Goncalves, Ewan Harrison, David K. Jackson, Ian Johnston, Dominic Kwiatkowski, Cordelia Langford, John Sillitoe on behalf of the Wellcome Sanger Institute COVID-19 Surveillance Team
EPI_ISL_2638404, EPI_ISL_2638406, EPI_ISL_2638409	Lighthouse Lab in Milton Keynes	Wellcome Sanger Institute for the COVID-19 Genomics UK (COG-UK) Consortium	The Lighthouse Lab in Milton Keynes and Alex Alderton, Roberto Amato, Jeffrey Barrett, Sonia Goncalves, Ewan Harrison, David K. Jackson, Ian Johnston, Dominic Kwiatkowski, Cordelia Langford, John Sillitoe on behalf of the Wellcome Sanger Institute COVID-19 Surveillance Team
EPI_ISL_2638419	Lighthouse Lab in Glasgow	Wellcome Sanger Institute for the COVID-19 Genomics UK (COG-UK) Consortium	Harper VanSteenhouse, Yumi Kasai, David Gray, Carol Clugston, Anna Dominiczak and Alex Alderton, Roberto Amato, Jeffrey Barrett, Sonia Goncalves, Ewan Harrison, David K. Jackson, Ian Johnston, Dominic Kwiatkowski, Cordelia Langford, John Sillitoe on behalf of the Wellcome Sanger Institute COVID-19 Surveillance Team
EPI_ISL_2638428, EPI_ISL_2638430	Lighthouse Lab in Milton Keynes	Wellcome Sanger Institute for the COVID-19 Genomics UK	The Lighthouse Lab in Milton Keynes and Alex Alderton, Roberto Amato, Jeffrey Barrett, Sonia Goncalves, Ewan Harrison, David K. Jackson, Ian Johnston,

		(COG-UK) Consortium	Dominic Kwiatkowski, Cordelia Langford, John Sillitoe on behalf of the Wellcome Sanger Institute COVID-19 Surveillance Team
EPI_ISL_2638435	Lighthouse Lab in Glasgow	Wellcome Sanger Institute for the COVID-19 Genomics UK (COG-UK) Consortium	Harper VanSteenhouse, Yumi Kasai, David Gray, Carol Clugston, Anna Dominiczak and Alex Alderton, Roberto Amato, Jeffrey Barrett, Sonia Goncalves, Ewan Harrison, David K. Jackson, Ian Johnston, Dominic Kwiatkowski, Cordelia Langford, John Sillitoe on behalf of the Wellcome Sanger Institute COVID-19 Surveillance Team
EPI_ISL_2638437, EPI_ISL_2638441	Lighthouse Lab in Milton Keynes	Wellcome Sanger Institute for the COVID-19 Genomics UK (COG-UK) Consortium	The Lighthouse Lab in Milton Keynes and Alex Alderton, Roberto Amato, Jeffrey Barrett, Sonia Goncalves, Ewan Harrison, David K. Jackson, Ian Johnston, Dominic Kwiatkowski, Cordelia Langford, John Sillitoe on behalf of the Wellcome Sanger Institute COVID-19 Surveillance Team
EPI_ISL_2638453	Lighthouse Lab in Glasgow	Wellcome Sanger Institute for the COVID-19 Genomics UK (COG-UK) Consortium	Harper VanSteenhouse, Yumi Kasai, David Gray, Carol Clugston, Anna Dominiczak and Alex Alderton, Roberto Amato, Jeffrey Barrett, Sonia Goncalves, Ewan Harrison, David K. Jackson, Ian Johnston, Dominic Kwiatkowski, Cordelia Langford, John Sillitoe on behalf of the Wellcome Sanger Institute COVID-19 Surveillance Team
EPI_ISL_2638478, EPI_ISL_2638487, EPI_ISL_2638554, EPI_ISL_2638588	Lighthouse Lab in Milton Keynes	Wellcome Sanger Institute for the COVID-19 Genomics UK (COG-UK) Consortium	The Lighthouse Lab in Milton Keynes and Alex Alderton, Roberto Amato, Jeffrey Barrett, Sonia Goncalves, Ewan Harrison, David K. Jackson, Ian Johnston, Dominic Kwiatkowski, Cordelia Langford, John Sillitoe on behalf of the Wellcome Sanger Institute COVID-19 Surveillance Team
EPI_ISL_2638595	Lighthouse Lab in Glasgow	Wellcome Sanger Institute for the COVID-19 Genomics UK (COG-UK) Consortium	Harper VanSteenhouse, Yumi Kasai, David Gray, Carol Clugston, Anna Dominiczak and Alex Alderton, Roberto Amato, Jeffrey Barrett, Sonia Goncalves, Ewan Harrison, David K. Jackson, Ian Johnston, Dominic Kwiatkowski, Cordelia Langford, John Sillitoe on behalf of the Wellcome Sanger Institute COVID-19 Surveillance Team
EPI_ISL_2638597, EPI_ISL_2638621, EPI_ISL_2638624	Lighthouse Lab in Milton Keynes	Wellcome Sanger Institute for the COVID-19 Genomics UK (COG-UK) Consortium	The Lighthouse Lab in Milton Keynes and Alex Alderton, Roberto Amato, Jeffrey Barrett, Sonia Goncalves, Ewan Harrison, David K. Jackson, Ian Johnston, Dominic Kwiatkowski, Cordelia Langford, John Sillitoe on behalf of the Wellcome Sanger Institute COVID-19 Surveillance Team
EPI_ISL_2638625	Lighthouse Lab in Glasgow	Wellcome Sanger Institute for the COVID-19 Genomics UK (COG-UK) Consortium	Harper VanSteenhouse, Yumi Kasai, David Gray, Carol Clugston, Anna Dominiczak and Alex Alderton, Roberto Amato, Jeffrey Barrett, Sonia Goncalves, Ewan Harrison, David K. Jackson, Ian Johnston, Dominic Kwiatkowski, Cordelia Langford, John Sillitoe on behalf of the Wellcome Sanger Institute COVID-19 Surveillance Team
EPI_ISL_2638631, EPI_ISL_2638639, EPI_ISL_2638640, EPI_ISL_2638645	Lighthouse Lab in Milton Keynes	Wellcome Sanger Institute for the COVID-19 Genomics UK (COG-UK) Consortium	The Lighthouse Lab in Milton Keynes and Alex Alderton, Roberto Amato, Jeffrey Barrett, Sonia Goncalves, Ewan Harrison, David K. Jackson, Ian Johnston, Dominic Kwiatkowski, Cordelia Langford, John Sillitoe on behalf of the Wellcome Sanger Institute COVID-19 Surveillance Team
EPI_ISL_2638651, EPI_ISL_2638665	Lighthouse Laboratory Plymouth	Wellcome Sanger Institute for the COVID-19 Genomics UK (COG-UK) Consortium	Lighthouse Laboratory Plymouth and Alex Alderton, Roberto Amato, Jeffrey Barrett, Sonia Goncalves, Ewan Harrison, David K. Jackson, Ian Johnston, Dominic Kwiatkowski, Cordelia Langford, John Sillitoe on behalf of the Wellcome Sanger Institute COVID-19 Surveillance Team
EPI_ISL_2638667	Lighthouse Lab in Milton Keynes	Wellcome Sanger Institute for the COVID-19 Genomics UK (COG-UK) Consortium	The Lighthouse Lab in Milton Keynes and Alex Alderton, Roberto Amato, Jeffrey Barrett, Sonia Goncalves, Ewan Harrison, David K. Jackson, Ian Johnston, Dominic Kwiatkowski, Cordelia Langford, John Sillitoe on behalf of the Wellcome Sanger Institute COVID-19 Surveillance Team
EPI_ISL_2638674, EPI_ISL_2638675, EPI_ISL_2638676	Lighthouse Lab in Glasgow	Wellcome Sanger Institute for the COVID-19 Genomics UK (COG-UK) Consortium	Harper VanSteenhouse, Yumi Kasai, David Gray, Carol Clugston, Anna Dominiczak and Alex Alderton, Roberto Amato, Jeffrey Barrett, Sonia Goncalves, Ewan Harrison, David K. Jackson, Ian Johnston, Dominic Kwiatkowski, Cordelia Langford, John Sillitoe on behalf of the Wellcome Sanger Institute COVID-19 Surveillance Team
EPI_ISL_2638678	Lighthouse Lab in Milton Keynes	Wellcome Sanger Institute for the COVID-19 Genomics UK (COG-UK) Consortium	The Lighthouse Lab in Milton Keynes and Alex Alderton, Roberto Amato, Jeffrey Barrett, Sonia Goncalves, Ewan Harrison, David K. Jackson, Ian Johnston, Dominic Kwiatkowski, Cordelia Langford, John Sillitoe on behalf of the Wellcome Sanger Institute COVID-19 Surveillance Team
EPI_ISL_2638681	Lighthouse Lab in Glasgow	Wellcome Sanger Institute for the COVID-19 Genomics UK (COG-UK) Consortium	Harper VanSteenhouse, Yumi Kasai, David Gray, Carol Clugston, Anna Dominiczak and Alex Alderton, Roberto Amato, Jeffrey Barrett, Sonia Goncalves, Ewan Harrison, David K. Jackson, Ian Johnston, Dominic Kwiatkowski, Cordelia Langford, John Sillitoe on behalf of the Wellcome Sanger Institute COVID-19 Surveillance Team
EPI_ISL_2638682	Lighthouse Lab in Milton Keynes	Wellcome Sanger Institute for the COVID-19 Genomics UK (COG-UK) Consortium	The Lighthouse Lab in Milton Keynes and Alex Alderton, Roberto Amato, Jeffrey Barrett, Sonia Goncalves, Ewan Harrison, David K. Jackson, Ian Johnston, Dominic Kwiatkowski, Cordelia Langford, John Sillitoe on behalf of the Wellcome Sanger Institute COVID-19 Surveillance Team
EPI_ISL_2638683	Lighthouse Lab in Glasgow	Wellcome Sanger Institute for the COVID-19 Genomics UK (COG-UK) Consortium	Harper VanSteenhouse, Yumi Kasai, David Gray, Carol Clugston, Anna Dominiczak and Alex Alderton, Roberto Amato, Jeffrey Barrett, Sonia Goncalves, Ewan Harrison, David K. Jackson, Ian Johnston, Dominic Kwiatkowski, Cordelia Langford, John Sillitoe on behalf of the Wellcome Sanger Institute COVID-19 Surveillance Team
EPI_ISL_2638687	Lighthouse Lab in Milton Keynes	Wellcome Sanger Institute for the COVID-19 Genomics UK (COG-UK) Consortium	The Lighthouse Lab in Milton Keynes and Alex Alderton, Roberto Amato, Jeffrey Barrett, Sonia Goncalves, Ewan Harrison, David K. Jackson, Ian Johnston, Dominic Kwiatkowski, Cordelia Langford, John Sillitoe on behalf of the Wellcome Sanger Institute COVID-19 Surveillance Team
EPI_ISL_2638691, EPI_ISL_2638692, EPI_ISL_2638696	Lighthouse Lab in Glasgow	Wellcome Sanger Institute for the COVID-19 Genomics UK (COG-UK) Consortium	Harper VanSteenhouse, Yumi Kasai, David Gray, Carol Clugston, Anna Dominiczak and Alex Alderton, Roberto Amato, Jeffrey Barrett, Sonia Goncalves, Ewan Harrison, David K. Jackson, Ian Johnston, Dominic Kwiatkowski, Cordelia Langford, John Sillitoe on behalf of the Wellcome Sanger Institute COVID-19 Surveillance Team
EPI_ISL_2638705, EPI_ISL_2638713, EPI_ISL_2638714, EPI_ISL_2638723	Lighthouse Lab in Milton Keynes	Wellcome Sanger Institute for the COVID-19 Genomics UK (COG-UK) Consortium	The Lighthouse Lab in Milton Keynes and Alex Alderton, Roberto Amato, Jeffrey Barrett, Sonia Goncalves, Ewan Harrison, David K. Jackson, Ian Johnston, Dominic Kwiatkowski, Cordelia Langford, John Sillitoe on behalf of the Wellcome Sanger Institute COVID-19 Surveillance Team
EPI_ISL_2638729, EPI_ISL_2638731	Lighthouse Lab in Glasgow	Wellcome Sanger Institute for the COVID-19 Genomics UK (COG-UK) Consortium	Harper VanSteenhouse, Yumi Kasai, David Gray, Carol Clugston, Anna Dominiczak and Alex Alderton, Roberto Amato, Jeffrey Barrett, Sonia Goncalves, Ewan Harrison, David K. Jackson, Ian Johnston, Dominic Kwiatkowski, Cordelia Langford, John Sillitoe on behalf of the Wellcome Sanger Institute COVID-19 Surveillance Team
EPI_ISL_2638732, EPI_ISL_2638737, EPI_ISL_2638794, EPI_ISL_2638795, EPI_ISL_2638862	Lighthouse Lab in Milton Keynes	Wellcome Sanger Institute for the COVID-19 Genomics UK (COG-UK) Consortium	The Lighthouse Lab in Milton Keynes and Alex Alderton, Roberto Amato, Jeffrey Barrett, Sonia Goncalves, Ewan Harrison, David K. Jackson, Ian Johnston, Dominic Kwiatkowski, Cordelia Langford, John Sillitoe on behalf of the Wellcome Sanger Institute COVID-19 Surveillance Team
EPI_ISL_2638865	Lighthouse Lab in Glasgow	Wellcome Sanger Institute for the COVID-19 Genomics UK (COG-UK) Consortium	Harper VanSteenhouse, Yumi Kasai, David Gray, Carol Clugston, Anna Dominiczak and Alex Alderton, Roberto Amato, Jeffrey Barrett, Sonia Goncalves, Ewan Harrison, David K. Jackson, Ian Johnston, Dominic Kwiatkowski, Cordelia Langford, John Sillitoe on behalf of the Wellcome Sanger Institute COVID-19 Surveillance Team
EPI_ISL_2638870	Lighthouse Lab in Milton Keynes	Wellcome Sanger Institute for the COVID-19 Genomics UK (COG-UK) Consortium	The Lighthouse Lab in Milton Keynes and Alex Alderton, Roberto Amato, Jeffrey Barrett, Sonia Goncalves, Ewan Harrison, David K. Jackson, Ian Johnston, Dominic Kwiatkowski, Cordelia Langford, John Sillitoe on behalf of the Wellcome Sanger Institute COVID-19 Surveillance Team
EPI_ISL_2638873	Lighthouse Laboratory Plymouth	Wellcome Sanger Institute for the COVID-19 Genomics UK (COG-UK) Consortium	Lighthouse Laboratory Plymouth and Alex Alderton, Roberto Amato, Jeffrey Barrett, Sonia Goncalves, Ewan Harrison, David K. Jackson, Ian Johnston, Dominic Kwiatkowski, Cordelia Langford, John Sillitoe on behalf of the Wellcome Sanger Institute COVID-19 Surveillance Team
EPI_ISL_2638876, EPI_ISL_2638880, EPI_ISL_2638882, EPI_ISL_2638886	Lighthouse Lab in Milton Keynes	Wellcome Sanger Institute for the COVID-19 Genomics UK (COG-UK) Consortium	The Lighthouse Lab in Milton Keynes and Alex Alderton, Roberto Amato, Jeffrey Barrett, Sonia Goncalves, Ewan Harrison, David K. Jackson, Ian Johnston, Dominic Kwiatkowski, Cordelia Langford, John Sillitoe on behalf of the Wellcome Sanger Institute COVID-19 Surveillance Team
EPI_ISL_2638887	Lighthouse Lab in Glasgow	Wellcome Sanger Institute for the COVID-19 Genomics UK (COG-UK) Consortium	Harper VanSteenhouse, Yumi Kasai, David Gray, Carol Clugston, Anna Dominiczak and Alex Alderton, Roberto Amato, Jeffrey Barrett, Sonia Goncalves, Ewan Harrison, David K. Jackson, Ian Johnston, Dominic Kwiatkowski, Cordelia Langford, John Sillitoe on behalf of the Wellcome Sanger Institute COVID-19 Surveillance Team
EPI_ISL_2638888, EPI_ISL_2638892	Lighthouse Lab in Milton Keynes	Wellcome Sanger Institute for the COVID-19 Genomics UK (COG-UK) Consortium	The Lighthouse Lab in Milton Keynes and Alex Alderton, Roberto Amato, Jeffrey Barrett, Sonia Goncalves, Ewan Harrison, David K. Jackson, Ian Johnston, Dominic Kwiatkowski, Cordelia Langford, John Sillitoe on behalf of the Wellcome Sanger Institute COVID-19 Surveillance Team
EPI_ISL_2638894, EPI_ISL_2638901	Lighthouse Laboratory Plymouth	Wellcome Sanger Institute for the COVID-19 Genomics UK (COG-UK) Consortium	Lighthouse Laboratory Plymouth and Alex Alderton, Roberto Amato, Jeffrey Barrett, Sonia Goncalves, Ewan Harrison, David K. Jackson, Ian Johnston, Dominic Kwiatkowski, Cordelia Langford, John Sillitoe on behalf of the Wellcome Sanger Institute COVID-19 Surveillance Team
EPI_ISL_2638902, EPI_ISL_2638904	Lighthouse Lab in Glasgow	Wellcome Sanger Institute for the COVID-19 Genomics UK (COG-UK) Consortium	Harper VanSteenhouse, Yumi Kasai, David Gray, Carol Clugston, Anna Dominiczak and Alex Alderton, Roberto Amato, Jeffrey Barrett, Sonia Goncalves, Ewan Harrison, David K. Jackson, Ian Johnston, Dominic Kwiatkowski, Cordelia Langford, John Sillitoe on behalf of the Wellcome Sanger Institute COVID-19 Surveillance Team
EPI_ISL_2638907	Lighthouse Lab in Milton Keynes	Wellcome Sanger Institute for the COVID-19 Genomics UK (COG-UK) Consortium	The Lighthouse Lab in Milton Keynes and Alex Alderton, Roberto Amato, Jeffrey Barrett, Sonia Goncalves, Ewan Harrison, David K. Jackson, Ian Johnston, Dominic Kwiatkowski, Cordelia Langford, John Sillitoe on behalf of the Wellcome Sanger Institute COVID-19 Surveillance Team
EPI_ISL_2638923	Lighthouse Laboratory Plymouth	Wellcome Sanger Institute for the COVID-19 Genomics UK	Lighthouse Laboratory Plymouth and Alex Alderton, Roberto Amato, Jeffrey Barrett, Sonia Goncalves, Ewan Harrison, David K. Jackson, Ian Johnston,

		(COG-UK) Consortium	Dominic Kwiatkowski, Cordelia Langford, John Sillitoe on behalf of the Wellcome Sanger Institute COVID-19 Surveillance Team
EPI_ISL_2638961	Health Services Laboratories	Wellcome Sanger Institute for the COVID-19 Genomics UK (COG-UK) Consortium	Health Services Laboratories and Alex Alderton, Roberto Amato, Jeffrey Barrett, Sonia Goncalves, Ewan Harrison, David K. Jackson, Ian Johnston, Dominic Kwiatkowski, Cordelia Langford, John Sillitoe on behalf of the Wellcome Sanger Institute COVID-19 Surveillance Team
EPI_ISL_2638975	Lighthouse Laboratory Plymouth	Wellcome Sanger Institute for the COVID-19 Genomics UK (COG-UK) Consortium	Lighthouse Laboratory Plymouth and Alex Alderton, Roberto Amato, Jeffrey Barrett, Sonia Goncalves, Ewan Harrison, David K. Jackson, Ian Johnston, Dominic Kwiatkowski, Cordelia Langford, John Sillitoe on behalf of the Wellcome Sanger Institute COVID-19 Surveillance Team
EPI_ISL_2639001	Health Services Laboratories	Wellcome Sanger Institute for the COVID-19 Genomics UK (COG-UK) Consortium	Health Services Laboratories and Alex Alderton, Roberto Amato, Jeffrey Barrett, Sonia Goncalves, Ewan Harrison, David K. Jackson, Ian Johnston, Dominic Kwiatkowski, Cordelia Langford, John Sillitoe on behalf of the Wellcome Sanger Institute COVID-19 Surveillance Team
EPI_ISL_2639002, EPI_ISL_2639004, EPI_ISL_2639018	Lighthouse Lab in Milton Keynes	Wellcome Sanger Institute for the COVID-19 Genomics UK (COG-UK) Consortium	The Lighthouse Lab in Milton Keynes and Alex Alderton, Roberto Amato, Jeffrey Barrett, Sonia Goncalves, Ewan Harrison, David K. Jackson, Ian Johnston, Dominic Kwiatkowski, Cordelia Langford, John Sillitoe on behalf of the Wellcome Sanger Institute COVID-19 Surveillance Team
EPI_ISL_2639023	Lighthouse Laboratory Plymouth	Wellcome Sanger Institute for the COVID-19 Genomics UK (COG-UK) Consortium	Lighthouse Laboratory Plymouth and Alex Alderton, Roberto Amato, Jeffrey Barrett, Sonia Goncalves, Ewan Harrison, David K. Jackson, Ian Johnston, Dominic Kwiatkowski, Cordelia Langford, John Sillitoe on behalf of the Wellcome Sanger Institute COVID-19 Surveillance Team
EPI_ISL_2639028	Lighthouse Lab in Milton Keynes	Wellcome Sanger Institute for the COVID-19 Genomics UK (COG-UK) Consortium	The Lighthouse Lab in Milton Keynes and Alex Alderton, Roberto Amato, Jeffrey Barrett, Sonia Goncalves, Ewan Harrison, David K. Jackson, Ian Johnston, Dominic Kwiatkowski, Cordelia Langford, John Sillitoe on behalf of the Wellcome Sanger Institute COVID-19 Surveillance Team
EPI_ISL_2639104	Lighthouse Laboratory Plymouth	Wellcome Sanger Institute for the COVID-19 Genomics UK (COG-UK) Consortium	Lighthouse Laboratory Plymouth and Alex Alderton, Roberto Amato, Jeffrey Barrett, Sonia Goncalves, Ewan Harrison, David K. Jackson, Ian Johnston, Dominic Kwiatkowski, Cordelia Langford, John Sillitoe on behalf of the Wellcome Sanger Institute COVID-19 Surveillance Team
EPI_ISL_2639110	Lighthouse Lab in Milton Keynes	Wellcome Sanger Institute for the COVID-19 Genomics UK (COG-UK) Consortium	The Lighthouse Lab in Milton Keynes and Alex Alderton, Roberto Amato, Jeffrey Barrett, Sonia Goncalves, Ewan Harrison, David K. Jackson, Ian Johnston, Dominic Kwiatkowski, Cordelia Langford, John Sillitoe on behalf of the Wellcome Sanger Institute COVID-19 Surveillance Team
EPI_ISL_2639113	Health Services Laboratories	Wellcome Sanger Institute for the COVID-19 Genomics UK (COG-UK) Consortium	Health Services Laboratories and Alex Alderton, Roberto Amato, Jeffrey Barrett, Sonia Goncalves, Ewan Harrison, David K. Jackson, Ian Johnston, Dominic Kwiatkowski, Cordelia Langford, John Sillitoe on behalf of the Wellcome Sanger Institute COVID-19 Surveillance Team
EPI_ISL_2639119	Lighthouse Laboratory Plymouth	Wellcome Sanger Institute for the COVID-19 Genomics UK (COG-UK) Consortium	Lighthouse Laboratory Plymouth and Alex Alderton, Roberto Amato, Jeffrey Barrett, Sonia Goncalves, Ewan Harrison, David K. Jackson, Ian Johnston, Dominic Kwiatkowski, Cordelia Langford, John Sillitoe on behalf of the Wellcome Sanger Institute COVID-19 Surveillance Team
EPI_ISL_2639145	Lighthouse Lab in Milton Keynes	Wellcome Sanger Institute for the COVID-19 Genomics UK (COG-UK) Consortium	The Lighthouse Lab in Milton Keynes and Alex Alderton, Roberto Amato, Jeffrey Barrett, Sonia Goncalves, Ewan Harrison, David K. Jackson, Ian Johnston, Dominic Kwiatkowski, Cordelia Langford, John Sillitoe on behalf of the Wellcome Sanger Institute COVID-19 Surveillance Team
EPI_ISL_2639168	Health Services Laboratories	Wellcome Sanger Institute for the COVID-19 Genomics UK (COG-UK) Consortium	Health Services Laboratories and Alex Alderton, Roberto Amato, Jeffrey Barrett, Sonia Goncalves, Ewan Harrison, David K. Jackson, Ian Johnston, Dominic Kwiatkowski, Cordelia Langford, John Sillitoe on behalf of the Wellcome Sanger Institute COVID-19 Surveillance Team
EPI_ISL_2639169	Lighthouse Laboratory Plymouth	Wellcome Sanger Institute for the COVID-19 Genomics UK (COG-UK) Consortium	Lighthouse Laboratory Plymouth and Alex Alderton, Roberto Amato, Jeffrey Barrett, Sonia Goncalves, Ewan Harrison, David K. Jackson, Ian Johnston, Dominic Kwiatkowski, Cordelia Langford, John Sillitoe on behalf of the Wellcome Sanger Institute COVID-19 Surveillance Team
EPI_ISL_2639170, EPI_ISL_2639195, EPI_ISL_2639197, EPI_ISL_2639198	Lighthouse Lab in Milton Keynes	Wellcome Sanger Institute for the COVID-19 Genomics UK (COG-UK) Consortium	The Lighthouse Lab in Milton Keynes and Alex Alderton, Roberto Amato, Jeffrey Barrett, Sonia Goncalves, Ewan Harrison, David K. Jackson, Ian Johnston, Dominic Kwiatkowski, Cordelia Langford, John Sillitoe on behalf of the Wellcome Sanger Institute COVID-19 Surveillance Team
EPI_ISL_2639200	Lighthouse Laboratory Plymouth	Wellcome Sanger Institute for the COVID-19 Genomics UK (COG-UK) Consortium	Lighthouse Laboratory Plymouth and Alex Alderton, Roberto Amato, Jeffrey Barrett, Sonia Goncalves, Ewan Harrison, David K. Jackson, Ian Johnston, Dominic Kwiatkowski, Cordelia Langford, John Sillitoe on behalf of the Wellcome Sanger Institute COVID-19 Surveillance Team
EPI_ISL_2639202	Lighthouse Lab in Milton Keynes	Wellcome Sanger Institute for the COVID-19 Genomics UK (COG-UK) Consortium	The Lighthouse Lab in Milton Keynes and Alex Alderton, Roberto Amato, Jeffrey Barrett, Sonia Goncalves, Ewan Harrison, David K. Jackson, Ian Johnston, Dominic Kwiatkowski, Cordelia Langford, John Sillitoe on behalf of the Wellcome Sanger Institute COVID-19 Surveillance Team
EPI_ISL_2639204	Health Services Laboratories	Wellcome Sanger Institute for the COVID-19 Genomics UK (COG-UK) Consortium	Health Services Laboratories and Alex Alderton, Roberto Amato, Jeffrey Barrett, Sonia Goncalves, Ewan Harrison, David K. Jackson, Ian Johnston, Dominic Kwiatkowski, Cordelia Langford, John Sillitoe on behalf of the Wellcome Sanger Institute COVID-19 Surveillance Team
EPI_ISL_2639223, EPI_ISL_2639252	Lighthouse Lab in Milton Keynes	Wellcome Sanger Institute for the COVID-19 Genomics UK (COG-UK) Consortium	The Lighthouse Lab in Milton Keynes and Alex Alderton, Roberto Amato, Jeffrey Barrett, Sonia Goncalves, Ewan Harrison, David K. Jackson, Ian Johnston, Dominic Kwiatkowski, Cordelia Langford, John Sillitoe on behalf of the Wellcome Sanger Institute COVID-19 Surveillance Team
EPI_ISL_2639257, EPI_ISL_2639258, EPI_ISL_2639260, EPI_ISL_2639261, EPI_ISL_2639262, EPI_ISL_2639263, EPI_ISL_2639264, EPI_ISL_2639267, EPI_ISL_2639268, EPI_ISL_2639269, EPI_ISL_2639300, EPI_ISL_2639302, EPI_ISL_2639303, EPI_ISL_2639306, EPI_ISL_2639307, EPI_ISL_2639308, EPI_ISL_2639309, EPI_ISL_2639310, EPI_ISL_2639311, EPI_ISL_2639316, EPI_ISL_2639317, EPI_ISL_2639319, EPI_ISL_2639320, EPI_ISL_2639321, EPI_ISL_2639322, EPI_ISL_2639323, EPI_ISL_2639324, EPI_ISL_2639325, EPI_ISL_2639326, EPI_ISL_2639327, EPI_ISL_2639328, EPI_ISL_2639329, EPI_ISL_2639331, EPI_ISL_2639332, EPI_ISL_2639333, EPI_ISL_2639335, EPI_ISL_2639336, EPI_ISL_2639337, EPI_ISL_2639338, EPI_ISL_2639339, EPI_ISL_2639341, EPI_ISL_2639343, EPI_ISL_2639344, EPI_ISL_2639345, EPI_ISL_2639347, EPI_ISL_2639352, EPI_ISL_2639354, EPI_ISL_2639355, EPI_ISL_2639356, EPI_ISL_2639357, EPI_ISL_2639358, EPI_ISL_2639359, EPI_ISL_2639361, EPI_ISL_2639362, EPI_ISL_2639363, EPI_ISL_2639364, EPI_ISL_2639365, EPI_ISL_2639366, EPI_ISL_2639368, EPI_ISL_2639369, EPI_ISL_2639370, EPI_ISL_2639371, EPI_ISL_2639372, EPI_ISL_2639373, EPI_ISL_2639374, EPI_ISL_2639375, EPI_ISL_2639376, EPI_ISL_2639378, EPI_ISL_2639379, EPI_ISL_2639380, EPI_ISL_2639381, EPI_ISL_2639383, EPI_ISL_2639384, EPI_ISL_2639385, EPI_ISL_2639387, EPI_ISL_2639388, EPI_ISL_2639389, EPI_ISL_2639390, EPI_ISL_2639391, EPI_ISL_2639392, EPI_ISL_2639394, EPI_ISL_2639395, EPI_ISL_2639396, EPI_ISL_2639397, EPI_ISL_2639398, EPI_ISL_2639400, EPI_ISL_2639401, EPI_ISL_2639403, EPI_ISL_2639430, EPI_ISL_2639431, EPI_ISL_2639433, EPI_ISL_2639436, EPI_ISL_2639438, EPI_ISL_2639439, EPI_ISL_2639441, EPI_ISL_2639442, EPI_ISL_2639443, EPI_ISL_2639446, EPI_ISL_2639448, EPI_ISL_2639449, EPI_ISL_2639451, EPI_ISL_2639453, EPI_ISL_2639456, EPI_ISL_2639457, EPI_ISL_2639459, EPI_ISL_2639460, EPI_ISL_2639461, EPI_ISL_2639462, EPI_ISL_2639463, EPI_ISL_2639465, EPI_ISL_2639466, EPI_ISL_2639469, EPI_ISL_2639470, EPI_ISL_2639473, EPI_ISL_2639474, EPI_ISL_2639477, EPI_ISL_2639478, EPI_ISL_2639479, EPI_ISL_2639480, EPI_ISL_2639481, EPI_ISL_2639482, EPI_ISL_2639483, EPI_ISL_2639485, EPI_ISL_2639486, EPI_ISL_2639487, EPI_ISL_2639488, EPI_ISL_2639489, EPI_ISL_2639490, EPI_ISL_2639491, EPI_ISL_2639492, EPI_ISL_2639494, EPI_ISL_2639495, EPI_ISL_2639497, EPI_ISL_2639503, EPI_ISL_2639505, EPI_ISL_2639506, EPI_ISL_2639507, EPI_ISL_2639508, EPI_ISL_2639510, EPI_ISL_2639513, EPI_ISL_2639514, EPI_ISL_2639515, EPI_ISL_2639517, EPI_ISL_2639519, EPI_ISL_2639520, EPI_ISL_2639521, EPI_ISL_2639522, EPI_ISL_2639524, EPI_ISL_2639525, EPI_ISL_2639527, EPI_ISL_2639528, EPI_ISL_2639529, EPI_ISL_2639530, EPI_ISL_2639532, EPI_ISL_2639533, EPI_ISL_2639534, EPI_ISL_2639536, EPI_ISL_2639537, EPI_ISL_2639538, EPI_ISL_2639540, EPI_ISL_2639541, EPI_ISL_2639542, EPI_ISL_2639545, EPI_ISL_2639546, EPI_ISL_2639549, EPI_ISL_2639550, EPI_ISL_2639551, EPI_ISL_2639554, EPI_ISL_2639555, EPI_ISL_2639556, EPI_ISL_2639557, EPI_ISL_2639558, EPI_ISL_2639559, EPI_ISL_2639560, EPI_ISL_2639561, EPI_ISL_2639562, EPI_ISL_2639563, EPI_ISL_2639564, EPI_ISL_2639565, EPI_ISL_2639567, EPI_ISL_2639569, EPI_ISL_2639572, EPI_ISL_2639573, EPI_ISL_2639574, EPI_ISL_2639577, EPI_ISL_2639581, EPI_ISL_2639582, EPI_ISL_2639591, EPI_ISL_2639594, EPI_ISL_2639595, EPI_ISL_2639597, EPI_ISL_2639670			
see above	Lighthouse Lab in Alderley Park	Wellcome Sanger Institute for the COVID-19 Genomics UK (COG-UK) Consortium	Jacquelyn Wynn, Mairead Hyland, The Lighthouse Lab in Alderley Park and Alex Alderton, Roberto Amato, Jeffrey Barrett, Sonia Goncalves, Ewan Harrison, David K. Jackson, Ian Johnston, Dominic Kwiatkowski, Cordelia Langford, John Sillitoe on behalf of the Wellcome Sanger Institute COVID-19 Surveillance Team
EPI_ISL_2639677	Lighthouse Lab in Glasgow	Wellcome Sanger Institute for the COVID-19 Genomics UK (COG-UK) Consortium	Harper VanSteenhouse, Yumi Kasai, David Gray, Carol Clugston, Anna Dominiczak and Alex Alderton, Roberto Amato, Jeffrey Barrett, Sonia Goncalves, Ewan Harrison, David K. Jackson, Ian Johnston, Dominic Kwiatkowski, Cordelia Langford, John Sillitoe on behalf of the Wellcome Sanger Institute COVID-19 Surveillance Team
EPI_ISL_2639678, EPI_ISL_2639680, EPI_ISL_2639681, EPI_ISL_2639687, EPI_ISL_2639692, EPI_ISL_2639711, EPI_ISL_2639714, EPI_ISL_2639715, EPI_ISL_2639719	Lighthouse Lab in Alderley Park	Wellcome Sanger Institute for the COVID-19 Genomics UK (COG-UK) Consortium	Jacquelyn Wynn, Mairead Hyland, The Lighthouse Lab in Alderley Park and Alex Alderton, Roberto Amato, Jeffrey Barrett, Sonia Goncalves, Ewan Harrison, David K. Jackson, Ian Johnston, Dominic Kwiatkowski, Cordelia Langford, John Sillitoe on behalf of the Wellcome Sanger Institute COVID-19 Surveillance Team
EPI_ISL_2639733	Lighthouse Lab in Glasgow	Wellcome Sanger Institute for the COVID-19 Genomics UK (COG-UK) Consortium	Harper VanSteenhouse, Yumi Kasai, David Gray, Carol Clugston, Anna Dominiczak and Alex Alderton, Roberto Amato, Jeffrey Barrett, Sonia Goncalves, Ewan Harrison, David K. Jackson, Ian Johnston, Dominic Kwiatkowski, Cordelia Langford, John Sillitoe on behalf of the Wellcome Sanger Institute COVID-19 Surveillance Team
EPI_ISL_2639734, EPI_ISL_2639735, EPI_ISL_2639739, EPI_ISL_2639744, EPI_ISL_2639745, EPI_ISL_2639749, EPI_ISL_2639754, EPI_ISL_2639761, EPI_ISL_2639763, EPI_ISL_2639766, EPI_ISL_2639767, EPI_ISL_2639768, EPI_ISL_2639771, EPI_ISL_2639772, EPI_ISL_2639776, EPI_ISL_2639785, EPI_ISL_2639786, EPI_ISL_2639787, EPI_ISL_2639795, EPI_ISL_2639800, EPI_ISL_2639801, EPI_ISL_2639803, EPI_ISL_2639808, EPI_ISL_2639809, EPI_ISL_2639810, EPI_ISL_2639817, EPI_ISL_2639819, EPI_ISL_2639821, EPI_ISL_2639826, EPI_ISL_2639881, EPI_ISL_2639882, EPI_ISL_2639883			
see above	Lighthouse Lab in Alderley Park	Wellcome Sanger Institute for the COVID-19 Genomics UK (COG-UK) Consortium	Jacquelyn Wynn, Mairead Hyland, The Lighthouse Lab in Alderley Park and Alex Alderton, Roberto Amato, Jeffrey Barrett, Sonia Goncalves, Ewan Harrison, David K. Jackson, Ian Johnston, Dominic Kwiatkowski, Cordelia Langford, John Sillitoe on behalf of the Wellcome Sanger Institute COVID-19 Surveillance Team
EPI_ISL_2639887	Lighthouse Laboratory Plymouth	Wellcome Sanger Institute for the COVID-19 Genomics UK (COG-UK) Consortium	Lighthouse Laboratory Plymouth and Alex Alderton, Roberto Amato, Jeffrey Barrett, Sonia Goncalves, Ewan Harrison, David K. Jackson, Ian Johnston, Dominic Kwiatkowski, Cordelia Langford, John Sillitoe on behalf of the Wellcome Sanger Institute COVID-19 Surveillance Team
EPI_ISL_2651414, EPI_ISL_2651425, EPI_ISL_2651449, EPI_ISL_2651461, EPI_ISL_2651478	Lighthouse Lab in Milton Keynes	Wellcome Sanger Institute for the COVID-19 Genomics UK (COG-UK) Consortium	The Lighthouse Lab in Milton Keynes and Alex Alderton, Roberto Amato, Jeffrey Barrett, Sonia Goncalves, Ewan Harrison, David K. Jackson, Ian Johnston, Dominic Kwiatkowski, Cordelia Langford, John Sillitoe on behalf of the Wellcome Sanger Institute COVID-19 Surveillance Team

EPI_ISL_2651661, EPI_ISL_2651697, EPI_ISL_2651847, EPI_ISL_2651867, EPI_ISL_2651892, EPI_ISL_2651893, EPI_ISL_2651894, EPI_ISL_2651895, EPI_ISL_2651897, EPI_ISL_2651898, EPI_ISL_2651900, EPI_ISL_2651901, EPI_ISL_2651902, EPI_ISL_2651903, EPI_ISL_2651904, EPI_ISL_2651905, EPI_ISL_2651907, EPI_ISL_2651908, EPI_ISL_2651909, EPI_ISL_2651910, EPI_ISL_2651911, EPI_ISL_2651912, EPI_ISL_2651913, EPI_ISL_2651915, EPI_ISL_2651916, EPI_ISL_2651917, EPI_ISL_2651918, EPI_ISL_2651920, EPI_ISL_2651921, EPI_ISL_2651922, EPI_ISL_2651923, EPI_ISL_2651924, EPI_ISL_2651925, EPI_ISL_2651926, EPI_ISL_2651927, EPI_ISL_2651928, EPI_ISL_2651931, EPI_ISL_2651932, EPI_ISL_2651933, EPI_ISL_2651934, EPI_ISL_2651935, EPI_ISL_2651936, EPI_ISL_2651937, EPI_ISL_2651939, EPI_ISL_2651940, EPI_ISL_2651941			
see above	Lighthouse Lab in Glasgow	Wellcome Sanger Institute for the COVID-19 Genomics UK (COG-UK) Consortium	Harper VanSteenhouse, Yumi Kasai, David Gray, Carol Clugston, Anna Dominiczak and Alex Alderton, Roberto Amato, Jeffrey Barrett, Sonia Goncalves, Ewan Harrison, David K. Jackson, Ian Johnston, Dominic Kwiatkowski, Cordelia Langford, John Sillitoe on behalf of the Wellcome Sanger Institute COVID-19 Surveillance Team
EPI_ISL_2651943	Lighthouse Laboratory Plymouth	Wellcome Sanger Institute for the COVID-19 Genomics UK (COG-UK) Consortium	Lighthouse Laboratory Plymouth and Alex Alderton, Roberto Amato, Jeffrey Barrett, Sonia Goncalves, Ewan Harrison, David K. Jackson, Ian Johnston, Dominic Kwiatkowski, Cordelia Langford, John Sillitoe on behalf of the Wellcome Sanger Institute COVID-19 Surveillance Team
EPI_ISL_2651947	Lighthouse Lab in Alderley Park	Wellcome Sanger Institute for the COVID-19 Genomics UK (COG-UK) Consortium	Jacquelyn Wynn, Mairead Hyland, The Lighthouse Lab in Alderley Park and Alex Alderton, Roberto Amato, Jeffrey Barrett, Sonia Goncalves, Ewan Harrison, David K. Jackson, Ian Johnston, Dominic Kwiatkowski, Cordelia Langford, John Sillitoe on behalf of the Wellcome Sanger Institute COVID-19 Surveillance Team
EPI_ISL_2663219	HealthPartners Central Lab	Minnesota Department of Health, Public Health Laboratory	Alexandra Lorentz, Jacob Garfin, Matt Plumb, and Xiong Wang
EPI_ISL_2663553	NYU Langone Health	Departments of Pathology and Medicine, New York University School of Medicine	Adriana Heguy, Dacia Dimartino, Emily Guzman, Christian Marier, Peter Meyn, Sitharam Ramaswami, Gael Westby, Paul Zappile, Yutong Zhang, Paolo Cotzia, Guiqing Wang
EPI_ISL_2663880	Lighthouse Lab in Milton Keynes	Wellcome Sanger Institute for the COVID-19 Genomics UK (COG-UK) Consortium	The Lighthouse Lab in Milton Keynes and Alex Alderton, Roberto Amato, Jeffrey Barrett, Sonia Goncalves, Ewan Harrison, David K. Jackson, Ian Johnston, Dominic Kwiatkowski, Cordelia Langford, John Sillitoe on behalf of the Wellcome Sanger Institute COVID-19 Surveillance Team
EPI_ISL_2664104, EPI_ISL_2664123, EPI_ISL_2664125, EPI_ISL_2664140, EPI_ISL_2664166, EPI_ISL_2664168, EPI_ISL_2664170, EPI_ISL_2664188, EPI_ISL_2664194, EPI_ISL_2664198, EPI_ISL_2664201, EPI_ISL_2664205, EPI_ISL_2664209, EPI_ISL_2664212, EPI_ISL_2664301, EPI_ISL_2664304, EPI_ISL_2664319, EPI_ISL_2664346, EPI_ISL_2664366, EPI_ISL_2664369, EPI_ISL_2664373, EPI_ISL_2664389, EPI_ISL_2664390, EPI_ISL_2664392, EPI_ISL_2664397, EPI_ISL_2664401, EPI_ISL_2664408, EPI_ISL_2664422			
see above	Lighthouse Lab in Alderley Park	Wellcome Sanger Institute for the COVID-19 Genomics UK (COG-UK) Consortium	Jacquelyn Wynn, Mairead Hyland, The Lighthouse Lab in Alderley Park and Alex Alderton, Roberto Amato, Jeffrey Barrett, Sonia Goncalves, Ewan Harrison, David K. Jackson, Ian Johnston, Dominic Kwiatkowski, Cordelia Langford, John Sillitoe on behalf of the Wellcome Sanger Institute COVID-19 Surveillance Team
EPI_ISL_2664423	Lighthouse Lab in Milton Keynes	Wellcome Sanger Institute for the COVID-19 Genomics UK (COG-UK) Consortium	The Lighthouse Lab in Milton Keynes and Alex Alderton, Roberto Amato, Jeffrey Barrett, Sonia Goncalves, Ewan Harrison, David K. Jackson, Ian Johnston, Dominic Kwiatkowski, Cordelia Langford, John Sillitoe on behalf of the Wellcome Sanger Institute COVID-19 Surveillance Team
EPI_ISL_2664440, EPI_ISL_2664549, EPI_ISL_2664566, EPI_ISL_2664584, EPI_ISL_2664680, EPI_ISL_2664706, EPI_ISL_2664709, EPI_ISL_2665042, EPI_ISL_2665225, EPI_ISL_2665260, EPI_ISL_2665296			
see above	Lighthouse Lab in Alderley Park	Wellcome Sanger Institute for the COVID-19 Genomics UK (COG-UK) Consortium	Jacquelyn Wynn, Mairead Hyland, The Lighthouse Lab in Alderley Park and Alex Alderton, Roberto Amato, Jeffrey Barrett, Sonia Goncalves, Ewan Harrison, David K. Jackson, Ian Johnston, Dominic Kwiatkowski, Cordelia Langford, John Sillitoe on behalf of the Wellcome Sanger Institute COVID-19 Surveillance Team
EPI_ISL_2665447, EPI_ISL_2665873, EPI_ISL_2666131, EPI_ISL_2666384, EPI_ISL_2666515, EPI_ISL_2666744, EPI_ISL_2666809, EPI_ISL_2666828, EPI_ISL_2666913, EPI_ISL_2667010, EPI_ISL_2667233, EPI_ISL_2667607, EPI_ISL_2667871, EPI_ISL_2668328, EPI_ISL_2668510, EPI_ISL_2668554, EPI_ISL_2668747, EPI_ISL_2668987, EPI_ISL_2669191, EPI_ISL_2669196, EPI_ISL_2669198, EPI_ISL_2669409, EPI_ISL_2669604, EPI_ISL_2669906, EPI_ISL_2670378, EPI_ISL_2670389, EPI_ISL_2670699, EPI_ISL_2670831, EPI_ISL_2671046			
see above	Department of Bacteria, Parasites and Fungi, Statens Serum Institut, Copenhagen, Denmark	Statens Serum Institut Bioinformatics and Microbial Genomics	Danish Covid-19 Genome Consortium
EPI_ISL_2671462	Center for Laboratory Medicine	Center for Laboratory Medicine	Yannick Gerth
EPI_ISL_2673381, EPI_ISL_2673385	Dutch COVID-19 response team	National Institute for Public Health and the Environment (RIVM)	Adam Meijer, Harry Vennema, Dirk Eggink, Jeroen Cremer, Sharon van den Brink, Bas van der Veer, AnneMarie van den Brandt, Lisa Wijsman, Kim Freriks, Ryanne Jaarsma, Euníce Then, Lynn Aarts, Sanne Bos, Melissa van Tuil, Linda van de Nes, Florian Zwagemaker, Dennis Schmitz, Annelies Kroneman, Karim Hajji, Chantal Reusken, on behalf of the national COVID-19 response team
EPI_ISL_2673676, EPI_ISL_2673677, EPI_ISL_2673678	OSPEDALE CIVILE TERAMO - CENTRO TRASFUSIONALE	Istituto Zooprofilattico Sperimentale dell'Abruzzo e Molise "G. Caporale"	Lorusso A, Marcacci M, Di Domenico M, Ancora M, Curini V, Di Lollo Valeria, Mangone I, Rinaldi A, Delli Compagni E, Scialabba S, Caporale M, Di Pasquale A, Cammà C, Puglia I, Calistri P, Savini G
EPI_ISL_2674808	SARS-CoV-2 testing team, National Institute of Infectious Diseases	Pathogen Genomics Center, National Institute of Infectious Diseases	Tsuyoshi Sekizuka, Kentaro Itokawa, Rina Tanaka, Masanori Hashino, Nozomu Hanaoka, Masumichi Saito, Naomi Nojiri, Hazuka Y Furihata, Hiromizu Takahashi, Tsuguto Fujimoto, Makoto Kuroda
EPI_ISL_2675078	Vestfold Hospital, Toensberg Department of Microbiology	Norwegian Institute of Public Health, Department of Virology	Kathrine Stene-Johansen, Kamilla Heddeland Instefjord, Hilde Elshaug, Garcia Llorente Ignacio, Jon Bråte, Engebretsen Serina Beate,Pedersen Benedikte Nevjen, Line Victoria Moen, Debech Nadia, Atiya R Ali,Marie Paulsen Madsen, Rasmus Riis Kopperud, Hilde Vollan, Karoline Bragstad, Olav Hungnes
EPI_ISL_2675096	Furst Medical Laboratory	Norwegian Institute of Public Health, Department of Virology	Kathrine Stene-Johansen, Kamilla Heddeland Instefjord, Hilde Elshaug, Garcia Llorente Ignacio, Jon Bråte, Engebretsen Serina Beate,Pedersen Benedikte Nevjen, Line Victoria Moen, Debech Nadia, Atiya R Ali,Marie Paulsen Madsen, Rasmus Riis Kopperud, Hilde Vollan, Karoline Bragstad, Olav Hungnes
EPI_ISL_2675204	Houston Health Dept.	Houston Health Dept.	Ryker Penn, Pamela Brown, Adolpho Lara, Yanlai Lai
EPI_ISL_2675462	Department of Bacteria, Parasites and Fungi, Statens Serum Institut, Copenhagen, Denmark	Statens Serum Institut Bioinformatics and Microbial Genomics	Danish Covid-19 Genome Consortium
EPI_ISL_2678743	Klinikum Ernst von Bergmann gemeinnützige GmbH - stationärer Bereich	Robert Koch Institute	unknown
EPI_ISL_2681473, EPI_ISL_2681474	University Hospitals of Geneva, Laboratory of Virology	HUG, Laboratory of Virology and the Health2030 Genome Center	Samuel Cordey, Ana Rita Goncalves, Laurent Kaiser, Lorenzo Cerutti, Henri Pegeot, Melyssa Elies, Deborah Penet, Keith Harshman, Ioannis Xenarios, Emmanouil Dermitzakis
EPI_ISL_2681759, EPI_ISL_2681767	Lighthouse Lab in Alderley Park	Wellcome Sanger Institute for the COVID-19 Genomics UK (COG-UK) Consortium	Jacquelyn Wynn, Mairead Hyland, The Lighthouse Lab in Alderley Park and Alex Alderton, Roberto Amato, Jeffrey Barrett, Sonia Goncalves, Ewan Harrison, David K. Jackson, Ian Johnston, Dominic Kwiatkowski, Cordelia Langford, John Sillitoe on behalf of the Wellcome Sanger Institute COVID-19 Surveillance Team
EPI_ISL_2682063, EPI_ISL_2682198	Lighthouse Lab in Glasgow	Wellcome Sanger Institute for the COVID-19 Genomics UK (COG-UK) Consortium	Harper VanSteenhouse, Yumi Kasai, David Gray, Carol Clugston, Anna Dominiczak and Alex Alderton, Roberto Amato, Jeffrey Barrett, Sonia Goncalves, Ewan Harrison, David K. Jackson, Ian Johnston, Dominic Kwiatkowski, Cordelia Langford, John Sillitoe on behalf of the Wellcome Sanger Institute COVID-19 Surveillance Team
EPI_ISL_2682388, EPI_ISL_2682402, EPI_ISL_2682433, EPI_ISL_2682562, EPI_ISL_2682662, EPI_ISL_2682666, EPI_ISL_2682802, EPI_ISL_2682804, EPI_ISL_2682812, EPI_ISL_2682816, EPI_ISL_2682818, EPI_ISL_2682821, EPI_ISL_2682822, EPI_ISL_2682823, EPI_ISL_2682825, EPI_ISL_2682830, EPI_ISL_2682832, EPI_ISL_2682838, EPI_ISL_2682843, EPI_ISL_2682844, EPI_ISL_2682854, EPI_ISL_2682856, EPI_ISL_2682857, EPI_ISL_2682862, EPI_ISL_2682864, EPI_ISL_2682865, EPI_ISL_2682866, EPI_ISL_2682876, EPI_ISL_2682882, EPI_ISL_2682884, EPI_ISL_2682890, EPI_ISL_2682893, EPI_ISL_2682897, EPI_ISL_2682898			
see above	Lighthouse Lab in Alderley Park	Wellcome Sanger Institute for the COVID-19 Genomics UK (COG-UK) Consortium	Jacquelyn Wynn, Mairead Hyland, The Lighthouse Lab in Alderley Park and Alex Alderton, Roberto Amato, Jeffrey Barrett, Sonia Goncalves, Ewan Harrison, David K. Jackson, Ian Johnston, Dominic Kwiatkowski, Cordelia Langford, John Sillitoe on behalf of the Wellcome Sanger Institute COVID-19 Surveillance Team
EPI_ISL_2683070, EPI_ISL_2683435, EPI_ISL_2683468, EPI_ISL_2683483	Lighthouse Lab in Glasgow	Wellcome Sanger Institute for the COVID-19 Genomics UK (COG-UK) Consortium	Harper VanSteenhouse, Yumi Kasai, David Gray, Carol Clugston, Anna Dominiczak and Alex Alderton, Roberto Amato, Jeffrey Barrett, Sonia Goncalves, Ewan Harrison, David K. Jackson, Ian Johnston, Dominic Kwiatkowski, Cordelia Langford, John Sillitoe on behalf of the Wellcome Sanger Institute COVID-19 Surveillance Team
EPI_ISL_2686643, EPI_ISL_2686652, EPI_ISL_2686653, EPI_ISL_2686660, EPI_ISL_2686666, EPI_ISL_2686670, EPI_ISL_2686673, EPI_ISL_2686674, EPI_ISL_2686676, EPI_ISL_2686679, EPI_ISL_2686685, EPI_ISL_2686686, EPI_ISL_2686687, EPI_ISL_2686688, EPI_ISL_2686692, EPI_ISL_2686693			
see above	Fulgent Genetics	Centers for Disease Control and Prevention Division of Viral Diseases, Pathogen Discovery	Dakota Howard, Dhwani Batra, Peter W. Cook, Kara Moser, Adrian Paskey, Jason Caravas, Benjamin Rambo-Martin, Shatavia Morrison, Christopher Gulvick, Scott Sammons, Yvette Unoarumhi, Darlene Wagner, Matthew Schmerer, Harry Gao, Mickey Li, John Gao, Joseph Fierro, Benafsh Sapra, Becky Tsai, Yan Meng, Doreen Ng, James Xie, Clinton R. Paden, Duncan MacCannell
EPI_ISL_2687996	National Institute of Infectious Diseases-Prof. Dr. Matei Bals Molecular Diagnostics Laboratory	National Institute of Infectious Diseases-Prof. Dr. Matei Bals Molecular Diagnostics Laboratory	Corina Casangiu, Leontina Banica, Marius Surleac, Petre Milu, Robert Hohan, Simona Paraschiv, Dan Otelea
EPI_ISL_2693033	Oregon State Public Health Laboratory	Oregon State Public Health Laboratory	Rafia Razzaque, Eugene Yeboah, Vanda Makris, Laura Tsaknaridis, John Fontana and Shane Sevey

EPI_ISL_2693086	NHLS_VIRO	KRISP, KZN Research Innovation and Sequencing Platform	Giandhari Jennifer, Pillay Sureshnee, Yajna Ramphal, Naidoo Yeshnee, Tshabuila Derek,Tegally Houriiyah, San James, Wilkinson Eduan, de Oliveira Tulio
EPI_ISL_2693271, EPI_ISL_2693272, EPI_ISL_2693274	UW Virology Lab	UW Virology Lab	Pavitra Roychoudhury, Hong Xie, Lasata Shrestha, Tien V. Nguyen, Shah Mohamed Bakhash, Michelle Lin, Noah R. Baker, Ricardo Perez, Sean Ellis, Nathan Breit, Robert J. Livingston, Meei-Li Huang, Keith R Jerome, Patrick Mathias, Alexander Greninger
EPI_ISL_2694808, EPI_ISL_2694821, EPI_ISL_2694832, EPI_ISL_2694847, EPI_ISL_2694859	Labor Berlin Charite Vivantes GmbH / Institut fur Virologie	Charite Universitatsmedizin Berlin, Institut fur Virologie/Labor Berlin	Peter Menzel, Christine Stephan, Rolf Schwarzer, Victor M Corman, Barbara Muhlemann, Terry Jones, Christian Drosten
EPI_ISL_2694898, EPI_ISL_2694899	H Santarem	Instituto Nacional de Saude (INSA)	Borges et al
EPI_ISL_2695088	H Vila Franca Xira	Instituto Nacional de Saude (INSA)	Borges et al
EPI_ISL_2695162	CH Setubal	Instituto Nacional de Saude (INSA)	Borges et al
EPI_ISL_2695206, EPI_ISL_2695207, EPI_ISL_2695208, EPI_ISL_2695209, EPI_ISL_2695210, EPI_ISL_2695211, EPI_ISL_2695212, EPI_ISL_2695214, EPI_ISL_2695215	ULS Litoral Alentejano	Instituto Nacional de Saude (INSA) and Institute of Biomedicine (iBiMed), Universidade de Aveiro	Borges et al
EPI_ISL_2695257	SESARAM	Instituto Nacional de Saude (INSA)	Borges et al
EPI_ISL_2695268	CHU Sao Joao, Porto	Instituto Nacional de Saude (INSA)	Borges et al
EPI_ISL_2695287	H Braga	Instituto Nacional de Saude (INSA)	Borges et al
EPI_ISL_2695295, EPI_ISL_2695296, EPI_ISL_2695297	CHTMAD	Instituto Nacional de Saude (INSA)	Borges et al
EPI_ISL_2695358, EPI_ISL_2695359	Lab Dra Celeste Formosinho	Instituto Nacional de Saude (INSA)	Borges et al
EPI_ISL_2695381, EPI_ISL_2695383, EPI_ISL_2695384, EPI_ISL_2695385, EPI_ISL_2695387	Labeto - CAB - Leiria	Instituto Nacional de Saude (INSA)	Borges et al
EPI_ISL_2695403	Lab La Salete Robles - VN Famalicao	Instituto Nacional de Saude (INSA)	Borges et al
EPI_ISL_2695416	H Guimaraes	Instituto Nacional de Saude (INSA)	Borges et al
EPI_ISL_2695466, EPI_ISL_2695467, EPI_ISL_2695468, EPI_ISL_2695498, EPI_ISL_2695499, EPI_ISL_2695500	ARS Algarve - Laboratorio Laura Ayres	Instituto Nacional de Saude (INSA)	Borges et al
EPI_ISL_2698595	WHO National Influenza Centre Russian Federation	WHO National Influenza Centre Russian Federation	Andrey Komissarov, Artem Fadeev, Kseniya Komissarova, Oula Mansour, Kirill Varchenko, Mikhail Bakaev, Tamila Musaeva, Veronika Eder, Maria Pisareva, Nikita Yolshin, Daria Danilenko, Ksenia Safina, Elena Nabieva, Georgii Bazykin, Dmitry Lioznov
EPI_ISL_2700097, EPI_ISL_2700999, EPI_ISL_2701131	Lighthouse Lab in Alderley Park	Wellcome Sanger Institute for the COVID-19 Genomics UK (COG-UK) Consortium	Jacquelyn Wynn, Mairead Hyland, The Lighthouse Lab in Alderley Park and Alex Alderton, Roberto Amato, Jeffrey Barrett, Sonia Goncalves, Ewan Harrison, David K. Jackson, Ian Johnston, Dominic Kwiatkowski, Cordelia Langford, John Sillitoe on behalf of the Wellcome Sanger Institute COVID-19 Surveillance Team
EPI_ISL_2701140	Lighthouse Lab in Glasgow	Wellcome Sanger Institute for the COVID-19 Genomics UK (COG-UK) Consortium	Harper VanSteenhouse, Yumi Kasai, David Gray, Carol Clugston, Anna Dominiczak and Alex Alderton, Roberto Amato, Jeffrey Barrett, Sonia Goncalves, Ewan Harrison, David K. Jackson, Ian Johnston, Dominic Kwiatkowski, Cordelia Langford, John Sillitoe on behalf of the Wellcome Sanger Institute COVID-19 Surveillance Team
EPI_ISL_2701344, EPI_ISL_2701352, EPI_ISL_2701377, EPI_ISL_2701392, EPI_ISL_2701412, EPI_ISL_2701419, EPI_ISL_2701503, EPI_ISL_2701510, EPI_ISL_2701524, EPI_ISL_2701566, EPI_ISL_2701573, EPI_ISL_2701598, EPI_ISL_2701603, EPI_ISL_2701607, EPI_ISL_2701611, EPI_ISL_2701717, EPI_ISL_2701720, EPI_ISL_2701749, EPI_ISL_2701777, EPI_ISL_2701782, EPI_ISL_2701795, EPI_ISL_2701808, EPI_ISL_2701809, EPI_ISL_2701815, EPI_ISL_2701820, EPI_ISL_2701821, EPI_ISL_2701853, EPI_ISL_2701854, EPI_ISL_2701883, EPI_ISL_2701888, EPI_ISL_2701941, EPI_ISL_2701973, EPI_ISL_2701979, EPI_ISL_2701987, EPI_ISL_2701993, EPI_ISL_2701996, EPI_ISL_2702022, EPI_ISL_2702024, EPI_ISL_2702035, EPI_ISL_2702482, EPI_ISL_2702487, EPI_ISL_2702607, EPI_ISL_2702610, EPI_ISL_2702611, EPI_ISL_2702630, EPI_ISL_2702631, EPI_ISL_2702661, EPI_ISL_2702665, EPI_ISL_2702689, EPI_ISL_2702706, EPI_ISL_2702708, EPI_ISL_2702713, EPI_ISL_2702717, EPI_ISL_2702722, EPI_ISL_2702728, EPI_ISL_2702738, EPI_ISL_2702812, EPI_ISL_2703186, EPI_ISL_2703199, EPI_ISL_2703300, EPI_ISL_2703353, EPI_ISL_2703387, EPI_ISL_2703388, EPI_ISL_2703390, EPI_ISL_2703785, EPI_ISL_2703793			
see above	Lighthouse Lab in Alderley Park	Wellcome Sanger Institute for the COVID-19 Genomics UK (COG-UK) Consortium	Jacquelyn Wynn, Mairead Hyland, The Lighthouse Lab in Alderley Park and Alex Alderton, Roberto Amato, Jeffrey Barrett, Sonia Goncalves, Ewan Harrison, David K. Jackson, Ian Johnston, Dominic Kwiatkowski, Cordelia Langford, John Sillitoe on behalf of the Wellcome Sanger Institute COVID-19 Surveillance Team
EPI_ISL_2703804	Lighthouse Lab in Milton Keynes	Wellcome Sanger Institute for the COVID-19 Genomics UK (COG-UK) Consortium	The Lighthouse Lab in Milton Keynes and Alex Alderton, Roberto Amato, Jeffrey Barrett, Sonia Goncalves, Ewan Harrison, David K. Jackson, Ian Johnston, Dominic Kwiatkowski, Cordelia Langford, John Sillitoe on behalf of the Wellcome Sanger Institute COVID-19 Surveillance Team
EPI_ISL_2704283, EPI_ISL_2704287, EPI_ISL_2704424, EPI_ISL_2704491, EPI_ISL_2704668	Lighthouse Lab in Alderley Park	Wellcome Sanger Institute for the COVID-19 Genomics UK (COG-UK) Consortium	Jacquelyn Wynn, Mairead Hyland, The Lighthouse Lab in Alderley Park and Alex Alderton, Roberto Amato, Jeffrey Barrett, Sonia Goncalves, Ewan Harrison, David K. Jackson, Ian Johnston, Dominic Kwiatkowski, Cordelia Langford, John Sillitoe on behalf of the Wellcome Sanger Institute COVID-19 Surveillance Team
EPI_ISL_2705571	University College London, Great Ormond Street Hospital for Children NHS Foundation Trust, Imperial College Healthcare NHS Trust	COVID-19 Genomics UK (COG-UK) Consortium	Sergi Castellano, Rachel Williams, Mark Kristiansen, Paola Resende Silva, Sunando Roy, Tony Brooks, Helena Tutill, Paola Niola, Patricia Dyal, Charlotte Williams, Leysa Forrest, Yasmin Panchbhaya, Jacqueline Findlay, Samuel Weeks, Julianne Brown, Kathryn Harris, Paul Randell, James Price, Alison Holmes, Judith Breuer
EPI_ISL_2705575	Centers for Disease Control, R.O.C. (Taiwan)	Centers for Disease Control, R.O.C. (Taiwan)	Ji-Rong Yang, Yu-Chi-Lin, Jung-Jung Mu, Ming-Tsan-Liu
EPI_ISL_2705590, EPI_ISL_2705700, EPI_ISL_2705702, EPI_ISL_2705705, EPI_ISL_2705706, EPI_ISL_2705785	University College London, Great Ormond Street Hospital for Children NHS Foundation Trust, Imperial College Healthcare NHS Trust	COVID-19 Genomics UK (COG-UK) Consortium	Sergi Castellano, Rachel Williams, Mark Kristiansen, Paola Resende Silva, Sunando Roy, Tony Brooks, Helena Tutill, Paola Niola, Patricia Dyal, Charlotte Williams, Leysa Forrest, Yasmin Panchbhaya, Jacqueline Findlay, Samuel Weeks, Julianne Brown, Kathryn Harris, Paul Randell, James Price, Alison Holmes, Judith Breuer
EPI_ISL_2706170, EPI_ISL_2706177, EPI_ISL_2706187, EPI_ISL_2706263, EPI_ISL_2706285, EPI_ISL_2706418, EPI_ISL_2706444, EPI_ISL_2706475, EPI_ISL_2706494	Northumbria University / South Tees Hospitals NHS Foundation Trust / North Cumbria Integrated Care NHS Foundation Trust / North Tees and Hartlepool NHS Foundation Trust / Newcastle Hospitals NHS Foundation Trust	COVID-19 Genomics UK (COG-UK) Consortium	Darren L Smith,Andrew Nelson,Matthew Bashton,Greg R Young,Joshua Loh,John Allan,Mohammad A Tariq,Giles S Holt,Gary Black,Wen C Yew,Lynn Dover,Paul Baker,Steve Liggett,Sarah Essex,Jane Greenaway,Debra Padgett,Clive Graham,Garren Scott,Edward Barton,Emma Swindells,Brendan Payne,Jennifer Collins,Yusri Taha,Gary Eltringham
EPI_ISL_2707798, EPI_ISL_2707908, EPI_ISL_2707910, EPI_ISL_2707911, EPI_ISL_2707912, EPI_ISL_2707913, EPI_ISL_2707915, EPI_ISL_2707918, EPI_ISL_2707919, EPI_ISL_2707920, EPI_ISL_2707923, EPI_ISL_2707925, EPI_ISL_2707927, EPI_ISL_2707933, EPI_ISL_2707937, EPI_ISL_2707940, EPI_ISL_2707943, EPI_ISL_2707946, EPI_ISL_2707952, EPI_ISL_2707953, EPI_ISL_2707954, EPI_ISL_2707957, EPI_ISL_2707959, EPI_ISL_2707960, EPI_ISL_2707964, EPI_ISL_2707970, EPI_ISL_2707981, EPI_ISL_2707984, EPI_ISL_2707986, EPI_ISL_2708008, EPI_ISL_2708010, EPI_ISL_2708022, EPI_ISL_2708023, EPI_ISL_2708024, EPI_ISL_2708026, EPI_ISL_2708032, EPI_ISL_2708044, EPI_ISL_2708046, EPI_ISL_2708047, EPI_ISL_2708048, EPI_ISL_2708104, EPI_ISL_2708105, EPI_ISL_2708113, EPI_ISL_2708114, EPI_ISL_2708116, EPI_ISL_2708120, EPI_ISL_2708130, EPI_ISL_2708136, EPI_ISL_2708137			
see above	Originating lab: Wales Specialist Virology Centre Sequencing lab: Pathogen Genomics Unit	Public Health Wales Microbiology Cardiff Wales Specialist Virology Centre	Catherine Moore, Johnathan Evans, Laura Gifford, Malorie Perry, Simon Cottrell, Angela Marchbank, Alec Birchley, Alexander Adams, Amy Gaskin, Bree Gatica-Wilcox, Jason Coombes, Joel Southgate, Lauren Gilbert, Lee Graham, Nicole Pacchiarini, Sara Kumziene-Summerhayes, Sarah Taylor, Sophie Jones, Sara Rey, Matthew Bull, Joanne Watkins, Sally Corden, Tom Connor
EPI_ISL_2709441	West Java Health Laboratory	Eijkman Institute for Molecular Biology, National Research and Innovation Agency; West Java Health Laboratory	Lydia V. Panggalo, Iskandar Adnan, Sukma Oktavianthi, Lidwina Priliani, Edison Johar, Frilasita A Yudhaputri, Muhammad Rezki Rasyak, Willy Agustine, Hidayat Trimarsanto, Azzania Fibriani, Ema Rahmawati, Ryan Bayusantika Ristandi, Rifky Waluyajati Rachman, Cut Nur Cinthia Alamanda, Safarina G Malik, Khin Saw Myint, Amin Soebandrio
EPI_ISL_2709442	West Java Health Laboratory	Eijkman Institute for Molecular Biology, National Research and Innovation Agency; West Java Health Laboratory	Iskandar Adnan, Sukma Oktavianthi, Lidwina Priliani, Edison Johar, Frilasita A Yudhaputri, Muhammad Rezki Rasyak, Willy Agustine, Hidayat Trimarsanto, Lydia V. Panggalo, Azzania Fibriani, Ema Rahmawati, Ryan Bayusantika Ristandi, Rifky Waluyajati Rachman, Cut Nur Cinthia Alamanda, Safarina G Malik, Khin Saw Myint, Amin Soebandrio

EPI_ISL_2709736, EPI_ISL_2709737	National Institute of Public Health	State Veterinary Institute Prague	Nagy,A;Cernikov,L;Stara,M;Suri,T;Vecerova,J;Jirincova,H
EPI_ISL_2710258	NHLS Charlotte Maxeke Johannesburg Academic Hospital and the University of the Witwatersrand	KRISP, KZN Research Innovation and Sequencing Platform	Florette Treurnicht, Bulelani Manene, Kathleen Subramoney, Giandhari Jennifer, Pillay Sureshnee, Yajna Ramphal, Naidoo Yeshnee, Tshabuila Derek,Tegally Houriiyah, San James, Wilkinson Eduan, de Oliveira Tulio
EPI_ISL_2712849, EPI_ISL_2712854, EPI_ISL_2712856	Hospital Universitari Vall d'Hebron - Vall d'Hebron Institut de Recerca	Hospital Universitari Vall d'Hebron - Vall d'Hebron Institut de Recerca	Cristina Andrés, Maria Piñana, Alejandra González-Sánchez, Damir Garcia-Cehic, Ariadna Rando, Juliana Esperalba, Maria Gema Codina, Carla Castillo, Maria Carmen Martin, Tomàs Pumarola, Josep Quer, Andrés Antón
EPI_ISL_2712939	Wyoming Public Health Laboratory	Wyoming Public Health Laboratory	Jim Mildenberger, Taylor Fearing, Channing Weber, Ashley Norberg, Chayse Rowley, Marley Goetz, Brian Dominguez, Elliot Thomasson, Sam Britz, Cari Sloma, Robert Petit, and Rob Christensen
EPI_ISL_2716210	The Jackson Laboratory	The Jackson Laboratory	Long J, Renzette N, Adams M, Omerza G, Kelly K, Li L
EPI_ISL_2716404	Arizona State Public Health Laboratory	Arizona State Public Health Laboratory	Trung Huynh, Jessica Escobar, Katherine Fullerton, Nobuko Fukushima, Matthew Contursi, Stacy White, Linda Getsinger, Victor Waddell
EPI_ISL_2716468	Washington State Department of Health Public Health Laboratories	Washington State Department of Health Public Health Laboratories	Drew MacKellar, Philip Dykema, Denny Russell, Joenice Gonzalez, Hannah Gray, Geoff Melly, Vanessa De Los Santos, Darren Lucas, JohnAric Peterson, Avi Singh, Rebecca Cao
EPI_ISL_2717791, EPI_ISL_2718283	Lighthouse Lab in Alderley Park	Wellcome Sanger Institute for the COVID-19 Genomics UK (COG-UK) Consortium	Jacquelyn Wynn, Mairead Hyland, The Lighthouse Lab in Alderley Park and Alex Alderton, Roberto Amato, Jeffrey Barrett, Sonia Goncalves, Ewan Harrison, David K. Jackson, Ian Johnston, Dominic Kwiatkowski, Cordelia Langford, John Sillitoe on behalf of the Wellcome Sanger Institute COVID-19 Surveillance Team
EPI_ISL_2718295, EPI_ISL_2718297, EPI_ISL_2718376, EPI_ISL_2718377, EPI_ISL_2718389, EPI_ISL_2718394, EPI_ISL_2718395, EPI_ISL_2718397, EPI_ISL_2718398, EPI_ISL_2718410, EPI_ISL_2718416, EPI_ISL_2718421, EPI_ISL_2718425, EPI_ISL_2718429, EPI_ISL_2718435, EPI_ISL_2718438, EPI_ISL_2718439, EPI_ISL_2718440, EPI_ISL_2718445, EPI_ISL_2718446, EPI_ISL_2718451, EPI_ISL_2718453, EPI_ISL_2718458, EPI_ISL_2718462, EPI_ISL_2718466, EPI_ISL_2718467, EPI_ISL_2718470, EPI_ISL_2718477, EPI_ISL_2718478, EPI_ISL_2718480, EPI_ISL_2718486, EPI_ISL_2718488			
see above	Lighthouse Lab in Glasgow	Wellcome Sanger Institute for the COVID-19 Genomics UK (COG-UK) Consortium	Harper VanSteenhouse, Yumi Kasai, David Gray, Carol Clugston, Anna Dominiczak and Alex Alderton, Roberto Amato, Jeffrey Barrett, Sonia Goncalves, Ewan Harrison, David K. Jackson, Ian Johnston, Dominic Kwiatkowski, Cordelia Langford, John Sillitoe on behalf of the Wellcome Sanger Institute COVID-19 Surveillance Team
EPI_ISL_2725550	Azienda Sanitaria dell'Alto Adige - Laboratorio Aziendale di Microbiologia e Virologia	Azienda Sanitaria dell'Alto Adige	Irene Bianconi
EPI_ISL_2728627	SYNLAB	GIGA Medical Genomics	Keith Durkin, Maria Artesi, Bouchra Boujemla, Nathalie Renotte, Nadine Cambisano, Sébastien Bontems, Cécile Meex, Claire Gourzonès, Olivier Ek, Laurent Gillet, Marie-Pierre Hayette, Vincent Bours
EPI_ISL_2731148, EPI_ISL_2731149, EPI_ISL_2731150, EPI_ISL_2731151, EPI_ISL_2731152, EPI_ISL_2731153, EPI_ISL_2731155, EPI_ISL_2731156, EPI_ISL_2731157, EPI_ISL_2731158, EPI_ISL_2731159, EPI_ISL_2731160, EPI_ISL_2731161, EPI_ISL_2731162, EPI_ISL_2731163, EPI_ISL_2731164, EPI_ISL_2731165, EPI_ISL_2731168, EPI_ISL_2731170, EPI_ISL_2731171, EPI_ISL_2731172, EPI_ISL_2731173, EPI_ISL_2731174, EPI_ISL_2731175, EPI_ISL_2731176, EPI_ISL_2731177, EPI_ISL_2731179, EPI_ISL_2731180, EPI_ISL_2731181, EPI_ISL_2731182, EPI_ISL_2731183, EPI_ISL_2731185, EPI_ISL_2731186, EPI_ISL_2731189, EPI_ISL_2731190, EPI_ISL_2731191, EPI_ISL_2731192, EPI_ISL_2731194, EPI_ISL_2731195, EPI_ISL_2731196, EPI_ISL_2731197, EPI_ISL_2731198, EPI_ISL_2731199, EPI_ISL_2731200, EPI_ISL_2731201, EPI_ISL_2731202, EPI_ISL_2731203, EPI_ISL_2731205, EPI_ISL_2731207, EPI_ISL_2731208, EPI_ISL_2731209, EPI_ISL_2731210, EPI_ISL_2731211, EPI_ISL_2731212, EPI_ISL_2731213, EPI_ISL_2731214, EPI_ISL_2731215, EPI_ISL_2731216, EPI_ISL_2731217, EPI_ISL_2731219, EPI_ISL_2731220, EPI_ISL_2731221, EPI_ISL_2731222, EPI_ISL_2731224, EPI_ISL_2731225, EPI_ISL_2731226, EPI_ISL_2731227, EPI_ISL_2731228, EPI_ISL_2731229, EPI_ISL_2731231, EPI_ISL_2731232, EPI_ISL_2731233, EPI_ISL_2731235, EPI_ISL_2731236, EPI_ISL_2731237, EPI_ISL_2731238, EPI_ISL_2731240, EPI_ISL_2731241, EPI_ISL_2731242, EPI_ISL_2731243, EPI_ISL_2731245, EPI_ISL_2731246, EPI_ISL_2731247, EPI_ISL_2731248, EPI_ISL_2731250, EPI_ISL_2731251, EPI_ISL_2731252, EPI_ISL_2731254, EPI_ISL_2731255, EPI_ISL_2731256, EPI_ISL_2731261, EPI_ISL_2731262, EPI_ISL_2731263, EPI_ISL_2731264, EPI_ISL_2731265, EPI_ISL_2731266, EPI_ISL_2731267, EPI_ISL_2731268, EPI_ISL_2731269			
see above	Lighthouse Lab in Glasgow	Wellcome Sanger Institute for the COVID-19 Genomics UK (COG-UK) Consortium	Harper VanSteenhouse, Yumi Kasai, David Gray, Carol Clugston, Anna Dominiczak and Alex Alderton, Roberto Amato, Jeffrey Barrett, Sonia Goncalves, Ewan Harrison, David K. Jackson, Ian Johnston, Dominic Kwiatkowski, Cordelia Langford, John Sillitoe on behalf of the Wellcome Sanger Institute COVID-19 Surveillance Team
EPI_ISL_2735701, EPI_ISL_2735706	Oxford Viromics, NDM, University of Oxford; Oxford University Hospitals; Basingstoke and North Hampshire Hospital	COVID-19 Genomics UK (COG-UK) Consortium	Tanya Golubchik, David Bonsall, George Macintyre, Amy Trebes, Mariateresa de Cesare, Catrin Moore, Alex Mobbs, Anita Justice, Robert Shaw, Monique Andersson, Timothy Peto, Emma Wise, Nathan Moore, Jessica Lynch, Nick Cortes, Matilde Mori, Stephen Kidd, David Buck, John Todd, Christophe Fraser
EPI_ISL_2745817, EPI_ISL_2745851, EPI_ISL_2746004, EPI_ISL_2746007, EPI_ISL_2746023, EPI_ISL_2746026, EPI_ISL_2746045, EPI_ISL_2746046, EPI_ISL_2746060, EPI_ISL_2746066, EPI_ISL_2746070, EPI_ISL_2746080, EPI_ISL_2746099, EPI_ISL_2746406, EPI_ISL_2746532, EPI_ISL_2746543, EPI_ISL_2746616			
see above	Respiratory Virus Unit, Microbiology Services Colindale, Public Health England	COVID-19 Genomics UK (COG-UK) Consortium	PHE Covid Sequencing Team
EPI_ISL_2750042, EPI_ISL_2750043	Originating lab: Wales Specialist Virology Centre Sequencing lab: Pathogen Genomics Unit	Public Health Wales Microbiology Cardiff Wales Specialist Virology Centre	Catherine Moore, Johnathan Evans, Laura Gifford, Malorie Perry, Simon Cottrell, Angela Marchbank, Alec Birchley, Alexander Adams, Amy Gaskin, Bree Gatica-Wilcox, Jason Coombes, Joel Southgate, Lauren Gilbert, Lee Graham, Nicole Pacchiarini, Sara Kumziene-Summerhayes, Sarah Taylor, Sophie Jones, Sara Rey, Matthew Bull, Joanne Watkins, Sally Corden, Tom Connor
EPI_ISL_2754296	Arizona State Public Health Laboratory	Arizona State Public Health Laboratory	Trung Huynh, Jessica Escobar, Katherine Fullerton, Nobuko Fukushima, Matthew Contursi, Stacy White, Linda Getsinger, Victor Waddell
EPI_ISL_2757667, EPI_ISL_2757669, EPI_ISL_2757670	Hospital	National Reference Center for Viruses of Respiratory Infections, Institut Pasteur, Paris	Marion Barbet, Sylvie Behillil, Méline Bizard, Angela Brisebarre, Camille Capel, Vincent Enouf, Louise Lefrançois, Frédéric Lemoine, Christophe Malabat, Corinne Maufrais, Etienne Simon-Lorière, Maud Vanpeene, Sylvie Van der Werf ,JéRôMe Durivault
EPI_ISL_2757817	OSPEDALE CIVILE TERAMO - CENTRO TRASFUSIONALE	Istituto Zooprofilattico Sperimentale dell'Abruzzo e Molise "G. Caporale"	Lorusso A, Marcacci M, Di Domenico M, Ancora M, Curini V, Di Lollo Valeria, Mangone I, Rinaldi A, Delli Compagni E, Scialabba S, Caporale M, Di Pasquale A, Cammà C, Puglia I, Calistri P, Savini G
EPI_ISL_2758029, EPI_ISL_2758032, EPI_ISL_2758034, EPI_ISL_2758035, EPI_ISL_2758036	Austrian Agency for Health and Food Safety (AGES)	Bergthaler laboratory, CeMM Research Center for Molecular Medicine of the Austrian Academy of Sciences	Lukas Endler, Anna Schedl, Fabian Amman, Petr Triska, Matthew Thornton, Thomas Penz, Benedikt Agerer, Maelle Le Moing, Michael Schuster, Bekir Erguner, Jan Laine, Martin Senekowitsch, Christoph Bock, Andreas Bergthaler
EPI_ISL_2759829	Bioscientia Labor Wermsdorf	Robert Koch Institute	unknown
EPI_ISL_2759999	SYNLAB MVZ Weiden	Robert Koch Institute	unknown
EPI_ISL_2761070, EPI_ISL_2761093, EPI_ISL_2761292, EPI_ISL_2761400, EPI_ISL_2761413, EPI_ISL_2761460, EPI_ISL_2761477	Bioscientia Labor Wermsdorf	Robert Koch Institute	unknown
EPI_ISL_2761654	Landesgesundheitsamt Baden-Württemberg	Robert Koch Institute	unknown
EPI_ISL_2761673, EPI_ISL_2761682	MVZ Labor Dr. Fenner und Kollegen (Standort Hamburg)	Robert Koch Institute	unknown
EPI_ISL_2761692	Labor Dr. Heidrich & Kollegen MVZ GmbH Hamburg	Robert Koch Institute	unknown
EPI_ISL_2761846	CENTOGENE Frankfurt Laboratory: Niederlassung Industriepark Höchst	Robert Koch Institute	unknown
EPI_ISL_2761909	Medizinisch-Diagnostisches Labor Kempten allgäulab	Robert Koch Institute	unknown
EPI_ISL_2762082	Bioscientia Labor Wermsdorf	Robert Koch Institute	unknown
EPI_ISL_2768037	Clinical Virology	Clinical Bacteriology	Tim Roloff, Fanny Wegner, Helena MB Seth-Smith, Alfredo Mari, Karoline Leuzinger, Julia Bielicki, Manuel Battegay, Hans Hirsch, Adrian Egli
EPI_ISL_2774386, EPI_ISL_2774388, EPI_ISL_2774390	Illinois Department of Public Health - Springfield Lab	Illinois Department of Public Health - Springfield Lab	Bryan Sim, Gordon McCall

Supp. Table S5

We gratefully acknowledge the following Authors from the Originating laboratories responsible for obtaining the specimens, as well as the Submitting laboratories where the genome data were generated and shared via GISAID, on which this research is based.

All Submitters of data may be contacted directly via www.gisaid.org

Authors are sorted alphabetically.

Accession ID	Originating Laboratory	Submitting Laboratory	Authors
EPI_ISL_1993258	Fulgent Genetics	Centers for Disease Control and Prevention Division of Viral Diseases, Pathogen Discovery	Dakota Howard, Dhwani Batra, Peter W. Cook, Kara Moser, Adrian Paskey, Jason Caravas, Benjamin Rambo-Martin, Shatavia Morrison, Christopher Gulvick, Scott Sammons, Yvette Unoarumhi, Darlene Wagner, Matthew Schmerer, Harry Gao, Mickey Li, John Gao, Joseph Fierro, Benafsh Sapra, Becky Tsai, Yan Meng, Doreen Ng, James Xie, Clinton R. Paden, Duncan MacCannell
EPI_ISL_2018101	Wyoming Public Health Laboratory	Wyoming Public Health Laboratory	Jim Mildenberger, Taylor Fearing, Channing Weber, Ashley Norberg, Chayse Rowley, Marley Goetz, Brian Dominguez, Elliot Thomasson, Brittany Oher, Sam Britz, Cari Sloma, and Rob Christensen
EPI_ISL_2037398	Kansas Health and Environmental Lab	Kansas Health and Environmental Lab	Mike Grose, Jonathan Barnell, Ben Olsen, and Phil Adam
EPI_ISL_2043725	Fulgent Genetics	Centers for Disease Control and Prevention Division of Viral Diseases, Pathogen Discovery	Dakota Howard, Dhwani Batra, Peter W. Cook, Kara Moser, Adrian Paskey, Jason Caravas, Benjamin Rambo-Martin, Shatavia Morrison, Christopher Gulvick, Scott Sammons, Yvette Unoarumhi, Darlene Wagner, Matthew Schmerer, Harry Gao, Mickey Li, John Gao, Joseph Fierro, Benafsh Sapra, Becky Tsai, Yan Meng, Doreen Ng, James Xie, Clinton R. Paden, Duncan MacCannell
EPI_ISL_2046753, EPI_ISL_2046773	Laboratory Corporation of America	Centers for Disease Control and Prevention Division of Viral Diseases, Pathogen Discovery	Dakota Howard, Dhwani Batra, Peter W. Cook, Kara Moser, Adrian Paskey, Jason Caravas, Benjamin Rambo-Martin, Shatavia Morrison, Christopher Gulvick, Scott Sammons, Yvette Unoarumhi, Darlene Wagner, Matthew Schmerer, Minoo Agarwal, Eyad Almasri, Debbie Boles, Ayla Burns, Nuthawin Charoensri, Oren Cohen, Susan Countryman, Mary Ann Cristobal, Bobbi Croy, Suzanne Dale, Hrushikesh Deshmukh, Amanda Douglas, Vincent Drouillon, Marcia Eisenberg, Howard Engler, Rama Ghatti, Prashant Gupta, Susan Hicks, Jake Humphrey, Lax Iyer, Manoj Jain, Mohan Kolli, Brian Krueger, Tim Kuphal, Stanley Letovsky, Michael Levandoski, Craig Lukasik, Jonathan Meltzer, Brian Norvell, Mindy Nye, Scott Parker, Christos Petropoulos, John Pruitt, Steven Ragan, Scott Ryan, Mike Sapeta, Jana Schroth, Suresh Babu Selvaraju, Goran Stevovic, Amanda Suchanek, Andrea Throop, Lyndon Tilson, Thomas Urban, Joe Voshell, Kimberly Wagner, Jonathan Williams, Mary Williamson, Qian Zeng, Tricia Zwiefelhofer, Clinton R. Paden, Duncan MacCannell
EPI_ISL_2090645	Idaho Bureau of Laboratories	Idaho Bureau of Laboratories	R. Beukelman, Matthew Charles Burns, Aimee Ceniseros, Robert L. Voermans, Christopher Ball
EPI_ISL_2090896	Nebraska Public Health Laboratory	NPHL COVID-19 Response Team	NPHL COVID-19 Response Team
EPI_ISL_2096131	Washington State Department of Health Public Health Laboratories	Washington State Department of Health Public Health Laboratories	Drew MacKellar, Philip Dykema, Denny Russell, Joenice Gonzalez, Hannah Gray, Geoff Melly, Vanessa De Los Santos, Darren Lucas, JohnAric Peterson, Avi Singh, Rebecca Cao
EPI_ISL_2103887, EPI_ISL_2103888, EPI_ISL_2103893, EPI_ISL_2104402	Broad Institute Clinical Research Sequencing Platform	Infectious Disease Program, Broad Institute of Harvard and MIT	Siddle,K.J., Adams,G., Pearlman,L., Gladden-Young,A., Vicente,G., Blumenstiel,B., DeFelice,M., Lee,M., McGovern,S., Lagerborg,K., Rudy,M., DeRuff,K., Carter,A., Normandin,E., Bauer,M., Reilly,S., Tomkins-Tinch,C., Loreth,C., Chaluvadi,S., Meldrim,J., Granger,B., Lemieux,J.E., Birren,B.W., Sabeti,P.C., Larkin,K., Dodge,S., Lennon,N., Madoff,L., Brown,C., Gallagher,G., Smole,S., Park,D.J., Gabriel,S., and MacInnis,B.L.
EPI_ISL_2104891, EPI_ISL_2104953, EPI_ISL_2104994, EPI_ISL_2105012, EPI_ISL_2105016, EPI_ISL_2105042, EPI_ISL_2105093	UW Virology Lab	UW Virology Lab	Pavitra Roychoudhury, Hong Xie, Lasata Shrestha, Tien V. Nguyen, Shah Mohamed Bakhash, Michelle Lin, Noah R. Baker, Ricardo Perez, Sean Ellis, Nathan Breit, Robert J. Livingston, Meei-Li Huang, Keith R Jerome, Patrick Mathias, Alexander Greninger
EPI_ISL_2106441	Pandemic Response Lab - NYC	Pandemic Response Lab, R&D	Henry Lee, Michael Hammerling, Melissa Hopkins, Cybill del Castillo, Shinyoung Clair Kang, William Ward, Pradeep Bugga, Sol Rey, Dylan Law, Katharine Nelson, Haiping Hao, Jon Laurent
EPI_ISL_2134018, EPI_ISL_2134111, EPI_ISL_2134183, EPI_ISL_2134327, EPI_ISL_2134372, EPI_ISL_2134432	Fulgent Genetics	Centers for Disease Control and Prevention Division of Viral Diseases, Pathogen Discovery	Dakota Howard, Dhwani Batra, Peter W. Cook, Kara Moser, Adrian Paskey, Jason Caravas, Benjamin Rambo-Martin, Shatavia Morrison, Christopher Gulvick, Scott Sammons, Yvette Unoarumhi, Darlene Wagner, Matthew Schmerer, Harry Gao, Mickey Li, John Gao, Joseph Fierro, Benafsh Sapra, Becky Tsai, Yan Meng, Doreen Ng, James Xie, Clinton R. Paden, Duncan MacCannell
EPI_ISL_2135835	Arizona State Public Health Laboratory	Arizona State Public Health Laboratory	Trung Huynh, Jessica Escobar, Katherine Fullerton, Nobuko Fukushima, Stacy White, Linda Getsinger, Victor Waddell
EPI_ISL_2136449	Alaska State Virology Laboratory	Alaska State Virology Laboratory	Stephanie DeRonde, Elva House, Jacob Zidek, Lisa Smith, Ph.D., Jack Chen, Ph.D.
EPI_ISL_2138873, EPI_ISL_2138907, EPI_ISL_2139642	Oregon State Public Health Laboratory	Oregon State Public Health Laboratory	Rafia Razzaque, Eugene Yeboah, Vanda Makris, Laura Tsaknaridis, John Fontana and Shane Sevey
EPI_ISL_2140161	Idaho Bureau of Laboratories	Idaho Bureau of Laboratories	R. Beukelman, Matthew Charles Burns, Aimee Ceniseros, Robert L. Voermans, Christopher Ball
EPI_ISL_2146105, EPI_ISL_2146116, EPI_ISL_2146207, EPI_ISL_2146226, EPI_ISL_2146291, EPI_ISL_2146338, EPI_ISL_2146410, EPI_ISL_2146501, EPI_ISL_2146558, EPI_ISL_2146572, EPI_ISL_2146578, EPI_ISL_2146658, EPI_ISL_2146799, EPI_ISL_2146857, EPI_ISL_2146956, EPI_ISL_2147097, EPI_ISL_2147215, EPI_ISL_2147413, EPI_ISL_2147414, EPI_ISL_2147415, EPI_ISL_2147416, EPI_ISL_2147518, EPI_ISL_2147628, EPI_ISL_2147934, EPI_ISL_2147958, EPI_ISL_2147986, EPI_ISL_2148114, EPI_ISL_2148241, EPI_ISL_2148927, EPI_ISL_2148941, EPI_ISL_2148964, EPI_ISL_2148965, EPI_ISL_2148969, EPI_ISL_2149161, EPI_ISL_2149176, EPI_ISL_2149179			
see above	Aegis Sciences Corporation	Centers for Disease Control and Prevention Division of Viral Diseases, Pathogen Discovery	Dakota Howard, Dhwani Batra, Peter W. Cook, Kara Moser, Adrian Paskey, Jason Caravas, Benjamin Rambo-Martin, Shatavia Morrison, Christopher Gulvick, Scott Sammons, Yvette Unoarumhi, Darlene Wagner, Matthew Schmerer, Cyndi Clark, Patrick Campbell, Rob Case, Vikramsinha Ghorpade, Holly Houdeshell, Ola Kvalvaag, Dillon Nall, Ethan Sanders, Alec Vest, Shaun Westlund, Matthew Hardison, Clinton R. Paden, Duncan MacCannell
EPI_ISL_2152865	Lighthouse Lab in Milton Keynes	Wellcome Sanger Institute for the COVID-19 Genomics UK (COG-UK) Consortium	The Lighthouse Lab in Milton Keynes and Alex Alderton, Roberto Amato, Jeffrey Barrett, Sonia Goncalves, Ewan Harrison, David K. Jackson, Ian Johnston, Dominic Kwiatkowski, Cordelia Langford, John Sillitoe on behalf of the Wellcome Sanger Institute COVID-19 Surveillance Team
EPI_ISL_2153101	Wyoming Public Health Laboratory	Wyoming Public Health Laboratory	Jim Mildenberger, Taylor Fearing, Channing Weber, Ashley Norberg, Chayse Rowley, Marley Goetz, Brian Dominguez, Elliot Thomasson, Brittany Oher, Sam Britz, Cari Sloma, and Rob Christensen
EPI_ISL_2158836	Servicio Virosis Respiratorias-Departamento Virología-INEI	Instituto Nacional Enfermedades Infecciosas C.G.Malbran	Baumeister E., Avaro M., Benedetti E., Russo M., Dattero ME, Pontoriero A., Cisterna D., Molina V., Perandones C., Tuduri E., Lorenzo F., Poklepovich T., Campos J.
EPI_ISL_2158869, EPI_ISL_2158901, EPI_ISL_2158953, EPI_ISL_2158954, EPI_ISL_2158956, EPI_ISL_2158960, EPI_ISL_2159062, EPI_ISL_2159131, EPI_ISL_2159155, EPI_ISL_2159193, EPI_ISL_2159360, EPI_ISL_2159371			
see above	Aegis Sciences Corporation	Centers for Disease Control and Prevention Division of Viral Diseases, Pathogen Discovery	Dakota Howard, Dhwani Batra, Peter W. Cook, Kara Moser, Adrian Paskey, Jason Caravas, Benjamin Rambo-Martin, Shatavia Morrison, Christopher Gulvick, Scott Sammons, Yvette Unoarumhi, Darlene Wagner, Matthew Schmerer, Cyndi Clark, Patrick Campbell, Rob Case, Vikramsinha Ghorpade, Holly Houdeshell, Ola Kvalvaag, Dillon Nall, Ethan Sanders, Alec Vest, Shaun Westlund, Matthew Hardison, Clinton R. Paden, Duncan MacCannell
EPI_ISL_2160981, EPI_ISL_2161009	Fulgent Genetics	Centers for Disease Control and Prevention Division of Viral Diseases, Pathogen Discovery	Dakota Howard, Dhwani Batra, Peter W. Cook, Kara Moser, Adrian Paskey, Jason Caravas, Benjamin Rambo-Martin, Shatavia Morrison, Christopher Gulvick, Scott Sammons, Yvette Unoarumhi, Darlene Wagner, Matthew Schmerer, Harry Gao, Mickey Li, John Gao, Joseph Fierro, Benafsh Sapra, Becky Tsai, Yan Meng, Doreen Ng, James Xie, Clinton R. Paden, Duncan MacCannell
EPI_ISL_2161821, EPI_ISL_2161824, EPI_ISL_2161825, EPI_ISL_2161828, EPI_ISL_2161830, EPI_ISL_2161864, EPI_ISL_2161868, EPI_ISL_2161870, EPI_ISL_2161877, EPI_ISL_2161884, EPI_ISL_2161889, EPI_ISL_2161890, EPI_ISL_2161898, EPI_ISL_2161899, EPI_ISL_2161933, EPI_ISL_2161946, EPI_ISL_2161948, EPI_ISL_2161987			
see above	UW Virology Lab	UW Virology Lab	Pavitra Roychoudhury, Hong Xie, Lasata Shrestha, Tien V. Nguyen, Shah Mohamed Bakhash, Michelle Lin, Noah R. Baker, Ricardo Perez, Sean Ellis, Nathan Breit, Robert J. Livingston, Meei-Li Huang, Keith R Jerome, Patrick Mathias, Alexander Greninger
EPI_ISL_2180605, EPI_ISL_2180615, EPI_ISL_2180625, EPI_ISL_2180687, EPI_ISL_2180777, EPI_ISL_2180903, EPI_ISL_2180904, EPI_ISL_2180936, EPI_ISL_2181140, EPI_ISL_2181355, EPI_ISL_2181369, EPI_ISL_2181427, EPI_ISL_2181454, EPI_ISL_2181468, EPI_ISL_2181484, EPI_ISL_2181546, EPI_ISL_2181585,			

EPI_ISL_2181587			
see above	Aegis Sciences Corporation	Centers for Disease Control and Prevention Division of Viral Diseases, Pathogen Discovery	Dakota Howard, Dhwani Batra, Peter W. Cook, Kara Moser, Adrian Paskey, Jason Caravas, Benjamin Rambo-Martin, Shatavia Morrison, Christopher Gulvick, Scott Sammons, Yvette Unoarumhi, Darlene Wagner, Matthew Schmerer, Cyndi Clark, Patrick Campbell, Rob Case, Vikramsinha Ghorpade, Holly Houdeshell, Ola Kvalvaag, Dillon Nall, Ethan Sanders, Alec Vest, Shaun Westlund, Matthew Hardison, Clinton R. Paden, Duncan MacCannell
EPI_ISL_2182205, EPI_ISL_2182468, EPI_ISL_2182815, EPI_ISL_2182825, EPI_ISL_2182861, EPI_ISL_2182899, EPI_ISL_2183979, EPI_ISL_2184230, EPI_ISL_2184253, EPI_ISL_2184943, EPI_ISL_2185081, EPI_ISL_2185202, EPI_ISL_2185268, EPI_ISL_2185465, EPI_ISL_2185516			
see above	Laboratory Corporation of America	Centers for Disease Control and Prevention Division of Viral Diseases, Pathogen Discovery	Dakota Howard, Dhwani Batra, Peter W. Cook, Kara Moser, Adrian Paskey, Jason Caravas, Benjamin Rambo-Martin, Shatavia Morrison, Christopher Gulvick, Scott Sammons, Yvette Unoarumhi, Darlene Wagner, Matthew Schmerer, Minoo Agarwal, Eyad Almasri, Debbie Boles, Ayla Burns, Nuthawin Charoensri, Oren Cohen, Susan Countryman, Mary Ann Cristobal, Bobbi Croy, Suzanne Dale, Hrushikesh Deshmukh, Amanda Douglas, Vincent Drouillon, Marcia Eisenberg, Howard Engler, Rama Ghatti, Prashant Gupta, Susan Hicks, Jake Humphrey, Lax Iyer, Manoj Jain, Mohan Kolli, Brian Krueger, Tim Kuphal, Stanley Letovsky, Michael Levandoski, Craig Lukasik, Jonathan Meltzer, Brian Norvell, Mindy Nye, Scott Parker, Christos Petropoulos, John Pruitt, Steven Ragan, Scott Ryan, Mike Sapeta, Jana Schroth, Suresh Babu Selvaraju, Goran Stevovic, Amanda Suchanek, Andrea Throop, Lyndon Tilson, Thomas Urban, Joe Voshell, Kimberly Wagner, Jonathan Williams, Mary Williamson, Qian Zeng, Tricia Zwiefelhofer, Clinton R. Paden, Duncan MacCannell
EPI_ISL_2186559, EPI_ISL_2186706	Infinity Biologix	Centers for Disease Control and Prevention Division of Viral Diseases, Pathogen Discovery	Dakota Howard, Dhwani Batra, Peter W. Cook, Kara Moser, Adrian Paskey, Jason Caravas, Benjamin Rambo-Martin, Shatavia Morrison, Christopher Gulvick, Scott Sammons, Yvette Unoarumhi, Darlene Wagner, Matthew Schmerer, Christian Bixby, Yihe Wang, Jonathan Schultz, Chirayu Goswami, Russ Hager, Robin Grimwood, Clinton R. Paden, Duncan MacCannell
EPI_ISL_2187526, EPI_ISL_2187595	Aegis Sciences Corporation	Centers for Disease Control and Prevention Division of Viral Diseases, Pathogen Discovery	Dakota Howard, Dhwani Batra, Peter W. Cook, Kara Moser, Adrian Paskey, Jason Caravas, Benjamin Rambo-Martin, Shatavia Morrison, Christopher Gulvick, Scott Sammons, Yvette Unoarumhi, Darlene Wagner, Matthew Schmerer, Cyndi Clark, Patrick Campbell, Rob Case, Vikramsinha Ghorpade, Holly Houdeshell, Ola Kvalvaag, Dillon Nall, Ethan Sanders, Alec Vest, Shaun Westlund, Matthew Hardison, Clinton R. Paden, Duncan MacCannell
EPI_ISL_2187634	Helix/Illumina	Centers for Disease Control and Prevention Division of Viral Diseases, Pathogen Discovery	Dakota Howard, Dhwani Batra, Peter W. Cook, Kara Moser, Adrian Paskey, Jason Caravas, Benjamin Rambo-Martin, Shatavia Morrison, Christopher Gulvick, Scott Sammons, Yvette Unoarumhi, Darlene Wagner, Matthew Schmerer, Eileen de Feo, Jan Antico, Christine Tran, Matthew Tolentino, Shannon Wickline, Kim Gietzen, Brad Sickler, Jingtao Liu, Eric Allen, Phil Febbo, Nicole L. Washington, Simon White, Geraint Levan, Kelly Schiabor Barrett, Elizabeth Cirulli, Alexandre Bolze, Ary Ascencio, Charlotte Rivera-Garcia, Ryan Cho, Jason Nguyen, Sherry Wang, Jimmy Ramirez, Tyler Cassens, Efren Sandoval, Magnus Isaksson, William Lee, David Becker, Marc Laurent, James Lu, Clinton R. Paden, Duncan MacCannell
EPI_ISL_2192845	Broad Institute Clinical Research Sequencing Platform	Infectious Disease Program, Broad Institute of Harvard and MIT	Siddle,K.J., Adams,G., Pearlman,L., Gladden-Young,A., Vicente,G., Blumenstiel,B., DeFelice,M., Lee,M., McGovern,S., Lagerborg,K., Rudy,M., DeRuff,K., Carter,A., Normandin,E., Bauer,M., Reilly,S., Tomkins-Tinch,C., Loreth,C., Chaluvadi,S., Meldrim,J., Granger,B., Lemieux,J.E., Birren,B.W., Sabeti,P.C., Larkin,K., Dodge,S., Lennon,N., Madoff,L., Brown,C., Gallagher,G., Smole,S., Park,D.J., Gabriel,S., and MacInnis,B.L.
EPI_ISL_2194040, EPI_ISL_2194043, EPI_ISL_2194064, EPI_ISL_2194066, EPI_ISL_2194073, EPI_ISL_2194077, EPI_ISL_2194096, EPI_ISL_2194107, EPI_ISL_2194124, EPI_ISL_2194145, EPI_ISL_2194155, EPI_ISL_2194164, EPI_ISL_2194166, EPI_ISL_2194168, EPI_ISL_2194169, EPI_ISL_2194181			
see above	UW Virology Lab	UW Virology Lab	Pavitra Roychoudhury, Hong Xie, Lasata Shrestha, Tien V. Nguyen, Shah Mohamed Bakhash, Michelle Lin, Noah R. Baker, Ricardo Perez, Sean Ellis, Nathan Breit, Robert J. Livingston, Meei-Li Huang, Keith R Jerome, Patrick Mathias, Alexander Greninger
EPI_ISL_2203056, EPI_ISL_2203080, EPI_ISL_2203085	Oregon State Public Health Laboratory	Oregon State Public Health Laboratory	Rafia Razzaque, Eugene Yeboah, Vanda Makris, Laura Tsaknaridis, John Fontana and Shane Sevey
EPI_ISL_2203812, EPI_ISL_2203833	Quest Diagnostics Incorporated	Centers for Disease Control and Prevention Division of Viral Diseases, Pathogen Discovery	Dakota Howard, Dhwani Batra, Peter W. Cook, Kara Moser, Adrian Paskey, Jason Caravas, Benjamin Rambo-Martin, Shatavia Morrison, Christopher Gulvick, Scott Sammons, Yvette Unoarumhi, Darlene Wagner, Matthew Schmerer, S. H. Rosenthal, A. Gerasimova, R. M. Kagan, B. Anderson, M. Hua, Y. Liu, L.E. Bernstein, K.E. Livingston, A. Perez, I. A. Shlyakhter, R. V. Rolando, R. Owen, P. Tanpaiboon, F. Lacbawan, Clinton R. Paden, Duncan MacCannell
EPI_ISL_2204946	WVU Rapid Development Lab	WVU and Marshall University Combined Genomics Core Facilities	James Denvir, Peter Stoilov, Peter Perrotta, Wesley Kimble, Ryan Percifield
EPI_ISL_2211077	Nevada State Public Health Laboratory	Nevada State Public Health Laboratory	Andrew Gorzalski, Mark Pandori
EPI_ISL_2225571	University of Wisconsin-Madison AIDS Vaccine Research Laboratories	University of Wisconsin-Madison AIDS Vaccine Research Laboratories	Gage Moreno, Katarina Braun, et al. AIDS Vaccine Research Laboratories
EPI_ISL_2226828	Wyoming Public Health Laboratory	Wyoming Public Health Laboratory	Jim Mildenberger, Wanda Manley, Noah Hull, Taylor Fearing, Lynette Gumbleton, Channing Weber, Ashley Norberg, Chayse Rowley, Marley Goetz, Brian Dominguez, Elliot Thomasson, Sam Britz, Cari Sloma, and Rob Christensen
EPI_ISL_2229285	"NM Dept. Health, Scientific Laboratory Division "	Centers for Disease Control and Prevention Division of Viral Diseases, Pathogen Discovery	Mili Sheth, Sarah Nobles, Jasmine Padilla, Mark Burroughs, Shoshona Le, Katie Dillon, Peter Cook, Clinton R. Paden, Dhwani Batra, Krista Queen, Kristen Knipe, Dakota Howard, Yvette Unoarumhi, Darlene Wagner, Matthew Schmerer, Ben L. Rambo-Martin, Kristine Lacek, Sam Shepard, Alison Laufer Halpin, Dave Wentworth, Vivien Dugan, Suxiang Tong, Justin Lee
EPI_ISL_2229291, EPI_ISL_2229292	MT Public Health Laboratory	Centers for Disease Control and Prevention Division of Viral Diseases, Pathogen Discovery	Mili Sheth, Sarah Nobles, Jasmine Padilla, Mark Burroughs, Shoshona Le, Katie Dillon, Peter Cook, Clinton R. Paden, Dhwani Batra, Krista Queen, Kristen Knipe, Dakota Howard, Yvette Unoarumhi, Darlene Wagner, Matthew Schmerer, Ben L. Rambo-Martin, Kristine Lacek, Sam Shepard, Alison Laufer Halpin, Dave Wentworth, Vivien Dugan, Suxiang Tong, Justin Lee
EPI_ISL_2232562	Minnesota Department of Health, Public Health Laboratory	Minnesota Department of Health, Public Health Laboratory	Alexandra Lorentz, Jacob Garfin, Matt Plumb, and Xiong Wang
EPI_ISL_2233255, EPI_ISL_2233260, EPI_ISL_2233294, EPI_ISL_2233299, EPI_ISL_2233304, EPI_ISL_2233306, EPI_ISL_2233312, EPI_ISL_2233314, EPI_ISL_2233327	UW Virology Lab	UW Virology Lab	Pavitra Roychoudhury, Hong Xie, Lasata Shrestha, Tien V. Nguyen, Shah Mohamed Bakhash, Michelle Lin, Noah R. Baker, Sean Ellis, Meei-Li Huang, Keith R Jerome, Alexander Greninger
EPI_ISL_2234923, EPI_ISL_2235044	Pandemic Response Lab - NYC	Pandemic Response Lab, R&D	Henry Lee, Michael Hammerling, Melissa Hopkins, Cybill del Castillo, Shinyoung Clair Kang, William Ward, Pradeep Bugga, Sol Rey, Dylan Law, Katharine Nelson, Haiping Hao, Jon Laurent
EPI_ISL_2242509	IN State Department of Health Laboratory Services	IN State Department of Health Laboratory Services	Cassandra Campion, Jamie Yeadon, Brian Pope, Lixia Liu, Kyle Brownlee, Melissa Hindenlang, Mark Glazier
EPI_ISL_2244949	M Health Fairview	Minnesota Department of Health, Public Health Laboratory	Alexandra Lorentz, Jacob Garfin, Matt Plumb, and Xiong Wang
EPI_ISL_2245492, EPI_ISL_2245591, EPI_ISL_2245592	Maine Health and Environmental Testing Laboratory	Tewhey Lab, The Jackson Laboratory	Matluk,N., Dewey,H., Iosue,F., Barter,M., Lynch,R., Munger,H. and Tewhey,R.
EPI_ISL_2247067, EPI_ISL_2247157, EPI_ISL_2247179, EPI_ISL_2247191	Quest Diagnostics Incorporated	Centers for Disease Control and Prevention Division of Viral Diseases, Pathogen Discovery	Dakota Howard, Dhwani Batra, Peter W. Cook, Kara Moser, Adrian Paskey, Jason Caravas, Benjamin Rambo-Martin, Shatavia Morrison, Christopher Gulvick, Scott Sammons, Yvette Unoarumhi, Darlene Wagner, Matthew Schmerer, S. H. Rosenthal, A. Gerasimova, R. M. Kagan, B. Anderson, M. Hua, Y. Liu, L.E. Bernstein, K.E. Livingston, A. Perez, I. A. Shlyakhter, R. V. Rolando, R. Owen, P. Tanpaiboon, F. Lacbawan, Clinton R. Paden, Duncan MacCannell
EPI_ISL_2247419, EPI_ISL_2247488, EPI_ISL_2247514, EPI_ISL_2247607, EPI_ISL_2248020, EPI_ISL_2248451	Helix/Illumina	Centers for Disease Control and Prevention Division of Viral Diseases, Pathogen Discovery	Dakota Howard, Dhwani Batra, Peter W. Cook, Kara Moser, Adrian Paskey, Jason Caravas, Benjamin Rambo-Martin, Shatavia Morrison, Christopher Gulvick, Scott Sammons, Yvette Unoarumhi, Darlene Wagner, Matthew Schmerer, Eileen de Feo, Jan Antico, Christine Tran, Matthew Tolentino, Shannon Wickline, Kim Gietzen, Brad Sickler, Jingtao Liu, Eric Allen, Phil Febbo, Nicole L. Washington, Simon White, Geraint Levan, Kelly Schiabor Barrett, Elizabeth Cirulli, Alexandre Bolze, Ary Ascencio, Charlotte Rivera-Garcia, Ryan Cho, Jason Nguyen, Sherry Wang, Jimmy Ramirez, Tyler Cassens, Efren Sandoval, Magnus Isaksson, William Lee, David Becker, Marc Laurent, James Lu, Clinton R. Paden, Duncan MacCannell
EPI_ISL_2248553, EPI_ISL_2248555, EPI_ISL_2248572, EPI_ISL_2248607, EPI_ISL_2248637, EPI_ISL_2248672	Quest Diagnostics Incorporated	Centers for Disease Control and Prevention Division of Viral Diseases, Pathogen Discovery	Dakota Howard, Dhwani Batra, Peter W. Cook, Kara Moser, Adrian Paskey, Jason Caravas, Benjamin Rambo-Martin, Shatavia Morrison, Christopher Gulvick, Scott Sammons, Yvette Unoarumhi, Darlene Wagner, Matthew Schmerer, S. H. Rosenthal, A. Gerasimova, R. M. Kagan, B. Anderson, M. Hua, Y. Liu, L.E. Bernstein, K.E. Livingston, A. Perez, I. A. Shlyakhter, R. V. Rolando, R. Owen, P. Tanpaiboon, F. Lacbawan, Clinton R. Paden, Duncan MacCannell

EPI_ISL_2249244, EPI_ISL_2249245	Hospital Margarita Maza de Juárez	Microbial Genomics Laboratory	Bruno Gomez-Gil, Julissa Enciso-Ibarra, Alejandra Garcia-Gasca, Daniel Fregoso-Rueda
EPI_ISL_2249569	M Health Fairview	Minnesota Department of Health, Public Health Laboratory	Alexandra Lorentz, Jacob Garfin, Matt Plumb, and Xiong Wang
EPI_ISL_2250089, EPI_ISL_2250091	Arizona State University	Arizona State University	Peter T. Skidmore, LaRinda A. Holland, Matthew F. Smith, Rabia Maqsood, Nicholas J. Mellor, Joy M. Blain, Valerie Harris, Joshua LaBaer, Vel Murugan, Efrem S. Lim
EPI_ISL_2250158, EPI_ISL_2250176	Oregon State Public Health Laboratory	Oregon State Public Health Laboratory	Rafia Razzaque, Eugene Yeboah, Vanda Makris, Laura Tsaknaridis, John Fontana and Shane Sevey
EPI_ISL_2263490	Arizona State Public Health Laboratory	Arizona State Public Health Laboratory	Trung Huynh, Jessica Escobar, Katherine Fullerton, Nobuko Fukushima, Stacy White, Linda Getsinger, Victor Waddell
EPI_ISL_2268345	Helix/Illumina	Centers for Disease Control and Prevention Division of Viral Diseases, Pathogen Discovery	Dakota Howard, Dhwani Batra, Peter W. Cook, Kara Moser, Adrian Paskey, Jason Caravas, Benjamin Rambo-Martin, Shatavia Morrison, Christopher Gulvick, Scott Sammons, Yvette Unoarumhi, Darlene Wagner, Matthew Schmerer, Eileen de Feo, Jan Antico, Christine Tran, Matthew Tolentino, Shannon Wickline, Kim Gietzen, Brad Sickler, Jingtao Liu, Eric Allen, Phil Febbo, Nicole L. Washington, Simon White, Geraint Levan, Kelly Schiabor Barrett, Elizabeth Cirulli, Alexandre Bolze, Ary Ascencio, Charlotte Rivera-Garcia, Ryan Cho, Jason Nguyen, Sherry Wang, Jimmy Ramirez, Tyler Cassens, Efren Sandoval, Magnus Isaksson, William Lee, David Becker, Marc Laurent, James Lu, Clinton R. Paden, Duncan MacCannell
EPI_ISL_2268397, EPI_ISL_2268485, EPI_ISL_2268690, EPI_ISL_2268751, EPI_ISL_2268958, EPI_ISL_2269031, EPI_ISL_2269179, EPI_ISL_2269201	Quest Diagnostics Incorporated	Centers for Disease Control and Prevention Division of Viral Diseases, Pathogen Discovery	Dakota Howard, Dhwani Batra, Peter W. Cook, Kara Moser, Adrian Paskey, Jason Caravas, Benjamin Rambo-Martin, Shatavia Morrison, Christopher Gulvick, Scott Sammons, Yvette Unoarumhi, Darlene Wagner, Matthew Schmerer, S. H. Rosenthal, A. Gerasimova, R. M. Kagan, B. Anderson, M. Hua, Y. Liu, L.E. Bernstein, K.E. Livingston, A. Perez, I. A. Shlyakhter, R. V. Rolando, R. Owen, P. Tanpaiboon, F. Lacbawan, Clinton R. Paden, Duncan MacCannell
EPI_ISL_2269269, EPI_ISL_2269316, EPI_ISL_2269518, EPI_ISL_2269561, EPI_ISL_2269610	Fulgent Genetics	Centers for Disease Control and Prevention Division of Viral Diseases, Pathogen Discovery	Dakota Howard, Dhwani Batra, Peter W. Cook, Kara Moser, Adrian Paskey, Jason Caravas, Benjamin Rambo-Martin, Shatavia Morrison, Christopher Gulvick, Scott Sammons, Yvette Unoarumhi, Darlene Wagner, Matthew Schmerer, Harry Gao, Mickey Li, John Gao, Joseph Fierro, Benafsh Sapra, Becky Tsai, Yan Meng, Doreen Ng, James Xie, Clinton R. Paden, Duncan MacCannell
EPI_ISL_2269886, EPI_ISL_2269916, EPI_ISL_2269987, EPI_ISL_2270044, EPI_ISL_2270164, EPI_ISL_2270183, EPI_ISL_2270233, EPI_ISL_2270275, EPI_ISL_2270298, EPI_ISL_2270319, EPI_ISL_2270337, EPI_ISL_2270390, EPI_ISL_2270432			
see above	Helix/Illumina	Centers for Disease Control and Prevention Division of Viral Diseases, Pathogen Discovery	Dakota Howard, Dhwani Batra, Peter W. Cook, Kara Moser, Adrian Paskey, Jason Caravas, Benjamin Rambo-Martin, Shatavia Morrison, Christopher Gulvick, Scott Sammons, Yvette Unoarumhi, Darlene Wagner, Matthew Schmerer, Eileen de Feo, Jan Antico, Christine Tran, Matthew Tolentino, Shannon Wickline, Kim Gietzen, Brad Sickler, Jingtao Liu, Eric Allen, Phil Febbo, Nicole L. Washington, Simon White, Geraint Levan, Kelly Schiabor Barrett, Elizabeth Cirulli, Alexandre Bolze, Ary Ascencio, Charlotte Rivera-Garcia, Ryan Cho, Jason Nguyen, Sherry Wang, Jimmy Ramirez, Tyler Cassens, Efren Sandoval, Magnus Isaksson, William Lee, David Becker, Marc Laurent, James Lu, Clinton R. Paden, Duncan MacCannell
EPI_ISL_2271693, EPI_ISL_2271700, EPI_ISL_2271702	Laboratorio Central, Ministerio de Salud Cordoba	Instituto de Patologia Vegetal (CIAP-INTA) on behalf of 'Proyecto Argentino Interinstitucional de genomica de SARS-CoV-2' (PAIS Consortium)	Fernandez, FD; Marquez, N.; Debat, HJ.; M; Re, V.; Pisano, M.B.; Castro, G.; Barbas, G.
EPI_ISL_2272499, EPI_ISL_2272500	LANIIA	LANIIA	Cristobal Chaidez, Bruno Gomez-Gil, Jean Pierre González-Gómez, Juan Daniel Lira-Morales, Irvin González-López, Julissa Enciso-Ibarra, Célida Isabel Martínez-Rodríguez
EPI_ISL_2272521	Servicio Diagnóstico Clínico	LANIIA	Cristobal Chaidez, Bruno Gomez-Gil, Jean Pierre González-Gómez, Juan Daniel Lira-Morales, Irvin González-López, Julissa Enciso-Ibarra, Célida Isabel Martínez-Rodríguez
EPI_ISL_2272545, EPI_ISL_2272546	Alaska State Virology Laboratory	Alaska State Virology Laboratory	Stephanie DeRonde, Elva House, Jacob Zidek, Lisa Smith, Ph.D., Jack Chen, Ph.D.
EPI_ISL_2272610, EPI_ISL_2272619, EPI_ISL_2272645, EPI_ISL_2272646, EPI_ISL_2273780, EPI_ISL_2273791, EPI_ISL_2273793, EPI_ISL_2273824, EPI_ISL_2273828	UW Virology Lab	UW Virology Lab	Pavitra Roychoudhury, Hong Xie, Lasata Shrestha, Tien V. Nguyen, Shah Mohamed Bakhash, Michelle Lin, Noah R. Baker, Ricardo Perez, Sean Ellis, Nathan Breit, Robert J. Livingston, Meei-Li Huang, Keith R Jerome, Patrick Mathias, Alexander Greninger
EPI_ISL_2280335, EPI_ISL_2280384, EPI_ISL_2280429, EPI_ISL_2280596, EPI_ISL_2280792, EPI_ISL_2280824, EPI_ISL_2280915, EPI_ISL_2281096, EPI_ISL_2281118, EPI_ISL_2281120, EPI_ISL_2281129, EPI_ISL_2281131, EPI_ISL_2281172			
see above	Aegis Sciences Corporation	Centers for Disease Control and Prevention Division of Viral Diseases, Pathogen Discovery	Dakota Howard, Dhwani Batra, Peter W. Cook, Kara Moser, Adrian Paskey, Jason Caravas, Benjamin Rambo-Martin, Shatavia Morrison, Christopher Gulvick, Scott Sammons, Yvette Unoarumhi, Darlene Wagner, Matthew Schmerer, Cyndi Clark, Patrick Campbell, Rob Case, Vikramsinha Ghorpade, Holly Houdeshell, Ola Kvalvaag, Dillon Nall, Ethan Sanders, Alec Vest, Shaun Westlund, Matthew Hardison, Clinton R. Paden, Duncan MacCannell
EPI_ISL_2282336, EPI_ISL_2282431, EPI_ISL_2282476, EPI_ISL_2282487, EPI_ISL_2282539, EPI_ISL_2282546, EPI_ISL_2282598, EPI_ISL_2282642, EPI_ISL_2282681, EPI_ISL_2282821, EPI_ISL_2282858, EPI_ISL_2282864			
see above	Infinity Biologix	Centers for Disease Control and Prevention Division of Viral Diseases, Pathogen Discovery	Dakota Howard, Dhwani Batra, Peter W. Cook, Kara Moser, Adrian Paskey, Jason Caravas, Benjamin Rambo-Martin, Shatavia Morrison, Christopher Gulvick, Scott Sammons, Yvette Unoarumhi, Darlene Wagner, Matthew Schmerer, Christian Bixby, Yihe Wang, Jonathan Schultz, Chirayu Goswami, Russ Hager, Robin Grimwood, Clinton R. Paden, Duncan MacCannell
EPI_ISL_2285099	Mako Medical	Centers for Disease Control and Prevention Division of Viral Diseases, Pathogen Discovery	Dakota Howard, Dhwani Batra, Peter W. Cook, Kara Moser, Adrian Paskey, Jason Caravas, Benjamin Rambo-Martin, Shatavia Morrison, Christopher Gulvick, Scott Sammons, Yvette Unoarumhi, Darlene Wagner, Matthew Schmerer,Matthew Tugwell, Lauren Moon, Clinton R. Paden, Duncan MacCannell
EPI_ISL_2287853	New Mexico Department of Health Scientific Laboratory	New Mexico Department of Health Scientific Laboratory	Ellie Johnson, Anastacia Griego-Fisher, D'eldra Malone, Jennifer Benoit
EPI_ISL_2291653, EPI_ISL_2292777, EPI_ISL_2292834, EPI_ISL_2292846, EPI_ISL_2292878, EPI_ISL_2292917	Utah Public Health Laboratory	Utah Public Health Laboratory	Erin L. Young, Kelly F. Oakeson, Tara Gallagher
EPI_ISL_2293023, EPI_ISL_2293024	LANIIA	LANIIA	Cristobal Chaidez, Bruno Gomez-Gil, Jean Pierre González-Gómez, Juan Daniel Lira-Morales, Irvin González-López, Julissa Enciso-Ibarra, Célida Isabel Martínez-Rodríguez
EPI_ISL_2296103	LESP Sinaloa	Instituto de Diagnostico y Referencia Epidemiologicos (INDRE)	Claudia Wong-Arambula, Abril Rodriguez-Maldonado, Vanessa Rivero-Arredondo, Ariadna Medina-Benitez, Joaquin Quiroz-Mercado, Sergio Rangel-Guerrero, Natividad Cruz-Ortiz, Tatiana Nunez-Garcia, Gisela Barrera-Badillo, Lucia Hernandez-Rivas, Irma Lopez-Martinez, Ernesto Ramirez-Gonzalez.
EPI_ISL_2296104	LESP Baja California	Instituto de Diagnostico y Referencia Epidemiologicos (INDRE)	Claudia Wong-Arambula, Abril Rodriguez-Maldonado, Vanessa Rivero-Arredondo, Ariadna Medina-Benitez, Joaquin Quiroz-Mercado, Sergio Rangel-Guerrero, Natividad Cruz-Ortiz, Tatiana Nunez-Garcia, Gisela Barrera-Badillo, Lucia Hernandez-Rivas, Irma Lopez-Martinez, Ernesto Ramirez-Gonzalez.
EPI_ISL_2296512	Centracare Laboratory Services	Minnesota Department of Health, Public Health Laboratory	Alexandra Lorentz, Jacob Garfin, Matt Plumb, and Xiong Wang
EPI_ISL_2299306, EPI_ISL_2299368, EPI_ISL_2299402, EPI_ISL_2299495, EPI_ISL_2300671, EPI_ISL_2300679, EPI_ISL_2300692, EPI_ISL_2300693, EPI_ISL_2300694, EPI_ISL_2300701	UW Virology Lab	UW Virology Lab	Pavitra Roychoudhury, Hong Xie, Lasata Shrestha, Tien V. Nguyen, Shah Mohamed Bakhash, Michelle Lin, Noah R. Baker, Sean Ellis, Meei-Li Huang, Keith R Jerome, Alexander Greninger
EPI_ISL_2304505, EPI_ISL_2304518, EPI_ISL_2304565, EPI_ISL_2304618, EPI_ISL_2304623	Quest Diagnostics Incorporated	Centers for Disease Control and Prevention Division of Viral Diseases, Pathogen Discovery	Dakota Howard, Dhwani Batra, Peter W. Cook, Kara Moser, Adrian Paskey, Jason Caravas, Benjamin Rambo-Martin, Shatavia Morrison, Christopher Gulvick, Scott Sammons, Yvette Unoarumhi, Darlene Wagner, Matthew Schmerer, S. H. Rosenthal, A. Gerasimova, R. M. Kagan, B. Anderson, M. Hua, Y. Liu, L.E. Bernstein, K.E. Livingston, A. Perez, I. A. Shlyakhter, R. V. Rolando, R. Owen, P. Tanpaiboon, F. Lacbawan, Clinton R. Paden, Duncan MacCannell
EPI_ISL_2304961, EPI_ISL_2305141, EPI_ISL_2305384, EPI_ISL_2306197, EPI_ISL_2306351, EPI_ISL_2306352, EPI_ISL_2306353, EPI_ISL_2306537, EPI_ISL_2306549, EPI_ISL_2306665, EPI_ISL_2306672			
see above	Laboratory Corporation of America	Centers for Disease Control and Prevention Division of Viral Diseases, Pathogen Discovery	Dakota Howard, Dhwani Batra, Peter W. Cook, Kara Moser, Adrian Paskey, Jason Caravas, Benjamin Rambo-Martin, Shatavia Morrison, Christopher Gulvick, Scott Sammons, Yvette Unoarumhi, Darlene Wagner, Matthew Schmerer, Minoo Agarwal, Eyad Almasri, Debbie Boles, Ayla Burns, Nuthawin Charoensri, Oren Cohen, Susan Countryman, Mary Ann Cristobal, Bobbi Croy, Suzanne Dale, Hrushikesh Deshmukh, Amanda Douglas, Vincent Drouillon,

			Marcia Eisenberg, Howard Engler, Rama Ghatti, Prashant Gupta, Susan Hicks, Jake Humphrey, Lax Iyer, Manoj Jain, Mohan Kolli, Brian Krueger, Tim Kuphal, Stanley Letovsky, Michael Levandoski, Craig Lukasik, Jonathan Meltzer, Brian Norvell, Mindy Nye, Scott Parker, Christos Petropoulos, John Pruitt, Steven Ragan, Scott Ryan, Mike Sapeta, Jana Schroth, Suresh Babu Selvaraju, Goran Stevovic, Amanda Suchanek, Andrea Throop, Lyndon Tilson, Thomas Urban, Joe Voshell, Kimberly Wagner, Jonathan Williams, Mary Williamson, Qian Zeng, Tricia Zwiefelhofer, Clinton R. Paden, Duncan MacCannell
EPI_ISL_2307199, EPI_ISL_2307243, EPI_ISL_2307286, EPI_ISL_2307304	Mako Medical	Centers for Disease Control and Prevention Division of Viral Diseases, Pathogen Discovery	Dakota Howard, Dhwani Batra, Peter W. Cook, Kara Moser, Adrian Paskey, Jason Caravas, Benjamin Rambo-Martin, Shatavia Morrison, Christopher Gulvick, Scott Sammons, Yvette Unoarumhi, Darlene Wagner, Matthew Schmerer,Matthew Tugwell, Lauren Moon, Clinton R. Paden, Duncan MacCannell
EPI_ISL_2308343	Montana Public Health Laboratory	Montana Public Health Laboratory	Joy Ritter, Michelle Mozer, Carrie Biskupiak, Deborah Gibson, Michael Dills
EPI_ISL_2308503	Kansas Health and Environmental Lab	Kansas Health and Environmental Lab	Mike Grose, Katherine Wiggins, Jonathan Barnell, Ben Olsen, and Phil Adam
EPI_ISL_2308682	Clinical Reference Laboratory	Kansas Health and Environmental Lab	Mike Grose, Katherine Wiggins, Jonathan Barnell, Ben Olsen, and Phil Adam
EPI_ISL_2309988, EPI_ISL_2309989, EPI_ISL_2310134, EPI_ISL_2310168	Colorado Department of Public Health and Environment	Colorado Department of Public Health and Environment	Laura Bankers, Molly C. Hetherington-Rauth, Diana Ir, Alexandria Rossheim, Shannon R. Matzinger, Sarah Elizabeth Totten, Emily A. Travanty
EPI_ISL_2319353	INDRE	Instituto Nacional de Medicina Genomica	Hidalgo-Miranda A, Mendoza-Vargas A, Reyes-Grajeda JP, Cedro-Tanda A, Gisela Barrera-Badillo, Irma Lopez-Martinez, Jose Ernesto Ramirez González, Gonzalez-Barrera D, Rangel-DeLeon D, Munguia-Garza P, Garcia-Cardenas FJ, Gonzalez-Woge MA, Herrera-Montalvo LA.
EPI_ISL_2320134, EPI_ISL_2320220, EPI_ISL_2320226, EPI_ISL_2320314, EPI_ISL_2320748, EPI_ISL_2320774, EPI_ISL_2320779, EPI_ISL_2320864, EPI_ISL_2320978, EPI_ISL_2321019, EPI_ISL_2321112			
see above	Helix/Illumina	Centers for Disease Control and Prevention Division of Viral Diseases, Pathogen Discovery	Dakota Howard, Dhwani Batra, Peter W. Cook, Kara Moser, Adrian Paskey, Jason Caravas, Benjamin Rambo-Martin, Shatavia Morrison, Christopher Gulvick, Scott Sammons, Yvette Unoarumhi, Darlene Wagner, Matthew Schmerer, Eileen de Feo, Jan Antico, Christine Tran, Matthew Tolentino, Shannon Wickline, Kim Gietzen, Brad Sickler, Jingtao Liu, Eric Allen, Phil Febbo, Nicole L. Washington, Simon White, Geraint Levan, Kelly Schiabor Barrett, Elizabeth Cirulli, Alexandre Bolze, Ary Ascencio, Charlotte Rivera-Garcia, Ryan Cho, Jason Nguyen, Sherry Wang, Jimmy Ramirez, Tyler Cassens, Efren Sandoval, Magnus Isaksson, William Lee, David Becker, Marc Laurent, James Lu, Clinton R. Paden, Duncan MacCannell
EPI_ISL_2321319, EPI_ISL_2321366, EPI_ISL_2321379	Aegis Sciences Corporation	Centers for Disease Control and Prevention Division of Viral Diseases, Pathogen Discovery	Dakota Howard, Dhwani Batra, Peter W. Cook, Kara Moser, Adrian Paskey, Jason Caravas, Benjamin Rambo-Martin, Shatavia Morrison, Christopher Gulvick, Scott Sammons, Yvette Unoarumhi, Darlene Wagner, Matthew Schmerer, Cyndi Clark, Patrick Campbell, Rob Case, Vikramsinha Ghorpade, Holly Houdeshell, Ola Kvalvaag, Dillon Nall, Ethan Sanders, Alec Vest, Shaun Westlund, Matthew Hardison, Clinton R. Paden, Duncan MacCannell
EPI_ISL_2322813, EPI_ISL_2322814, EPI_ISL_2322815, EPI_ISL_2322816, EPI_ISL_2322817, EPI_ISL_2322818	New Mexico Department of Health Scientific Laboratory	New Mexico Department of Health Scientific Laboratory	Ellie Johnson, Anastacia Griego-Fisher, D'eldra Malone, Jennifer Benoit
EPI_ISL_2323735, EPI_ISL_2323755, EPI_ISL_2323910, EPI_ISL_2323939, EPI_ISL_2324053, EPI_ISL_2324081, EPI_ISL_2324082	Infinity Biologix	Centers for Disease Control and Prevention Division of Viral Diseases, Pathogen Discovery	Dakota Howard, Dhwani Batra, Peter W. Cook, Kara Moser, Adrian Paskey, Jason Caravas, Benjamin Rambo-Martin, Shatavia Morrison, Christopher Gulvick, Scott Sammons, Yvette Unoarumhi, Darlene Wagner, Matthew Schmerer, Christian Bixby, Yihe Wang, Jonathan Schultz, Chirayu Goswami, Russ Hager, Robin Grimwood, Clinton R. Paden, Duncan MacCannell
EPI_ISL_2324480, EPI_ISL_2324527, EPI_ISL_2324550	Laboratory Corporation of America	Centers for Disease Control and Prevention Division of Viral Diseases, Pathogen Discovery	Dakota Howard, Dhwani Batra, Peter W. Cook, Kara Moser, Adrian Paskey, Jason Caravas, Benjamin Rambo-Martin, Shatavia Morrison, Christopher Gulvick, Scott Sammons, Yvette Unoarumhi, Darlene Wagner, Matthew Schmerer, Minoo Agarwal, Eyad Almasri, Debbie Boles, Ayla Burns, Nuthawin Charoensri, Oren Cohen, Susan Countryman, Mary Ann Cristobal, Bobbi Croy, Suzanne Dale, Hrushikesh Deshmukh, Amanda Douglas, Vincent Drouillon, Marcia Eisenberg, Howard Engler, Rama Ghatti, Prashant Gupta, Susan Hicks, Jake Humphrey, Lax Iyer, Lisa Pfefferle, Manoj Jain, Matthew Robinson, Mohan Kolli, Brian Krueger, Tim Kuphal, Stanley Letovsky, Michael Levandoski, Craig Lukasik, Jonathan Meltzer, Brian Norvell, Mindy Nye, Scott Parker, Christos Petropoulos, John Pruitt, Steven Ragan, Scott Ryan, Mike Sapeta, Jana Schroth, Suresh Babu Selvaraju, Goran Stevovic, Amanda Suchanek, Andrea Throop, Lyndon Tilson, Thomas Urban, Joe Voshell, Kimberly Wagner, Jonathan Williams, Mary Williamson, Qian Zeng, Tricia Zwiefelhofer, Clinton R. Paden, Duncan MacCannell
EPI_ISL_2325081	Minnesota Department of Health, Public Health Laboratory	Minnesota Department of Health, Public Health Laboratory	Alexandra Lorentz, Jacob Garfin, Matt Plumb, and Xiong Wang
EPI_ISL_2329444	Sharp HealthCare Laboratory	Andersen lab at Scripps Research	SEARCH Alliance San Diego with Aaron Harding, Jacquelyn Berumen, Cathy Woerle, Liam McGinnis, Art Mendoza, Omid Bakhtar
EPI_ISL_2332703	Centracare Laboratory Services	Minnesota Department of Health, Public Health Laboratory	Alexandra Lorentz, Jacob Garfin, Matt Plumb, and Xiong Wang
EPI_ISL_2332863, EPI_ISL_2332882, EPI_ISL_2332892, EPI_ISL_2332895, EPI_ISL_2332910, EPI_ISL_2332912, EPI_ISL_2332927, EPI_ISL_2332930, EPI_ISL_2332935, EPI_ISL_2332957, EPI_ISL_2332958, EPI_ISL_2332996, EPI_ISL_2332999, EPI_ISL_2333015, EPI_ISL_2333058			
see above	UW Virology Lab	UW Virology Lab	Pavitra Roychoudhury, Hong Xie, Lasata Shrestha, Tien V. Nguyen, Shah Mohamed Bakhash, Michelle Lin, Noah R. Baker, Ricardo Perez, Sean Ellis, Nathan Breit, Robert J. Livingston, Meei-Li Huang, Keith R Jerome, Patrick Mathias, Alexander Greninger
EPI_ISL_2335092, EPI_ISL_2335094	University of Michigan Clinical Microbiology Laboratory	Lauring Lab, University of Michigan, Department of Microbiology and Immunology	Gilbert
EPI_ISL_2336195, EPI_ISL_2336198, EPI_ISL_2336219, EPI_ISL_2336221	Washington State Department of Health Public Health Laboratories	Washington State Department of Health Public Health Laboratories	Drew MacKellar, Philip Dykema, Denny Russell, Joenice Gonzalez, Hannah Gray, Geoff Melly, Vanessa De Los Santos, Darren Lucas, JohnAric Peterson, Avi Singh, Rebecca Cao
EPI_ISL_2339333, EPI_ISL_2339341	Oregon State Public Health Laboratory	Oregon State Public Health Laboratory	Rafia Razzaque, Eugene Yeboah, Vanda Makris, Laura Tsaknaridis, John Fontana and Shane Sevey
EPI_ISL_2339395	Illinois Department of Public Health - Springfield Lab	Illinois Department of Public Health - Springfield Lab	Bryan Sim, Gordon McCall
EPI_ISL_2339997, EPI_ISL_2340022	North Dakota Department of Health, Public Health Laboratory	North Dakota Department of Health, Public Health Laboratory	Lisa Wingerter
EPI_ISL_2340691, EPI_ISL_2340704, EPI_ISL_2340714, EPI_ISL_2340724, EPI_ISL_2340732, EPI_ISL_2340734, EPI_ISL_2340740, EPI_ISL_2340742, EPI_ISL_2340749, EPI_ISL_2340752, EPI_ISL_2340783, EPI_ISL_2340784, EPI_ISL_2340791, EPI_ISL_2340795, EPI_ISL_2340796			
see above	UW Virology Lab	UW Virology Lab	Pavitra Roychoudhury, Hong Xie, Lasata Shrestha, Tien V. Nguyen, Shah Mohamed Bakhash, Michelle Lin, Noah R. Baker, Sean Ellis, Meei-Li Huang, Keith R Jerome, Alexander Greninger
EPI_ISL_2341035	Salud Digna	Instituto Nacional de Medicina Genomica	Hidalgo-Miranda A, Cedro-Tanda A, Cisneros-Villanueva M, Orjuela-Rodríguez M, Mendoza-Vargas A, Reyes-Grajeda JP, Abraham Campos-Romero, Moreno-Camacho José Luis, Rodriguez-Gallegos Jorge, Luna-Ruiz Marco, Gonzalez-Barrera D, Rangel-DeLeon D, Munguia-Garza P, Ramirez-Vega O, Escobar-Arrazola, M, Herrera-Montalvo LA.
EPI_ISL_2344511	Virginia Division of Consolidated Laboratory Services	Virginia Division of Consolidated Laboratory Services	Virginia DCLS
EPI_ISL_2348932, EPI_ISL_2348934, EPI_ISL_2348935	Oregon State Public Health Laboratory	Oregon State Public Health Laboratory	Rafia Razzaque, Eugene Yeboah, Vanda Makris, Laura Tsaknaridis, John Fontana and Shane Sevey
EPI_ISL_2349901, EPI_ISL_2349923, EPI_ISL_2349993	Salud Digna	Instituto Nacional de Medicina Genomica	Hidalgo-Miranda A, Cedro-Tanda A, Mendoza-Vargas A, Reyes-Grajeda JP, Abraham Campos-Romero, Moreno-Camacho José Luis, Rodriguez-Gallegos Jorge, Luna-Ruiz Marco, Gonzalez-Barrera D, Rangel-DeLeon D, Munguia-Garza P, Ramirez-Vega O, Escobar-Arrazola, M, Herrera-Montalvo LA.
EPI_ISL_2367807, EPI_ISL_2367809, EPI_ISL_2367903, EPI_ISL_2367909, EPI_ISL_2367934	Quest Diagnostics Incorporated	Centers for Disease Control and Prevention Division of Viral Diseases, Pathogen Discovery	Dakota Howard, Dhwani Batra, Peter W. Cook, Kara Moser, Adrian Paskey, Jason Caravas, Benjamin Rambo-Martin, Shatavia Morrison, Christopher Gulvick, Scott Sammons, Yvette Unoarumhi, Darlene Wagner, Matthew Schmerer, S. H. Rosenthal, A. Gerasimova, R. M. Kagan, B. Anderson, M. Hua, Y. Liu, L.E. Bernstein, K.E. Livingston, A. Perez, I. A. Shlyakhter, R. V. Rolando, R. Owen, P. Tanpaiboon, F. Lacbawan, Clinton R. Paden, Duncan MacCannell
EPI_ISL_2368136, EPI_ISL_2368200, EPI_ISL_2368201	Fulgent Genetics	Centers for Disease Control and Prevention Division of Viral Diseases, Pathogen Discovery	Dakota Howard, Dhwani Batra, Peter W. Cook, Kara Moser, Adrian Paskey, Jason Caravas, Benjamin Rambo-Martin, Shatavia Morrison, Christopher Gulvick, Scott Sammons, Yvette Unoarumhi, Darlene Wagner, Matthew Schmerer, Harry Gao, Mickey Li, John Gao, Joseph Fierro, Benafsh Sapra, Becky Tsai, Yan Meng, Doreen Ng, James Xie, Clinton R. Paden, Duncan MacCannell
EPI_ISL_2368275, EPI_ISL_2368400, EPI_ISL_2368409, EPI_ISL_2368699	Aegis Sciences Corporation	Centers for Disease Control and Prevention Division of Viral Diseases, Pathogen Discovery	Dakota Howard, Dhwani Batra, Peter W. Cook, Kara Moser, Adrian Paskey, Jason Caravas, Benjamin Rambo-Martin, Shatavia Morrison, Christopher Gulvick, Scott Sammons, Yvette Unoarumhi, Darlene Wagner, Matthew Schmerer, Cyndi Clark, Patrick Campbell, Rob Case, Vikramsinha Ghorpade, Holly Houdeshell, Ola Kvalvaag, Dillon Nall, Ethan Sanders, Alec Vest, Shaun Westlund, Matthew Hardison, Clinton R. Paden, Duncan MacCannell

EPI_ISL_2369028, EPI_ISL_2369192, EPI_ISL_2369416	Quest Diagnostics Incorporated	Centers for Disease Control and Prevention Division of Viral Diseases, Pathogen Discovery	Dakota Howard, Dhwani Batra, Peter W. Cook, Kara Moser, Adrian Paskey, Jason Caravas, Benjamin Rambo-Martin, Shatavia Morrison, Christopher Gulvick, Scott Sammons, Yvette Unoarumhi, Darlene Wagner, Matthew Schmerer, S. H. Rosenthal, A. Gerasimova, R. M. Kagan, B. Anderson, M. Hua, Y. Liu, L.E. Bernstein, K.E. Livingston, A. Perez, I. A. Shlyakhter, R. V. Rolando, R. Owen, P. Tanpaiboon, F. Lacbawan, Clinton R. Paden, Duncan MacCannell
EPI_ISL_2370054, EPI_ISL_2370117, EPI_ISL_2370530, EPI_ISL_2370536	Aegis Sciences Corporation	Centers for Disease Control and Prevention Division of Viral Diseases, Pathogen Discovery	Dakota Howard, Dhwani Batra, Peter W. Cook, Kara Moser, Adrian Paskey, Jason Caravas, Benjamin Rambo-Martin, Shatavia Morrison, Christopher Gulvick, Scott Sammons, Yvette Unoarumhi, Darlene Wagner, Matthew Schmerer, Cyndi Clark, Patrick Campbell, Rob Case, Vikramsinha Ghorpade, Holly Houdeshell, Ola Kvalvaag, Dillon Nall, Ethan Sanders, Alec Vest, Shaun Westlund, Matthew Hardison, Clinton R. Paden, Duncan MacCannell
EPI_ISL_2371560, EPI_ISL_2371568, EPI_ISL_2371572, EPI_ISL_2371583, EPI_ISL_2371671	Fulgent Genetics	Centers for Disease Control and Prevention Division of Viral Diseases, Pathogen Discovery	Dakota Howard, Dhwani Batra, Peter W. Cook, Kara Moser, Adrian Paskey, Jason Caravas, Benjamin Rambo-Martin, Shatavia Morrison, Christopher Gulvick, Scott Sammons, Yvette Unoarumhi, Darlene Wagner, Matthew Schmerer, Harry Gao, Mickey Li, John Gao, Joseph Fierro, Benafsh Sapra, Becky Tsai, Yan Meng, Doreen Ng, James Xie, Clinton R. Paden, Duncan MacCannell
EPI_ISL_2371748, EPI_ISL_2372141, EPI_ISL_2372158, EPI_ISL_2372182, EPI_ISL_2372228	Quest Diagnostics Incorporated	Centers for Disease Control and Prevention Division of Viral Diseases, Pathogen Discovery	Dakota Howard, Dhwani Batra, Peter W. Cook, Kara Moser, Adrian Paskey, Jason Caravas, Benjamin Rambo-Martin, Shatavia Morrison, Christopher Gulvick, Scott Sammons, Yvette Unoarumhi, Darlene Wagner, Matthew Schmerer, S. H. Rosenthal, A. Gerasimova, R. M. Kagan, B. Anderson, M. Hua, Y. Liu, L.E. Bernstein, K.E. Livingston, A. Perez, I. A. Shlyakhter, R. V. Rolando, R. Owen, P. Tanpaiboon, F. Lacbawan, Clinton R. Paden, Duncan MacCannell
EPI_ISL_2375569, EPI_ISL_2375583, EPI_ISL_2375651, EPI_ISL_2375775	Infinity Biologix	Centers for Disease Control and Prevention Division of Viral Diseases, Pathogen Discovery	Dakota Howard, Dhwani Batra, Peter W. Cook, Kara Moser, Adrian Paskey, Jason Caravas, Benjamin Rambo-Martin, Shatavia Morrison, Christopher Gulvick, Scott Sammons, Yvette Unoarumhi, Darlene Wagner, Matthew Schmerer, Christian Bixby, Yihe Wang, Jonathan Schultz, Chirayu Goswami, Russ Hager, Robin Grimwood, Clinton R. Paden, Duncan MacCannell
EPI_ISL_2376012, EPI_ISL_2376013, EPI_ISL_2376014, EPI_ISL_2376015, EPI_ISL_2376016	Alaska State Virology Laboratory	Alaska State Virology Laboratory	Stephanie DeRonde, Elva House, Jacob Zidek, Lisa Smith, Ph.D., Jack Chen, Ph.D.
EPI_ISL_2376248, EPI_ISL_2376258, EPI_ISL_2376259	Gundersen Clinical Microbiology Laboratory	Kabara Cancer Research Institute	Craig S. Richmond, Paraic A. Kenny
EPI_ISL_2378556, EPI_ISL_2378568, EPI_ISL_2378583, EPI_ISL_2378587, EPI_ISL_2378590, EPI_ISL_2378593	State Testing Facility	Altius Institute for Biomedical Research	Daniel Bates, Rebecca Bruders, Michael Buckley, Mark Frerker, Amanda Gale, Clem Green, Muhammad Halimun, Kneshay Harper, Matt Hartman, Alex Isner, Audra Johnson, Jessica Kunder, Lauren Mitchell, Jemma Nelson, Alex Nguyen, Sofia Olsson, Sadie Patraw, Tobias Ragoczy, Joshua Richards, Jean Robinston, Jacob Rodriguez, John Stamatoyannopoulos, Eric Thorland, Julia Wald
EPI_ISL_2378837, EPI_ISL_2378878, EPI_ISL_2378888	Washington State Department of Health Public Health Laboratories	Washington State Department of Health Public Health Laboratories	Drew MacKellar, Philip Dykema, Denny Russell, Joenice Gonzalez, Hannah Gray, Geoff Melly, Vanessa De Los Santos, Darren Lucas, JohnAric Peterson, Avi Singh, Rebecca Cao
EPI_ISL_2381933, EPI_ISL_2381934, EPI_ISL_2381940	New Mexico Department of Health Scientific Laboratory	New Mexico Department of Health Scientific Laboratory	Ellie Johnson,D'eldra Malone, Jennifer Benoit, Ratheesh Rajan, Linda Salazar, Keila Gutierrez, Anastacia Griego-Fisher
EPI_ISL_2384011	MT Public Health Laboratory	Centers for Disease Control and Prevention Division of Viral Diseases, Pathogen Discovery	Mili Sheth, Sarah Nobles, Jasmine Padilla, Mark Burroughs, Shoshona Le, Katie Dillon, Peter Cook, Clinton R. Paden, Dhwani Batra, Krista Queen, Kristen Knipe, Dakota Howard, Yvette Unoarumhi, Darlene Wagner, Matthew Schmerer, Ben L. Rambo-Martin, Kristine Lacek, Sam Shepard, Alison Laufer Halpin, Dave Wentworth, Vivien Dugan, Suxiang Tong, Justin Lee
EPI_ISL_2388567	LabKom - Labor Augsburg MVZ GmbH	Robert Koch Institute	unknown
EPI_ISL_2391329	Genetica Molecular and Subdepartamento de Virologia ISP Chile	Instituto de Salud Publica de Chile	Karen Orostica, Constanza Campano, Barbara Parra, Loredana Arata, Gisselle Barra, Patricia Bustos, Rodrigo Fasce, Javier Tognarelli, Andres Castillo, Soledad Ulloa, Jorge Fernandez
EPI_ISL_2397427, EPI_ISL_2397608, EPI_ISL_2397718	Quest Diagnostics Incorporated	Centers for Disease Control and Prevention Division of Viral Diseases, Pathogen Discovery	Dakota Howard, Dhwani Batra, Peter W. Cook, Kara Moser, Adrian Paskey, Jason Caravas, Benjamin Rambo-Martin, Shatavia Morrison, Christopher Gulvick, Scott Sammons, Yvette Unoarumhi, Darlene Wagner, Matthew Schmerer, S. H. Rosenthal, A. Gerasimova, R. M. Kagan, B. Anderson, M. Hua, Y. Liu, L.E. Bernstein, K.E. Livingston, A. Perez, I. A. Shlyakhter, R. V. Rolando, R. Owen, P. Tanpaiboon, F. Lacbawan, Clinton R. Paden, Duncan MacCannell
EPI_ISL_2398161	Laboratory Corporation of America	Centers for Disease Control and Prevention Division of Viral Diseases, Pathogen Discovery	Dakota Howard, Dhwani Batra, Peter W. Cook, Kara Moser, Adrian Paskey, Jason Caravas, Benjamin Rambo-Martin, Shatavia Morrison, Christopher Gulvick, Scott Sammons, Yvette Unoarumhi, Darlene Wagner, Matthew Schmerer, Minoo Agarwal, Eyad Almasri, Debbie Boles, Ayla Burns, Nuthawin Charoensri, Oren Cohen, Susan Countryman, Mary Ann Cristobal, Bobbi Croy, Suzanne Dale, Hrushikesh Deshmukh, Amanda Douglas, Vincent Drouillon, Marcia Eisenberg, Howard Engler, Rama Ghatti, Prashant Gupta, Susan Hicks, Jake Humphrey, Lax Iyer, Lisa Pfefferle, Manoj Jain, Matthew Robinson, Mohan Kolli, Brian Krueger, Tim Kuphal, Stanley Letovsky, Michael Levandoski, Craig Lukasik, Jonathan Meltzer, Brian Norvell, Mindy Nye, Scott Parker, Christos Petropoulos, John Pruitt, Steven Ragan, Scott Ryan, Mike Sapeta, Jana Schroth, Suresh Babu Selvaraju, Goran Stevovic, Amanda Suchanek, Andrea Throop, Lyndon Tilson, Thomas Urban, Joe Voshell, Kimberly Wagner, Jonathan Williams, Mary Williamson, Qian Zeng, Tricia Zwiefelhofer, Clinton R. Paden, Duncan MacCannell
EPI_ISL_2403718	Minnesota Department of Health, Public Health Laboratory	Minnesota Department of Health, Public Health Laboratory	Alexandra Lorentz, Jacob Garfin, Matt Plumb, and Xiong Wang
EPI_ISL_2404749	Oregon State Public Health Laboratory	Oregon State Public Health Laboratory	Rafia Razzaque, Eugene Yeboah, Vanda Makris, Laura Tsaknaridis, John Fontana and Shane Sevey
EPI_ISL_2405202	Pomona Valley Hospital Medical Center	Los Angeles County PHL	P. Hemarajata et al.
EPI_ISL_2421151	URMC LABS	Wadsworth Center, New York State Department of Health	Kirsten St. George, Daryl M. Lamson, Alexis Russell, Matthew Shudt, Melissa A Leisner, Jonathan Plitnick, Catharine Prussing, Navjot Singh, John Kelly, Erasmus Schneider, Erica Lasek-Nesselquist
EPI_ISL_2422114	Aegis Sciences Corporation	Centers for Disease Control and Prevention Division of Viral Diseases, Pathogen Discovery	Dakota Howard, Dhwani Batra, Peter W. Cook, Kara Moser, Adrian Paskey, Jason Caravas, Benjamin Rambo-Martin, Shatavia Morrison, Christopher Gulvick, Scott Sammons, Yvette Unoarumhi, Darlene Wagner, Matthew Schmerer, Cyndi Clark, Patrick Campbell, Rob Case, Vikramsinha Ghorpade, Holly Houdeshell, Ola Kvalvaag, Dillon Nall, Ethan Sanders, Alec Vest, Shaun Westlund, Matthew Hardison, Clinton R. Paden, Duncan MacCannell
EPI_ISL_2423015, EPI_ISL_2423016	San Diego County Public Health Laboratory	Andersen lab at Scripps Research	SEARCH Alliance San Diego with Ashleigh Murphy, Jovan Shephard, Brett Austin
EPI_ISL_2423551	TXDSHS	TXDSHS	Rashmi Tuladhar, Bonnie Oh, Jenny Zhang, Maliha Rahman, Mayela Pedrueza, Anita Pokharel, Karen Bobier, Lorraine Rodriguez, Myong Koag, Chun Wang, Rachel Lee, Grace Kubin
EPI_ISL_2424189, EPI_ISL_2424190	UCLA Clinical Micro Lab	Los Angeles County PHL	P. Hemarajata et al.
EPI_ISL_2424239, EPI_ISL_2424240	Oregon State Public Health Laboratory	Oregon State Public Health Laboratory	Rafia Razzaque, Eugene Yeboah, Vanda Makris, Laura Tsaknaridis, John Fontana and Shane Sevey
EPI_ISL_2425402, EPI_ISL_2425403	M Health Fairview	Minnesota Department of Health, Public Health Laboratory	Alexandra Lorentz, Jacob Garfin, Matt Plumb, and Xiong Wang
EPI_ISL_2426294, EPI_ISL_2426305, EPI_ISL_2426308, EPI_ISL_2426380	UW Virology Lab	UW Virology Lab	Pavitra Roychoudhury, Hong Xie, Lasata Shrestha, Tien V. Nguyen, Shah Mohamed Bakhash, Michelle Lin, Noah R. Baker, Ricardo Perez, Sean Ellis, Nathan Breit, Robert J. Livingston, Meei-Li Huang, Keith R Jerome, Patrick Mathias, Alexander Greninger
EPI_ISL_2426416, EPI_ISL_2426455, EPI_ISL_2426516	UW Virology Lab	UW Virology Lab	Pavitra Roychoudhury, Hong Xie, Lasata Shrestha, Tien V. Nguyen, Shah Mohamed Bakhash, Michelle Lin, Noah R. Baker, Sean Ellis, Meei-Li Huang, Keith R Jerome, Alexander Greninger
EPI_ISL_2432618, EPI_ISL_2432695, EPI_ISL_2432734, EPI_ISL_2432763, EPI_ISL_2432790, EPI_ISL_2432819, EPI_ISL_2432824, EPI_ISL_2432832, EPI_ISL_2432833	UW Virology Lab	UW Virology Lab	Pavitra Roychoudhury, Hong Xie, Lasata Shrestha, Tien V. Nguyen, Shah Mohamed Bakhash, Michelle Lin, Noah R. Baker, Ricardo Perez, Sean Ellis, Nathan Breit, Robert J. Livingston, Meei-Li Huang, Keith R Jerome, Patrick Mathias, Alexander Greninger
EPI_ISL_2432845, EPI_ISL_2432861, EPI_ISL_2432865, EPI_ISL_2432871, EPI_ISL_2432895, EPI_ISL_2432905	UW Virology Lab	UW Virology Lab	Pavitra Roychoudhury, Hong Xie, Lasata Shrestha, Tien V. Nguyen, Shah Mohamed Bakhash, Michelle Lin, Noah R. Baker, Sean Ellis, Meei-Li Huang, Keith R Jerome, Alexander Greninger

EPI_ISL_2439165	IN State Department of Health Laboratory Services	IN State Department of Health Laboratory Services	Cassandra Campion, Jamie Yeadon, Brian Pope, Lixia Liu, Kyle Brownlee, Melissa Hindenlang, Mark Glazier
EPI_ISL_2439415, EPI_ISL_2439559, EPI_ISL_2439616, EPI_ISL_2439773, EPI_ISL_2439793	Quest Diagnostics Incorporated	Centers for Disease Control and Prevention Division of Viral Diseases, Pathogen Discovery	Dakota Howard, Dhwani Batra, Peter W. Cook, Kara Moser, Adrian Paskey, Jason Caravas, Benjamin Rambo-Martin, Shatavia Morrison, Christopher Gulvick, Scott Sammons, Yvette Unoarumhi, Darlene Wagner, Matthew Schmerer, S. H. Rosenthal, A. Gerasimova, R. M. Kagan, B. Anderson, M. Hua, Y. Liu, L.E. Bernstein, K.E. Livingston, A. Perez, I. A. Shlyakhter, R. V. Rolando, R. Owen, P. Tanpaiboon, F. Lacbawan, Clinton R. Paden, Duncan MacCannell
EPI_ISL_2440207	Helix/Illumina	Centers for Disease Control and Prevention Division of Viral Diseases, Pathogen Discovery	Dakota Howard, Dhwani Batra, Peter W. Cook, Kara Moser, Adrian Paskey, Jason Caravas, Benjamin Rambo-Martin, Shatavia Morrison, Christopher Gulvick, Scott Sammons, Yvette Unoarumhi, Darlene Wagner, Matthew Schmerer, Eileen de Feo, Jan Antico, Christine Tran, Matthew Tolentino, Shannon Wickline, Kim Gietzen, Brad Sickler, Jingtao Liu, Eric Allen, Phil Febbo, Nicole L. Washington, Simon White, Geraint Levan, Kelly Schiabor Barrett, Elizabeth Cirulli, Alexandre Bolze, Ary Ascencio, Charlotte Rivera-Garcia, Ryan Cho, Jason Nguyen, Sherry Wang, Jimmy Ramirez, Tyler Cassens, Efren Sandoval, Magnus Isaksson, William Lee, David Becker, Marc Laurent, James Lu, Clinton R. Paden, Duncan MacCannell
EPI_ISL_2440622	Quest Diagnostics Incorporated	Centers for Disease Control and Prevention Division of Viral Diseases, Pathogen Discovery	Dakota Howard, Dhwani Batra, Peter W. Cook, Kara Moser, Adrian Paskey, Jason Caravas, Benjamin Rambo-Martin, Shatavia Morrison, Christopher Gulvick, Scott Sammons, Yvette Unoarumhi, Darlene Wagner, Matthew Schmerer, S. H. Rosenthal, A. Gerasimova, R. M. Kagan, B. Anderson, M. Hua, Y. Liu, L.E. Bernstein, K.E. Livingston, A. Perez, I. A. Shlyakhter, R. V. Rolando, R. Owen, P. Tanpaiboon, F. Lacbawan, Clinton R. Paden, Duncan MacCannell
EPI_ISL_2440901	Mako Medical	Centers for Disease Control and Prevention Division of Viral Diseases, Pathogen Discovery	Dakota Howard, Dhwani Batra, Peter W. Cook, Kara Moser, Adrian Paskey, Jason Caravas, Benjamin Rambo-Martin, Shatavia Morrison, Christopher Gulvick, Scott Sammons, Yvette Unoarumhi, Darlene Wagner, Matthew Schmerer,Matthew Tugwell, Lauren Moon, Clinton R. Paden, Duncan MacCannell
EPI_ISL_2441902, EPI_ISL_2441903	Fulgent Genetics	Centers for Disease Control and Prevention Division of Viral Diseases, Pathogen Discovery	Dakota Howard, Dhwani Batra, Peter W. Cook, Kara Moser, Adrian Paskey, Jason Caravas, Benjamin Rambo-Martin, Shatavia Morrison, Christopher Gulvick, Scott Sammons, Yvette Unoarumhi, Darlene Wagner, Matthew Schmerer, Harry Gao, Mickey Li, John Gao, Joseph Fierro, Benafsh Sapra, Becky Tsai, Yan Meng, Doreen Ng, James Xie, Clinton R. Paden, Duncan MacCannell
EPI_ISL_2443165, EPI_ISL_2443174	IN State Department of Health Laboratory Services	IN State Department of Health Laboratory Services	Cassandra Campion, Jamie Yeadon, Brian Pope, Lixia Liu, Kyle Brownlee, Melissa Hindenlang, Mark Glazier
EPI_ISL_2443292	TXDSHS	TXDSHS	Rashmi Tuladhar, Bonnie Oh, Jenny Zhang, Maliha Rahman, Mayela Pedrueza, Anita Pokharel, Karen Bobier, Lorraine Rodriguez, Myong Koag, Chun Wang, Rachel Lee, Grace Kubin
EPI_ISL_2443854	Minnesota Department of Health, Public Health Laboratory	Minnesota Department of Health, Public Health Laboratory	Alexandra Lorentz, Jacob Garfin, Matt Plumb, and Xiong Wang
EPI_ISL_2445619, EPI_ISL_2445632	Humboldt County Public Health Laboratory	Humboldt County Public Health Laboratory	Jeremy Corrigan
EPI_ISL_2451230	Mako Medical	Centers for Disease Control and Prevention Division of Viral Diseases, Pathogen Discovery	Dakota Howard, Dhwani Batra, Peter W. Cook, Kara Moser, Adrian Paskey, Jason Caravas, Benjamin Rambo-Martin, Shatavia Morrison, Christopher Gulvick, Scott Sammons, Yvette Unoarumhi, Darlene Wagner, Matthew Schmerer,Matthew Tugwell, Lauren Moon, Clinton R. Paden, Duncan MacCannell
EPI_ISL_2451300, EPI_ISL_2451330, EPI_ISL_2451376	Quest Diagnostics Incorporated	Centers for Disease Control and Prevention Division of Viral Diseases, Pathogen Discovery	Dakota Howard, Dhwani Batra, Peter W. Cook, Kara Moser, Adrian Paskey, Jason Caravas, Benjamin Rambo-Martin, Shatavia Morrison, Christopher Gulvick, Scott Sammons, Yvette Unoarumhi, Darlene Wagner, Matthew Schmerer, S. H. Rosenthal, A. Gerasimova, R. M. Kagan, B. Anderson, M. Hua, Y. Liu, L.E. Bernstein, K.E. Livingston, A. Perez, I. A. Shlyakhter, R. V. Rolando, R. Owen, P. Tanpaiboon, F. Lacbawan, Clinton R. Paden, Duncan MacCannell
EPI_ISL_2451592	NV-Southern Nevada Public Health Laboratory	Centers for Disease Control and Prevention Division of Viral Diseases, Pathogen Discovery	Mili Sheth, Sarah Nobles, Jasmine Padilla, Mark Burroughs, Shoshona Le, Katie Dillon, Peter Cook, Clinton R. Paden, Dhwani Batra, Krista Queen, Kristen Knipe, Dakota Howard, Yvette Unoarumhi, Darlene Wagner, Matthew Schmerer, Ben L. Rambo-Martin, Kristine Lacek, Sam Shepard, Alison Laufer Halpin, Dave Wentworth, Vivien Dugan, Suxiang Tong, Justin Lee
EPI_ISL_2451806	IL Dept. of Public Health Springfield Laboratory	Centers for Disease Control and Prevention Division of Viral Diseases, Pathogen Discovery	Mili Sheth, Sarah Nobles, Jasmine Padilla, Mark Burroughs, Shoshona Le, Katie Dillon, Peter Cook, Clinton R. Paden, Dhwani Batra, Krista Queen, Kristen Knipe, Dakota Howard, Yvette Unoarumhi, Darlene Wagner, Matthew Schmerer, Ben L. Rambo-Martin, Kristine Lacek, Sam Shepard, Alison Laufer Halpin, Dave Wentworth, Vivien Dugan, Suxiang Tong, Justin Lee
EPI_ISL_2451903	ND Dept. of Health Laboratory Services-Microbiology	Centers for Disease Control and Prevention Division of Viral Diseases, Pathogen Discovery	Mili Sheth, Sarah Nobles, Jasmine Padilla, Mark Burroughs, Shoshona Le, Katie Dillon, Peter Cook, Clinton R. Paden, Dhwani Batra, Krista Queen, Kristen Knipe, Dakota Howard, Yvette Unoarumhi, Darlene Wagner, Matthew Schmerer, Ben L. Rambo-Martin, Kristine Lacek, Sam Shepard, Alison Laufer Halpin, Dave Wentworth, Vivien Dugan, Suxiang Tong, Justin Lee
EPI_ISL_2451937, EPI_ISL_2451943	AK State Public Health Lab, State Health Department	Centers for Disease Control and Prevention Division of Viral Diseases, Pathogen Discovery	Mili Sheth, Sarah Nobles, Jasmine Padilla, Mark Burroughs, Shoshona Le, Katie Dillon, Peter Cook, Clinton R. Paden, Dhwani Batra, Krista Queen, Kristen Knipe, Dakota Howard, Yvette Unoarumhi, Darlene Wagner, Matthew Schmerer, Ben L. Rambo-Martin, Kristine Lacek, Sam Shepard, Alison Laufer Halpin, Dave Wentworth, Vivien Dugan, Suxiang Tong, Justin Lee
EPI_ISL_2451984, EPI_ISL_2451985	OR State PHL-Virology/Immunology Section	Centers for Disease Control and Prevention Division of Viral Diseases, Pathogen Discovery	Mili Sheth, Sarah Nobles, Jasmine Padilla, Mark Burroughs, Shoshona Le, Katie Dillon, Peter Cook, Clinton R. Paden, Dhwani Batra, Krista Queen, Kristen Knipe, Dakota Howard, Yvette Unoarumhi, Darlene Wagner, Matthew Schmerer, Ben L. Rambo-Martin, Kristine Lacek, Sam Shepard, Alison Laufer Halpin, Dave Wentworth, Vivien Dugan, Suxiang Tong, Justin Lee
EPI_ISL_2452125, EPI_ISL_2452134	HI Dept. of Health, State Laboratories Division	Centers for Disease Control and Prevention Division of Viral Diseases, Pathogen Discovery	Mili Sheth, Sarah Nobles, Jasmine Padilla, Mark Burroughs, Shoshona Le, Katie Dillon, Peter Cook, Clinton R. Paden, Dhwani Batra, Krista Queen, Kristen Knipe, Dakota Howard, Yvette Unoarumhi, Darlene Wagner, Matthew Schmerer, Ben L. Rambo-Martin, Kristine Lacek, Sam Shepard, Alison Laufer Halpin, Dave Wentworth, Vivien Dugan, Suxiang Tong, Justin Lee
EPI_ISL_2454335	TriCore Reference Laboratories	Center for Global Health, University of New Mexico Health Sciences Center	Daryl Domman, Kurt Schwalm, Valerie Morley, Cecilia Thompson, Kendra Pesko, Karissa Culbreath, Darrell Dinwiddie
EPI_ISL_2455437	Alaska State Virology Laboratory	Alaska State Virology Laboratory	Stephanie DeRonde, Elva House, Jacob Zidek, Lisa Smith, Ph.D., Jack Chen, Ph.D.
EPI_ISL_2456111, EPI_ISL_2456114, EPI_ISL_2456149, EPI_ISL_2456592, EPI_ISL_2458494, EPI_ISL_2458530, EPI_ISL_2458542, EPI_ISL_2458551, EPI_ISL_2458555, EPI_ISL_2458589, EPI_ISL_2458596, EPI_ISL_2458598, EPI_ISL_2458599, EPI_ISL_2458617, EPI_ISL_2458620			
see above	Utah Public Health Laboratory	Utah Public Health Laboratory	Erin L. Young, Kelly F. Oakeson, Tara Gallagher
EPI_ISL_2463209, EPI_ISL_2463213, EPI_ISL_2463229	Quest Diagnostics Incorporated	Centers for Disease Control and Prevention Division of Viral Diseases, Pathogen Discovery	Dakota Howard, Dhwani Batra, Peter W. Cook, Kara Moser, Adrian Paskey, Jason Caravas, Benjamin Rambo-Martin, Shatavia Morrison, Christopher Gulvick, Scott Sammons, Yvette Unoarumhi, Darlene Wagner, Matthew Schmerer, S. H. Rosenthal, A. Gerasimova, R. M. Kagan, B. Anderson, M. Hua, Y. Liu, L.E. Bernstein, K.E. Livingston, A. Perez, I. A. Shlyakhter, R. V. Rolando, R. Owen, P. Tanpaiboon, F. Lacbawan, Clinton R. Paden, Duncan MacCannell
EPI_ISL_2466048, EPI_ISL_2466049	Alaska State Virology Lab, Alaska State Public Health Labs	Alaska State Virology Lab, Alaska State Public Health Labs	DeRonde,S., House,E., Zidek,J., Redlinger,M., Smith,L., Chen,J.
EPI_ISL_2467527	GA Department of Public Health	GA Department of Public Health	Stacy Reeves, Jonathan Edwards, Cynthia Dixey, Tonia Parrott, Aliyah Fields, Taylor Smith
EPI_ISL_2472968, EPI_ISL_2472969, EPI_ISL_2472978, EPI_ISL_2472979, EPI_ISL_2473002, EPI_ISL_2473004, EPI_ISL_2473048, EPI_ISL_2473050, EPI_ISL_2473095, EPI_ISL_2473337, EPI_ISL_2473348, EPI_ISL_2473360, EPI_ISL_2473361, EPI_ISL_2473369, EPI_ISL_2473372, EPI_ISL_2473376, EPI_ISL_2473418, EPI_ISL_2473425, EPI_ISL_2473430, EPI_ISL_2473435, EPI_ISL_2473438, EPI_ISL_2473456, EPI_ISL_2473492, EPI_ISL_2473501, EPI_ISL_2473504, EPI_ISL_2473519, EPI_ISL_2473544, EPI_ISL_2473556, EPI_ISL_2473593, EPI_ISL_2473594, EPI_ISL_2473595, EPI_ISL_2473600, EPI_ISL_2473616, EPI_ISL_2473629, EPI_ISL_2473636, EPI_ISL_2473640, EPI_ISL_2473641, EPI_ISL_2473651, EPI_ISL_2473652			
see above	State Testing Facility	Altius Institute for Biomedical Research	Daniel Bates, Rebecca Bruders, Michael Buckley, Mark Frerker, Amanda Gale, Clem Green, Muhammad Halimun, Kneshay Harper, Matt Hartman, Alex Isner, Audra Johnson, Jessica Kunder, Lauren Mitchell, Jemma Nelson, Alex Nguyen, Sofia Olsson, Sadie Patraw, Tobias Ragoczy, Joshua Richards, Jean Robinston, Jacob Rodriguez, John Stamatoyannopoulos, Eric Thorland, Julia Wald
EPI_ISL_2473676	Oregon State Public Health Laboratory	Oregon State Public Health Laboratory	Rafia Razzaque, Eugene Yeboah, Vanda Makris, Laura Tsaknaridis, John Fontana and Shane Sevey
EPI_ISL_2476441	LESP Chihuahua	Instituto de Diagnostico y Referencia Epidemiologicos (INDRE)	Claudia Wong-Arambula, Abril Rodriguez-Maldonado, Vanessa Rivero-Arredondo, Ariadna Medina-Benitez, Joaquin Quiroz-Mercado, Sergio Rangel-Guerrero, Natividad Cruz-Ortiz, Tatiana Nunez-Garcia, Gisela Barrera-Badillo, Lucia Hernandez-Rivas, Irma Lopez-Martinez, Ernesto Ramirez-Gonzalez.
EPI_ISL_2476442	LESP Campeche	Instituto de Diagnostico y Referencia Epidemiologicos (INDRE)	Claudia Wong-Arambula, Abril Rodriguez-Maldonado, Vanessa Rivero-Arredondo, Ariadna Medina-Benitez, Joaquin Quiroz-Mercado, Sergio Rangel-Guerrero, Natividad Cruz-Ortiz, Tatiana Nunez-Garcia, Gisela Barrera-Badillo, Lucia Hernandez-Rivas, Irma Lopez-Martinez, Ernesto Ramirez-Gonzalez.
EPI_ISL_2476443	LESP Baja California	Instituto de Diagnostico y Referencia Epidemiologicos	Claudia Wong-Arambula, Abril Rodriguez-Maldonado, Vanessa Rivero-Arredondo, Ariadna Medina-Benitez, Joaquin Quiroz-Mercado, Sergio

		(INDRE)	Rangel-Guerrero, Natividad Cruz-Ortiz, Tatiana Nunez-Garcia, Gisela Barrera-Badillo, Lucia Hernandez-Rivas, Irma Lopez-Martinez, Ernesto Ramirez-Gonzalez.
EPI_ISL_2476444	LESP Sinaloa	Instituto de Diagnostico y Referencia Epidemiologicos (INDRE)	Claudia Wong-Arambula, Abril Rodriguez-Maldonado, Vanessa Rivero-Arredondo, Ariadna Medina-Benitez, Joaquin Quiroz-Mercado, Sergio Rangel-Guerrero, Natividad Cruz-Ortiz, Tatiana Nunez-Garcia, Gisela Barrera-Badillo, Lucia Hernandez-Rivas, Irma Lopez-Martinez, Ernesto Ramirez-Gonzalez.
EPI_ISL_2479894, EPI_ISL_2479898	LESP Nuevo Leon	Instituto de Diagnostico y Referencia Epidemiologicos (INDRE)	Claudia Wong-Arambula, Abril Rodriguez-Maldonado, Vanessa Rivero-Arredondo, Ariadna Medina-Benitez, Joaquin Quiroz-Mercado, Sergio Rangel-Guerrero, Natividad Cruz-Ortiz, Tatiana Nunez-Garcia, Gisela Barrera-Badillo, Lucia Hernandez-Rivas, Irma Lopez-Martinez, Ernesto Ramirez-Gonzalez.
EPI_ISL_2479906	LESP Tamaulipas	Instituto de Diagnostico y Referencia Epidemiologicos (INDRE)	Claudia Wong-Arambula, Abril Rodriguez-Maldonado, Vanessa Rivero-Arredondo, Ariadna Medina-Benitez, Joaquin Quiroz-Mercado, Sergio Rangel-Guerrero, Natividad Cruz-Ortiz, Tatiana Nunez-Garcia, Gisela Barrera-Badillo, Lucia Hernandez-Rivas, Irma Lopez-Martinez, Ernesto Ramirez-Gonzalez.
EPI_ISL_2480128	Fulgent Genetics	Centers for Disease Control and Prevention Division of Viral Diseases, Pathogen Discovery	Dakota Howard, Dhwani Batra, Peter W. Cook, Kara Moser, Adrian Paskey, Jason Caravas, Benjamin Rambo-Martin, Shatavia Morrison, Christopher Gulvick, Scott Sammons, Yvette Unoarumhi, Darlene Wagner, Matthew Schmerer, Harry Gao, Mickey Li, John Gao, Joseph Fierro, Benafsh Sapra, Becky Tsai, Yan Meng, Doreen Ng, James Xie, Clinton R. Paden, Duncan MacCannell
EPI_ISL_2480209, EPI_ISL_2480293, EPI_ISL_2481627, EPI_ISL_2481707, EPI_ISL_2481730, EPI_ISL_2482011, EPI_ISL_2482056, EPI_ISL_2482167, EPI_ISL_2482257	Laboratory Corporation of America	Centers for Disease Control and Prevention Division of Viral Diseases, Pathogen Discovery	Dakota Howard, Dhwani Batra, Peter W. Cook, Kara Moser, Adrian Paskey, Jason Caravas, Benjamin Rambo-Martin, Shatavia Morrison, Christopher Gulvick, Scott Sammons, Yvette Unoarumhi, Darlene Wagner, Matthew Schmerer, Minoo Agarwal, Eyad Almasri, Debbie Boles, Ayla Burns, Nuthawin Charoensri, Oren Cohen, Susan Countryman, Mary Ann Cristobal, Bobbi Croy, Suzanne Dale, Hrushikesh Deshmukh, Amanda Douglas, Vincent Drouillon, Marcia Eisenberg, Howard Engler, Rama Ghatti, Prashant Gupta, Susan Hicks, Jake Humphrey, Lax Iyer, Lisa Pfefferle, Manoj Jain, Matthew Robinson, Mohan Kolli, Brian Krueger, Tim Kuphal, Stanley Letovsky, Michael Levandoski, Craig Lukasik, Jonathan Meltzer, Brian Norvell, Mindy Nye, Scott Parker, Christos Petropoulos, John Pruitt, Steven Ragan, Scott Ryan, Mike Sapeta, Jana Schroth, Suresh Babu Selvaraju, Goran Stevovic, Amanda Suchanek, Andrea Throop, Lyndon Tilson, Thomas Urban, Joe Voshell, Kimberly Wagner, Jonathan Williams, Mary Williamson, Qian Zeng, Tricia Zwiefelhofer, Clinton R. Paden, Duncan MacCannell
EPI_ISL_2488881, EPI_ISL_2488892, EPI_ISL_2488929, EPI_ISL_2488968, EPI_ISL_2489018, EPI_ISL_2489031	Mako Medical	Centers for Disease Control and Prevention Division of Viral Diseases, Pathogen Discovery	Dakota Howard, Dhwani Batra, Peter W. Cook, Kara Moser, Adrian Paskey, Jason Caravas, Benjamin Rambo-Martin, Shatavia Morrison, Christopher Gulvick, Scott Sammons, Yvette Unoarumhi, Darlene Wagner, Matthew Schmerer,Matthew Tugwell, Lauren Moon, Clinton R. Paden, Duncan MacCannell
EPI_ISL_2489072, EPI_ISL_2489133, EPI_ISL_2489160, EPI_ISL_2489349, EPI_ISL_2489450, EPI_ISL_2489458, EPI_ISL_2489492	Aegis Sciences Corporation	Centers for Disease Control and Prevention Division of Viral Diseases, Pathogen Discovery	Dakota Howard, Dhwani Batra, Peter W. Cook, Kara Moser, Adrian Paskey, Jason Caravas, Benjamin Rambo-Martin, Shatavia Morrison, Christopher Gulvick, Scott Sammons, Yvette Unoarumhi, Darlene Wagner, Matthew Schmerer, Cyndi Clark, Patrick Campbell, Rob Case, Vikramsinha Ghorpade, Holly Houdeshell, Ola Kvalvaag, Dillon Nall, Ethan Sanders, Alec Vest, Shaun Westlund, Matthew Hardison, Clinton R. Paden, Duncan MacCannell
EPI_ISL_2489790, EPI_ISL_2489792	State Testing Facility	Altius Institute for Biomedical Research	Daniel Bates, Rebecca Bruders, Michael Buckley, Mark Frerker, Amanda Gale, Clem Green, Muhammad Halimun, Kneshay Harper, Matt Hartman, Alex Isner, Audra Johnson, Jessica Kunder, Lauren Mitchell, Jemma Nelson, Alex Nguyen, Sofia Olsson, Sadie Patraw, Tobias Ragoczy, Joshua Richards, Jean Robinston, Jacob Rodriguez, John Stamatoyannopoulos, Eric Thorland, Julia Wald
EPI_ISL_2490046, EPI_ISL_2490077	Seattle Flu Study	Seattle Flu Study	Deborah A. Nickerson, Chris D. Frazar, Jover Lee, Benjamin Pelle, Erica Ryke, Matthew Richardson, Amanda Adler, Elisabeth Brandstetter, Peter D. Han, Kairsten Fay, Misja Ilcisin, Kirsten Lacombe, Thomas R. Sibley, Melissa Truong, Caitlin R. Wolf, Karen Cowgill, Stephanie Schrag, Jeff Duchin, Michael Boeckh, Janet A. Englund, Michael Famulare, Barry R. Lutz, Mark J. Rieder, Lea M. Starita, Matthew Thompson, Helen Y. Chu, Trevor Bedford, Jay Shendure
EPI_ISL_2490181, EPI_ISL_2490198	Northwest Laboratory	Seattle Flu Study	Deborah A. Nickerson, Chris D. Frazar, Jover Lee, Benjamin Pelle, Erica Ryke, Matthew Richardson, Amanda Adler, Elisabeth Brandstetter, Peter D. Han, Kairsten Fay, Misja Ilcisin, Kirsten Lacombe, Thomas R. Sibley, Melissa Truong, Caitlin R. Wolf, Romesh Gautom, Geoff Melly, Brian Hiatt, Philip Dykema, Scott Lindquist, Michael Boeckh, Janet A. Englund, Michael Famulare, Barry R. Lutz, Mark J. Rieder, Lea M. Starita, Matthew Thompson, Helen Y. Chu, Jay Shendure, Trevor Bedford
EPI_ISL_2490266, EPI_ISL_2490289	Incyte Diagnostics Spokane	Seattle Flu Study	Deborah A. Nickerson, Chris D. Frazar, Jover Lee, Benjamin Pelle, Erica Ryke, Matthew Richardson, Amanda Adler, Elisabeth Brandstetter, Peter D. Han, Kairsten Fay, Misja Ilcisin, Kirsten Lacombe, Thomas R. Sibley, Melissa Truong, Caitlin R. Wolf, Romesh Gautom, Geoff Melly, Brian Hiatt, Philip Dykema, Scott Lindquist, Michael Boeckh, Janet A. Englund, Michael Famulare, Barry R. Lutz, Mark J. Rieder, Lea M. Starita, Matthew Thompson, Helen Y. Chu, Jay Shendure, Trevor Bedford
EPI_ISL_2490394, EPI_ISL_2490409, EPI_ISL_2490411, EPI_ISL_2490445, EPI_ISL_2490457, EPI_ISL_2490462	Centro de Investigación Biomédica del Noreste (CIBIN)	Centro de Investigación en Enfermedades Infecciosas (CIENI), Instituto Nacional de Enfermedades Respiratorias (INER)	Consorcio Mexicano de Vigilancia Genómica (CoViGen-Mex). Authors (in alphabetical order): Julio Elias Alvarado-Yaah, Carlos F. Arias, Santiago Ávila-Ríos, Eduardo Becerril-Vargas, Víctor Hugo Borja-Aburto, Celia Boukadida, Cristóbal Cháidez-Quiróz, Juan Bautista Chale-Dzul, Ricardo Ciria Merce, Andreu Comas-García, Célida Duque Molina, Julissa Enciso-Ibarra, José Antonio Enciso-Moreno, Gloria Elena Espinosa-Ayala, Fernando Fontove-Herrera, Daniel Fregoso-Rueda, Victor Eduardo Garcia-Arias, Alejandra Garcia-Gasca, Bruno Gomez-Gil, Jean Pierre González, Irvin González-López, Concepción Grajales-Muñiz, Ricardo Grande, Rosa María Gutierrez Rios, Alejandra Hernández-Terán, Alfredo Herrera-Estrella, Carla Ivón Herrera-Najera, Pavel Isa, Daniel Lira Morales, Susana Lopez, Antonio Loza Román, Brenda Irasema Maldonado-Meza, Bernardo Martínez-Miguel, José Arturo Martínez-Orozco, Célida Martínez- Rodríguez, Margarita Matías-Florentino, Fidencio Mejía-Nepomuceno, María Guadalupe de Jesús Mireles-Rivera, Gloria María Molina-Salinas, Hector Montoya-Fuentes, , Mario Mújica-Sánchez, José Esteban Muñoz-Medina, José de Jesús Nuñez-Contreras, Alicia Ocaña-Mondragón, Luis Alberto Ochoa-Carrera, Hector Esteban Paz-Juárez, Marissa Perez-Garcia, Francisco Pulido, Helen Haydee Fernanda Ramírez-Plascencia, Jorge Salas-Hernández, Angel Gustavo Salas-Lais, Alejandro Sanchez-Flores, Clara Esperanza Santacruz-Tinoco, María Guadalupe Santiago-Mauricio, Selene Zárate, Nelly Sélem-Mojica, Blanca Taboada, Gloria Vazquez, Joel Armando Vázquez-Pérez.
EPI_ISL_2490501, EPI_ISL_2490504, EPI_ISL_2490506, EPI_ISL_2490514, EPI_ISL_2490515, EPI_ISL_2490524, EPI_ISL_2490531, EPI_ISL_2490533, EPI_ISL_2490535	Unidad de Investigación Biomédica de Zacatecas (UIBZ)	Centro de Investigación en Enfermedades Infecciosas (CIENI), Instituto Nacional de Enfermedades Respiratorias (INER)	Consorcio Mexicano de Vigilancia Genómica (CoViGen-Mex). Authors (in alphabetical order): Julio Elias Alvarado-Yaah, Carlos F. Arias, Santiago Ávila-Ríos, Eduardo Becerril-Vargas, Víctor Hugo Borja-Aburto, Celia Boukadida, Cristóbal Cháidez-Quiróz, Juan Bautista Chale-Dzul, Ricardo Ciria Merce, Andreu Comas-García, Célida Duque Molina, Julissa Enciso-Ibarra, José Antonio Enciso-Moreno, Gloria Elena Espinosa-Ayala, Fernando Fontove-Herrera, Daniel Fregoso-Rueda, Victor Eduardo Garcia-Arias, Alejandra Garcia-Gasca, Bruno Gomez-Gil, Jean Pierre González, Irvin González-López, Concepción Grajales-Muñiz, Ricardo Grande, Rosa María Gutierrez Rios, Alejandra Hernández-Terán, Alfredo Herrera-Estrella, Carla Ivón Herrera-Najera, Pavel Isa, Daniel Lira Morales, Susana Lopez, Antonio Loza Román, Brenda Irasema Maldonado-Meza, Bernardo Martínez-Miguel, José Arturo Martínez-Orozco, Célida Martínez- Rodríguez, Margarita Matías-Florentino, Fidencio Mejía-Nepomuceno, María Guadalupe de Jesús Mireles-Rivera, Gloria María Molina-Salinas, Hector Montoya-Fuentes, , Mario Mújica-Sánchez, José Esteban Muñoz-Medina, José de Jesús Nuñez-Contreras, Alicia Ocaña-Mondragón, Luis Alberto Ochoa-Carrera, Hector Esteban Paz-Juárez, Marissa Perez-Garcia, Francisco Pulido, Helen Haydee Fernanda Ramírez-Plascencia, Jorge Salas-Hernández, Angel Gustavo Salas-Lais, Alejandro Sanchez-Flores, Clara Esperanza Santacruz-Tinoco, María Guadalupe Santiago-Mauricio, Selene Zárate, Nelly Sélem-Mojica, Blanca Taboada, Gloria Vazquez, Joel Armando Vázquez-Pérez.
EPI_ISL_2490567	Laboratorio Central de Epidemiología (LCE)	Centro de Investigación en Enfermedades Infecciosas (CIENI), Instituto Nacional de Enfermedades Respiratorias (INER)	Consorcio Mexicano de Vigilancia Genómica (CoViGen-Mex). Authors (in alphabetical order): Julio Elias Alvarado-Yaah, Carlos F. Arias, Santiago Ávila-Ríos, Eduardo Becerril-Vargas, Víctor Hugo Borja-Aburto, Celia Boukadida, Cristóbal Cháidez-Quiróz, Juan Bautista Chale-Dzul, Ricardo Ciria Merce, Andreu Comas-García, Célida Duque Molina, Julissa Enciso-Ibarra, José Antonio Enciso-Moreno, Gloria Elena Espinosa-Ayala, Fernando Fontove-Herrera, Daniel Fregoso-Rueda, Victor Eduardo Garcia-Arias, Alejandra Garcia-Gasca, Bruno Gomez-Gil, Jean Pierre González, Irvin González-López, Concepción Grajales-Muñiz, Ricardo Grande, Rosa María Gutierrez Rios, Alejandra Hernández-Terán, Alfredo Herrera-Estrella, Carla Ivón Herrera-Najera, Pavel Isa, Daniel Lira Morales, Susana Lopez, Antonio Loza Román, Brenda Irasema Maldonado-Meza, Bernardo Martínez-Miguel, José Arturo Martínez-Orozco, Célida Martínez- Rodríguez, Margarita Matías-Florentino, Fidencio Mejía-Nepomuceno, María Guadalupe de Jesús Mireles-Rivera, Gloria María Molina-Salinas, Hector Montoya-Fuentes, , Mario Mújica-Sánchez, José Esteban Muñoz-Medina, José de Jesús Nuñez-Contreras, Alicia Ocaña-Mondragón, Luis Alberto Ochoa-Carrera, Hector Esteban Paz-Juárez, Marissa Perez-Garcia, Francisco Pulido, Helen Haydee Fernanda Ramírez-Plascencia, Jorge Salas-Hernández, Angel Gustavo Salas-Lais, Alejandro Sanchez-Flores, Clara Esperanza Santacruz-Tinoco, María Guadalupe Santiago-Mauricio, Selene Zárate, Nelly Sélem-Mojica, Blanca Taboada, Gloria Vazquez, Joel Armando Vázquez-Pérez.

EPI_ISL_2494634	UCSC Genomics Institute	UCSC Genomics Institute	Mark Akeson, Ikenna Anigbogu, Eric Beraut, Isabel Bjork, Molly Cassatt-Johnstone, Terren Chang, Russell Corbett-Detig, Namrita Dhillon, Maximilian Haeussler, David Haussler, Angie Hinrichs, Miten Jain, Joshua Kapp, A. Marm Kilpatrick, Jakob McBroome, Hugh Olsen, Jeremy Sanford, Beth Shapiro, Michael Stone, Bryan Thornlow, Yatish Turakhia, Ciara Wanket
EPI_ISL_2496915, EPI_ISL_2496963, EPI_ISL_2496970, EPI_ISL_2496974	CA DPH Viral and Rickettsial Disease Laboratory	Chan-Zuckerberg Biohub	CZB Cliahub Consortium
EPI_ISL_2496976	Santa Clara County Public Health Laboratory	Chan-Zuckerberg Biohub	CZB Cliahub Consortium
EPI_ISL_2496984, EPI_ISL_2496985, EPI_ISL_2496991, EPI_ISL_2496993, EPI_ISL_2496994, EPI_ISL_2496997	CA DPH Viral and Rickettsial Disease Laboratory	Chan-Zuckerberg Biohub	CZB Cliahub Consortium
EPI_ISL_2497254, EPI_ISL_2497306, EPI_ISL_2497326, EPI_ISL_2497335, EPI_ISL_2497360, EPI_ISL_2497369, EPI_ISL_2497373, EPI_ISL_2497404, EPI_ISL_2497411	UW Virology Lab	UW Virology Lab	Pavitra Roychoudhury, Hong Xie, Lasata Shrestha, Tien V. Nguyen, Shah Mohamed Bakhash, Michelle Lin, Noah R. Baker, Ricardo Perez, Sean Ellis, Nathan Breit, Robert J. Livingston, Meei-Li Huang, Keith R Jerome, Patrick Mathias, Alexander Greninger
EPI_ISL_2497697	Humboldt County Public Health Laboratory	Chan-Zuckerberg Biohub	CZB Cliahub Consortium
EPI_ISL_2497710, EPI_ISL_2497768	CA DPH Viral and Rickettsial Disease Laboratory	Chan-Zuckerberg Biohub	CZB Cliahub Consortium
EPI_ISL_2497931	UW Virology Lab	UW Virology Lab	Pavitra Roychoudhury, Hong Xie, Lasata Shrestha, Tien V. Nguyen, Shah Mohamed Bakhash, Michelle Lin, Noah R. Baker, Ricardo Perez, Sean Ellis, Nathan Breit, Robert J. Livingston, Meei-Li Huang, Keith R Jerome, Patrick Mathias, Alexander Greninger
EPI_ISL_2499966	LESP Sinaloa	Instituto de Diagnostico y Referencia Epidemiologicos (INDRE)	Claudia Wong-Arambula, Abril Rodriguez-Maldonado, Vanessa Rivero-Arredondo, Ariadna Medina-Benitez, Joaquin Quiroz-Mercado, Sergio Rangel-Guerrero, Natividad Cruz-Ortiz, Tatiana Nunez-Garcia, Gisela Barrera-Badillo, Lucia Hernandez-Rivas, Irma Lopez-Martinez, Ernesto Ramirez-Gonzalez.
EPI_ISL_2499967	LESP Michoacan	Instituto de Diagnostico y Referencia Epidemiologicos (INDRE)	Claudia Wong-Arambula, Abril Rodriguez-Maldonado, Vanessa Rivero-Arredondo, Ariadna Medina-Benitez, Joaquin Quiroz-Mercado, Sergio Rangel-Guerrero, Natividad Cruz-Ortiz, Tatiana Nunez-Garcia, Gisela Barrera-Badillo, Lucia Hernandez-Rivas, Irma Lopez-Martinez, Ernesto Ramirez-Gonzalez.
EPI_ISL_2500078, EPI_ISL_2500202	Humboldt County Public Health Laboratory	Chan-Zuckerberg Biohub	CZB Cliahub Consortium
EPI_ISL_2501000	Gundersen Clinical Microbiology Laboratory	Kabara Cancer Research Institute	Craig S. Richmond, Paraic A. Kenny
EPI_ISL_2501523, EPI_ISL_2501524	UW Virology Lab	UW Virology Lab	Pavitra Roychoudhury, Hong Xie, Lasata Shrestha, Tien V. Nguyen, Shah Mohamed Bakhash, Michelle Lin, Noah R. Baker, Ricardo Perez, Sean Ellis, Nathan Breit, Robert J. Livingston, Meei-Li Huang, Keith R Jerome, Patrick Mathias, Alexander Greninger
EPI_ISL_2502729	HOSPITAL LA ANEXION	Inciensa, Instituto Costarricense de InvestigaciOn y Enseñanza en NutriciOn y Salud	Francisco Duarte, Hebleen Porras, Claudio Soto-Garita, Estela Cordero, Adriana Godínez, Melany CalderOn, Jose Luis Vargas, Mariela Gutierrez, Joselyn Prado & Ivanna Krize-Morún
EPI_ISL_2504066	A. Krumbholz, Labor Dr. Krause und Kollegen MVZ GmbH, Kiel	Charite Universitatsmedizin Berlin, Institut fur Virologie/Labor Berlin	Victor M Corman, Andi Krumbholz, Joern Beheim-Schwarzbach, Barbara Müehlemann, Julia Schneider, Talitha Veith, Tobias Bleicker, Julia Tesch, Peter Menzel, Christine Stephan, Rolf Schwarzer, Terry Jones, Christian Drosten
EPI_ISL_2508590	Genetica Molecular and Subdepartamento de Virologia ISP Chile	Instituto de Salud Publica de Chile	Karen Orostica, Constanza Campano, Barbara Parra, Loredana Arata, Gisselle Barra, Patricia Bustos, Rodrigo Fasce, Javier Tognarelli, Andres Castillo, Soledad Ulloa, Jorge Fernandez
EPI_ISL_2525466, EPI_ISL_2525484, EPI_ISL_2525533, EPI_ISL_2525654, EPI_ISL_2525752, EPI_ISL_2527584	Fulgent Genetics	Centers for Disease Control and Prevention Division of Viral Diseases, Pathogen Discovery	Dakota Howard, Dhwani Batra, Peter W. Cook, Kara Moser, Adrian Paskey, Jason Caravas, Benjamin Rambo-Martin, Shatavia Morrison, Christopher Gulvick, Scott Sammons, Yvette Unoarumhi, Darlene Wagner, Matthew Schmerer, Harry Gao, Mickey Li, John Gao, Joseph Fierro, Benafsh Sapra, Becky Tsai, Yan Meng, Doreen Ng, James Xie, Clinton R. Paden, Duncan MacCannell
EPI_ISL_2528580, EPI_ISL_2528933	Aegis Sciences Corporation	Centers for Disease Control and Prevention Division of Viral Diseases, Pathogen Discovery	Dakota Howard, Dhwani Batra, Peter W. Cook, Kara Moser, Adrian Paskey, Jason Caravas, Benjamin Rambo-Martin, Shatavia Morrison, Christopher Gulvick, Scott Sammons, Yvette Unoarumhi, Darlene Wagner, Matthew Schmerer, Cyndi Clark, Patrick Campbell, Rob Case, Vikramsinha Ghorpade, Holly Houdeshell, Ola Kvalvaag, Dillon Nall, Ethan Sanders, Alec Vest, Shaun Westlund, Matthew Hardison, Clinton R. Paden, Duncan MacCannell
EPI_ISL_2529223	Quest Diagnostics Incorporated	Centers for Disease Control and Prevention Division of Viral Diseases, Pathogen Discovery	Dakota Howard, Dhwani Batra, Peter W. Cook, Kara Moser, Adrian Paskey, Jason Caravas, Benjamin Rambo-Martin, Shatavia Morrison, Christopher Gulvick, Scott Sammons, Yvette Unoarumhi, Darlene Wagner, Matthew Schmerer, S. H. Rosenthal, A. Gerasimova, R. M. Kagan, B. Anderson, M. Hua, Y. Liu, L.E. Bernstein, K.E. Livingston, A. Perez, I. A. Shlyakhter, R. V. Rolando, R. Owen, P. Tanpaiboon, F. Lacbawan, Clinton R. Paden, Duncan MacCannell
EPI_ISL_2533921	Hospital Sharp	Microbial Genomics Laboratory	Consorcio Mexicano de Vigilancia Genómica (CoViGen-Mex). Authors (in alphabetical order): Julio Elias Alvarado-Yaah, Carlos F. Arias, Santiago Ávila-Ríos, Eduardo Becerril-Vargas, Víctor Hugo Borja-Aburto, Celia Boukadida, Cristóbal Cháidez-Quiróz, Juan Bautista Chale-Dzul, Ricardo Ciria Merce, Andreu Comas-García, Célida Duque Molina, Julissa Enciso-Ibarra, José Antonio Enciso-Moreno, Gloria Elena Espinosa-Ayala, Fernando Fontove-Herrera, Daniel Fregoso-Rueda, Victor Eduardo García-Arias, Alejandra García-Gasca, Bruno Gómez-Gil, Jean Pierre González, Irvin González-López, Concepción Grajales-Muñiz, Ricardo Grande, Rosa María Gutiérrez Rios, Jesús Hernández, Alejandra Hernández-Terán, Alfredo Herrera-Estrella, Carla Ivón Herrera-Najera, Pavel Isa, Verónica Mata-Haro, Daniel Lira Morales, Susana Lopez, Antonio Loza Román, Brenda Irasema Maldonado-Meza, Bernardo Martínez-Miguel, José Arturo Martínez-Orozco, Célida Martínez- Rodríguez, Margarita Matías-Florentino, Fidencio Mejía-Nepomuceno, María Guadalupe de Jesús Mireles-Rivera, Gloria María Molina-Salinas, Hector Montoya-Fuentes, , Mario Mújica-Sánchez, José Esteban Muñoz-Medina, José de Jesús Nuñez-Contreras, Alicia Ocaña-Mondragón, Luis Alberto Ochoa-Carrera, Hector Esteban Paz-Juárez, Marissa Perez-Garcia, Francisco Pulido, Helen Haydee Fernanda Ramírez-Plascencia, Jorge Salas-Hernández, Angel Gustavo Salas-Lais, Alejandro Sánchez-Flores, Clara Esperanza Santacruz-Tinoco, María Guadalupe Santiago-Mauricio, Selene Zárate, Nelly Sélem-Mojica, Blanca Taboada, Gloria Vazquez, Joel Armando Vázquez-Pérez.
EPI_ISL_2536580	Northwest Laboratory	Seattle Flu Study	Deborah A. Nickerson, Chris D. Frazar, Jover Lee, Benjamin Pelle, Erica Ryke, Matthew Richardson, Amanda Adler, Elisabeth Brandstetter, Peter D. Han, Kairsten Fay, Misja Ilcisin, Kirsten Lacombe, Thomas R. Sibley, Melissa Truong, Caitlin R. Wolf, Romesh Gautom, Geoff Melly, Brian Hiatt, Philip Dykema, Scott Lindquist, Michael Boeckh, Janet A. Englund, Michael Famulare, Barry R. Lutz, Mark J. Rieder, Lea M. Starita, Matthew Thompson, Helen Y. Chu, Jay Shendure, Trevor Bedford
EPI_ISL_2536939	Centracare Laboratory Services	Minnesota Department of Health, Public Health Laboratory	Alexandra Lorentz, Jacob Garfin, Matt Plumb, and Xiong Wang
EPI_ISL_2543569	Genetica Molecular and Subdepartamento de Virologia ISP Chile	Instituto de Salud Publica de Chile	Karen Orostica, Constanza Campano, Barbara Parra, Loredana Arata, Gisselle Barra, Patricia Bustos, Rodrigo Fasce, Javier Tognarelli, Andres Castillo, Soledad Ulloa, Jorge Fernandez
EPI_ISL_2544528	Basurto University Hospital: Clinical Microbiology Laboratory	Biocruces Bizkaia	Mikel Urrutikoetxea-Gutiérrez, Mª Carmen Nieto Toboso, Estibaliz Ugalde Zarraga, Mikel Gallego Rodrigo, Ana de la Hoz, José Luis Díaz de Tuesta del Arco
EPI_ISL_2544984, EPI_ISL_2545001, EPI_ISL_2545002, EPI_ISL_2545060, EPI_ISL_2545128, EPI_ISL_2545178	UW Virology Lab	UW Virology Lab	Pavitra Roychoudhury, Hong Xie, Lasata Shrestha, Shah Mohamed Bakhash, Tien V. Nguyen, Noah R. Baker, Sean Ellis, Meei-Li Huang, Keith R Jerome, Alexander Greninger
EPI_ISL_2545700, EPI_ISL_2545701	LESP Queretaro	Instituto de Diagnostico y Referencia Epidemiologicos (INDRE)	Claudia Wong-Arambula, Abril Rodriguez-Maldonado, Vanessa Rivero-Arredondo, Ariadna Medina-Benitez, Joaquin Quiroz-Mercado, Sergio Rangel-Guerrero, Natividad Cruz-Ortiz, Tatiana Nunez-Garcia, Gisela Barrera-Badillo, Lucia Hernandez-Rivas, Irma Lopez-Martinez, Ernesto Ramirez-Gonzalez.
EPI_ISL_2545702	LESP Michoacan	Instituto de Diagnostico y Referencia Epidemiologicos (INDRE)	Claudia Wong-Arambula, Abril Rodriguez-Maldonado, Vanessa Rivero-Arredondo, Ariadna Medina-Benitez, Joaquin Quiroz-Mercado, Sergio Rangel-Guerrero, Natividad Cruz-Ortiz, Tatiana Nunez-Garcia, Gisela Barrera-Badillo, Lucia Hernandez-Rivas, Irma Lopez-Martinez, Ernesto Ramirez-Gonzalez.

EPI_ISL_2545703, EPI_ISL_2545704	LESP Sinaloa	Instituto de Diagnostico y Referencia Epidemiologicos (INDRE)	Claudia Wong-Arambula, Abril Rodriguez-Maldonado, Vanessa Rivero-Arredondo, Ariadna Medina-Benitez, Joaquin Quiroz-Mercado, Sergio Rangel-Guerrero, Natividad Cruz-Ortiz, Tatiana Nunez-Garcia, Gisela Barrera-Badillo, Lucia Hernandez-Rivas, Irma Lopez-Martinez, Ernesto Ramirez-Gonzalez.
EPI_ISL_2549333, EPI_ISL_2549339, EPI_ISL_2549415, EPI_ISL_2549456, EPI_ISL_2549508, EPI_ISL_2549547, EPI_ISL_2549550, EPI_ISL_2549554	UW Virology Lab	UW Virology Lab	Pavitra Roychoudhury, Hong Xie, Lasata Shrestha, Tien V. Nguyen, Shah Mohamed Bakhash, Michelle Lin, Noah R. Baker, Ricardo Perez, Sean Ellis, Nathan Breit, Robert J. Livingston, Meei-Li Huang, Keith R Jerome, Patrick Mathias, Alexander Greninger
EPI_ISL_2550795	Maryland Public Health Laboratory (MD PHL)	Maryland Public Health Laboratory (MD PHL)	Maryland Department of Health Laboratories Administration
EPI_ISL_2558098, EPI_ISL_2558191, EPI_ISL_2558212, EPI_ISL_2558257	Wisconsin State Laboratory of Hygiene Communicable Disease Division	Wisconsin State Laboratory of Hygiene Communicable Disease Division	Abigail C. Shockey, Alicia J. Mooney, Erika M. Hanson, Tonya Danz, Richard Griesser, Sara Wagner, Kelsey R. Florek
EPI_ISL_2566354, EPI_ISL_2566358	Operation Infectious Diseases, Naval Health Research Center	Operation Infectious Diseases, Naval Health Research Center	Pan,R.W., Underwood,R.S., Balansay-Ames,M.S., Myers,C.A., Fierro,M., Kriner,P., Iniguez-Stevens,E., Moser,K., Phippard,A.E.
EPI_ISL_2567110	Florida Bureau of Public Health Laboratories	Florida Bureau of Public Health Laboratories	Sarah Schmedes, Jason Blanton
EPI_ISL_2587237, EPI_ISL_2587416, EPI_ISL_2587751	SK-Roy Romanow Provincial Laboratory	National Microbiology Laboratory (NML)	Anna Majer, Shari Tyson, Grace Seo, Philip Mabon, Elsie Grudeski, Rhiannon Huzarewich, Russell Mandes, Anneliese Landgraff, Jennifer Tanner, Natalie Knox, Morag Graham, Gary Van Domselaar, Ryan McDonald, Amanda Lang, Rachel DePaulo, Jessica Minion, Nathalie Bastien, Yan Li, Timothy Booth, Darian Hole, Madison Chapel, Kirsten Biggar, CanCOGeN's metadata curation team, Public Health Agency of Canada CanCOGeN team
EPI_ISL_2598722	Fulgent Genetics	Centers for Disease Control and Prevention Division of Viral Diseases, Pathogen Discovery	Dakota Howard, Dhwani Batra, Peter W. Cook, Kara Moser, Adrian Paskey, Jason Caravas, Benjamin Rambo-Martin, Shatavia Morrison, Christopher Gulvick, Scott Sammons, Yvette Unoarumhi, Darlene Wagner, Matthew Schmerer, Harry Gao, Mickey Li, John Gao, Joseph Fierro, Benafsh Sapra, Becky Tsai, Yan Meng, Doreen Ng, James Xie, Clinton R. Paden, Duncan MacCannell
EPI_ISL_2599608	Helix/Illumina	Centers for Disease Control and Prevention Division of Viral Diseases, Pathogen Discovery	Dakota Howard, Dhwani Batra, Peter W. Cook, Kara Moser, Adrian Paskey, Jason Caravas, Benjamin Rambo-Martin, Shatavia Morrison, Christopher Gulvick, Scott Sammons, Yvette Unoarumhi, Darlene Wagner, Matthew Schmerer, Eileen de Feo, Jan Antico, Christine Tran, Matthew Tolentino, Shannon Wickline, Kim Gietzen, Brad Sickler, Jingtao Liu, Eric Allen, Phil Febbo, Nicole L. Washington, Simon White, Geraint Levan, Kelly Schiabor Barrett, Elizabeth Cirulli, Alexandre Bolze, Ary Ascencio, Charlotte Rivera-Garcia, Ryan Cho, Jason Nguyen, Sherry Wang, Jimmy Ramirez, Tyler Cassens, Efren Sandoval, Magnus Isaksson, William Lee, David Becker, Marc Laurent, James Lu, Clinton R. Paden, Duncan MacCannell
EPI_ISL_2602373	Gravity Diagnostics, LLC	Gravity Diagnostics, LLC	Gravity Diagnostics
EPI_ISL_2611257, EPI_ISL_2611278, EPI_ISL_2611348	Laboratory Corporation of America	Centers for Disease Control and Prevention Division of Viral Diseases, Pathogen Discovery	Dakota Howard, Dhwani Batra, Peter W. Cook, Kara Moser, Adrian Paskey, Jason Caravas, Benjamin Rambo-Martin, Shatavia Morrison, Christopher Gulvick, Scott Sammons, Yvette Unoarumhi, Darlene Wagner, Matthew Schmerer, Minoo Agarwal, Eyad Almasri, Debbie Boles, Ayla Burns, Nuthawin Charoensri, Oren Cohen, Susan Countryman, Mary Ann Cristobal, Bobbi Croy, Suzanne Dale, Hrushikesh Deshmukh, Amanda Douglas, Vincent Drouillon, Marcia Eisenberg, Howard Engler, Rama Ghatti, Prashant Gupta, Susan Hicks, Jake Humphrey, Lax Iyer, Lisa Pfefferle, Manoj Jain, Matthew Robinson, Mohan Kolli, Brian Krueger, Tim Kuphal, Stanley Letovsky, Michael Levandoski, Craig Lukasik, Jonathan Meltzer, Brian Norvell, Mindy Nye, Scott Parker, Christos Petropoulos, John Pruitt, Steven Ragan, Scott Ryan, Mike Sapeta, Jana Schroth, Suresh Babu Selvaraju, Goran Stevovic, Amanda Suchanek, Andrea Throop, Lyndon Tilson, Thomas Urban, Joe Voshell, Kimberly Wagner, Jonathan Williams, Mary Williamson, Qian Zeng, Tricia Zwiefelhofer, Clinton R. Paden, Duncan MacCannell
EPI_ISL_2612520	Centracare Laboratory Services	Minnesota Department of Health, Public Health Laboratory	Alexandra Lorentz, Jacob Garfin, Matt Plumb, and Xiong Wang
EPI_ISL_2612548	Oregon State Public Health Laboratory	Oregon State Public Health Laboratory	Rafia Razzaque, Eugene Yeboah, Vanda Makris, Laura Tsaknaridis, John Fontana and Shane Sevey
EPI_ISL_2613845	Gravity Diagnostics, LLC	Gravity Diagnostics, LLC	Gravity Diagnostics
EPI_ISL_2617995, EPI_ISL_2618032, EPI_ISL_2618034, EPI_ISL_2618082	UW Virology Lab	UW Virology Lab	Pavitra Roychoudhury, Hong Xie, Lasata Shrestha, Tien V. Nguyen, Shah Mohamed Bakhash, Michelle Lin, Noah R. Baker, Ricardo Perez, Sean Ellis, Nathan Breit, Robert J. Livingston, Meei-Li Huang, Keith R Jerome, Patrick Mathias, Alexander Greninger
EPI_ISL_2618141, EPI_ISL_2618150, EPI_ISL_2618183, EPI_ISL_2618229, EPI_ISL_2618235, EPI_ISL_2618344, EPI_ISL_2618348, EPI_ISL_2618670, EPI_ISL_2618673, EPI_ISL_2618683, EPI_ISL_2618703, EPI_ISL_2618707, EPI_ISL_2618725			
see above	UW Virology Lab	UW Virology Lab	Pavitra Roychoudhury, Hong Xie, Lasata Shrestha, Shah Mohamed Bakhash, Tien V. Nguyen, Noah R. Baker, Sean Ellis, Meei-Li Huang, Keith R Jerome, Alexander Greninger
EPI_ISL_2627045, EPI_ISL_2627046	URMC LABS	Wadsworth Center, New York State Department of Health	Kirsten St. George, Daryl M. Lamson, Alexis Russell, Matthew Shudt, Melissa A Leisner, Jonathan Plitnick, Catharine Prussing, Navjot Singh, John Kelly, Erasmus Schneider, Erica Lasek-Nesselquist
EPI_ISL_2628812, EPI_ISL_2628819	San Diego County Public Health Laboratory	Andersen lab at Scripps Research	SEARCH Alliance San Diego with Ashleigh Murphy, Jovan Shephard, Brett Austin
EPI_ISL_2632714	Limbach - MVZ Humangenetik Ulm	Robert Koch Institute	unknown
EPI_ISL_2645346	MI - Michigan Department of Health and Human Services - Bureau of Laboratories	Centers for Disease Control and Prevention Division of Viral Diseases, Pathogen Discovery	Mili Sheth, Sarah Nobles, Jasmine Padilla, Mark Burroughs, Shoshona Le, Katie Dillon, Peter Cook, Clinton R. Paden, Dhwani Batra, Krista Queen, Kristen Knipe, Dakota Howard, Yvette Unoarumhi, Darlene Wagner, Matthew Schmerer, Ben L. Rambo-Martin, Kristine Lacek, Sam Shepard, Alison Laufer Halpin, Dave Wentworth, Vivien Dugan, Suxiang Tong, Justin Lee
EPI_ISL_2646370, EPI_ISL_2646424, EPI_ISL_2646438, EPI_ISL_2646471, EPI_ISL_2646522, EPI_ISL_2646586, EPI_ISL_2646594, EPI_ISL_2646649, EPI_ISL_2646693, EPI_ISL_2646701, EPI_ISL_2647239, EPI_ISL_2647259, EPI_ISL_2647395, EPI_ISL_2647496, EPI_ISL_2647615, EPI_ISL_2647685, EPI_ISL_2647797, EPI_ISL_2647875			
see above	Mako Medical	Centers for Disease Control and Prevention Division of Viral Diseases, Pathogen Discovery	Dakota Howard, Dhwani Batra, Peter W. Cook, Kara Moser, Adrian Paskey, Jason Caravas, Benjamin Rambo-Martin, Shatavia Morrison, Christopher Gulvick, Scott Sammons, Yvette Unoarumhi, Darlene Wagner, Matthew Schmerer,Matthew Tugwell, Lauren Moon, Clinton R. Paden, Duncan MacCannell
EPI_ISL_2650519	University of Oregon COVID-19 MAP Laboratory	University of Oregon Genomics and Cell Characterization Core Facility (GC3F)	Douglas Turnbull, Ariana White, Jeff Bishop, Jason Carriere, Jason Sydes, Peter Batzel
EPI_ISL_2651069, EPI_ISL_2651072, EPI_ISL_2651078	Washington State Department of Health Public Health Laboratories	Washington State Department of Health Public Health Laboratories	Drew MacKellar, Philip Dykema, Denny Russell, Joenice Gonzalez, Hannah Gray, Geoff Melly, Vanessa De Los Santos, Darren Lucas, JohnAric Peterson, Avi Singh, Rebecca Cao
EPI_ISL_2652303, EPI_ISL_2652377, EPI_ISL_2652384	Quest Diagnostics Incorporated	Centers for Disease Control and Prevention Division of Viral Diseases, Pathogen Discovery	Dakota Howard, Dhwani Batra, Peter W. Cook, Kara Moser, Adrian Paskey, Jason Caravas, Benjamin Rambo-Martin, Shatavia Morrison, Christopher Gulvick, Scott Sammons, Yvette Unoarumhi, Darlene Wagner, Matthew Schmerer, S. H. Rosenthal, A. Gerasimova, R. M. Kagan, B. Anderson, M. Hua, Y. Liu, L.E. Bernstein, K.E. Livingston, A. Perez, I. A. Shlyakhter, R. V. Rolando, R. Owen, P. Tanpaiboon, F. Lacbawan, Clinton R. Paden, Duncan MacCannell
EPI_ISL_2652628, EPI_ISL_2652681	Infinity Biologix	Centers for Disease Control and Prevention Division of Viral Diseases, Pathogen Discovery	Dakota Howard, Dhwani Batra, Peter W. Cook, Kara Moser, Adrian Paskey, Jason Caravas, Benjamin Rambo-Martin, Shatavia Morrison, Christopher Gulvick, Scott Sammons, Yvette Unoarumhi, Darlene Wagner, Matthew Schmerer, Christian Bixby, Yihe Wang, Jonathan Schultz, Chirayu Goswami, Russ Hager, Robin Grimwood, Clinton R. Paden, Duncan MacCannell
EPI_ISL_2656549, EPI_ISL_2656553, EPI_ISL_2656664, EPI_ISL_2656820	BCCDC Public Health Laboratory	BCCDC Public Health Laboratory	Prystajecky Natalie, Linda Hoang, Dan Fornika, John Tyson, Shannon Russell, Kim Macdonald, Kimia Kamelian, Ana Pacagnella, Corrinne Ng, Loretta Janz, Robert Azana, Mel Krajden
EPI_ISL_2658683, EPI_ISL_2658685	CA DPH Viral and Rickettsial Disease Laboratory	Chan-Zuckerberg Biohub	CZB Cliahub Consortium
EPI_ISL_2658951, EPI_ISL_2658952	LESP San Luis Potosi	Instituto de Diagnostico y Referencia Epidemiologicos (INDRE)	Claudia Wong-Arambula, Abril Rodriguez-Maldonado, Vanessa Rivero-Arredondo, Ariadna Medina-Benitez, Joaquin Quiroz-Mercado, Sergio Rangel-Guerrero, Natividad Cruz-Ortiz, Tatiana Nunez-Garcia, Gisela Barrera-Badillo, Lucia Hernandez-Rivas, Irma Lopez-Martinez, Ernesto Ramirez-Gonzalez.
EPI_ISL_2658953	LESP Nuevo Leon	Instituto de Diagnostico y Referencia Epidemiologicos (INDRE)	Claudia Wong-Arambula, Abril Rodriguez-Maldonado, Vanessa Rivero-Arredondo, Ariadna Medina-Benitez, Joaquin Quiroz-Mercado, Sergio Rangel-Guerrero, Natividad Cruz-Ortiz, Tatiana Nunez-Garcia, Gisela Barrera-Badillo, Lucia Hernandez-Rivas, Irma Lopez-Martinez, Ernesto Ramirez-Gonzalez.
EPI_ISL_2659153, EPI_ISL_2663428,	Genetica Molecular and Subdepartamento de Virologia ISP	Instituto de Salud Publica de Chile	Karen Orostica, Constanza Campano, Barbara Parra, Loredana Arata, Gisselle Barra, Patricia Bustos, Rodrigo Fasce, Javier Tognarelli, Andres Castillo,

EPI_ISL_2663589, EPI_ISL_2663590, EPI_ISL_2663591	Chile		Soledad Ulloa, Jorge Fernandez
EPI_ISL_2663663, EPI_ISL_2663664, EPI_ISL_2663681, EPI_ISL_2663689, EPI_ISL_2663709, EPI_ISL_2663731, EPI_ISL_2663737, EPI_ISL_2663778	OHSU Lab Services Molecular Microbiology Lab	Oregon SARS-CoV-2 Genome Sequencing Center	Brendan L. O'Connell, Sonia Acharya, Cierra LaBlanc, Ruth V. Nichols, Alec J. Hirsch, Donna Hansel, Guang Fan, Xuan Qin, Daniel N. Streblow, William B. Messer, Andrew C. Adey, Benjamin N. Bimber, Brian J. O'Roak
EPI_ISL_2671571	Centro de Investigacion Biomedica de Occidente (CIBO)	Unidad de Genomica Avanzada	Consorcio Mexicano de Vigilancia Genomica (CoViGen-Mex). Authors (in alphabetical order): Julio Elias Alvarado-Yaah, Carlos F. Arias, Santiago avila-Rios, Eduardo Becerril-Vargas, Victor Hugo Borja-Aburto, Celia Boukadida, Cristobal Chaidez-Quiroz, Juan Bautista Chale-Dzul, Ricardo Ciria Merce, Andreu Comas-Garcia, Celida Duque Molina, Julissa Enciso-Ibarra, Jose Antonio Enciso-Moreno, Gloria Elena Espinosa-Ayala, Fernando Fontove-Herrera, Daniel Fregoso-Rueda, Victor Eduardo Garcia-Arias, Alejandra Garcia-Gasca, Bruno Gomez-Gil, Jean Pierre Gonzalez, Irvin Gonzalez-Lopez, Concepcion Grajales-Muñiz, Ricardo Grande, Rosa Maria Gutierrez Rios, Jesus Hernandez, Alejandra Hernandez-Teran, Alfredo Herrera-Estrella, Carla Ivon Herrera-Najera, Pavel Isa, Veronica Mata-Haro, Daniel Lira Morales, Susana Lopez, Antonio Loza Roman, Brenda Irasema Maldonado-Meza, Bernardo Martinez-Miguel, Jose Arturo Martinez-Orozco, Celida Martinez- Rodriguez, Margarita Matias-Florentino, Fidencio Mejia-Nepomuceno, Maria Guadalupe de Jesus Mireles-Rivera, Gloria Maria Molina-Salinas, Hector Montoya-Fuentes, , Mario Mujica-Sanchez, Jose Esteban Muñoz-Medina, Jose de Jesus Nuñez-Contreras, Alicia Ocaña-Mondragon, Luis Alberto Ochoa-Carrera, Hector Esteban Paz-Juarez, Marissa Perez-Garcia, Francisco Pulido, Helen Haydee Fernanda Ramirez-Plascencia, Jorge Salas-Hernandez, Angel Gustavo Salas-Lais, Alejandro Sanchez-Flores, Clara Esperanza Santacruz-Tinoco, Maria Guadalupe Santiago-Mauricio, Selene Zarate, Nelly Selem-Mojica, Blanca Taboada, Gloria Vazquez, Joel Armando Vazquez-Perez.
EPI_ISL_2671620, EPI_ISL_2671630	Centro de Investigacion Biomedica del Noreste (CIBIN)	Unidad de Genomica Avanzada	Consorcio Mexicano de Vigilancia Genomica (CoViGen-Mex). Authors (in alphabetical order): Julio Elias Alvarado-Yaah, Carlos F. Arias, Santiago avila-Rios, Eduardo Becerril-Vargas, Victor Hugo Borja-Aburto, Celia Boukadida, Cristobal Chaidez-Quiroz, Juan Bautista Chale-Dzul, Ricardo Ciria Merce, Andreu Comas-Garcia, Celida Duque Molina, Julissa Enciso-Ibarra, Jose Antonio Enciso-Moreno, Gloria Elena Espinosa-Ayala, Fernando Fontove-Herrera, Daniel Fregoso-Rueda, Victor Eduardo Garcia-Arias, Alejandra Garcia-Gasca, Bruno Gomez-Gil, Jean Pierre Gonzalez, Irvin Gonzalez-Lopez, Concepcion Grajales-Muñiz, Ricardo Grande, Rosa Maria Gutierrez Rios, Jesus Hernandez, Alejandra Hernandez-Teran, Alfredo Herrera-Estrella, Carla Ivon Herrera-Najera, Pavel Isa, Veronica Mata-Haro, Daniel Lira Morales, Susana Lopez, Antonio Loza Roman, Brenda Irasema Maldonado-Meza, Bernardo Martinez-Miguel, Jose Arturo Martinez-Orozco, Celida Martinez- Rodriguez, Margarita Matias-Florentino, Fidencio Mejia-Nepomuceno, Maria Guadalupe de Jesus Mireles-Rivera, Gloria Maria Molina-Salinas, Hector Montoya-Fuentes, , Mario Mujica-Sanchez, Jose Esteban Muñoz-Medina, Jose de Jesus Nuñez-Contreras, Alicia Ocaña-Mondragon, Luis Alberto Ochoa-Carrera, Hector Esteban Paz-Juarez, Marissa Perez-Garcia, Francisco Pulido, Helen Haydee Fernanda Ramirez-Plascencia, Jorge Salas-Hernandez, Angel Gustavo Salas-Lais, Alejandro Sanchez-Flores, Clara Esperanza Santacruz-Tinoco, Maria Guadalupe Santiago-Mauricio, Selene Zarate, Nelly Selem-Mojica, Blanca Taboada, Gloria Vazquez, Joel Armando Vazquez-Perez.
EPI_ISL_2671701	Unidad de Investigacion Biomedica de Zacatecas (UIBZ)	Unidad de Genomica Avanzada	Consorcio Mexicano de Vigilancia Genomica (CoViGen-Mex). Authors (in alphabetical order): Julio Elias Alvarado-Yaah, Carlos F. Arias, Santiago avila-Rios, Eduardo Becerril-Vargas, Victor Hugo Borja-Aburto, Celia Boukadida, Cristobal Chaidez-Quiroz, Juan Bautista Chale-Dzul, Ricardo Ciria Merce, Andreu Comas-Garcia, Celida Duque Molina, Julissa Enciso-Ibarra, Jose Antonio Enciso-Moreno, Gloria Elena Espinosa-Ayala, Fernando Fontove-Herrera, Daniel Fregoso-Rueda, Victor Eduardo Garcia-Arias, Alejandra Garcia-Gasca, Bruno Gomez-Gil, Jean Pierre Gonzalez, Irvin Gonzalez-Lopez, Concepcion Grajales-Muñiz, Ricardo Grande, Rosa Maria Gutierrez Rios, Jesus Hernandez, Alejandra Hernandez-Teran, Alfredo Herrera-Estrella, Carla Ivon Herrera-Najera, Pavel Isa, Veronica Mata-Haro, Daniel Lira Morales, Susana Lopez, Antonio Loza Roman, Brenda Irasema Maldonado-Meza, Bernardo Martinez-Miguel, Jose Arturo Martinez-Orozco, Celida Martinez- Rodriguez, Margarita Matias-Florentino, Fidencio Mejia-Nepomuceno, Maria Guadalupe de Jesus Mireles-Rivera, Gloria Maria Molina-Salinas, Hector Montoya-Fuentes, , Mario Mujica-Sanchez, Jose Esteban Muñoz-Medina, Jose de Jesus Nuñez-Contreras, Alicia Ocaña-Mondragon, Luis Alberto Ochoa-Carrera, Hector Esteban Paz-Juarez, Marissa Perez-Garcia, Francisco Pulido, Helen Haydee Fernanda Ramirez-Plascencia, Jorge Salas-Hernandez, Angel Gustavo Salas-Lais, Alejandro Sanchez-Flores, Clara Esperanza Santacruz-Tinoco, Maria Guadalupe Santiago-Mauricio, Selene Zarate, Nelly Selem-Mojica, Blanca Taboada, Gloria Vazquez, Joel Armando Vazquez-Perez.
EPI_ISL_2671747	Unidad de Investigacion Medica de Yucatan (UIMY)	Unidad de Genomica Avanzada	Consorcio Mexicano de Vigilancia Genomica (CoViGen-Mex). Authors (in alphabetical order): Julio Elias Alvarado-Yaah, Carlos F. Arias, Santiago avila-Rios, Eduardo Becerril-Vargas, Victor Hugo Borja-Aburto, Celia Boukadida, Cristobal Chaidez-Quiroz, Juan Bautista Chale-Dzul, Ricardo Ciria Merce, Andreu Comas-Garcia, Celida Duque Molina, Julissa Enciso-Ibarra, Jose Antonio Enciso-Moreno, Gloria Elena Espinosa-Ayala, Fernando Fontove-Herrera, Daniel Fregoso-Rueda, Victor Eduardo Garcia-Arias, Alejandra Garcia-Gasca, Bruno Gomez-Gil, Jean Pierre Gonzalez, Irvin Gonzalez-Lopez, Concepcion Grajales-Muñiz, Ricardo Grande, Rosa Maria Gutierrez Rios, Jesus Hernandez, Alejandra Hernandez-Teran, Alfredo Herrera-Estrella, Carla Ivon Herrera-Najera, Pavel Isa, Veronica Mata-Haro, Daniel Lira Morales, Susana Lopez, Antonio Loza Roman, Brenda Irasema Maldonado-Meza, Bernardo Martinez-Miguel, Jose Arturo Martinez-Orozco, Celida Martinez- Rodriguez, Margarita Matias-Florentino, Fidencio Mejia-Nepomuceno, Maria Guadalupe de Jesus Mireles-Rivera, Gloria Maria Molina-Salinas, Hector Montoya-Fuentes, , Mario Mujica-Sanchez, Jose Esteban Muñoz-Medina, Jose de Jesus Nuñez-Contreras, Alicia Ocaña-Mondragon, Luis Alberto Ochoa-Carrera, Hector Esteban Paz-Juarez, Marissa Perez-Garcia, Francisco Pulido, Helen Haydee Fernanda Ramirez-Plascencia, Jorge Salas-Hernandez, Angel Gustavo Salas-Lais, Alejandro Sanchez-Flores, Clara Esperanza Santacruz-Tinoco, Maria Guadalupe Santiago-Mauricio, Selene Zarate, Nelly Selem-Mojica, Blanca Taboada, Gloria Vazquez, Joel Armando Vazquez-Perez.
EPI_ISL_2678983, EPI_ISL_2679276	Utah Public Health Laboratory	Utah Public Health Laboratory	Erin L. Young, Kelly F. Oakeson, Tara Gallagher
EPI_ISL_2680485	Humboldt County Public Health Laboratory	Humboldt County Public Health Laboratory	Jeremy Corrigan
EPI_ISL_2680922	Laboratorios Delia Barraza	Laboratorio nacional-LANIIA-CIAD	Consorcio Mexicano de Vigilancia Genómica (CoViGen-Mex). Authors (in alphabetical order): Julio Elias Alvarado-Yaah, Carlos F. Arias, Santiago Ávila-Ríos, Eduardo Becerril-Vargas, Víctor Hugo Borja-Aburto, Celia Boukadida, Cristóbal Cháidez-Quiróz, Juan Bautista Chale-Dzul, Ricardo Ciria Merce, Andreu Comas-García, Célida Duque Molina, Julissa Enciso-Ibarra, José Antonio Enciso-Moreno, Gloria Elena Espinosa-Ayala, Fernando Fontove-Herrera, Daniel Fregoso-Rueda, Victor Eduardo García-Arias, Alejandra García-Gasca, Bruno Gómez-Gil, Jean Pierre González, Irvin González-López, Concepción Grajales-Muñiz, Ricardo Grande, Rosa María Gutiérrez Rios, Jesús Hernández, Alejandra Hernández-Terán, Alfredo Herrera-Estrella, Carla Ivón Herrera-Najera, Pavel Isa, Verónica Mata-Haro, Daniel Lira Morales, Susana Lopez, Antonio Loza Román, Brenda Irasema Maldonado-Meza, Bernardo Martínez-Miguel, José Arturo Martínez-Orozco, Célida Martínez- Rodríguez, Margarita Matías-Florentino, Fidencio Mejía-Nepomuceno, María Guadalupe de Jesús Mireles-Rivera, Gloria María Molina-Salinas, Hector Montoya-Fuentes, , Mario Mújica-Sánchez, José Esteban Muñoz-Medina, José de Jesús Nuñez-Contreras, Alicia Ocaña-Mondragón, Luis Alberto Ochoa-Carrera, Hector Esteban Paz-Juárez, Marissa Perez-Garcia, Francisco Pulido, Helen Haydee Fernanda Ramírez-Plascencia, Jorge Salas-Hernández, Angel Gustavo Salas-Lais, Alejandro Sánchez-Flores, Clara Esperanza Santacruz-Tinoco, María Guadalupe Santiago-Mauricio, Selene Zárate, Nelly Sélem-Mojica, Blanca Taboada, Gloria Vazquez, Joel Armando Vázquez-Pérez. Laboratorios Delia Barraza: María Delia Barraza Sámano, Claudia Soto Félix, Melissa García Angulo.
EPI_ISL_2681236	Unidad de Investigación Médica de Yucatán (UIMY)	Instituto de Biotecnología de la UNAM	Consorcio Mexicano de Vigilancia Genómica (CoViGen-Mex). Authors (in alphabetical order): Julio Elias Alvarado-Yaah, Carlos F. Arias, Santiago Ávila-Ríos, Eduardo Becerril-Vargas, Víctor Hugo Borja-Aburto, Celia Boukadida, Cristóbal Cháidez-Quiróz, Juan Bautista Chale-Dzul, Ricardo Ciria Merce, Andreu Comas-García, Célida Duque Molina, Julissa Enciso-Ibarra, José Antonio Enciso-Moreno, Gloria Elena Espinosa-Ayala, Fernando Fontove-Herrera, Daniel Fregoso-Rueda, Victor Eduardo García-Arias, Alejandra García-Gasca, Bruno Gómez-Gil, Jean Pierre González, Irvin González-López, Concepción Grajales-Muñiz, Ricardo Grande, Rosa María Gutiérrez Rios, Jesús Hernández, Alejandra Hernández-Terán, Alfredo Herrera-Estrella, Carla Ivón Herrera-Najera, Pavel Isa, Verónica Mata-Haro, Daniel Lira Morales, Susana Lopez, Antonio Loza Román, Brenda Irasema Maldonado-Meza, Bernardo Martínez-Miguel, José Arturo Martínez-Orozco, Célida Martínez- Rodríguez, Margarita Matías-Florentino, Fidencio Mejía-Nepomuceno, María Guadalupe de Jesús Mireles-Rivera, Gloria María Molina-Salinas, Hector Montoya-Fuentes, , Mario Mújica-Sánchez, José Esteban Muñoz-Medina, José de Jesús Nuñez-Contreras, Alicia Ocaña-Mondragón, Luis Alberto Ochoa-Carrera, Hector Esteban Paz-Juárez, Marissa Perez-Garcia, Francisco Pulido, Helen Haydee Fernanda Ramírez-Plascencia, Jorge Salas-Hernández, Angel Gustavo Salas-Lais, Alejandro Sánchez-Flores, Clara Esperanza Santacruz-Tinoco, María Guadalupe Santiago-Mauricio, Selene Zárate, Nelly Sélem-Mojica, Blanca Taboada, Gloria Vazquez, Joel Armando Vázquez-Pérez.
EPI_ISL_2681252	Laboratorio Central de Epidemiología (LCE)	Instituto de Biotecnología de la UNAM	Consorcio Mexicano de Vigilancia Genómica (CoViGen-Mex). Authors (in alphabetical order): Julio Elias Alvarado-Yaah, Carlos F. Arias, Santiago Ávila-Ríos, Eduardo Becerril-Vargas, Víctor Hugo Borja-Aburto, Celia Boukadida, Cristóbal Cháidez-Quiróz, Juan Bautista Chale-Dzul, Ricardo Ciria

			Merce, Andreu Comas-García, Célida Duque Molina, Julissa Enciso-Ibarra, José Antonio Enciso-Moreno, Gloria Elena Espinosa-Ayala, Fernando Fontove-Herrera, Daniel Fregoso-Rueda, Victor Eduardo García-Arias, Alejandra García-Gasca, Bruno Gómez-Gil, Jean Pierre González, Irvin González-López, Concepción Grajales-Muñiz, Ricardo Grande, Rosa María Gutiérrez Rios, Jesús Hernández, Alejandra Hernández-Terán, Alfredo Herrera-Estrella, Carla Ivón Herrera-Najera, Pavel Isa, Verónica Mata-Haro, Daniel Lira Morales, Susana Lopez, Antonio Loza Román, Brenda Irasema Maldonado-Meza, Bernardo Martínez-Miguel, José Arturo Martínez-Orozco, Célida Martínez- Rodríguez, Margarita Matías-Florentino, Fidencio Mejía-Nepomuceno, María Guadalupe de Jesús Mireles-Rivera, Gloria María Molina-Salinas, Hector Montoya-Fuentes, , Mario Mújica-Sánchez, José Esteban Muñoz-Medina, José de Jesús Nuñez-Contreras, Alicia Ocaña-Mondragón, Luis Alberto Ochoa-Carrera, Hector Esteban Paz-Juárez, Marissa Perez-Garcia, Francisco Pulido, Helen Haydee Fernanda Ramírez-Plascencia, Jorge Salas-Hernández, Angel Gustavo Salas-Lais, Alejandro Sánchez-Flores, Clara Esperanza Santacruz-Tinoco, María Guadalupe Santiago-Mauricio, Selene Zárate, Nelly Sélem-Mojica, Blanca Taboada, Gloria Vazquez, Joel Armando Vázquez-Pérez.
EPI_ISL_2681271	Centro de Investigación Biomédica del Noreste (CIBIN)	Instituto de Biotecnología de la UNAM	Consorcio Mexicano de Vigilancia Genómica (CoViGen-Mex). Authors (in alphabetical order): Julio Elias Alvarado-Yaah, Carlos F. Arias, Santiago Ávila-Ríos, Eduardo Becerril-Vargas, Víctor Hugo Borja-Aburto, Celia Boukadida, Cristóbal Cháidez-Quiróz, Juan Bautista Chale-Dzul, Ricardo Ciria Merce, Andreu Comas-García, Célida Duque Molina, Julissa Enciso-Ibarra, José Antonio Enciso-Moreno, Gloria Elena Espinosa-Ayala, Fernando Fontove-Herrera, Daniel Fregoso-Rueda, Victor Eduardo García-Arias, Alejandra García-Gasca, Bruno Gómez-Gil, Jean Pierre González, Irvin González-López, Concepción Grajales-Muñiz, Ricardo Grande, Rosa María Gutiérrez Rios, Jesús Hernández, Alejandra Hernández-Terán, Alfredo Herrera-Estrella, Carla Ivón Herrera-Najera, Pavel Isa, Verónica Mata-Haro, Daniel Lira Morales, Susana Lopez, Antonio Loza Román, Brenda Irasema Maldonado-Meza, Bernardo Martínez-Miguel, José Arturo Martínez-Orozco, Célida Martínez- Rodríguez, Margarita Matías-Florentino, Fidencio Mejía-Nepomuceno, María Guadalupe de Jesús Mireles-Rivera, Gloria María Molina-Salinas, Hector Montoya-Fuentes, , Mario Mújica-Sánchez, José Esteban Muñoz-Medina, José de Jesús Nuñez-Contreras, Alicia Ocaña-Mondragón, Luis Alberto Ochoa-Carrera, Hector Esteban Paz-Juárez, Marissa Perez-Garcia, Francisco Pulido, Helen Haydee Fernanda Ramírez-Plascencia, Jorge Salas-Hernández, Angel Gustavo Salas-Lais, Alejandro Sánchez-Flores, Clara Esperanza Santacruz-Tinoco, María Guadalupe Santiago-Mauricio, Selene Zárate, Nelly Sélem-Mojica, Blanca Taboada, Gloria Vazquez, Joel Armando Vázquez-Pérez.
EPI_ISL_2686407, EPI_ISL_2686419, EPI_ISL_2686426, EPI_ISL_2686597	Fulgent Genetics	Centers for Disease Control and Prevention Division of Viral Diseases, Pathogen Discovery	Dakota Howard, Dhwani Batra, Peter W. Cook, Kara Moser, Adrian Paskey, Jason Caravas, Benjamin Rambo-Martin, Shatavia Morrison, Christopher Gulvick, Scott Sammons, Yvette Unoarumhi, Darlene Wagner, Matthew Schmerer, Harry Gao, Mickey Li, John Gao, Joseph Fierro, Benafsh Sapra, Becky Tsai, Yan Meng, Doreen Ng, James Xie, Clinton R. Paden, Duncan MacCannell
EPI_ISL_2686938, EPI_ISL_2687004, EPI_ISL_2687041, EPI_ISL_2687042	Aegis Sciences Corporation	Centers for Disease Control and Prevention Division of Viral Diseases, Pathogen Discovery	Dakota Howard, Dhwani Batra, Peter W. Cook, Kara Moser, Adrian Paskey, Jason Caravas, Benjamin Rambo-Martin, Shatavia Morrison, Christopher Gulvick, Scott Sammons, Yvette Unoarumhi, Darlene Wagner, Matthew Schmerer, Cyndi Clark, Patrick Campbell, Rob Case, Vikramsinha Ghorpade, Holly Houdeshell, Ola Kvalvaag, Dillon Nall, Ethan Sanders, Alec Vest, Shaun Westlund, Matthew Hardison, Clinton R. Paden, Duncan MacCannell
EPI_ISL_2689673	Nebraska Public Health Laboratory	NPHL COVID-19 Response Team	NPHL COVID-19 Response Team
EPI_ISL_2689985	OR State PHL-Virology/Immunology Section	Centers for Disease Control and Prevention Division of Viral Diseases, Pathogen Discovery	Mili Sheth, Sarah Nobles, Jasmine Padilla, Mark Burroughs, Shoshona Le, Katie Dillon, Peter Cook, Clinton R. Paden, Dhwani Batra, Krista Queen, Kristen Knipe, Dakota Howard, Yvette Unoarumhi, Darlene Wagner, Matthew Schmerer, Ben L. Rambo-Martin, Kristine Lacek, Sam Shepard, Alison Laufer Halpin, Dave Wentworth, Vivien Dugan, Suxiang Tong, Justin Lee
EPI_ISL_2690075	ID Bureau of Laboratories	Centers for Disease Control and Prevention Division of Viral Diseases, Pathogen Discovery	Mili Sheth, Sarah Nobles, Jasmine Padilla, Mark Burroughs, Shoshona Le, Katie Dillon, Peter Cook, Clinton R. Paden, Dhwani Batra, Krista Queen, Kristen Knipe, Dakota Howard, Yvette Unoarumhi, Darlene Wagner, Matthew Schmerer, Ben L. Rambo-Martin, Kristine Lacek, Sam Shepard, Alison Laufer Halpin, Dave Wentworth, Vivien Dugan, Suxiang Tong, Justin Lee
EPI_ISL_2690111	WA State Department of Health	Centers for Disease Control and Prevention Division of Viral Diseases, Pathogen Discovery	Mili Sheth, Sarah Nobles, Jasmine Padilla, Mark Burroughs, Shoshona Le, Katie Dillon, Peter Cook, Clinton R. Paden, Dhwani Batra, Krista Queen, Kristen Knipe, Dakota Howard, Yvette Unoarumhi, Darlene Wagner, Matthew Schmerer, Ben L. Rambo-Martin, Kristine Lacek, Sam Shepard, Alison Laufer Halpin, Dave Wentworth, Vivien Dugan, Suxiang Tong, Justin Lee
EPI_ISL_2692208, EPI_ISL_2692220, EPI_ISL_2692221, EPI_ISL_2692222, EPI_ISL_2692223, EPI_ISL_2692224, EPI_ISL_2692225	Seattle Flu Study	Seattle Flu Study	Deborah A. Nickerson, Chris D. Frazar, Jover Lee, Benjamin Pelle, Erica Ryke, Matthew Richardson, Amanda Adler, Elisabeth Brandstetter, Peter D. Han, Kairsten Fay, Misja Ilcisin, Kirsten Lacombe, Thomas R. Sibley, Melissa Truong, Caitlin R. Wolf, Karen Cowgill, Stephanie Schrag, Jeff Duchin, Michael Boeckh, Janet A. Englund, Michael Famulare, Barry R. Lutz, Mark J. Rieder, Lea M. Starita, Matthew Thompson, Helen Y. Chu, Trevor Bedford, Jay Shendure
EPI_ISL_2692243, EPI_ISL_2692266, EPI_ISL_2692284	Atlas Genomics	Seattle Flu Study	Deborah A. Nickerson, Chris D. Frazar, Jover Lee, Benjamin Pelle, Erica Ryke, Matthew Richardson, Amanda Adler, Elisabeth Brandstetter, Peter D. Han, Kairsten Fay, Misja Ilcisin, Kirsten Lacombe, Thomas R. Sibley, Melissa Truong, Caitlin R. Wolf, Romesh Gautom, Geoff Melly, Brian Hiatt, Philip Dykema, Scott Lindquist, Michael Boeckh, Janet A. Englund, Michael Famulare, Barry R. Lutz, Mark J. Rieder, Lea M. Starita, Matthew Thompson, Helen Y. Chu, Jay Shendure, Trevor Bedford
EPI_ISL_2692309	Seattle Flu Study	Seattle Flu Study	Deborah A. Nickerson, Chris D. Frazar, Jover Lee, Benjamin Pelle, Erica Ryke, Matthew Richardson, Amanda Adler, Elisabeth Brandstetter, Peter D. Han, Kairsten Fay, Misja Ilcisin, Kirsten Lacombe, Thomas R. Sibley, Melissa Truong, Caitlin R. Wolf, Karen Cowgill, Stephanie Schrag, Jeff Duchin, Michael Boeckh, Janet A. Englund, Michael Famulare, Barry R. Lutz, Mark J. Rieder, Lea M. Starita, Matthew Thompson, Helen Y. Chu, Trevor Bedford, Jay Shendure
EPI_ISL_2692395, EPI_ISL_2692398	Northwest Laboratory	Seattle Flu Study	Deborah A. Nickerson, Chris D. Frazar, Jover Lee, Benjamin Pelle, Erica Ryke, Matthew Richardson, Amanda Adler, Elisabeth Brandstetter, Peter D. Han, Kairsten Fay, Misja Ilcisin, Kirsten Lacombe, Thomas R. Sibley, Melissa Truong, Caitlin R. Wolf, Romesh Gautom, Geoff Melly, Brian Hiatt, Philip Dykema, Scott Lindquist, Michael Boeckh, Janet A. Englund, Michael Famulare, Barry R. Lutz, Mark J. Rieder, Lea M. Starita, Matthew Thompson, Helen Y. Chu, Jay Shendure, Trevor Bedford
EPI_ISL_2692424, EPI_ISL_2692432	Incyte Diagnostics Spokane	Seattle Flu Study	Deborah A. Nickerson, Chris D. Frazar, Jover Lee, Benjamin Pelle, Erica Ryke, Matthew Richardson, Amanda Adler, Elisabeth Brandstetter, Peter D. Han, Kairsten Fay, Misja Ilcisin, Kirsten Lacombe, Thomas R. Sibley, Melissa Truong, Caitlin R. Wolf, Romesh Gautom, Geoff Melly, Brian Hiatt, Philip Dykema, Scott Lindquist, Michael Boeckh, Janet A. Englund, Michael Famulare, Barry R. Lutz, Mark J. Rieder, Lea M. Starita, Matthew Thompson, Helen Y. Chu, Jay Shendure, Trevor Bedford
EPI_ISL_2692462, EPI_ISL_2692464, EPI_ISL_2692466, EPI_ISL_2692473, EPI_ISL_2692480, EPI_ISL_2692485	Atlas Genomics	Seattle Flu Study	Deborah A. Nickerson, Chris D. Frazar, Jover Lee, Benjamin Pelle, Erica Ryke, Matthew Richardson, Amanda Adler, Elisabeth Brandstetter, Peter D. Han, Kairsten Fay, Misja Ilcisin, Kirsten Lacombe, Thomas R. Sibley, Melissa Truong, Caitlin R. Wolf, Romesh Gautom, Geoff Melly, Brian Hiatt, Philip Dykema, Scott Lindquist, Michael Boeckh, Janet A. Englund, Michael Famulare, Barry R. Lutz, Mark J. Rieder, Lea M. Starita, Matthew Thompson, Helen Y. Chu, Jay Shendure, Trevor Bedford
EPI_ISL_2692515	Overlake Hospital	Seattle Flu Study	Deborah A. Nickerson, Chris D. Frazar, Jover Lee, Benjamin Pelle, Erica Ryke, Matthew Richardson, Amanda Adler, Elisabeth Brandstetter, Peter D. Han, Kairsten Fay, Misja Ilcisin, Kirsten Lacombe, Thomas R. Sibley, Melissa Truong, Caitlin R. Wolf, Romesh Gautom, Geoff Melly, Brian Hiatt, Philip Dykema, Scott Lindquist, Michael Boeckh, Janet A. Englund, Michael Famulare, Barry R. Lutz, Mark J. Rieder, Lea M. Starita, Matthew Thompson, Helen Y. Chu, Jay Shendure, Trevor Bedford
EPI_ISL_2693294, EPI_ISL_2693298, EPI_ISL_2693305	UW Virology Lab	UW Virology Lab	Pavitra Roychoudhury, Hong Xie, Lasata Shrestha, Tien V. Nguyen, Shah Mohamed Bakhash, Michelle Lin, Noah R. Baker, Ricardo Perez, Sean Ellis, Nathan Breit, Robert J. Livingston, Meei-Li Huang, Keith R Jerome, Patrick Mathias, Alexander Greninger
EPI_ISL_2693356, EPI_ISL_2693357	ISDH	Quantigen Biosciences	Paul J. Childress
EPI_ISL_2693699, EPI_ISL_2693700, EPI_ISL_2693701, EPI_ISL_2693727, EPI_ISL_2693733, EPI_ISL_2693750, EPI_ISL_2693785, EPI_ISL_2693800	UW Virology Lab	UW Virology Lab	Pavitra Roychoudhury, Hong Xie, Lasata Shrestha, Shah Mohamed Bakhash, Tien V. Nguyen, Noah R. Baker, Sean Ellis, Meei-Li Huang, Keith R Jerome, Alexander Greninger
EPI_ISL_2696869	Laboratory, Gravity Diagnostics	Laboratory, Gravity Diagnostics	Diagnostics,G.
EPI_ISL_2698120, EPI_ISL_2698128, EPI_ISL_2698143, EPI_ISL_2698145, EPI_ISL_2698195, EPI_ISL_2698206, EPI_ISL_2698217, EPI_ISL_2698244, EPI_ISL_2698255, EPI_ISL_2698263, EPI_ISL_2698311, EPI_ISL_2698315, EPI_ISL_2698454, EPI_ISL_2698456, EPI_ISL_2698459, EPI_ISL_2698485, EPI_ISL_2698497, EPI_ISL_2698508, EPI_ISL_2698569, EPI_ISL_2698573, EPI_ISL_2699233, EPI_ISL_2699252, EPI_ISL_2699254, EPI_ISL_2699264, EPI_ISL_2699270, EPI_ISL_2699280, EPI_ISL_2699309, EPI_ISL_2699323, EPI_ISL_2699343, EPI_ISL_2699349, EPI_ISL_2699360, EPI_ISL_2699373, EPI_ISL_2699382, EPI_ISL_2709029, EPI_ISL_2709040, EPI_ISL_2709071, EPI_ISL_2709090, EPI_ISL_2709118, EPI_ISL_2709134, EPI_ISL_2709148, EPI_ISL_2709156, EPI_ISL_2709185			

see above	Public Health Laboratory, Minnesota Department of Health	University of Minnesota Genomics Center	Daryl M. Gohl, John Garbe, Jaquelyn Kuriger-Laber, Corbin Dirkx, and Sean Wang
EPI_ISL_2712539	Sharp HealthCare Laboratory	Andersen lab at Scripps Research	SEARCH Alliance San Diego with Aaron Harding, Jacquelyn Berumen, Cathy Woerle, Liam McGinnis, Art Mendoza, Omid Bakhtar
EPI_ISL_2712831	San Diego County Public Health Laboratory	Andersen lab at Scripps Research	SEARCH Alliance San Diego with Ashleigh Murphy, Jovan Shephard, Brett Austin
EPI_ISL_2714923, EPI_ISL_2714936	Incyte Diagnostics Spokane	Seattle Flu Study	Deborah A. Nickerson, Chris D. Frazar, Jover Lee, Benjamin Pelle, Erica Ryke, Matthew Richardson, Amanda Adler, Elisabeth Brandstetter, Peter D. Han, Kairsten Fay, Misja Ilcisin, Kirsten Lacombe, Thomas R. Sibley, Melissa Truong, Caitlin R. Wolf, Romesh Gautom, Geoff Melly, Brian Hiatt, Philip Dykema, Scott Lindquist, Michael Boeckh, Janet A. Englund, Michael Famulare, Barry R. Lutz, Mark J. Rieder, Lea M. Starita, Matthew Thompson, Helen Y. Chu, Jay Shendure, Trevor Bedford
EPI_ISL_2714947, EPI_ISL_2714950, EPI_ISL_2714961, EPI_ISL_2714979, EPI_ISL_2714984, EPI_ISL_2714985, EPI_ISL_2715000, EPI_ISL_2715001, EPI_ISL_2715006	Interpath Laboratory	Seattle Flu Study	Deborah A. Nickerson, Chris D. Frazar, Jover Lee, Benjamin Pelle, Erica Ryke, Matthew Richardson, Amanda Adler, Elisabeth Brandstetter, Peter D. Han, Kairsten Fay, Misja Ilcisin, Kirsten Lacombe, Thomas R. Sibley, Melissa Truong, Caitlin R. Wolf, Romesh Gautom, Geoff Melly, Brian Hiatt, Philip Dykema, Scott Lindquist, Michael Boeckh, Janet A. Englund, Michael Famulare, Barry R. Lutz, Mark J. Rieder, Lea M. Starita, Matthew Thompson, Helen Y. Chu, Jay Shendure, Trevor Bedford
EPI_ISL_2715089	Incyte Diagnostics Spokane	Seattle Flu Study	Deborah A. Nickerson, Chris D. Frazar, Jover Lee, Benjamin Pelle, Erica Ryke, Matthew Richardson, Amanda Adler, Elisabeth Brandstetter, Peter D. Han, Kairsten Fay, Misja Ilcisin, Kirsten Lacombe, Thomas R. Sibley, Melissa Truong, Caitlin R. Wolf, Romesh Gautom, Geoff Melly, Brian Hiatt, Philip Dykema, Scott Lindquist, Michael Boeckh, Janet A. Englund, Michael Famulare, Barry R. Lutz, Mark J. Rieder, Lea M. Starita, Matthew Thompson, Helen Y. Chu, Jay Shendure, Trevor Bedford
EPI_ISL_2715203	Atlas Genomics	Seattle Flu Study	Deborah A. Nickerson, Chris D. Frazar, Jover Lee, Benjamin Pelle, Erica Ryke, Matthew Richardson, Amanda Adler, Elisabeth Brandstetter, Peter D. Han, Kairsten Fay, Misja Ilcisin, Kirsten Lacombe, Thomas R. Sibley, Melissa Truong, Caitlin R. Wolf, Romesh Gautom, Geoff Melly, Brian Hiatt, Philip Dykema, Scott Lindquist, Michael Boeckh, Janet A. Englund, Michael Famulare, Barry R. Lutz, Mark J. Rieder, Lea M. Starita, Matthew Thompson, Helen Y. Chu, Jay Shendure, Trevor Bedford
EPI_ISL_2720367, EPI_ISL_2720671, EPI_ISL_2720750, EPI_ISL_2721512	BCCDC Public Health Laboratory	BCCDC Public Health Laboratory	Prystajecky Natalie, Linda Hoang, Dan Fornika, John Tyson, Shannon Russell, Kim Macdonald, Kimia Kamelian, Ana Pacagnella, Corrinne Ng, Loretta Janz, Robert Azana, Mel Krajden
EPI_ISL_2724528, EPI_ISL_2724541, EPI_ISL_2724566, EPI_ISL_2724591, EPI_ISL_2724594, EPI_ISL_2724595, EPI_ISL_2724596, EPI_ISL_2724604, EPI_ISL_2724616, EPI_ISL_2724623, EPI_ISL_2724625, EPI_ISL_2724626, EPI_ISL_2724632, EPI_ISL_2724634, EPI_ISL_2724638, EPI_ISL_2724640, EPI_ISL_2724648, EPI_ISL_2724653, EPI_ISL_2724654, EPI_ISL_2724662, EPI_ISL_2724663, EPI_ISL_2724668, EPI_ISL_2724674, EPI_ISL_2724675, EPI_ISL_2724682, EPI_ISL_2724688, EPI_ISL_2724702, EPI_ISL_2724706, EPI_ISL_2724714, EPI_ISL_2724716, EPI_ISL_2724726, EPI_ISL_2724736, EPI_ISL_2724747, EPI_ISL_2724758, EPI_ISL_2724765, EPI_ISL_2724802, EPI_ISL_2724804, EPI_ISL_2724808, EPI_ISL_2724809, EPI_ISL_2724810, EPI_ISL_2724817, EPI_ISL_2724821, EPI_ISL_2724827, EPI_ISL_2724838, EPI_ISL_2724840, EPI_ISL_2724875, EPI_ISL_2724895, EPI_ISL_2724907, EPI_ISL_2724912			
see above	State Laboratories Division, Hawaii State Department of Health	State Laboratories Division, Hawaii State Department of Health	Pamela O'Brien, Drew Kuwazaki, Ayana Garnet, Razvan Sultana, Edward Desmond
EPI_ISL_2735972, EPI_ISL_2736001	Incyte Diagnostics Spokane	Seattle Flu Study	Deborah A. Nickerson, Chris D. Frazar, Jover Lee, Benjamin Pelle, Erica Ryke, Matthew Richardson, Amanda Adler, Elisabeth Brandstetter, Peter D. Han, Kairsten Fay, Misja Ilcisin, Kirsten Lacombe, Thomas R. Sibley, Melissa Truong, Caitlin R. Wolf, Romesh Gautom, Geoff Melly, Brian Hiatt, Philip Dykema, Scott Lindquist, Michael Boeckh, Janet A. Englund, Michael Famulare, Barry R. Lutz, Mark J. Rieder, Lea M. Starita, Matthew Thompson, Helen Y. Chu, Jay Shendure, Trevor Bedford
EPI_ISL_2736825	LESP Michoacan	Instituto de Diagnostico y Referencia Epidemiologicos (INDRE)	Claudia Wong-Arambula, Abril Rodriguez-Maldonado, Vanessa Rivero-Arredondo, Ariadna Medina-Benitez, Joaquin Quiroz-Mercado, Sergio Rangel-Guerrero, Natividad Cruz-Ortiz, Tatiana Nunez-Garcia, Gisela Barrera-Badillo, Lucia Hernandez-Rivas, Irma Lopez-Martinez, Ernesto Ramirez-Gonzalez.
EPI_ISL_2736827	LESP Guanajuato	Instituto de Diagnostico y Referencia Epidemiologicos (INDRE)	Claudia Wong-Arambula, Abril Rodriguez-Maldonado, Vanessa Rivero-Arredondo, Ariadna Medina-Benitez, Joaquin Quiroz-Mercado, Sergio Rangel-Guerrero, Natividad Cruz-Ortiz, Tatiana Nunez-Garcia, Gisela Barrera-Badillo, Lucia Hernandez-Rivas, Irma Lopez-Martinez, Ernesto Ramirez-Gonzalez.
EPI_ISL_2736829	LESP Coahuila	Instituto de Diagnostico y Referencia Epidemiologicos (INDRE)	Claudia Wong-Arambula, Abril Rodriguez-Maldonado, Vanessa Rivero-Arredondo, Ariadna Medina-Benitez, Joaquin Quiroz-Mercado, Sergio Rangel-Guerrero, Natividad Cruz-Ortiz, Tatiana Nunez-Garcia, Gisela Barrera-Badillo, Lucia Hernandez-Rivas, Irma Lopez-Martinez, Ernesto Ramirez-Gonzalez.
EPI_ISL_2736832	LESP Baja California	Instituto de Diagnostico y Referencia Epidemiologicos (INDRE)	Claudia Wong-Arambula, Abril Rodriguez-Maldonado, Vanessa Rivero-Arredondo, Ariadna Medina-Benitez, Joaquin Quiroz-Mercado, Sergio Rangel-Guerrero, Natividad Cruz-Ortiz, Tatiana Nunez-Garcia, Gisela Barrera-Badillo, Lucia Hernandez-Rivas, Irma Lopez-Martinez, Ernesto Ramirez-Gonzalez.
EPI_ISL_2736834	LESP Michoacan	Instituto de Diagnostico y Referencia Epidemiologicos (INDRE)	Claudia Wong-Arambula, Abril Rodriguez-Maldonado, Vanessa Rivero-Arredondo, Ariadna Medina-Benitez, Joaquin Quiroz-Mercado, Sergio Rangel-Guerrero, Natividad Cruz-Ortiz, Tatiana Nunez-Garcia, Gisela Barrera-Badillo, Lucia Hernandez-Rivas, Irma Lopez-Martinez, Ernesto Ramirez-Gonzalez.
EPI_ISL_2756322	Genetica Molecular and Subdepartamento de Virologia ISP Chile	Instituto de Salud Publica de Chile	Karen Orostica, Constanza Campano, Barbara Parra, Loredana Arata, Gisselle Barra, Patricia Bustos, Rodrigo Fasce, Javier Tognarelli, Andres Castillo, Soledad Ulloa, Jorge Fernandez
EPI_ISL_2759228, EPI_ISL_2759231, EPI_ISL_2759243, EPI_ISL_2759331, EPI_ISL_2759334	Population Medicine and Diagnostic Sciences, Cornell University	Population Medicine and Diagnostic Sciences, Cornell University	Mitchell,P.K., Caserta,L.C., Cronk,B.D., Anderson,R.R., Laverack,M., Venugopalan,R., Plocharczyk,E., Diel,D.G.

Supp. Table S6

We gratefully acknowledge the following Authors from the Originating laboratories responsible for obtaining the specimens, as well as the Submitting laboratories where the genome data were generated and shared via GISAID, on which this research is based.

All Submitters of data may be contacted directly via www.gisaid.org

Authors are sorted alphabetically.

Accession ID	Originating Laboratory	Submitting Laboratory	Authors
EPI_ISL_2466750	Nebraska Public Health Laboratory	NPHL COVID-19 Response Team	NPHL COVID-19 Response Team
EPI_ISL_2466754, EPI_ISL_2466757	Wichita State University - Molecular Diagnostics Lab	Kansas Health and Environmental Lab	Mike Grose, Katherine Wiggins, Jonathan Barnell, Ben Olsen, and Phil Adam
EPI_ISL_2473665	Oregon State Public Health Laboratory	Oregon State Public Health Laboratory	Rafia Razzaque, Eugene Yeboah, Vanda Makris, Laura Tsaknaridis, John Fontana and Shane Sevey
EPI_ISL_2479923	UPA UNIDADE DE PRONTO ATENDIMENTO 24 HORAS BOM JESUS	Instituto Butantan	Dimas Tadeu Covas, Antonio Jorge Martins, Claudia Renata dos Santos Barros, David Schlesinger, Debora Botequio Moretti, Elaine Cristina Marqueze, Elaine Vieira Santos, Evandra Strazza Rodrigues, Heidge Fukumasu, Jayme Augusto de Souza-Neto, José Salvatore Leister Patané, Luiz Alcantara, Luiz Lehmann Coutinho, Maria Carolina Elias, Maurício Lacerda Nogueira, Rafael dos Santos Bezerra, Raul Machado Neto, Rejane Maria Tommasini Grotto, Ricardo Haddad, Sandra Coccuzzo Sampaio Vessoni, Simone Kashima, Svetoslav Nanev Slavov, Vincent Louis Viala
EPI_ISL_2483256, EPI_ISL_2483279, EPI_ISL_2483291, EPI_ISL_2483294, EPI_ISL_2483300	Platform BIS UZA/UAntwerpen	Labo Klinische Biologie, UZA	Jasmine Coppens, Marie Le Mercier, Basil Britto Xavier, Christine Lammens, Veerle Matheeussen, Herman Goossens
EPI_ISL_2484017, EPI_ISL_2484018	Hospital General Universitario Gregorio Marañón	Hospital General Universitario Gregorio Marañón	Sergio Buenestado Serrano, Pedro Sola Campoy, Laura Pérez-Lago, Cristina Rodriguez-Grande, Marta Herranz Martin, Victor Manuel de la Cueva, Julia Suárez, Pilar Catalán, Patricia Muñoz, Darío García de Viedma
EPI_ISL_2492423, EPI_ISL_2492425	Cliniques universitaires Saint-Luc	UCLouvain/IREC/MBLG	Jean Ruelle, Benoit Kabamba Mukadi
EPI_ISL_2499942, EPI_ISL_2499954	Wichita State University - Molecular Diagnostics Lab	Kansas Health and Environmental Lab	Mike Grose, Katherine Wiggins, Jonathan Barnell, Ben Olsen, and Phil Adam
EPI_ISL_2499975, EPI_ISL_2499977, EPI_ISL_2499978, EPI_ISL_2499979, EPI_ISL_2499981, EPI_ISL_2499983	SYNLAB	GIGA Medical Genomics	Keith Durkin, Maria Artesi, Sébastien Bontems, Raphaël Boreux, Bouchra Boujemla, Nathalie Renotte, Cécile Meex, Pierrette Melin, Marie-Pierre Hayette, Vincent Bours
EPI_ISL_2500004, EPI_ISL_2500005	University of Liège COVID-19 testing center	GIGA Medical Genomics	Keith Durkin, Maria Artesi, Bouchra Boujemla, Nathalie Renotte, Cécile Meex, Sébastien Bontems, Fabrice Bureau, Laurent Gillet, Wouter Coppieters, Marie-Pierre Hayette, Vincent Bours
EPI_ISL_2501152	Minnesota Department of Health, Public Health Laboratory	Minnesota Department of Health, Public Health Laboratory	Alexandra Lorentz, Jacob Garfin, Matt Plumb, and Xiong Wang
EPI_ISL_2504004	Alaska State Virology Laboratory	Alaska State Virology Laboratory	Stephanie DeRonde, Elva House, Jacob Zidek, Lisa Smith, Ph.D., Jack Chen, Ph.D.
EPI_ISL_2516752, EPI_ISL_2516755, EPI_ISL_2516859	Servicio de Microbiología Clínica (Complejo Hospitalario de Navarra, Pamplona)	Centro de Secuenciación NASERTIC	Carmen Ezpeleta Baquedano, Ana Navascués, Ana Miqueleiz
EPI_ISL_2521960, EPI_ISL_2521969	Plateforme de testing Namuroise	Plateforme de testing Namuroise	Nicolas Gilliard; Demars Aurore ; Maschietto Céline ; Nobis Chloé ; Otto Gaetan ; Denis Olivier ; Degosserie Jonathan ; Mullier François
EPI_ISL_2524776	Hospital Universitari Arnau de Vilanova	Hospital Universitari Vall d'Hebron - Vall d'Hebron Institut de Recerca	Cristina Andrés, Maria Piñana, Alejandra González-Sánchez, Damir Garcia-Cehic, Ariadna Rando, Juliana Esperalba, Maria Gema Codina, Carla Castillo, Maria Carmen Martin, Tomàs Pumarola, Josep Quer, Andrés Antón
EPI_ISL_2527565, EPI_ISL_2527592, EPI_ISL_2527650, EPI_ISL_2527735, EPI_ISL_2527739, EPI_ISL_2527741, EPI_ISL_2527753, EPI_ISL_2527757, EPI_ISL_2527760, EPI_ISL_2527801, EPI_ISL_2527811, EPI_ISL_2527816			
see above	Fulgent Genetics	Centers for Disease Control and Prevention Division of Viral Diseases, Pathogen Discovery	Dakota Howard, Dhwani Batra, Peter W. Cook, Kara Moser, Adrian Paskey, Jason Caravas, Benjamin Rambo-Martin, Shatavia Morrison, Christopher Gulvick, Scott Sammons, Yvette Unoarumhi, Darlene Wagner, Matthew Schmerer, Harry Gao, Mickey Li, John Gao, Joseph Fierro, Benafsh Sapra, Becky Tsai, Yan Meng, Doreen Ng, James Xie, Clinton R. Paden, Duncan MacCannell
EPI_ISL_2527882, EPI_ISL_2527899, EPI_ISL_2527909, EPI_ISL_2527937, EPI_ISL_2527954, EPI_ISL_2527961, EPI_ISL_2527974, EPI_ISL_2527995, EPI_ISL_2528163, EPI_ISL_2528165, EPI_ISL_2528218, EPI_ISL_2528226, EPI_ISL_2528233, EPI_ISL_2528235, EPI_ISL_2528237, EPI_ISL_2528283, EPI_ISL_2528299, EPI_ISL_2528310, EPI_ISL_2528318, EPI_ISL_2528372, EPI_ISL_2528380, EPI_ISL_2528394, EPI_ISL_2528419, EPI_ISL_2528425, EPI_ISL_2528428, EPI_ISL_2528862, EPI_ISL_2529005			
see above	Aegis Sciences Corporation	Centers for Disease Control and Prevention Division of Viral Diseases, Pathogen Discovery	Dakota Howard, Dhwani Batra, Peter W. Cook, Kara Moser, Adrian Paskey, Jason Caravas, Benjamin Rambo-Martin, Shatavia Morrison, Christopher Gulvick, Scott Sammons, Yvette Unoarumhi, Darlene Wagner, Matthew Schmerer, Cyndi Clark, Patrick Campbell, Rob Case, Vikramsinha Ghorpade, Holly Houdeshell, Ola Kvalvaag, Dillon Nall, Ethan Sanders, Alec Vest, Shaun Westlund, Matthew Hardison, Clinton R. Paden, Duncan MacCannell
EPI_ISL_2534256, EPI_ISL_2534262	Wichita State University - Molecular Diagnostics Lab	Kansas Health and Environmental Lab	Mike Grose, Katherine Wiggins, Jonathan Barnell, Ben Olsen, and Phil Adam
EPI_ISL_2534552	Aegis Sciences Corporation	Centers for Disease Control and Prevention Division of Viral Diseases, Pathogen Discovery	Dakota Howard, Dhwani Batra, Peter W. Cook, Kara Moser, Adrian Paskey, Jason Caravas, Benjamin Rambo-Martin, Shatavia Morrison, Christopher Gulvick, Scott Sammons, Yvette Unoarumhi, Darlene Wagner, Matthew Schmerer, Cyndi Clark, Patrick Campbell, Rob Case, Vikramsinha Ghorpade, Holly Houdeshell, Ola Kvalvaag, Dillon Nall, Ethan Sanders, Alec Vest, Shaun Westlund, Matthew Hardison, Clinton R. Paden, Duncan MacCannell
EPI_ISL_2536905	Centracare Laboratory Services	Minnesota Department of Health, Public Health Laboratory	Alexandra Lorentz, Jacob Garfin, Matt Plumb, and Xiong Wang
EPI_ISL_2544257	Medica	Institute of Medical Virology	Verena Kufner, Gabriela Ziltener, Maryam Zaheri, Stefan Schmutz, Annette Audigé, Maria Grünberg, Kevin Steiner, Jon Huder, Cyril Shah, Riccarda Capaul, Guido Bloemberg, Jürg Böni, Michael Huber, Alexandra Trkola
EPI_ISL_2544269	UniversitätsSpital Zürich 009	Institute of Medical Virology	Verena Kufner, Gabriela Ziltener, Maryam Zaheri, Stefan Schmutz, Annette Audigé, Maria Grünberg, Kevin Steiner, Jon Huder, Cyril Shah, Riccarda Capaul, Guido Bloemberg, Jürg Böni, Michael Huber, Alexandra Trkola
EPI_ISL_2544982	UW Virology Lab	UW Virology Lab	Pavitra Roychoudhury, Hong Xie, Lasata Shrestha, Shah Mohamed Bakhash, Tien V. Nguyen, Noah R. Baker, Sean Ellis, Meei-Li Huang, Keith R Jerome, Alexander Greninger
EPI_ISL_2545446, EPI_ISL_2545468	National Platform bis UMONS/Jolimont	National Platform bis UMONS/Jolimont	François Dufrasne, Guillaume Bayon-Vicente, Florian Juszczak, Gautier Detry, Ruddy Wattiez
EPI_ISL_2545596, EPI_ISL_2545598, EPI_ISL_2545599, EPI_ISL_2545600, EPI_ISL_2545610, EPI_ISL_2545636	Platform BIS UZA/UAntwerpen	Labo Klinische Biologie, UZA	Jasmine Coppens, Marie Le Mercier, Basil Britto Xavier, Christine Lammens, Veerle Matheeussen, Herman Goossens
EPI_ISL_2545657, EPI_ISL_2545658	LHUB-ULB	Labo Klinische Biologie, UZA	Jasmine Coppens, Marie Le Mercier, Basil Britto Xavier, Christine Lammens, Veerle Matheeussen, Herman Goossens
EPI_ISL_2549337, EPI_ISL_2549343, EPI_ISL_2549346, EPI_ISL_2549354, EPI_ISL_2549359, EPI_ISL_2549371, EPI_ISL_2549378, EPI_ISL_2549382, EPI_ISL_2549385, EPI_ISL_2549399, EPI_ISL_2549409, EPI_ISL_2549432, EPI_ISL_2549442, EPI_ISL_2549447, EPI_ISL_2549450, EPI_ISL_2549451, EPI_ISL_2549458, EPI_ISL_2549471, EPI_ISL_2549476, EPI_ISL_2549478, EPI_ISL_2549479, EPI_ISL_2549481, EPI_ISL_2549493, EPI_ISL_2549495, EPI_ISL_2549496, EPI_ISL_2549504, EPI_ISL_2549507, EPI_ISL_2549519			
see above	UW Virology Lab	UW Virology Lab	Pavitra Roychoudhury, Hong Xie, Lasata Shrestha, Tien V. Nguyen, Shah Mohamed Bakhash, Michelle Lin, Noah R. Baker, Ricardo Perez, Sean Ellis, Nathan Breit, Robert J. Livingston, Meei-Li Huang, Keith R Jerome, Patrick Mathias, Alexander Greninger
EPI_ISL_2550983, EPI_ISL_2551117, EPI_ISL_2551127	Istituto Zooprofilattico Sperimentale del Mezzogiorno	TIGEM	Antonio Grimaldi Patrizia Annunziata Francesco Panariello Biancamaria Pierri Claudia Tiberio Teresa Giuliano Valentina Bouche Chiara Colantuono Maria Concetta Cuomo Denise Di Concilio Lucio Di Filippo Anna Manfredi Marcello Salvi Antonio Limone Luigi Atripaldi Pellegrino Cerino Andrea Ballabio Davide Cacchiarelli
EPI_ISL_2557148, EPI_ISL_2557157	NJDOH, Public Health and Environmental Laboratories	NJ_PHEL	Lindsey Bodnar, Shiv K. Verma, Ryan Pachucki, Chelsea San Filippo, Dana Woell, Allison Roder, Byeong Jeong

EPI_ISL_2557242	IN State Department of Health Laboratory Services	IN State Department of Health Laboratory Services	Cassandra Campion, Jamie Yeadon, Brian Pope, Lixia Liu, Kyle Brownlee, Melissa Hindenlang, Mark Glazier
EPI_ISL_2557409, EPI_ISL_2557410	Laboratory of Respiratory Viruses and Measles, Oswaldo Cruz Institute, FIOCRUZ	Laboratory of Respiratory Viruses and Measles, Oswaldo Cruz Institute, FIOCRUZ	Paola Resende, Luciana Appolinario, Fernando Motta, Anna Carolina Paixao, Ana Carolina Mendonca, Alice Sampaio Rocha, Taina Venas, Elisa Cavalcante Pereira, Renata Serrano Lopes, Marilda Siqueira on behalf of the Fiocruz COVID-19 Genomic Surveillance Network
EPI_ISL_2558702	Kansas Health and Environmental Lab	Kansas Health and Environmental Lab	Katherine Wiggins, Mike Grose, Jonathan Barnell, Ben Olsen, and Phil Adam
EPI_ISL_2574072, EPI_ISL_2574075	Center for Laboratory Medicine	Center for Laboratory Medicine	Yannick Gerth
EPI_ISL_2597899	WSSE w Gdasku	1. Tricity SARS-CoV-2 sequencing consortium: University of Gdansk, Medical University of Gdansk, Vaxican Ltd., Invicta Ltd. 2. National Institute of Public Health - National Institute of Hygiene, Warsaw, Poland	Maciej Kosinski, Celina Cybulska, Krystyna Bienkowska Szewczyk, Maciej Grzybek, Karolina Gackowska, Marcin Lubocki, Katarzyna Groth, Lukasz Rabalski, Katarzyna Zacharczuk, Magdalena Nowakowska, Magorzata Sadkowska-Todys, Tomasz Wokowicz
EPI_ISL_2597931	Nebraska Public Health Laboratory	NPHL COVID-19 Response Team	NPHL COVID-19 Response Team
EPI_ISL_2598160	Minnesota Department of Health, Public Health Laboratory	Minnesota Department of Health, Public Health Laboratory	Alexandra Lorentz, Jacob Garfin, Matt Plumb, and Xiong Wang
EPI_ISL_2598704, EPI_ISL_2598710, EPI_ISL_2598724, EPI_ISL_2598726, EPI_ISL_2598733, EPI_ISL_2598764, EPI_ISL_2598778, EPI_ISL_2598791, EPI_ISL_2598822, EPI_ISL_2598828, EPI_ISL_2599922, EPI_ISL_2599923			
see above	Fulgent Genetics	Centers for Disease Control and Prevention Division of Viral Diseases, Pathogen Discovery	Dakota Howard, Dhwani Batra, Peter W. Cook, Kara Moser, Adrian Paskey, Jason Caravas, Benjamin Rambo-Martin, Shatavia Morrison, Christopher Gulvick, Scott Sammons, Yvette Unoarumhi, Darlene Wagner, Matthew Schmerer, Harry Gao, Mickey Li, John Gao, Joseph Fierro, Benafsh Sapra, Becky Tsai, Yan Meng, Doreen Ng, James Xie, Clinton R. Paden, Duncan MacCannell
EPI_ISL_2600917	Servicio de Microbiología. Hospital Clínico Universitario de Valencia	SeqCOVID-SPAIN consortium/IBV(CSIC)	David Navarro Ortega, Eliseo Albert Vicent, Ignacio Torres and SeqCOVID-SPAIN consortium
EPI_ISL_2600943, EPI_ISL_2600944, EPI_ISL_2600948	Plateforme de testing Namuroise	Plateforme de testing Namuroise	Nicolas GILLIARD, Lesly NYINKEU KEMAMEN; Demars Aurore ; Maschietto Céline ; Nobis Chloé ; Otto Gaetan ; Denis Olivier ; Degosserie Jonathan ; Mullier François
EPI_ISL_2601701	Hospital General Universitario Gregorio Marañón	Hospital General Universitario Gregorio Marañón	Sergio Buenestado Serrano, Pedro Sola Campoy, Laura Pérez-Lago, Cristina Rodriguez-Grande, Marta Herranz Martin, Victor Manuel de la Cueva, Julia Suárez, Pilar Catalán, Patricia Muñoz, Darío García de Viedma
EPI_ISL_2601973, EPI_ISL_2601975, EPI_ISL_2601979	Jessa	Jessa	Berden et al. on behalf of the Jessa_cmdLab
EPI_ISL_2604198, EPI_ISL_2604209	Oregon State Public Health Laboratory	Oregon State Public Health Laboratory	Rafia Razzaque, Eugene Yeboah, Vanda Makris, Laura Tsaknaridis, John Fontana and Shane Sevey
EPI_ISL_2604871	Omics Sciences Laboratory	Omics Sciences Laboratory	Derly Andrade Molina, Gabriel Morey León, Darlyn Amaya, Rubén Armas González, Juan Carlos Fernández Cadena
EPI_ISL_2604875, EPI_ISL_2604876, EPI_ISL_2604877	INTERLAB	Omics Sciences Laboratory	Derly Andrade Molina, Gabriel Morey León, Darlyn Amaya, Rubén Armas González, Juan Carlos Fernández Cadena
EPI_ISL_2610665, EPI_ISL_2610672, EPI_ISL_2610673, EPI_ISL_2610674, EPI_ISL_2610691, EPI_ISL_2610697, EPI_ISL_2610700, EPI_ISL_2610733, EPI_ISL_2610743, EPI_ISL_2610753, EPI_ISL_2610754, EPI_ISL_2610765, EPI_ISL_2610766, EPI_ISL_2610776			
see above	Dutch COVID-19 response team	National Institute for Public Health and the Environment (RIVM)	Adam Meijer, Harry Vennema, Dirk Eggink, Jeroen Cremer, Sharon van den Brink, Bas van der Veer, AnneMarie van den Brandt, Lisa Wijsman, Kim Freriks, Ryanne Jaarsma, Euníce Then, Lynn Aarts, Sanne Bos, Melissa van Tuil, Linda van de Nes, Florian Zwagemaker, Dennis Schmitz, Annelies Kroneman, Karim Hajji, Chantal Reusken, on behalf of the national COVID-19 response team
EPI_ISL_2611074	Viollier AG	Department of Biosystems Science and Engineering, ETH Zürich	Christian Beisel, Sarah Nadeau, Chaoran Chen, Ivan Topolsky, Kim Philipp Jablonski, Lara Fuhrmann, Rebecca Denes, Mirjam Feldkamp, Ina Nissen, Natascha Santacroce, Elodie Burcklen, Christiane Beckmann, Maurice Redondo, Olivier Kobel, Christoph Noppen, Niko Beerenwinkel, Tanja Stadler
EPI_ISL_2611209, EPI_ISL_2611229, EPI_ISL_2611230, EPI_ISL_2611232, EPI_ISL_2611233, EPI_ISL_2611238, EPI_ISL_2611239, EPI_ISL_2611240, EPI_ISL_2611242, EPI_ISL_2611304, EPI_ISL_2611313, EPI_ISL_2611314, EPI_ISL_2611321, EPI_ISL_2611323, EPI_ISL_2611324, EPI_ISL_2611332, EPI_ISL_2611334, EPI_ISL_2611338, EPI_ISL_2611344, EPI_ISL_2611349, EPI_ISL_2611364, EPI_ISL_2611372, EPI_ISL_2611380, EPI_ISL_2611384, EPI_ISL_2611392, EPI_ISL_2611397, EPI_ISL_2611398, EPI_ISL_2611413, EPI_ISL_2611418, EPI_ISL_2611420, EPI_ISL_2611428, EPI_ISL_2611607, EPI_ISL_2611626, EPI_ISL_2611627, EPI_ISL_2611631, EPI_ISL_2611645, EPI_ISL_2611646, EPI_ISL_2611656, EPI_ISL_2611657, EPI_ISL_2611692, EPI_ISL_2611767, EPI_ISL_2611772, EPI_ISL_2611812, EPI_ISL_2611823			
see above	Laboratory Corporation of America	Centers for Disease Control and Prevention Division of Viral Diseases, Pathogen Discovery	Dakota Howard, Dhwani Batra, Peter W. Cook, Kara Moser, Adrian Paskey, Jason Caravas, Benjamin Rambo-Martin, Shatavia Morrison, Christopher Gulvick, Scott Sammons, Yvette Unoarumhi, Darlene Wagner, Matthew Schmerer, Minoo Agarwal, Eyad Almasri, Debbie Boles, Ayla Burns, Nuthawin Charoensri, Oren Cohen, Susan Countryman, Mary Ann Cristobal, Bobbi Croy, Suzanne Dale, Hrushikesh Deshmukh, Amanda Douglas, Vincent Drouillon, Marcia Eisenberg, Howard Engler, Rama Ghatti, Prashant Gupta, Susan Hicks, Jake Humphrey, Lax Iyer, Lisa Pfefferle, Manoj Jain, Matthew Robinson, Mohan Kolli, Brian Krueger, Tim Kuphal, Stanley Letovsky, Michael Levandoski, Craig Lukasik, Jonathan Meltzer, Brian Norvell, Mindy Nye, Scott Parker, Christos Petropoulos, John Pruitt, Steven Ragan, Scott Ryan, Mike Sapeta, Jana Schroth, Suresh Babu Selvaraju, Goran Stevovic, Amanda Suchanek, Andrea Throop, Lyndon Tilson, Thomas Urban, Joe Voshell, Kimberly Wagner, Jonathan Williams, Mary Williamson, Qian Zeng, Tricia Zwiefelhofer, Clinton R. Paden, Duncan MacCannell
EPI_ISL_2612191, EPI_ISL_2612195	Wichita State University - Molecular Diagnostics Lab	Kansas Health and Environmental Lab	Mike Grose, Katherine Wiggins, Jonathan Barnell, Ben Olsen, and Phil Adam
EPI_ISL_2612507	Nevada State Public Health Laboratory	Nevada State Public Health Laboratory	Andrew Gorzalski, Mark Pandori
EPI_ISL_2612540, EPI_ISL_2612541, EPI_ISL_2612545	Oregon State Public Health Laboratory	Oregon State Public Health Laboratory	Rafia Razzaque, Eugene Yeboah, Vanda Makris, Laura Tsaknaridis, John Fontana and Shane Sevey
EPI_ISL_2612553, EPI_ISL_2612554, EPI_ISL_2612555	DOHMH Corona	New York City Public Health Laboratory	Jade Wang, et al.
EPI_ISL_2616771	Houston Health Dept.	Houston Health Dept.	Ryker Penn, Pamela Brown, Adolpho Lara, Yanlai Lai
EPI_ISL_2617009, EPI_ISL_2617010, EPI_ISL_2617011, EPI_ISL_2617012, EPI_ISL_2617013, EPI_ISL_2617015, EPI_ISL_2617016, EPI_ISL_2617017	Salud Digna	Instituto Nacional de Medicina Genomica	Hidalgo-Miranda A, Cedro-Tanda A, Mendoza-Vargas A, Reyes-Grajeda JP, Abraham Campos-Romero, Moreno-Camacho José Luis, Rodriguez-Gallegos Jorge, Luna-Ruiz Marco, Gonzalez-Barrera D, Rangel-DeLeon D, Munguia-Garza P, Ramirez-Vega O, Escobar-Arrazola, M, Herrera-Montalvo LA.
EPI_ISL_2617603, EPI_ISL_2617605, EPI_ISL_2617606, EPI_ISL_2617622, EPI_ISL_2617623, EPI_ISL_2617624, EPI_ISL_2617625	HLAGYN - Laboratorio de Imunologia de Transplantes de Goias	HLAGYN - Laboratorio de Imunologia de Transplantes de Goias	Fernando Antonio Vinhal dos Santos, Erika Lopes Rocha Batista, Alessandro Leonardo Alvares Magalhaes, Frederico Rodrigues Vinhal, Sabrina Sara Moreira Duarte, Lucas Carlos Gomes Pereira, Daniel Ferreira de Sousa
EPI_ISL_2617628, EPI_ISL_2617970, EPI_ISL_2617975, EPI_ISL_2617987, EPI_ISL_2617992, EPI_ISL_2617997, EPI_ISL_2617998, EPI_ISL_2618002, EPI_ISL_2618003, EPI_ISL_2618004, EPI_ISL_2618005, EPI_ISL_2618012, EPI_ISL_2618023, EPI_ISL_2618038, EPI_ISL_2618049, EPI_ISL_2618053, EPI_ISL_2618056, EPI_ISL_2618070, EPI_ISL_2618085, EPI_ISL_2618088, EPI_ISL_2618098			
see above	UW Virology Lab	UW Virology Lab	Pavitra Roychoudhury, Hong Xie, Lasata Shrestha, Tien V. Nguyen, Shah Mohamed Bakhash, Michelle Lin, Noah R. Baker, Ricardo Perez, Sean Ellis, Nathan Breit, Robert J. Livingston, Meei-Li Huang, Keith R Jerome, Patrick Mathias, Alexander Greninger
EPI_ISL_2620963, EPI_ISL_2620966	Hospital Universitario Virgen de las Nieves de Granada-SAS	SeqCOVID-SPAIN consortium/IBV(CSIC)	Sara Sanbonmatsu Gámez, Irene Pedrosa Corral, José M. Navarro-Marí and SeqCOVID-SPAIN consortium
EPI_ISL_2621554	Genomics, Poplar Healthcare	Genomics, Poplar Healthcare	Truong,M.
EPI_ISL_2621962, EPI_ISL_2621963	National Public Health Laboratory, National Centre for Infectious Diseases	National Public Health Laboratory, National Centre for Infectious Diseases	Tze Minn Mak, Zhenyang Zhou, Royce Ang, Lin Cui, Raymond Tzer Pin Lin
EPI_ISL_2625749, EPI_ISL_2625753	AZDelta	AZ Delta Medical Laboratories in Roeselare, Belgium	Geert Martens, Dieter De Smet, Merijn Vanhee, on behalf of AZ Delta COVID-19 Genomics core (member of Genomic surveillance of SARS-CoV-2 in Belgium network)
EPI_ISL_2626011, EPI_ISL_2626014, EPI_ISL_2626019, EPI_ISL_2626029,	Cliniques universitaires Saint-Luc	UCLouvain/IREC/MBLG	Jean Ruelle, Bertrand Bearzatto, Benoit Kabamba Mukadi

EPI_ISL_2626043			
EPI_ISL_2626066	Clinique Saint-Pierre Ottignies	UCLouvain/IREC/MBLG	Jean Ruelle, Bertrand Bearzatto, Benoit Kabamba Mukadi
EPI_ISL_2626082	Cliniques universitaires Saint-Luc	UCLouvain/IREC/MBLG	Jean Ruelle, Bertrand Bearzatto, Benoit Kabamba Mukadi
EPI_ISL_2626156	HOSPITAL MANACOR	HOSPITAL UNIVERSITARIO SON ESPASES	Servicio de Microbiología, Hospital Universitario Son Espases
EPI_ISL_2626160	HOSPITAL UNIVERSITARIO SON ESPASES	HOSPITAL UNIVERSITARIO SON ESPASES	Servicio de Microbiología, Hospital Universitario Son Espases
EPI_ISL_2626722, EPI_ISL_2626723, EPI_ISL_2626727, EPI_ISL_2626735, EPI_ISL_2626737, EPI_ISL_2626738, EPI_ISL_2626743, EPI_ISL_2626745	Alaska State Virology Laboratory	Alaska State Virology Laboratory	Stephanie DeRonde, Elva House, Jacob Zidek, Lisa Smith, Ph.D., Jack Chen, Ph.D.
EPI_ISL_2626831	Baylor Scott & White-Temple	Baylor Scott & White-Temple	Ari Rao, Linden Morales, Kimberly Walker, Marcus Volz, Shelby Johnson
EPI_ISL_2626853, EPI_ISL_2626882, EPI_ISL_2626883, EPI_ISL_2626884, EPI_ISL_2626903	Platform BIS UZA/UAntwerpen	Labo Klinische Biologie, UZA	Marie Le Mercier, Jasmine Coppens, Basil Britto Xavier, Christine Lammens, Veerle Matheeussen, Herman Goossens
EPI_ISL_2627274	Platform BIS UZA/UAntwerpen	Labo Klinische Biologie, UZA	Jasmine Coppens, Marie Le Mercier, Basil Britto Xavier, Christine Lammens, Veerle Matheeussen, Herman Goossens
EPI_ISL_2627864, EPI_ISL_2627865, EPI_ISL_2627866	CNR Institut Pasteur de la Guyane	Institut Pasteur de la Guyane	Anne Lavergne, Dominique Rousset, Antoine Enfissi, Arielle Salmier
EPI_ISL_2627930, EPI_ISL_2627938, EPI_ISL_2627939	Wichita State University - Molecular Diagnostics Lab	Kansas Health and Environmental Lab	Mike Grose, Katherine Wiggins, Jonathan Barnell, Ben Olsen, and Phil Adam
EPI_ISL_2628061, EPI_ISL_2628062, EPI_ISL_2628063, EPI_ISL_2628064, EPI_ISL_2628065, EPI_ISL_2628069	Platform BIS UZA/UAntwerpen	Labo Klinische Biologie, UZA	Marie Le Mercier, Jasmine Coppens, Basil Britto Xavier, Christine Lammens, Veerle Matheeussen, Herman Goossens
EPI_ISL_2628717, EPI_ISL_2628825, EPI_ISL_2628962	UNILABS	Instituto Nacional de Saude (INSA)	Borges et al
EPI_ISL_2629224, EPI_ISL_2629232, EPI_ISL_2629233, EPI_ISL_2629234	CHC Andrée Rosemon	Institut Pasteur de la Guyane	Anne Lavergne, Dominique Rousset, Antoine Enfissi, Arielle Salmier
EPI_ISL_2629235, EPI_ISL_2629236, EPI_ISL_2629237	CNR Institut Pasteur de la Guyane	Institut Pasteur de la Guyane	Anne Lavergne, Dominique Rousset, Antoine Enfissi, Arielle Salmier
EPI_ISL_2631155	Oregon State Public Health Laboratory	Oregon State Public Health Laboratory	Rafia Razzaque, Eugene Yeboah, Vanda Makris, Laura Tsaknaridis, John Fontana and Shane Sevey
EPI_ISL_2631350, EPI_ISL_2631354, EPI_ISL_2631359, EPI_ISL_2631360, EPI_ISL_2631386, EPI_ISL_2631407	Pandemic Response Lab - NYC	Pandemic Response Lab, R&D	Henry Lee, Michael Hammerling, Melissa Hopkins, Cybill del Castillo, Shinyoung Clair Kang, William Ward, Pradeep Bugga, Sol Rey, Dylan Law, Katharine Nelson, Haiping Hao, Jon Laurent
EPI_ISL_2632831	MVZ Labor Dr. Limbach & Kollegen GbR	Robert Koch Institute	unknown
EPI_ISL_2633310	SYNLAB MVZ Leverkusen	Robert Koch Institute	unknown
EPI_ISL_2633321	SYNLAB MVZ Leinfelden-Echterdingen	Robert Koch Institute	unknown
EPI_ISL_2633914	Bioscientia MVZ Labor Karlsruhe GmbH	Robert Koch Institute	unknown
EPI_ISL_2634916, EPI_ISL_2634943, EPI_ISL_2634965	Pandemic Response Lab - NYC	Pandemic Response Lab, R&D	Henry Lee, Michael Hammerling, Melissa Hopkins, Cybill del Castillo, Shinyoung Clair Kang, William Ward, Pradeep Bugga, Sol Rey, Dylan Law, Katharine Nelson, Haiping Hao, Jon Laurent
EPI_ISL_2635200, EPI_ISL_2635291	SYNLAB MVZ Leverkusen	Robert Koch Institute	unknown
EPI_ISL_2635480	MVZ für Laboratoriumsmedizin und Mikrobiologie Würzburg	Robert Koch Institute	unknown
EPI_ISL_2635865, EPI_ISL_2635874, EPI_ISL_2635888	MVZ Labor Dr. Limbach & Kollegen GbR	Robert Koch Institute	unknown
EPI_ISL_2636711	Diagnosticum - Labor Neukirchen	Robert Koch Institute	unknown
EPI_ISL_2636815	Medizinisch-Diagnostisches Labor Kempten allgäulab	Robert Koch Institute	unknown
EPI_ISL_2637060, EPI_ISL_2637156, EPI_ISL_2637157	Bioscientia Labor Wermsdorf	Robert Koch Institute	unknown
EPI_ISL_2637186	Labor Dr. Schumacher MVZ	Robert Koch Institute	unknown
EPI_ISL_2637260	CLILAB	Microbiology Department	Sara Marti, Aida Gonzalez-Diaz, Laura Calatayud, Jordi Niubó, Miguel Fernandez-Huerta, Carmen Ardanuy, Jordi Camara, M Angeles Domínguez
EPI_ISL_2637324	Hospital Universitari Bellvitge	Microbiology Department	Sara Marti, Aida Gonzalez-Diaz, Laura Calatayud, Jordi Niubó, Miguel Fernandez-Huerta, Carmen Ardanuy, Jordi Camara, M Angeles Domínguez
EPI_ISL_2637629, EPI_ISL_2637761	Laboratoire LENYS	Department of Virology, Henri Mondor University Hospital, Assistance Publique Hôpitaux de Paris, Université Paris-Est Créteil, INSERM U955	Christophe Rodriguez, Slim Fourati, Vanessa Demontant, Guillaume Gricourt, Melissa N'Debi, Alexandre Soulier, Elisabeth Trawinski, Jean-Michel Pawlotsky
EPI_ISL_2638007	Biogroup Bio Lam-LCD Saint-Denis	Department of Virology, Henri Mondor University Hospital, Assistance Publique Hôpitaux de Paris, Université Paris-Est Créteil, INSERM U955	Christophe Rodriguez, Slim Fourati, Vanessa Demontant, Guillaume Gricourt, Melissa N'Debi, Alexandre Soulier, Elisabeth Trawinski, Jean-Michel Pawlotsky
EPI_ISL_2641746, EPI_ISL_2641751, EPI_ISL_2641754, EPI_ISL_2641771, EPI_ISL_2641775, EPI_ISL_2641782, EPI_ISL_2641789, EPI_ISL_2641793, EPI_ISL_2641794, EPI_ISL_2641800, EPI_ISL_2641816, EPI_ISL_2641817, EPI_ISL_2641818, EPI_ISL_2641820, EPI_ISL_2641824, EPI_ISL_2641829, EPI_ISL_2641835, EPI_ISL_2641862, EPI_ISL_2641865, EPI_ISL_2641868, EPI_ISL_2641884, EPI_ISL_2641889, EPI_ISL_2641906, EPI_ISL_2641907, EPI_ISL_2641926, EPI_ISL_2641938, EPI_ISL_2641973, EPI_ISL_2641984, EPI_ISL_2642007, EPI_ISL_2642011, EPI_ISL_2642029, EPI_ISL_2642040			
see above	Istituto Zooprofilattico Sperimentale del Mezzogiorno	Telethon Institute of Genetics and Medicine (TIGEM)	Antonio Grimaldi Patrizia Annunziata Francesco Panariello Biancamaria Pierri Claudia Tiberio Teresa Giuliano Valentina Bouche Chiara Colantuono Maria Concetta Cuomo Denise Di Concilio Lucio Di Filippo Anna Manfredi Marcello Salvi Antonio Limone Luigi Atripaldi Pellegrino Cerino Andrea Ballabio Davide Cacchiarelli
EPI_ISL_2645408, EPI_ISL_2645409, EPI_ISL_2645410	Laboratorio Central de Saude Publica do Estado da Bahia (LACEN/BA)	Laboratory of Respiratory Viruses and Measles, Oswaldo Cruz Institute, FIOCRUZ	Paola Resende, Luciana Appolinario, Fernando Motta, Anna Carolina Paixao, Ana Carolina Mendonca, Alice Sampaio Rocha, Taina Venas, Elisa Cavalcante Pereira, Renata Serrano Lopes,Felicidade Pereira, Marilda Siqueira on behalf of the Fiocruz COVID-19 Genomic Surveillance Network
EPI_ISL_2645995, EPI_ISL_2646099	UNILABS	Instituto Nacional de Saude (INSA)	Borges et al
EPI_ISL_2646210, EPI_ISL_2646212	CH Valenciennes	CHU Lille - Laboratoire de Virologie	AIT YAHYA Emilie, ALIDJINOU Enagnon Kazali, BOCKET Laurence, CREPIN Michel, DEMAY Christophe, ENGELMANN Ilka, GEFFROY Sandrine, GUIGON Aurélie, LAMBERT Valérie, LAZREK Mouna, NOBILLIAUX Florian, PREVOST Brigitte, THUILLIER Caroline, TINEZ Claire ,TCHANTCHOU NJOSSE YANICK
EPI_ISL_2646337, EPI_ISL_2646340	MS Public Health Laboratory	University of Mississippi Medical Center, Molecular and Genomics Core Facility	Ashley C. Johnson, Wenjie Wu, Ithiel J. Frame, Krishna K. Ayyalasomayajula, Michael R. Garrett, D. Ashley Robinson
EPI_ISL_2649831	NJDOH, Public Health and Environmental Laboratories	NJ_PHEL	Lindsey Bodnar, Shiv K. Verma, Ryan Pachucki, Chelsea San Filippo, Jacquelyn Deverell, Dana Woell, Allison Roder, Byeong Jeong

EPI_ISL_2649965, EPI_ISL_2649966, EPI_ISL_2649967, EPI_ISL_2649968, EPI_ISL_2649969, EPI_ISL_2649976, EPI_ISL_2649978, EPI_ISL_2649979, EPI_ISL_2649980, EPI_ISL_2649981, EPI_ISL_2649982, EPI_ISL_2649984, EPI_ISL_2649986, EPI_ISL_2649987, EPI_ISL_2649988, EPI_ISL_2649989, EPI_ISL_2649990			
see above	Quest Diagnostics Incorporated	Centers for Disease Control and Prevention Division of Viral Diseases, Pathogen Discovery	Dakota Howard, Dhwani Batra, Peter W. Cook, Kara Moser, Adrian Paskey, Jason Caravas, Benjamin Rambo-Martin, Shatavia Morrison, Christopher Gulvick, Scott Sammons, Yvette Unoarumhi, Darlene Wagner, Matthew Schmerer, S. H. Rosenthal, A. Gerasimova, R. M. Kagan, B. Anderson, M. Hua, Y. Liu, L.E. Bernstein, K.E. Livingston, A. Perez, I. A. Shlyakhter, R. V. Rolando, R. Owen, P. Tanpaiboon, F. Lacbawan, Clinton R. Paden, Duncan MacCannell
EPI_ISL_2650136, EPI_ISL_2650148	Arizona State Public Health Laboratory	Arizona State Public Health Laboratory	Trung Huynh, Jessica Escobar, Katherine Fullerton, Nobuko Fukushima, Matthew Contursi, Stacy White, Linda Getsinger, Victor Waddell
EPI_ISL_2651062, EPI_ISL_2651066, EPI_ISL_2651067, EPI_ISL_2651083, EPI_ISL_2651084, EPI_ISL_2651086, EPI_ISL_2651097, EPI_ISL_2651105	Washington State Department of Health Public Health Laboratories	Washington State Department of Health Public Health Laboratories	Drew MacKellar, Philip Dykema, Denny Russell, Joenice Gonzalez, Hannah Gray, Geoff Melly, Vanessa De Los Santos, Darren Lucas, JohnAric Peterson, Avi Singh, Rebecca Cao
EPI_ISL_2652076	New Mexico Department of Health Scientific Laboratory	New Mexico Department of Health Scientific Laboratory	Ellie Johnson, D'eldra Malone, Jennifer Benoit, Ratheesh Rajan, Linda Salazar, Keila Gutierrez, Anastacia Griego-Fisher
EPI_ISL_2652157	AZ Klina	AZ Klina	Carl Vael - Lynsey Berckmans
EPI_ISL_2653015, EPI_ISL_2653035, EPI_ISL_2653056, EPI_ISL_2653059	Infinity Biologix	Centers for Disease Control and Prevention Division of Viral Diseases, Pathogen Discovery	Dakota Howard, Dhwani Batra, Peter W. Cook, Kara Moser, Adrian Paskey, Jason Caravas, Benjamin Rambo-Martin, Shatavia Morrison, Christopher Gulvick, Scott Sammons, Yvette Unoarumhi, Darlene Wagner, Matthew Schmerer, Christian Bixby, Yihe Wang, Jonathan Schultz, Chirayu Goswami, Russ Hager, Robin Grimwood, Clinton R. Paden, Duncan MacCannell
EPI_ISL_2658950	Basurto University Hospital: Clinical Microbiology Laboratory	Biocruces Bizkaia	Mikel Urrutikoetxea-Gutiérrez, Mª Carmen Nieto Toboso, Estibaliz Ugalde Zarraga, Mikel Gallego Rodrigo, Ana de la Hoz, José Luis Díaz de Tuesta del Arco
EPI_ISL_2658982	County of San Luis Obispo Public Health Laboratory	County of San Luis Obispo Public Health Laboratory	Katrina Erwin, Frances Sidhu
EPI_ISL_2659142, EPI_ISL_2659174, EPI_ISL_2659193	Genetica Molecular and Subdepartamento de Virologia ISP Chile	Instituto de Salud Publica de Chile	Karen Orostica, Constanza Campano, Barbara Parra, Loredana Arata, Gisselle Barra, Patricia Bustos, Rodrigo Fasce, Javier Tognarelli, Andres Castillo, Soledad Ulloa, Jorge Fernandez
EPI_ISL_2661538, EPI_ISL_2661546, EPI_ISL_2661547, EPI_ISL_2661548, EPI_ISL_2661549	UHTL, University Hospitals	UHTL, University Hospitals	Sadri,N., Alouani,D., Song,X.
EPI_ISL_2662403	Viollier AG	Department of Biosystems Science and Engineering, ETH Zürich	Andrea Patrizia Salzmann, Henriette Kurth, Christiane Beckmann, Ivan Topolsky, Chaoran Chen, Sarah Nadeau, Kim Philipp Jablonski, Lara Fuhrmann, Niko Beerenwinkel, Christoph Noppen, Olivier Kobel, Maurice Redondo, Tanja Stadler
EPI_ISL_2662776	DPHL	Delaware Public Health Lab	Rebecca Savage
EPI_ISL_2663225	HealthPartners Central Lab	Minnesota Department of Health, Public Health Laboratory	Alexandra Lorentz, Jacob Garfin, Matt Plumb, and Xiong Wang
EPI_ISL_2663232, EPI_ISL_2663238	Mayo Clinic & Mayo Clinic Laboratories	Minnesota Department of Health, Public Health Laboratory	Alexandra Lorentz, Jacob Garfin, Matt Plumb, and Xiong Wang
EPI_ISL_2665775, EPI_ISL_2667603, EPI_ISL_2669458, EPI_ISL_2670239, EPI_ISL_2671258	Department of Bacteria, Parasites and Fungi, Statens Serum Institut, Copenhagen, Denmark	Statens Serum Institut Bioinformatics and Microbial Genomics	Danish Covid-19 Genome Consortium
EPI_ISL_2671459, EPI_ISL_2671469	Center for Laboratory Medicine	Center for Laboratory Medicine	Yannick Gerth
EPI_ISL_2672303, EPI_ISL_2672626, EPI_ISL_2672638, EPI_ISL_2672650, EPI_ISL_2672760, EPI_ISL_2672934, EPI_ISL_2672978, EPI_ISL_2673074, EPI_ISL_2673075, EPI_ISL_2673093, EPI_ISL_2673192, EPI_ISL_2673214, EPI_ISL_2673225, EPI_ISL_2673243, EPI_ISL_2673244, EPI_ISL_2673275			
see above	Dutch COVID-19 response team	National Institute for Public Health and the Environment (RIVM)	Adam Meijer, Harry Vennema, Dirk Eggink, Jeroen Cremer, Sharon van den Brink, Bas van der Veer, AnneMarie van den Brandt, Lisa Wijsman, Kim Freriks, Ryanne Jaarsma, Eunice Then, Lynn Aarts, Sanne Bos, Melissa van Tuil, Linda van de Nes, Florian Zwagemaker, Dennis Schmitz, Annelies Kroneman, Karim Hajji, Chantal Reusken, on behalf of the national COVID-19 response team
EPI_ISL_2674636, EPI_ISL_2674637, EPI_ISL_2674638, EPI_ISL_2674639, EPI_ISL_2674640	LESP Tabasco	Instituto de Diagnostico y Referencia Epidemiologicos (INDRE)	Claudia Wong-Arambula, Abril Rodriguez-Maldonado, Vanessa Rivero-Arredondo, Ariadna Medina-Benitez, Joaquin Quiroz-Mercado, Sergio Rangel-Guerrero, Natividad Cruz-Ortiz, Tatiana Nunez-Garcia, Gisela Barrera-Badillo, Lucia Hernandez-Rivas, Irma Lopez-Martinez, Ernesto Ramirez-Gonzalez.
EPI_ISL_2674644, EPI_ISL_2674645, EPI_ISL_2674646, EPI_ISL_2674647, EPI_ISL_2674648, EPI_ISL_2674649	LESP Sonora	Instituto de Diagnostico y Referencia Epidemiologicos (INDRE)	Claudia Wong-Arambula, Abril Rodriguez-Maldonado, Vanessa Rivero-Arredondo, Ariadna Medina-Benitez, Joaquin Quiroz-Mercado, Sergio Rangel-Guerrero, Natividad Cruz-Ortiz, Tatiana Nunez-Garcia, Gisela Barrera-Badillo, Lucia Hernandez-Rivas, Irma Lopez-Martinez, Ernesto Ramirez-Gonzalez.
EPI_ISL_2674650	LESP Jalisco	Instituto de Diagnostico y Referencia Epidemiologicos (INDRE)	Claudia Wong-Arambula, Abril Rodriguez-Maldonado, Vanessa Rivero-Arredondo, Ariadna Medina-Benitez, Joaquin Quiroz-Mercado, Sergio Rangel-Guerrero, Natividad Cruz-Ortiz, Tatiana Nunez-Garcia, Gisela Barrera-Badillo, Lucia Hernandez-Rivas, Irma Lopez-Martinez, Ernesto Ramirez-Gonzalez.
EPI_ISL_2674674, EPI_ISL_2674675, EPI_ISL_2674676, EPI_ISL_2674677, EPI_ISL_2674678, EPI_ISL_2674679, EPI_ISL_2674680, EPI_ISL_2674681, EPI_ISL_2674682	LESP Hidalgo	Instituto de Diagnostico y Referencia Epidemiologicos (INDRE)	Claudia Wong-Arambula, Abril Rodriguez-Maldonado, Vanessa Rivero-Arredondo, Ariadna Medina-Benitez, Joaquin Quiroz-Mercado, Sergio Rangel-Guerrero, Natividad Cruz-Ortiz, Tatiana Nunez-Garcia, Gisela Barrera-Badillo, Lucia Hernandez-Rivas, Irma Lopez-Martinez, Ernesto Ramirez-Gonzalez.
EPI_ISL_2674683	LESP Queretaro	Instituto de Diagnostico y Referencia Epidemiologicos (INDRE)	Claudia Wong-Arambula, Abril Rodriguez-Maldonado, Vanessa Rivero-Arredondo, Ariadna Medina-Benitez, Joaquin Quiroz-Mercado, Sergio Rangel-Guerrero, Natividad Cruz-Ortiz, Tatiana Nunez-Garcia, Gisela Barrera-Badillo, Lucia Hernandez-Rivas, Irma Lopez-Martinez, Ernesto Ramirez-Gonzalez.
EPI_ISL_2674689	LESP Coahuila	Instituto de Diagnostico y Referencia Epidemiologicos (INDRE)	Claudia Wong-Arambula, Abril Rodriguez-Maldonado, Vanessa Rivero-Arredondo, Ariadna Medina-Benitez, Joaquin Quiroz-Mercado, Sergio Rangel-Guerrero, Natividad Cruz-Ortiz, Tatiana Nunez-Garcia, Gisela Barrera-Badillo, Lucia Hernandez-Rivas, Irma Lopez-Martinez, Ernesto Ramirez-Gonzalez.
EPI_ISL_2674690	LESP Hidalgo	Instituto de Diagnostico y Referencia Epidemiologicos (INDRE)	Claudia Wong-Arambula, Abril Rodriguez-Maldonado, Vanessa Rivero-Arredondo, Ariadna Medina-Benitez, Joaquin Quiroz-Mercado, Sergio Rangel-Guerrero, Natividad Cruz-Ortiz, Tatiana Nunez-Garcia, Gisela Barrera-Badillo, Lucia Hernandez-Rivas, Irma Lopez-Martinez, Ernesto Ramirez-Gonzalez.
EPI_ISL_2674695, EPI_ISL_2674696	LESP Guanajuato	Instituto de Diagnostico y Referencia Epidemiologicos (INDRE)	Claudia Wong-Arambula, Abril Rodriguez-Maldonado, Vanessa Rivero-Arredondo, Ariadna Medina-Benitez, Joaquin Quiroz-Mercado, Sergio Rangel-Guerrero, Natividad Cruz-Ortiz, Tatiana Nunez-Garcia, Gisela Barrera-Badillo, Lucia Hernandez-Rivas, Irma Lopez-Martinez, Ernesto Ramirez-Gonzalez.
EPI_ISL_2674698	LESP Ciudad de Mexico	Instituto de Diagnostico y Referencia Epidemiologicos (INDRE)	Claudia Wong-Arambula, Abril Rodriguez-Maldonado, Vanessa Rivero-Arredondo, Ariadna Medina-Benitez, Joaquin Quiroz-Mercado, Sergio Rangel-Guerrero, Natividad Cruz-Ortiz, Tatiana Nunez-Garcia, Gisela Barrera-Badillo, Lucia Hernandez-Rivas, Irma Lopez-Martinez, Ernesto Ramirez-Gonzalez.
EPI_ISL_2674700	LESP Puebla	Instituto de Diagnostico y Referencia Epidemiologicos (INDRE)	Claudia Wong-Arambula, Abril Rodriguez-Maldonado, Vanessa Rivero-Arredondo, Ariadna Medina-Benitez, Joaquin Quiroz-Mercado, Sergio Rangel-Guerrero, Natividad Cruz-Ortiz, Tatiana Nunez-Garcia, Gisela Barrera-Badillo, Lucia Hernandez-Rivas, Irma Lopez-Martinez, Ernesto Ramirez-Gonzalez.
EPI_ISL_2674703, EPI_ISL_2674704, EPI_ISL_2674705, EPI_ISL_2674706, EPI_ISL_2674707, EPI_ISL_2674708, EPI_ISL_2674709, EPI_ISL_2674710, EPI_ISL_2674711, EPI_ISL_2674712, EPI_ISL_2674713			
see above	LESP Yucatan	Instituto de Diagnostico y Referencia Epidemiologicos (INDRE)	Claudia Wong-Arambula, Abril Rodriguez-Maldonado, Vanessa Rivero-Arredondo, Ariadna Medina-Benitez, Joaquin Quiroz-Mercado, Sergio Rangel-Guerrero, Natividad Cruz-Ortiz, Tatiana Nunez-Garcia, Gisela Barrera-Badillo, Lucia Hernandez-Rivas, Irma Lopez-Martinez, Ernesto Ramirez-Gonzalez.

EPI_ISL_2674714	LESP Quintana Roo	Instituto de Diagnostico y Referencia Epidemiologicos (INDRE)	Claudia Wong-Arambula, Abril Rodriguez-Maldonado, Vanessa Rivero-Arredondo, Ariadna Medina-Benitez, Joaquin Quiroz-Mercado, Sergio Rangel-Guerrero, Natividad Cruz-Ortiz, Tatiana Nunez-Garcia, Gisela Barrera-Badillo, Lucia Hernandez-Rivas, Irma Lopez-Martinez, Ernesto Ramirez-Gonzalez.
EPI_ISL_2674715	LESP Yucatan	Instituto de Diagnostico y Referencia Epidemiologicos (INDRE)	Claudia Wong-Arambula, Abril Rodriguez-Maldonado, Vanessa Rivero-Arredondo, Ariadna Medina-Benitez, Joaquin Quiroz-Mercado, Sergio Rangel-Guerrero, Natividad Cruz-Ortiz, Tatiana Nunez-Garcia, Gisela Barrera-Badillo, Lucia Hernandez-Rivas, Irma Lopez-Martinez, Ernesto Ramirez-Gonzalez.
EPI_ISL_2674718, EPI_ISL_2674719	LESP Sonora	Instituto de Diagnostico y Referencia Epidemiologicos (INDRE)	Claudia Wong-Arambula, Abril Rodriguez-Maldonado, Vanessa Rivero-Arredondo, Ariadna Medina-Benitez, Joaquin Quiroz-Mercado, Sergio Rangel-Guerrero, Natividad Cruz-Ortiz, Tatiana Nunez-Garcia, Gisela Barrera-Badillo, Lucia Hernandez-Rivas, Irma Lopez-Martinez, Ernesto Ramirez-Gonzalez.
EPI_ISL_2676629	DYOMEDEA-LABORATOIRE DE LA SAUVEGARDE	CNR Virus des Infections Respiratoires - France SUD	Antonin Bal, Gregory Destras, Gwendolyne Burfin, Hadrien Regue, Quentin Semanas, Martine Valette, Bruno Lina, Laurence Josset
EPI_ISL_2678714	MVZ Labor Dr. Limbach & Kollegen GbR	Robert Koch Institute	unknown
EPI_ISL_2680902, EPI_ISL_2680903, EPI_ISL_2680904, EPI_ISL_2680905, EPI_ISL_2680906, EPI_ISL_2680907, EPI_ISL_2680908, EPI_ISL_2680909, EPI_ISL_2680910, EPI_ISL_2680911, EPI_ISL_2680912, EPI_ISL_2680914, EPI_ISL_2680915			
see above	HLAGYN - Laboratorio de Imunologia de Transplantes de Goias	HLAGYN - Laboratorio de Imunologia de Transplantes de Goias	Fernando Antonio Vinhal dos Santos, Erika Lopes Rocha Batista, Alessandro Leonardo Alvares Magalhaes, Frederico Rodrigues Vinhal, Sabrina Sara Moreira Duarte, Lucas Carlos Gomes Pereira, Daniel Ferreira de Sousa, Danielle de Paiva Rezende
EPI_ISL_2681091, EPI_ISL_2681109	Unidad de Investigación Médica de Yucatán (UIMY)	Instituto de Biotecnología de la UNAM	Consorcio Mexicano de Vigilancia Genómica (CoViGen-Mex). Authors (in alphabetical order): Julio Elias Alvarado-Yaah, Carlos F. Arias, Santiago Ávila-Ríos, Eduardo Becerril-Vargas, Víctor Hugo Borja-Aburto, Celia Boukadida, Cristóbal Cháidez-Quiróz, Juan Bautista Chale-Dzul, Ricardo Ciria Merce, Andreu Comas-García, Célida Duque Molina, Julissa Enciso-Ibarra, José Antonio Enciso-Moreno, Gloria Elena Espinosa-Ayala, Fernando Fontove-Herrera, Daniel Fregoso-Rueda, Victor Eduardo García-Arias, Alejandra García-Gasca, Bruno Gómez-Gil, Jean Pierre González, Irvin González-López, Concepción Grajales-Muñiz, Ricardo Grande, Rosa María Gutiérrez Rios, Jesús Hernández, Alejandra Hernández-Terán, Alfredo Herrera-Estrella, Carla Ivón Herrera-Najera, Pavel Isa, Verónica Mata-Haro, Daniel Lira Morales, Susana Lopez, Antonio Loza Román, Brenda Irasema Maldonado-Meza, Bernardo Martínez-Miguel, José Arturo Martínez-Orozco, Célida Martínez- Rodríguez, Margarita Matías-Florentino, Fidencio Mejía-Nepomuceno, María Guadalupe de Jesús Mireles-Rivera, Gloria María Molina-Salinas, Hector Montoya-Fuentes, , Mario Mújica-Sánchez, José Esteban Muñoz-Medina, José de Jesús Nuñez-Contreras, Alicia Ocaña-Mondragón, Luis Alberto Ochoa-Carrera, Hector Esteban Paz-Juárez, Marissa Perez-Garcia, Francisco Pulido, Helen Haydee Fernanda Ramírez-Plascencia, Jorge Salas-Hernández, Angel Gustavo Salas-Lais, Alejandro Sánchez-Flores, Clara Esperanza Santacruz-Tinoco, María Guadalupe Santiago-Mauricio, Selene Zárate, Nelly Sélem-Mojica, Blanca Taboada, Gloria Vazquez, Joel Armando Vázquez-Pérez.
EPI_ISL_2681457	University Hospitals of Geneva, Laboratory of Virology	HUG, Laboratory of Virology and the Health2030 Genome Center	Samuel Cordey, Ana Rita Goncalves, Laurent Kaiser, Lorenzo Cerutti, Henri Pegeot, Melyssa Elies, Deborah Penet, Keith Harshman, Ioannis Xenarios, Emmanouil Dermitzakis
EPI_ISL_2685850, EPI_ISL_2685878	SYNLAB	GIGA Medical Genomics	Keith Durkin, Maria Artesi, Sébastien Bontems, Raphaël Boreux, Bouchra Boujemla, Nathalie Renotte, Cécile Meex, Pierrette Melin, Marie-Pierre Hayette, Vincent Bours
EPI_ISL_2685890, EPI_ISL_2685891	Department of Clinical Microbiology	GIGA Medical Genomics	Keith Durkin, Maria Artesi, Sébastien Bontems, Raphaël Boreux, Bouchra Boujemla, Nathalie Renotte, Cécile Meex, Pierrette Melin, Marie-Pierre Hayette, Vincent Bours
EPI_ISL_2686065	Azienda USL Umbria 2	Istituto Zooprofilattico Sperimentale dell'Abruzzo e Molise "G. Caporale"	Proietti A, Pistoni E, Lorusso A, Marcacci M, Di Domenico M, Ancora M, Curini V, Mangone I, Rinaldi A, Delli Compagni E, Scialabba S, Di Pasquale A, Cammà C, Puglia I, Calistri P, Savini G
EPI_ISL_2686359, EPI_ISL_2686360, EPI_ISL_2686361, EPI_ISL_2686362, EPI_ISL_2686363, EPI_ISL_2686364, EPI_ISL_2686365, EPI_ISL_2686366, EPI_ISL_2686367, EPI_ISL_2686368, EPI_ISL_2686369, EPI_ISL_2686370, EPI_ISL_2686371, EPI_ISL_2686372, EPI_ISL_2686373, EPI_ISL_2686374, EPI_ISL_2686375, EPI_ISL_2686376, EPI_ISL_2686377, EPI_ISL_2686378, EPI_ISL_2686379, EPI_ISL_2686380, EPI_ISL_2686381, EPI_ISL_2686382, EPI_ISL_2686383, EPI_ISL_2686384, EPI_ISL_2686386, EPI_ISL_2686387, EPI_ISL_2686388			
see above	Quest Diagnostics Incorporated	Centers for Disease Control and Prevention Division of Viral Diseases, Pathogen Discovery	Dakota Howard, Dhwani Batra, Peter W. Cook, Kara Moser, Adrian Paskey, Jason Caravas, Benjamin Rambo-Martin, Shatavia Morrison, Christopher Gulvick, Scott Sammons, Yvette Unoarumhi, Darlene Wagner, Matthew Schmerer, S. H. Rosenthal, A. Gerasimova, R. M. Kagan, B. Anderson, M. Hua, Y. Liu, L.E. Bernstein, K.E. Livingston, A. Perez, I. A. Shlyakhter, R. V. Rolando, R. Owen, P. Tanpaiboon, F. Lacbawan, Clinton R. Paden, Duncan MacCannell
EPI_ISL_2686408, EPI_ISL_2686431, EPI_ISL_2686436, EPI_ISL_2686449, EPI_ISL_2686452, EPI_ISL_2686552, EPI_ISL_2686554, EPI_ISL_2686555, EPI_ISL_2686561, EPI_ISL_2686573, EPI_ISL_2686609, EPI_ISL_2686623, EPI_ISL_2686632, EPI_ISL_2686651, EPI_ISL_2686717, EPI_ISL_2686720, EPI_ISL_2686732, EPI_ISL_2686733, EPI_ISL_2686762, EPI_ISL_2686764, EPI_ISL_2686777, EPI_ISL_2686790, EPI_ISL_2686793, EPI_ISL_2686795, EPI_ISL_2686797, EPI_ISL_2686799			
see above	Fulgent Genetics	Centers for Disease Control and Prevention Division of Viral Diseases, Pathogen Discovery	Dakota Howard, Dhwani Batra, Peter W. Cook, Kara Moser, Adrian Paskey, Jason Caravas, Benjamin Rambo-Martin, Shatavia Morrison, Christopher Gulvick, Scott Sammons, Yvette Unoarumhi, Darlene Wagner, Matthew Schmerer, Harry Gao, Mickey Li, John Gao, Joseph Fierro, Benafsh Sapra, Becky Tsai, Yan Meng, Doreen Ng, James Xie, Clinton R. Paden, Duncan MacCannell
EPI_ISL_2686856, EPI_ISL_2686857, EPI_ISL_2686880, EPI_ISL_2686883, EPI_ISL_2686889	Infinity Biologix	Centers for Disease Control and Prevention Division of Viral Diseases, Pathogen Discovery	Dakota Howard, Dhwani Batra, Peter W. Cook, Kara Moser, Adrian Paskey, Jason Caravas, Benjamin Rambo-Martin, Shatavia Morrison, Christopher Gulvick, Scott Sammons, Yvette Unoarumhi, Darlene Wagner, Matthew Schmerer, Christian Bixby, Yihe Wang, Jonathan Schultz, Chirayu Goswami, Russ Hager, Robin Grimwood, Clinton R. Paden, Duncan MacCannell
EPI_ISL_2686906, EPI_ISL_2686908, EPI_ISL_2686911, EPI_ISL_2686917, EPI_ISL_2686919, EPI_ISL_2686920, EPI_ISL_2686921, EPI_ISL_2686950, EPI_ISL_2686962, EPI_ISL_2686974, EPI_ISL_2686981, EPI_ISL_2686982, EPI_ISL_2686984, EPI_ISL_2686985, EPI_ISL_2686989, EPI_ISL_2686997, EPI_ISL_2687015, EPI_ISL_2687021, EPI_ISL_2687034, EPI_ISL_2687035, EPI_ISL_2687046, EPI_ISL_2687047, EPI_ISL_2687057, EPI_ISL_2687058, EPI_ISL_2687063, EPI_ISL_2687094, EPI_ISL_2687095, EPI_ISL_2687096, EPI_ISL_2687098, EPI_ISL_2687120, EPI_ISL_2687121, EPI_ISL_2687136, EPI_ISL_2687162, EPI_ISL_2687165, EPI_ISL_2687179, EPI_ISL_2687186, EPI_ISL_2687196, EPI_ISL_2687202, EPI_ISL_2687205, EPI_ISL_2687213, EPI_ISL_2687242, EPI_ISL_2687243, EPI_ISL_2687248, EPI_ISL_2687253, EPI_ISL_2687265, EPI_ISL_2687276, EPI_ISL_2687287, EPI_ISL_2687292, EPI_ISL_2687301, EPI_ISL_2687303, EPI_ISL_2687308, EPI_ISL_2687321, EPI_ISL_2687326, EPI_ISL_2687378, EPI_ISL_2687380, EPI_ISL_2687381, EPI_ISL_2687388, EPI_ISL_2687403, EPI_ISL_2687407, EPI_ISL_2687408, EPI_ISL_2687413, EPI_ISL_2687416, EPI_ISL_2687419, EPI_ISL_2687444, EPI_ISL_2687455, EPI_ISL_2687488, EPI_ISL_2687489, EPI_ISL_2687499, EPI_ISL_2687503, EPI_ISL_2687551, EPI_ISL_2687554, EPI_ISL_2687558, EPI_ISL_2687559			
see above	Aegis Sciences Corporation	Centers for Disease Control and Prevention Division of Viral Diseases, Pathogen Discovery	Dakota Howard, Dhwani Batra, Peter W. Cook, Kara Moser, Adrian Paskey, Jason Caravas, Benjamin Rambo-Martin, Shatavia Morrison, Christopher Gulvick, Scott Sammons, Yvette Unoarumhi, Darlene Wagner, Matthew Schmerer, Cyndi Clark, Patrick Campbell, Rob Case, Vikramsinha Ghorpade, Holly Houdeshell, Ola Kvalvaag, Dillon Nall, Ethan Sanders, Alec Vest, Shaun Westlund, Matthew Hardison, Clinton R. Paden, Duncan MacCannell
EPI_ISL_2688819	Massachusetts State Public Health Laboratory	Massachusetts State Public Health Laboratory	Andrew Lang, Timelia Fink, Glen Gallagher, Sandra Smole
EPI_ISL_2689682	Nebraska Public Health Laboratory	NPHL COVID-19 Response Team	NPHL COVID-19 Response Team
EPI_ISL_2690127	MT Public Health Laboratory	Centers for Disease Control and Prevention Division of Viral Diseases, Pathogen Discovery	Mili Sheth, Sarah Nobles, Jasmine Padilla, Mark Burroughs, Shoshona Le, Katie Dillon, Peter Cook, Clinton R. Paden, Dhwani Batra, Krista Queen, Kristen Knipe, Dakota Howard, Yvette Unoarumhi, Darlene Wagner, Matthew Schmerer, Ben L. Rambo-Martin, Kristine Lacek, Sam Shepard, Alison Laufer Halpin, Dave Wentworth, Vivien Dugan, Suxiang Tong, Justin Lee
EPI_ISL_2690251	University of Mississippi Medical Center, Department of Pathology	University of Mississippi Medical Center, Molecular and Genomics Core Facility	Ashley C. Johnson, Wenjie Wu, Ithiel J. Frame, Krishna K. Ayyalasomayajula, Michael R. Garrett, D. Ashley Robinson
EPI_ISL_2690272	Nevada State Public Health Laboratory	Nevada State Public Health Laboratory	Andrew Gorzalski, Mark Pandori
EPI_ISL_2691501, EPI_ISL_2691502, EPI_ISL_2691503, EPI_ISL_2691504, EPI_ISL_2691511, EPI_ISL_2691512, EPI_ISL_2691517, EPI_ISL_2691518, EPI_ISL_2691520, EPI_ISL_2691521, EPI_ISL_2691522, EPI_ISL_2691525, EPI_ISL_2691533, EPI_ISL_2691547, EPI_ISL_2691548, EPI_ISL_2691549, EPI_ISL_2691550, EPI_ISL_2691551, EPI_ISL_2691552, EPI_ISL_2691568			
see above	Laboratorio Central Noel Nutels	Bioinformatics Laboratory / LNCC	Luiz G P de Almeida, Alessandra P Lamarca, Ronaldo da Silva F Jr, Liliane Cavalcante, Alexandra L Gerber, Ana Paula de C Guimaraes, Douglas Terra Machado, Cassia Alves, Diana Mariani, Cintia Policarpo, Gleidson da Silva de Oliveira, Mario Sergio Ribeiro, Silvia Carvalho, Flavio Dias da Silva, Marcio Henrique de Oliveira Garcia, Leandro Magalhaes de Souza, Cristiane Gomes da Silva, Caio Luiz Pereira Ribeiro, Andrea Cony Cavalcanti, Claudia Maria Braga de Mello, Amilcar Tanuri, Ana Tereza R Vasconcelos
EPI_ISL_2691573	Unidade de apoio ao diagnostico da COVID - UNADIG	Bioinformatics Laboratory / LNCC	Luiz G P de Almeida, Alessandra P Lamarca, Ronaldo da Silva F Jr, Liliane Cavalcante, Alexandra L Gerber, Ana Paula de C Guimaraes, Douglas Terra Machado, Cassia Alves, Diana Mariani, Cintia Policarpo, Gleidson da Silva de Oliveira, Mario Sergio Ribeiro, Silvia Carvalho, Flavio Dias da Silva, Marcio Henrique de Oliveira Garcia, Leandro Magalhaes de Souza, Cristiane Gomes da Silva, Caio Luiz Pereira Ribeiro, Andrea Cony Cavalcanti, Claudia Maria Braga de Mello, Amilcar Tanuri, Ana Tereza R Vasconcelos

EPI_ISL_2692541, EPI_ISL_2692544, EPI_ISL_2692549, EPI_ISL_2692552, EPI_ISL_2692554, EPI_ISL_2692556, EPI_ISL_2692560, EPI_ISL_2692562, EPI_ISL_2692564, EPI_ISL_2692565, EPI_ISL_2692566, EPI_ISL_2692572, EPI_ISL_2692580, EPI_ISL_2692584, EPI_ISL_2692587, EPI_ISL_2692590, EPI_ISL_2692593, EPI_ISL_2692614, EPI_ISL_2692619, EPI_ISL_2692622, EPI_ISL_2692626, EPI_ISL_2692629, EPI_ISL_2692632, EPI_ISL_2692634, EPI_ISL_2692638, EPI_ISL_2692643, EPI_ISL_2692644, EPI_ISL_2692648, EPI_ISL_2692653, EPI_ISL_2692654, EPI_ISL_2692658, EPI_ISL_2692659, EPI_ISL_2692661, EPI_ISL_2692667, EPI_ISL_2692669, EPI_ISL_2692670, EPI_ISL_2692673, EPI_ISL_2692677, EPI_ISL_2692678, EPI_ISL_2692680, EPI_ISL_2692685, EPI_ISL_2692690, EPI_ISL_2692692, EPI_ISL_2692699			
see above	Salud Digna	Instituto Nacional de Medicina Genomica	Cedro-Tanda A, Hidalgo-Miranda A, Mendoza-Vargas A, Reyes-Grajeda JP, Abraham Campos-Romero, Moreno-Camacho José Luis, Rodriguez-Gallegos Jorge, Luna-Ruiz Marco, Gonzalez-Barrera D, Rangel-DeLeon D, Munguia-Garza P, Ramirez-Vega O, Escobar-Arrazola, M, Herrera-Montalvo LA.
EPI_ISL_2693129, EPI_ISL_2693139, EPI_ISL_2693277, EPI_ISL_2693278, EPI_ISL_2693299, EPI_ISL_2693301, EPI_ISL_2693307, EPI_ISL_2693310, EPI_ISL_2693312	UW Virology Lab	UW Virology Lab	Pavitra Roychoudhury, Hong Xie, Lasata Shrestha, Tien V. Nguyen, Shah Mohamed Bakhash, Michelle Lin, Noah R. Baker, Ricardo Perez, Sean Ellis, Nathan Breit, Robert J. Livingston, Meei-Li Huang, Keith R Jerome, Patrick Mathias, Alexander Greninger
EPI_ISL_2693684, EPI_ISL_2693739, EPI_ISL_2693746, EPI_ISL_2693753, EPI_ISL_2693763, EPI_ISL_2693801, EPI_ISL_2693813	UW Virology Lab	UW Virology Lab	Pavitra Roychoudhury, Hong Xie, Lasata Shrestha, Shah Mohamed Bakhash, Tien V. Nguyen, Noah R. Baker, Sean Ellis, Meei-Li Huang, Keith R Jerome, Alexander Greninger
EPI_ISL_2694805, EPI_ISL_2694858	Labor Berlin Charite Vivantes GmbH / Institut fur Virologie	Charite Universitatsmedizin Berlin, Institut fur Virologie/Labor Berlin	Peter Menzel, Christine Stephan, Rolf Schwarzer, Victor M Corman, Barbara Muhlemann, Terry Jones, Christian Drosten
EPI_ISL_2695098	CHULN - H Santa Maria	Instituto Nacional de Saude (INSA)	Borges et al
EPI_ISL_2695275	H Braga	Instituto Nacional de Saude (INSA)	Borges et al
EPI_ISL_2695325, EPI_ISL_2695326	ULSM - Matosinhos	Instituto Nacional de Saude (INSA)	Borges et al
EPI_ISL_2695345	CHEDV	Instituto Nacional de Saude (INSA)	Borges et al
EPI_ISL_2695390, EPI_ISL_2695391	CH Tondela Viseu	Instituto Nacional de Saude (INSA)	Borges et al
EPI_ISL_2695394	Lab La Salete Robles - VN Famalicao	Instituto Nacional de Saude (INSA)	Borges et al
EPI_ISL_2695447	ARS Algarve - Laboratorio Laura Ayres	Instituto Nacional de Saude (INSA)	Borges et al
EPI_ISL_2709012	UMC Groningen, Clinical Virology, Department of Medical Microbiology and Infection Prevention	UMC Groningen, Clinical Virology, Department of Medical Microbiology and Infection Prevention	Hubert Niesters, Alexander Friedrich, Erley Lizarazo-Forero, Monika Fliss, Lilli Gard, Sigrid Rosema, Coretta Van Leer-Buter, Xuewei Zhou, Marjolein Knoester
EPI_ISL_2709231	Public Health Laboratory, Minnesota Department of Health	University of Minnesota Genomics Center	Daryl M. Gohl, John Garbe, Jaquelyn Kuriger-Laber, Corbin Dirkx, and Sean Wang
EPI_ISL_2709498, EPI_ISL_2709517, EPI_ISL_2709541, EPI_ISL_2709543	Aegis Sciences Corporation	Centers for Disease Control and Prevention Division of Viral Diseases, Pathogen Discovery	Dakota Howard, Dhwani Batra, Peter W. Cook, Kara Moser, Adrian Paskey, Jason Caravas, Benjamin Rambo-Martin, Shatavia Morrison, Christopher Gulvick, Scott Sammons, Yvette Unoarumhi, Darlene Wagner, Matthew Schmerer, Cyndi Clark, Patrick Campbell, Rob Case, Vikramsinha Ghorpade, Holly Houdeshell, Ola Kvalvaag, Dillon Nall, Ethan Sanders, Alec Vest, Shaun Westlund, Matthew Hardison, Clinton R. Paden, Duncan MacCannell
EPI_ISL_2709816	MD PHL	MD PHL	Maryland Department of Health Laboratories Administration
EPI_ISL_2712536, EPI_ISL_2712537	Sharp HealthCare Laboratory	Andersen lab at Scripps Research	SEARCH Alliance San Diego with Aaron Harding, Jacquelyn Berumen, Cathy Woerle, Liam McGinnis, Art Mendoza, Omid Bakhtar
EPI_ISL_2712921	Wyoming Public Health Laboratory	Wyoming Public Health Laboratory	Jim Mildenberger, Taylor Fearing, Channing Weber, Ashley Norberg, Chayse Rowley, Marley Goetz, Brian Dominguez, Elliot Thomasson, Sam Britz, Cari Sloma, Robert Petit, and Rob Christensen
EPI_ISL_2716293	Kansas Health and Environmental Lab	Kansas Health and Environmental Lab	Katherine Wiggins, Mike Grose, Jonathan Barnell, Ben Olsen, and Phil Adam
EPI_ISL_2716405, EPI_ISL_2716406, EPI_ISL_2716407, EPI_ISL_2716411	Arizona State Public Health Laboratory	Arizona State Public Health Laboratory	Trung Huynh, Jessica Escobar, Katherine Fullerton, Nobuko Fukushima, Matthew Contursi, Stacy White, Linda Getsinger, Victor Waddell
EPI_ISL_2716463, EPI_ISL_2716464, EPI_ISL_2716466	Washington State Department of Health Public Health Laboratories	Washington State Department of Health Public Health Laboratories	Drew MacKellar, Philip Dykema, Denny Russell, Joenice Gonzalez, Hannah Gray, Geoff Melly, Vanessa De Los Santos, Darren Lucas, JohnAric Peterson, Avi Singh, Rebecca Cao
EPI_ISL_2716479, EPI_ISL_2716488, EPI_ISL_2716734	UW Virology Lab	UW Virology Lab	Pavitra Roychoudhury, Hong Xie, Lasata Shrestha, Shah Mohamed Bakhash, Tien V. Nguyen, Noah R. Baker, Sean Ellis, Meei-Li Huang, Keith R Jerome, Alexander Greninger
EPI_ISL_2716912, EPI_ISL_2716924, EPI_ISL_2716935	Southern Nevada Public Health Laboratory	Southern Nevada Public Health Laboratory	Michael Picker
EPI_ISL_2724792, EPI_ISL_2724855, EPI_ISL_2724856, EPI_ISL_2724857, EPI_ISL_2724858, EPI_ISL_2724862, EPI_ISL_2724869, EPI_ISL_2724873, EPI_ISL_2724876, EPI_ISL_2724880, EPI_ISL_2724885, EPI_ISL_2724889, EPI_ISL_2724891, EPI_ISL_2724899, EPI_ISL_2724900, EPI_ISL_2724901, EPI_ISL_2724902			
see above	State Laboratories Division, Hawaii State Department of Health	State Laboratories Division, Hawaii State Department of Health	Pamela O'Brien, Drew Kuwazaki, Ayana Garnet, Razvan Sultana, Edward Desmond
EPI_ISL_2725184, EPI_ISL_2725186, EPI_ISL_2725190, EPI_ISL_2725204, EPI_ISL_2725206, EPI_ISL_2725209, EPI_ISL_2725210, EPI_ISL_2725212, EPI_ISL_2725213, EPI_ISL_2725223, EPI_ISL_2725229, EPI_ISL_2725238, EPI_ISL_2725239, EPI_ISL_2725245, EPI_ISL_2725248, EPI_ISL_2725249, EPI_ISL_2725250, EPI_ISL_2725252, EPI_ISL_2725256, EPI_ISL_2725257, EPI_ISL_2725263, EPI_ISL_2725265, EPI_ISL_2725266, EPI_ISL_2725268, EPI_ISL_2725271, EPI_ISL_2725275, EPI_ISL_2725276, EPI_ISL_2725281, EPI_ISL_2725282, EPI_ISL_2725294			
see above	Lab. Microbiologia e Virologia Cotugno A.O. dei Colli - Istituto Zooprofilattico Sperimentale del Mezzogiorno	Telethon Institute of Genetics and Medicine (TIGEM)	Antonio Grimaldi Patrizia Annunziata Francesco Panariello Biancamaria Pierri Claudia Tiberio Teresa Giuliano Valentina Bouche Chiara Colantuono Maria Concetta Cuomo Denise Di Concilio Lucio Di Filippo Anna Manfredi Marcello Salvi Antonio Limone Luigi Atripaldi Pellegrino Cerino Andrea Ballabio Davide Cacchiarelli
EPI_ISL_2726819	Wyoming Public Health Laboratory	Wyoming Public Health Laboratory	Jim Mildenberger, Taylor Fearing, Channing Weber, Ashley Norberg, Chayse Rowley, Marley Goetz, Brian Dominguez, Elliot Thomasson, Sam Britz, Cari Sloma, Robert Petit, and Rob Christensen
EPI_ISL_2726937, EPI_ISL_2726940	ISDH	Quantigen Biosciences	Paul J. Childress
EPI_ISL_2727766, EPI_ISL_2727767, EPI_ISL_2727775, EPI_ISL_2727782, EPI_ISL_2727792, EPI_ISL_2727817, EPI_ISL_2727826	Wisconsin State Laboratory of Hygiene Communicable Disease Division	Wisconsin State Laboratory of Hygiene Communicable Disease Division	Abigail C. Shockey, Alicia J. Mooney, Erika M. Hanson, Tonya Danz, Richard Griesser, Sara Wagner, Kelsey R. Florek
EPI_ISL_2727868	HealthPartners Central Lab	Minnesota Department of Health, Public Health Laboratory	Alexandra Lorentz, Jacob Garfin, Matt Plumb, and Xiong Wang
EPI_ISL_2731453, EPI_ISL_2731454, EPI_ISL_2731455, EPI_ISL_2731456	Laboratorio Central de Saude Publica do Estado do Para (LACEN/PA)	Laboratory of Respiratory Viruses and Measles, Oswaldo Cruz Institute, FIOCRUZ	Paola Resende, Luciana Appolinario, Fernando Motta, Anna Carolina Paixao, Ana Carolina Mendonca, Alice Sampaio Rocha, Taina Venas, Elisa Cavalcante Pereira, Renata Serrano Lopes, Valnete Andrade, Marilda Siqueira on behalf of the Fiocruz COVID-19 Genomic Surveillance Network
EPI_ISL_2736280	Atlas Genomics	Seattle Flu Study	Deborah A. Nickerson, Chris D. Frazar, Jover Lee, Benjamin Pelle, Erica Ryke, Matthew Richardson, Amanda Adler, Elisabeth Brandstetter, Peter D. Han, Kairsten Fay, Misja Ilcisin, Kirsten Lacombe, Thomas R. Sibley, Melissa Truong, Caitlin R. Wolf, Romesh Gautom, Geoff Melly, Brian Hiatt, Philip Dykema, Scott Lindquist, Michael Boeckh, Janet A. Englund, Michael Famulare, Barry R. Lutz, Mark J. Rieder, Lea M. Starita, Matthew Thompson, Helen Y. Chu, Jay Shendure, Trevor Bedford
EPI_ISL_2753648	Missouri State Public Health Laboratory	Missouri State Public Health Laboratory	Matthew Sinn, Joshua Barry, Ashley New
EPI_ISL_2753941	NYU Langone Health	Departments of Pathology and Medicine, New York University School of Medicine	Adriana Heguy, Dacia Dimartino, Emily Guzman, Christian Marier, Peter Meyn, Sitharam Ramaswami, Gael Westby, Paul Zappile, Yutong Zhang, Paolo Cotzia, Guiqing Wang
EPI_ISL_2754064	Wyoming Public Health Laboratory	Wyoming Public Health Laboratory	Jim Mildenberger, Taylor Fearing, Channing Weber, Ashley Norberg, Chayse Rowley, Marley Goetz, Brian Dominguez, Elliot Thomasson, Sam Britz, Cari Sloma, Robert Petit, and Rob Christensen
EPI_ISL_2754305	Arizona State Public Health Laboratory	Arizona State Public Health Laboratory	Trung Huynh, Jessica Escobar, Katherine Fullerton, Nobuko Fukushima, Matthew Contursi, Stacy White, Linda Getsinger, Victor Waddell

EPI_ISL_2756057, EPI_ISL_2756058, EPI_ISL_2756060, EPI_ISL_2756061, EPI_ISL_2756062, EPI_ISL_2756063, EPI_ISL_2756064, EPI_ISL_2756066, EPI_ISL_2756067, EPI_ISL_2756068, EPI_ISL_2756069, EPI_ISL_2756070, EPI_ISL_2756071, EPI_ISL_2756072, EPI_ISL_2756073, EPI_ISL_2756077, EPI_ISL_2756078, EPI_ISL_2756079, EPI_ISL_2756080, EPI_ISL_2756081, EPI_ISL_2756082, EPI_ISL_2756085, EPI_ISL_2756088, EPI_ISL_2756092, EPI_ISL_2756094, EPI_ISL_2756095, EPI_ISL_2756097, EPI_ISL_2756100, EPI_ISL_2756101, EPI_ISL_2756104, EPI_ISL_2756105, EPI_ISL_2756106, EPI_ISL_2756108, EPI_ISL_2756109, EPI_ISL_2756112, EPI_ISL_2756114, EPI_ISL_2756115, EPI_ISL_2756116, EPI_ISL_2756119, EPI_ISL_2756121, EPI_ISL_2756122, EPI_ISL_2756124, EPI_ISL_2756125, EPI_ISL_2756126, EPI_ISL_2756128, EPI_ISL_2756129, EPI_ISL_2756130, EPI_ISL_2756134, EPI_ISL_2756138, EPI_ISL_2756139, EPI_ISL_2756140, EPI_ISL_2756141, EPI_ISL_2756142, EPI_ISL_2756143, EPI_ISL_2756144, EPI_ISL_2756147, EPI_ISL_2756148, EPI_ISL_2756149, EPI_ISL_2756172, EPI_ISL_2756173, EPI_ISL_2756175, EPI_ISL_2756180, EPI_ISL_2756181, EPI_ISL_2756182, EPI_ISL_2756183, EPI_ISL_2756184, EPI_ISL_2756185, EPI_ISL_2756186, EPI_ISL_2756187, EPI_ISL_2756188, EPI_ISL_2756189, EPI_ISL_2756190, EPI_ISL_2756191, EPI_ISL_2756192, EPI_ISL_2756193, EPI_ISL_2756194, EPI_ISL_2756195			
see above	Genetica Molecular and Subdepartamento de Virologia ISP Chile	Instituto de Salud Publica de Chile	Karen Orostica, Constanza Campano, Barbara Parra, Loredana Arata, Gisselle Barra, Patricia Bustos, Rodrigo Fasce, Javier Tognarelli, Andres Castillo, Soledad Ulloa, Jorge Fernandez
EPI_ISL_2756212	National Platform bis UMONS/Jolimont	National Platform bis UMONS/Jolimont	François Dufrasne, Guillaume Bayon-Vicente, Florian Juszczak, Eric Tarantino, Gautier Detry, Ruddy Wattiez
EPI_ISL_2756222, EPI_ISL_2756223, EPI_ISL_2756226, EPI_ISL_2756228, EPI_ISL_2756230, EPI_ISL_2756233, EPI_ISL_2756234, EPI_ISL_2756236, EPI_ISL_2756237, EPI_ISL_2756240, EPI_ISL_2756243, EPI_ISL_2756244, EPI_ISL_2756245, EPI_ISL_2756246, EPI_ISL_2756247, EPI_ISL_2756249, EPI_ISL_2756251, EPI_ISL_2756252, EPI_ISL_2756253, EPI_ISL_2756256, EPI_ISL_2756257, EPI_ISL_2756260, EPI_ISL_2756264, EPI_ISL_2756269, EPI_ISL_2756272, EPI_ISL_2756273, EPI_ISL_2756274, EPI_ISL_2756286, EPI_ISL_2756287, EPI_ISL_2756289, EPI_ISL_2756291, EPI_ISL_2756293, EPI_ISL_2756294, EPI_ISL_2756295, EPI_ISL_2756296, EPI_ISL_2756297, EPI_ISL_2756299, EPI_ISL_2756302, EPI_ISL_2756303, EPI_ISL_2756304, EPI_ISL_2756306, EPI_ISL_2756309, EPI_ISL_2756310, EPI_ISL_2756311, EPI_ISL_2756313, EPI_ISL_2756314, EPI_ISL_2756315, EPI_ISL_2756317, EPI_ISL_2756325, EPI_ISL_2756327, EPI_ISL_2756331, EPI_ISL_2756335, EPI_ISL_2756338			
see above	Genetica Molecular and Subdepartamento de Virologia ISP Chile	Instituto de Salud Publica de Chile	Karen Orostica, Constanza Campano, Barbara Parra, Loredana Arata, Gisselle Barra, Patricia Bustos, Rodrigo Fasce, Javier Tognarelli, Andres Castillo, Soledad Ulloa, Jorge Fernandez
EPI_ISL_2758010, EPI_ISL_2758012	Austrian Agency for Health and Food Safety (AGES)	Bergthaler laboratory, CeMM Research Center for Molecular Medicine of the Austrian Academy of Sciences	Lukas Endler, Anna Schedl, Fabian Amman, Petr Triska, Matthew Thornton, Thomas Penz, Benedikt Agerer, Maelle Le Moing, Michael Schuster, Bekir Erguner, Jan Laine, Martin Senekowitsch, Christoph Bock, Andreas Bergthaler
EPI_ISL_2758372, EPI_ISL_2758373	Berkeley Medical Center	WVU and Marshall University Combined Genomics Core Facilities	James Denvir, Peter Stoilov, Peter Perrotta, Wesley Kimble, Ryan Percifield
EPI_ISL_2758565, EPI_ISL_2758594	Servicio de Microbiologia Hospital Ramon y Cajal	Servicio de Microbiologia Hospital Ramon y Cajal	Ponce M, Galan JC, Martinez L. Abreu M, y Gonzalez-Alba JM
EPI_ISL_2758925	The Ohio State University Applied Microbiology Services Laboratory	The Ohio State University Applied Microbiology Services Laboratory	Seth A. Faith PhD
EPI_ISL_2759880	Washington State Department of Health Public Health Laboratories	Washington State Department of Health Public Health Laboratories	Drew MacKellar, Philip Dykema, Denny Russell, Joenice Gonzalez, Hannah Gray, Geoff Melly, Vanessa De Los Santos, Darren Lucas, JohnAric Peterson, Avi Singh, Rebecca Cao
EPI_ISL_2761224	Sonic - Bioscientia - MVZ Labor Saar GmbH	Robert Koch Institute	unknown
EPI_ISL_2761541	LabKom - Labor Hannover MVZ GmbH	Robert Koch Institute	unknown
EPI_ISL_2762312, EPI_ISL_2762347, EPI_ISL_2762356, EPI_ISL_2762365, EPI_ISL_2762395, EPI_ISL_2762431	Diagnosticum - Labor Neukirchen	Robert Koch Institute	unknown
EPI_ISL_2769118	UniversitätsSpital Zürich	Institute of Medical Virology	Verena Kufner, Gabriela Ziltener, Maryam Zaheri, Stefan Schmutz, Annette Audigé, Maria Grünberg, Kevin Steiner, Jon Huder, Cyril Shah, Riccarda Capaul, Guido Bloemberg, Jürg Böni, Michael Huber, Alexandra Trkola
EPI_ISL_2769191	TGen North	TGen North	"Jolene Bowers, Heather Centner, Brett Van Tassel, Chris French, Hayley Yaglom, Darrin Lemmer, Dave Engelthaler"
EPI_ISL_2773856	Hospital Universitario Virgen de las Nieves de Granada-SAS	SeqCOVID-SPAIN consortium/IBV(CSIC)	Sara Sanbonmatsu Gámez, Irene Pedrosa Corral, José M. Navarro-Marí and SeqCOVID-SPAIN consortium
EPI_ISL_2774104	Microbiology Department, University Hospital Donostia	Microbiology Department, University Hospital Donostia	Marimon JM, Montes M, Piñeiro L, Martin-Peñaranda T, Gomez M, Sorarrain A, Cilla G.
EPI_ISL_2774367, EPI_ISL_2774391	Illinois Department of Public Health - Springfield Lab	Illinois Department of Public Health - Springfield Lab	Bryan Sim, Gordon McCall
EPI_ISL_2774572	Torrance Memorial Medical Center	Los Angeles County PHL	P. Hemarajata et al.
EPI_ISL_2811913	Laboratory Medicine, University of Washington	Laboratory Medicine, University of Washington	Castor,J.L.

Supp. Table S7

We gratefully acknowledge the following Authors from the Originating laboratories responsible for obtaining the specimens, as well as the Submitting laboratories where the genome data were generated and shared via GISAID, on which this research is based.

All Submitters of data may be contacted directly via www.gisaid.org

Authors are sorted alphabetically.

Accession ID	Originating Laboratory	Submitting Laboratory	Authors
EPI_ISL_1471863, EPI_ISL_1471954	Pandemic Response Lab - NYC	Pandemic Response Lab, R&D	Henry Lee, Michael Hammerling, Melissa Hopkins, Cybill del Castillo, Shinyoung Clair Kang, William Ward, Pradeep Bugga, Sol Rey, Dylan Law, Haiping Hao, Jon Laurent
EPI_ISL_1477036, EPI_ISL_1477037	National Public Health Laboratory, National Centre for Infectious Diseases	National Public Health Laboratory, National Centre for Infectious Diseases	Tze Minn Mak, Zhenyang Zhou, Grace Jie Yin Ngan, Royce Ang, Lin Cui, Raymond Tzer Pin Lin
EPI_ISL_1529030, EPI_ISL_1529047, EPI_ISL_1529049, EPI_ISL_1529060	Berkeley Medical Center	WVU and Marshall University Combined Genomics Core Facilities	"James Denvir, Peter Stoilov, Peter Perrotta, Wesley Kimble, Ryan Percifield"
EPI_ISL_1531739	School of Pharmacy, Shenandoah University	School of Pharmacy, Shenandoah University	Adams,S.M., Harralson,A.F., Kidd,R.S., Sawyer,G.W.
EPI_ISL_1534465, EPI_ISL_1534477, EPI_ISL_1534478	Ministry of Health Turkey	Ministry of Health Turkey	Fatma Bayrakdar, Yasemin Cosgun, Suleyman Yalcin, Gulay Korukluoglu
EPI_ISL_1534725, EPI_ISL_1534731	AZDelta	AZDelta	Geert Martens; Dieter De Smet
EPI_ISL_1536186	Lighthouse Lab in Alderley Park	Wellcome Sanger Institute for the COVID-19 Genomics UK (COG-UK) Consortium	Jacquelyn Wynn, Mairead Hyland, The Lighthouse Lab in Alderley Park and Alex Alderton, Roberto Amato, Jeffrey Barrett, Sonia Goncalves, Ewan Harrison, David K. Jackson, Ian Johnston, Dominic Kwiatkowski, Cordelia Langford, John Sillitoe on behalf of the Wellcome Sanger Institute COVID-19 Surveillance Team
EPI_ISL_1542264, EPI_ISL_1543103	Pandemic Response Lab - NYC	Pandemic Response Lab, R&D	Henry Lee, Michael Hammerling, Melissa Hopkins, Cybill del Castillo, Shinyoung Clair Kang, William Ward, Pradeep Bugga, Sol Rey, Dylan Law, Katharine Nelson, Haiping Hao, Jon Laurent
EPI_ISL_1547506	Cerballiance Pont Audemer	Cerba Lab	Haïm-Boukobza S, Prigent A, Leriche A, Verdurme L, Trombert-Paolantoni S, Lecorche E, Roquebert B
EPI_ISL_1567223	Limbach - MVZ Humangenetik Ulm	Robert Koch Institute	unknown
EPI_ISL_1571169, EPI_ISL_1571188	Limbach - MVZ Labor Eveld & Kollegen Essen	Robert Koch Institute	unknown
EPI_ISL_1573183	MVZ Labor Dr. Limbach & Kollegen GbR	Robert Koch Institute	unknown
EPI_ISL_1593082	Helix/Illumina	Centers for Disease Control and Prevention Division of Viral Diseases, Pathogen Discovery	Dakota Howard, Dhwani Batra, Peter W. Cook, Kara Moser, Adrian Paskey, Jason Caravas, Benjamin Rambo-Martin, Shatavia Morrison, Christopher Gulvick, Scott Sammons, Yvette Unoarumhi, Darlene Wagner, Matthew Schmerer, Eileen de Feo, Jan Antico, Christine Tran, Matthew Tolentino, Shannon Wickline, Kim Gietzen, Brad Sickler, Jingtao Liu, Eric Allen, Phil Febbo, Nicole L. Washington, Simon White, Geraint Levan, Kelly Schiabor Barrett, Elizabeth Cirulli, Alexandre Bolze, Ary Ascencio, Charlotte Rivera-Garcia, Ryan Cho, Jason Nguyen, Sherry Wang, Jimmy Ramirez, Tyler Cassens, Efren Sandoval, Magnus Isaksson, William Lee, David Becker, Marc Laurent, James Lu, Clinton R. Paden, Duncan MacCannell
EPI_ISL_1595840	Armed Forces Institute of Pathology (AFIP), Dhaka Cantonment	Genomic Research Lab, BCSIR	Md. Saddam Hossain, Md. Murshed Hasan Sarkar, Mohammad Samir Uzzaman, Eshrar Osman, Md. Ahasan Habib, Shahina Akter, Tanjina Akhter Banu, Abu Sayeed Mohammad Mahmud, Barna Goswami, Iffat Jahan, Mohammad Mohi Uddin, Md. Kamrul Islam, Mohammad Mizanur Rahman, Susane Giti, Md. Salim Khan
EPI_ISL_1601510	UW Virology Lab	UW Virology Lab	Pavitra Roychoudhury, Hong Xie, Lasata Shrestha, Shah Mohamed Bakhash, Michelle Lin, Noah R. Baker, Sean Ellis, Saraswathi Sathees, Meei-Li Huang, Keith R Jerome, Alexander Greninger
EPI_ISL_1623609	CH VALENCE	CNR Virus des Infections Respiratoires - France SUD	Antonin Bal, Gregory Destras, Gwendolyne Burfin, Hadrien Regue, Quentin Semanas, Martine Valette, Bruno Lina, Laurence Josset
EPI_ISL_1624779, EPI_ISL_1624804, EPI_ISL_1624823, EPI_ISL_1624837, EPI_ISL_1624845, EPI_ISL_1624878, EPI_ISL_1624884	Berkeley Medical Center	WVU and Marshall University Combined Genomics Core Facilities	James Denvir, Peter Stoilov, Peter Perrotta, Wesley Kimble, Ryan Percifield
EPI_ISL_1624920	QLabs	WVU and Marshall University Combined Genomics Core Facilities	James Denvir, Peter Stoilov, Peter Perrotta, Wesley Kimble, Ryan Percifield
EPI_ISL_1624963, EPI_ISL_1624964, EPI_ISL_1624966, EPI_ISL_1624968, EPI_ISL_1624971, EPI_ISL_1624981	Berkeley Medical Center	WVU and Marshall University Combined Genomics Core Facilities	James Denvir, Peter Stoilov, Peter Perrotta, Wesley Kimble, Ryan Percifield
EPI_ISL_1628193	UW Virology Lab	UW Virology Lab	Pavitra Roychoudhury, Hong Xie, Lasata Shrestha, Shah Mohamed Bakhash, Michelle Lin, Noah R. Baker, Sean Ellis, Meei-Li Huang, Keith R Jerome, Alexander Greninger
EPI_ISL_1632926, EPI_ISL_1632929, EPI_ISL_1632930, EPI_ISL_1632933	Istituto Zooprofilattico Sperimentale del Mezzogiorno	Telethon Institute of Genetics and Medicine (TIGEM)	Antonio Grimaldi Patrizia Annunziata Francesco Panariello Biancamaria Pierri Claudia Tiberio Teresa Giuliano Valentina Bouche Chiara Colantuono Maria Concetta Cuomo Denise Di Concilio Lucio Di Filippo Anna Manfredi Marcello Salvi Antonio Limone Luigi Atripaldi Pellegrino Cerino Andrea Ballabio Davide Cacchiarelli
EPI_ISL_1633953, EPI_ISL_1634233, EPI_ISL_1636298	Pandemic Response Lab - NYC	Pandemic Response Lab, R&D	Henry Lee, Michael Hammerling, Melissa Hopkins, Cybill del Castillo, Shinyoung Clair Kang, William Ward, Pradeep Bugga, Sol Rey, Dylan Law, Katharine Nelson, Haiping Hao, Jon Laurent
EPI_ISL_1636430	Microbiology Department, Laboratori Clínic Metropolitana Nord. Hospital Universitari Germans Trias i Pujol.	Can Ruti SARS-CoV-2 Sequencing Hub (HUGTiP/IrsiCaixa/IGTP)	Marc Noguera-Julian, Pilar Armengol, Ignacio Blanco, Antoni E Bordoy, Francesc Catala-Moll, Pere-Joan Cardona, Maria Casadellà, Cristina Casañ, Gemma Clara, Bonaventura Clotet, Cristina Esteban, Montserrat Giménez, Mercedes Guerrero, Anna Not, Roger Paredes, Mariona Parera, Verónica Saludes, Alba Sánchez, and Elisa Martró on behalf of the Can Ruti SARS-CoV-2 Sequencing Hub.
EPI_ISL_1638287	MDI Limbach Berlin GmbH; MVZ Labor Berlin	Robert Koch Institute	unknown
EPI_ISL_1638309	Labor Prof. Dr. G. Enders MVZ GbR	Robert Koch Institute	unknown
EPI_ISL_1638315, EPI_ISL_1638326, EPI_ISL_1638343	MDI Limbach Berlin GmbH; MVZ Labor Berlin	Robert Koch Institute	unknown
EPI_ISL_1638359	MVZ Labor Dr. Limbach & Kollegen GbR	Robert Koch Institute	unknown
EPI_ISL_1638390, EPI_ISL_1638418	Labor Prof. Dr. G. Enders MVZ GbR	Robert Koch Institute	unknown
EPI_ISL_1638423, EPI_ISL_1638447, EPI_ISL_1638454	MDI Limbach Berlin GmbH; MVZ Labor Berlin	Robert Koch Institute	unknown
EPI_ISL_1640138	Uniklinikum Carl Gustav Carus an der TU Dresden; Institut fÃ¼r Virologie	Robert Koch Institute	unknown
EPI_ISL_1640231	Diagnosticum - Labor Neukirchen	Robert Koch Institute	unknown

EPI_ISL_1643785, EPI_ISL_1643786	Labor Prof. Dr. G. Enders MVZ GbR	Robert Koch Institute	unknown
EPI_ISL_1643809	Labor MÃ¶nchengladbach MVZ Dr. Stein + Kollegen GbR	Robert Koch Institute	unknown
EPI_ISL_1643861, EPI_ISL_1643867	MDI Limbach Berlin GmbH; MVZ Labor Berlin	Robert Koch Institute	unknown
EPI_ISL_1653428	Lighthouse Lab in Cambridge	Wellcome Sanger Institute for the COVID-19 Genomics UK (COG-UK) Consortium	Rob Howes, The Lighthouse Lab in Cambridge and Alex Alderton, Roberto Amato, Jeffrey Barrett, Sonia Goncalves, Ewan Harrison, David K. Jackson, Ian Johnston, Dominic Kwiatkowski, Cordelia Langford, John Sillitoe on behalf of the Wellcome Sanger Institute COVID-19 Surveillance Team
EPI_ISL_1653817	Washington State Department of Health Public Health Laboratories	Washington State Department of Health Public Health Laboratories	Drew MacKellar, Philip Dykema, Denny Russell, Joenice Gonzalez, Hannah Gray, Geoff Melly, Vanessa De Los Santos, Darren Lucas, JohnAric Peterson, Avi Singh, Rebecca Cao
EPI_ISL_1659194	Viollier AG	Department of Biosystems Science and Engineering, ETH Zurich	Chaoran Chen, Sarah Nadeau, Ivan Topolsky, Emmanouil Dermitzakis, Keith Harshman, Ioannis Xenarios, Henri Pegeot, Lorenzo Cerutti, Deborah Penet, Philipp Jablonski, Lara Fuhrmann, David Dreifuss, Katharina Jahn, Christiane Beckmann, Maurice Redondo, Olivier Kobel, Christoph Noppen, Sophie Seidel, Noemie Santamaria de Souza, Niko Beerenwinkel, Tanja Stadler
EPI_ISL_1663494	Capital hospital, Bhubaneswar	Institute of Life Sciences - INSACOG	Sunil K. Raghav, Safal Walia, Arup Ghosh, Atimukta Jha, Amol M. Kanampalliwar, Shifu Aggarwal, Rupesh Dash, Rajeeb Swain, Punit Prasad, INSACOG Consortium, Ajay Parida
EPI_ISL_1669441	Hospital Universitari Arnau de Vilanova	Hospital Universitari Vall d'Hebron - Vall d'Hebron Institut de Recerca	Cristina Andrés, Maria Piñana, Damir Garcia-Cehic, Ariadna Rando, Juliana Esperalba, Maria Gema Codina, Carla Castillo, Maria Carmen Martin, Tomàs Pumarola, Josep Quer, Andrés Antón
EPI_ISL_1670824	UW Virology Lab	UW Virology Lab	Pavitra Roychoudhury, Hong Xie, Lasata Shrestha, Shah Mohamed Bakhash, Michelle Lin, Noah R. Baker, Sean Ellis, Meei-Li Huang, Keith R Jerome, Alexander Greninger
EPI_ISL_1671819	CHU Lille	CHU Lille - Laboratoire de Virologie	AIT YAHYA Emilie, ALIDJINOU Enagnon Kazali, BOCKET Laurence, CREPIN Michel, DEMAY Christophe, ENGELMANN Ilka, GEFFROY Sandrine, GUIGON Aurélie, LAMBERT Valérie, LAZREK Mouna, NOBILLIAUX Florian, PREVOST Brigitte, THUILLIER Caroline, TINEZ Claire
EPI_ISL_1671823	CH Armentières	CHU Lille - Laboratoire de Virologie	AIT YAHYA Emilie, ALIDJINOU Enagnon Kazali, BOCKET Laurence, CREPIN Michel, DEMAY Christophe, ENGELMANN Ilka, GEFFROY Sandrine, GUIGON Aurélie, LAMBERT Valérie, LAZREK Mouna, NOBILLIAUX Florian, PREVOST Brigitte, THUILLIER Caroline, TINEZ Claire
EPI_ISL_1672812, EPI_ISL_1672821, EPI_ISL_1672825, EPI_ISL_1672827	Laboratorio Aziendale di Microbiologia e Virologia, Azienda Sanitaria dell'Alto Adige	Laboratorio Aziendale di Microbiologia e Virologia, Azienda Sanitaria dell'Alto Adige	Irene Bianconi, Bartolomeo Mobilio Rodriguez, Elisabetta Giacobazzi, Elisa Masi, Stefanie Wieser, Anne Picard, Claudia Volpato, Chiara Cantaloni, Elisabetta Pagani
EPI_ISL_1673424, EPI_ISL_1673587	Istituto Zooprofilattico Sperimentale del Mezzogiorno	TIGEM	Antonio Grimaldi Patrizia Annunziata Francesco Panariello Biancamaria Pierri Claudia Tiberio Teresa Giuliano Valentina Bouche Chiara Colantuono Maria Concetta Cuomo Denise Di Concilio Lucio Di Filippo Anna Manfredi Marcello Salvi Antonio Limone Luigi Atripaldi Pellegrino Cerino Andrea Ballabio Davide Cacchiarelli
EPI_ISL_1680352	UW Virology Lab	UW Virology Lab	Pavitra Roychoudhury, Hong Xie, Lasata Shrestha, Shah Mohamed Bakhash, Michelle Lin, Noah R. Baker, Sean Ellis, Meei-Li Huang, Keith R Jerome, Alexander Greninger
EPI_ISL_1692649, EPI_ISL_1692652	Istituto Zooprofilattico Sperimentale del Mezzogiorno	Telethon Institute of Genetics and Medicine (TIGEM)	Antonio Grimaldi Patrizia Annunziata Francesco Panariello Biancamaria Pierri Claudia Tiberio Teresa Giuliano Valentina Bouche Chiara Colantuono Maria Concetta Cuomo Denise Di Concilio Lucio Di Filippo Anna Manfredi Marcello Salvi Antonio Limone Luigi Atripaldi Pellegrino Cerino Andrea Ballabio Davide Cacchiarelli
EPI_ISL_1695028, EPI_ISL_1695029	Laboratory Corporation of America	Centers for Disease Control and Prevention Division of Viral Diseases, Pathogen Discovery	Dakota Howard, Dhwani Batra, Peter W. Cook, Kara Moser, Adrian Paskey, Jason Caravas, Benjamin Rambo-Martin, Shatavia Morrison, Christopher Gulvick, Scott Sammons, Yvette Unoarumhi, Darlene Wagner, Matthew Schmerer, Minoo Agarwal, Eyad Almasri, Debbie Boles, Ayla Burns, Nuthawin Charoensri, Oren Cohen, Susan Countryman, Mary Ann Cristobal, Bobbi Croy, Suzanne Dale, Hrushikesh Deshmukh, Amanda Douglas, Vincent Drouillon, Marcia Eisenberg, Howard Engler, Rama Ghatti, Prashant Gupta, Susan Hicks, Jake Humphrey, Lax Iyer, Manoj Jain, Mohan Kolli, Brian Krueger, Tim Kuphal, Stanley Letovsky, Michael Levandoski, Craig Lukasik, Jonathan Meltzer, Brian Norvell, Mindy Nye, Scott Parker, Christos Petropoulos, John Pruitt, Steven Ragan, Scott Ryan, Mike Sapeta, Jana Schroth, Suresh Babu Selvaraju, Goran Stevovic, Amanda Suchanek, Andrea Throop, Lyndon Tilson, Thomas Urban, Joe Voshell, Kimberly Wagner, Jonathan Williams, Mary Williamson, Qian Zeng, Tricia Zwiefelhofer, Clinton R. Paden, Duncan MacCannell
EPI_ISL_1695436, EPI_ISL_1695443, EPI_ISL_1695444	LABORATOIRE DE BIOLOGIE MEDICALE	CNR Virus des Infections Respiratoires - France SUD	Antonin Bal, Gregory Destras, Gwendolyne Burfin, Hadrien Regue, Quentin Semanas, Martine Valette, Bruno Lina, Laurence Josset
EPI_ISL_1701870, EPI_ISL_1701871, EPI_ISL_1701875	Laboratory Corporation of America	Centers for Disease Control and Prevention Division of Viral Diseases, Pathogen Discovery	Dakota Howard, Dhwani Batra, Peter W. Cook, Kara Moser, Adrian Paskey, Jason Caravas, Benjamin Rambo-Martin, Shatavia Morrison, Christopher Gulvick, Scott Sammons, Yvette Unoarumhi, Darlene Wagner, Matthew Schmerer, Minoo Agarwal, Eyad Almasri, Debbie Boles, Ayla Burns, Nuthawin Charoensri, Oren Cohen, Susan Countryman, Mary Ann Cristobal, Bobbi Croy, Suzanne Dale, Hrushikesh Deshmukh, Amanda Douglas, Vincent Drouillon, Marcia Eisenberg, Howard Engler, Rama Ghatti, Prashant Gupta, Susan Hicks, Jake Humphrey, Lax Iyer, Manoj Jain, Mohan Kolli, Brian Krueger, Tim Kuphal, Stanley Letovsky, Michael Levandoski, Craig Lukasik, Jonathan Meltzer, Brian Norvell, Mindy Nye, Scott Parker, Christos Petropoulos, John Pruitt, Steven Ragan, Scott Ryan, Mike Sapeta, Jana Schroth, Suresh Babu Selvaraju, Goran Stevovic, Amanda Suchanek, Andrea Throop, Lyndon Tilson, Thomas Urban, Joe Voshell, Kimberly Wagner, Jonathan Williams, Mary Williamson, Qian Zeng, Tricia Zwiefelhofer, Clinton R. Paden, Duncan MacCannell
EPI_ISL_1704843, EPI_ISL_1704844	National Public Health Laboratory, National Centre for Infectious Diseases	National Public Health Laboratory, National Centre for Infectious Diseases	Tze Minn Mak, Zhenyang Zhou, Grace Jie Yin Ngan, Royce Ang, Lin Cui, Raymond Tzer Pin Lin
EPI_ISL_1710705	School of Pharmacy, Shenandoah University	School of Pharmacy, Shenandoah University	Adams,S.M., Harralson,A.F., Kidd,R.S., Sawyer,G.W.
EPI_ISL_1715048	Istituto Zooprofilattico Sperimentale del Mezzogiorno	TIGEM	Antonio Grimaldi Patrizia Annunziata Francesco Panariello Biancamaria Pierri Claudia Tiberio Teresa Giuliano Valentina Bouche Chiara Colantuono Maria Concetta Cuomo Denise Di Concilio Lucio Di Filippo Anna Manfredi Marcello Salvi Antonio Limone Luigi Atripaldi Pellegrino Cerino Andrea Ballabio Davide Cacchiarelli
EPI_ISL_1718858	Lighthouse Lab in Milton Keynes	Wellcome Sanger Institute for the COVID-19 Genomics UK (COG-UK) Consortium	The Lighthouse Lab in Milton Keynes and Alex Alderton, Roberto Amato, Jeffrey Barrett, Sonia Goncalves, Ewan Harrison, David K. Jackson, Ian Johnston, Dominic Kwiatkowski, Cordelia Langford, John Sillitoe on behalf of the Wellcome Sanger Institute COVID-19 Surveillance Team
EPI_ISL_1719870	National Public Health Laboratory, National Centre for Infectious Diseases	National Public Health Laboratory, National Centre for Infectious Diseases	Tze Minn Mak, Zhenyang Zhou, Grace Jie Yin Ngan, Royce Ang, Lin Cui, Raymond Tzer Pin Lin
EPI_ISL_1720971	MVZ Dr. Eberhard & Partner Dortmund	Robert Koch Institute	unknown
EPI_ISL_1722672	MVZ Labor Dr. Limbach & Kollegen GbR	Robert Koch Institute	unknown
EPI_ISL_1724308, EPI_ISL_1724310	Labor Prof. Dr. G. Enders MVZ GbR	Robert Koch Institute	unknown
EPI_ISL_1724422, EPI_ISL_1724436	MVZ fÃ¼r Laboratoriumsmedizin und Mikrobiologie Koblenz-Mittelrhein (Labor Koblenz)	Robert Koch Institute	unknown
EPI_ISL_1725248	MDI Limbach Berlin GmbH; MVZ Labor Berlin	Robert Koch Institute	unknown
EPI_ISL_1725261	MVZ fÃ¼r Laboratoriumsmedizin und Mikrobiologie Koblenz-Mittelrhein (Labor Koblenz)	Robert Koch Institute	unknown
EPI_ISL_1725609, EPI_ISL_1725614, EPI_ISL_1725616, EPI_ISL_1725617, EPI_ISL_1725619, EPI_ISL_1725623, EPI_ISL_1725629	MDI Limbach Berlin GmbH; MVZ Labor Berlin	Robert Koch Institute	unknown
EPI_ISL_1725636, EPI_ISL_1725645	MVZ fÃ¼r Laboratoriumsmedizin und Mikrobiologie Koblenz-Mittelrhein (Labor Koblenz)	Robert Koch Institute	unknown

EPI_ISL_1726900	MVZ Labor Krone GbR	Robert Koch Institute	unknown
EPI_ISL_1727004, EPI_ISL_1727028, EPI_ISL_1727049	Medizinische Laboratorien DÃ¼sseldorf	Robert Koch Institute	unknown
EPI_ISL_1727121	Labor Dr. Heidrich & Kollegen MVZ GmbH Hamburg	Robert Koch Institute	unknown
EPI_ISL_1731352	National Virus Reference Laboratory	National Virus Reference Laboratory	Zoe Yandle, Charlene Bennett, Gabriel Gonzalez, Michael Carr, Jonathan Dean, Cillian F De Gascun
EPI_ISL_1737184	Aegis Sciences Corporation	Centers for Disease Control and Prevention Division of Viral Diseases, Pathogen Discovery	Dakota Howard, Dhwani Batra, Peter W. Cook, Kara Moser, Adrian Paskey, Jason Caravas, Benjamin Rambo-Martin, Shatavia Morrison, Christopher Gulvick, Scott Sammons, Yvette Unoarumhi, Darlene Wagner, Matthew Schmerer, Cyndi Clark, Patrick Campbell, Rob Case, Vikramsinha Ghorpade, Holly Houdeshell, Ola Kvalvaag, Dillon Nall, Ethan Sanders, Alec Vest, Shaun Westlund, Matthew Hardison, Clinton R. Paden, Duncan MacCannell
EPI_ISL_1742737	Berkeley Medical Center	WVU and Marshall University Combined Genomics Core Facilities	James Denvir, Peter Stoilov, Peter Perrotta, Wesley Kimble, Ryan Percifield
EPI_ISL_1745239, EPI_ISL_1745277, EPI_ISL_1745284, EPI_ISL_1745291, EPI_ISL_1745299, EPI_ISL_1745303, EPI_ISL_1745306, EPI_ISL_1745324, EPI_ISL_1745328	Azienda Sanitaria dell'Alto Adige Laboratorio Aziendale di Microbiologia e Virologia	Istituto di Genomica Applicata	Elisabetta Pagani, Irene Bianconi, Elisabetta Giacobazzi, Elisa Masi, Stefanie Wieser, Irena Jurman, Vera Vendramin, Gabriele Magris, Eleonora Paparelli, Davide Scaglione, Michele Morgante
EPI_ISL_1747406, EPI_ISL_1747407, EPI_ISL_1747420, EPI_ISL_1747436	Washington State Department of Health Public Health Laboratories	Washington State Department of Health Public Health Laboratories	Drew MacKellar, Philip Dykema, Denny Russell, Joenice Gonzalez, Hannah Gray, Geoff Melly, Vanessa De Los Santos, Darren Lucas, JohnAric Peterson, Avi Singh, Rebecca Cao
EPI_ISL_1755433	Biogroup Bio Lam-LCD Saint-Denis	Department of Virology, Henri Mondor University Hospital, Assistance Publique Hôpitaux de Paris, Université Paris-Est Créteil, INSERM U955	Christophe Rodriguez, Slim Fourati, Vanessa Demontant, Guillaume Gricourt, Melissa N'Debi, Alexandre Soulier, Elisabeth Trawinski, Jean-Michel Pawlotsky
EPI_ISL_1755791	Hôpital Avicenne	Department of Virology, Henri Mondor University Hospital, Assistance Publique Hôpitaux de Paris, Université Paris-Est Créteil, INSERM U955	Christophe Rodriguez, Slim Fourati, Vanessa Demontant, Guillaume Gricourt, Melissa N'Debi, Alexandre Soulier, Elisabeth Trawinski, Jean-Michel Pawlotsky
EPI_ISL_1756563, EPI_ISL_1756612, EPI_ISL_1756631, EPI_ISL_1756637	Biogroup Bio Lam-LCD Saint-Denis	Department of Virology, Henri Mondor University Hospital, Assistance Publique Hôpitaux de Paris, Université Paris-Est Créteil, INSERM U955	Christophe Rodriguez, Slim Fourati, Vanessa Demontant, Guillaume Gricourt, Melissa N'Debi, Alexandre Soulier, Elisabeth Trawinski, Jean-Michel Pawlotsky
EPI_ISL_1759578, EPI_ISL_1759697	Istituto Zooprofilattico Sperimentale del Mezzogiorno	TIGEM	Antonio Grimaldi Patrizia Annunziata Francesco Panariello Biancamaria Pierri Claudia Tiberio Teresa Giuliano Valentina Bouche Chiara Colantuono Maria Concetta Cuomo Denise Di Concilio Lucio Di Filippo Anna Manfredi Marcello Salvi Antonio Limone Luigi Atripaldi Pellegrino Cerino Andrea Ballabio Davide Cacchiarelli
EPI_ISL_1760146	Ministry of Health Turkey	Ministry of Health Turkey	Fatma Bayrakdar, Yasemin Cosgun, Suleyman Yalcin, Gulay Korukluoglu
EPI_ISL_1760379	Microbiologia e Virologia	Microbiologia e Virologia	L.Atripaldi, C. Tiberio, A.Perfetti
EPI_ISL_1761554	P.O.CARDARELLI	P.O.CARDARELLI	Scutellà M, Felice V, Niro G
EPI_ISL_1785083	SIESP CHIETI - DRIVE IN CHIETI	Istituto Zooprofilattico Sperimentale dell'Abruzzo e Molise "G. Caporale"	Lorusso A, Marcacci M, Di Domenico M, Ancora M, Curini V, Di Lollo Valeria, Mangone I, Rinaldi A, Delli Compagni E, Scialabba S, Caporale M, Di Pasquale A, Cammà C, Puglia I, Calistri P, Savini G
EPI_ISL_1785213, EPI_ISL_1785227	Università degli Studi di Perugia	Istituto Zooprofilattico Sperimentale dell'Abruzzo e Molise "G. Caporale"	Mencacci A, Camilloni B,Lorusso A, Marcacci M, Di Domenico M, Ancora M, Curini V, Di Lollo Valeria, Mangone I, Rinaldi A, Delli Compagni E, Scialabba S, Caporale M, Di Pasquale A, Cammà C, Puglia I, Calistri P, Savini G
EPI_ISL_1785311	National Virus Reference Laboratory	National Virus Reference Laboratory	Zoe Yandle, Charlene Bennett, Gabriel Gonzalez, Michael Carr, Jonathan Dean, Cillian F De Gascun
EPI_ISL_1785840, EPI_ISL_1785841	UW Virology Lab	UW Virology Lab	Pavitra Roychoudhury, Hong Xie, Lasata Shrestha, Tien V. Nguyen, Shah Mohamed Bakhash, Michelle Lin, Noah R. Baker, Sean Ellis, Meei-Li Huang, Keith R Jerome, Alexander Greninger
EPI_ISL_1788425	HOPITAL SAINT ANDRE	CNR Virus des Infections Respiratoires - France SUD	Antonin Bal, Gregory Destras, Gwendolyne Burfin, Hadrien Regue, Quentin Semanas, Martine Valette, Bruno Lina, Laurence Josset
EPI_ISL_1788551, EPI_ISL_1788552	LBM CLUSES	CNR Virus des Infections Respiratoires - France SUD	Antonin Bal, Gregory Destras, Gwendolyne Burfin, Hadrien Regue, Quentin Semanas, Martine Valette, Bruno Lina, Laurence Josset
EPI_ISL_1789246, EPI_ISL_1789456	UW Virology Lab	UW Virology Lab	Pavitra Roychoudhury, Hong Xie, Lasata Shrestha, Tien V. Nguyen, Shah Mohamed Bakhash, Michelle Lin, Noah R. Baker, Sean Ellis, Meei-Li Huang, Keith R Jerome, Alexander Greninger
EPI_ISL_1789618	M Health Fairview	Minnesota Department of Health, Public Health Laboratory	Alexandra Lorentz, Jacob Garfin, Matt Plumb, and Xiong Wang
EPI_ISL_1792899	Dutch COVID-19 response team	National Institute for Public Health and the Environment (RIVM)	Adam Meijer, Harry Vennema, Dirk Eggink, Jeroen Cremer, Sharon van den Brink, Bas van der Veer, AnneMarie van den Brandt, Lisa Wijsman, Kim Freriks, Ryanne Jaarsma, Euníce Then, Lynn Aarts, Sanne Bos, Melissa van Tuil, Robert Kohl, Linda van de Nes, Sjoerd Kuiling, James Groot, Florian Zwagemaker, Dennis Schmitz, Annelies Kroneman, Karim Hajji, Chantal Reusken, on behalf of the national COVID-19 response team
EPI_ISL_1793318, EPI_ISL_1793319	Istituto Zooprofilattico Sperimentale del Mezzogiorno	TIGEM	Antonio Grimaldi Patrizia Annunziata Francesco Panariello Biancamaria Pierri Claudia Tiberio Teresa Giuliano Valentina Bouche Chiara Colantuono Maria Concetta Cuomo Denise Di Concilio Lucio Di Filippo Anna Manfredi Marcello Salvi Antonio Limone Luigi Atripaldi Pellegrino Cerino Andrea Ballabio Davide Cacchiarelli
EPI_ISL_1798716	RI State Health Laboratories	Centers for Disease Control and Prevention Division of Viral Diseases, Pathogen Discovery	Mili Sheth, Sarah Nobles, Jasmine Padilla, Mark Burroughs, Shoshona Le, Katie Dillon, Peter Cook, Clinton R. Paden, Dhwani Batra, Krista Queen, Kristen Knipe, Dakota Howard, Yvette Unoarumhi, Darlene Wagner, Matthew Schmerer, Ben L. Rambo-Martin, Kristine Lacek, Sam Shepard, Alison Laufer Halpin, Dave Wentworth, Vivien Dugan, Suxiang Tong, Justin Lee
EPI_ISL_1804911, EPI_ISL_1804916, EPI_ISL_1804923	Laboratorio Aziendale di Microbiologia e Virologia, Azienda Sanitaria dell'Alto Adige	Laboratorio Aziendale di Microbiologia e Virologia, Azienda Sanitaria dell'Alto Adige	Irene Bianconi, Bartolomeo Mobilio Rodriguez, Elisabetta Giacobazzi, Elisa Masi, Stefanie Wieser, Anne Picard, Claudia Volpato, Chiara Cantaloni, Elisabetta Pagani
EPI_ISL_1804970	CQRC_QUALITY CONTROL CHEMICAL BIOLOGICAL RISK_AOOR Villa Sofia Cervello Palermo	CQRC_QUALITY CONTROL CHEMICAL BIOLOGICAL RISK_AOOR Villa Sofia Cervello Palermo	Di Gaudio, F., Contino, F., Brunacci, G.,
EPI_ISL_1808671	Swedish national genomic surveillance program of SARS-CoV-2	The Public Health Agency of Sweden	Maximilian Riess, Maria Lind Karlberg, Alma Brolund, Swedish national genomic surveillance program of SARS-CoV-2
EPI_ISL_1817303	National Laboratory for Health, Environment and Food, OMM, Kranj	NLZOH (National Laboratory for Health, Environment and Food) / CISLD (Clinical Institute of Special Laboratory Diagnostics), University Children's Hospital, University Medical Center Ljubljana	Sandra Janezic, Aleksander Mahnic, Maja Rupnik, Tjasa Žohar retnik, Alenka Štorman, Nika Gobec, Aleksander Kocuvan, Kaja Tominc, Maša Jari, Mateja Ravnik, Monika Korošec, Marjana Petrevi / Jernej Kova, Barbara Jenko Bizjan, Tine Tesovnik, Robert Šket, Katarina Kozmos, Ana Grom, Maruša Debeljak, Marko Pokorn, Tadej Battelino
EPI_ISL_1818074, EPI_ISL_1818075, EPI_ISL_1818077, EPI_ISL_1818078	Laboratory Corporation of America	Centers for Disease Control and Prevention Division of Viral Diseases, Pathogen Discovery	Dakota Howard, Dhwani Batra, Peter W. Cook, Kara Moser, Adrian Paskey, Jason Caravas, Benjamin Rambo-Martin, Shatavia Morrison, Christopher Gulvick, Scott Sammons, Yvette Unoarumhi, Darlene Wagner, Matthew Schmerer, Minoo Agarwal, Eyad Almasri, Debbie Boles, Ayla Burns, Nuthawin Charoensri, Oren Cohen, Susan Countryman, Mary Ann Cristobal, Bobbi Croy, Suzanne Dale, Hrushikesh Deshmukh, Amanda Douglas, Vincent Drouillon, Marcia Eisenberg, Howard Engler, Rama Ghatti, Prashant Gupta, Susan Hicks, Jake Humphrey, Lax Iyer, Manoj Jain, Mohan Kolli, Brian Krueger, Tim Kuphal, Stanley Letovsky, Michael Levandoski, Craig Lukasik, Jonathan Meltzer, Brian Norvell, Mindy Nye, Scott Parker, Christos Petropoulos, John Pruitt, Steven Ragan, Scott Ryan, Mike Sapeta, Jana Schroth, Suresh Babu Selvaraju, Goran Stevovic, Amanda Suchanek, Andrea Throop, Lyndon Tilson, Thomas Urban, Joe Voshell, Kimberly Wagner, Jonathan Williams, Mary Williamson, Qian Zeng, Tricia Zwiefelhofer, Clinton R. Paden, Duncan MacCannell
EPI_ISL_1820311, EPI_ISL_1820312,	Furst Medical Laboratory	Norwegian Institute of Public Health, Department of Virology	Kathrine Stene-Johansen, Kamilla Heddeland Instefjord, Hilde Elshaug, Garcia Llorente Ignacio, Jon Bråte, Engebretsen Serina Beate,Pedersen Benedikte

EPI_ISL_1820313			Nevjen, Debech Nadia, Atiya R Ali,Marie Paulsen Madsen, Rasmus Riis Kopperud, Hilde Vollan, Karoline Bragstad, Olav Hungnes
EPI_ISL_1820314	Medical Microbiology Unit, Department for Laboratory Medicine, Drammen Hospital, Vestre Viken Health Trust,	Norwegian Institute of Public Health, Department of Virology	Kathrine Stene-Johansen, Kamilla Heddeland Instefjord, Hilde Elshaug, Garcia Llorente Ignacio, Jon Bråte, Engebretsen Serina Beate,Pedersen Benedikte Nevjen, Debech Nadia, Atiya R Ali,Marie Paulsen Madsen, Rasmus Riis Kopperud, Hilde Vollan, Karoline Bragstad, Olav Hungnes
EPI_ISL_1820315	Furst Medical Laboratory	Norwegian Institute of Public Health, Department of Virology	Kathrine Stene-Johansen, Kamilla Heddeland Instefjord, Hilde Elshaug, Garcia Llorente Ignacio, Jon Bråte, Engebretsen Serina Beate,Pedersen Benedikte Nevjen, Debech Nadia, Atiya R Ali,Marie Paulsen Madsen, Rasmus Riis Kopperud, Hilde Vollan, Karoline Bragstad, Olav Hungnes
EPI_ISL_1820779	Hospital of Southern Norway - Kristiansand, Department of Medical Microbiology	Norwegian Institute of Public Health, Department of Virology	Kathrine Stene-Johansen, Kamilla Heddeland Instefjord, Hilde Elshaug, Garcia Llorente Ignacio, Jon Bråte, Engebretsen Serina Beate,Pedersen Benedikte Nevjen, Debech Nadia, Atiya R Ali,Marie Paulsen Madsen, Rasmus Riis Kopperud, Hilde Vollan, Karoline Bragstad, Olav Hungnes
EPI_ISL_1822672	Ministry of Health Turkey	Ministry of Health Turkey	Fatma Bayrakdar, Yasemin Cosgun, Suleyman Yalcin, Gulay Korukluoglu
EPI_ISL_1824146	Ospedale S.M. Goretti di Latina - ASL Latina presidio ospedaliero Nord	INMI Lazzaro Spallanzani IRCCS	B Bartolini, O Butera, F Messina, F De Nicola, A Massacci, A Lucci, L Di Biase, E Giombini
EPI_ISL_1826134, EPI_ISL_1826136	UW Virology Lab	UW Virology Lab	Pavitra Roychoudhury, Hong Xie, Lasata Shrestha, Tien V. Nguyen, Shah Mohamed Bakhash, Michelle Lin, Noah R. Baker, Sean Ellis, Meei-Li Huang, Keith R Jerome, Alexander Greninger
EPI_ISL_1830347	Lighthouse Lab in Alderley Park	Wellcome Sanger Institute for the COVID-19 Genomics UK (COG-UK) Consortium	Jacquelyn Wynn, Mairead Hyland, The Lighthouse Lab in Alderley Park and Alex Alderton, Roberto Amato, Jeffrey Barrett, Sonia Goncalves, Ewan Harrison, David K. Jackson, Ian Johnston, Dominic Kwiatkowski, Cordelia Langford, John Sillitoe on behalf of the Wellcome Sanger Institute COVID-19 Surveillance Team
EPI_ISL_1831596, EPI_ISL_1831620, EPI_ISL_1831643	Laboratori Clínic Territorial de Girona	Can Ruti SARS-CoV-2 Sequencing Hub (HUGTiP/IrsiCaixa/IGTP)	Marc Noguera-Julian, Pilar Armengol, Ignacio Blanco, Antoni E Bordoy, Francesc Catala-Moll, Pere-Joan Cardona, Maria Casadellà, Cristina Casañ, Gemma Clara, Bonaventura Clotet, Cristina Esteban, Montserrat Giménez, Mercedes Guerrero, Anna Not, Roger Paredes, Mariona Parera, Verónica Saludes, Alba Sánchez, and Elisa Martró on behalf of the Can Ruti SARS-CoV-2 Sequencing Hub.
EPI_ISL_1831681	Althaia. Xarxa Assistencial Universitària de Manresa	IrsiCaixa	Marc Noguera-Julian, Mariona Parera, Maria Casadellà, Pilar Armengol, Francesc Catala-Moll, Roger Paredes, Bonaventura Clotet Gloria Trujillo, Rafel Perez Vidal, Jaume Trape Pujol, Carolina Gonzalez Fernandez, Miquel Micó, Roger Paredes, Eulalia Grau, Bonaventura Clotet
EPI_ISL_1838837, EPI_ISL_1838838, EPI_ISL_1838848, EPI_ISL_1838857, EPI_ISL_1838858, EPI_ISL_1838859	CSIR-Centre for Cellular and Molecular Biology	CSIR-Centre for Cellular and Molecular Biology-INSACOG	Payel Mukherjee,Lamuk Zaveri,Tulasi Nagabandi, Ara Sreenivas,Shreekant Verma, Amareshwar Vodapalli ,Blessy B John,Viswagithe S L,B Himasri,Valli Nagalakshmi Undamatla,Onkar Kulkarni,Sofia Banu,Archana Bharadwaj Siva,Sharath Chandra Thota,Karthik Bharadwaj Tallapaka,Rakesh K Mishra,Divya Tej Sowpati
EPI_ISL_1840705, EPI_ISL_1840706, EPI_ISL_1840707, EPI_ISL_1840725, EPI_ISL_1840726, EPI_ISL_1840774	Azienda Sanitaria dell'Alto Adige Laboratorio Aziendale di Microbiologia e Virologia	Istituto di Genomica Applicata	Elisabetta Pagani, Irene Bianconi, Elisabetta Giacobazzi, Elisa Masi, Stefanie Wieser, Irena Jurman, Vera Vendramin, Gabriele Magris, Eleonora Paparelli, Davide Scaglione, Michele Morgante
EPI_ISL_1843336, EPI_ISL_1843342	Labor Prof. Dr. G. Enders MVZ GbR	Robert Koch Institute	unknown
EPI_ISL_1843353	MDI Limbach Berlin GmbH; MVZ Labor Berlin	Robert Koch Institute	unknown
EPI_ISL_1843356, EPI_ISL_1843359	Labor Prof. Dr. G. Enders MVZ GbR	Robert Koch Institute	unknown
EPI_ISL_1847179	MDI Limbach Berlin GmbH; MVZ Labor Berlin	Robert Koch Institute	unknown
EPI_ISL_1847182	MVZ fÃ¼r Laboratoriumsmedizin und Mikrobiologie Koblenz-Mittelrhein (Labor Koblenz)	Robert Koch Institute	unknown
EPI_ISL_1847194, EPI_ISL_1847204	MDI Limbach Berlin GmbH; MVZ Labor Berlin	Robert Koch Institute	unknown
EPI_ISL_1847217	MVZ fÃ¼r Laboratoriumsmedizin und Mikrobiologie Koblenz-Mittelrhein (Labor Koblenz)	Robert Koch Institute	unknown
EPI_ISL_1847221, EPI_ISL_1847318, EPI_ISL_1847330	MDI Limbach Berlin GmbH; MVZ Labor Berlin	Robert Koch Institute	unknown
EPI_ISL_1847724	MVZ fÃ¼r Laboratoriumsmedizin und Mikrobiologie Koblenz-Mittelrhein (Labor Koblenz)	Robert Koch Institute	unknown
EPI_ISL_1847725, EPI_ISL_1847729, EPI_ISL_1847734	MDI Limbach Berlin GmbH; MVZ Labor Berlin	Robert Koch Institute	unknown
EPI_ISL_1848754	Labor MÃ¶nchengladbach MVZ Dr. Stein + Kollegen GbR	Robert Koch Institute	unknown
EPI_ISL_1848783, EPI_ISL_1848801, EPI_ISL_1848803	MDI Limbach Berlin GmbH; MVZ Labor Berlin	Robert Koch Institute	unknown
EPI_ISL_1849374	UW Virology Lab	UW Virology Lab	Pavitra Roychoudhury, Hong Xie, Lasata Shrestha, Tien V. Nguyen, Shah Mohamed Bakhash, Michelle Lin, Noah R. Baker, Sean Ellis, Meei-Li Huang, Keith R Jerome, Alexander Greninger
EPI_ISL_1849448	MVZ Labor Krone GbR	Robert Koch Institute	unknown
EPI_ISL_1850314, EPI_ISL_1850325	MDI Limbach Berlin GmbH; MVZ Labor Berlin	Robert Koch Institute	unknown
EPI_ISL_1850360	MVZ Dr. Eberhard & Partner Dortmund	Robert Koch Institute	unknown
EPI_ISL_1854254	Instituto Nacional de Saude (INSA) and Institute of Biomedicine (iBiMed), Universidade de Aveiro	Instituto Nacional de Saude (INSA) and Institute of Biomedicine (iBiMed), Universidade de Aveiro	Borges et al
EPI_ISL_1854858	Clinical Molecular Microbiology Laboratory, UNC Hospitals	Jeremy Wang	Jeremy Wang, Alexander Rubinsteyn, Colleen Rice, Jason Smedberg, Shawn Hawken, Melissa Miller, Corbin Jones, Robert Hagan
EPI_ISL_1859259, EPI_ISL_1860240, EPI_ISL_1860959, EPI_ISL_1861130, EPI_ISL_1861219, EPI_ISL_1861375, EPI_ISL_1862921, EPI_ISL_1864252, EPI_ISL_1864267, EPI_ISL_1864300, EPI_ISL_1864685, EPI_ISL_1864956, EPI_ISL_1865121, EPI_ISL_1865528, EPI_ISL_1865859, EPI_ISL_1866443, EPI_ISL_1866463, EPI_ISL_1866643, EPI_ISL_1866657, EPI_ISL_1867049, EPI_ISL_1867586, EPI_ISL_1867794, EPI_ISL_1867948, EPI_ISL_1867985, EPI_ISL_1868453, EPI_ISL_1868805, EPI_ISL_1869041			
see above	Department of Virus and Microbiological Special Diagnostics, Statens Serum Institut, Copenhagen, Denmark	Aalborg University	Danish Covid-19 Genome Consortium
EPI_ISL_1869115	Department of Clinical Microbiology, Odense University Hospital, Odense, Denmark	Aalborg University	Danish Covid-19 Genome Consortium
EPI_ISL_1869166, EPI_ISL_1869173, EPI_ISL_1870601, EPI_ISL_1871094, EPI_ISL_1872516, EPI_ISL_1872655, EPI_ISL_1872832, EPI_ISL_1873667, EPI_ISL_1874383, EPI_ISL_1874957, EPI_ISL_1875518, EPI_ISL_1876635, EPI_ISL_1877251, EPI_ISL_1877698, EPI_ISL_1877699, EPI_ISL_1878067, EPI_ISL_1878539			
see above	Department of Virus and Microbiological Special Diagnostics, Statens Serum Institut, Copenhagen, Denmark	Aalborg University	Danish Covid-19 Genome Consortium
EPI_ISL_1878864	Department of Clinical Microbiology, Odense University Hospital, Odense, Denmark	Aalborg University	Danish Covid-19 Genome Consortium
EPI_ISL_1880159, EPI_ISL_1880486, EPI_ISL_1881473, EPI_ISL_1882091, EPI_ISL_1882513, EPI_ISL_1882669	Department of Virus and Microbiological Special Diagnostics, Statens Serum Institut, Copenhagen, Denmark	Aalborg University	Danish Covid-19 Genome Consortium
EPI_ISL_1883032	Department of Clinical Microbiology, Odense University Hospital, Odense, Denmark	Aalborg University	Danish Covid-19 Genome Consortium
EPI_ISL_1883194, EPI_ISL_1883530,	Department of Virus and Microbiological Special Diagnostics,	Aalborg University	Danish Covid-19 Genome Consortium

EPI_ISL_1883824, EPI_ISL_1884732, EPI_ISL_1885395, EPI_ISL_1885672, EPI_ISL_1885838, EPI_ISL_1885890	Statens Serum Institut, Copenhagen, Denmark		
EPI_ISL_1886140, EPI_ISL_1886168, EPI_ISL_1886196	Labor Berlin Charite Vivantes GmbH / Institut fur Virologie	Charite Universitatsmedizin Berlin, Institut fur Virologie/Labor Berlin	Peter Menzel, Christine Stephan, Rolf Schwarzer, Victor M Corman, Barbara Muhlemann, Terry Jones, Christian Drosten
EPI_ISL_1886602, EPI_ISL_1887377, EPI_ISL_1887695, EPI_ISL_1887862, EPI_ISL_1888564, EPI_ISL_1888933, EPI_ISL_1888934, EPI_ISL_1888978, EPI_ISL_1889098, EPI_ISL_1889264, EPI_ISL_1889401, EPI_ISL_1889907, EPI_ISL_1889991, EPI_ISL_1890001, EPI_ISL_1890828			
see above	Department of Virus and Microbiological Special Diagnostics, Statens Serum Institut, Copenhagen, Denmark	Aalborg University	Danish Covid-19 Genome Consortium
EPI_ISL_1891211, EPI_ISL_1891230, EPI_ISL_1891238, EPI_ISL_1891257, EPI_ISL_1891271, EPI_ISL_1891284	National Virus Reference Laboratory	National Virus Reference Laboratory	Zoe Yandle, Charlene Bennett, Gabriel Gonzalez, Michael Carr, Jonathan Dean, Cillian F De Gascun
EPI_ISL_1891710, EPI_ISL_1892157, EPI_ISL_1892287, EPI_ISL_1892288, EPI_ISL_1892426, EPI_ISL_1892499	Department of Virus and Microbiological Special Diagnostics, Statens Serum Institut, Copenhagen, Denmark	Aalborg University	Danish Covid-19 Genome Consortium
EPI_ISL_1892701	Department of Clinical Microbiology and Center for Genomic Medicine, Rigshospitalet, Copenhagen, Denmark	Aalborg University	Danish Covid-19 Genome Consortium
EPI_ISL_1892959	Laboratori Clínic Territorial de Girona	Can Ruti SARS-CoV-2 Sequencing Hub (HUGTiP/IrsiCaixa/IGTP)	Marc Noguera-Julian, Pilar Armengol, Ignacio Blanco, Antoni E Bordoy, Francesc Catala-Moll, Pere-Joan Cardona, Maria Casadellà, Cristina Casañ, Gemma Clara, Bonaventura Clotet, Cristina Esteban, Montserrat Giménez, Mercedes Guerrero, Anna Not, Roger Paredes, Mariona Parera, Verónica Saludes, Alba Sánchez, and Elisa Martró on behalf of the Can Ruti SARS-CoV-2 Sequencing Hub.
EPI_ISL_1892978, EPI_ISL_1893842, EPI_ISL_1894042, EPI_ISL_1894348, EPI_ISL_1894828, EPI_ISL_1894962, EPI_ISL_1894963	Department of Virus and Microbiological Special Diagnostics, Statens Serum Institut, Copenhagen, Denmark	Aalborg University	Danish Covid-19 Genome Consortium
EPI_ISL_1895041	Department of Clinical Microbiology, Odense University Hospital, Odense, Denmark	Aalborg University	Danish Covid-19 Genome Consortium
EPI_ISL_1895477, EPI_ISL_1895630, EPI_ISL_1895770, EPI_ISL_1896003, EPI_ISL_1896059, EPI_ISL_1896533, EPI_ISL_1896651	Department of Virus and Microbiological Special Diagnostics, Statens Serum Institut, Copenhagen, Denmark	Aalborg University	Danish Covid-19 Genome Consortium
EPI_ISL_1899146	Swedish national genomic surveillance program of SARS-CoV-2	The Public Health Agency of Sweden	Maximilian Riess, Maria Lind Karlberg, Alma Brolund, Swedish national genomic surveillance program of SARS-CoV-2
EPI_ISL_1904330	AZDelta	AZDelta	Geert Martens; Dieter De Smet
EPI_ISL_1905108	Labor Berlin Charite Vivantes GmbH / Institut fur Virologie	Charite Universitatsmedizin Berlin, Institut fur Virologie/Labor Berlin	Peter Menzel, Christine Stephan, Rolf Schwarzer, Victor M Corman, Barbara Muhlemann, Terry Jones, Christian Drosten
EPI_ISL_1908870	Hospital Universitari Arnau de Vilanova	Hospital Universitari Vall d'Hebron - Vall d'Hebron Institut de Recerca	Cristina Andrés, Maria Piñana, Damir Garcia-Cehic, Ariadna Rando, Juliana Esperalba, Maria Gema Codina, Carla Castillo, Maria Carmen Martin, Tomàs Pumarola, Josep Quer, Andrés Antón
EPI_ISL_1913252	Laboratorio de Virología HUCA	Laboratorio de Virología HUCA	Sandoval M, Castelló C, Gómez de Oña J, Boga JA, Rojo S, Alvarez-Arguelles ME, Abreu F, Costales I, Perez-Martínez Z, Martín-Rodríguez G, Coto E, Melón S
EPI_ISL_1914210	Viollier AG	Department of Biosystems Science and Engineering, ETH Zurich	Chaoran Chen, Sarah Nadeau, Catharine Aquino, Ivan Topolsky, Philipp Jablonski, Lara Fuhrmann, David Dreifuss, Katharina Jahn, Andreia Cabral de Gouvea, Maria Domenica Moccia, Simon Gruter, Timothy Sykes, Lennart Opitz, Griffin White, Laura Neff, Doris Popovic, Andrea Patrignani, Jay Tracy, Ralph Schlapbach, Christiane Beckmann, Maurice Redondo, Olivier Kobel, Christoph Noppen, Sophie Seidel, Noemie Santamaria de Souza, Niko Beerenwinkel, Tanja Stadler
EPI_ISL_1914285	ULSS 3 Venezia	Istituto Zooprofilattico Sperimentale delle Venezie	Adelaide Milani, Alessia Schivo, Annalisa Salviato, Elisa Palumbo, Erika Giorgia Quaranta, Luca Tassoni, Ambra Pastori, Edoardo Giussani, Alice Fusaro, Isabella Monne, Calogero Terregino, Antonia Ricci
EPI_ISL_1914286, EPI_ISL_1914287	ULSS 2 Marca Trevigiana	Istituto Zooprofilattico Sperimentale delle Venezie	Adelaide Milani, Alessia Schivo, Annalisa Salviato, Elisa Palumbo, Erika Giorgia Quaranta, Luca Tassoni, Ambra Pastori, Edoardo Giussani, Alice Fusaro, Isabella Monne, Calogero Terregino, Antonia Ricci
EPI_ISL_1914646, EPI_ISL_1914650	National Public Health Laboratory, National Centre for Infectious Diseases	National Public Health Laboratory, National Centre for Infectious Diseases	Tze Minn Mak, Zhenyang Zhou, Grace Jie Yin Ngan, Royce Ang, Lin Cui, Raymond Tzer Pin Lin
EPI_ISL_1918245	Laboratoire national de sante, Microbiology, Virology	Laboratoire national de sante, Microbiology, Microbial Genomics Platform	Anke Wienecke-Baldacchino, Catherine Ragimbeau,Jessica Tapp, Fatu Djabi, Lise Pignon, Raoul Salmon, Trung Nguyen Nguyen, Tamir Abdelrahman
EPI_ISL_1919379	Furst Medical Laboratory	Norwegian Institute of Public Health, Department of Virology	Kathrine Stene-Johansen, Kamilla Heddeland Instefjord, Hilde Elshaug, Garcia Llorente Ignacio, Jon Bråte, Engebretsen Serina Beate,Pedersen Benedikte Nevjen, Debech Nadia, Atiya R Ali,Marie Paulsen Madsen, Rasmus Riis Kopperud, Hilde Vollan, Karoline Bragstad, Olav Hungnes
EPI_ISL_1921894	OLVZ Aalst	OLVZ Aalst	Anne Vankeerberghen
EPI_ISL_1923538, EPI_ISL_1923583	Laboratorio Aziendale di Microbiologia e Virologia, Azienda Sanitaria dell'Alto Adige	Laboratorio Aziendale di Microbiologia e Virologia, Azienda Sanitaria dell'Alto Adige	Irene Bianconi, Bartolomeo Mobilio Rodriguez, Elisabetta Giacobazzi, Elisa Masi, Stefanie Wieser, Anne Picard, Claudia Volpato, Chiara Cantaloni, Elisabetta Pagani
EPI_ISL_1927302, EPI_ISL_1927303	SARS-CoV-2 testing team, National Institute of Infectious Diseases	Pathogen Genomics Center, National Institute of Infectious Diseases	Tsuyoshi Sekizuka, Kentaro Itokawa, Rina Tanaka, Masanori Hashino, Nozomu Hanaoka, Masumichi Saito, Naomi Nojiri, Hazuka Y Furihata, Sana Uchikoba, Tsuguto Fujimoto, Makoto Kuroda
EPI_ISL_1935392	LABORATOIRE DEPARTEMENTAL D'ANALYSES	CNR Virus des Infections Respiratoires - France SUD	Antonin Bal, Gregory Destras, Gwendolyne Burfin, Hadrien Regue, Quentin Semanas, Martine Valette, Bruno Lina, Laurence Josset
EPI_ISL_1935393	LIV VILLEFRANCHE	CNR Virus des Infections Respiratoires - France SUD	Antonin Bal, Gregory Destras, Gwendolyne Burfin, Hadrien Regue, Quentin Semanas, Martine Valette, Bruno Lina, Laurence Josset
EPI_ISL_1936189	MD PHL	MD PHL	Maryland Department of Health Laboratories Administration
EPI_ISL_1941525, EPI_ISL_1941566	Azienda Sanitaria dell'Alto Adige Laboratorio Aziendale di Microbiologia e Virologia	Istituto di Genomica Applicata	Elisabetta Pagani, Irene Bianconi, Elisabetta Giacobazzi, Elisa Masi, Stefanie Wieser, Irena Jurman, Vera Vendramin, Gabriele Magris, Eleonora Paparelli, Davide Scaglione, Michele Morgante
EPI_ISL_1942236, EPI_ISL_1942238, EPI_ISL_1942239, EPI_ISL_1942240	Laboratory Corporation of America	Centers for Disease Control and Prevention Division of Viral Diseases, Pathogen Discovery	Dakota Howard, Dhwani Batra, Peter W. Cook, Kara Moser, Adrian Paskey, Jason Caravas, Benjamin Rambo-Martin, Shatavia Morrison, Christopher Gulvick, Scott Sammons, Yvette Unoarumhi, Darlene Wagner, Matthew Schmerer, Minoo Agarwal, Eyad Almasri, Debbie Boles, Ayla Burns, Nuthawin Charoensri, Oren Cohen, Susan Countryman, Mary Ann Cristobal, Bobbi Croy, Suzanne Dale, Hrushikesh Deshmukh, Amanda Douglas, Vincent Drouillon, Marcia Eisenberg, Howard Engler, Rama Ghatti, Prashant Gupta, Susan Hicks, Jake Humphrey, Lax Iyer, Manoj Jain, Mohan Kolli, Brian Krueger, Tim Kuphal, Stanley Letovsky, Michael Levandoski, Craig Lukasik, Jonathan Meltzer, Brian Norvell, Mindy Nye, Scott Parker, Christos Petropoulos, John Pruitt, Steven Ragan, Scott Ryan, Mike Sapeta, Jana Schroth, Suresh Babu Selvaraju, Goran Stevovic, Amanda Suchanek, Andrea Throop, Lyndon Tilson, Thomas Urban, Joe Voshell, Kimberly Wagner, Jonathan Williams, Mary Williamson, Qian Zeng, Tricia Zwiefelhofer, Clinton R. Paden, Duncan MacCannell
EPI_ISL_1962937, EPI_ISL_1962938, EPI_ISL_1962939	Dutch COVID-19 response team	National Institute for Public Health and the Environment (RIVM)	Adam Meijer, Harry Vennema, Dirk Eggink, Jeroen Cremer, Sharon van den Brink, Bas van der Veer, AnneMarie van den Brandt, Lisa Wijsman, Kim Freriks, Ryanne Jaarsma, Euníce Then, Lynn Aarts, Sanne Bos, Melissa van Tuil, Linda van de Nes, Sjoerd Kuiling, James Groot, Florian Zwagemaker, Dennis Schmitz, Annelies Kroneman, Karim Hajji, Chantal Reusken, on behalf of the national COVID-19 response team

EPI_ISL_1963287, EPI_ISL_1963288, EPI_ISL_1963289, EPI_ISL_1963290	University Hospitals of Geneva, Laboratory of Virology	HUG, Laboratory of Virology and the Health2030 Genome Center	Samuel Cordey, Ana Rita Goncalves, Laurent Kaiser, Lorenzo Cerutti, Henri Pegeot, Melyssa Elies, Deborah Penet, Keith Harshman, Ioannis Xenarios, Emmanouil Dermitzakis
EPI_ISL_1970399	Centre for Dengue Research and AICBU, Department of Immunology and Molecular Medicine	Centre for Dengue Research and AICBU, Department of Immunology and Molecular Medicine	Chandima Jeewandara, Deshni Jayathilaka, Dinuka Ariyaratne, Deshan Madhusanka, Diyanath Ranasinghe, Laksiri Gomes, Gathsaurie Neelika Malavige
EPI_ISL_1990051	Laboratory Corporation of America	Centers for Disease Control and Prevention Division of Viral Diseases, Pathogen Discovery	Dakota Howard, Dhwani Batra, Peter W. Cook, Kara Moser, Adrian Paskey, Jason Caravas, Benjamin Rambo-Martin, Shatavia Morrison, Christopher Gulvick, Scott Sammons, Yvette Unoarumhi, Darlene Wagner, Matthew Schmerer, Minoo Agarwal, Eyad Almasri, Debbie Boles, Ayla Burns, Nuthawin Charoensri, Oren Cohen, Susan Countryman, Mary Ann Cristobal, Bobbi Croy, Suzanne Dale, Hrushikesh Deshmukh, Amanda Douglas, Vincent Drouillon, Marcia Eisenberg, Howard Engler, Rama Ghatti, Prashant Gupta, Susan Hicks, Jake Humphrey, Lax Iyer, Manoj Jain, Mohan Kolli, Brian Krueger, Tim Kuphal, Stanley Letovsky, Michael Levandoski, Craig Lukasik, Jonathan Meltzer, Brian Norvell, Mindy Nye, Scott Parker, Christos Petropoulos, John Pruitt, Steven Ragan, Scott Ryan, Mike Sapeta, Jana Schroth, Suresh Babu Selvaraju, Goran Stevovic, Amanda Suchanek, Andrea Throop, Lyndon Tilson, Thomas Urban, Joe Voshell, Kimberly Wagner, Jonathan Williams, Mary Williamson, Qian Zeng, Tricia Zwiefelhofer, Clinton R. Paden, Duncan MacCannell
EPI_ISL_1999837, EPI_ISL_1999851	Laboratori Clínic Territorial de Girona	Can Ruti SARS-CoV-2 Sequencing Hub (HUGTiP/IrsiCaixa/IGTP)	Marc Noguera-Julian, Pilar Armengol, Ignacio Blanco, Antoni E Bordoy, Francesc Catala-Moll, Pere-Joan Cardona, Maria Casadellà, Cristina Casañ, Gemma Clara, Bonaventura Clotet, Cristina Esteban, Montserrat Giménez, Mercedes Guerrero, Anna Not, Roger Paredes, Mariona Parera, Verónica Saludes, Alba Sánchez, and Elisa Martró on behalf of the Can Ruti SARS-CoV-2 Sequencing Hub.
EPI_ISL_2001059	PHV-FSS	PHV-FSS	Son Nguyen
EPI_ISL_2001420	SIESP DIPARTIMENTO DI PREVENZIONE TERAMO	Istituto Zooprofilattico Sperimentale dell'Abruzzo e Molise "G. Caporale"	Lorusso A, Marcacci M, Di Domenico M, Ancora M, Curini V, Di Lollo Valeria, Mangone I, Rinaldi A, Delli Compagni E, Scialabba S, Caporale M, Di Pasquale A, Cammà C, Puglia I, Calistri P, Savini G
EPI_ISL_2001431	Università degli Studi di Perugia	Istituto Zooprofilattico Sperimentale dell'Abruzzo e Molise "G. Caporale"	Mencacci A, Camilloni B, Lorusso A, Marcacci M, Di Domenico M, Ancora M, Curini V, Mangone I, Rinaldi A, Scialabba S, Di Pasquale A, Cammà C, Puglia I, Calistri P, Savini G
EPI_ISL_2001451, EPI_ISL_2001452	Laboratorio Analisi Osp. Città di Castello - Azienda USL Umbria1	Istituto Zooprofilattico Sperimentale dell'Abruzzo e Molise "G. Caporale"	Malagigi V, Tacconi P, Lorusso A, Marcacci M, Di Domenico M, Ancora M, Curini V, Mangone I, Rinaldi A, Scialabba S, Di Pasquale A, Cammà C, Puglia I, Calistri P, Savini G
EPI_ISL_2001459, EPI_ISL_2001460	Azienda Ospedaliera Terni	Istituto Zooprofilattico Sperimentale dell'Abruzzo e Molise "G. Caporale"	Palumbo M, Scaccetti A, Lorusso A, Marcacci M, Di Domenico M, Ancora M, Curini V, Mangone I, Rinaldi A, Scialabba S, Di Pasquale A, Cammà C, Puglia I, Calistri P, Savini G
EPI_ISL_2001524	Hospital of Southern Norway - Kristiansand, Department of Medical Microbiology	Norwegian Institute of Public Health, Department of Virology	Kathrine Stene-Johansen, Kamilla Heddeland Instefjord, Hilde Elshaug, Garcia Llorente Ignacio, Jon Bråte, Engebretsen Serina Beate,Pedersen Benedikte Nevjen, Debech Nadia, Atiya R Ali,Marie Paulsen Madsen, Rasmus Riis Kopperud, Hilde Vollan, Karoline Bragstad, Olav Hungnes
EPI_ISL_2001527	Furst Medical Laboratory	Norwegian Institute of Public Health, Department of Virology	Kathrine Stene-Johansen, Kamilla Heddeland Instefjord, Hilde Elshaug, Garcia Llorente Ignacio, Jon Bråte, Engebretsen Serina Beate,Pedersen Benedikte Nevjen, Debech Nadia, Atiya R Ali,Marie Paulsen Madsen, Rasmus Riis Kopperud, Hilde Vollan, Karoline Bragstad, Olav Hungnes
EPI_ISL_2002145, EPI_ISL_2002161, EPI_ISL_2002166, EPI_ISL_2002191, EPI_ISL_2002192, EPI_ISL_2002250, EPI_ISL_2002281, EPI_ISL_2002345, EPI_ISL_2002353, EPI_ISL_2002383, EPI_ISL_2002386, EPI_ISL_2002694, EPI_ISL_2002885, EPI_ISL_2002887, EPI_ISL_2002892, EPI_ISL_2002921			
see above	UW Virology Lab	UW Virology Lab	Pavitra Roychoudhury, Hong Xie, Lasata Shrestha, Tien V. Nguyen, Shah Mohamed Bakhash, Michelle Lin, Noah R. Baker, Sean Ellis, Meei-Li Huang, Keith R Jerome, Alexander Greninger
EPI_ISL_2003981	Basurto University Hospital: Clinical Microbiology Laboratory	Biocruces Bizkaia	Mikel Urrutikoetxea-Gutiérrez, Mª Carmen Nieto Toboso, Estibaliz Ugalde Zarraga, Mikel Gallego Rodrigo, Ana de la Hoz, José Luis Díaz de Tuesta del Arco
EPI_ISL_2004140	Instituto Nacional de Saude (INSA) and i3S - Instituto de Investigação e Inovação em Saúde	Instituto Nacional de Saude (INSA) and i3S - Instituto de Investigação e Inovação em Saúde	Borges et al
EPI_ISL_2004292	Instituto Nacional de Saude (INSA) and i3S - Instituto de Investigação e Inovação em Saúde	Instituto Nacional de Saude (INSA)	Borges et al
EPI_ISL_2006523	Lighthouse Lab in Milton Keynes	Wellcome Sanger Institute for the COVID-19 Genomics UK (COG-UK) Consortium	The Lighthouse Lab in Milton Keynes and Alex Alderton, Roberto Amato, Jeffrey Barrett, Sonia Goncalves, Ewan Harrison, David K. Jackson, Ian Johnston, Dominic Kwiatkowski, Cordelia Langford, John Sillitoe on behalf of the Wellcome Sanger Institute COVID-19 Surveillance Team
EPI_ISL_2006684	Max von Pettenkofer Institute, Virology, National Reference Center for Retroviruses, LMU Munich	Laboratory for Functional Genome Analysis; Dept. Genomics; Gene Center of the LMU Munich	Max Muenchhoff; Stefan Krebs; Alexander Graf; Oliver Keppler; Helmut Blum
EPI_ISL_2007318, EPI_ISL_2007330, EPI_ISL_2007331	Azienda Sanitaria dell'Alto Adige Laboratorio Aziendale di Microbiologia e Virologia	Istituto di Genomica Applicata	Elisabetta Pagani, Irene Bianconi, Elisabetta Giacobazzi, Elisa Masi, Stefanie Wieser, Irena Jurman, Vera Vendramin, Gabriele Magris, Eleonora Paparelli, Davide Scaglione, Michele Morgante
EPI_ISL_2015212	Minnesota Department of Health, Public Health Laboratory	Minnesota Department of Health, Public Health Laboratory	Alexandra Lorentz, Jacob Garfin, Matt Plumb, and Xiong Wang
EPI_ISL_2015899, EPI_ISL_2015920	SELARL MIRIALIS CLUSES	CNR Virus des Infections Respiratoires - France SUD	Antonin Bal, Gregory Destras, Gwendolyne Burfin, Hadrien Regue, Quentin Semanas, Martine Valette, Bruno Lina, Laurence Josset
EPI_ISL_2015999, EPI_ISL_2016006	MIRIALIS CLUSES BECHET	CNR Virus des Infections Respiratoires - France SUD	Antonin Bal, Gregory Destras, Gwendolyne Burfin, Hadrien Regue, Quentin Semanas, Martine Valette, Bruno Lina, Laurence Josset
EPI_ISL_2016154	CNR Virus des Infections Respiratoires - France SUD	CNR Virus des Infections Respiratoires - France SUD	Antonin Bal, Gregory Destras, Gwendolyne Burfin, Hadrien Regue, Quentin Semanas, Martine Valette, Bruno Lina, Laurence Josset
EPI_ISL_2020526	Istituto Zooprofilattico Sperimentale del Mezzogiorno	TIGEM	Antonio Grimaldi Patrizia Annunziata Francesco Panariello Biancamaria Pierri Claudia Tiberio Teresa Giuliano Valentina Bouche Chiara Colantuono Maria Concetta Cuomo Denise Di Concilio Lucio Di Filippo Anna Manfredi Marcello Salvi Antonio Limone Luigi Atripaldi Pellegrino Cerino Andrea Ballabio Davide Cacchiarelli
EPI_ISL_2021918	Lighthouse Lab in Alderley Park	Wellcome Sanger Institute for the COVID-19 Genomics UK (COG-UK) Consortium	Jacquelyn Wynn, Mairead Hyland, The Lighthouse Lab in Alderley Park and Alex Alderton, Roberto Amato, Jeffrey Barrett, Sonia Goncalves, Ewan Harrison, David K. Jackson, Ian Johnston, Dominic Kwiatkowski, Cordelia Langford, John Sillitoe on behalf of the Wellcome Sanger Institute COVID-19 Surveillance Team
EPI_ISL_2023710, EPI_ISL_2024170, EPI_ISL_2027877, EPI_ISL_2028587	Department of Virus and Microbiological Special Diagnostics, Statens Serum Institut, Copenhagen, Denmark	Aalborg University	Danish Covid-19 Genome Consortium
EPI_ISL_2029351, EPI_ISL_2029352	National Virus Reference Laboratory	National Virus Reference Laboratory	Zoe Yandle, Charlene Bennett, Gabriel Gonzalez, Michael Carr, Jonathan Dean, Cillian F De Gascun
EPI_ISL_2029366, EPI_ISL_2029441	Istituto Zooprofilattico Sperimentale del Mezzogiorno	TIGEM	Antonio Grimaldi Patrizia Annunziata Francesco Panariello Biancamaria Pierri Claudia Tiberio Teresa Giuliano Valentina Bouche Chiara Colantuono Maria Concetta Cuomo Denise Di Concilio Lucio Di Filippo Anna Manfredi Marcello Salvi Antonio Limone Luigi Atripaldi Pellegrino Cerino Andrea Ballabio Davide Cacchiarelli
EPI_ISL_2031506, EPI_ISL_2031507	UW Virology Lab	UW Virology Lab	Pavitra Roychoudhury, Hong Xie, Lasata Shrestha, Tien V. Nguyen, Shah Mohamed Bakhash, Michelle Lin, Noah R. Baker, Sean Ellis, Meei-Li Huang, Keith R Jerome, Alexander Greninger
EPI_ISL_2033624, EPI_ISL_2034597	Swedish national genomic surveillance program of SARS-CoV-2	The Public Health Agency of Sweden	Maximilian Riess, Maria Lind Karlberg, Alma Brolund, Swedish national genomic surveillance program of SARS-CoV-2
EPI_ISL_2037206, EPI_ISL_2037245, EPI_ISL_2037269	Pandemic Response Lab - NYC	Pandemic Response Lab, R&D	Henry Lee, Michael Hammerling, Melissa Hopkins, Cybill del Castillo, Shinyoung Clair Kang, William Ward, Pradeep Bugga, Sol Rey, Dylan Law, Katharine Nelson, Haiping Hao, Jon Laurent
EPI_ISL_2037519, EPI_ISL_2037520, EPI_ISL_2037658, EPI_ISL_2037669	UW Virology Lab	UW Virology Lab	Pavitra Roychoudhury, Hong Xie, Lasata Shrestha, Tien V. Nguyen, Shah Mohamed Bakhash, Michelle Lin, Noah R. Baker, Sean Ellis, Meei-Li Huang, Keith R Jerome, Alexander Greninger
EPI_ISL_2080296, EPI_ISL_2080312, EPI_ISL_2080341, EPI_ISL_2080346, EPI_ISL_2080348	Azienda Sanitaria dell'Alto Adige Laboratorio Aziendale di Microbiologia e Virologia	Istituto di Genomica Applicata	Elisabetta Pagani, Irene Bianconi, Elisabetta Giacobazzi, Elisa Masi, Stefanie Wieser, Irena Jurman, Vera Vendramin, Gabriele Magris, Eleonora Paparelli, Davide Scaglione, Michele Morgante
EPI_ISL_2082552	UW Virology Lab	UW Virology Lab	Pavitra Roychoudhury, Hong Xie, Lasata Shrestha, Tien V. Nguyen, Shah Mohamed Bakhash, Michelle Lin, Noah R. Baker, Sean Ellis, Meei-Li Huang,

			Keith R Jerome, Alexander Greninger
EPI_ISL_2086174	Microbiology Department. Complexo Hospitalario Universitario de Vigo	Microbiology Department. Complexo Hospitalario Universitario de Vigo	Alfaya N, Alonso I, Alvarez M, Cabrera JJ, Carballo R, Cores O, Cortizo S, del-Campo V, Martinez L, Mediero G, Perez S, Potel C, Regueiro B, Rey S, Vasallo FJ
EPI_ISL_2088160	National Virus Reference Laboratory	National Virus Reference Laboratory	Zoe Yandle, Charlene Bennett, Gabriel Gonzalez, Michael Carr, Jonathan Dean, Cillian F De Gascun
EPI_ISL_2091931	Lighthouse Lab in Alderley Park	Wellcome Sanger Institute for the COVID-19 Genomics UK (COG-UK) Consortium	Jacquelyn Wynn, Mairead Hyland, The Lighthouse Lab in Alderley Park and Alex Alderton, Roberto Amato, Jeffrey Barrett, Sonia Goncalves, Ewan Harrison, David K. Jackson, Ian Johnston, Dominic Kwiatkowski, Cordelia Langford, John Sillitoe on behalf of the Wellcome Sanger Institute COVID-19 Surveillance Team
EPI_ISL_2094351	Dutch COVID-19 response team	National Institute for Public Health and the Environment (RIVM)	Adam Meijer, Harry Vennema, Dirk Eggink, Jeroen Cremer, Sharon van den Brink, Bas van der Veer, AnneMarie van den Brandt, Lisa Wijsman, Kim Freriks, Ryanne Jaarsma, Euníce Then, Lynn Aarts, Sanne Bos, Melissa van Tuil, Linda van de Nes, Sjoerd Kuiling, James Groot, Florian Zwagemaker, Dennis Schmitz, Annelies Kroneman, Karim Hajji, Chantal Reusken, on behalf of the national COVID-19 response team
EPI_ISL_2094436	cerballiance-IDF	Cerba lab	Emmanuel Lecorche, Sabine Trombert-Paolantoni, Aude Lessenne, Kader Merah, Thierry Collin, Laura Verdurme, Patrice Herisson, Stéphanie Haïm-Boukobza, Bénédicte Roquebert
EPI_ISL_2104621, EPI_ISL_2104664	University Hospitals of Geneva, Laboratory of Virology	HUG, Laboratory of Virology and the Health2030 Genome Center	Samuel Cordey, Ana Rita Goncalves, Laurent Kaiser, Lorenzo Cerutti, Henri Pegeot, Melyssa Elies, Deborah Penet, Keith Harshman, Ioannis Xenarios, Emmanouil Dermitzakis
EPI_ISL_2105968	Respiratory Viruses Branch, Centers for Disease Control and Prevention	Respiratory Viruses Branch, Centers for Disease Control and Prevention	Howard,D., Batra,D., Cook,P.W., Moser,K., Paskey,A., Caravas,J., Rambo-Martin,B., Morrison,S., Gulvick,C., Sammons,S., Unoarumhi,Y., Wagner,D., Roychoudhury,M.S., Xie,H., Shrestha,L., Nguyen,T.V., Bakhash,S.M., Lin,M., Baker,N.R., Perez,R., Ellis,S., Breit,N., Livingston,R.J., Huang,M.-L., Jerome,K.R., Mathias,P., Greninger,A., Paden,C.R., MacCannell,D.
EPI_ISL_2106358, EPI_ISL_2106547, EPI_ISL_2106548	Pandemic Response Lab - NYC	Pandemic Response Lab, R&D	Henry Lee, Michael Hammerling, Melissa Hopkins, Cybill del Castillo, Shinyoung Clair Kang, William Ward, Pradeep Bugga, Sol Rey, Dylan Law, Katharine Nelson, Haiping Hao, Jon Laurent
EPI_ISL_2115393	Kath. St.-Johannes-Gesellschaft Dortmund gGmbH	Robert Koch Institute	unknown
EPI_ISL_2115400, EPI_ISL_2115409, EPI_ISL_2115485, EPI_ISL_2115495	MVZ Dr. Eberhard & Partner Dortmund	Robert Koch Institute	unknown
EPI_ISL_2120312, EPI_ISL_2120318, EPI_ISL_2120327, EPI_ISL_2120333, EPI_ISL_2120335, EPI_ISL_2120341, EPI_ISL_2120351, EPI_ISL_2120427, EPI_ISL_2120481, EPI_ISL_2120502	MDI Limbach Berlin GmbH; MVZ Labor Berlin	Robert Koch Institute	unknown
EPI_ISL_2120555	MVZ fur Laboratoriumsmedizin und Mikrobiologie Koblenz-Mittelrhein (Labor Koblenz)	Robert Koch Institute	unknown
EPI_ISL_2120568	MDI Limbach Berlin GmbH; MVZ Labor Berlin	Robert Koch Institute	unknown
EPI_ISL_2122436	MVZ Dr. Eberhard & Partner Dortmund	Robert Koch Institute	unknown
EPI_ISL_2122454	Laborarztpraxis Osnabruck	Robert Koch Institute	unknown
EPI_ISL_2122539, EPI_ISL_2122544, EPI_ISL_2122558	MVZ Dr. Eberhard & Partner Dortmund	Robert Koch Institute	unknown
EPI_ISL_2122732	MDI Limbach Berlin GmbH; MVZ Labor Berlin	Robert Koch Institute	unknown
EPI_ISL_2123425	MVZ fur Laboratoriumsmedizin und Mikrobiologie Koblenz-Mittelrhein (Labor Koblenz)	Robert Koch Institute	unknown
EPI_ISL_2125050	MDI Limbach Berlin GmbH; MVZ Labor Berlin	Robert Koch Institute	unknown
EPI_ISL_2125094, EPI_ISL_2125110	MVZ fur Laboratoriumsmedizin und Mikrobiologie Koblenz-Mittelrhein (Labor Koblenz)	Robert Koch Institute	unknown
EPI_ISL_2125114	Labor Prof. Dr. G. Enders MVZ GbR	Robert Koch Institute	unknown
EPI_ISL_2128961	MDI Limbach Berlin GmbH; MVZ Labor Berlin	Robert Koch Institute	unknown
EPI_ISL_2128966	MVZ fur Laboratoriumsmedizin und Mikrobiologie Koblenz-Mittelrhein (Labor Koblenz)	Robert Koch Institute	unknown
EPI_ISL_2128971	MDI Limbach Berlin GmbH; MVZ Labor Berlin	Robert Koch Institute	unknown
EPI_ISL_2128973	MVZ fur Laboratoriumsmedizin und Mikrobiologie Koblenz-Mittelrhein (Labor Koblenz)	Robert Koch Institute	unknown
EPI_ISL_2128975, EPI_ISL_2128976	MDI Limbach Berlin GmbH; MVZ Labor Berlin	Robert Koch Institute	unknown
EPI_ISL_2128980, EPI_ISL_2128983	MVZ fur Laboratoriumsmedizin und Mikrobiologie Koblenz-Mittelrhein (Labor Koblenz)	Robert Koch Institute	unknown
EPI_ISL_2128988	MDI Limbach Berlin GmbH; MVZ Labor Berlin	Robert Koch Institute	unknown
EPI_ISL_2129577, EPI_ISL_2129603	MVZ Dr. Eberhard & Partner Dortmund	Robert Koch Institute	unknown
EPI_ISL_2129605	Medizinisches Labor Wahl LÃ¼denscheid	Robert Koch Institute	unknown
EPI_ISL_2129608	MVZ Dr. Eberhard & Partner Dortmund	Robert Koch Institute	unknown
EPI_ISL_2131459, EPI_ISL_2131460, EPI_ISL_2131462, EPI_ISL_2131484	Università degli Studi di Perugia	Istituto Zooprofilattico Sperimentale dell'Abruzzo e Molise "G. Caporale"	Mencacci A, Camilloni B, Lorusso A, Marcacci M, Di Domenico M, Ancora M, Curini V, Mangone I, Rinaldi A, Delli Compagni E, Scialabba S, Di Pasquale A, Cammà C, Puglia I, Calistri P, Savini G
EPI_ISL_2131887, EPI_ISL_2131927, EPI_ISL_2131933, EPI_ISL_2132118	National Virus Reference Laboratory	National Virus Reference Laboratory	Zoe Yandle, Charlene Bennett, Gabriel Gonzalez, Michael Carr, Jonathan Dean, Cillian F De Gascun
EPI_ISL_2136915	Laboratori Clínic Territorial de Girona	Can Ruti SARS-CoV-2 Sequencing Hub (HUGTiP/IrsiCaixa/IGTP)	Marc Noguera-Julian, Pilar Armengol, Ignacio Blanco, Antoni E Bordoy, Francesc Catala-Moll, Pere-Joan Cardona, Maria Casadellà, Cristina Casañ, Gemma Clara, Bonaventura Clotet, Cristina Esteban, Montserrat Giménez, Mercedes Guerrero, Anna Not, Roger Paredes, Mariona Parera, Verónica Saludes, Alba Sánchez, and Elisa Martró on behalf of the Can Ruti SARS-CoV-2 Sequencing Hub.
EPI_ISL_2137592	Istituto Zooprofilattico Sperimentale del Mezzogiorno	TIGEM	Antonio Grimaldi Patrizia Annunziata Francesco Panariello Biancamaria Pierri Claudia Tiberio Teresa Giuliano Valentina Bouche Chiara Colantuono Maria Concetta Cuomo Denise Di Concilio Lucio Di Filippo Anna Manfredi Marcello Salvi Antonio Limone Luigi Atripaldi Pellegrino Cerino Andrea Ballabio Davide Cacchiarelli
EPI_ISL_2137768	Università degli Studi di Perugia	Istituto Zooprofilattico Sperimentale dell'Abruzzo e Molise "G. Caporale"	Mencacci A, Camilloni B, Lorusso A, Marcacci M, Di Domenico M, Ancora M, Curini V, Mangone I, Rinaldi A, Delli Compagni E, Scialabba S, Di Pasquale A, Cammà C, Puglia I, Calistri P, Savini G
EPI_ISL_2138808	Ostfold Hospital Trust - Kalnes, Centre for Laboratory Medicine, Section for gene technology and infection serology	Norwegian Institute of Public Health, Department of Virology	Kathrine Stene-Johansen, Kamilla Heddeland Instefjord, Hilde Elshaug, Garcia Llorente Ignacio, Jon Bråte, Engebretsen Serina Beate,Pedersen Benedikte Nevjen, Debech Nadia, Atiya R Ali,Marie Paulsen Madsen, Rasmus Riis Kopperud, Hilde Vollan, Karoline Bragstad, Olav Hungnes
EPI_ISL_2140548	Laboratori Clínic Territorial de Girona	Can Ruti SARS-CoV-2 Sequencing Hub	Marc Noguera-Julian, Pilar Armengol, Ignacio Blanco, Antoni E Bordoy, Francesc Catala-Moll, Pere-Joan Cardona, Maria Casadellà, Cristina Casañ,

		(HUGTiP/IrsiCaixa/IGTP)	Gemma Clara, Bonaventura Clotet, Cristina Esteban, Montserrat Giménez, Mercedes Guerrero, Anna Not, Roger Paredes, Mariona Parera, Verónica Saludes, Alba Sánchez, and Elisa Martró on behalf of the Can Ruti SARS-CoV-2 Sequencing Hub.
EPI_ISL_2140632	Microbiology Department, Laboratori Clínic Metropolitana Nord. Hospital Universitari Germans Trias i Pujol	Can Ruti SARS-CoV-2 Sequencing Hub (HUGTiP/IrsiCaixa/IGTP)	Marc Noguera-Julian, Pilar Armengol, Ignacio Blanco, Antoni E Bordoy, Francesc Catala-Moll, Pere-Joan Cardona, Maria Casadellà, Cristina Casañ, Gemma Clara, Bonaventura Clotet, Cristina Esteban, Montserrat Giménez, Mercedes Guerrero, Anna Not, Roger Paredes, Mariona Parera, Verónica Saludes, Alba Sánchez, and Elisa Martró on behalf of the Can Ruti SARS-CoV-2 Sequencing Hub.
EPI_ISL_2141965	BIOR	Latvian Biomedical Research and Study Centre	Janis Pjalkovskis, Nikita Zrelovs, Monta Ustinova, Ivars Silamikelis, Liga Birzniece, Kaspars Megnis, Una Krumina, Guntars Zarins, Vita Rovite, Lauma Freimane, Laila Silamikele, Laura Ansone, Davids Fridmanis, Elina Dimina, Irena Meistere, Daina Pule, Jurijs Perevoscikovs, Uga Dumpis, Janis Klovins
EPI_ISL_2142669	LAM COLONNA DE CINARCA	CNR Virus des Infections Respiratoires - France SUD	Antonin Bal, Gregory Destras, Gwendolyne Burfin, Hadrien Regue, Quentin Semanas, Martine Valette, Bruno Lina, Laurence Josset
EPI_ISL_2143400, EPI_ISL_2143519	CH TOURCOING	CHU Lille - Laboratoire de Virologie	AIT YAHYA Emilie, ALIDJINOU Enagnon Kazali, BOCKET Laurence, CREPIN Michel, DEMAY Christophe, ENGELMANN Ilka, GEFFROY Sandrine, GUIGON Aurélie, LAMBERT Valérie, LAZREK Mouna, NOBILLIAUX Florian, PREVOST Brigitte, THUILLIER Caroline, TINEZ Claire
EPI_ISL_2143688, EPI_ISL_2143703	Istituto Zooprofilattico Sperimentale del Mezzogiorno	Telethon Institute of Genetics and Medicine (TIGEM)	Antonio Grimaldi Patrizia Annunziata Francesco Panariello Biancamaria Pierri Claudia Tiberio Teresa Giuliano Valentina Bouche Chiara Colantuono Maria Concetta Cuomo Denise Di Concilio Lucio Di Filippo Anna Manfredi Marcello Salvi Antonio Limone Luigi Atripaldi Pellegrino Cerino Andrea Ballabio Davide Cacchiarelli
EPI_ISL_2151335	Basurto University Hospital Clinical Microbiology Laboratory	Biocruces Bizkaia	Mikel Urrutikoetxea-Gutierrez, Mª Carmen Nieto Toboso, Estibaliz Ugalde Zarraga, Mikel Gallego Rodrigo, Ana de la Hoz, José Luis Díaz de Tuesta del Arco
EPI_ISL_2153172, EPI_ISL_2153173	Istituto Zooprofilattico Sperimentale del Mezzogiorno	TIGEM	Antonio Grimaldi Patrizia Annunziata Francesco Panariello Biancamaria Pierri Claudia Tiberio Teresa Giuliano Valentina Bouche Chiara Colantuono Maria Concetta Cuomo Denise Di Concilio Lucio Di Filippo Anna Manfredi Marcello Salvi Antonio Limone Luigi Atripaldi Pellegrino Cerino Andrea Ballabio Davide Cacchiarelli
EPI_ISL_2153429	Fimlab Laboratories	Fimlab Laboratories	Minna Paloniemi, Leena Huhti, Sara Lehtinen, Bruno Luukinen, Tapio Seiskari, Mauri Keinänen
EPI_ISL_2156070	Laboratorio Aziendale di Microbiologia e Virologia, Azienda Sanitaria dell'Alto Adige	Laboratorio Aziendale di Microbiologia e Virologia, Azienda Sanitaria dell'Alto Adige	Irene Bianconi, Bartolomeo Mobilio Rodriguez, Elisabetta Giacobazzi, Elisa Masi, Stefanie Wieser, Anne Picard, Claudia Volpato, Chiara Cantaloni, Elisabetta Pagani
EPI_ISL_2156768	Centre de Recherches Médicales de Lambaréné (CERMEL)	Centre de Recherches Médicales de Lambaréné (CERMEL)	Gédéon Prince Manouana, Moustapha Nzamba Maloum, Sam O'neilla Oye Bingono, Georgelin Nguema Ondo, Rodrigue Bikangui, Samira Zoa Assoumou, Srinivas reddy Pallerla, Jean Bernard Lekana-Douki, Joël-Fleury Djoba Siawaya, Steffen Borrmann, Thirumalaisamy P. Velavan, Bertrand Lell and Ayola Akim Adegnika
EPI_ISL_2156841	CSIR-Centre for Cellular and Molecular Biology	CSIR-Centre for Cellular and Molecular Biology-INSACOG	Tulasi Nagabandi, Lamuk Zaveri,Ara Sreenivas,Shreekant Verma, Amareshwar Vodapalli ,Blessy B John,Viswagithe S L,B Himasri,Valli Nagalakshmi Undamatla,Payel Mukherjee,Sofia Banu,Archana Bharadwaj Siva,Sharath Chandra Thota,Karthik Bharadwaj Tallapaka,Rakesh K Mishra,Divya Tej Sowpati
EPI_ISL_2156972	Mapmygenome	CSIR-Centre for Cellular and Molecular Biology-INSACOG	Ara Sreenivas,Shreekant Verma,Tulasi Nagabandi, Lamuk Zaveri, Amareshwar Vodapalli ,Blessy B John,Viswagithe S L,B Himasri,Valli Nagalakshmi Undamatla,Payel Mukherjee,Sofia Banu,Archana Bharadwaj Siva,Sharath Chandra Thota,Karthik Bharadwaj Tallapaka,Rakesh K Mishra,Divya Tej Sowpati
EPI_ISL_2157073	Mapmygenome	CSIR-Centre for Cellular and Molecular Biology-INSACOG	Payel Mukherjee,Lamuk Zaveri,Tulasi Nagabandi, Ara Sreenivas,Shreekant Verma, Amareshwar Vodapalli ,Blessy B John,Viswagithe S L,B Himasri,Valli Nagalakshmi Undamatla,Sofia Banu,Archana Bharadwaj Siva,Sharath Chandra Thota,Karthik Bharadwaj Tallapaka,Rakesh K Mishra,Divya Tej Sowpati
EPI_ISL_2157166	Mapmygenome	CSIR-Centre for Cellular and Molecular Biology - INSACOG	Ara Sreenivas, Onkar Kulkarni, Lamuk Zaveri, Sofia Banu, Shreekant Verma, Amareshwar Vodapalli , Viswagithe S L, B Himasri, Sharath Chandra Thota, Karthik Bharadwaj Tallapaka, Rakesh K Mishra,Divya Tej Sowpati
EPI_ISL_2164238	Basurto University Hospital: Clinical Microbiology Laboratory	Biocruces Bizkaia	Mikel Urrutikoetxea-Gutiérrez, Mª Carmen Nieto Toboso, Estibaliz Ugalde Zarraga, Mikel Gallego Rodrigo, Ana de la Hoz, José Luis Díaz de Tuesta del Arco
EPI_ISL_2164421	Basurto University Hospital: Clinical Microbiology Laboratory	Biocruces Bizkaia	Basurto University Hospital: Clinical Microbiology Laboratory
EPI_ISL_2168688, EPI_ISL_2168693, EPI_ISL_2168694, EPI_ISL_2168696, EPI_ISL_2168697, EPI_ISL_2168698, EPI_ISL_2168699, EPI_ISL_2168700, EPI_ISL_2168702, EPI_ISL_2168705, EPI_ISL_2168707, EPI_ISL_2168708, EPI_ISL_2168709, EPI_ISL_2168711, EPI_ISL_2168712, EPI_ISL_2168714, EPI_ISL_2168715, EPI_ISL_2168716, EPI_ISL_2168717, EPI_ISL_2168718, EPI_ISL_2168721, EPI_ISL_2168723, EPI_ISL_2168724, EPI_ISL_2168725, EPI_ISL_2168728, EPI_ISL_2168730, EPI_ISL_2168741, EPI_ISL_2168746, EPI_ISL_2169255, EPI_ISL_2169256, EPI_ISL_2169258, EPI_ISL_2169266, EPI_ISL_2169274, EPI_ISL_2169280, EPI_ISL_2169281, EPI_ISL_2169283, EPI_ISL_2169285, EPI_ISL_2169289, EPI_ISL_2169290, EPI_ISL_2169294, EPI_ISL_2169297, EPI_ISL_2169309, EPI_ISL_2169365, EPI_ISL_2169499, EPI_ISL_2169505, EPI_ISL_2169515, EPI_ISL_2169519, EPI_ISL_2169527, EPI_ISL_2169540, EPI_ISL_2169544, EPI_ISL_2169654, EPI_ISL_2169655, EPI_ISL_2169656, EPI_ISL_2169663, EPI_ISL_2169670, EPI_ISL_2169894, EPI_ISL_2169911, EPI_ISL_2169926			
see above	Alberta Precision Labs (APL)	Public Health Agency of Canada (PHAC) National Microbiology Laboratory	Buss, E, Croxen M, Deo A, Dieu P, Gill K, Ferrato C, Khan F, Koleva P, Li V, Lloyd C, Lynch T, Ma R, Murphy S, Pabbaraju K, Shokoples S, Tipples G, Thayer J, Whitehouse M, Wong A, Yu C, Zelyas N
EPI_ISL_2171315, EPI_ISL_2171556	Istituto Zooprofilattico Sperimentale del Mezzogiorno	TIGEM	Antonio Grimaldi Patrizia Annunziata Francesco Panariello Biancamaria Pierri Claudia Tiberio Teresa Giuliano Valentina Bouche Chiara Colantuono Maria Concetta Cuomo Denise Di Concilio Lucio Di Filippo Anna Manfredi Marcello Salvi Antonio Limone Luigi Atripaldi Pellegrino Cerino Andrea Ballabio Davide Cacchiarelli
EPI_ISL_2171878, EPI_ISL_2171984	Lighthouse Lab in Alderley Park	Wellcome Sanger Institute for the COVID-19 Genomics UK (COG-UK) Consortium	Jacquelyn Wynn, Mairead Hyland, The Lighthouse Lab in Alderley Park and Alex Alderton, Roberto Amato, Jeffrey Barrett, Sonia Goncalves, Ewan Harrison, David K. Jackson, Ian Johnston, Dominic Kwiatkowski, Cordelia Langford, John Sillitoe on behalf of the Wellcome Sanger Institute COVID-19 Surveillance Team
EPI_ISL_2178480	Labo analyses med	National Reference Center for Viruses of Respiratory Infections, Institut Pasteur, Paris	Marion Barbet, Sylvie Behillil, Méline Bizard, Angela Brisebarre, Camille Capel, Vincent Enouf, Louise Lefrançois, Frédéric Lemoine, Christophe Malabat, Corinne Maufrais, Amaury Vaysse, Etienne Simon-Lorière, Maud Vanpeene, Sylvie Van der Werf ,Jean-FrançOis Comes
EPI_ISL_2178832	Hospital General Universitario Gregorio Marañón	Hospital General Universitario Gregorio Marañón	Sergio Buenestado Serrano, Pedro Sola Campoy, Laura Pérez-Lago, Cristina Rodriguez-Grande, Marta Herranz Martin, Victor de la Cueva, Pilar Catalán, Patricia Muñoz, Darío García de Viedma
EPI_ISL_2179503	SELAS BC-LAB	Department of Virology, Henri Mondor University Hospital, Assistance Publique Hôpitaux de Paris, Université Paris-Est Créteil, INSERM U955	Christophe Rodriguez, Slim Fourati, Vanessa Demontant, Guillaume Gricourt, Melissa N'Debi, Alexandre Soulier, Elisabeth Trawinski, Jean-Michel Pawlotsky
EPI_ISL_2179747, EPI_ISL_2179748, EPI_ISL_2179749	Servicio Microbiología Hospital La Paz	Servicio Microbiología Hospital La Paz	Fernando Lázaro, Rubén Cáceres, Jesús Mingorance Cruz, Elie Dahdouh
EPI_ISL_2188356	Labo Analyses Med	National Reference Center for Viruses of Respiratory Infections, Institut Pasteur, Paris	Marion Barbet, Sylvie Behillil, Méline Bizard, Angela Brisebarre, Camille Capel, Vincent Enouf, Louise Lefrançois, Frédéric Lemoine, Christophe Malabat, Corinne Maufrais, Damien Mornico, Etienne Simon-Lorière, Maud Vanpeene, Sylvie Van der Werf ,Franck Ennouchi
EPI_ISL_2188485	Labo Analyses Med	National Reference Center for Viruses of Respiratory Infections, Institut Pasteur, Paris	Marion Barbet, Sylvie Behillil, Méline Bizard, Angela Brisebarre, Camille Capel, Vincent Enouf, Louise Lefrançois, Frédéric Lemoine, Christophe Malabat, Corinne Maufrais, Gael Millot, Etienne Simon-Lorière, Maud Vanpeene, Sylvie Van der Werf ,Adamou Lagare
EPI_ISL_2193398, EPI_ISL_2193399	University Hospitals Translational Laboratory (UHTL), University Hospitals	University Hospitals Translational Laboratory (UHTL), University Hospitals	Sadri,N., Alouani,D., Song,X.
EPI_ISL_2193872	National Virus Reference Laboratory	National Virus Reference Laboratory	Zoe Yandle, Charlene Bennett, Gabriel Gonzalez, Michael Carr, Jonathan Dean, Cillian F De Gascun
EPI_ISL_2194359, EPI_ISL_2194447, EPI_ISL_2194467	Istituto Zooprofilattico Sperimentale del Mezzogiorno	TIGEM	Antonio Grimaldi Patrizia Annunziata Francesco Panariello Biancamaria Pierri Claudia Tiberio Teresa Giuliano Valentina Bouche Chiara Colantuono Maria Concetta Cuomo Denise Di Concilio Lucio Di Filippo Anna Manfredi Marcello Salvi Antonio Limone Luigi Atripaldi Pellegrino Cerino Andrea Ballabio Davide Cacchiarelli
EPI_ISL_2195785, EPI_ISL_2195786, EPI_ISL_2195787, EPI_ISL_2195792, EPI_ISL_2195794, EPI_ISL_2195797, EPI_ISL_2195800, EPI_ISL_2195801, EPI_ISL_2195803, EPI_ISL_2195804, EPI_ISL_2195868, EPI_ISL_2195873, EPI_ISL_2195877			
see above	Berkeley Medical Center	WVU and Marshall University Combined Genomics Core Facilities	James Denvir, Peter Stoilov, Peter Perrotta, Wesley Kimble, Ryan Percifield
EPI_ISL_2196064, EPI_ISL_2196067,	BMC LAB	WVU and Marshall University Combined Genomics Core	James Denvir, Peter Stoilov, Peter Perrotta, Wesley Kimble, Ryan Percifield

EPI_ISL_2196070		Facilities	
EPI_ISL_2196111	West Virigina Labs	WVU and Marshall University Combined Genomics Core Facilities	James Denvir, Peter Stoilov, Peter Perrotta, Wesley Kimble, Ryan Percifield
EPI_ISL_2196170	QLabs	WVU and Marshall University Combined Genomics Core Facilities	James Denvir, Peter Stoilov, Peter Perrotta, Wesley Kimble, Ryan Percifield
EPI_ISL_2199343	Lighthouse Lab in Alderley Park	Wellcome Sanger Institute for the COVID-19 Genomics UK (COG-UK) Consortium	Jacquelyn Wynn, Mairead Hyland, The Lighthouse Lab in Alderley Park and Alex Alderton, Roberto Amato, Jeffrey Barrett, Sonia Goncalves, Ewan Harrison, David K. Jackson, Ian Johnston, Dominic Kwiatkowski, Cordelia Langford, John Sillitoe on behalf of the Wellcome Sanger Institute COVID-19 Surveillance Team
EPI_ISL_2202386, EPI_ISL_2202387	Ministry of Health Turkey	Ministry of Health Turkey	Fatma Bayrakdar, Yasemin Cosgun, Suleyman Yalcin, Gulay Korukluoglu
EPI_ISL_2204356, EPI_ISL_2204389, EPI_ISL_2204392	BMC LAB	WVU and Marshall University Combined Genomics Core Facilities	James Denvir, Peter Stoilov, Peter Perrotta, Wesley Kimble, Ryan Percifield
EPI_ISL_2204409, EPI_ISL_2204413, EPI_ISL_2204414, EPI_ISL_2204424, EPI_ISL_2204425, EPI_ISL_2204431, EPI_ISL_2204438, EPI_ISL_2204444, EPI_ISL_2204459, EPI_ISL_2204472, EPI_ISL_2204478, EPI_ISL_2204489, EPI_ISL_2204512, EPI_ISL_2204514			
see above	Berkeley Medical Center	WVU and Marshall University Combined Genomics Core Facilities	James Denvir, Peter Stoilov, Peter Perrotta, Wesley Kimble, Ryan Percifield
EPI_ISL_2204735	BMC LAB	WVU and Marshall University Combined Genomics Core Facilities	James Denvir, Peter Stoilov, Peter Perrotta, Wesley Kimble, Ryan Percifield
EPI_ISL_2204748, EPI_ISL_2204756, EPI_ISL_2204776, EPI_ISL_2204823	Berkeley Medical Center	WVU and Marshall University Combined Genomics Core Facilities	James Denvir, Peter Stoilov, Peter Perrotta, Wesley Kimble, Ryan Percifield
EPI_ISL_2213595	University Hospitals of Geneva, Laboratory of Virology	HUG, Laboratory of Virology and the Health2030 Genome Center	Samuel Cordey, Ana Rita Goncalves, Laurent Kaiser, Lorenzo Cerutti, Henri Pegeot, Melyssa Elies, Deborah Penet, Keith Harshman, Ioannis Xenarios, Emmanouil Dermitzakis
EPI_ISL_2225610, EPI_ISL_2225611	Basurto University Hospital: Clinical Microbiology Laboratory	Biocruces Bizkaia	Mikel Urrutikoetxea-Gutiérrez, Mª Carmen Nieto Toboso, Estibaliz Ugalde Zarraga, Mikel Gallego Rodrigo, Ana de la Hoz, José Luis Díaz de Tuesta del Arco
EPI_ISL_2226117	Furst Medical Laboratory	Norwegian Institute of Public Health, Department of Virology	Kathrine Stene-Johansen, Kamilla Heddeland Instefjord, Hilde Elshaug, Garcia Llorente Ignacio, Jon Bråte, Engebretsen Serina Beate,Pedersen Benedikte Nevjen, Debech Nadia, Atiya R Ali,Marie Paulsen Madsen, Rasmus Riis Kopperud, Hilde Vollan, Karoline Bragstad, Olav Hungnes
EPI_ISL_2226754, EPI_ISL_2226755, EPI_ISL_2226759	ADMED Microbiologie	Genomics and Transcriptomics, Philip Morris International	Reto Lienhard, Marie-Lise Tritten, Emmanuel Guedj, Nicolas Sierro, Rémi Dulize, David Bornand, Mehdi Auberson, Maxime Berthouzoz, Nikolai Ivanov, Manuel Peitsch
EPI_ISL_2227039, EPI_ISL_2227044, EPI_ISL_2227048, EPI_ISL_2227064, EPI_ISL_2227070, EPI_ISL_2227074	Università Federico II - Dipartimento di scienze mediche traslazionali - Napoli	TIGEM	Antonio Grimaldi Patrizia Annunziata Francesco Panariello Teresa Giuliano Michele Cennamo Valentina Bouche Chiara Colantuono Lucio Di Filippo Mariano Fiorenza Anna Manfredi Marcello Salvi Giuseppe Portella Andrea Ballabio Davide Cacchiarelli
EPI_ISL_2227095	Istituto Zooprofilattico Sperimentale del Mezzogiorno	TIGEM	Antonio Grimaldi Patrizia Annunziata Francesco Panariello Biancamaria Pierri Claudia Tiberio Teresa Giuliano Valentina Bouche Chiara Colantuono Maria Concetta Cuomo Denise Di Concilio Lucio Di Filippo Anna Manfredi Marcello Salvi Antonio Limone Luigi Atripaldi Pellegrino Cerino Andrea Ballabio Davide Cacchiarelli
EPI_ISL_2235210	Pandemic Response Lab - NYC	Pandemic Response Lab, R&D	Henry Lee, Michael Hammerling, Melissa Hopkins, Cybill del Castillo, Shinyoung Clair Kang, William Ward, Pradeep Bugga, Sol Rey, Dylan Law, Katharine Nelson, Haiping Hao, Jon Laurent
EPI_ISL_2235459	Università degli Studi di Perugia	Istituto Zooprofilattico Sperimentale dell'Abruzzo e Molise "G. Caporale"	Mencacci A, Camilloni B, Lorusso A, Marcacci M, Di Domenico M, Ancora M, Curini V, Di Lollo Valeria, Mangone I, Rinaldi A, Delli Compagni E, Scialabba S, Caporale M, Di Pasquale A, Cammà C, Puglia I, Calistri P, Savini G
EPI_ISL_2236335, EPI_ISL_2236373, EPI_ISL_2237742	Lighthouse Lab in Alderley Park	Wellcome Sanger Institute for the COVID-19 Genomics UK (COG-UK) Consortium	Jacquelyn Wynn, Mairead Hyland, The Lighthouse Lab in Alderley Park and Alex Alderton, Roberto Amato, Jeffrey Barrett, Sonia Goncalves, Ewan Harrison, David K. Jackson, Ian Johnston, Dominic Kwiatkowski, Cordelia Langford, John Sillitoe on behalf of the Wellcome Sanger Institute COVID-19 Surveillance Team
EPI_ISL_2239501	Northumbria University / South Tees Hospitals NHS Foundation Trust / North Cumbria Integrated Care NHS Foundation Trust / North Tees and Hartlepool NHS Foundation Trust / Newcastle Hospitals NHS Foundation Trust	COVID-19 Genomics UK (COG-UK) Consortium	Darren L Smith,Andrew Nelson,Matthew Bashton,Greg R Young,Joshua Loh,John Allan,Mohammad A Tariq,Giles S Holt,Gary Black,Wen C Yew,Lynn Dover,Paul Baker,Steve Liggett,Sarah Essex,Jane Greenaway,Debra Padgett,Clive Graham,Garren Scott,Edward Barton,Emma Swindells,Brendan Payne,Jennifer Collins,Yusri Taha,Gary Eltringham
EPI_ISL_2240830, EPI_ISL_2240985, EPI_ISL_2241015, EPI_ISL_2241025, EPI_ISL_2241138, EPI_ISL_2241212, EPI_ISL_2241259, EPI_ISL_2241309	National Virus Reference Laboratory	National Virus Reference Laboratory	Zoe Yandle, Charlene Bennett, Gabriel Gonzalez, Michael Carr, Jonathan Dean, Cillian F De Gascun
EPI_ISL_2241690, EPI_ISL_2241691, EPI_ISL_2241692, EPI_ISL_2241693	Laboratory Corporation of America	Centers for Disease Control and Prevention Division of Viral Diseases, Pathogen Discovery	Dakota Howard, Dhwani Batra, Peter W. Cook, Kara Moser, Adrian Paskey, Jason Caravas, Benjamin Rambo-Martin, Shatavia Morrison, Christopher Gulvick, Scott Sammons, Yvette Unoarumhi, Darlene Wagner, Matthew Schmerer, Minoo Agarwal, Eyad Almasri, Debbie Boles, Ayla Burns, Nuthawin Charoensri, Oren Cohen, Susan Countryman, Mary Ann Cristobal, Bobbi Croy, Suzanne Dale, Hrushikesh Deshmukh, Amanda Douglas, Vincent Drouillon, Marcia Eisenberg, Howard Engler, Rama Ghatti, Prashant Gupta, Susan Hicks, Jake Humphrey, Lax Iyer, Manoj Jain, Mohan Kolli, Brian Krueger, Tim Kuphal, Stanley Letovsky, Michael Levandoski, Craig Lukasik, Jonathan Meltzer, Brian Norvell, Mindy Nye, Scott Parker, Christos Petropoulos, John Pruitt, Steven Ragan, Scott Ryan, Mike Sapeta, Jana Schroth, Suresh Babu Selvaraju, Goran Stevovic, Amanda Suchanek, Andrea Throop, Lyndon Tilson, Thomas Urban, Joe Voshell, Kimberly Wagner, Jonathan Williams, Mary Williamson, Qian Zeng, Tricia Zwiefelhofer, Clinton R. Paden, Duncan MacCannell
EPI_ISL_2249022	unknown	Instituto Nacional de Saude (INSA)	Borges et al
EPI_ISL_2253206, EPI_ISL_2253208, EPI_ISL_2253210	Respiratory Viruses Branch, Centers for Disease Control and Prevention	Respiratory Viruses Branch, Centers for Disease Control and Prevention	Howard,D., Batra,D., Cook,P.W., Moser,K., Paskey,A., Caravas,J., Rambo-Martin,B., Morrison,S., Gulvick,C., Sammons,S., Unoarumhi,Y., Wagner,D., Schmerer,M., Agarwal,M., Almasri,E., Boles,D., Burns,A., Charoensri,N., Cohen,O., Countryman,S., Cristobal,M.A., Croy,B., Dale,S., Deshmukh,H., Douglas,A., Drouillon,V., Eisenberg,M., Engler,H., Ghatti,R., Gupta,P., Hicks,S., Humphrey,J., Iyer,L., Jain,M., Kolli,M., Krueger,B., Kuphal,T., Letovsky,S., Levandoski,M., Lukasik,C., Meltzer,J., Norvell,B., Nye,M., Parker,S., Petropoulos,C., Pruitt,J., Ragan,S., Ryan,S., Sapeta,M., Schroth,J., Selvaraju,S.B., Stevovic,G., Suchanek,A., Throop,A., Tilson,L., Urban,T., Voshell,J., Wagner,K., Williams,J., Williamson,M., Zeng,Q., Zwiefelhofer,T., Paden,C.R., MacCannell,D.
EPI_ISL_2254233	Microbiology Department, Laboratori Clínic Metropolitana Nord. Hospital Universitari Germans Trias i Pujol.	Can Ruti SARS-CoV-2 Sequencing Hub (HUGTiP/IrsiCaixa/IGTP)	Marc Noguera-Julian, Pilar Armengol, Ignacio Blanco, Antoni E Bordoy, Francesc Catala-Moll, Pere-Joan Cardona, Maria Casadellà, Cristina Casañ, Gemma Clara, Bonaventura Clotet, Cristina Esteban, Montserrat Giménez, Mercedes Guerrero, Anna Not, Roger Paredes, Mariona Parera, Verónica Saludes, Alba Sánchez, and Elisa Martró on behalf of the Can Ruti SARS-CoV-2 Sequencing Hub.
EPI_ISL_2260982, EPI_ISL_2260986, EPI_ISL_2260989, EPI_ISL_2260992, EPI_ISL_2260996, EPI_ISL_2260998, EPI_ISL_2261021, EPI_ISL_2261024, EPI_ISL_2261036	Laborarztpraxis Osnabrück	Robert Koch Institute	unknown
EPI_ISL_2261046	MVZ Dr. Eberhard & Partner Dortmund	Robert Koch Institute	unknown
EPI_ISL_2263037	Labor Dr. Wisplinghoff - Köln	Robert Koch Institute	unknown
EPI_ISL_2263120, EPI_ISL_2264544	MDI Limbach Berlin GmbH; MVZ Labor Berlin	Robert Koch Institute	unknown

EPI_ISL_2264550	Labor Prof. Dr. G. Enders MVZ GbR	Robert Koch Institute	unknown
EPI_ISL_2264712	MVZ für Laboratoriumsmedizin und Mikrobiologie Koblenz-Mittelrhein (Labor Koblenz)	Robert Koch Institute	unknown
EPI_ISL_2264731, EPI_ISL_2264733	MDI Limbach Berlin GmbH; MVZ Labor Berlin	Robert Koch Institute	unknown
EPI_ISL_2265532	Labor Prof. Dr. G. Enders MVZ GbR	Robert Koch Institute	unknown
EPI_ISL_2266641	MVZ Labor Krone GbR	Robert Koch Institute	unknown
EPI_ISL_2275970	Istituto Zooprofilattico Sperimentale del Mezzogiorno - Azienda Ospedaliera "Pugliese Ciaccio" di Catanzaro	TIGEM	Antonio Grimaldi Patrizia Annunziata Francesco Panariello Biancamaria Pierri Claudia Tiberio Teresa Giuliano Valentina Bouche Chiara Colantuono Maria Concetta Cuomo Denise Di Concilio Lucio Di Filippo Anna Manfredi Pasquale Minchella Marcello Salvi Antonio Limone Luigi Atripaldi Pellegrino Cerino Andrea Ballabio Davide Cacchiarelli
EPI_ISL_2282048, EPI_ISL_2282136	Istituto Zooprofilattico Sperimentale del Mezzogiorno	TIGEM	Antonio Grimaldi Patrizia Annunziata Francesco Panariello Biancamaria Pierri Claudia Tiberio Teresa Giuliano Valentina Bouche Chiara Colantuono Maria Concetta Cuomo Denise Di Concilio Lucio Di Filippo Anna Manfredi Marcello Salvi Antonio Limone Luigi Atripaldi Pellegrino Cerino Andrea Ballabio Davide Cacchiarelli
EPI_ISL_2303932	Dutch COVID-19 response team	National Institute for Public Health and the Environment (RIVM)	Adam Meijer, Harry Vennema, Dirk Eggink, Jeroen Cremer, Sharon van den Brink, Bas van der Veer, AnneMarie van den Brandt, Lisa Wijsman, Kim Freriks, Ryanne Jaarsma, Euníce Then, Lynn Aarts, Sanne Bos, Melissa van Tuil, Linda van de Nes, Sjoerd Kuiling, James Groot, Florian Zwagemaker, Dennis Schmitz, Annelies Kroneman, Karim Hajji, Chantal Reusken, on behalf of the national COVID-19 response team
EPI_ISL_2306684, EPI_ISL_2307166	Laboratory Corporation of America	Centers for Disease Control and Prevention Division of Viral Diseases, Pathogen Discovery	Dakota Howard, Dhwani Batra, Peter W. Cook, Kara Moser, Adrian Paskey, Jason Caravas, Benjamin Rambo-Martin, Shatavia Morrison, Christopher Gulvick, Scott Sammons, Yvette Unoarumhi, Darlene Wagner, Matthew Schmerer, Minoo Agarwal, Eyad Almasri, Debbie Boles, Ayla Burns, Nuthawin Charoensri, Oren Cohen, Susan Countryman, Mary Ann Cristobal, Bobbi Croy, Suzanne Dale, Hrushikesh Deshmukh, Amanda Douglas, Vincent Drouillon, Marcia Eisenberg, Howard Engler, Rama Ghatti, Prashant Gupta, Susan Hicks, Jake Humphrey, Lax Iyer, Manoj Jain, Mohan Kolli, Brian Krueger, Tim Kuphal, Stanley Letovsky, Michael Levandoski, Craig Lukasik, Jonathan Meltzer, Brian Norvell, Mindy Nye, Scott Parker, Christos Petropoulos, John Pruitt, Steven Ragan, Scott Ryan, Mike Sapeta, Jana Schroth, Suresh Babu Selvaraju, Goran Stevovic, Amanda Suchanek, Andrea Throop, Lyndon Tilson, Thomas Urban, Joe Voshell, Kimberly Wagner, Jonathan Williams, Mary Williamson, Qian Zeng, Tricia Zwiefelhofer, Clinton R. Paden, Duncan MacCannell
EPI_ISL_2308562, EPI_ISL_2308563, EPI_ISL_2308571, EPI_ISL_2308572, EPI_ISL_2308573, EPI_ISL_2308593, EPI_ISL_2308595, EPI_ISL_2308596	SIESP DIPARTIMENTO DI PREVENZIONE TERAMO	Istituto Zooprofilattico Sperimentale dell'Abruzzo e Molise "G. Caporale"	Lorusso A, Marcacci M, Di Domenico M, Ancora M, Curini V, Di Lollo Valeria, Mangone I, Rinaldi A, Delli Compagni E, Scialabba S, Caporale M, Di Pasquale A, Cammà C, Puglia I, Calistri P, Savini G
EPI_ISL_2308706	Università degli Studi di Perugia	Istituto Zooprofilattico Sperimentale dell'Abruzzo e Molise "G. Caporale"	Mencacci A, Camilloni B, Biagetti M, GiammarioliM, Lorusso A, Marcacci M, Di Domenico M, Ancora M, Curini V, Mangone I, Rinaldi A, Delli Compagni E, Scialabba S, Di Pasquale A, Cammà C, Puglia I, Calistri P, Savini G
EPI_ISL_2313255, EPI_ISL_2313261, EPI_ISL_2313269, EPI_ISL_2313274, EPI_ISL_2313280, EPI_ISL_2313295	Laborarztpraxis Osnabrück	Robert Koch Institute	unknown
EPI_ISL_2313305	MVZ Dr. Eberhard & Partner Dortmund	Robert Koch Institute	unknown
EPI_ISL_2313307, EPI_ISL_2313323, EPI_ISL_2313337	Laborarztpraxis Osnabrück	Robert Koch Institute	unknown
EPI_ISL_2314411, EPI_ISL_2314664	MDI Limbach Berlin GmbH; MVZ Labor Berlin	Robert Koch Institute	unknown
EPI_ISL_2314778	MVZ für Laboratoriumsmedizin und Mikrobiologie Koblenz-Mittelrhein (Labor Koblenz)	Robert Koch Institute	unknown
EPI_ISL_2314790	MDI Limbach Berlin GmbH; MVZ Labor Berlin	Robert Koch Institute	unknown
EPI_ISL_2323015	cerballiance-IDF	Cerba lab	Emmanuel Lecorche, Sabine Trombert-Paolantoni, Aude Lessenne, Kader Merah, Thierry Collin, Laura Verdurme, Patrice Herisson, Stéphanie Haïm-Boukobza, Bénédicte Roquebert
EPI_ISL_2323467	CH ANGOULEME	CHU Poitiers	Caroline MICHAUD, Maxime PICHON, Manon PRAT, Valentin BON-BARET, Birama N'DIAYE, Magali GARCIA, Agnes BEBY-DEFAUX, Nicolas LEVEQUE
EPI_ISL_2331636	Johns Hopkins Hospital Department of Pathology	Johns Hopkins Hospital Department of Pathology	C. Paul Morris, Chun Huai Luo, Adannaya Amadi, Matthew Schwartz, Nicholas Gallagher, Heba H. Mostafa
EPI_ISL_2332583	Microbiology Department, University Hospital Donostia	Microbiology Department, University Hospital Donostia	Marimon JM, Montes M, Piñeiro L, Grandioso D, Martin-Peñaranda T, Gomez M, Cilla G.
EPI_ISL_2332616	Azienda Sanitaria Locale di Piacenza - Presidio Ospedaliero - Laboratorio di Microbiologia	Istituto Zooprofilattico Sperimentale della Lombardia e dell'Emilia Romagna (IZSLER), Risk Analysis and Genomic Epidemiology Unit	Lo Cascio Giuliana, Schiavo Roberta, Peroni Irene, Marina Morganti, Ilaria Menozzi, Erika Scaltriti, Stefano Pongolini
EPI_ISL_2332986	UW Virology Lab	UW Virology Lab	Pavitra Roychoudhury, Hong Xie, Lasata Shrestha, Tien V. Nguyen, Shah Mohamed Bakhash, Michelle Lin, Noah R. Baker, Ricardo Perez, Sean Ellis, Nathan Breit, Robert J. Livingston, Meei-Li Huang, Keith R Jerome, Patrick Mathias, Alexander Greninger
EPI_ISL_2333501	Norwegian Institute of Public Health, Department of Virology	Norwegian Institute of Public Health, Department of Virology	'Kathrine Stene-Johansen, Kamilla Heddeland Instefjord, Hilde Elshaug, Garcia Llorente Ignacio, Jon Bråte, Engebretsen Serina Beate,Pedersen Benedikte Nevjen, Debech Nadia, Line Victoria Moen, Atiya R Ali,Marie Paulsen Madsen, Rasmus Riis Kopperud, Hilde Vollan, Karoline Bragstad, Olav Hungnes
EPI_ISL_2340278	Instituto Nacional de Saude (INSA)	Instituto Nacional de Saude (INSA)	Borges et al
EPI_ISL_2340797	UW Virology Lab	UW Virology Lab	Pavitra Roychoudhury, Hong Xie, Lasata Shrestha, Tien V. Nguyen, Shah Mohamed Bakhash, Michelle Lin, Noah R. Baker, Sean Ellis, Meei-Li Huang, Keith R Jerome, Alexander Greninger
EPI_ISL_2346425, EPI_ISL_2346427, EPI_ISL_2346428, EPI_ISL_2346429, EPI_ISL_2346433, EPI_ISL_2346435	MRC/UVRI & LSHTM Uganda Research Unit, Central Public Health Laboratories	MRC/UVRI & LSHTM Uganda Research Unit, Central Public Health Laboratories	Matthew Cotten, Dan Lule Bugembe, My V.T. Phan, Pontiano Kaleebu,Isaac Sseeewanyana, Patrick Semanda, Susan Nabadda
EPI_ISL_2348189	Lighthouse Lab in Alderley Park	Wellcome Sanger Institute for the COVID-19 Genomics UK (COG-UK) Consortium	Jacquelyn Wynn, Mairead Hyland, The Lighthouse Lab in Alderley Park and Alex Alderton, Roberto Amato, Jeffrey Barrett, Sonia Goncalves, Ewan Harrison, David K. Jackson, Ian Johnston, Dominic Kwiatkowski, Cordelia Langford, John Sillitoe on behalf of the Wellcome Sanger Institute COVID-19 Surveillance Team
EPI_ISL_2348662	Genome Center	Genome Center	Ovinu Kibria Islam, M. Tanvir Islam, A. S. M. Rubayet Ul Alam, Md. Shazid Hasan, Shovon Lal Sarkar, Ali Ahsan Setu, Tanay Chakrabarty,Prosanto Kumar Das, M. Shaminur Rahman, Hassan M. Al-Emran, Iqbal Kabir Jahid, M. Anwar Hossain
EPI_ISL_2361529	CHU LILLE	CHU Lille - Laboratoire de Virologie	AIT YAHYA Emilie, ALIDJINOU Enagnon Kazali, BOCKET Laurence, CREPIN Michel, DEMAY Christophe, ENGELMANN Ilka, GEFFROY Sandrine, GUIGON Aurélie, LAMBERT Valérie, LAZREK Mouna, NOBILLIAUX Florian, PREVOST Brigitte, THUILLIER Caroline, TINEZ Claire ,TCHANTCHOU NJOSSE YANICK
EPI_ISL_2363193	National Virus Reference Laboratory	National Virus Reference Laboratory	Zoe Yandle, Charlene Bennett, Gabriel Gonzalez, Michael Carr, Jonathan Dean, Cillian F De Gascun
EPI_ISL_2363374	National Virus Reference Laboratory	National Virus Reference Laboratory	Fiona Crispie, Calum Walsh, Matthew McCabe, Zoe Yandle, Charlene Bennet, Gabriel Gonzalez, Michael Carr, Jonathan Dean, Paul Cotter, Cillian F De Gascun
EPI_ISL_2373194	AP SSO	CSIR-Centre for Cellular and Molecular Biology-INSACOG	Blessy B John,Viswagithe S L,Tulasi Nagabandi, Lamuk Zaveri,Ara Sreenivas,Shreekant Verma, Amareshwar Vodapalli ,B Himasri,Valli Nagalakshmi Undamatla,Payel Mukherjee,Sofia Banu,Archana Bharadwaj Siva,Sharath Chandra Thota,Karthik Bharadwaj Tallapaka,Rakesh K Mishra,Divya Tej

			Sowpati
EPI_ISL_2373258	CSIR-Centre for Cellular and Molecular Biology	CSIR-Centre for Cellular and Molecular Biology-INSACOG	Lamuk Zaveri,Tulasi Nagabandi, Ara Sreenivas,Shreekant Verma, Amareshwar Vodapalli ,Blessy B John,Viswagithe S L,B Himasri,Valli Nagalakshmi Undamatla,Payel Mukherjee,Sofia Banu,Archana Bharadwaj Siva,Sharath Chandra Thota,Karthik Bharadwaj Tallapaka,Rakesh K Mishra,Divya Tej Sowpati
EPI_ISL_2373312	Mapmygenome	CSIR-Centre for Cellular and Molecular Biology-INSACOG	Ara Sreenivas,Shreekant Verma,Tulasi Nagabandi, Lamuk Zaveri, Amareshwar Vodapalli ,Blessy B John,Viswagithe S L,B Himasri,Valli Nagalakshmi Undamatla,Payel Mukherjee,Sofia Banu,Archana Bharadwaj Siva,Sharath Chandra Thota,Karthik Bharadwaj Tallapaka,Rakesh K Mishra,Divya Tej Sowpati
EPI_ISL_2373375	Mapmygenome	CSIR-Centre for Cellular and Molecular Biology-INSACOG	B Himasri,Valli Nagalakshmi Undamatla,Lamuk Zaveri,Tulasi Nagabandi, Ara Sreenivas,Shreekant Verma, Amareshwar Vodapalli ,Blessy B John,Viswagithe S L,Payel Mukherjee,Sofia Banu,Archana Bharadwaj Siva,Sharath Chandra Thota,Karthik Bharadwaj Tallapaka,Rakesh K Mishra,Divya Tej Sowpati
EPI_ISL_2373848	BIOMNIS LYON	CNR Virus des Infections Respiratoires - France SUD	Antonin Bal, Gregory Destras, Gwendolyne Burfin, Hadrien Regue, Quentin Semanas, Martine Valette, Bruno Lina, Laurence Josset
EPI_ISL_2373986, EPI_ISL_2373987, EPI_ISL_2373988, EPI_ISL_2373989, EPI_ISL_2373991, EPI_ISL_2373992, EPI_ISL_2373993, EPI_ISL_2373995	Laboratoire Virologie Saint Louis APHP	Laboratoire Virologie Saint Louis APHP	Maud Salmona, Marie Laure Chaix, Severine Mercier Delarue, Marie Laure Néré, Linda Feghoul, Jérome Le Goff, Constance Delauguerre, Sophia Achaibou
EPI_ISL_2376398	ULSS 2 Marca Trevigiana	Istituto Zooprofilattico Sperimentale delle Venezie	Adelaide Milani, Alessia Schivo, Annalisa Salviato, Elisa Palumbo, Erika Giorgia Quaranta, Luca Tassoni, Ambra Pastori, Edoardo Giussani, Alice Fusaro, Isabella Monne, Calogero Terregino, Antonia Ricci
EPI_ISL_2379487	Dr S Raju, Director of Public Heath and Preventive Medicine	inStem NCBS - INSACOG	Uma Ramakrishnan Dasaradhi Palakodeti Aswin SaiNarain
EPI_ISL_2385971, EPI_ISL_2385974	PHV-FSS	PHV-FSS	Son Nguyen
EPI_ISL_2386845	Labor Becker & Kollegen (Standort München)	Robert Koch Institute	unknown
EPI_ISL_2387394	MVZ Dr. Eberhard & Partner Dortmund	Robert Koch Institute	unknown
EPI_ISL_2388219, EPI_ISL_2388220, EPI_ISL_2388222, EPI_ISL_2388246, EPI_ISL_2388250, EPI_ISL_2388315	Laborarztpraxis Osnabrück	Robert Koch Institute	unknown
EPI_ISL_2396153	Lighthouse Lab in Alderley Park	Wellcome Sanger Institute for the COVID-19 Genomics UK (COG-UK) Consortium	Jacquelyn Wynn, Mairead Hyland, The Lighthouse Lab in Alderley Park and Alex Alderton, Roberto Amato, Jeffrey Barrett, Sonia Goncalves, Ewan Harrison, David K. Jackson, Ian Johnston, Dominic Kwiatkowski, Cordelia Langford, John Sillitoe on behalf of the Wellcome Sanger Institute COVID-19 Surveillance Team
EPI_ISL_2398498	Laboratory Corporation of America	Centers for Disease Control and Prevention Division of Viral Diseases, Pathogen Discovery	Dakota Howard, Dhwani Batra, Peter W. Cook, Kara Moser, Adrian Paskey, Jason Caravas, Benjamin Rambo-Martin, Shatavia Morrison, Christopher Gulvick, Scott Sammons, Yvette Unoarumhi, Darlene Wagner, Matthew Schmerer, Minoo Agarwal, Eyad Almasri, Debbie Boles, Ayla Burns, Nuthawin Charoensri, Oren Cohen, Susan Countryman, Mary Ann Cristobal, Bobbi Croy, Suzanne Dale, Hrushikesh Deshmukh, Amanda Douglas, Vincent Drouillon, Marcia Eisenberg, Howard Engler, Rama Ghatti, Prashant Gupta, Susan Hicks, Jake Humphrey, Lax Iyer, Lisa Pfefferle, Manoj Jain, Matthew Robinson, Mohan Kolli, Brian Krueger, Tim Kuphal, Stanley Letovsky, Michael Levandoski, Craig Lukasik, Jonathan Meltzer, Brian Norvell, Mindy Nye, Scott Parker, Christos Petropoulos, John Pruitt, Steven Ragan, Scott Ryan, Mike Sapeta, Jana Schroth, Suresh Babu Selvaraju, Goran Stevovic, Amanda Suchanek, Andrea Throop, Lyndon Tilson, Thomas Urban, Joe Voshell, Kimberly Wagner, Jonathan Williams, Mary Williamson, Qian Zeng, Tricia Zwiefelhofer, Clinton R. Paden, Duncan MacCannell
EPI_ISL_2399786, EPI_ISL_2399836	National Virus Reference Laboratory	National Virus Reference Laboratory	Zoe Yandle, Charlene Bennett, Gabriel Gonzalez, Michael Carr, Jonathan Dean, Cillian F De Gascun
EPI_ISL_2400460	Microbiologia e Virologia	Istituto Zooprofilattico Sperimentale delle Venezie	Adelaide Milani, Alessia Schivo, Annalisa Salviato, Elisa Palumbo, Erika Giorgia Quaranta, Luca Tassoni, Ambra Pastori, Edoardo Giussani, Alice Fusaro, Isabella Monne, Calogero Terregino, Antonia Ricci
EPI_ISL_2400504	cerballiance-NOR	Cerba lab	Emmanuel Lecorche, Sabine Trombert-Paolantoni, Gwénolé Prigent, Antoine Prigent, Francois Xavier Le Guillou, Alexandre Leriche, Laura Verdurme, Sylvain Metge, Stéphanie Haïm-Boukobza, Bénédicte Roquebert
EPI_ISL_2400558, EPI_ISL_2400636	Laboratoires d'analyses medicales - Ketterthill	Laboratoire national de sante, Microbiology, Microbial Genomics Platform	Anke Wienecke-Baldacchino, Catherine Ragimbeau,Jessica Tapp, Fatu Djabi, Lise Pignon, Raoul Salmon, Serge Vedy, Caroline Scheiber, Tamir Abdelrahman
EPI_ISL_2401539, EPI_ISL_2401597	BioneXt Lab	Laboratoire national de sante, Microbiology, Microbial Genomics Platform	Anke Wienecke-Baldacchino, Catherine Ragimbeau,Jessica Tapp, Fatu Djabi, Lise Pignon, Raoul Salmon, Thibault Ferrandon, Tamir Abdelrahman
EPI_ISL_2403649	Ministry of Health Turkey	Ministry of Health Turkey	Fatma Bayrakdar, Yasemin Cosgun, Suleyman Yalcin, Gulay Korukluoglu
EPI_ISL_2403794	Dianalabs SA	Genesupport	Tanguy ARAUD
EPI_ISL_2406496, EPI_ISL_2406497	Laboratoire Virologie Saint Louis APHP	Laboratoire Virologie Saint Louis APHP	Maud Salmona, Marie Laure Chaix, Severine Mercier Delarue, Marie Laure Néré, Linda Feghoul, Jérome Le Goff, Constance Delauguerre, Sophia Achaibou
EPI_ISL_2420349	cerballiance-IDF	Cerba lab	Emmanuel Lecorche, Sabine Trombert-Paolantoni, Aude Lessenne, Kader Merah, Thierry Collin, Laura Verdurme, Patrice Herisson, Stéphanie Haïm-Boukobza, Bénédicte Roquebert
EPI_ISL_2425247, EPI_ISL_2425251, EPI_ISL_2425252, EPI_ISL_2425330, EPI_ISL_2425331	KU Leuven, Rega Institute, Clinical and Epidemiological Virology	KU Leuven, Rega Institute, Clinical and Epidemiological Virology	Tony Wawina-Bokalanga, Bert Vanmechelen, Joan Marti-Carerras, Piet Maes
EPI_ISL_2425566	cerballiance-PACA	Cerba lab	Emmanuel Lecorche, Sabine Trombert-Paolantoni, Laurence Prots, Jean-Christophe Roig, Edouard Delaunay, Isabelle Butin, Laura Verdurme, Bruno Roure, Stéphanie Haïm-Boukobza, Bénédicte Roquebert
EPI_ISL_2425571	cerballiance-IDF	Cerba lab	Emmanuel Lecorche, Sabine Trombert-Paolantoni, Aude Lessenne, Kader Merah, Thierry Collin, Laura Verdurme, Patrice Herisson, Stéphanie Haïm-Boukobza, Bénédicte Roquebert
EPI_ISL_2425598	cerballiance-PACA	Cerba lab	Emmanuel Lecorche, Sabine Trombert-Paolantoni, Laurence Prots, Jean-Christophe Roig, Edouard Delaunay, Isabelle Butin, Laura Verdurme, Bruno Roure, Stéphanie Haïm-Boukobza, Bénédicte Roquebert
EPI_ISL_2425607	cerballiance-IDF	Cerba lab	Emmanuel Lecorche, Sabine Trombert-Paolantoni, Aude Lessenne, Kader Merah, Thierry Collin, Laura Verdurme, Patrice Herisson, Stéphanie Haïm-Boukobza, Bénédicte Roquebert
EPI_ISL_2429686	Department of Bacteria, Parasites and Fungi, Statens Serum Institut, Copenhagen, Denmark	Statens Serum Institut Bioinformatics and Microbial Genomics	Danish Covid-19 Genome Consortium
EPI_ISL_2445698, EPI_ISL_2445790, EPI_ISL_2445844, EPI_ISL_2445864, EPI_ISL_2445865	unknown	Instituto Nacional de Saude (INSA)	Borges et al
EPI_ISL_2446047, EPI_ISL_2446082, EPI_ISL_2446084	Pandemic Response Lab - NYC	Pandemic Response Lab, R&D	Henry Lee, Michael Hammerling, Melissa Hopkins, Cybill del Castillo, Shinyoung Clair Kang, William Ward, Pradeep Bugga, Sol Rey, Dylan Law, Katharine Nelson, Haiping Hao, Jon Laurent
EPI_ISL_2448424	ULSS 3 Venezia	Istituto Zooprofilattico Sperimentale delle Venezie	Adelaide Milani, Alessia Schivo, Annalisa Salviato, Elisa Palumbo, Erika Giorgia Quaranta, Luca Tassoni, Ambra Pastori, Edoardo Giussani, Alice Fusaro, Isabella Monne, Calogero Terregino, Antonia Ricci
EPI_ISL_2448436, EPI_ISL_2448439, EPI_ISL_2448440, EPI_ISL_2448444	ULSS 2 Marca Trevigiana	Istituto Zooprofilattico Sperimentale delle Venezie	Adelaide Milani, Alessia Schivo, Annalisa Salviato, Elisa Palumbo, Erika Giorgia Quaranta, Luca Tassoni, Ambra Pastori, Edoardo Giussani, Alice Fusaro, Isabella Monne, Calogero Terregino, Antonia Ricci

EPI_ISL_2448447, EPI_ISL_2448448, EPI_ISL_2448451, EPI_ISL_2448452, EPI_ISL_2448455, EPI_ISL_2448461, EPI_ISL_2448462, EPI_ISL_2448463, EPI_ISL_2448464	ULSS 6 Euganea	Istituto Zooprofilattico Sperimentale delle Venezie	Adelaide Milani, Alessia Schivo, Annalisa Salviato, Elisa Palumbo, Erika Giorgia Quaranta, Luca Tassoni, Ambra Pastori, Edoardo Giussani, Alice Fusaro, Isabella Monne, Calogero Terregino, Antonia Ricci
EPI_ISL_2448467, EPI_ISL_2448468	ULSS 5 Polesana	Istituto Zooprofilattico Sperimentale delle Venezie	Adelaide Milani, Alessia Schivo, Annalisa Salviato, Elisa Palumbo, Erika Giorgia Quaranta, Luca Tassoni, Ambra Pastori, Edoardo Giussani, Alice Fusaro, Isabella Monne, Calogero Terregino, Antonia Ricci
EPI_ISL_2449937	UniversitŠtsSpital ZŸrich	Institute of Medical Virology	Daniel Ehrsam, Isabel StŸrmer, Catharine Aquino, Joel Wirz, Weihong Qi, Hubert Rehrauer, Verena Kufner, Gabriela Ziltener, Maryam Zaheri, Stefan Schmutz, Annette AudigŽ, Maria GrŸnberg, Kevin Steiner, Jon Huder, Cyril Shah, Riccarda Capaul, Guido Bloemberg, JŸrg Bšni, Michael Huber, Alexandra Trkola
EPI_ISL_2450791	National Public Health Laboratory, Ministry of Health, Ministry of Health, Republic of South Sudan	South Sudan Ministry of Health, WHO South Sudan, MRC/UVRI & LSHTM Uganda Research Unit	Joseph Francis Wamala, Abe G. Abias, Dennis Kenyi Lodiongo, Sudhir Bunga, James Ayei, Lul Lojok Deng, Richard Lino Loro Lako, John Rumunu, Juma John HM, Dan Lule Bugembe, My V.T. Phan, Pontiano Kaleebu, Matthew Cotten
EPI_ISL_2455642, EPI_ISL_2455710, EPI_ISL_2455728	Università Federico II - Dipartimento di scienze mediche traslazionali - Napoli	TIGEM	Antonio Grimaldi Patrizia Annunziata Francesco Panariello Teresa Giuliano Michele Cennamo Valentina Bouche Chiara Colantuono Lucio Di Filippo Mariano Fiorenza Anna Manfredi Marcello Salvi Giuseppe Portella Andrea Ballabio Davide Cacchiarelli
EPI_ISL_2462425	South Eastern Area Laboratory Services (SEALS)	NSW Health Pathology - Institute of Clinical Pathology and Medical Research; Westmead Hospital; University of Sydney	CIDM-PH et al.
EPI_ISL_2467865	Usansolo-Galdakao University Hospital	Cruces University Hospital	Ana Gual-de-Torrella, Izaskun Alejo-Cancho, Mikel Gallego, Ana Belén de la Hoz
EPI_ISL_2469753	Med. Labor Prof. Schenk Dr. Ansorge & Kollegen	Robert Koch Institute	unknown
EPI_ISL_2471612	Eurofins LifeCodexx GmbH	Robert Koch Institute	unknown
EPI_ISL_2471762	Medizinisch-Diagnostisches Labor Kempten allgäulab	Robert Koch Institute	unknown
EPI_ISL_2471936	MDI Limbach Berlin GmbH; MVZ Labor Berlin	Robert Koch Institute	unknown
EPI_ISL_2476255	Dutch COVID-19 response team	National Institute for Public Health and the Environment (RIVM)	Adam Meijer, Harry Vennema, Dirk Eggink, Jeroen Cremer, Sharon van den Brink, Bas van der Veer, AnneMarie van den Brandt, Lisa Wijsman, Kim Freriks, Ryanne Jaarsma, Euníce Then, Lynn Aarts, Sanne Bos, Melissa van Tuil, Linda van de Nes, Florian Zwagemaker, Dennis Schmitz, Annelies Kroneman, Karim Hajji, Chantal Reusken, on behalf of the national COVID-19 response team
EPI_ISL_2477718, EPI_ISL_2477730, EPI_ISL_2478300, EPI_ISL_2478308, EPI_ISL_2478332, EPI_ISL_2478338, EPI_ISL_2479079, EPI_ISL_2479082, EPI_ISL_2479089, EPI_ISL_2479140, EPI_ISL_2479141, EPI_ISL_2479147, EPI_ISL_2479165, EPI_ISL_2479227, EPI_ISL_2479229			
see above	Edmonton Provincial Lab	Public Health Agency of Canada (PHAC) National Microbiology Laboratory	Buss, E, Croxen M, Deo A, Dieu P, Gill K, Ferrato C, Khan F, Koleva P, Li V, Lloyd C, Lynch T, Ma R, Murphy S, Pabbaraju K, Shokoples S, Tipples G, Thayer J, Whitehouse M, Wong A, Yu C, Zelyas N
EPI_ISL_2483575, EPI_ISL_2483576	SIESP DIPARTIMENTO DI PREVENZIONE TERAMO	Istituto Zooprofilattico Sperimentale dell'Abruzzo e Molise "G. Caporale"	Lorusso A, Marcacci M, Di Domenico M, Ancora M, Curini V, Di Lollo Valeria, Mangone I, Rinaldi A, Delli Compagni E, Scialabba S, Caporale M, Di Pasquale A, Cammà C, Puglia I, Calistri P, Savini G
EPI_ISL_2489270	Aegis Sciences Corporation	Centers for Disease Control and Prevention Division of Viral Diseases, Pathogen Discovery	Dakota Howard, Dhwani Batra, Peter W. Cook, Kara Moser, Adrian Paskey, Jason Caravas, Benjamin Rambo-Martin, Shatavia Morrison, Christopher Gulvick, Scott Sammons, Yvette Unoarumhi, Darlene Wagner, Matthew Schmerer, Cyndi Clark, Patrick Campbell, Rob Case, Vikramsinha Ghorpade, Holly Houdeshell, Ola Kvalvaag, Dillon Nall, Ethan Sanders, Alec Vest, Shaun Westlund, Matthew Hardison, Clinton R. Paden, Duncan MacCannell
EPI_ISL_2490026, EPI_ISL_2490036	Seattle Flu Study	Seattle Flu Study	Deborah A. Nickerson, Chris D. Frazar, Jover Lee, Benjamin Pelle, Erica Ryke, Matthew Richardson, Amanda Adler, Elisabeth Brandstetter, Peter D. Han, Kairsten Fay, Misja Ilcisin, Kirsten Lacombe, Thomas R. Sibley, Melissa Truong, Caitlin R. Wolf, Karen Cowgill, Stephanie Schrag, Jeff Duchin, Michael Boeckh, Janet A. Englund, Michael Famulare, Barry R. Lutz, Mark J. Rieder, Lea M. Starita, Matthew Thompson, Helen Y. Chu, Trevor Bedford, Jay Shendure
EPI_ISL_2495344	HOSPITAL GENERAL UNIVERSITARIO DE GUADALAJARA	Instituto de Salud Carlos III	Iglesias-Caballero, M. Sandonís,V. Vázquez-Morón, S. Camarero, S. Pozo, F. Casas, I. Jiménez, P. Zaballos, A. Monzón, S. Varona, S. Cuesta, I.GONZALEZ PRAETORIUS, ALEJANDRO
EPI_ISL_2500436	CT Department of Public Health	CT Department of Public Health	Claire_Pearson,Tu_N_Nguyen
EPI_ISL_2533640, EPI_ISL_2533669	National Virus Reference Laboratory	National Virus Reference Laboratory	Zoe Yandle, Charlene Bennett, Gabriel Gonzalez, Michael Carr, Jonathan Dean, Cillian F De Gascun
EPI_ISL_2536216	Laboratorio HUB -Azienda Ospedaliero Universitaria - AOU - Cagliari	Laboratorio SPOKE Biologia Molecolare -Azienda Ospedaliero Universitaria - AOU - Cagliari	Germano Orrù, Sara Fais, Valentina Medda, Alessandra Scano, Miriam Loddo, Riccardo Cappai, Ferdinando Coghe
EPI_ISL_2536480	Incyte Diagnostics Spokane	Seattle Flu Study	Deborah A. Nickerson, Chris D. Frazar, Jover Lee, Benjamin Pelle, Erica Ryke, Matthew Richardson, Amanda Adler, Elisabeth Brandstetter, Peter D. Han, Kairsten Fay, Misja Ilcisin, Kirsten Lacombe, Thomas R. Sibley, Melissa Truong, Caitlin R. Wolf, Romesh Gautom, Geoff Melly, Brian Hiatt, Philip Dykema, Scott Lindquist, Michael Boeckh, Janet A. Englund, Michael Famulare, Barry R. Lutz, Mark J. Rieder, Lea M. Starita, Matthew Thompson, Helen Y. Chu, Jay Shendure, Trevor Bedford
EPI_ISL_2546745	ICMR-National Institute of Virology - INSACOG	NIV Influenza	Dr. Varsha Potdar and NIC Team
EPI_ISL_2548609	INSACOG-WB	National Institute of Biomedical Genomics - INSACOG	Arindam Maitra, Bhaswati Bandyopadhyay, Nidhan Kumar Biswas, Tamal Ghosh, Sreedhar Chinnaswamy, Ajay Chakraborti, Saumitra Das
EPI_ISL_2549566, EPI_ISL_2549567, EPI_ISL_2549568, EPI_ISL_2549569, EPI_ISL_2549570, EPI_ISL_2549571, EPI_ISL_2549572, EPI_ISL_2549573, EPI_ISL_2549574, EPI_ISL_2549575	Virology, Istituto Zooprofilattico Sperimentale dell'Abruzzo e del Molise 'G. Caporale'	Virology, Istituto Zooprofilattico Sperimentale dell'Abruzzo e del Molise 'G. Caporale'	Delli Compagni,E., Mangone,I., Scialabba,S., Caporale,M., Lorusso,A.
EPI_ISL_2557459	MOH - Jaber Al-Ahmad Hospital (Innovation Research Laboratory)	MOH - Jaber Al-Ahmad Hospital (Innovation Research Laboratory)	Salman Al-Sabah , Mohammad Alghounaim
EPI_ISL_2558084	LBM ALPHABIO, Marseille	LBM ALPHABIO, Marseille	Vincent GARCIA
EPI_ISL_2561989	cerballiance-IDF	Cerba lab	Emmanuel Lecorche, Sabine Trombert-Paolantoni, Laura Verdurme, Stéphanie Haïm-Boukobza, Bénédicte Roquebert
EPI_ISL_2562007	cerballiance-cote d'azur	Cerba lab	Emmanuel Lecorche, Sabine Trombert-Paolantoni, Laura Verdurme, Stéphanie Haïm-Boukobza, Bénédicte Roquebert
EPI_ISL_2597134, EPI_ISL_2597152, EPI_ISL_2597160, EPI_ISL_2597189	MEPHI, Aix Marseille University	MEPHI, Aix Marseille University	Anthony LEVASSEUR
EPI_ISL_2604147	Azienda Ospedaliera Terni	Istituto Zooprofilattico Sperimentale dell'Abruzzo e Molise "G. Caporale"	Palumbo M, Scaccetti A, Lorusso A, Marcacci M, Di Domenico M, Ancora M, Curini V, Mangone I, Rinaldi A, Delli Compagni E, Scialabba S, Di Pasquale A, Cammà C, Puglia I, Calistri P, Savini G
EPI_ISL_2615960	Berkeley Medical Center	WVU and Marshall University Combined Genomics Core Facilities	James Denvir, Peter Stoilov, Peter Perrotta, Wesley Kimble, Ryan Percifield
EPI_ISL_2615961	QLabs	WVU and Marshall University Combined Genomics Core Facilities	James Denvir, Peter Stoilov, Peter Perrotta, Wesley Kimble, Ryan Percifield
EPI_ISL_2615962	WV Labs	WVU and Marshall University Combined Genomics Core Facilities	James Denvir, Peter Stoilov, Peter Perrotta, Wesley Kimble, Ryan Percifield
EPI_ISL_2615963, EPI_ISL_2615964, EPI_ISL_2615965	Berkeley Medical Center	WVU and Marshall University Combined Genomics Core Facilities	James Denvir, Peter Stoilov, Peter Perrotta, Wesley Kimble, Ryan Percifield

EPI_ISL_2615966	WV Labs	WVU and Marshall University Combined Genomics Core Facilities	James Denvir, Peter Stoilov, Peter Perrotta, Wesley Kimble, Ryan Percifield
EPI_ISL_2615967	J.W. Ruby Memorial Hospital	WVU and Marshall University Combined Genomics Core Facilities	James Denvir, Peter Stoilov, Peter Perrotta, Wesley Kimble, Ryan Percifield
EPI_ISL_2615968, EPI_ISL_2615969, EPI_ISL_2615972	Berkeley Medical Center	WVU and Marshall University Combined Genomics Core Facilities	James Denvir, Peter Stoilov, Peter Perrotta, Wesley Kimble, Ryan Percifield
EPI_ISL_2617525	Lab Genomik Solidaritas Indonesia	National Institute of Health Research and Development	Vivi Setiawaty, Hana Apsari Pawestri, Hartanti Dian Ikawati, Subangkit, Kartika Dewi Puspa, Arie Ardiansyah Nugraha, Triyani Soekarso, Krisna Pangesti,Meutia Kumaheri, Nelly Puspandari
EPI_ISL_2620734	Functional Genomics and Proteomics Laboratory (FGPL), Department of Genetic Engineering and Biotechnology, University of Chittagong	Virology Laboratory, International Centre for Diarrhoeal Disease Research, Bangladesh (ICDDR,B)	Dr. Mohammad Al-Forkan, Dr. Sujat Paul, Dr. Laila Khaleda, Mojnu Miah, Rashedul Hasan, Md. Jibran Alam, Amit Datta, Rahee Hasan Chowdhury, Shuva Das, Inzamamul Ismail Shawon, Md. Abdur Rahman, Miftah Mushfique, Amlan Bhattacharjee, Dr. Mustafizur Rahman, Dr. Mohammad Enayet Hossain
EPI_ISL_2620903, EPI_ISL_2620905	Fondazione IRCCS Ca' Granda Ospedale Maggiore Policlinico	Fondazione IRCCS Ca' Granda Ospedale Maggiore Policlinico	Ferruccio Ceriotti, Sara Uceda Renteria
EPI_ISL_2622164, EPI_ISL_2622165, EPI_ISL_2622166, EPI_ISL_2622167, EPI_ISL_2622169, EPI_ISL_2622171, EPI_ISL_2622172, EPI_ISL_2622174, EPI_ISL_2622175	Azienda Ospedaliera Pugliese Ciaccio di Catanzaro SOC Microbiologia e Virologia	Azienda Ospedaliera Pugliese Ciaccio di Catanzaro SOC Microbiologia e Virologia	Rossana Tallerico Federica Pasceri Marco De Fazio Ilenia Talotta Giuseppina Panduri Pasquale Minchella
EPI_ISL_2628710	UNILABS	Instituto Nacional de Saude (INSA)	Borges et al
EPI_ISL_2631766, EPI_ISL_2631775, EPI_ISL_2631779	MVZ für Laboratoriumsmedizin und Mikrobiologie Koblenz-Mittelrhein (Labor Koblenz)	Robert Koch Institute	unknown
EPI_ISL_2631829, EPI_ISL_2631835	Labor Dr. Wisplinghoff - Köln	Robert Koch Institute	unknown
EPI_ISL_2632026	Medizinisches Labor Wahl Lüdenscheid	Robert Koch Institute	unknown
EPI_ISL_2632037, EPI_ISL_2632085	Laborarztpraxis Osnabrück	Robert Koch Institute	unknown
EPI_ISL_2632102, EPI_ISL_2633436	Medizinisches Labor Wahl Lüdenscheid	Robert Koch Institute	unknown
EPI_ISL_2633440, EPI_ISL_2633443	Laborarztpraxis Osnabrück	Robert Koch Institute	unknown
EPI_ISL_2634508	Eurofins LifeCodexx GmbH	Robert Koch Institute	unknown
EPI_ISL_2635787	Med. Labor Prof. Schenk Dr. Ansorge & Kollegen	Robert Koch Institute	unknown
EPI_ISL_2635841	MVZ für Laboratoriumsmedizin und Mikrobiologie Koblenz-Mittelrhein (Labor Koblenz)	Robert Koch Institute	unknown
EPI_ISL_2636094	Medizinisch-Diagnostisches Labor Kempten allgäulab	Robert Koch Institute	unknown
EPI_ISL_2636153	Corona-Testzentrum ifp Institut für Produktqualität GmbH	Robert Koch Institute	unknown
EPI_ISL_2636491	MVZ für Laboratoriumsmedizin und Mikrobiologie Koblenz-Mittelrhein (Labor Koblenz)	Robert Koch Institute	unknown
EPI_ISL_2636898	MVZ Dr. Eberhard & Partner Dortmund	Robert Koch Institute	unknown
EPI_ISL_2636900	Laborarztpraxis Osnabrück	Robert Koch Institute	unknown
EPI_ISL_2646019, EPI_ISL_2646083	UNILABS	Instituto Nacional de Saude (INSA)	Borges et al
EPI_ISL_2649124, EPI_ISL_2649497	SARS-CoV-2 Sequencing Castilla y Leon-Spain Consortium	SARS-CoV-2 Sequencing Castilla y Leon-Spain Consortium	Antonio Orduña-Domingo, Marta Hernandez, David Abad, Marta Dominguez-Gil, Silvia Rojo, Laura Sánchez de Prada, Sonsoles Garcinuño Pérez, Carmen Aldea-Mansilla, Mª Fe Brezmes-Valdivieso, Gregoria Megías Lobón, María Antonia García Castro, Carmen Gimeno Crespo, Noelia Arenal Andrés, Carlos Fuster Foz, M. Isabel Fernandez-Natal, Jose María Eiros Bouza
EPI_ISL_2650188, EPI_ISL_2650252, EPI_ISL_2650289	Microbiology Department, Laboratori Clínic Metropolitana Nord. Hospital Universitari Germans Trias i Pujol	Can Ruti SARS-CoV-2 Sequencing Hub (HUGTiP/IrsiCaixa/IGTP)	Marc Noguera-Julian, Pilar Armengol, Ignacio Blanco, Antoni E Bordoy, Francesc Catala-Moll, Pere-Joan Cardona, Maria Casadellà, Cristina Casañ, Gemma Clara, Bonaventura Clotet, Cristina Esteban, Montserrat Giménez, Mercedes Guerrero, Anna Not, Roger Paredes, Mariona Parera, Verónica Saludes, Alba Sánchez, and Elisa Martró on behalf of the Can Ruti SARS-CoV-2 Sequencing Hub
EPI_ISL_2652057	Hospital Universitari Joan XXIII	Hospital Universitari Joan XXIII	Gemma Recio, Cristina Gutiérrez, Clara Benavent, Carla Martín, Margarida Terrón, Ester Picó, Natalia Bastón
EPI_ISL_2654226, EPI_ISL_2654944, EPI_ISL_2654990, EPI_ISL_2655020, EPI_ISL_2655731, EPI_ISL_2655915, EPI_ISL_2656238, EPI_ISL_2656241, EPI_ISL_2656310	BCCDC Public Health Laboratory	BCCDC Public Health Laboratory	Prystajecky Natalie, Linda Hoang, Dan Fornika, John Tyson, Shannon Russell, Kim Macdonald, Kimia Kamelian, Ana Pacagnella, Corrinne Ng, Loretta Janz, Robert Azana, Mel Krajden
EPI_ISL_2661118	Mapmygenome	CSIR-Centre for Cellular and Molecular Biology-INSACOG	Tulasi Nagabandi,Lamuk Zaveri,Payel Mukherjee,Onkar Kulkarni, Sofia Banu, Ara Sreenivas,Shreekant Verma, Amareshwar Vodapalli ,B Himasri,Valli Nagalakshmi Undamatla, Priya Nurkurthy,Sumedha Avadhanula, Vidhyadhari Methuku, Archana Bharadwaj Siva, Karthik Bharadwaj Tallapaka,Rakesh K Mishra,Divya Tej Sowpati
EPI_ISL_2661132, EPI_ISL_2661141	Mapmygenome	CSIR-Centre for Cellular and Molecular Biology-INSACOG	Ara Sreenivas,Shreekant Verma, Amareshwar Vodapalli ,B Himasri,Valli Nagalakshmi Undamatla,Payel Mukherjee, Lamuk Zaveri, Onkar Kulkarni,Tulasi Nagabandi, Sofia Banu, ,Priya Nurkurthy, Vidhyadhari Methuku, Sumedha Avadhanula, Archana Bharadwaj Siva, Karthik Bharadwaj Tallapaka,Rakesh K Mishra,Divya Tej Sowpati
EPI_ISL_2672210, EPI_ISL_2672216	INSP, Bamako	Malaria Research and Training Center-Parasito	Antoine Dara, Ibrehima Guindo, Amadou Daou, Demba Koita, Abdoulaye Djimde
EPI_ISL_2678695	MVZ Labor Dr. Limbach & Kollegen GbR	Robert Koch Institute	unknown
EPI_ISL_2681581	Microbiology Department, Laboratori Clínic Metropolitana Nord. Hospital Universitari Germans Trias i Pujol	Can Ruti SARS-CoV-2 Sequencing Hub (HUGTiP/IrsiCaixa/IGTP)	Marc Noguera-Julian, Pilar Armengol, Ignacio Blanco, Antoni E Bordoy, Francesc Catala-Moll, Pere-Joan Cardona, Maria Casadellà, Cristina Casañ, Gemma Clara, Bonaventura Clotet, Cristina Esteban, Montserrat Giménez, Mercedes Guerrero, Anna Not, Roger Paredes, Mariona Parera, Verónica Saludes, Alba Sánchez, and Elisa Martró on behalf of the Can Ruti SARS-CoV-2 Sequencing Hub
EPI_ISL_2690456, EPI_ISL_2690457, EPI_ISL_2690459	MRC/UVRI & LSHTM Uganda Research Unit, Central Public Health Laboratories, Rakai Health Sciences Program	MRC/UVRI & LSHTM Uganda Research Unit, Central Public Health Laboratories	Matthew Cotten, Dan Lule Bugembe, My V.T. Phan, Pontiano Kaleebu,Isaac Sseeewanyana, Patrick Semanda, Susan Nabadda, Hellen Nansumba
EPI_ISL_2690465, EPI_ISL_2690466, EPI_ISL_2690467, EPI_ISL_2690468, EPI_ISL_2690469, EPI_ISL_2690470, EPI_ISL_2690471, EPI_ISL_2690472, EPI_ISL_2690473, EPI_ISL_2690474, EPI_ISL_2690475, EPI_ISL_2690476, EPI_ISL_2690477, EPI_ISL_2690478			
see above	MRC/UVRI & LSHTM Uganda Research Unit, Central Public Health Laboratories, Rakai Health Sciences Program	MRC/UVRI & LSHTM Uganda Research Unit, Rakai Health Sciences Program	Matthew Cotten, Dan Lule Bugembe, My V.T. Phan, Pontiano Kaleebu, Charles Ssuuna, Ronald Moses Galiwango, Steven J Reynolds
EPI_ISL_2691703	Analytica	Institute of Medical Virology	Verena Kufner, Gabriela Ziltener, Maryam Zaheri, Stefan Schmutz, Annette Audigé, Maria Grünberg, Kevin Steiner, Jon Huder, Cyril Shah, Riccarda Capaul, Guido Bloemberg, Jürg Böni, Michael Huber, Alexandra Trkola
EPI_ISL_2694831	Labor Berlin Charite Vivantes GmbH / Institut fur Virologie	Charite Universitatsmedizin Berlin, Institut fur Virologie/Labor Berlin	Peter Menzel, Christine Stephan, Rolf Schwarzer, Victor M Corman, Barbara Muhlemann, Terry Jones, Christian Drosten
EPI_ISL_2695344	CHEDV	Instituto Nacional de Saude (INSA)	Borges et al

EPI_ISL_2718650, EPI_ISL_2718858, EPI_ISL_2719916, EPI_ISL_2720267, EPI_ISL_2720309, EPI_ISL_2720322	BCCDC Public Health Laboratory	BCCDC Public Health Laboratory	Prystajecky Natalie, Linda Hoang, Dan Fornika, John Tyson, Shannon Russell, Kim Macdonald, Kimia Kamelian, Ana Pacagnella, Corrinne Ng, Loretta Janz, Robert Azana, Mel Krajden
EPI_ISL_2720402	A.S.L. VCO	Fondazione del Piemonte per l'Oncologia IRCCS	Antonino Sottile, Silvia Brossa, Paola Marino, Giorgio Giardina
EPI_ISL_2720404	A.S.L. VERCELLI	Fondazione del Piemonte per l'Oncologia IRCCS	Antonino Sottile, Silvia Brossa, Paola Marino, Giorgio Giardina
EPI_ISL_2720511	A.S.L. TO4	Fondazione del Piemonte per l'Oncologia IRCCS	Antonino Sottile, Silvia Brossa, Paola Marino, Giorgio Giardina
EPI_ISL_2720584, EPI_ISL_2720585, EPI_ISL_2720807, EPI_ISL_2721136	BCCDC Public Health Laboratory	BCCDC Public Health Laboratory	Prystajecky Natalie, Linda Hoang, Dan Fornika, John Tyson, Shannon Russell, Kim Macdonald, Kimia Kamelian, Ana Pacagnella, Corrinne Ng, Loretta Janz, Robert Azana, Mel Krajden
EPI_ISL_2721308	A.S.L. TO4	Fondazione del Piemonte per l'Oncologia IRCCS	Antonino Sottile, Silvia Brossa, Paola Marino, Giorgio Giardina
EPI_ISL_2721422, EPI_ISL_2721426, EPI_ISL_2721453, EPI_ISL_2721840, EPI_ISL_2721869	BCCDC Public Health Laboratory	BCCDC Public Health Laboratory	Prystajecky Natalie, Linda Hoang, Dan Fornika, John Tyson, Shannon Russell, Kim Macdonald, Kimia Kamelian, Ana Pacagnella, Corrinne Ng, Loretta Janz, Robert Azana, Mel Krajden
EPI_ISL_2725219, EPI_ISL_2725247, EPI_ISL_2725296	Lab. Microbiologia e Virologia Cotugno A.O. dei Colli - Istituto Zooprofilattico Sperimentale del Mezzogiorno	Telethon Institute of Genetics and Medicine (TIGEM)	Antonio Grimaldi Patrizia Annunziata Francesco Panariello Biancamaria Pierri Claudia Tiberio Teresa Giuliano Valentina Bouche Chiara Colantuono Maria Concetta Cuomo Denise Di Concilio Lucio Di Filippo Anna Manfredi Marcello Salvi Antonio Limone Luigi Atripaldi Pellegrino Cerino Andrea Ballabio Davide Cacchiarelli
EPI_ISL_2726488	KU Leuven, Rega Institute, Clinical and Epidemiological Virology	KU Leuven, Rega Institute, Clinical and Epidemiological Virology	Tony Wawina-Bokalanga, Bert Vanmechelen, Joan Marti-Carerras, Piet Maes
EPI_ISL_2727349	ZARV/NHLS, Department Medical Virology, University of Pretoria	KRISP, KZn Research Innovation and Sequencing Platform	Amy Strydom, Adriano Mendes, Micheala Davids, Sim Mayaphi and Marietjie Venter, Giandhari J, Pillay S, Naidoo Y, Ramphal U, Lessells R, Emmanuel SJ, Tegally H, Wilkinson E, de Oliveira T
EPI_ISL_2747989, EPI_ISL_2748655	Ohio Department of Health Laboratory	Ohio Department of Health Laboratory	Holmes, Jennifer; Eric Brandt, Keoni Omura, Glen McGillivary, Caitlin McDonnell, Jade Mowery, Stephanie Mccracken, Tyler Payne, Kirtana Ramadugu, Erica Leasure, Brent Lee, Kelsey Florek, Heather Blankenship, Allison Black, Quanta Brown, and Tammy Bannerman
EPI_ISL_2753948	NYU Langone Health	Departments of Pathology and Medicine, New York University School of Medicine	Adriana Heguy, Dacia Dimartino, Emily Guzman, Christian Marier, Peter Meyn, Sitharam Ramaswami, Gael Westby, Paul Zappile, Yutong Zhang, Paolo Cotzia, Guiqing Wang
EPI_ISL_2758356	QLabs	WVU and Marshall University Combined Genomics Core Facilities	James Denvir, Peter Stoilov, Peter Perrotta, Wesley Kimble, Ryan Percifield
EPI_ISL_2762285	MVZ Labor Dr. Fenner und Kollegen (Standort Hamburg)	Robert Koch Institute	unknown
EPI_ISL_2771492	Ohio Department of Health Laboratory	Ohio Department of Health Laboratory	Holmes, Jennifer; Eric Brandt, Keoni Omura, Glen McGillivary, Caitlin McDonnell, Jade Mowery, Stephanie Mccracken, Tyler Payne, Kirtana Ramadugu, Erica Leasure, Brent Lee, Kelsey Florek, Heather Blankenship, Allison Black, Quanta Brown, and Tammy Bannerman

Supp. Table S8

We gratefully acknowledge the following Authors from the Originating laboratories responsible for obtaining the specimens, as well as the Submitting laboratories where the genome data were generated and shared via GISAID, on which this research is based.

All Submitters of data may be contacted directly via www.gisaid.org

Authors are sorted alphabetically.

Accession ID	Originating Laboratory	Submitting Laboratory	Authors
EPI_ISL_2349561, EPI_ISL_2349577, EPI_ISL_2349580, EPI_ISL_2349597, EPI_ISL_2349608, EPI_ISL_2349652, EPI_ISL_2349653, EPI_ISL_2349682	Pandemic Response Lab - NYC	Pandemic Response Lab, R&D	Henry Lee, Michael Hammerling, Melissa Hopkins, Cybill del Castillo, Shinyoung Clair Kang, William Ward, Pradeep Bugga, Sol Rey, Dylan Law, Katharine Nelson, Haiping Hao, Jon Laurent
EPI_ISL_2362005	HOSPITAL UNIVERSITARIO SON ESPASES	HOSPITAL UNIVERSITARIO SON ESPASES	Carla López-Causapé, Pablo Fraile-Ribot, Antonio Oliver, SeqCovid
EPI_ISL_2370368, EPI_ISL_2370374, EPI_ISL_2370384, EPI_ISL_2370566, EPI_ISL_2370606, EPI_ISL_2370608, EPI_ISL_2370630, EPI_ISL_2370706	Aegis Sciences Corporation	Centers for Disease Control and Prevention Division of Viral Diseases, Pathogen Discovery	Dakota Howard, Dhwani Batra, Peter W. Cook, Kara Moser, Adrian Paskey, Jason Caravas, Benjamin Rambo-Martin, Shatavia Morrison, Christopher Gulvick, Scott Sammons, Yvette Unoarumhi, Darlene Wagner, Matthew Schmerer, Cyndi Clark, Patrick Campbell, Rob Case, Vikramsinha Ghorpade, Holly Houdeshell, Ola Kvalvaag, Dillon Nall, Ethan Sanders, Alec Vest, Shaun Westlund, Matthew Hardison, Clinton R. Paden, Duncan MacCannell
EPI_ISL_2371541, EPI_ISL_2371551, EPI_ISL_2371565	Fulgent Genetics	Centers for Disease Control and Prevention Division of Viral Diseases, Pathogen Discovery	Dakota Howard, Dhwani Batra, Peter W. Cook, Kara Moser, Adrian Paskey, Jason Caravas, Benjamin Rambo-Martin, Shatavia Morrison, Christopher Gulvick, Scott Sammons, Yvette Unoarumhi, Darlene Wagner, Matthew Schmerer, Harry Gao, Mickey Li, John Gao, Joseph Fierro, Benafsh Sapra, Becky Tsai, Yan Meng, Doreen Ng, James Xie, Clinton R. Paden, Duncan MacCannell
EPI_ISL_2380811, EPI_ISL_2380814	Clinical Molecular Microbiology Laboratory, UNC Hospitals	Jeremy Wang	Jeremy Wang, Alexander Rubinsteyn, Colleen Rice, Jason Smedberg, Shawn Hawken, Melissa Miller, Corbin Jones, Robert Hagan
EPI_ISL_2398063, EPI_ISL_2399345, EPI_ISL_2399360, EPI_ISL_2399361, EPI_ISL_2399362, EPI_ISL_2399369, EPI_ISL_2399370, EPI_ISL_2399371, EPI_ISL_2399382, EPI_ISL_2399393, EPI_ISL_2399396, EPI_ISL_2399409			
see above	Laboratory Corporation of America	Centers for Disease Control and Prevention Division of Viral Diseases, Pathogen Discovery	Dakota Howard, Dhwani Batra, Peter W. Cook, Kara Moser, Adrian Paskey, Jason Caravas, Benjamin Rambo-Martin, Shatavia Morrison, Christopher Gulvick, Scott Sammons, Yvette Unoarumhi, Darlene Wagner, Matthew Schmerer, Minoo Agarwal, Eyad Almasri, Debbie Boles, Ayla Burns, Nuthawin Charoensri, Oren Cohen, Susan Countryman, Mary Ann Cristobal, Bobbi Croy, Suzanne Dale, Hrushikesh Deshmukh, Amanda Douglas, Vincent Drouillon, Marcia Eisenberg, Howard Engler, Rama Ghatti, Prashant Gupta, Susan Hicks, Jake Humphrey, Lax Iyer, Lisa Pfefferle, Manoj Jain, Matthew Robinson, Mohan Kolli, Brian Krueger, Tim Kuphal, Stanley Letovsky, Michael Levandoski, Craig Lukasik, Jonathan Meltzer, Brian Norvell, Mindy Nye, Scott Parker, Christos Petropoulos, John Pruitt, Steven Ragan, Scott Ryan, Mike Sapeta, Jana Schroth, Suresh Babu Selvaraju, Goran Stevovic, Amanda Suchanek, Andrea Throop, Lyndon Tilson, Thomas Urban, Joe Voshell, Kimberly Wagner, Jonathan Williams, Mary Williamson, Qian Zeng, Tricia Zwiefelhofer, Clinton R. Paden, Duncan MacCannell
EPI_ISL_2400241	Wichita State University - Molecular Diagnostics Lab	Kansas Health and Environmental Lab	Mike Grose, Katherine Wiggins, Jonathan Barnell, Ben Olsen, and Phil Adam
EPI_ISL_2405148, EPI_ISL_2405149	INTERLAB	Omics Sciences Laboratory	Derly Andrade Molina, Gabriel Morey León, Darlyn Amaya, Rubén Armas González, Juan Carlos Fernández Cadena
EPI_ISL_2405151	Hospital Luis Vernaza	Omics Sciences Laboratory	Derly Andrade Molina, Gabriel Morey León, Darlyn Amaya, Rubén Armas González, Juan Carlos Fernández Cadena
EPI_ISL_2405153	Omics Sciences Laboratory	Omics Sciences Laboratory	Derly Andrade Molina, Gabriel Morey León, Darlyn Amaya, Rubén Armas González, Juan Carlos Fernández Cadena
EPI_ISL_2405156, EPI_ISL_2405164, EPI_ISL_2405166	Hospital Luis Vernaza	Omics Sciences Laboratory	Derly Andrade Molina, Gabriel Morey León, Darlyn Amaya, Rubén Armas González, Juan Carlos Fernández Cadena
EPI_ISL_2405548, EPI_ISL_2405595, EPI_ISL_2405629	Dutch COVID-19 response team	National Institute for Public Health and the Environment (RIVM)	Adam Meijer, Harry Vennema, Dirk Eggink, Jeroen Cremer, Sharon van den Brink, Bas van der Veer, AnneMarie van den Brandt, Lisa Wijsman, Kim Freriks, Ryanne Jaarsma, Euníce Then, Lynn Aarts, Sanne Bos, Melissa van Tuil, Linda van de Nes, Sjoerd Kuiling, James Groot, Florian Zwagemaker, Dennis Schmitz, Annelies Kroneman, Karim Hajji, Chantal Reusken, on behalf of the national COVID-19 response team
EPI_ISL_2420987	Montana Public Health Laboratory	Montana Public Health Laboratory	Joy Ritter, Michelle Mozer, Carrie Biskupiak, Deborah Gibson, Michael Dills
EPI_ISL_2422013, EPI_ISL_2422025, EPI_ISL_2422044, EPI_ISL_2422109, EPI_ISL_2422118	Aegis Sciences Corporation	Centers for Disease Control and Prevention Division of Viral Diseases, Pathogen Discovery	Dakota Howard, Dhwani Batra, Peter W. Cook, Kara Moser, Adrian Paskey, Jason Caravas, Benjamin Rambo-Martin, Shatavia Morrison, Christopher Gulvick, Scott Sammons, Yvette Unoarumhi, Darlene Wagner, Matthew Schmerer, Cyndi Clark, Patrick Campbell, Rob Case, Vikramsinha Ghorpade, Holly Houdeshell, Ola Kvalvaag, Dillon Nall, Ethan Sanders, Alec Vest, Shaun Westlund, Matthew Hardison, Clinton R. Paden, Duncan MacCannell
EPI_ISL_2424257, EPI_ISL_2424261	Oregon State Public Health Laboratory	Oregon State Public Health Laboratory	Rafia Razzaque, Eugene Yeboah, Vanda Makris, Laura Tsaknaridis, John Fontana and Shane Sevey
EPI_ISL_2425398, EPI_ISL_2425399	Minnesota Department of Health, Public Health Laboratory	Minnesota Department of Health, Public Health Laboratory	Alexandra Lorentz, Jacob Garfin, Matt Plumb, and Xiong Wang
EPI_ISL_2427480	NYU Langone Health	Departments of Pathology and Medicine, New York University School of Medicine	Adriana Heguy, Dacia Dimartino, Emily Guzman, Christian Marier, Peter Meyn, Sitharam Ramaswami, Gael Westby, Paul Zappile, Yutong Zhang, Paolo Cotzia, Guiqing Wang
EPI_ISL_2432609, EPI_ISL_2432626, EPI_ISL_2432648	UW Virology Lab	UW Virology Lab	Pavitra Roychoudhury, Hong Xie, Lasata Shrestha, Tien V. Nguyen, Shah Mohamed Bakhash, Michelle Lin, Noah R. Baker, Ricardo Perez, Sean Ellis, Nathan Breit, Robert J. Livingston, Meei-Li Huang, Keith R Jerome, Patrick Mathias, Alexander Greninger
EPI_ISL_2438698, EPI_ISL_2438717, EPI_ISL_2438725	HOSPITAL UNIVERSITARIO SON ESPASES	HOSPITAL UNIVERSITARIO SON ESPASES	Servicio de Microbiología, Hospital Universitario Son Espases
EPI_ISL_2439546, EPI_ISL_2439703, EPI_ISL_2440635, EPI_ISL_2440726, EPI_ISL_2440792, EPI_ISL_2440810, EPI_ISL_2440818, EPI_ISL_2440826, EPI_ISL_2440852, EPI_ISL_2440872, EPI_ISL_2440887, EPI_ISL_2440890			
see above	Quest Diagnostics Incorporated	Centers for Disease Control and Prevention Division of Viral Diseases, Pathogen Discovery	Dakota Howard, Dhwani Batra, Peter W. Cook, Kara Moser, Adrian Paskey, Jason Caravas, Benjamin Rambo-Martin, Shatavia Morrison, Christopher Gulvick, Scott Sammons, Yvette Unoarumhi, Darlene Wagner, Matthew Schmerer, S. H. Rosenthal, A. Gerasimova, R. M. Kagan, B. Anderson, M. Hua, Y. Liu, L.E. Bernstein, K.E. Livingston, A. Perez, I. A. Shlyakhter, R. V. Rolando, R. Owen, P. Tanpaiboon, F. Lacbawan, Clinton R. Paden, Duncan MacCannell
EPI_ISL_2441920	Fulgent Genetics	Centers for Disease Control and Prevention Division of Viral Diseases, Pathogen Discovery	Dakota Howard, Dhwani Batra, Peter W. Cook, Kara Moser, Adrian Paskey, Jason Caravas, Benjamin Rambo-Martin, Shatavia Morrison, Christopher Gulvick, Scott Sammons, Yvette Unoarumhi, Darlene Wagner, Matthew Schmerer, Harry Gao, Mickey Li, John Gao, Joseph Fierro, Benafsh Sapra, Becky Tsai, Yan Meng, Doreen Ng, James Xie, Clinton R. Paden, Duncan MacCannell
EPI_ISL_2443110, EPI_ISL_2443125	NJDOH, Public Health and Environmental Laboratories	NJ_PHEL	Lindsey Bodnar, Shiv K. Verma, Ryan Pachucki, Chelsea San Filippo, Dana Woell, Allison Roder, Byeong Jeong
EPI_ISL_2443167	IN State Department of Health Laboratory Services	IN State Department of Health Laboratory Services	Cassandra Campion, Jamie Yeadon, Brian Pope, Lixia Liu, Kyle Brownlee, Melissa Hindenlang, Mark Glazier
EPI_ISL_2443851, EPI_ISL_2443852	Minnesota Department of Health, Public Health Laboratory	Minnesota Department of Health, Public Health Laboratory	Alexandra Lorentz, Jacob Garfin, Matt Plumb, and Xiong Wang
EPI_ISL_2444439, EPI_ISL_2444447	Salud Digna	Instituto Nacional de Medicina Genomica	Hidalgo-Miranda A, Cedro-Tanda A, Mendoza-Vargas A, Reyes-Grajeda JP, Abraham Campos-Romero, Moreno-Camacho José Luis, Rodriguez-Gallegos Jorge, Luna-Ruiz Marco, Gonzalez-Barrera D, Rangel-DeLeon D, Munguia-Garza P, Ramirez-Vega O, Escobar-Arrazola, M, Herrera-Montalvo LA.
EPI_ISL_2446040, EPI_ISL_2446042, EPI_ISL_2446048, EPI_ISL_2446054, EPI_ISL_2446056, EPI_ISL_2446067, EPI_ISL_2446074, EPI_ISL_2446079, EPI_ISL_2446096	Pandemic Response Lab - NYC	Pandemic Response Lab, R&D	Henry Lee, Michael Hammerling, Melissa Hopkins, Cybill del Castillo, Shinyoung Clair Kang, William Ward, Pradeep Bugga, Sol Rey, Dylan Law, Katharine Nelson, Haiping Hao, Jon Laurent
EPI_ISL_2448099	Microbiology Department, Laboratori Clínic Metropolitana	Can Ruti SARS-CoV-2 Sequencing Hub	

Nord. Hospital Universitari Germans Trias i Pujol	(HUGTiP/IrsiCaixa/IGTP)		Marc Noguera-Julian, Pilar Armengol, Ignacio Blanco, Antoni E Bordoy, Francesc Catala-Moll, Pere-Joan Cardona, Maria Casadell, Cristina Casa, Gemma Clara, Bonaventura Clotet, Cristina Esteban, Montserrat Gimnez, Mercedes Guerrero, Anna Not, Roger Paredes, Mariona Parera, Vernica Saludes, Alba S nchez, and Elisa Martron behalf of the Can Ruti SARS-CoV-2 Sequencing Hub.
EPI_ISL_2451268, EPI_ISL_2451269, EPI_ISL_2451277, EPI_ISL_2451317, EPI_ISL_2451325, EPI_ISL_2451326, EPI_ISL_2451332, EPI_ISL_2451335, EPI_ISL_2451340, EPI_ISL_2451344, EPI_ISL_2451355, EPI_ISL_2451360, EPI_ISL_2451367, EPI_ISL_2451372, EPI_ISL_2451387, EPI_ISL_2451392, EPI_ISL_2451394, EPI_ISL_2451399, EPI_ISL_2451401			
see above	Quest Diagnostics Incorporated	Centers for Disease Control and Prevention Division of Viral Diseases, Pathogen Discovery	Dakota Howard, Dhwani Batra, Peter W. Cook, Kara Moser, Adrian Paskey, Jason Caravas, Benjamin Rambo-Martin, Shatavia Morrison, Christopher Gulvick, Scott Sammons, Yvette Unoarumhi, Darlene Wagner, Matthew Schmerer, S. H. Rosenthal, A. Gerasimova, R. M. Kagan, B. Anderson, M. Hua, Y. Liu, L.E. Bernstein, K.E. Livingston, A. Perez, I. A. Shlyakhter, R. V. Rolando, R. Owen, P. Tanpaiboon, F. Lacbawan, Clinton R. Paden, Duncan MacCannell
EPI_ISL_2455301	Johns Hopkins Hospital Department of Pathology	Johns Hopkins Hospital Department of Pathology	C. Paul Morris, Chun Huai Luo, Adannaya Amadi, Matthew Schwartz, Nicholas Gallagher, Heba H. Mostafa
EPI_ISL_2455346, EPI_ISL_2455351, EPI_ISL_2455355, EPI_ISL_2455361	Minnesota Department of Health, Public Health Laboratory	Minnesota Department of Health, Public Health Laboratory	Alexandra Lorentz, Jacob Garfin, Matt Plumb, and Xiong Wang
EPI_ISL_2455380	HealthPartners Central Lab	Minnesota Department of Health, Public Health Laboratory	Alexandra Lorentz, Jacob Garfin, Matt Plumb, and Xiong Wang
EPI_ISL_2458208	NYU Langone Health	Departments of Pathology and Medicine, New York University School of Medicine	Adriana Heguy, Dacia Dimartino, Emily Guzman, Christian Marier, Peter Meyn, Sitharam Ramaswami, Gael Westby, Paul Zappile, Yutong Zhang, Paolo Cotzia, Guiqing Wang
EPI_ISL_2463036, EPI_ISL_2463039, EPI_ISL_2463044, EPI_ISL_2463063, EPI_ISL_2463065, EPI_ISL_2463090, EPI_ISL_2463122, EPI_ISL_2463130, EPI_ISL_2463136, EPI_ISL_2463145, EPI_ISL_2463165, EPI_ISL_2463173, EPI_ISL_2463195, EPI_ISL_2463197			
see above	Quest Diagnostics Incorporated	Centers for Disease Control and Prevention Division of Viral Diseases, Pathogen Discovery	Dakota Howard, Dhwani Batra, Peter W. Cook, Kara Moser, Adrian Paskey, Jason Caravas, Benjamin Rambo-Martin, Shatavia Morrison, Christopher Gulvick, Scott Sammons, Yvette Unoarumhi, Darlene Wagner, Matthew Schmerer, S. H. Rosenthal, A. Gerasimova, R. M. Kagan, B. Anderson, M. Hua, Y. Liu, L.E. Bernstein, K.E. Livingston, A. Perez, I. A. Shlyakhter, R. V. Rolando, R. Owen, P. Tanpaiboon, F. Lacbawan, Clinton R. Paden, Duncan MacCannell
EPI_ISL_2464863	Biogroup Bio Lam-LCD Saint-Denis	Department of Virology, Henri Mondor University Hospital, Assistance Publique Hôpitaux de Paris, Université Paris-Est Créteil, INSERM U955	Christophe Rodriguez, Slim Fourati, Vanessa Demontant, Guillaume Gricourt, Melissa N'Debi, Alexandre Soulier, Elisabeth Trawinski, Jean-Michel Pawlotsky
EPI_ISL_2466670	Stony Brook Medicine	New York Genome Center	Michael Zody, Andre Corvelo, Dayna M. Oschwald, Samantha Fennessey, Tom Maniatis , Sarah A. Halbohn, Alaa B. Salaheldin, Sarah A. Malik, Josue M. Flores Acuna, Dimitri Gnatenko, Silvia G. Spitzer, Margaret M. McGovern, Eric D. Spitzer, Richard R. Kew, Kenneth R. Shroyer, Jiang Chen, Susan V. Donelan
EPI_ISL_2466695	Sherman Abrams Laboratory	New York City Public Health Laboratory	Jade Wang, et al.
EPI_ISL_2466697	Department of Homeless Services	New York City Public Health Laboratory	Jade Wang, et al.
EPI_ISL_2466700	OCME Office Of Chief Medical Examiner	New York City Public Health Laboratory	Jade Wang, et al.
EPI_ISL_2470118	LabKom - Labor an der Salzbrücke MVZ GmbH	Robert Koch Institute	unknown
EPI_ISL_2473668, EPI_ISL_2473669, EPI_ISL_2473673, EPI_ISL_2473679	Oregon State Public Health Laboratory	Oregon State Public Health Laboratory	Rafia Razzaque, Eugene Yeboah, Vanda Makris, Laura Tsaknaridis, John Fontana and Shane Sevey
EPI_ISL_2476337, EPI_ISL_2476341	Dutch COVID-19 response team	National Institute for Public Health and the Environment (RIVM)	Adam Meijer, Harry Vennema, Dirk Eggink, Jeroen Cremer, Sharon van den Brink, Bas van der Veer, AnneMarie van den Brandt, Lisa Wijsman, Kim Freriks, Ryanne Jaarsma, Euníce Then, Lynn Aarts, Sanne Bos, Melissa van Tuil, Linda van de Nes, Florian Zwagemaker, Dennis Schmitz, Annelies Kroneman, Karim Hajji, Chantal Reusken, on behalf of the national COVID-19 response team
EPI_ISL_2480024, EPI_ISL_2480094	Fulgent Genetics	Centers for Disease Control and Prevention Division of Viral Diseases, Pathogen Discovery	Dakota Howard, Dhwani Batra, Peter W. Cook, Kara Moser, Adrian Paskey, Jason Caravas, Benjamin Rambo-Martin, Shatavia Morrison, Christopher Gulvick, Scott Sammons, Yvette Unoarumhi, Darlene Wagner, Matthew Schmerer, Harry Gao, Mickey Li, John Gao, Joseph Fierro, Benafsh Sapra, Becky Tsai, Yan Meng, Doreen Ng, James Xie, Clinton R. Paden, Duncan MacCannell
EPI_ISL_2480205, EPI_ISL_2480206, EPI_ISL_2480219, EPI_ISL_2480228, EPI_ISL_2480231, EPI_ISL_2480249, EPI_ISL_2480257, EPI_ISL_2480291, EPI_ISL_2480296, EPI_ISL_2480320, EPI_ISL_2480327, EPI_ISL_2480328, EPI_ISL_2480340, EPI_ISL_2480386, EPI_ISL_2480388, EPI_ISL_2480412, EPI_ISL_2480463, EPI_ISL_2480464, EPI_ISL_2480466, EPI_ISL_2480480, EPI_ISL_2480482, EPI_ISL_2480496, EPI_ISL_2480497, EPI_ISL_2480498, EPI_ISL_2480503, EPI_ISL_2480508, EPI_ISL_2480514, EPI_ISL_2480518, EPI_ISL_2480523, EPI_ISL_2480529, EPI_ISL_2480788, EPI_ISL_2480795, EPI_ISL_2480802, EPI_ISL_2480813, EPI_ISL_2480820, EPI_ISL_2480833, EPI_ISL_2480838, EPI_ISL_2480843, EPI_ISL_2480850, EPI_ISL_2480879, EPI_ISL_2480885, EPI_ISL_2480894, EPI_ISL_2480897, EPI_ISL_2480916, EPI_ISL_2480925, EPI_ISL_2480937, EPI_ISL_2480946, EPI_ISL_2480947, EPI_ISL_2480948, EPI_ISL_2480950, EPI_ISL_2480958, EPI_ISL_2480966, EPI_ISL_2480971, EPI_ISL_2481122, EPI_ISL_2481139, EPI_ISL_2481150, EPI_ISL_2481160, EPI_ISL_2481176, EPI_ISL_2481198, EPI_ISL_2481210, EPI_ISL_2481226, EPI_ISL_2481227, EPI_ISL_2481228, EPI_ISL_2481239, EPI_ISL_2481241, EPI_ISL_2481242, EPI_ISL_2481246, EPI_ISL_2481271, EPI_ISL_2481275, EPI_ISL_2481277, EPI_ISL_2481284, EPI_ISL_2481304, EPI_ISL_2481305, EPI_ISL_2481306, EPI_ISL_2481315, EPI_ISL_2481316, EPI_ISL_2481317, EPI_ISL_2481392, EPI_ISL_2481407, EPI_ISL_2481436, EPI_ISL_2481439, EPI_ISL_2481440, EPI_ISL_2481444, EPI_ISL_2481452, EPI_ISL_2481453, EPI_ISL_2481457, EPI_ISL_2481482, EPI_ISL_2481483, EPI_ISL_2481584, EPI_ISL_2481585, EPI_ISL_2481586, EPI_ISL_2481629, EPI_ISL_2481665, EPI_ISL_2481675, EPI_ISL_2481735, EPI_ISL_2481747, EPI_ISL_2481773, EPI_ISL_2481791, EPI_ISL_2481818, EPI_ISL_2481824, EPI_ISL_2481863, EPI_ISL_2481873, EPI_ISL_2481923, EPI_ISL_2481924, EPI_ISL_2481925, EPI_ISL_2481978, EPI_ISL_2481983, EPI_ISL_2481995, EPI_ISL_2482005, EPI_ISL_2482019, EPI_ISL_2482028, EPI_ISL_2482071, EPI_ISL_2482091, EPI_ISL_2482095, EPI_ISL_2482098, EPI_ISL_2482132, EPI_ISL_2482187, EPI_ISL_2482207, EPI_ISL_2482258, EPI_ISL_2482265, EPI_ISL_2482268			
see above	Laboratory Corporation of	Centers for Disease	Dakota Howard, Dhwani Batra, Peter W. Cook, Kara Moser, Adrian Paskey, Jason

	America	Control and Prevention Division of Viral Diseases, Pathogen Discovery	Caravas, Benjamin Rambo-Martin, Shatavia Morrison, Christopher Gulvick, Scott Sammons, Yvette Unoarumhi, Darlene Wagner, Matthew Schmerer, Minoo Agarwal, Eyad Almasri, Debbie Boles, Ayla Burns, Nuthawin Charoensri, Oren Cohen, Susan Countryman, Mary Ann Cristobal, Bobbi Croy, Suzanne Dale, Hrushikesh Deshmukh, Amanda Douglas, Vincent Drouillon, Marcia Eisenberg, Howard Engler, Rama Ghatti, Prashant Gupta, Susan Hicks, Jake Humphrey, Lax Iyer, Lisa Pfefferle, Manoj Jain, Matthew Robinson, Mohan Kolli, Brian Krueger, Tim Kuphal, Stanley Letovsky, Michael Levandoski, Craig Lukasik, Jonathan Meltzer, Brian Norvell, Mindy Nye, Scott Parker, Christos Petropoulos, John Pruitt, Steven Ragan, Scott Ryan, Mike Sapeta, Jana Schroth, Suresh Babu Selvaraju, Goran Stevovic, Amanda Suchanek, Andrea Throop, Lyndon Tilson, Thomas Urban, Joe Voshell, Kimberly Wagner, Jonathan Williams, Mary Williamson, Qian Zeng, Tricia Zwiefelhofer, Clinton R. Paden, Duncan MacCannell
EPI_ISL_2484115	Arizona State University	Arizona State University	Peter T. Skidmore, LaRinda A. Holland, Matthew F. Smith, Rabia Maqsood, Nicholas J. Mellor, Joy M. Blain, Valerie Harris, Joshua LaBaer, Vel Murugan, Efrem S. Lim
EPI_ISL_2489070, EPI_ISL_2489071, EPI_ISL_2489111, EPI_ISL_2489140, EPI_ISL_2489179, EPI_ISL_2489180, EPI_ISL_2489188, EPI_ISL_2489254, EPI_ISL_2489256, EPI_ISL_2489259, EPI_ISL_2489264, EPI_ISL_2489276, EPI_ISL_2489287, EPI_ISL_2489296, EPI_ISL_2489299, EPI_ISL_2489304, EPI_ISL_2489310, EPI_ISL_2489311, EPI_ISL_2489326, EPI_ISL_2489333, EPI_ISL_2489336, EPI_ISL_2489339, EPI_ISL_2489359, EPI_ISL_2489388, EPI_ISL_2489399, EPI_ISL_2489400, EPI_ISL_2489408, EPI_ISL_2489466, EPI_ISL_2489467, EPI_ISL_2489468, EPI_ISL_2489469, EPI_ISL_2489484, EPI_ISL_2489487, EPI_ISL_2489556, EPI_ISL_2489563, EPI_ISL_2489569, EPI_ISL_2489571, EPI_ISL_2489586, EPI_ISL_2489595, EPI_ISL_2489596, EPI_ISL_2489600, EPI_ISL_2489606, EPI_ISL_2489641, EPI_ISL_2489644, EPI_ISL_2489660, EPI_ISL_2489661, EPI_ISL_2489663, EPI_ISL_2489669, EPI_ISL_2489738, EPI_ISL_2489744, EPI_ISL_2489745, EPI_ISL_2489747, EPI_ISL_2489772, EPI_ISL_2489775, EPI_ISL_2489778, EPI_ISL_2489780, EPI_ISL_2489794, EPI_ISL_2489796, EPI_ISL_2489799			
see above	Aegis Sciences Corporation	Centers for Disease Control and Prevention Division of Viral Diseases, Pathogen Discovery	Dakota Howard, Dhwani Batra, Peter W. Cook, Kara Moser, Adrian Paskey, Jason Caravas, Benjamin Rambo-Martin, Shatavia Morrison, Christopher Gulvick, Scott Sammons, Yvette Unoarumhi, Darlene Wagner, Matthew Schmerer, Cyndi Clark, Patrick Campbell, Rob Case, Vikramsinha Ghorpade, Holly Houdeshell, Ola Kvalvaag, Dillon Nall, Ethan Sanders, Alec Vest, Shaun Westlund, Matthew Hardison, Clinton R. Paden, Duncan MacCannell
EPI_ISL_2492341	Institute of Microbiology, Universidad San Francisco de Quito	Institute of Microbiology, Universidad San Francisco de Quito	Belén Prado-Vivar, Sully Márquez, Juan José Guadalupe, Monica Becerra-Wong, Fernanda Zurita, Bernardo Gutiérrez, Luis Morales, Verónica Barragán, Patricio Rojas-Silva, Gabriel Trueba, Michelle Grunauer, Paúl Cárdenas
EPI_ISL_2492353	Institute of Microbiology, Universidad San Francisco de Quito	Institute of Microbiology, Universidad San Francisco de Quito	Fernanda Zurita, Sully Márquez, Juan José Guadalupe, Belén Prado-Vivar, Monica Becerra-Wong, Bernardo Gutiérrez, Lofrein Vera, Verónica Barragán, Patricio Rojas-Silva, Gabriel Trueba, Michelle Grunauer, Paúl Cárdenas
EPI_ISL_2495707, EPI_ISL_2495708, EPI_ISL_2495710, EPI_ISL_2495711, EPI_ISL_2495712	MB-Cadham Provincial laboratory	National Microbiology Laboratory (NML)	Anna Majer, Shari Tyson, Grace Seo, Philip Mabon, Elsie Grudeski, Rhiannon Huzarewich, Russell Mandes, Anneliese Landgraff, Jennifer Tanner, Natalie Knox, Morag Graham, Gary Van Domselaar, Paul Van Caeseele, Jared Bullard, David Alexander, Kerry Dust, Nathalie Bastien, Yan Li, Timothy Booth, Darian Hole, Madison Chapel, Kirsten Biggar, CanCOGeN's metadata curation team, Public Health Agency of Canada CanCOGeN team
EPI_ISL_2495921	Nacionaline visuomenes sveikatos prieziuros laboratorija	Vilnius University Hospital Santaros Klinikos, Center of Laboratory Medicine	Ligita Raugaite, Monika Katenaite, Rimvydas Norvilas, Dovile Ezerskyte, Ingrida Olendraite, Gytis Dudas, Daniel Naumovas
EPI_ISL_2496092	North Dakota Department of Health, Public Health Laboratory	North Dakota Department of Health, Public Health Laboratory	Lisa Wingerter
EPI_ISL_2497192, EPI_ISL_2497224, EPI_ISL_2497270, EPI_ISL_2497317, EPI_ISL_2497417, EPI_ISL_2497427	UW Virology Lab	UW Virology Lab	Pavitra Roychoudhury, Hong Xie, Lasata Shrestha, Tien V. Nguyen, Shah Mohamed Bakhash, Michelle Lin, Noah R. Baker, Ricardo Perez, Sean Ellis, Nathan Breit, Robert J. Livingston, Meei-Li Huang, Keith R Jerome, Patrick Mathias, Alexander Greninger
EPI_ISL_2497892	University of Mississippi Medical Center, Department of Pathology	University of Mississippi Medical Center, Molecular and Genomics Core Facility	Ashley C. Johnson, Ithiel J. Frame, Krishna K. Ayyalasomayajula, Michael R. Garrett, D. Ashley Robinson
EPI_ISL_2497926, EPI_ISL_2497927	UW Virology Lab	UW Virology Lab	Pavitra Roychoudhury, Hong Xie, Lasata Shrestha, Tien V. Nguyen, Shah Mohamed Bakhash, Michelle Lin, Noah R. Baker, Ricardo Perez, Sean Ellis, Nathan Breit, Robert J. Livingston, Meei-Li Huang, Keith R Jerome, Patrick Mathias, Alexander Greninger
EPI_ISL_2499856	DC Public Health Lab/ Dept. of Forensic Sciences	DC Public Health Lab/ Dept. of Forensic Sciences	Janis Doss, Scott Nguyen, Elizabeth Zelaya, Sarah Scott, Connie Maza, Monica Mann, Brittany Hamilton, David Payne, Jocelyn Hauser
EPI_ISL_2500459	Hackensack Medical Center	New York Genome Center	Michael Zody, Andre Corvelo, Dayna M. Oschwald, Samantha Fennessey, Tom Maniatis, Liang Chen, Jose Mediavilla, Marcus Cunningham, Kaelea Composto, Kar Chow, David Perlin, Barry Kreiswirth
EPI_ISL_2501004, EPI_ISL_2501013	Gundersen Clinical Microbiology Laboratory	Kabara Cancer Research Institute	Craig S. Richmond, Paraic A. Kenny
EPI_ISL_2501377, EPI_ISL_2501378	Microbiology Department. Complexo Hospitalario Universitario de Vigo	Microbiology Department. Complexo Hospitalario Universitario de Vigo	Alfaya N, Alonso I, Alvarez M, Cabrera JJ, Carballo R, Cores O, Cortizo S, del-Campo V, Martinez L, Mediero G, Perez S, Potel C, Regueiro B, Rey S, Vasallo FJ
EPI_ISL_2501498, EPI_ISL_2501508, EPI_ISL_2501517, EPI_ISL_2501518, EPI_ISL_2501519, EPI_ISL_2501520, EPI_ISL_2501521, EPI_ISL_2501522	UW Virology Lab	UW Virology Lab	Pavitra Roychoudhury, Hong Xie, Lasata Shrestha, Tien V. Nguyen, Shah Mohamed Bakhash, Michelle Lin, Noah R. Baker, Ricardo Perez, Sean Ellis, Nathan Breit, Robert J. Livingston, Meei-Li Huang, Keith R Jerome, Patrick Mathias, Alexander Greninger

EPI_ISL_2501539	CT Department of Public Health	CT Department of Public Health	Claire_Pearson,Tu_N_Nguyen
EPI_ISL_2501696, EPI_ISL_2501706, EPI_ISL_2501727	Yale Clinical Virology Lab	Grubaugh Lab - Yale School of Public Health	Joseph Fauver, Mallery Breban, Isabel Ott, Tara Alpert, Mary Petrone, Anderson Brito, Chantal Vogels, Annie Watkins, Chaney Kalinich, Jessica Rothman, Marie L. Landry, Nathan Grubaugh
EPI_ISL_2503994, EPI_ISL_2503996	Alaska State Virology Laboratory	Alaska State Virology Laboratory	Stephanie DeRonde, Elva House, Jacob Zidek, Lisa Smith, Ph.D., Jack Chen, Ph.D.
EPI_ISL_2508533	Microbiology Division, SC DHEC	Microbiology Division, SC DHEC	Freeman,J., Flores,H.
EPI_ISL_2525599, EPI_ISL_2525754	Fulgent Genetics	Centers for Disease Control and Prevention Division of Viral Diseases, Pathogen Discovery	Dakota Howard, Dhwani Batra, Peter W. Cook, Kara Moser, Adrian Paskey, Jason Caravas, Benjamin Rambo-Martin, Shatavia Morrison, Christopher Gulvick, Scott Sammons, Yvette Unoarumhi, Darlene Wagner, Matthew Schmerer, Harry Gao, Mickey Li, John Gao, Joseph Fierro, Benafsh Sapra, Becky Tsai, Yan Meng, Doreen Ng, James Xie, Clinton R. Paden, Duncan MacCannell
EPI_ISL_2526772, EPI_ISL_2526783, EPI_ISL_2526811, EPI_ISL_2526816, EPI_ISL_2526827, EPI_ISL_2526844, EPI_ISL_2526867, EPI_ISL_2526875, EPI_ISL_2526976, EPI_ISL_2526994, EPI_ISL_2527028, EPI_ISL_2527034			
see above	Quest Diagnostics Incorporated	Centers for Disease Control and Prevention Division of Viral Diseases, Pathogen Discovery	Dakota Howard, Dhwani Batra, Peter W. Cook, Kara Moser, Adrian Paskey, Jason Caravas, Benjamin Rambo-Martin, Shatavia Morrison, Christopher Gulvick, Scott Sammons, Yvette Unoarumhi, Darlene Wagner, Matthew Schmerer, S. H. Rosenthal, A. Gerasimova, R. M. Kagan, B. Anderson, M. Hua, Y. Liu, L.E. Bernstein, K.E. Livingston, A. Perez, I. A. Shlyakhter, R. V. Rolando, R. Owen, P. Tanpaiboon, F. Lacbawan, Clinton R. Paden, Duncan MacCannell
EPI_ISL_2527594, EPI_ISL_2527601, EPI_ISL_2527714, EPI_ISL_2527745, EPI_ISL_2527831	Fulgent Genetics	Centers for Disease Control and Prevention Division of Viral Diseases, Pathogen Discovery	Dakota Howard, Dhwani Batra, Peter W. Cook, Kara Moser, Adrian Paskey, Jason Caravas, Benjamin Rambo-Martin, Shatavia Morrison, Christopher Gulvick, Scott Sammons, Yvette Unoarumhi, Darlene Wagner, Matthew Schmerer, Harry Gao, Mickey Li, John Gao, Joseph Fierro, Benafsh Sapra, Becky Tsai, Yan Meng, Doreen Ng, James Xie, Clinton R. Paden, Duncan MacCannell
EPI_ISL_2527904, EPI_ISL_2527929, EPI_ISL_2528008, EPI_ISL_2528010, EPI_ISL_2528058, EPI_ISL_2528123, EPI_ISL_2528173, EPI_ISL_2528199, EPI_ISL_2528257, EPI_ISL_2528314, EPI_ISL_2528354, EPI_ISL_2528430, EPI_ISL_2528464, EPI_ISL_2528472, EPI_ISL_2528479, EPI_ISL_2528486, EPI_ISL_2528510, EPI_ISL_2528513, EPI_ISL_2528528, EPI_ISL_2528530, EPI_ISL_2528659, EPI_ISL_2528668, EPI_ISL_2528670, EPI_ISL_2528686, EPI_ISL_2528714, EPI_ISL_2528800, EPI_ISL_2528898, EPI_ISL_2528900, EPI_ISL_2528916, EPI_ISL_2528927, EPI_ISL_2528929, EPI_ISL_2528931, EPI_ISL_2528951			
see above	Aegis Sciences Corporation	Centers for Disease Control and Prevention Division of Viral Diseases, Pathogen Discovery	Dakota Howard, Dhwani Batra, Peter W. Cook, Kara Moser, Adrian Paskey, Jason Caravas, Benjamin Rambo-Martin, Shatavia Morrison, Christopher Gulvick, Scott Sammons, Yvette Unoarumhi, Darlene Wagner, Matthew Schmerer, Cyndi Clark, Patrick Campbell, Rob Case, Vikramsinha Ghorpade, Holly Houdeshell, Ola Kvalvaag, Dillon Nall, Ethan Sanders, Alec Vest, Shaun Westlund, Matthew Hardison, Clinton R. Paden, Duncan MacCannell
EPI_ISL_2529102, EPI_ISL_2529104, EPI_ISL_2529152, EPI_ISL_2529190, EPI_ISL_2529219, EPI_ISL_2529244, EPI_ISL_2529256, EPI_ISL_2529276, EPI_ISL_2529293, EPI_ISL_2529310, EPI_ISL_2529335			
see above	Quest Diagnostics Incorporated	Centers for Disease Control and Prevention Division of Viral Diseases, Pathogen Discovery	Dakota Howard, Dhwani Batra, Peter W. Cook, Kara Moser, Adrian Paskey, Jason Caravas, Benjamin Rambo-Martin, Shatavia Morrison, Christopher Gulvick, Scott Sammons, Yvette Unoarumhi, Darlene Wagner, Matthew Schmerer, S. H. Rosenthal, A. Gerasimova, R. M. Kagan, B. Anderson, M. Hua, Y. Liu, L.E. Bernstein, K.E. Livingston, A. Perez, I. A. Shlyakhter, R. V. Rolando, R. Owen, P. Tanpaiboon, F. Lacbawan, Clinton R. Paden, Duncan MacCannell
EPI_ISL_2534389, EPI_ISL_2534397, EPI_ISL_2534400	HOSPITAL UNIVERSITARIO SON ESPASES	HOSPITAL UNIVERSITARIO SON ESPASES	Servicio de Microbiología, Hospital Universitario Son Espases
EPI_ISL_2534404	HOSPITAL CAN MISSES	HOSPITAL UNIVERSITARIO SON ESPASES	Servicio de Microbiología, Hospital Universitario Son Espases
EPI_ISL_2534405, EPI_ISL_2534407, EPI_ISL_2534415, EPI_ISL_2534417, EPI_ISL_2534425	HOSPITAL UNIVERSITARIO SON ESPASES	HOSPITAL UNIVERSITARIO SON ESPASES	Servicio de Microbiología, Hospital Universitario Son Espases
EPI_ISL_2536950	Centracare Laboratory Services	Minnesota Department of Health, Public Health Laboratory	Alexandra Lorentz, Jacob Garfin, Matt Plumb, and Xiong Wang
EPI_ISL_2537154, EPI_ISL_2537170, EPI_ISL_2537175, EPI_ISL_2537224, EPI_ISL_2537245, EPI_ISL_2537267	Gravity Diagnostics, LLC	Gravity Diagnostics, LLC	Gravity Diagnostics
EPI_ISL_2537367, EPI_ISL_2537371	Montana Public Health Laboratory	Montana Public Health Laboratory	Joy Ritter, Michelle Mozer, Carrie Biskupiak, Deborah Gibson, Michael Dills
EPI_ISL_2544621	IN State Department of Health Laboratory Services	IN State Department of Health Laboratory Services	Cassandra Campion, Jamie Yeadon, Brian Pope, Lixia Liu, Kyle Brownlee, Melissa Hindenlang, Mark Glazier
EPI_ISL_2544915, EPI_ISL_2545005, EPI_ISL_2545015, EPI_ISL_2545016, EPI_ISL_2545017, EPI_ISL_2545021, EPI_ISL_2545029, EPI_ISL_2545055, EPI_ISL_2545125, EPI_ISL_2545134, EPI_ISL_2545149			
see above	UW Virology Lab	UW Virology Lab	Pavitra Roychoudhury, Hong Xie, Lasata Shrestha, Shah Mohamed Bakhash, Tien V. Nguyen, Noah R. Baker, Sean Ellis, Meei-Li Huang, Keith R Jerome, Alexander Greninger
EPI_ISL_2547288, EPI_ISL_2547289	UHTL, University Hospitals	UHTL, University Hospitals	Sadri,N., Alouani,D., Song,X.
EPI_ISL_2549330, EPI_ISL_2549360, EPI_ISL_2549364, EPI_ISL_2549373, EPI_ISL_2549377, EPI_ISL_2549402, EPI_ISL_2549403, EPI_ISL_2549410, EPI_ISL_2549420, EPI_ISL_2549427, EPI_ISL_2549429, EPI_ISL_2549461, EPI_ISL_2549463, EPI_ISL_2549467, EPI_ISL_2549468, EPI_ISL_2549470, EPI_ISL_2549486, EPI_ISL_2549487, EPI_ISL_2549489, EPI_ISL_2549491, EPI_ISL_2549500, EPI_ISL_2549513, EPI_ISL_2549514			
see above	UW Virology Lab	UW Virology Lab	Pavitra Roychoudhury, Hong Xie, Lasata Shrestha, Tien V. Nguyen, Shah Mohamed

			Bakhash, Michelle Lin, Noah R. Baker, Ricardo Perez, Sean Ellis, Nathan Breit, Robert J. Livingston, Meei-Li Huang, Keith R Jerome, Patrick Mathias, Alexander Greninger
EPI_ISL_2550374, EPI_ISL_2550427	Colorado Department of Public Health and Environment	Colorado Department of Public Health and Environment	Laura Bankers, Molly C. Hetherington-Rauth, Diana Ir, Alexandria Rossheim, Shannon R. Matzinger, Sarah Elizabeth Totten, Emily A. Travanty
EPI_ISL_2550763	Maryland Public Health Laboratory (MD PHL)	Maryland Public Health Laboratory (MD PHL)	Maryland Department of Health Laboratories Administration
EPI_ISL_2557051	Washington State Department of Health Public Health Laboratories	Washington State Department of Health Public Health Laboratories	Drew MacKellar, Philip Dykema, Denny Russell, Joenice Gonzalez, Hannah Gray, Geoff Melly, Vanessa De Los Santos, Darren Lucas, JohnAric Peterson, Avi Singh, Rebecca Cao
EPI_ISL_2557124, EPI_ISL_2557128, EPI_ISL_2557146, EPI_ISL_2557147, EPI_ISL_2557151	NJDOH, Public Health and Environmental Laboratories	NJ_PHEL	Lindsey Bodnar, Shiv K. Verma, Ryan Pachucki, Chelsea San Filippo, Dana Woell, Allison Roder, Byeong Jeong
EPI_ISL_2557433, EPI_ISL_2557439, EPI_ISL_2557526, EPI_ISL_2557540, EPI_ISL_2557561, EPI_ISL_2557591, EPI_ISL_2557595	Infinity Biologix	Centers for Disease Control and Prevention Division of Viral Diseases, Pathogen Discovery	Dakota Howard, Dhwani Batra, Peter W. Cook, Kara Moser, Adrian Paskey, Jason Caravas, Benjamin Rambo-Martin, Shatavia Morrison, Christopher Gulvick, Scott Sammons, Yvette Unoarumhi, Darlene Wagner, Matthew Schmerer, Christian Bixby, Yihe Wang, Jonathan Schultz, Chirayu Goswami, Russ Hager, Robin Grimwood, Clinton R. Paden, Duncan MacCannell
EPI_ISL_2557929, EPI_ISL_2557934	Oregon State Public Health Laboratory	Oregon State Public Health Laboratory	Rafia Razzaque, Eugene Yeboah, Vanda Makris, Laura Tsaknaridis, John Fontana and Shane Sevey
EPI_ISL_2560433	Wyoming Public Health Laboratory	Wyoming Public Health Laboratory	Jim Mildenberger, Taylor Fearing, Channing Weber, Ashley Norberg, Chayse Rowley, Marley Goetz, Brian Dominguez, Elliot Thomasson, Sam Britz, Cari Sloma, Robert Petit, and Rob Christensen
EPI_ISL_2562121, EPI_ISL_2562122	The Jackson Laboratory	The Jackson Laboratory	Long J,Bergeron D, Renzette N, Adams M, Omerza G, Kelly K, Li L
EPI_ISL_2565775	TXDSHS	TXDSHS	Rashmi Tuladhar, Bonnie Oh, Jenny Zhang, Maliha Rahman, Mayela Pedrueza, Anita Pokharel, Karen Bobier, Lorraine Rodriguez, Myong Koag, Chun Wang, Rachel Lee, Grace Kubin
EPI_ISL_2565868	Minnesota Department of Health, Public Health Laboratory	Minnesota Department of Health, Public Health Laboratory	Alexandra Lorentz, Jacob Garfin, Matt Plumb, and Xiong Wang
EPI_ISL_2565915, EPI_ISL_2565957, EPI_ISL_2565964	North Dakota Department of Health, Public Health Laboratory	North Dakota Department of Health, Public Health Laboratory	Lisa Wingerter
EPI_ISL_2566090	Wyoming Public Health Laboratory	Wyoming Public Health Laboratory	Jim Mildenberger, Taylor Fearing, Channing Weber, Ashley Norberg, Chayse Rowley, Marley Goetz, Brian Dominguez, Elliot Thomasson, Sam Britz, Cari Sloma, Robert Petit, and Rob Christensen
EPI_ISL_2566122	Nevada State Public Health Laboratory	Nevada State Public Health Laboratory	Andrew Gorzalski, Mark Pandori
EPI_ISL_2597647, EPI_ISL_2597694, EPI_ISL_2597695, EPI_ISL_2597704	Hospital of the University of Pennsylvania Molecular Pathology Lab	Bushman Lab - University of Pennsylvania	John K. Everett, Kyle Rodino, Shantan Reddy, Aoife M. Roche, Young Hwang, Scott Sherrill-Mix, Samantha A. Whiteside, Jevon Graham-Wooten, Layla A. Khatib, Ayannah S. Fitzgerald, Arupa Ganguly, Mike Feldman, Brendan Kelly, Ronald G. Collman and Frederic Bushan
EPI_ISL_2597928	Nebraska Public Health Laboratory	NPHL COVID-19 Response Team	NPHL COVID-19 Response Team
EPI_ISL_2598016	Hackensack Medical Center	New York Genome Center	Michael Zody, Andre Corvelo, Dayna M. Oschwald, Samantha Fennessey, Tom Maniatis, Liang Chen, Jose Mediavilla, Marcus Cunningham, Kaelea Composto, Kar Chow, David Perlin, Barry Kreiswirth
EPI_ISL_2598076, EPI_ISL_2598078	MD PHL	MD PHL	Maryland Department of Health Laboratories Administration
EPI_ISL_2598468, EPI_ISL_2598482	Maryland Genomics, Institute for Genome Sciences, University of Maryland School of Medicine	Maryland Genomics, Institute for Genome Sciences, University of Maryland School of Medicine	Tallon, Luke J; Sadzewicz, Lisa D; Humphrys, Mike; Ott, Sandra; Roussey, Holly; Santana-Cruz, Ivette; Vavikolanu, Kranthi; Lim, Jonathan;Hazen, Tracy;Fraser, Claire M; Ravel, Jacques
EPI_ISL_2598735	Fulgent Genetics	Centers for Disease Control and Prevention Division of Viral Diseases, Pathogen Discovery	Dakota Howard, Dhwani Batra, Peter W. Cook, Kara Moser, Adrian Paskey, Jason Caravas, Benjamin Rambo-Martin, Shatavia Morrison, Christopher Gulvick, Scott Sammons, Yvette Unoarumhi, Darlene Wagner, Matthew Schmerer, Harry Gao, Mickey Li, John Gao, Joseph Fierro, Benafsh Sapra, Becky Tsai, Yan Meng, Doreen Ng, James Xie, Clinton R. Paden, Duncan MacCannell
EPI_ISL_2599642, EPI_ISL_2599673, EPI_ISL_2599683, EPI_ISL_2599695, EPI_ISL_2599700, EPI_ISL_2599747, EPI_ISL_2599749, EPI_ISL_2599750, EPI_ISL_2599782, EPI_ISL_2599835, EPI_ISL_2599848, EPI_ISL_2599883, EPI_ISL_2599916			
see above	Quest Diagnostics Incorporated	Centers for Disease Control and Prevention Division of Viral Diseases, Pathogen Discovery	Dakota Howard, Dhwani Batra, Peter W. Cook, Kara Moser, Adrian Paskey, Jason Caravas, Benjamin Rambo-Martin, Shatavia Morrison, Christopher Gulvick, Scott Sammons, Yvette Unoarumhi, Darlene Wagner, Matthew Schmerer, S. H. Rosenthal, A. Gerasimova, R. M. Kagan, B. Anderson, M. Hua, Y. Liu, L.E. Bernstein, K.E. Livingston, A. Perez, I. A. Shlyakhter, R. V. Rolando, R. Owen, P. Tanpaiboon, F. Lacbawan, Clinton R. Paden, Duncan MacCannell
EPI_ISL_2601037	Instituto de Medicina Tropical & Salud Global, Universidad Iberoamericana (UNIBE)	Instituto de Medicina Tropical & Salud Global, Universidad Iberoamericana (UNIBE)	Victor Virgilio Calderon, Alejandro Vallejo Degaudenzi & Robert Paulino-Ramirez
EPI_ISL_2603211, EPI_ISL_2603216	Virginia Division of Consolidated Laboratory Services	Virginia Division of Consolidated Laboratory Services	Virginia DCLS

EPI_ISL_2603317	Rhode Island State Health Laboratory	Rhode Island State Health Laboratory	Ewa King, Richard C. Huard, Kristin Carpenter-Azevedo
EPI_ISL_2603351, EPI_ISL_2603354	Virginia Division of Consolidated Laboratory Services	Virginia Division of Consolidated Laboratory Services	Virginia DCLS
EPI_ISL_2603805	Salud Digna	Instituto Nacional de Medicina Genomica	Hidalgo-Miranda A, Cedro-Tanda A, Mendoza-Vargas A, Reyes-Grajeda JP, Abraham Campos-Romero, Moreno-Camacho José Luis, Rodriguez-Gallegos Jorge, Luna-Ruiz Marco, Gonzalez-Barrera D, Rangel-DeLeon D, Munguia-Garza P, Ramirez-Vega O, Escobar-Arrazola, M, Herrera-Montalvo LA.
EPI_ISL_2604126, EPI_ISL_2604128, EPI_ISL_2604168	Arizona State University	Arizona State University	Peter T. Skidmore, LaRinda A. Holland, Matthew F. Smith, Rabia Maqsood, Nicholas J. Mellor, Joy M. Blain, Valerie Harris, Joshua LaBaer, Vel Murugan, Efrem S. Lim
EPI_ISL_2604199, EPI_ISL_2604200, EPI_ISL_2604201, EPI_ISL_2604203, EPI_ISL_2604206	Oregon State Public Health Laboratory	Oregon State Public Health Laboratory	Rafia Razzaque, Eugene Yeboah, Vanda Makris, Laura Tsaknaridis, John Fontana and Shane Sevey
EPI_ISL_2604227	Montana Public Health Laboratory	Montana Public Health Laboratory	Joy Ritter, Michelle Mozer, Carrie Biskupiak, Deborah Gibson, Michael Dills
EPI_ISL_2604868	Omics Sciences Laboratory	Omics Sciences Laboratory	Derly Andrade Molina, Gabriel Morey León, Darlyn Amaya, Rubén Armas González, Juan Carlos Fernández Cadena
EPI_ISL_2604873, EPI_ISL_2604874, EPI_ISL_2604879, EPI_ISL_2604880	INTERLAB	Omics Sciences Laboratory	Derly Andrade Molina, Gabriel Morey León, Darlyn Amaya, Rubén Armas González, Juan Carlos Fernández Cadena
EPI_ISL_2604881	Omics Sciences Laboratory	Omics Sciences Laboratory	Derly Andrade Molina, Gabriel Morey León, Darlyn Amaya, Rubén Armas González, Juan Carlos Fernández Cadena
EPI_ISL_2609623, EPI_ISL_2610127, EPI_ISL_2610178, EPI_ISL_2610186, EPI_ISL_2610683, EPI_ISL_2610692, EPI_ISL_2610712, EPI_ISL_2610724	Dutch COVID-19 response team	National Institute for Public Health and the Environment (RIVM)	Adam Meijer, Harry Vennema, Dirk Eggink, Jeroen Cremer, Sharon van den Brink, Bas van der Veer, AnneMarie van den Brandt, Lisa Wijsman, Kim Freriks, Ryanne Jaarsma, Euníce Then, Lynn Aarts, Sanne Bos, Melissa van Tuil, Linda van de Nes, Florian Zwagemaker, Dennis Schmitz, Annelies Kroneman, Karim Hajji, Chantal Reusken, on behalf of the national COVID-19 response team
EPI_ISL_2611126, EPI_ISL_2611127, EPI_ISL_2611128, EPI_ISL_2611156, EPI_ISL_2611157, EPI_ISL_2611158, EPI_ISL_2611159, EPI_ISL_2611171, EPI_ISL_2611173, EPI_ISL_2611180, EPI_ISL_2611182, EPI_ISL_2611215, EPI_ISL_2611217, EPI_ISL_2611235, EPI_ISL_2611295, EPI_ISL_2611297, EPI_ISL_2611300, EPI_ISL_2611302, EPI_ISL_2611306, EPI_ISL_2611308, EPI_ISL_2611318, EPI_ISL_2611319, EPI_ISL_2611358, EPI_ISL_2611360, EPI_ISL_2611394, EPI_ISL_2611432, EPI_ISL_2611438, EPI_ISL_2611439, EPI_ISL_2611440, EPI_ISL_2611445, EPI_ISL_2611454, EPI_ISL_2611456, EPI_ISL_2611457, EPI_ISL_2611460, EPI_ISL_2611467, EPI_ISL_2611468, EPI_ISL_2611491, EPI_ISL_2611492, EPI_ISL_2611523, EPI_ISL_2611530, EPI_ISL_2611531, EPI_ISL_2611537, EPI_ISL_2611541, EPI_ISL_2611542, EPI_ISL_2611543, EPI_ISL_2611545, EPI_ISL_2611547, EPI_ISL_2611566, EPI_ISL_2611588, EPI_ISL_2611591, EPI_ISL_2611599, EPI_ISL_2611618, EPI_ISL_2611621, EPI_ISL_2611636, EPI_ISL_2611650, EPI_ISL_2611675, EPI_ISL_2611686, EPI_ISL_2611695, EPI_ISL_2611696, EPI_ISL_2611698, EPI_ISL_2611716, EPI_ISL_2611720, EPI_ISL_2611728, EPI_ISL_2611757, EPI_ISL_2611774, EPI_ISL_2611775, EPI_ISL_2611783, EPI_ISL_2611800, EPI_ISL_2611806, EPI_ISL_2611814, EPI_ISL_2611815, EPI_ISL_2611820, EPI_ISL_2611821			
see above	Laboratory Corporation of America	Centers for Disease Control and Prevention Division of Viral Diseases, Pathogen Discovery	Dakota Howard, Dhwani Batra, Peter W. Cook, Kara Moser, Adrian Paskey, Jason Caravas, Benjamin Rambo-Martin, Shatavia Morrison, Christopher Gulvick, Scott Sammons, Yvette Unoarumhi, Darlene Wagner, Matthew Schmerer, Minoo Agarwal, Eyad Almasri, Debbie Boles, Ayla Burns, Nuthawin Charoensri, Oren Cohen, Susan Countryman, Mary Ann Cristobal, Bobbi Croy, Suzanne Dale, Hrushikesh Deshmukh, Amanda Douglas, Vincent Drouillon, Marcia Eisenberg, Howard Engler, Rama Ghatti, Prashant Gupta, Susan Hicks, Jake Humphrey, Lax Iyer, Lisa Pfefferle, Manoj Jain, Matthew Robinson, Mohan Kolli, Brian Krueger, Tim Kuphal, Stanley Letovsky, Michael Levandoski, Craig Lukasik, Jonathan Meltzer, Brian Norvell, Mindy Nye, Scott Parker, Christos Petropoulos, John Pruitt, Steven Ragan, Scott Ryan, Mike Sapeta, Jana Schroth, Suresh Babu Selvaraju, Goran Stevovic, Amanda Suchanek, Andrea Throop, Lyndon Tilson, Thomas Urban, Joe Voshell, Kimberly Wagner, Jonathan Williams, Mary Williamson, Qian Zeng, Tricia Zwiefelhofer, Clinton R. Paden, Duncan MacCannell
EPI_ISL_2611834, EPI_ISL_2611854, EPI_ISL_2611856	Quest Diagnostics Incorporated	Centers for Disease Control and Prevention Division of Viral Diseases, Pathogen Discovery	Dakota Howard, Dhwani Batra, Peter W. Cook, Kara Moser, Adrian Paskey, Jason Caravas, Benjamin Rambo-Martin, Shatavia Morrison, Christopher Gulvick, Scott Sammons, Yvette Unoarumhi, Darlene Wagner, Matthew Schmerer, S. H. Rosenthal, A. Gerasimova, R. M. Kagan, B. Anderson, M. Hua, Y. Liu, L.E. Bernstein, K.E. Livingston, A. Perez, I. A. Shlyakhter, R. V. Rolando, R. Owen, P. Tanpaiboon, F. Lacbawan, Clinton R. Paden, Duncan MacCannell
EPI_ISL_2612459	Montana Public Health Laboratory	Montana Public Health Laboratory	Joy Ritter, Michelle Mozer, Carrie Biskupiak, Deborah Gibson, Michael Dills
EPI_ISL_2612532	Centracare Laboratory Services	Minnesota Department of Health, Public Health Laboratory	Alexandra Lorentz, Jacob Garfin, Matt Plumb, and Xiong Wang
EPI_ISL_2612538, EPI_ISL_2612547	Oregon State Public Health Laboratory	Oregon State Public Health Laboratory	Rafia Razzaque, Eugene Yeboah, Vanda Makris, Laura Tsaknaridis, John Fontana and Shane Sevey
EPI_ISL_2614898	Nacionaline visuomenes sveikatos prieziuros laboratorija	National Public Health Surveillance Laboratory	Lukas Zemaitis, Migle Gabrielaite, Jelena Razmuk, Svajune Muralyte, Ana Steponkiene, Lukas Vasionis, Danas Baksa
EPI_ISL_2615045	Nacionalinis maisto ir veterinarijos rizikos vertinimo institutas	National Public Health Surveillance Laboratory	Lukas Zemaitis, Migle Gabrielaite, Jelena Razmuk, Svajune Muralyte, Ana Steponkiene, Lukas Vasionis, Danas Baksa
EPI_ISL_2615726	QLabs	WVU and Marshall University Combined Genomics Core Facilities	James Denvir, Peter Stoilov, Peter Perrotta, Wesley Kimble, Ryan Percifield
EPI_ISL_2615984	J.W. Ruby Memorial Hospital	WVU and Marshall University Combined	James Denvir, Peter Stoilov, Peter Perrotta, Wesley Kimble, Ryan Percifield

		Genomics Core Facilities	
EPI_ISL_2615985, EPI_ISL_2615986	Summersville Regional Medical Center	WVU and Marshall University Combined Genomics Core Facilities	James Denvir, Peter Stoilov, Peter Perrotta, Wesley Kimble, Ryan Percifield
EPI_ISL_2615987	QLabs	WVU and Marshall University Combined Genomics Core Facilities	James Denvir, Peter Stoilov, Peter Perrotta, Wesley Kimble, Ryan Percifield
EPI_ISL_2615992	Mon Health Medical Center	WVU and Marshall University Combined Genomics Core Facilities	James Denvir, Peter Stoilov, Peter Perrotta, Wesley Kimble, Ryan Percifield
EPI_ISL_2616041	Berkeley Medical Center	WVU and Marshall University Combined Genomics Core Facilities	James Denvir, Peter Stoilov, Peter Perrotta, Wesley Kimble, Ryan Percifield
EPI_ISL_2616042	QLabs	WVU and Marshall University Combined Genomics Core Facilities	James Denvir, Peter Stoilov, Peter Perrotta, Wesley Kimble, Ryan Percifield
EPI_ISL_2616057, EPI_ISL_2616058	Berkeley Medical Center	WVU and Marshall University Combined Genomics Core Facilities	James Denvir, Peter Stoilov, Peter Perrotta, Wesley Kimble, Ryan Percifield
EPI_ISL_2616062	Mon Health Medical Center	WVU and Marshall University Combined Genomics Core Facilities	James Denvir, Peter Stoilov, Peter Perrotta, Wesley Kimble, Ryan Percifield
EPI_ISL_2616063, EPI_ISL_2616064, EPI_ISL_2616065, EPI_ISL_2616066, EPI_ISL_2616067	QLabs	WVU and Marshall University Combined Genomics Core Facilities	James Denvir, Peter Stoilov, Peter Perrotta, Wesley Kimble, Ryan Percifield
EPI_ISL_2616074, EPI_ISL_2616075, EPI_ISL_2616076	J.W. Ruby Memorial Hospital	WVU and Marshall University Combined Genomics Core Facilities	James Denvir, Peter Stoilov, Peter Perrotta, Wesley Kimble, Ryan Percifield
EPI_ISL_2616094	Berkeley Medical Center	WVU and Marshall University Combined Genomics Core Facilities	James Denvir, Peter Stoilov, Peter Perrotta, Wesley Kimble, Ryan Percifield
EPI_ISL_2616095	J.W. Ruby Memorial Hospital	WVU and Marshall University Combined Genomics Core Facilities	James Denvir, Peter Stoilov, Peter Perrotta, Wesley Kimble, Ryan Percifield
EPI_ISL_2616098	QLabs	WVU and Marshall University Combined Genomics Core Facilities	James Denvir, Peter Stoilov, Peter Perrotta, Wesley Kimble, Ryan Percifield
EPI_ISL_2616103	Berkeley Medical Center	WVU and Marshall University Combined Genomics Core Facilities	James Denvir, Peter Stoilov, Peter Perrotta, Wesley Kimble, Ryan Percifield
EPI_ISL_2616104	Hampshire Memorial Hospital	WVU and Marshall University Combined Genomics Core Facilities	James Denvir, Peter Stoilov, Peter Perrotta, Wesley Kimble, Ryan Percifield
EPI_ISL_2616105, EPI_ISL_2616106	QLabs	WVU and Marshall University Combined Genomics Core Facilities	James Denvir, Peter Stoilov, Peter Perrotta, Wesley Kimble, Ryan Percifield
EPI_ISL_2616107	Berkeley Medical Center	WVU and Marshall University Combined Genomics Core Facilities	James Denvir, Peter Stoilov, Peter Perrotta, Wesley Kimble, Ryan Percifield
EPI_ISL_2617018, EPI_ISL_2617019	Salud Digna	Instituto Nacional de Medicina Genomica	Hidalgo-Miranda A, Cedro-Tanda A, Mendoza-Vargas A, Reyes-Grajeda JP, Abraham Campos-Romero, Moreno-Camacho José Luis, Rodriguez-Gallegos Jorge, Luna-Ruiz Marco, Gonzalez-Barrera D, Rangel-DeLeon D, Munguia-Garza P, Ramirez-Vega O, Escobar-Arrazola, M, Herrera-Montalvo LA.
EPI_ISL_2617629, EPI_ISL_2617630, EPI_ISL_2617980, EPI_ISL_2617983, EPI_ISL_2618054, EPI_ISL_2618073, EPI_ISL_2618080	UW Virology Lab	UW Virology Lab	Pavitra Roychoudhury, Hong Xie, Lasata Shrestha, Tien V. Nguyen, Shah Mohamed Bakhash, Michelle Lin, Noah R. Baker, Ricardo Perez, Sean Ellis, Nathan Breit, Robert J. Livingston, Meei-Li Huang, Keith R Jerome, Patrick Mathias, Alexander Greninger
EPI_ISL_2626098	HOSPITAL UNIVERSITARIO SON ESPASES	HOSPITAL UNIVERSITARIO SON ESPASES	Servicio de Microbiología, Hospital Universitario Son Espases
EPI_ISL_2626128	HOSPITAL MANACOR	HOSPITAL UNIVERSITARIO SON ESPASES	Servicio de Microbiología, Hospital Universitario Son Espases
EPI_ISL_2626131	HOSPITAL UNIVERSITARIO SON ESPASES	HOSPITAL UNIVERSITARIO SON ESPASES	Servicio de Microbiología, Hospital Universitario Son Espases
EPI_ISL_2626147, EPI_ISL_2626155	HOSPITAL MANACOR	HOSPITAL UNIVERSITARIO SON ESPASES	Servicio de Microbiología, Hospital Universitario Son Espases
EPI_ISL_2626165	HOSPITAL CAN MISSES	HOSPITAL UNIVERSITARIO SON ESPASES	Servicio de Microbiología, Hospital Universitario Son Espases
EPI_ISL_2626168	HOSPITAL MANACOR	HOSPITAL UNIVERSITARIO SON ESPASES	Servicio de Microbiología, Hospital Universitario Son Espases
EPI_ISL_2626704,	Alaska State Virology	Alaska State Virology	Stephanie DeRonde, Elva House, Jacob Zidek, Lisa Smith, Ph.D., Jack Chen, Ph.D.

EPI_ISL_2626742, EPI_ISL_2626747	Laboratory	Laboratory	
EPI_ISL_2626784, EPI_ISL_2626796	Baylor Scott & White-Temple	Baylor Scott & White-Temple	Ari Rao, Linden Morales, Kimberly Walker, Marcus Volz, Shelby Johnson
EPI_ISL_2626932, EPI_ISL_2626937, EPI_ISL_2626939, EPI_ISL_2626940, EPI_ISL_2626953, EPI_ISL_2626958, EPI_ISL_2626971	TEMPUS LABS INC	Wadsworth Center, New York State Department of Health	Kirsten St. George, Daryl M. Lamson, Alexis Russell, Matthew Shudt, Melissa A Leisner, Jonathan Plitnick, Catharine Prussing, Navjot Singh, John Kelly, Erasmus Schneider, Erica Lasek-Nesselquist
EPI_ISL_2626987	URMC LABS	Wadsworth Center, New York State Department of Health	Kirsten St. George, Daryl M. Lamson, Alexis Russell, Matthew Shudt, Melissa A Leisner, Jonathan Plitnick, Catharine Prussing, Navjot Singh, John Kelly, Erasmus Schneider, Erica Lasek-Nesselquist
EPI_ISL_2627000, EPI_ISL_2627002	ALBANY MEDICAL CENTER	Wadsworth Center, New York State Department of Health	Kirsten St. George, Daryl M. Lamson, Alexis Russell, Matthew Shudt, Melissa A Leisner, Jonathan Plitnick, Catharine Prussing, Navjot Singh, John Kelly, Erasmus Schneider, Erica Lasek-Nesselquist
EPI_ISL_2627144, EPI_ISL_2627145	MONTEFIORE MEDICAL CENTER LABORATORIES	Wadsworth Center, New York State Department of Health	Kirsten St. George, Daryl M. Lamson, Alexis Russell, Matthew Shudt, Melissa A Leisner, Jonathan Plitnick, Catharine Prussing, Navjot Singh, John Kelly, Erasmus Schneider, Erica Lasek-Nesselquist
EPI_ISL_2627770	NCSLPH	NCSLPH	Chase K, Miller MC, Greene S, Glover W
EPI_ISL_2629191, EPI_ISL_2629195, EPI_ISL_2629206, EPI_ISL_2629209, EPI_ISL_2629216	Microbiology Division, South Carolina Department of Health and Environmental Control	Microbiology Division, South Carolina Department of Health and Environmental Control	Freeman,J.S., Flores,H.V.
EPI_ISL_2631368, EPI_ISL_2631373, EPI_ISL_2631377	Pandemic Response Lab - NYC	Pandemic Response Lab, R&D	Henry Lee, Michael Hammerling, Melissa Hopkins, Cybill del Castillo, Shinyoung Clair Kang, William Ward, Pradeep Bugga, Sol Rey, Dylan Law, Katharine Nelson, Haiping Hao, Jon Laurent
EPI_ISL_2631590, EPI_ISL_2631596	SYNLAB MVZ Trier	Robert Koch Institute	unknown
EPI_ISL_2633584	CENTOGENE Frankfurt Laboratory: Niederlassung Industriepark Höchst	Robert Koch Institute	unknown
EPI_ISL_2634588	Eurofins LifeCodexx GmbH	Robert Koch Institute	unknown
EPI_ISL_2634936	Pandemic Response Lab - NYC	Pandemic Response Lab, R&D	Henry Lee, Michael Hammerling, Melissa Hopkins, Cybill del Castillo, Shinyoung Clair Kang, William Ward, Pradeep Bugga, Sol Rey, Dylan Law, Katharine Nelson, Haiping Hao, Jon Laurent
EPI_ISL_2645310, EPI_ISL_2645313, EPI_ISL_2645315	SC Dept of Health and Env. Control-Bureau of Laboratories	Centers for Disease Control and Prevention Division of Viral Diseases, Pathogen Discovery	Mili Sheth, Sarah Nobles, Jasmine Padilla, Mark Burroughs, Shoshona Le, Katie Dillon, Peter Cook, Clinton R. Paden, Dhwani Batra, Krista Queen, Kristen Knipe, Dakota Howard, Yvette Unoarumhi, Darlene Wagner, Matthew Schmerer, Ben L. Rambo-Martin, Kristine Lacek, Sam Shepard, Alison Laufer Halpin, Dave Wentworth, Vivien Dugan, Suxiang Tong, Justin Lee
EPI_ISL_2645351	RI State Health Laboratories	Centers for Disease Control and Prevention Division of Viral Diseases, Pathogen Discovery	Mili Sheth, Sarah Nobles, Jasmine Padilla, Mark Burroughs, Shoshona Le, Katie Dillon, Peter Cook, Clinton R. Paden, Dhwani Batra, Krista Queen, Kristen Knipe, Dakota Howard, Yvette Unoarumhi, Darlene Wagner, Matthew Schmerer, Ben L. Rambo-Martin, Kristine Lacek, Sam Shepard, Alison Laufer Halpin, Dave Wentworth, Vivien Dugan, Suxiang Tong, Justin Lee
EPI_ISL_2645373	NH Dept. of Health and Human Services Public Health Labs	Centers for Disease Control and Prevention Division of Viral Diseases, Pathogen Discovery	Mili Sheth, Sarah Nobles, Jasmine Padilla, Mark Burroughs, Shoshona Le, Katie Dillon, Peter Cook, Clinton R. Paden, Dhwani Batra, Krista Queen, Kristen Knipe, Dakota Howard, Yvette Unoarumhi, Darlene Wagner, Matthew Schmerer, Ben L. Rambo-Martin, Kristine Lacek, Sam Shepard, Alison Laufer Halpin, Dave Wentworth, Vivien Dugan, Suxiang Tong, Justin Lee
EPI_ISL_2647397, EPI_ISL_2647462, EPI_ISL_2647490, EPI_ISL_2647497, EPI_ISL_2647514, EPI_ISL_2647531, EPI_ISL_2647591, EPI_ISL_2647597, EPI_ISL_2647606, EPI_ISL_2647632, EPI_ISL_2647640, EPI_ISL_2647660, EPI_ISL_2647677, EPI_ISL_2647697, EPI_ISL_2647703, EPI_ISL_2647719, EPI_ISL_2647808, EPI_ISL_2647817, EPI_ISL_2647829, EPI_ISL_2647892, EPI_ISL_2647893, EPI_ISL_2647895, EPI_ISL_2647908, EPI_ISL_2647955, EPI_ISL_2648005			
see above	Mako Medical	Centers for Disease Control and Prevention Division of Viral Diseases, Pathogen Discovery	Dakota Howard, Dhwani Batra, Peter W. Cook, Kara Moser, Adrian Paskey, Jason Caravas, Benjamin Rambo-Martin, Shatavia Morrison, Christopher Gulvick, Scott Sammons, Yvette Unoarumhi, Darlene Wagner, Matthew Schmerer,Matthew Tugwell, Lauren Moon, Clinton R. Paden, Duncan MacCannell
EPI_ISL_2649850, EPI_ISL_2649875, EPI_ISL_2649883, EPI_ISL_2649884	NJDOH, Public Health and Environmental Laboratories	NJ_PHEL	Lindsey Bodnar, Shiv K. Verma, Ryan Pachucki, Chelsea San Filippo, Jacquelyn Deverell, Dana Woell, Allison Roder, Byeong Jeong
EPI_ISL_2650248	Microbiologia CATLAB	Can Ruti SARS-CoV-2 Sequencing Hub (HUGTiP/IrsiCaixa/IGTP)	Marc Noguera-Julian, Pilar Armengol, Ignacio Blanco, Antoni E Bordoy, Francesc Catala-Moll, Pere-Joan Cardona, Maria Casadellà, Cristina Casañ, Gemma Clara, Bonaventura Clotet, Cristina Esteban, Montserrat Giménez, Mercedes Guerrero, Anna Not, Roger Paredes, Mariona Parera, Verónica Saludes, Alba Sánchez, and Elisa Martró on behalf of the Can Ruti SARS-CoV-2 Sequencing Hub
EPI_ISL_2650521	University of Oregon COVID-19 MAP Laboratory	University of Oregon Genomics and Cell Charactericzation Core Facility (GC3F)	Douglas Turnbull, Ariana White, Jeff Bishop, Jason Carriere, Jason Sydes, Peter Batzel
EPI_ISL_2651044	Washington State Department of Health Public Health Laboratories	Washington State Department of Health Public Health Laboratories	Drew MacKellar, Philip Dykema, Denny Russell, Joenice Gonzalez, Hannah Gray, Geoff Melly, Vanessa De Los Santos, Darren Lucas, JohnAric Peterson, Avi Singh, Rebecca Cao
EPI_ISL_2652041	New Mexico Department	New Mexico Department	Ellie Johnson, D'eldra Malone, Jennifer Benoit, Ratheesh Rajan, Linda Salazar,

	of Health Scientific Laboratory	of Health Scientific Laboratory	Keila Gutierrez, Anastacia Griego-Fisher
EPI_ISL_2652270, EPI_ISL_2652296, EPI_ISL_2652320, EPI_ISL_2652329, EPI_ISL_2652330, EPI_ISL_2652355, EPI_ISL_2652370, EPI_ISL_2652385, EPI_ISL_2652418, EPI_ISL_2652420, EPI_ISL_2652426, EPI_ISL_2652430, EPI_ISL_2652471, EPI_ISL_2652475			
see above	Quest Diagnostics Incorporated	Centers for Disease Control and Prevention Division of Viral Diseases, Pathogen Discovery	Dakota Howard, Dhwani Batra, Peter W. Cook, Kara Moser, Adrian Paskey, Jason Caravas, Benjamin Rambo-Martin, Shatavia Morrison, Christopher Gulvick, Scott Sammons, Yvette Unoarumhi, Darlene Wagner, Matthew Schmerer, S. H. Rosenthal, A. Gerasimova, R. M. Kagan, B. Anderson, M. Hua, Y. Liu, L.E. Bernstein, K.E. Livingston, A. Perez, I. A. Shlyakhter, R. V. Rolando, R. Owen, P. Tanpaiboon, F. Lacbawan, Clinton R. Paden, Duncan MacCannell
EPI_ISL_2652716, EPI_ISL_2652959, EPI_ISL_2652975, EPI_ISL_2653043, EPI_ISL_2653053, EPI_ISL_2653066	Infinity Biologix	Centers for Disease Control and Prevention Division of Viral Diseases, Pathogen Discovery	Dakota Howard, Dhwani Batra, Peter W. Cook, Kara Moser, Adrian Paskey, Jason Caravas, Benjamin Rambo-Martin, Shatavia Morrison, Christopher Gulvick, Scott Sammons, Yvette Unoarumhi, Darlene Wagner, Matthew Schmerer, Christian Bixby, Yihe Wang, Jonathan Schultz, Chirayu Goswami, Russ Hager, Robin Grimwood, Clinton R. Paden, Duncan MacCannell
EPI_ISL_2657665, EPI_ISL_2657666	NCSLPH	NCSLPH	Chase K, Miller MC, Greene S, Glover W
EPI_ISL_2658561, EPI_ISL_2658563	Minnesota Department of Health, Public Health Laboratory	Minnesota Department of Health, Public Health Laboratory	Alexandra Lorentz, Jacob Garfin, Matt Plumb, and Xiong Wang
EPI_ISL_2660408	Infectious Disease Program, Broad Institute of Harvard and MIT	Infectious Disease Program, Broad Institute of Harvard and MIT	Lemieux,J.E., Siddle,K.J., Shaw,B., Adams,G., Pierce,V., Turbett,S., Anahtar,M., Branda,J., Slater,D., Harris,J., Lin,A.E., Gladden-Young,A., Lagerborg,K., Rudy,M., DeRuff,K., Carter,A., Normandin,E., Bauer,M., Reilly,S., Tomkins-Tinch,C., Loreth,C., Chaluvadi,S., Neumann,A., Cusick,C., Chapman,S.B., Gnirke,A., Flowers,K., Cerrato,F., Birren,B.W., Gallagher,G., Smole,S., Park,D.J., MacInnis,B.L., Ryan,E., LaRocque,R., Rosenberg,E., Sabeti,P.C.
EPI_ISL_2661536, EPI_ISL_2661537	UHTL, University Hospitals	UHTL, University Hospitals	Sadri,N., Alouani,D., Song,X.
EPI_ISL_2662751, EPI_ISL_2662767, EPI_ISL_2662770, EPI_ISL_2662778, EPI_ISL_2662814	DPHL	Delaware Public Health Lab	Rebecca Savage
EPI_ISL_2663233	Mayo Clinic & Mayo Clinic Laboratories	Minnesota Department of Health, Public Health Laboratory	Alexandra Lorentz, Jacob Garfin, Matt Plumb, and Xiong Wang
EPI_ISL_2663500, EPI_ISL_2663503	Virginia Division of Consolidated Laboratory Services	Virginia Division of Consolidated Laboratory Services	Virginia Division of Consolidated Laboratory Services
EPI_ISL_2663516, EPI_ISL_2663517, EPI_ISL_2663534, EPI_ISL_2663537, EPI_ISL_2663540, EPI_ISL_2663541, EPI_ISL_2663542, EPI_ISL_2663543, EPI_ISL_2663550	NYU Langone Health	Departments of Pathology and Medicine, New York University School of Medicine	Adriana Heguy, Dacia Dimartino, Emily Guzman, Christian Marier, Peter Meyn, Sitharam Ramaswami, Gael Westby, Paul Zappile, Yutong Zhang, Paolo Cotzia, Guiqing Wang
EPI_ISL_2665592	Department of Bacteria, Parasites and Fungi, Statens Serum Institut, Copenhagen, Denmark	Statens Serum Institut Bioinformatics and Microbial Genomics	Danish Covid-19 Genome Consortium
EPI_ISL_2671584	Centro de Investigacion Biomedica de Occidente (CIBO)	Unidad de Genomica Avanzada	Consorcio Mexicano de Vigilancia Genomica (CoViGen-Mex). Authors (in alphabetical order): Julio Elias Alvarado-Yaah, Carlos F. Arias, Santiago avila-Rios, Eduardo Becerril-Vargas, Victor Hugo Borja-Aburto, Celia Boukadida, Cristobal Chaidez-Quiroz, Juan Bautista Chale-Dzul, Ricardo Ciria Merce, Andreu Comas-Garcia, Celida Duque Molina, Julissa Enciso-Ibarra, Jose Antonio Enciso-Moreno, Gloria Elena Espinosa-Ayala, Fernando Fontove-Herrera, Daniel Fregoso-Rueda, Victor Eduardo Garcia-Arias, Alejandra Garcia-Gasca, Bruno Gomez-Gil, Jean Pierre Gonzalez, Irvin Gonzalez-Lopez, Concepcion Grajales-Muñiz, Ricardo Grande, Rosa Maria Gutierrez Rios, Jesus Hernandez, Alejandra Hernandez-Teran, Alfredo Herrera-Estrella, Carla Ivon Herrera-Najera, Pavel Isa, Veronica Mata-Haro, Daniel Lira Morales, Susana Lopez, Antonio Loza Roman, Brenda Irasema Maldonado-Meza, Bernardo Martinez-Miguel, Jose Arturo Martinez-Orozco, Celida Martinez- Rodriguez, Margarita Matias-Florentino, Fidencio Mejia-Nepomuceno, Maria Guadalupe de Jesus Mireles-Rivera, Gloria Maria Molina-Salinas, Hector Montoya-Fuentes, , Mario Mujica-Sanchez, Jose Esteban Muñoz-Medina, Jose de Jesus Nuñez-Contreras, Alicia Ocaña-Mondragon, Luis Alberto Ochoa-Carrera, Hector Esteban Paz-Juarez, Marissa Perez-Garcia, Francisco Pulido, Helen Haydee Fernanda Ramirez-Plascencia, Jorge Salas-Hernandez, Angel Gustavo Salas-Lais, Alejandro Sanchez-Flores, Clara Esperanza Santacruz-Tinoco, Maria Guadalupe Santiago-Mauricio, Selene Zarate, Nelly Selem-Mojica, Blanca Taboada, Gloria Vazquez, Joel Armando Vazquez-Perez.
EPI_ISL_2673234, EPI_ISL_2673252, EPI_ISL_2673262, EPI_ISL_2673274	Dutch COVID-19 response team	National Institute for Public Health and the Environment (RIVM)	Adam Meijer, Harry Vennema, Dirk Eggink, Jeroen Cremer, Sharon van den Brink, Bas van der Veer, AnneMarie van den Brandt, Lisa Wijsman, Kim Freriks, Ryanne Jaarsma, Euníce Then, Lynn Aarts, Sanne Bos, Melissa van Tuil, Linda van de Nes, Florian Zwagemaker, Dennis Schmitz, Annelies Kroneman, Karim Hajji, Chantal Reusken, on behalf of the national COVID-19 response team
EPI_ISL_2678987, EPI_ISL_2678989, EPI_ISL_2679303	Utah Public Health Laboratory	Utah Public Health Laboratory	Erin L. Young, Kelly F. Oakeson, Tara Gallagher

EPI_ISL_2680476	Humboldt County Public Health Laboratory	Humboldt County Public Health Laboratory	Jeremy Corrigan
EPI_ISL_2681110	Centro de Investigación Biomédica del Noreste (CIBIN)	Instituto de Biotecnología de la UNAM	Consorcio Mexicano de Vigilancia Genómica (CoViGen-Mex). Authors (in alphabetical order): Julio Elias Alvarado-Yaah, Carlos F. Arias, Santiago Ávila-Ríos, Eduardo Becerril-Vargas, Víctor Hugo Borja-Aburto, Celia Boukadida, Cristóbal Cháidez-Quiróz, Juan Bautista Chale-Dzul, Ricardo Ciria Merce, Andreu Comas-García, Célida Duque Molina, Julissa Enciso-Ibarra, José Antonio Enciso-Moreno, Gloria Elena Espinosa-Ayala, Fernando Fontove-Herrera, Daniel Fregoso-Rueda, Victor Eduardo García-Arias, Alejandra García-Gasca, Bruno Gómez-Gil, Jean Pierre González, Irvin González-López, Concepción Grajales-Muñiz, Ricardo Grande, Rosa María Gutiérrez Rios, Jesús Hernández, Alejandra Hernández-Terán, Alfredo Herrera-Estrella, Carla Ivón Herrera-Najera, Pavel Isa, Verónica Mata-Haro, Daniel Lira Morales, Susana Lopez, Antonio Loza Román, Brenda Irasema Maldonado-Meza, Bernardo Martínez-Miguel, José Arturo Martínez-Orozco, Célida Martínez- Rodríguez, Margarita Matías-Florentino, Fidencio Mejía-Nepomuceno, María Guadalupe de Jesús Mireles-Rivera, Gloria María Molina-Salinas, Hector Montoya-Fuentes, , Mario Mújica-Sánchez, José Esteban Muñoz-Medina, José de Jesús Nuñez-Contreras, Alicia Ocaña-Mondragón, Luis Alberto Ochoa-Carrera, Hector Esteban Paz-Juárez, Marissa Perez-Garcia, Francisco Pulido, Helen Haydee Fernanda Ramírez-Plascencia, Jorge Salas-Hernández, Angel Gustavo Salas-Lais, Alejandro Sánchez-Flores, Clara Esperanza Santacruz-Tinoco, María Guadalupe Santiago-Mauricio, Selene Zárate, Nelly Sélem-Mojica, Blanca Taboada, Gloria Vazquez, Joel Armando Vázquez-Pérez.
EPI_ISL_2681221	Unidad de Investigación Biomédica de Zacatecas (UIBZ)	Instituto de Biotecnología de la UNAM	Consorcio Mexicano de Vigilancia Genómica (CoViGen-Mex). Authors (in alphabetical order): Julio Elias Alvarado-Yaah, Carlos F. Arias, Santiago Ávila-Ríos, Eduardo Becerril-Vargas, Víctor Hugo Borja-Aburto, Celia Boukadida, Cristóbal Cháidez-Quiróz, Juan Bautista Chale-Dzul, Ricardo Ciria Merce, Andreu Comas-García, Célida Duque Molina, Julissa Enciso-Ibarra, José Antonio Enciso-Moreno, Gloria Elena Espinosa-Ayala, Fernando Fontove-Herrera, Daniel Fregoso-Rueda, Victor Eduardo García-Arias, Alejandra García-Gasca, Bruno Gómez-Gil, Jean Pierre González, Irvin González-López, Concepción Grajales-Muñiz, Ricardo Grande, Rosa María Gutiérrez Rios, Jesús Hernández, Alejandra Hernández-Terán, Alfredo Herrera-Estrella, Carla Ivón Herrera-Najera, Pavel Isa, Verónica Mata-Haro, Daniel Lira Morales, Susana Lopez, Antonio Loza Román, Brenda Irasema Maldonado-Meza, Bernardo Martínez-Miguel, José Arturo Martínez-Orozco, Célida Martínez- Rodríguez, Margarita Matías-Florentino, Fidencio Mejía-Nepomuceno, María Guadalupe de Jesús Mireles-Rivera, Gloria María Molina-Salinas, Hector Montoya-Fuentes, , Mario Mújica-Sánchez, José Esteban Muñoz-Medina, José de Jesús Nuñez-Contreras, Alicia Ocaña-Mondragón, Luis Alberto Ochoa-Carrera, Hector Esteban Paz-Juárez, Marissa Perez-Garcia, Francisco Pulido, Helen Haydee Fernanda Ramírez-Plascencia, Jorge Salas-Hernández, Angel Gustavo Salas-Lais, Alejandro Sánchez-Flores, Clara Esperanza Santacruz-Tinoco, María Guadalupe Santiago-Mauricio, Selene Zárate, Nelly Sélem-Mojica, Blanca Taboada, Gloria Vazquez, Joel Armando Vázquez-Pérez.
EPI_ISL_2686412, EPI_ISL_2686414, EPI_ISL_2686455, EPI_ISL_2686506, EPI_ISL_2686603, EPI_ISL_2686634, EPI_ISL_2686677, EPI_ISL_2686700, EPI_ISL_2686701, EPI_ISL_2686703, EPI_ISL_2686748			
see above	Fulgent Genetics	Centers for Disease Control and Prevention Division of Viral Diseases, Pathogen Discovery	Dakota Howard, Dhwani Batra, Peter W. Cook, Kara Moser, Adrian Paskey, Jason Caravas, Benjamin Rambo-Martin, Shatavia Morrison, Christopher Gulvick, Scott Sammons, Yvette Unoarumhi, Darlene Wagner, Matthew Schmerer, Harry Gao, Mickey Li, John Gao, Joseph Fierro, Benafsh Sapra, Becky Tsai, Yan Meng, Doreen Ng, James Xie, Clinton R. Paden, Duncan MacCannell
EPI_ISL_2686842, EPI_ISL_2686858, EPI_ISL_2686876	Infinity Biologix	Centers for Disease Control and Prevention Division of Viral Diseases, Pathogen Discovery	Dakota Howard, Dhwani Batra, Peter W. Cook, Kara Moser, Adrian Paskey, Jason Caravas, Benjamin Rambo-Martin, Shatavia Morrison, Christopher Gulvick, Scott Sammons, Yvette Unoarumhi, Darlene Wagner, Matthew Schmerer, Christian Bixby, Yihe Wang, Jonathan Schultz, Chirayu Goswami, Russ Hager, Robin Grimwood, Clinton R. Paden, Duncan MacCannell
EPI_ISL_2686915, EPI_ISL_2686931, EPI_ISL_2686945, EPI_ISL_2686951, EPI_ISL_2686957, EPI_ISL_2687007, EPI_ISL_2687008, EPI_ISL_2687016, EPI_ISL_2687017, EPI_ISL_2687064, EPI_ISL_2687071, EPI_ISL_2687072, EPI_ISL_2687079, EPI_ISL_2687082, EPI_ISL_2687116, EPI_ISL_2687122, EPI_ISL_2687140, EPI_ISL_2687173, EPI_ISL_2687216, EPI_ISL_2687271, EPI_ISL_2687384, EPI_ISL_2687430, EPI_ISL_2687441, EPI_ISL_2687466, EPI_ISL_2687484, EPI_ISL_2687519, EPI_ISL_2687552			
see above	Aegis Sciences Corporation	Centers for Disease Control and Prevention Division of Viral Diseases, Pathogen Discovery	Dakota Howard, Dhwani Batra, Peter W. Cook, Kara Moser, Adrian Paskey, Jason Caravas, Benjamin Rambo-Martin, Shatavia Morrison, Christopher Gulvick, Scott Sammons, Yvette Unoarumhi, Darlene Wagner, Matthew Schmerer, Cyndi Clark, Patrick Campbell, Rob Case, Vikramsinha Ghorpade, Holly Houdeshell, Ola Kvalvaag, Dillon Nall, Ethan Sanders, Alec Vest, Shaun Westlund, Matthew Hardison, Clinton R. Paden, Duncan MacCannell
EPI_ISL_2688317	Puerto Rico Department of Health	Centers for Disease Control and Prevention, Dengue Branch	Gilberto A. Santiago, Glenda Gonzalez, Betzabel Flores, Keyla Charriez, Gabriela Paz-Bailey, Jorge L. Munoz-Jordan
EPI_ISL_2688999	DC Public Health Lab/ Dept. of Forensic Sciences	DC Public Health Lab/ Dept. of Forensic Sciences	Janis Doss, Scott Nguyen, Elizabeth Zelaya, Sarah Scott, Connie Maza, Monica Mann, Brittany Hamilton, David Payne, Jocelyn Hauser
EPI_ISL_2689031, EPI_ISL_2689032, EPI_ISL_2689037	Mayo Clinic & Mayo Clinic Laboratories	Minnesota Department of Health, Public Health Laboratory	Alexandra Lorentz, Jacob Garfin, Matt Plumb, and Xiong Wang
EPI_ISL_2689057, EPI_ISL_2689058, EPI_ISL_2689102	MD PHL	MD PHL	Maryland Department of Health Laboratories Administration
EPI_ISL_2689601	GA Department of Public Health Laboratory	Genomics and Discovery, Respiratory Viruses Branch, Division of Viral Diseases,	Jing Zhang, Anna Kelleher, Ying Tao, Yan Li, Brian Lynch, Krista Queen, Anna Uehara, Peter Cook, Han Jia Justin Ng, Rachel Marine, Clinton R. Paden, Dhwani Batra, Haibin Wang, Tara Coalter, Jasmine Padilla, Morgan Davis, Mili Sheth, Sarah Nobles, Mark Burroughs, Justin Lee, Adam Retchless, Suxiang Tong

		Centers for Disease Control and Prevention	
EPI_ISL_2689603	OK Public Health Laboratory, Oklahoma State DOH	Genomics and Discovery, Respiratory Viruses Branch, Division of Viral Diseases, Centers for Disease Control and Prevention	Jing Zhang, Anna Kelleher, Ying Tao, Yan Li, Brian Lynch, Krista Queen, Anna Uehara, Peter Cook, Han Jia Justin Ng, Rachel Marine, Clinton R. Paden, Dhwani Batra, Haibin Wang, Tara Coalter, Jasmine Padilla, Morgan Davis, Mili Sheth, Sarah Nobles, Mark Burroughs, Justin Lee, Adam Retchless, Suxiang Tong
EPI_ISL_2689848, EPI_ISL_2689856, EPI_ISL_2689863	Virginia Division of Consolidated Laboratory Services	Virginia Division of Consolidated Laboratory Services	Virginia Division of Consolidated Laboratory Services
EPI_ISL_2689910, EPI_ISL_2689911	RI State Health Laboratories	Centers for Disease Control and Prevention Division of Viral Diseases, Pathogen Discovery	Mili Sheth, Sarah Nobles, Jasmine Padilla, Mark Burroughs, Shoshona Le, Katie Dillon, Peter Cook, Clinton R. Paden, Dhwani Batra, Krista Queen, Kristen Knipe, Dakota Howard, Yvette Unoarumhi, Darlene Wagner, Matthew Schmerer, Ben L. Rambo-Martin, Kristine Lacek, Sam Shepard, Alison Laufer Halpin, Dave Wentworth, Vivien Dugan, Suxiang Tong, Justin Lee
EPI_ISL_2689961	LA Office of Public Health Laboratories	Centers for Disease Control and Prevention Division of Viral Diseases, Pathogen Discovery	Mili Sheth, Sarah Nobles, Jasmine Padilla, Mark Burroughs, Shoshona Le, Katie Dillon, Peter Cook, Clinton R. Paden, Dhwani Batra, Krista Queen, Kristen Knipe, Dakota Howard, Yvette Unoarumhi, Darlene Wagner, Matthew Schmerer, Ben L. Rambo-Martin, Kristine Lacek, Sam Shepard, Alison Laufer Halpin, Dave Wentworth, Vivien Dugan, Suxiang Tong, Justin Lee
EPI_ISL_2689980	OR State PHL-Virology/Immunology Section	Centers for Disease Control and Prevention Division of Viral Diseases, Pathogen Discovery	Mili Sheth, Sarah Nobles, Jasmine Padilla, Mark Burroughs, Shoshona Le, Katie Dillon, Peter Cook, Clinton R. Paden, Dhwani Batra, Krista Queen, Kristen Knipe, Dakota Howard, Yvette Unoarumhi, Darlene Wagner, Matthew Schmerer, Ben L. Rambo-Martin, Kristine Lacek, Sam Shepard, Alison Laufer Halpin, Dave Wentworth, Vivien Dugan, Suxiang Tong, Justin Lee
EPI_ISL_2690005	IN State Department of Health Laboratory Services	Centers for Disease Control and Prevention Division of Viral Diseases, Pathogen Discovery	Mili Sheth, Sarah Nobles, Jasmine Padilla, Mark Burroughs, Shoshona Le, Katie Dillon, Peter Cook, Clinton R. Paden, Dhwani Batra, Krista Queen, Kristen Knipe, Dakota Howard, Yvette Unoarumhi, Darlene Wagner, Matthew Schmerer, Ben L. Rambo-Martin, Kristine Lacek, Sam Shepard, Alison Laufer Halpin, Dave Wentworth, Vivien Dugan, Suxiang Tong, Justin Lee
EPI_ISL_2690023	UT-Unified State Labs: Public Health Utah DOH	Centers for Disease Control and Prevention Division of Viral Diseases, Pathogen Discovery	Mili Sheth, Sarah Nobles, Jasmine Padilla, Mark Burroughs, Shoshona Le, Katie Dillon, Peter Cook, Clinton R. Paden, Dhwani Batra, Krista Queen, Kristen Knipe, Dakota Howard, Yvette Unoarumhi, Darlene Wagner, Matthew Schmerer, Ben L. Rambo-Martin, Kristine Lacek, Sam Shepard, Alison Laufer Halpin, Dave Wentworth, Vivien Dugan, Suxiang Tong, Justin Lee
EPI_ISL_2690074	ID Bureau of Laboratories	Centers for Disease Control and Prevention Division of Viral Diseases, Pathogen Discovery	Mili Sheth, Sarah Nobles, Jasmine Padilla, Mark Burroughs, Shoshona Le, Katie Dillon, Peter Cook, Clinton R. Paden, Dhwani Batra, Krista Queen, Kristen Knipe, Dakota Howard, Yvette Unoarumhi, Darlene Wagner, Matthew Schmerer, Ben L. Rambo-Martin, Kristine Lacek, Sam Shepard, Alison Laufer Halpin, Dave Wentworth, Vivien Dugan, Suxiang Tong, Justin Lee
EPI_ISL_2690113	WA State Department of Health	Centers for Disease Control and Prevention Division of Viral Diseases, Pathogen Discovery	Mili Sheth, Sarah Nobles, Jasmine Padilla, Mark Burroughs, Shoshona Le, Katie Dillon, Peter Cook, Clinton R. Paden, Dhwani Batra, Krista Queen, Kristen Knipe, Dakota Howard, Yvette Unoarumhi, Darlene Wagner, Matthew Schmerer, Ben L. Rambo-Martin, Kristine Lacek, Sam Shepard, Alison Laufer Halpin, Dave Wentworth, Vivien Dugan, Suxiang Tong, Justin Lee
EPI_ISL_2690116, EPI_ISL_2690119	PR Public Health Lab	Centers for Disease Control and Prevention Division of Viral Diseases, Pathogen Discovery	Mili Sheth, Sarah Nobles, Jasmine Padilla, Mark Burroughs, Shoshona Le, Katie Dillon, Peter Cook, Clinton R. Paden, Dhwani Batra, Krista Queen, Kristen Knipe, Dakota Howard, Yvette Unoarumhi, Darlene Wagner, Matthew Schmerer, Ben L. Rambo-Martin, Kristine Lacek, Sam Shepard, Alison Laufer Halpin, Dave Wentworth, Vivien Dugan, Suxiang Tong, Justin Lee
EPI_ISL_2690121, EPI_ISL_2690123	MT Public Health Laboratory	Centers for Disease Control and Prevention Division of Viral Diseases, Pathogen Discovery	Mili Sheth, Sarah Nobles, Jasmine Padilla, Mark Burroughs, Shoshona Le, Katie Dillon, Peter Cook, Clinton R. Paden, Dhwani Batra, Krista Queen, Kristen Knipe, Dakota Howard, Yvette Unoarumhi, Darlene Wagner, Matthew Schmerer, Ben L. Rambo-Martin, Kristine Lacek, Sam Shepard, Alison Laufer Halpin, Dave Wentworth, Vivien Dugan, Suxiang Tong, Justin Lee
EPI_ISL_2690357, EPI_ISL_2690387	Arizona State University	Arizona State University	Peter T. Skidmore, LaRinda A. Holland, Matthew F. Smith, Rabia Maqsood, Nicholas J. Mellor, Joy M. Blain, Valerie Harris, Joshua LaBaer, Vel Murugan, Efrem S. Lim
EPI_ISL_2691299	Santa Clara Valley Medical Center	Santa Clara County Public Health Laboratory	Santa Clara County Public Health Department
EPI_ISL_2691950	Rush University Medical Center	RIPHL at Rush University Medical Center	Stefan Green, Kevin Kunstman, Max Kolton, Marieta Hyde, Laura Furtado, Felix Araujo Perez, Mary Hayden, Joyce Houlihan, Diane Springer
EPI_ISL_2692020, EPI_ISL_2692021	University of Chicago Medicine	RIPHL at Rush University Medical Center	Stefan Green, Kevin Kunstman, Max Kolton, Marieta Hyde, Laura Furtado, Felix Araujo Perez, Lauren Megger
EPI_ISL_2693029, EPI_ISL_2693030	Oregon State Public Health Laboratory	Oregon State Public Health Laboratory	Rafia Razzaque, Eugene Yeboah, Vanda Makris, Laura Tsaknaridis, John Fontana and Shane Sevey
EPI_ISL_2693137, EPI_ISL_2693141, EPI_ISL_2693142, EPI_ISL_2693266, EPI_ISL_2693288, EPI_ISL_2693292, EPI_ISL_2693311	UW Virology Lab	UW Virology Lab	Pavitra Roychoudhury, Hong Xie, Lasata Shrestha, Tien V. Nguyen, Shah Mohamed Bakhash, Michelle Lin, Noah R. Baker, Ricardo Perez, Sean Ellis, Nathan Breit, Robert J. Livingston, Meei-Li Huang, Keith R Jerome, Patrick Mathias, Alexander Greninger
EPI_ISL_2693678, EPI_ISL_2693682, EPI_ISL_2693693, EPI_ISL_2693711, EPI_ISL_2693714, EPI_ISL_2693721, EPI_ISL_2693722, EPI_ISL_2693723, EPI_ISL_2693730, EPI_ISL_2693734, EPI_ISL_2693735, EPI_ISL_2693745, EPI_ISL_2693773, EPI_ISL_2693774, EPI_ISL_2693788, EPI_ISL_2693789, EPI_ISL_2693791, EPI_ISL_2693799, EPI_ISL_2693808, EPI_ISL_2693818			
see above	UW Virology Lab	UW Virology Lab	Pavitra Roychoudhury, Hong Xie, Lasata Shrestha, Shah Mohamed Bakhash, Tien V. Nguyen, Noah R. Baker, Sean Ellis, Meei-Li Huang, Keith R Jerome, Alexander Greninger

EPI_ISL_2699440, EPI_ISL_2699466	Public Health Laboratory, Minnesota Department of Health	University of Minnesota Genomics Center	Daryl M. Gohl, John Garbe, Jaquelyn Kuriger-Laber, Corbin Dirkx, and Sean Wang
EPI_ISL_2709537	Aegis Sciences Corporation	Centers for Disease Control and Prevention Division of Viral Diseases, Pathogen Discovery	Dakota Howard, Dhwani Batra, Peter W. Cook, Kara Moser, Adrian Paskey, Jason Caravas, Benjamin Rambo-Martin, Shatavia Morrison, Christopher Gulvick, Scott Sammons, Yvette Unoarumhi, Darlene Wagner, Matthew Schmerer, Cyndi Clark, Patrick Campbell, Rob Case, Vikramsinha Ghorpade, Holly Houdeshell, Ola Kvalvaag, Dillon Nall, Ethan Sanders, Alec Vest, Shaun Westlund, Matthew Hardison, Clinton R. Paden, Duncan MacCannell
EPI_ISL_2709687	LESP Baja California	Instituto de Diagnostico y Referencia Epidemiologicos (INDRE)	Claudia Wong-Arambula, Abril Rodriguez-Maldonado, Vanessa Rivero-Arredondo, Ariadna Medina-Benitez, Joaquin Quiroz-Mercado, Sergio Rangel-Guerrero, Natividad Cruz-Ortiz, Tatiana Nunez-Garcia, Gisela Barrera-Badillo, Lucia Hernandez-Rivas, Irma Lopez-Martinez, Ernesto Ramirez-Gonzalez.
EPI_ISL_2710201	HOSPITAL UNIVERSITARIO SON ESPASES	HOSPITAL UNIVERSITARIO SON ESPASES	Servicio de Microbiología, Hospital Universitario Son Espases
EPI_ISL_2712543, EPI_ISL_2712549, EPI_ISL_2712553	Sharp HealthCare Laboratory	Andersen lab at Scripps Research	SEARCH Alliance San Diego with Aaron Harding, Jacquelyn Berumen, Cathy Woerle, Liam McGinnis, Art Mendoza, Omid Bakhtar
EPI_ISL_2713062	Oregon State Public Health Laboratory	Oregon State Public Health Laboratory	Rafia Razzaque, Eugene Yeboah, Vanda Makris, Laura Tsaknaridis, John Fontana and Shane Sevey
EPI_ISL_2713292	Infectious Disease Program, Broad Institute of Harvard and MIT	Infectious Disease Program, Broad Institute of Harvard and MIT	Lemieux,J.E., Siddle,K.J., Shaw,B., Adams,G., Pierce,V., Turbett,S., Anahtar,M., Branda,J., Slater,D., Harris,J., Lin,A.E., Gladden-Young,A., Lagerborg,K., Rudy,M., DeRuff,K., Carter,A., Normandin,E., Bauer,M., Reilly,S., Tomkins-Tinch,C., Loreth,C., Chaluvadi,S., Neumann,A., Cusick,C., Chapman,S.B., Gnirke,A., Flowers,K., Cerrato,F., Birren,B.W., Gallagher,G., Smole,S., Park,D.J., MacInnis,B.L., Ryan,E., LaRocque,R., Rosenberg,E., Sabeti,P.C.
EPI_ISL_2715247	North Dakota Department of Health, Public Health Laboratory	North Dakota Department of Health, Public Health Laboratory	Lisa Wingerter
EPI_ISL_2716212, EPI_ISL_2716213, EPI_ISL_2716214	The Jackson Laboratory	The Jackson Laboratory	Long J, Renzette N, Adams M, Omerza G, Kelly K, Li L
EPI_ISL_2716222, EPI_ISL_2716229, EPI_ISL_2716240, EPI_ISL_2716241, EPI_ISL_2716253, EPI_ISL_2716254	Yale Clinical Virology Lab	Grubaugh Lab - Yale School of Public Health	Joseph Fauver, Mallery Breban, Isabel Ott, Tara Alpert, Mary Petrone, Anderson Brito, Chantal Vogels, Annie Watkins, Chaney Kalinich, Jessica Rothman, Marie L. Landry, Nathan Grubaugh
EPI_ISL_2716916	Southern Nevada Public Health Laboratory	Southern Nevada Public Health Laboratory	Michael Picker
EPI_ISL_2725058	IN State Department of Health Laboratory Services	IN State Department of Health Laboratory Services	Cassandra Campion, Jamie Yeadon, Brian Pope, Lixia Liu, Kyle Brownlee, Melissa Hindenlang, Mark Glazier
EPI_ISL_2725591	Rhode Island State Health Laboratory	Rhode Island State Health Laboratory	Ewa King, Richard C. Huard, Kristin Carpenter-Azevedo
EPI_ISL_2727104, EPI_ISL_2727113	ALBANY MEDICAL CENTER	Wadsworth Center, New York State Department of Health	Kirsten St. George, Daryl M. Lamson, Alexis Russell, Matthew Shudt, Melissa A Leisner, Jonathan Plitnick, Catharine Prussing, Navjot Singh, John Kelly, Erasmus Schneider, Erica Lasek-Nesselquist
EPI_ISL_2727473	Oregon State Public Health Laboratory	Oregon State Public Health Laboratory	Rafia Razzaque, Eugene Yeboah, Vanda Makris, Laura Tsaknaridis, John Fontana and Shane Sevey
EPI_ISL_2727572, EPI_ISL_2727579, EPI_ISL_2727588	NJDOH, Public Health and Environmental Laboratories	NJ_PHEL	Lindsey Bodnar, Shiv K. Verma, Jacquelyn Deverell, Ryan Pachucki, Dana Woell, Allison Roder, Byeong Jeong
EPI_ISL_2727726, EPI_ISL_2727728, EPI_ISL_2727737, EPI_ISL_2727743, EPI_ISL_2727784, EPI_ISL_2727802, EPI_ISL_2727805	Wisconsin State Laboratory of Hygiene Communicable Disease Division	Wisconsin State Laboratory of Hygiene Communicable Disease Division	Abigail C. Shockey, Alicia J. Mooney, Erika M. Hanson, Tonya Danz, Richard Griesser, Sara Wagner, Kelsey R. Florek
EPI_ISL_2727871	Centracare Laboratory Services	Minnesota Department of Health, Public Health Laboratory	Alexandra Lorentz, Jacob Garfin, Matt Plumb, and Xiong Wang
EPI_ISL_2728144	Wyoming Public Health Laboratory	Wyoming Public Health Laboratory	Jim Mildenberger, Taylor Fearing, Channing Weber, Ashley Norberg, Chayse Rowley, Marley Goetz, Brian Dominguez, Elliot Thomasson, Sam Britz, Cari Sloma, Robert Petit, and Rob Christensen
EPI_ISL_2735900	Seattle Flu Study	Seattle Flu Study	Deborah A. Nickerson, Chris D. Frazar, Jover Lee, Benjamin Pelle, Erica Ryke, Matthew Richardson, Amanda Adler, Elisabeth Brandstetter, Peter D. Han, Kairsten Fay, Misja Ilcisin, Kirsten Lacombe, Thomas R. Sibley, Melissa Truong, Caitlin R. Wolf, Karen Cowgill, Stephanie Schrag, Jeff Duchin, Michael Boeckh, Janet A. Englund, Michael Famulare, Barry R. Lutz, Mark J. Rieder, Lea M. Starita, Matthew Thompson, Helen Y. Chu, Trevor Bedford, Jay Shendure
EPI_ISL_2735987	Incyte Diagnostics Spokane	Seattle Flu Study	Deborah A. Nickerson, Chris D. Frazar, Jover Lee, Benjamin Pelle, Erica Ryke, Matthew Richardson, Amanda Adler, Elisabeth Brandstetter, Peter D. Han, Kairsten Fay, Misja Ilcisin, Kirsten Lacombe, Thomas R. Sibley, Melissa Truong, Caitlin R. Wolf, Romesh Gautom, Geoff Melly, Brian Hiatt, Philip Dykema, Scott Lindquist, Michael Boeckh, Janet A. Englund, Michael Famulare, Barry R. Lutz, Mark J. Rieder, Lea M. Starita, Matthew Thompson, Helen Y. Chu, Jay Shendure, Trevor Bedford
EPI_ISL_2736230	Atlas Genomics	Seattle Flu Study	Deborah A. Nickerson, Chris D. Frazar, Jover Lee, Benjamin Pelle, Erica Ryke, Matthew Richardson, Amanda Adler, Elisabeth Brandstetter, Peter D. Han, Kairsten

			Fay, Misja Ilcisin, Kirsten Lacombe, Thomas R. Sibley, Melissa Truong, Caitlin R. Wolf, Romesh Gautom, Geoff Melly, Brian Hiatt, Philip Dykema, Scott Lindquist, Michael Boeckh, Janet A. Englund, Michael Famulare, Barry R. Lutz, Mark J. Rieder, Lea M. Starita, Matthew Thompson, Helen Y. Chu, Jay Shendure, Trevor Bedford
EPI_ISL_2736837	LESP Tabasco	Instituto de Diagnostico y Referencia Epidemiologicos (INDRE)	Claudia Wong-Arambula, Abril Rodriguez-Maldonado, Vanessa Rivero-Arredondo, Ariadna Medina-Benitez, Joaquin Quiroz-Mercado, Sergio Rangel-Guerrero, Natividad Cruz-Ortiz, Tatiana Nunez-Garcia, Gisela Barrera-Badillo, Lucia Hernandez-Rivas, Irma Lopez-Martinez, Ernesto Ramirez-Gonzalez.
EPI_ISL_2753764	Missouri State Public Health Laboratory	Missouri State Public Health Laboratory	Matthew Sinn, Joshua Barry, Ashley New
EPI_ISL_2754008	Kaiser Regional	Santa Clara County Public Health Laboratory	Santa Clara County Public Health Department
EPI_ISL_2754921	GA Department of Public Health Laboratory	Genomics and Discovery, Respiratory Viruses Branch, Division of Viral Diseases, Centers for Disease Control and Prevention	Anna Kelleher, Ying Tao, Yan Li, Jing Zhang, Brian Lynch, Krista Queen, Anna Uehara, Peter Cook, Han Jia Justin Ng, Rachel Marine, Clinton R. Paden, Dhwani Batra, Haibin Wang, Tara Coalter, Jasmine Padilla, Morgan Davis, Mili Sheth, Sarah Nobles, Mark Burroughs, Justin Lee, Adam Retchless, Suxiang Tong
EPI_ISL_2757590, EPI_ISL_2757591, EPI_ISL_2757601, EPI_ISL_2757603, EPI_ISL_2757605, EPI_ISL_2757606, EPI_ISL_2757607, EPI_ISL_2757610, EPI_ISL_2757615, EPI_ISL_2757617, EPI_ISL_2757620, EPI_ISL_2757625, EPI_ISL_2757627, EPI_ISL_2757631, EPI_ISL_2757632, EPI_ISL_2757634, EPI_ISL_2757637, EPI_ISL_2757639, EPI_ISL_2757643, EPI_ISL_2757646, EPI_ISL_2757648			
see above	INSPI-CRN DE INFLUENZA Y OTROS VIRUS RESPIRATORIOS	NIC-INSPI	Alfredo Bruno , Maritza Olmedo, Michelle Páez, Jimmy Garcés, Johanna Laines, Lizbeth Patiño, Manuel Gonzalez, Domenica de Mora.
EPI_ISL_2758278	Berkeley Medical Center	WVU and Marshall University Combined Genomics Core Facilities	James Denvir, Peter Stoilov, Peter Perrotta, Wesley Kimble, Ryan Percifield
EPI_ISL_2758354, EPI_ISL_2758355, EPI_ISL_2758357, EPI_ISL_2758381, EPI_ISL_2758384, EPI_ISL_2758387, EPI_ISL_2758389, EPI_ISL_2758393	QLabs	WVU and Marshall University Combined Genomics Core Facilities	James Denvir, Peter Stoilov, Peter Perrotta, Wesley Kimble, Ryan Percifield
EPI_ISL_2758617	Microbiology Division, South Carolina Department of Health and Environmental Control	Microbiology Division, South Carolina Department of Health and Environmental Control	Freeman,J.S., Flores,H.V.
EPI_ISL_2758887, EPI_ISL_2758908	The Ohio State University Applied Microbiology Services Laboratory	The Ohio State University Applied Microbiology Services Laboratory	Seth A. Faith PhD
EPI_ISL_2769232	TGen North	TGen North	"Jolene Bowers, Heather Centner, Brett Van Tassel, Chris French, Hayley Yaglom, Darrin Lemmer, Dave Engelthaler"
EPI_ISL_2774272	Baylor Scott & White-Temple	Baylor Scott & White-Temple	Ari Rao, Linden Morales, Kimberly Walker, Marcus Volz, Shelby Johnson
EPI_ISL_2774596, EPI_ISL_2774608, EPI_ISL_2774613	Santa Clara Valley Medical Center	Santa Clara County Public Health Laboratory	Santa Clara County Public Health Department

Supp. Table S9

We gratefully acknowledge the following Authors from the Originating laboratories responsible for obtaining the specimens, as well as the Submitting laboratories where the genome data were generated and shared via GISAID, on which this research is based.

All Submitters of data may be contacted directly via www.gisaid.org

Authors are sorted alphabetically.

Acknowledgement EPI_SET Identifier: EPI_SET_20220408ow

Accession ID	Originating Laboratory	Submitting Laboratory	Authors
EPI_ISL_9602934, EPI_ISL_9602935, EPI_ISL_9602936, EPI_ISL_9602937, EPI_ISL_9602945	C H LEON BINET	CERBA HealthCare	Bénédicte Roquebert; Laura Verdurme; Mathilde Roussel; Sabine Trombert; Stéphanie Haim-Boukobza
EPI_ISL_9602914, EPI_ISL_9602916	CERBALLIANCE BOURGOGNE SITE DIJON VALMY	CERBA HealthCare	Bénédicte Roquebert; Laura Verdurme; Mathilde Roussel; Sabine Trombert; Stéphanie Haim-Boukobza
EPI_ISL_9602919, EPI_ISL_9602920, EPI_ISL_9602921, EPI_ISL_9602922, EPI_ISL_9602923, EPI_ISL_9602924, EPI_ISL_9602925, EPI_ISL_9602926, EPI_ISL_9602947, EPI_ISL_9602948, EPI_ISL_9602949			
see above	CERBALLIANCE HAUTS DE FRANCE	CERBA HealthCare	Bénédicte Roquebert; Laura Verdurme; Mathilde Roussel; Sabine Trombert; Stéphanie Haim-Boukobza
EPI_ISL_9602913	CERBALLIANCE NORMANDIE OUEST CARENTAN	CERBA HealthCare	Bénédicte Roquebert; Laura Verdurme; Mathilde Roussel; Sabine Trombert; Stéphanie Haim-Boukobza
EPI_ISL_9602930, EPI_ISL_9602931, EPI_ISL_9602932, EPI_ISL_9602933, EPI_ISL_9602950, EPI_ISL_9602951	CERBALLIANCE PARIS ET IDF EST	CERBA HealthCare	Bénédicte Roquebert; Laura Verdurme; Mathilde Roussel; Sabine Trombert; Stéphanie Haim-Boukobza
EPI_ISL_9604897	CH William MOREY	Department of Virology, Henri Mondor University Hospital, Assistance Publique Hôpitaux de Paris, Université Paris-Est Créteil, INSERM U955	Alexandre Soulier; Christophe Rodriguez; Elisabeth Trawinski; Guillaume Gricourt; Jean-Michel Pawlotsky; Melissa N'Debi; Slim Fourati; Vanessa Demontant
EPI_ISL_9430471, EPI_ISL_9430490, EPI_ISL_9430509, EPI_ISL_9430527, EPI_ISL_9651140	CSIR-NEERI, Nagpur Covid-19 Testing Lab	CSIR-NEERI, Nagpur Covid-19 Testing Lab	Krishna Khairnar et al.
EPI_ISL_9523229	Center of Advanced Studies and Technology, Molecular Genetics Laboratory	Center of Advanced Studies and Technology, Molecular Genetics Laboratory	Anaclerio Federico; Liborio Stuppia
EPI_ISL_9563117, EPI_ISL_9563553, EPI_ISL_9563944, EPI_ISL_9564040, EPI_ISL_9564183, EPI_ISL_9564261, EPI_ISL_9564409, EPI_ISL_9564452, EPI_ISL_9564694, EPI_ISL_9564727, EPI_ISL_9564963, EPI_ISL_9565195, EPI_ISL_9565385, EPI_ISL_9565886, EPI_ISL_9566398, EPI_ISL_9566748, EPI_ISL_9566828, EPI_ISL_9566880, EPI_ISL_9566933, EPI_ISL_9567009, EPI_ISL_9567053, EPI_ISL_9567150, EPI_ISL_9567249, EPI_ISL_9567285, EPI_ISL_9567296, EPI_ISL_9567547, EPI_ISL_9567567, EPI_ISL_9567689, EPI_ISL_9567801, EPI_ISL_9567885, EPI_ISL_9567893, EPI_ISL_9567945, EPI_ISL_9567972, EPI_ISL_9567997, EPI_ISL_9568039, EPI_ISL_9568145, EPI_ISL_9568289, EPI_ISL_9568308, EPI_ISL_9568326, EPI_ISL_9568343, EPI_ISL_9568353, EPI_ISL_9568368, EPI_ISL_9568390, EPI_ISL_9568797, EPI_ISL_9568978, EPI_ISL_9569114, EPI_ISL_9569154, EPI_ISL_9569168, EPI_ISL_9569204, EPI_ISL_9569338, EPI_ISL_9569401, EPI_ISL_9569402, EPI_ISL_9569415, EPI_ISL_9569420, EPI_ISL_9569440, EPI_ISL_9569460, EPI_ISL_9569675, EPI_ISL_9650689, EPI_ISL_9650695, EPI_ISL_9650697, EPI_ISL_9650701, EPI_ISL_9650702, EPI_ISL_9650710, EPI_ISL_9650714, EPI_ISL_9650715, EPI_ISL_9650719, EPI_ISL_9650740, EPI_ISL_9650751, EPI_ISL_9650756, EPI_ISL_9650761, EPI_ISL_9650763, EPI_ISL_9650764, EPI_ISL_9650767, EPI_ISL_9650775, EPI_ISL_9650776, EPI_ISL_9650780, EPI_ISL_9650789, EPI_ISL_9650792, EPI_ISL_9650806, EPI_ISL_9650904, EPI_ISL_9650906, EPI_ISL_9650908, EPI_ISL_9650914, EPI_ISL_9650916, EPI_ISL_9650920, EPI_ISL_9650922, EPI_ISL_9650924, EPI_ISL_9650927, EPI_ISL_9650935, EPI_ISL_9650937, EPI_ISL_9650938, EPI_ISL_9650943, EPI_ISL_9650945, EPI_ISL_9650957, EPI_ISL_9650959, EPI_ISL_9650961, EPI_ISL_9650963, EPI_ISL_9650964, EPI_ISL_9651012, EPI_ISL_9651015, EPI_ISL_9651019, EPI_ISL_9651021, EPI_ISL_9651025, EPI_ISL_9651032, EPI_ISL_9651034, EPI_ISL_9651041, EPI_ISL_9651043, EPI_ISL_9651046, EPI_ISL_9651081, EPI_ISL_9651087, EPI_ISL_9651090, EPI_ISL_9651092, EPI_ISL_9651175, EPI_ISL_9651177, EPI_ISL_9651187, EPI_ISL_9651192, EPI_ISL_9651196, EPI_ISL_9651198, EPI_ISL_9651220, EPI_ISL_9651223, EPI_ISL_9651230, EPI_ISL_9651232, EPI_ISL_9651235, EPI_ISL_9651237, EPI_ISL_9651351, EPI_ISL_9651355, EPI_ISL_9651358, EPI_ISL_9651363, EPI_ISL_9651364, EPI_ISL_9651367, EPI_ISL_9651369, EPI_ISL_9651370, EPI_ISL_9651376, EPI_ISL_9651377, EPI_ISL_9651378, EPI_ISL_9651385, EPI_ISL_9651394, EPI_ISL_9651400, EPI_ISL_9651407, EPI_ISL_9651418, EPI_ISL_9651424, EPI_ISL_9651429, EPI_ISL_9651434, EPI_ISL_9651452, EPI_ISL_9651453, EPI_ISL_9651465, EPI_ISL_9651466, EPI_ISL_9651469, EPI_ISL_9651504, EPI_ISL_9651512, EPI_ISL_9651514, EPI_ISL_9651526, EPI_ISL_9651527, EPI_ISL_9651541, EPI_ISL_9651557, EPI_ISL_9651561, EPI_ISL_9651563, EPI_ISL_9651586, EPI_ISL_9651622, EPI_ISL_9651623, EPI_ISL_9651626, EPI_ISL_9651628, EPI_ISL_9651638, EPI_ISL_9651639, EPI_ISL_9651642, EPI_ISL_9651643, EPI_ISL_9651650, EPI_ISL_9651651, EPI_ISL_9651678, EPI_ISL_9651705, EPI_ISL_9651717, EPI_ISL_9651723, EPI_ISL_9651725, EPI_ISL_9651738, EPI_ISL_9651740, EPI_ISL_9651747, EPI_ISL_9651752, EPI_ISL_9651753, EPI_ISL_9651755, EPI_ISL_9651756, EPI_ISL_9651758, EPI_ISL_9651761, EPI_ISL_9651764, EPI_ISL_9651770, EPI_ISL_9651776, EPI_ISL_9651778, EPI_ISL_9651779, EPI_ISL_9651782, EPI_ISL_9651784, EPI_ISL_9651786, EPI_ISL_9651792, EPI_ISL_9651795, EPI_ISL_9651799, EPI_ISL_9651800, EPI_ISL_9651808, EPI_ISL_9651809, EPI_ISL_9651810, EPI_ISL_9651812, EPI_ISL_9651817, EPI_ISL_9651820, EPI_ISL_9651823, EPI_ISL_9651825, EPI_ISL_9651837, EPI_ISL_9651840, EPI_ISL_9651851, EPI_ISL_9651853, EPI_ISL_9651857, EPI_ISL_9651858, EPI_ISL_9651862, EPI_ISL_9651863, EPI_ISL_9651864, EPI_ISL_9651868, EPI_ISL_9651871, EPI_ISL_9651872, EPI_ISL_9651874, EPI_ISL_9651887, EPI_ISL_9651890, EPI_ISL_9651894, EPI_ISL_9651904, EPI_ISL_9651911, EPI_ISL_9651914, EPI_ISL_9651916, EPI_ISL_9651919, EPI_ISL_9651924, EPI_ISL_9651925, EPI_ISL_9651926, EPI_ISL_9651984, EPI_ISL_9651987, EPI_ISL_9652003, EPI_ISL_9652008, EPI_ISL_9652015, EPI_ISL_9652018, EPI_ISL_9652021, EPI_ISL_9652029, EPI_ISL_9652033, EPI_ISL_9652034, EPI_ISL_9652044, EPI_ISL_9652047, EPI_ISL_9652048, EPI_ISL_9652050, EPI_ISL_9652051, EPI_ISL_9652053, EPI_ISL_9652055, EPI_ISL_9652056, EPI_ISL_9652064, EPI_ISL_9652068, EPI_ISL_9652114, EPI_ISL_9652115, EPI_ISL_9652120, EPI_ISL_9652144, EPI_ISL_9652153, EPI_ISL_9652156, EPI_ISL_9652162, EPI_ISL_9652172, EPI_ISL_9652174, EPI_ISL_9652179, EPI_ISL_9652182, EPI_ISL_9652184, EPI_ISL_9652185, EPI_ISL_9652186, EPI_ISL_9652189, EPI_ISL_9652191, EPI_ISL_9652200, EPI_ISL_9652202, EPI_ISL_9652204, EPI_ISL_9652210, EPI_ISL_9652219, EPI_ISL_9652225, EPI_ISL_9652228, EPI_ISL_9652232, EPI_ISL_9652240, EPI_ISL_9652244, EPI_ISL_9652245, EPI_ISL_9652246, EPI_ISL_9652247, EPI_ISL_9652249, EPI_ISL_9652252, EPI_ISL_9652258, EPI_ISL_9652260, EPI_ISL_9652262, EPI_ISL_9652267, EPI_ISL_9652277, EPI_ISL_9652278, EPI_ISL_9652279, EPI_ISL_9652281, EPI_ISL_9652285, EPI_ISL_9652289, EPI_ISL_9652300, EPI_ISL_9652386, EPI_ISL_9652406, EPI_ISL_9652408, EPI_ISL_9652423, EPI_ISL_9652424, EPI_ISL_9652432, EPI_ISL_9652442, EPI_ISL_9652445, EPI_ISL_9652446, EPI_ISL_9652447, EPI_ISL_9652449, EPI_ISL_9652450, EPI_ISL_9652454, EPI_ISL_9652456, EPI_ISL_9652459, EPI_ISL_9652460, EPI_ISL_9652462, EPI_ISL_9652465, EPI_ISL_9652473, EPI_ISL_9652482, EPI_ISL_9652484, EPI_ISL_9652500, EPI_ISL_9652502, EPI_ISL_9652513, EPI_ISL_9652529, EPI_ISL_9652531, EPI_ISL_9652538, EPI_ISL_9652539, EPI_ISL_9652541, EPI_ISL_9652549, EPI_ISL_9652552, EPI_ISL_9652556, EPI_ISL_9652557, EPI_ISL_9652559, EPI_ISL_9652564, EPI_ISL_9652567, EPI_ISL_9652569, EPI_ISL_9652570, EPI_ISL_9652572, EPI_ISL_9652578, EPI_ISL_9652579, EPI_ISL_9652581, EPI_ISL_9652584, EPI_ISL_9652595, EPI_ISL_9652596, EPI_ISL_9652599, EPI_ISL_9652603, EPI_ISL_9652604, EPI_ISL_9652608, EPI_ISL_9652611, EPI_ISL_9652612, EPI_ISL_9652613, EPI_ISL_9652614, EPI_ISL_9652625, EPI_ISL_9652628, EPI_ISL_9652632, EPI_ISL_9652639, EPI_ISL_9652640, EPI_ISL_9652644, EPI_ISL_9652648, EPI_ISL_9652651, EPI_ISL_9652652, EPI_ISL_9652656, EPI_ISL_9652660, EPI_ISL_9652667, EPI_ISL_9652669, EPI_ISL_9652673, EPI_ISL_9652675, EPI_ISL_9652676, EPI_ISL_9652680, EPI_ISL_9652684, EPI_ISL_9652691, EPI_ISL_9652707, EPI_ISL_9652709, EPI_ISL_9652712, EPI_ISL_9652713, EPI_ISL_9652715, EPI_ISL_9652717, EPI_ISL_9652718, EPI_ISL_9652726, EPI_ISL_9652727, EPI_ISL_9652733, EPI_ISL_9652734, EPI_ISL_9652737, EPI_ISL_9652740, EPI_ISL_9652741, EPI_ISL_9652744, EPI_ISL_9652745, EPI_ISL_9652749, EPI_ISL_9652757, EPI_ISL_9652771, EPI_ISL_9652772, EPI_ISL_9652774, EPI_ISL_9652775, EPI_ISL_9652776, EPI_ISL_9652779, EPI_ISL_9652780, EPI_ISL_9652783, EPI_ISL_9652785, EPI_ISL_9652788, EPI_ISL_9652793, EPI_ISL_9652801, EPI_ISL_9652810, EPI_ISL_9652814, EPI_ISL_9652816, EPI_ISL_9652829, EPI_ISL_9652830, EPI_ISL_9652838, EPI_ISL_9652846, EPI_ISL_9652850, EPI_ISL_9652851, EPI_ISL_9652855, EPI_ISL_9652869, EPI_ISL_9652879, EPI_ISL_9652881, EPI_ISL_9652884, EPI_ISL_9652896, EPI_ISL_9652898, EPI_ISL_9652902, EPI_ISL_9652913, EPI_ISL_9652915, EPI_ISL_9652916, EPI_ISL_9652919, EPI_ISL_9652921, EPI_ISL_9652922, EPI_ISL_9652923			
see above	Department of Bacteria, Parasites and Fungi, Statens Serum Institut, Copenhagen, Denmark	Statens Serum Institut Bioinformatics and Microbial Genomics	Danish Covid-19 Genome Consortium
EPI_ISL_9615096, EPI_ISL_9615099, EPI_ISL_9615101, EPI_ISL_9615108, EPI_ISL_9615111, EPI_ISL_9615116, EPI_ISL_9615117			
see above	Dept. of Microbiology and Infection Control, Akershus University Hospital HF	Dept. of Microbiology and Infection Control, Akershus University Hospital HF	Alexander Hesselberg Løvestad; Hanne Berggreen; Hege Vangstein Aamot
EPI_ISL_9612629	Hopital	National Reference Center for Viruses of Respiratory Infections, Institut Pasteur, Paris	Angela Brisebarre; Camille Capel; Christophe Malabat; Corinne Maufrais; Etienne Simon-Lorière; Frédéric Lemoine; Julien Fumey; Louise Lefrançois; Marion Barbet; Maud Vanpeene; Méline Bizard; Sandrine CASTELAIN; Slim El Khiari; Sylvie Behillil; Sylvie Van der Werf; Vincent Enouf
EPI_ISL_9489590	Institute for Infectious Diseases	Institute for Infectious Diseases, University of Bern	Alban Ramette; Christian Baumann; Cora Sägesser; Franziska Suter-Riniker; Loïc Borcard; Miguel A Terrazos Miani; Nicole Liechti; Pascal Bittel; Peter Keller; Sonja Gempeler; Stefan Neuenschwander; Stephen L Leib
EPI_ISL_9651930	Jessa	Jessa	Severine Berden et al. on behalf of the Jessa_cmdLab
EPI_ISL_9627723	Kansas Health and Environmental Lab	Kansas Health and Environmental Lab	Amanda Bradley; Carrie Welch; Gary Buruss; Jonathan Barnell; Mike Grose; Victor Anderson; and Phil Adam
EPI_ISL_9602928, EPI_ISL_9602929	LABM CEA SACLAY	CERBA HealthCare	Bénédicte Roquebert; Laura Verdurme; Mathilde Roussel; Sabine Trombert; Stéphanie Haim-Boukobza
EPI_ISL_9602938, EPI_ISL_9602952	LBA - SITE DE LECTOURE	CERBA HealthCare	Bénédicte Roquebert; Laura Verdurme; Mathilde Roussel; Sabine Trombert; Stéphanie Haim-Boukobza
EPI_ISL_9603879	LKO	Jessa	Severine Berden et al. on behalf of the Jessa_cmdLab
EPI_ISL_9640610, EPI_ISL_9640612, EPI_ISL_9640615, EPI_ISL_9640618, EPI_ISL_9640626, EPI_ISL_9640634, EPI_ISL_9640637, EPI_ISL_9640642, EPI_ISL_9640650, EPI_ISL_9640652, EPI_ISL_9640655, EPI_ISL_9640661, EPI_ISL_9640664, EPI_ISL_9640666, EPI_ISL_9640673, EPI_ISL_9640676, EPI_ISL_9640681			
see above	Lab. Microbiologia e Virologia Cotugno A.O. dei Colli	TIGEM	Antonio Grimaldi Patrizia Annunziata Francesco Panariello Claudia Tiberio Teresa Giuliano Valentina Bouche Chiara Colantuono Michela Daniele Lucio Di Filippo Anna Manfredi Marcello Salvi Antonio Limone Luigi Atripaldi Andrea Ballabio Davide Cacchiarelli
EPI_ISL_9646270	Lighthouse Lab in Alderley Park	Wellcome Sanger Institute for the COVID-19 Genomics UK (COG-UK) Consortium	Cordelia Langford; David K. Jackson; Dominic Kwiatkowski; Ewan Harrison; Ian Johnston; Jacquelyn Wynn; Jeffrey Barrett; John Sillitoe on behalf of the Wellcome Sanger Institute COVID-19 Surveillance Team; Mairead Hyland; Roberto Amato; Sonia Goncalves; The Lighthouse Lab in Alderley Park and Alex Alderton
EPI_ISL_9620810, EPI_ISL_9620913	Lighthouse Lab in Milton Keynes	Wellcome Sanger Institute for the COVID-19 Genomics UK (COG-UK) Consortium	Cordelia Langford; David K. Jackson; Dominic Kwiatkowski; Ewan Harrison; Ian Johnston; Jeffrey Barrett; John Sillitoe on behalf of the Wellcome Sanger Institute COVID-19 Surveillance Team; Roberto Amato; Sonia Goncalves; The Lighthouse Lab in Milton Keynes and Alex Alderton
EPI_ISL_9596876, EPI_ISL_9596877, EPI_ISL_9596878, EPI_ISL_9596879, EPI_ISL_9596880, EPI_ISL_9596881, EPI_ISL_9596882, EPI_ISL_9596884, EPI_ISL_9596885, EPI_ISL_9596886, EPI_ISL_9596887, EPI_ISL_9596888, EPI_ISL_9596889, EPI_ISL_9596890, EPI_ISL_9596891, EPI_ISL_9596892			
see above	National Public Health Laboratory, National Centre for Infectious Diseases	National Public Health Laboratory, National Centre for Infectious Diseases	Benny Yeo; Grace Ngan; Lin Cui; Raymond Tzer Pin Lin; Royce Ang; Samuel Loo; Yichen Ding; Zhenyang Zhou
EPI_ISL_9637625	Nevada State Public Health Laboratory	Nevada State Public Health Laboratory	Andrew Gorzalski; Lynette Gumbleton; Mark Pandori
EPI_ISL_9651494	U.O. Microbiologia Laboratorio Unico Centro Servizi - AUSL della Romagna	U.O. Microbiologia, Laboratorio Unico Centro Servizi - AUSL della Romagna	GiorgioDirani

We gratefully acknowledge the following Authors from the Originating laboratories responsible for obtaining the specimens, as well as the Submitting laboratories where the genome data were generated and shared via GISAID, on which this research is based.

All Submitters of data may be contacted directly via www.gisaid.org

Authors are sorted alphabetically.

Acknowledgement EPI_SET Identifier: EPI_SET_20220408tc

EPI_ISL_9652924, EPI_ISL_9652925, EPI_ISL_9652927, EPI_ISL_9652929, EPI_ISL_9652930, EPI_ISL_9652933, EPI_ISL_9652935, EPI_ISL_9652940, EPI_ISL_9652942, EPI_ISL_9652944, EPI_ISL_9652952, EPI_ISL_9652958, EPI_ISL_9652974, EPI_ISL_9652976, EPI_ISL_9652981, EPI_ISL_9652991, EPI_ISL_9652992, EPI_ISL_9652999, EPI_ISL_9653009, EPI_ISL_9653010, EPI_ISL_9653011, EPI_ISL_9653023, EPI_ISL_9653024, EPI_ISL_9653025, EPI_ISL_9653029, EPI_ISL_9653032, EPI_ISL_9653035, EPI_ISL_9653038, EPI_ISL_9653044, EPI_ISL_9653045, EPI_ISL_9653046, EPI_ISL_9653047, EPI_ISL_9653049, EPI_ISL_9653051, EPI_ISL_9653052, EPI_ISL_9653057, EPI_ISL_9653060, EPI_ISL_9653063, EPI_ISL_9653065, EPI_ISL_9653066, EPI_ISL_9653072, EPI_ISL_9653073, EPI_ISL_9653074, EPI_ISL_9653083, EPI_ISL_9653090, EPI_ISL_9653092, EPI_ISL_9653093, EPI_ISL_9653097, EPI_ISL_9653098, EPI_ISL_9653099, EPI_ISL_9653100, EPI_ISL_9653106, EPI_ISL_9653107, EPI_ISL_9653110, EPI_ISL_9653115, EPI_ISL_9653119, EPI_ISL_9653131, EPI_ISL_9653142, EPI_ISL_9653144, EPI_ISL_9653150, EPI_ISL_9653151, EPI_ISL_9653152, EPI_ISL_9653153, EPI_ISL_9653154, EPI_ISL_9653156, EPI_ISL_9653159, EPI_ISL_9653162, EPI_ISL_9653167, EPI_ISL_9653168, EPI_ISL_9653175, EPI_ISL_9653177, EPI_ISL_9653179, EPI_ISL_9653181, EPI_ISL_9653184, EPI_ISL_9653185, EPI_ISL_9653188, EPI_ISL_9653195, EPI_ISL_9653204, EPI_ISL_9653205, EPI_ISL_9653214, EPI_ISL_9653215, EPI_ISL_9653216, EPI_ISL_9653218, EPI_ISL_9653223, EPI_ISL_9653224, EPI_ISL_9653227, EPI_ISL_9653229, EPI_ISL_9653233, EPI_ISL_9653235, EPI_ISL_9653238, EPI_ISL_9653240, EPI_ISL_9653243, EPI_ISL_9653255, EPI_ISL_9653257, EPI_ISL_9653258, EPI_ISL_9653262, EPI_ISL_9653269, EPI_ISL_9653271, EPI_ISL_9653272, EPI_ISL_9653273, EPI_ISL_9653274, EPI_ISL_9653286, EPI_ISL_9653291, EPI_ISL_9653297, EPI_ISL_9653298, EPI_ISL_9653299, EPI_ISL_9653300, EPI_ISL_9653305, EPI_ISL_9653308, EPI_ISL_9653310, EPI_ISL_9653311, EPI_ISL_9653312, EPI_ISL_9653313, EPI_ISL_9653316, EPI_ISL_9653318, EPI_ISL_9653320, EPI_ISL_9653330, EPI_ISL_9653338, EPI_ISL_9653340, EPI_ISL_9653344, EPI_ISL_9653347, EPI_ISL_9653350, EPI_ISL_9653352, EPI_ISL_9653354, EPI_ISL_9653356, EPI_ISL_9653359, EPI_ISL_9653360, EPI_ISL_9653361, EPI_ISL_9653365, EPI_ISL_9653370, EPI_ISL_9653376, EPI_ISL_9653385, EPI_ISL_9653402, EPI_ISL_9653410, EPI_ISL_9653412, EPI_ISL_9653414, EPI_ISL_9653418, EPI_ISL_9653427, EPI_ISL_9653428, EPI_ISL_9653429, EPI_ISL_9653437, EPI_ISL_9653438, EPI_ISL_9653445, EPI_ISL_9653448, EPI_ISL_9653451, EPI_ISL_9653457, EPI_ISL_9653459, EPI_ISL_9653461, EPI_ISL_9653462, EPI_ISL_9653463, EPI_ISL_9653466, EPI_ISL_9653467, EPI_ISL_9653468, EPI_ISL_9653470, EPI_ISL_9653471, EPI_ISL_9653476, EPI_ISL_9653489, EPI_ISL_9653492, EPI_ISL_9653494, EPI_ISL_9653496, EPI_ISL_9653498, EPI_ISL_9653502, EPI_ISL_9653504, EPI_ISL_9653509, EPI_ISL_9653510, EPI_ISL_9653516, EPI_ISL_9653523, EPI_ISL_9653524, EPI_ISL_9653527, EPI_ISL_9653530, EPI_ISL_9653539, EPI_ISL_9653540, EPI_ISL_9653542, EPI_ISL_9653543, EPI_ISL_9653546, EPI_ISL_9653549, EPI_ISL_9653550, EPI_ISL_9653552, EPI_ISL_9653557, EPI_ISL_9653559, EPI_ISL_9653568, EPI_ISL_9653571, EPI_ISL_9653572, EPI_ISL_9653573, EPI_ISL_9653574, EPI_ISL_9653578, EPI_ISL_9653585, EPI_ISL_9653586, EPI_ISL_9653592, EPI_ISL_9653595, EPI_ISL_9653616, EPI_ISL_9653619, EPI_ISL_9653626, EPI_ISL_9653630, EPI_ISL_9653631, EPI_ISL_9653640, EPI_ISL_9653643, EPI_ISL_9653645, EPI_ISL_9653646, EPI_ISL_9653657, EPI_ISL_9653662, EPI_ISL_9653663, EPI_ISL_9653666, EPI_ISL_9653668, EPI_ISL_9653670, EPI_ISL_9653677, EPI_ISL_9653679, EPI_ISL_9653687, EPI_ISL_9653691, EPI_ISL_9653703, EPI_ISL_9653706, EPI_ISL_9653708, EPI_ISL_9653718, EPI_ISL_9653719, EPI_ISL_9653722, EPI_ISL_9653728, EPI_ISL_9653729, EPI_ISL_9653731, EPI_ISL_9653740, EPI_ISL_9653741, EPI_ISL_9653745, EPI_ISL_9653746, EPI_ISL_9653749, EPI_ISL_9653750, EPI_ISL_9653755, EPI_ISL_9653756, EPI_ISL_9653760, EPI_ISL_9653764, EPI_ISL_9653768, EPI_ISL_9653769, EPI_ISL_9653771, EPI_ISL_9653774, EPI_ISL_9653777, EPI_ISL_9653781, EPI_ISL_9653787, EPI_ISL_9653801, EPI_ISL_9653805, EPI_ISL_9653809, EPI_ISL_9653813, EPI_ISL_9653820, EPI_ISL_9653829, EPI_ISL_9653832, EPI_ISL_9653835, EPI_ISL_9653838, EPI_ISL_9653839, EPI_ISL_9653844, EPI_ISL_9653846, EPI_ISL_9653861, EPI_ISL_9653862, EPI_ISL_9653864, EPI_ISL_9653867, EPI_ISL_9653873, EPI_ISL_9653877, EPI_ISL_9653878, EPI_ISL_9653885, EPI_ISL_9653886, EPI_ISL_9653891, EPI_ISL_9653894, EPI_ISL_9653895, EPI_ISL_9653897, EPI_ISL_9653898, EPI_ISL_9653902, EPI_ISL_9653905, EPI_ISL_9653919, EPI_ISL_9653924, EPI_ISL_9653925, EPI_ISL_9653931, EPI_ISL_9653933, EPI_ISL_9653935, EPI_ISL_9653940, EPI_ISL_9653941, EPI_ISL_9653944, EPI_ISL_9653947, EPI_ISL_9653948, EPI_ISL_9653951, EPI_ISL_9653958, EPI_ISL_9653959, EPI_ISL_9653969, EPI_ISL_9653972, EPI_ISL_9653973, EPI_ISL_9653974, EPI_ISL_9653977, EPI_ISL_9653981, EPI_ISL_9653989, EPI_ISL_9653997, EPI_ISL_9653999, EPI_ISL_9654000, EPI_ISL_9654006, EPI_ISL_9654012, EPI_ISL_9654013, EPI_ISL_9654014, EPI_ISL_9654018, EPI_ISL_9654020, EPI_ISL_9654120, EPI_ISL_9654123, EPI_ISL_9654133, EPI_ISL_9654155, EPI_ISL_9654157, EPI_ISL_9654166, EPI_ISL_9654167, EPI_ISL_9654182, EPI_ISL_9654188, EPI_ISL_9654191, EPI_ISL_9654193, EPI_ISL_9654194, EPI_ISL_9654201, EPI_ISL_9654216, EPI_ISL_9654217, EPI_ISL_9654219, EPI_ISL_9654220, EPI_ISL_9654223, EPI_ISL_9654224, EPI_ISL_9654229, EPI_ISL_9654236, EPI_ISL_9654247, EPI_ISL_9654248, EPI_ISL_9654249, EPI_ISL_9654252, EPI_ISL_9654256, EPI_ISL_9654264, EPI_ISL_9654266, EPI_ISL_9654274, EPI_ISL_9654278, EPI_ISL_9654280, EPI_ISL_9654281, EPI_ISL_9654283, EPI_ISL_9654288, EPI_ISL_9654293, EPI_ISL_9654304, EPI_ISL_9654310, EPI_ISL_9654321, EPI_ISL_9654332, EPI_ISL_9654333, EPI_ISL_9654335, EPI_ISL_9654337, EPI_ISL_9654342, EPI_ISL_9654343, EPI_ISL_9654351, EPI_ISL_9654352, EPI_ISL_9654353, EPI_ISL_9654357, EPI_ISL_9654366, EPI_ISL_9654368, EPI_ISL_9654370, EPI_ISL_9654374, EPI_ISL_9654377, EPI_ISL_9654379, EPI_ISL_9654383, EPI_ISL_9654385, EPI_ISL_9654387, EPI_ISL_9654388, EPI_ISL_9654390, EPI_ISL_9654391, EPI_ISL_9654392, EPI_ISL_9654393, EPI_ISL_9654394, EPI_ISL_9654398, EPI_ISL_9654418, EPI_ISL_9654420, EPI_ISL_9654423, EPI_ISL_9654436, EPI_ISL_9654440, EPI_ISL_9654448, EPI_ISL_9654450, EPI_ISL_9654451, EPI_ISL_9654455, EPI_ISL_9654457, EPI_ISL_9654466, EPI_ISL_9654467, EPI_ISL_9654471, EPI_ISL_9654472, EPI_ISL_9654483, EPI_ISL_9654488, EPI_ISL_9654489, EPI_ISL_9654493, EPI_ISL_9654495, EPI_ISL_9654497, EPI_ISL_9654503, EPI_ISL_9654508, EPI_ISL_9654510, EPI_ISL_9654511, EPI_ISL_9654513, EPI_ISL_9654514, EPI_ISL_9654530, EPI_ISL_9654532, EPI_ISL_9654538, EPI_ISL_9654544, EPI_ISL_9654550, EPI_ISL_9654554, EPI_ISL_9654556, EPI_ISL_9654568, EPI_ISL_9654569, EPI_ISL_9654570, EPI_ISL_9654571, EPI_ISL_9654572, EPI_ISL_9654575, EPI_ISL_9654580, EPI_ISL_9654588, EPI_ISL_9654590, EPI_ISL_9654595, EPI_ISL_9654599, EPI_ISL_9654610, EPI_ISL_9654612, EPI_ISL_9654621, EPI_ISL_9654623, EPI_ISL_9654626, EPI_ISL_9654629, EPI_ISL_9654633, EPI_ISL_9654634, EPI_ISL_9654638, EPI_ISL_9654639, EPI_ISL_9654644, EPI_ISL_9654666, EPI_ISL_9654672, EPI_ISL_9654675, EPI_ISL_9654676, EPI_ISL_9654680, EPI_ISL_9654682, EPI_ISL_9654692, EPI_ISL_9654695, EPI_ISL_9654706, EPI_ISL_9654709, EPI_ISL_9654717, EPI_ISL_9654720, EPI_ISL_9654721, EPI_ISL_9654724, EPI_ISL_9654726, EPI_ISL_9654728, EPI_ISL_9654730, EPI_ISL_9654735, EPI_ISL_9654741, EPI_ISL_9654745, EPI_ISL_9654750, EPI_ISL_9654759, EPI_ISL_9654761, EPI_ISL_9654774, EPI_ISL_9654776, EPI_ISL_9654783, EPI_ISL_9654878, EPI_ISL_9654970, EPI_ISL_9654980, EPI_ISL_9654981, EPI_ISL_9654985, EPI_ISL_9654986, EPI_ISL_9654989, EPI_ISL_9654998, EPI_ISL_9655002, EPI_ISL_9655004, EPI_ISL_9655010, EPI_ISL_9655025, EPI_ISL_9655028, EPI_ISL_9655031, EPI_ISL_9655032, EPI_ISL_9655050, EPI_ISL_9655055, EPI_ISL_9655069, EPI_ISL_9655072, EPI_ISL_9655074, EPI_ISL_9655075, EPI_ISL_9655080, EPI_ISL_9655087, EPI_ISL_9655088, EPI_ISL_9655089, EPI_ISL_9655091, EPI_ISL_9655100, EPI_ISL_9655103, EPI_ISL_9655109, EPI_ISL_9655118, EPI_ISL_9655122, EPI_ISL_9655124, EPI_ISL_9655125, EPI_ISL_9655126, EPI_ISL_9655127, EPI_ISL_9655129, EPI_ISL_9655130, EPI_ISL_9655134, EPI_ISL_9655136, EPI_ISL_9655137, EPI_ISL_9655138, EPI_ISL_9655145, EPI_ISL_9655148, EPI_ISL_9655149, EPI_ISL_9655155, EPI_ISL_9655160, EPI_ISL_9655162, EPI_ISL_9655170, EPI_ISL_9655172, EPI_ISL_9655174, EPI_ISL_9655177, EPI_ISL_9655178, EPI_ISL_9655189, EPI_ISL_9655190, EPI_ISL_9655191, EPI_ISL_9655193, EPI_ISL_9655197, EPI_ISL_9655198, EPI_ISL_9655210, EPI_ISL_9655212, EPI_ISL_9655215, EPI_ISL_9655219

Accession ID	Originating Laboratory	Submitting Laboratory	Authors
see above	Department of Bacteria, Parasites and Fungi, Statens Serum Institut, Copenhagen, Denmark	Statens Serum Institut Bioinformatics and Microbial Genomics	Danish Covid-19 Genome Consortium
